# Sizing the role of London dispersion in the dissociation of all-*meta tert*-butyl hexaphenylethane†
†Electronic supplementary information (ESI) available: Experimental procedures, spectra and computational details. See DOI: 10.1039/c6sc02727j



**DOI:** 10.1039/c6sc02727j

**Published:** 2016-08-23

**Authors:** Sören Rösel, Ciro Balestrieri, Peter R. Schreiner

**Affiliations:** a Institute of Organic Chemistry , Justus-Liebig University , Heinrich-Buff-Ring 17 , 35392 Giessen , Germany . Email: prs@uni-giessen.de; b Department of Chemical Sciences , University of Padova , Via Marzolo 1 , 35131 Padova , Italy

## Abstract

The structure and dynamics of enigmatic hexa(3,5-di-*tert*-butylphenyl)ethane was characterized *via* NMR spectroscopy for the first time. Our variable temperature NMR analysis demonstrates an enthalpy–entropy compensation that results in a vanishingly low dissociation energy (Δ*G*298d = –1.60(6) kcal mol^–1^). An *in silico* study of increasingly larger all-*meta* alkyl substituted hexaphenylethane derivatives (Me, ^i^Pr, ^*t*^Bu, Cy, 1-Ad) reveals a non-intuitive correlation between increased dimer stability with increasing steric crowding. This stabilization originates from London dispersion as expressed through the increasing polarizability of the alkyl substituents. Substitution with conformationally flexible hydrocarbon moieties, *e.g.*, cyclohexyl, introduces large unfavourable entropy contributions. Therefore, using rigid alkyl groups like *tert*-butyl or adamantyl as dispersion energy donors (DED) is essential to help stabilize extraordinary bonding situations.

## Abstract 2

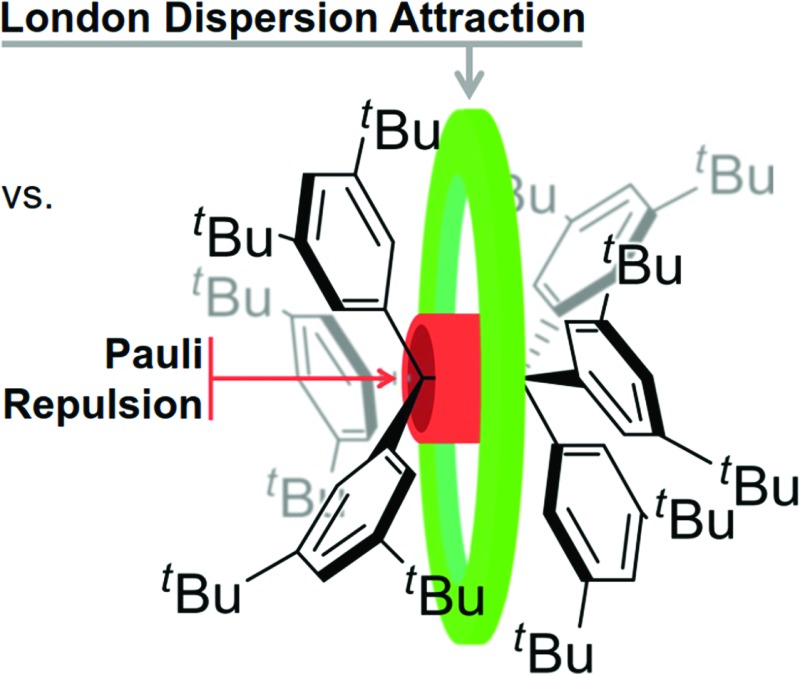
The structure and dynamics of enigmatic hexa(3,5-di-*tert*-butylphenyl)ethane was characterized *via* NMR spectroscopy for the first time.

Hexaphenylethane (HPE; H-**1**_2_) was long assumed to have been synthesized by Gomberg in 1900 in the context of preparing the first organic free radical, the triphenylmethyl radical (H-**1·**) (Scheme 1).^1,2^ However, it was shown in 1968 that H-**1**_2_ rather corresponded to quinoid H-**2**.^3^ While steric repulsion between the phenyl moieties was readily made responsible for instability of H-**1**_2_, the introduction of bulky *tert*-butyl groups in all *meta* positions, which should lead to significantly higher steric crowding, resulted in isolable hexa(3,5-di-*tert*-butylphenyl)ethane (^*t*^Bu-**1**_2_) that could even be characterized through X-ray crystal diffraction.^4–6^ Clearly, this questions our (conceptual) understanding of the balance of steric repulsion *vs.* noncovalent attraction, and a dispersion energy donor (DED) concept employing large alkyl groups may be invoked to rationalize such surprising bonding situations.^7^ The classic HPE system therefore seems well suited to probe this concept. Here we report on the challenging re-synthesis and the first NMR-spectroscopic characterization of ^*t*^Bu-**1**_2_ as well on density functional theory (DFT) computations to gauge the fine balance between Pauli repulsion and London dispersion (LD) attraction.

**Scheme 1:**
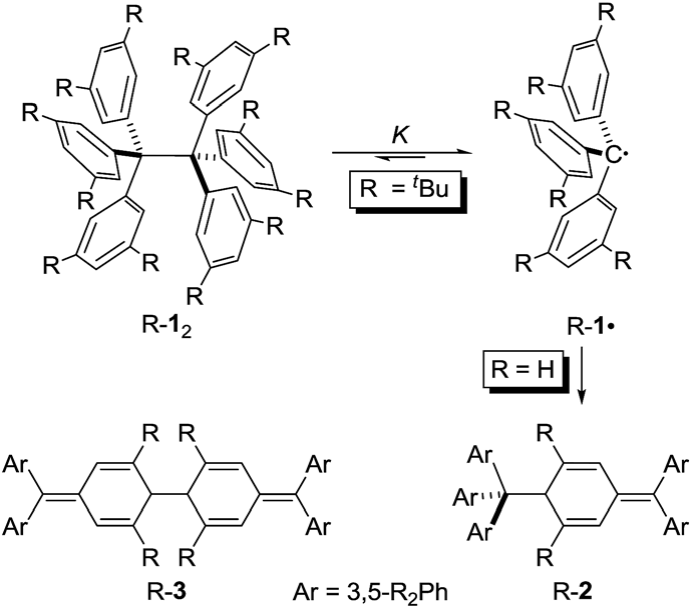
The Jacobsen–Nauta structure H-**2** is exclusively observed for the parent trityl radical H-**1·**. All-*meta tert*-butyl substitution leads to equilibration between the ^*t*^Bu-**1·** and dimeric ^*t*^Bu-**1**_2_ in solution.

It is quite remarkable that the structural proposal for H-**1**_2_ prevailed for almost 70 years because Gomberg himself noted that “Der Körper zeigt in höchstem Maasse die Eigenschaften einer ungesättigten Verbindung” (the substance shows pronounced properties of unsaturation) and readily adds oxygen and halogens.^1^ He concluded: “Die oben mitgetheilten experimentellen Ergebnisse zwingen mich zu der Annahme, dass in dem ‘ungesättigten Kohlenwasserstoff’ das Radical Triphenylmethyl vorliegt” (the disclosed experimental findings force me to assume that the “unsaturated hydrocarbon” presents the radical triphenylmethyl).^1^ In addition, the neat colourless hydrocarbon was found to be in equilibrium with a coloured form in solution.^8^ This triggered an intense discussion regarding the proper structure of triphenylmethyl where, apart from the highly symmetric HPE structure,^9^ quinoid structures H-**2** ^10^ and H-**3** ^11^ were suggested (Scheme 1). In an early review Gomberg described an equilibrium between an associated and a free radical, the monoquinoidal structure, and HPE.^12^ In the following years an equilibrium between H-**1·** and H-**1**_2_ suggested first by Flürscheim^13,14^ was gradually accepted and the quinoid structures vanished from the discussion, despite Gomberg mentioning later that a quinoid tautomer of H-**1·** causes the yellow colour of the solution.^15^

Notwithstanding the fact that some had misgivings regarding the correct structure of HPE,^16^ dimer H-**1**_2_ was generally accepted until Nauta *et al.* reinvestigated it utilizing ^1^H-NMR and IR spectroscopies. These studies demonstrated that HPE adopted Jacobsen's monoquinoidal dimer H-**2**.^3^ The first computational investigation of H-**1**_2_ was reported by Mislow *et al.* utilizing empirical force field (EFF) computations that revealed a very long central *R*_CC_ of 1.64 Å in H-**1**_2_ due to the repulsion between the trityl moieties.^17^ The *D*_3_ symmetric structure was found to be 2.6 kcal mol^–1^ more stable than the *S*_6_ structure. A QM/MM (ONIOM) study of 2002 favoured the *S*_6_ form and gave a BDE = 16.6 kcal mol^–1^ for H-**1**_2_ and a central bond length of 1.72 Å.^18^

The first isolation of an unbridged HPE derivative was achieved by Rieker in 1978 through the introduction of *tert*-butyl groups in all 2- and 6-positions onto the Schlenk (tri(4-biphenyl)-methyl) radical.^4,5^ The previously reported introduction of ^*t*^Bu groups onto the trityl radical H-**1·** yielded tris(3,5-di-*tert*-butylphenyl)methyl (^*t*^Bu-**1·**) that was found to be monomeric in benzene^19^ and Rieker concluded that […] steric hindering of formation of the Jacobson–Nauta structure [R-**2**] by incorporation of bulky groups [in *meta* and/or *para* positions], [R-**1·**] must then either be persistent as the monomer—or dimerize to [R-**1**_2_].^4,5^ Indeed, “poor, colourless crystals” could be grown from the orange cyclohexane solution consisting of the first unbridged HPE derivative 1,1,1,2,2,2-hexakis(2,6-di-*tert*-butyl-[1,1′-biphenyl]-4-yl)ethane (^*t*^Bu-**4**_2_).^4,5^ The dimer crystallized in *S*_6_ symmetry with an astonishingly short experimental *R*_CC_ of only 1.47 Å. Mislow responded with improved EFF and MNDO SCF-MO computations that gave a *R*_CC_ between 1.62 and 1.68 Å.^20^ Finally, a central bond length of 1.67 Å was determined from a new crystal structure of ^*t*^Bu-**1**_2_;^6^ magic angle spinning (MAS) nutation experiments gave *R*_CC_ = 1.64–1.65 Å,^21^ confirming the earlier bond distance and refuting Rieker's structure. Our recent computational reinvestigation of the “HPE riddle” with the modern DFT implementations confirmed these results.^22^

Both H-**2** and ^*t*^Bu-**1**_2_ afford yellowish-orange, EPR active solutions, in line with very low computed dissociation energies.^22^ The equilibrium constants for radical formation form the dimers should therefore be accessible by variable temperature NMR spectroscopy despite Rieker's notion that “Because of the high degree of dissociation and the sensitivity of the radical towards traces of oxygen no definite proof of the structure of [^*t*^Bu-**1**_2_] can be obtained with ^1^H-NMR, ^13^C-NMR, and mass spectroscopy, […]”.^4^ The stronger magnetic fields available with current NMR spectrometers together with sophisticated techniques encouraged us to investigate the dissociation/association equilibrium of ^*t*^Bu-**1**_2_.

The precursor tris(3,5-di-*tert*-butylphenyl)methyl chloride (**5**) was synthesized from benzene (**6**) *via* Friedel–Crafts alkylation with ^*t*^BuCl, *ipso*-bromination followed by lithiation at –78 °C, subsequent triple addition to diethylcarbonate and chlorination of the central methyl carbon with acetyl chloride (Scheme 2). The classic reduction of **5** to ^*t*^Bu-**1·** with silver in benzene^1,21,23^ under meticulous exclusion of oxygen was slow and mainly yielded bis(tri(3,5-di-*tert*-butylphenyl)methyl) peroxide (**7**). Even silver derived from reduction of silver nitrate seemed to introduce a significant amount of oxygen. Using the zinc–copper couple instead gave rapid quantitative conversion. The reduction in benzene-*d*_6_ resulted in an orange solution with a ^1^H-NMR spectrum that contained, besides several sharp signals, a very broad peak at *δ* = 1.88 ppm with a total width of 3 ppm (Fig. 1a, top). The sharp signals were assigned to **7**, tris(3,5-di-*tert*-butylphenyl)methane (**8**) and tris(3,5-di-*tert*-butylphenyl)methanol (**9**) by comparison with the NMR spectra of the pure compounds (*cf.* ESI†). The sample gave an EPR signal featuring the known spin pattern.^19,24^ This, together with the known NMR lifetime broadening of organic radicals^25^ as well as the *r* ^–6^ line width dependence of the NMR signal with respect to the radical centroid in the molecule,^26^ made it possible to identify the broad peak as the *tert*-butyl group hydrogen resonance of ^*t*^Bu-**1·**.

**Scheme 2:**
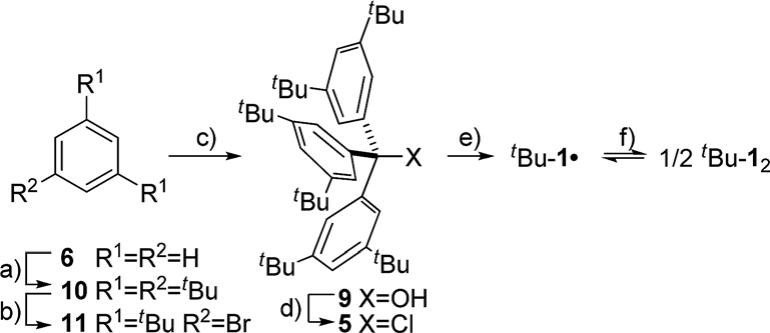
Synthesis of ^*t*^Bu-**1**_2_: (a) ^*t*^BuCl, AlCl_3_, (–40) → (–15) °C, 81% (b) Br_2_, Fe, CCl_4_, rt, 86% (c) 1. ^*t*^BuLi, Et_2_O, –78 °C → rt 2. (EtO)_2_CO, 78% (d) AcCl, *n*-hexane, 95% (e) Zn(Cu), C_6_D_6_ or C_6_D_12_, rt (f) only in C_6_D_12_.

**Fig. 1:**
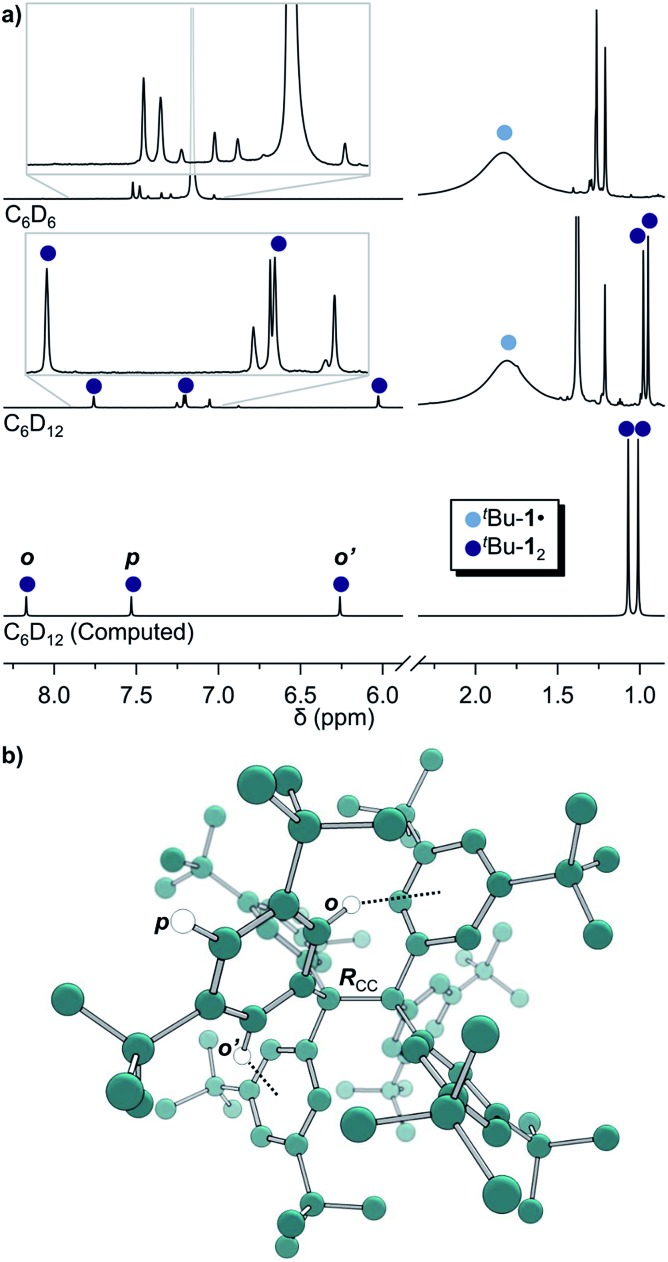
(a) ^1^H-NMR of ^*t*^Bu-**1·** in benzene-*d*_6_ (top) in equilibrium with ^*t*^Bu-**1**_2_ in cyclohexane-*d*_12_ (middle) and the computed spectrum^(ii)^ of ^*t*^Bu-**1**_2_ (bottom); see ESI† for full spectra. (b) The structure of ^*t*^Bu-**1**_2_ for NMR computations. *R*_cc_: exp.^4^ 1.67(3) Å, comp. 1.662 Å. ^(ii)^(B3LYP-D3(BJ)/6-31G(d,p)/C-PCM:cyclohexane).

The *in situ* radical generation in cylohexane-*d*_12_ through reduction with Zn(Cu) is slower as compared to benzene-*d*_6_ but gave the same orange solution and a similar ^1^H-NMR spectrum with five additional peaks at *δ* = 7.75, 7.19, 6.02, 0.98 and 0.95 ppm in a ratio of 1 : 1 : 1 : 9 : 9, respectively (Fig. 1a, middle). This indicates a highly symmetric structure that would be incompatible with a less symmetric quinoid structure for which an allylic resonance at 5 ppm and a vinylic resonance at 6.4 ppm in a 1 : 2 ratio would be expected.^3,27^ The spectrum is also in excellent agreement with the B3LYP-D3(BJ)/6-31G(d,p) C-PCM:cyclohexane computed spectrum of ^*t*^Bu-**1**_2_ (Fig. 1a, bottom).

This first ^1^H-NMR spectrum of an unbridged HPE derivative features a significant chemically inequivalence of the *ortho* or *meta* phenyl nuclei (for the ^13^C-NMR spectrum see the ESI†). The fast inversion of the local helical chirality of an individual trityl group through a two-ring flip mechanism^28–30^ that makes the two *ortho* or *meta* nuclei appear as a single signal at rt is blocked in ^*t*^Bu-**1**_2_. That is, one edge of the phenyl ring points outside and the other inside towards the *S*_6_ axis. While the phenyl groups in the X-ray crystal structure are indistinguishable due to the superposition of four disordered ^*t*^Bu-**1**_2_ arrangements,^6^ the computed structure (Fig. 1b) nicely features this structural difference between inside (o′) and outside (o) protons (Fig. 1a, bottom). Fortunately, the equilibrium between the ^*t*^Bu-**1·** radical and ^*t*^Bu-**1**_2_ revealed a strong temperature dependence (Fig. 2a), allowing us to determine parameters to calculate the equilibrium constant *K* at a particular temperature through integration of
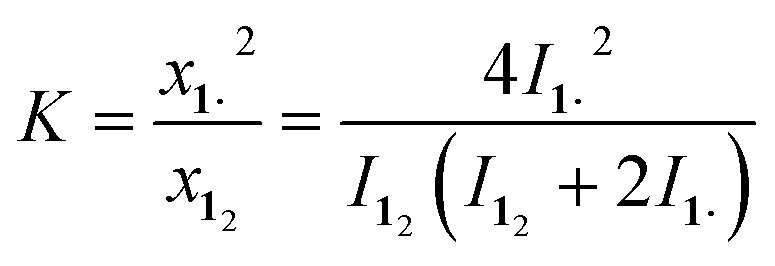
with *I* being the ^1^H-NMR integrals, *x* the molar fraction, **1·** the radical monomer and **1**_2_ the dimer (for the derivation see the ESI†).

**Fig. 2:**
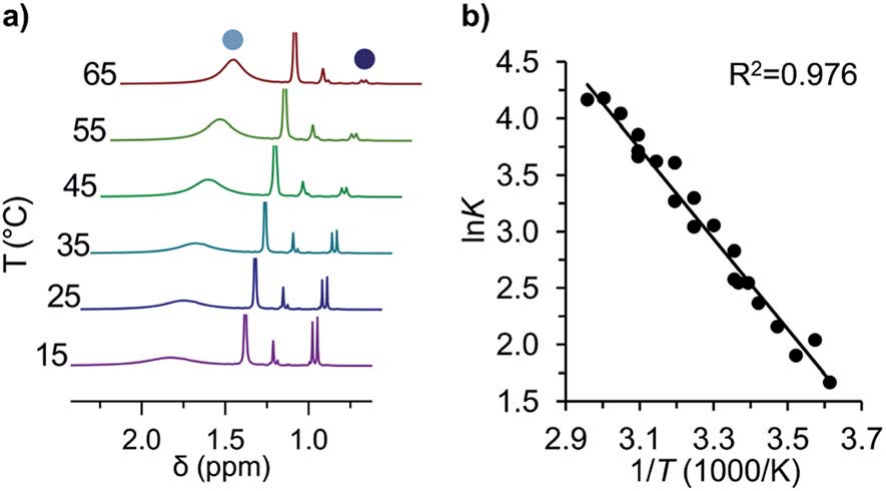
(a) Variable temperature ^1^H-NMR spectra of the equilibrium between ^*t*^Bu-**1·** (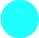) and ^*t*^Bu-**1**_2_ (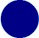) and (b) the corresponding van't Hoff plot (see ESI† for complete spectra).

The van't Hoff plot (Fig. 2b) shows a good linear correlation and Δ*G*298d = –1.60(6) kcal mol^–1^ for the homolysis of ^*t*^Bu-**1**_2_ into two radicals reveals a very low dissociation energy. This Δ*G*298d value agrees reasonably well with computational predictions of Δ*G*298d = –3 to +1 kcal mol^–1^ in cyclohexane with PWPB95-D3/TZV(2d,2p),^22^ and it is in the range of the weakest experimentally determined Δ*G*298d of –0.2(1) kcal mol^–1^ for a C–C single bond reported for the 2,6-di-*tert*-butyl-4-methoxyphenoxyl dimer (**12**, Fig. 3).^31^ As found for **12**, Δ*H*298d of 6.1(5) kcal mol^–1^ is outbalanced by *T*Δ*S*298d = 6.3 kcal mol^–1^ (298 K). We find a similar enthalpy–entropy compensation for ^*t*^Bu-**1**_2_ with Δ*H*298d = 7.94(3) kcal mol^–1^ and *T*Δ*S*298d = 9.5(3) kcal mol^–1^ (298 K); this compares well with other weakly bonded hydrocarbons (*cf.* ESI†).

**Fig. 3:**
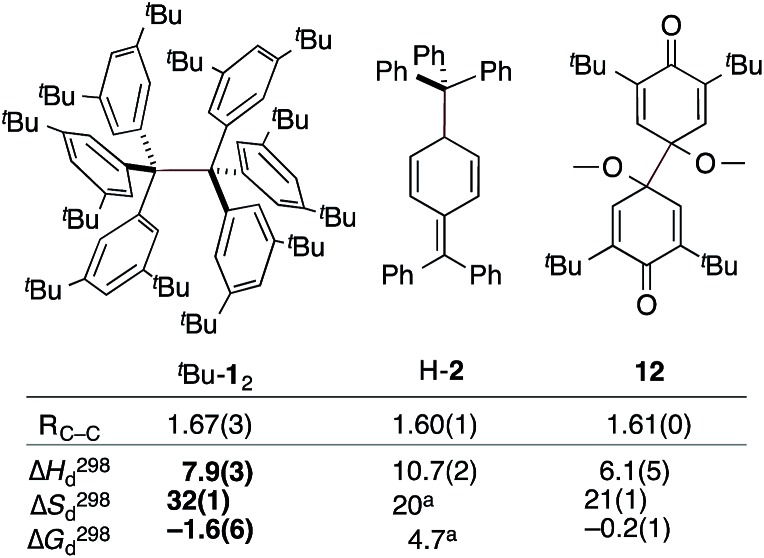
The experimental bond lengths *R*_CC_ do not correlate well with the bond dissociation enthalpy, entropy, and free energy. Bond lengths given in Å, enthalpies and energies in kcal mol^–1^, entropies in cal K^–1^ mol^–1^. Bold numbers are from this work. ^a^No error given. ^*t*^Bu-**1**_2_;^6^ H-**2**;^32,33^ **12**.^31^

The dissociation enthalpy of ^*t*^Bu-**1**_2_ is 2.8 kcal mol^–1^ smaller than the 10.7(2) kcal mol^–1^ Δ*H*298d of H-**2**,^32^ while the entropy contribution in ^*t*^Bu-**1**_2_ is significantly larger (*T*Δ*S*298d = 6.0 kcal mol^–1^). This is not surprising because covalent ^*t*^Bu-**1**_2_ requires much more ordering of the ^*t*^Bu-groups for optimizing the LD interactions, leading to a large, counteracting Δ*S* contribution. This is likely to be a general phenomenon for structures with large LD contributions because the DEDs often require optimal structural alignment for maximizing the LD interactions.

Differences in LD contributions between gas phase computations and solution experiments were rationalized with a di-*n*-hexyl substituted Wilcox-type balance by Cockroft and co-workers.^34,35^ Another approach outlined here is the use of the radical dimerization energies to determine the effect of DEDs on chemical equilibria. While large, polarizable electron donor substituents stabilize the dimers and the free radicals to different degrees, the radicals are stabilized mostly by conjugation. Depending on which radical stability scale one uses, *meta* alkyl substitution of benzyl radicals may be slightly stabilizing^36–38^ or destabilizing,^39,40^ but it is small in all cases. That is, the increased stability of ^*t*^Bu-**1**_2_ relative to parent H-**1**_2_ must originate from the LD contributions of the ^*t*^Bu groups.

Does the common measure for bond strengths, the bond dissociation energy (BDE), still apply here? BDEs were initially developed on the basis of small (often diatomic) molecules for which LD is a minor component and the overall bond stability reflects the covalent bond strength. For larger, crowded molecules as R-**1**_2_ the covalent bond increasingly is influenced by Pauli repulsion and LD attraction. While for H-**1**_2_ LD attenuates about half of the repulsion, in ^*t*^Bu-**1**_2_ the increased repulsion must be compensated by LD to a higher degree.^41^ The difference between the Δ*G*298d computed in the gas phase (13.7 kcal mol^–1^)^22^ and the experimental Δ*G*298d value measured here (–1.6 kcal mol^–1^) can be taken as a rough measure of the attenuation of the LD interactions in solution. As the corrections to account for LD effects are large (*ca.* 60 kcal mol^–1^),^22^ a significant LD contribution must therefore persist also in solution. In light of such large LD contributions (*cf.* Table S3†), the BDE does not seem to be a good measure for the covalent bond strength of the central bond.

As large-scale computations including properly computed entropy terms can reproduce Δ*G*298d reasonably well,^22^ we used more approximate DFT computations to make some qualitative predictions regarding other DEDs in R-**1**_2_ as model (Fig. 4). We employed the commonly used alkyl groups Me, *^i^*Pr, ^*t*^Bu, Cy, and 1-Ad as DEDs. With up to 356 atoms in Ad-**1**_2_ only DFT methods are currently feasible; these computations still take a very long time because of the very flat potentials caused by the rotations of these groups. The B3LYP^42,43^ functional with the Becke–Johnson damped dispersion correction D3(BJ) introduced by Grimme^44,45^ as well as the hybrid GGA functional M06-2X^46^ of Truhlar *et al.* were used in conjunction with a cc-pVDZ basis set (Table S3†).^47^ In the HPE derivatives the symmetry was restricted to the *S*_6_ point group^6,18^ (as experimentally observed for ^*t*^Bu-**1**_2_) while the radical monomers ^*t*^Bu-**1·** were assumed to have *C*_3_ symmetry. The C-PCM model was employed to account for bulk solvent effects.^48^

**Fig. 4:**
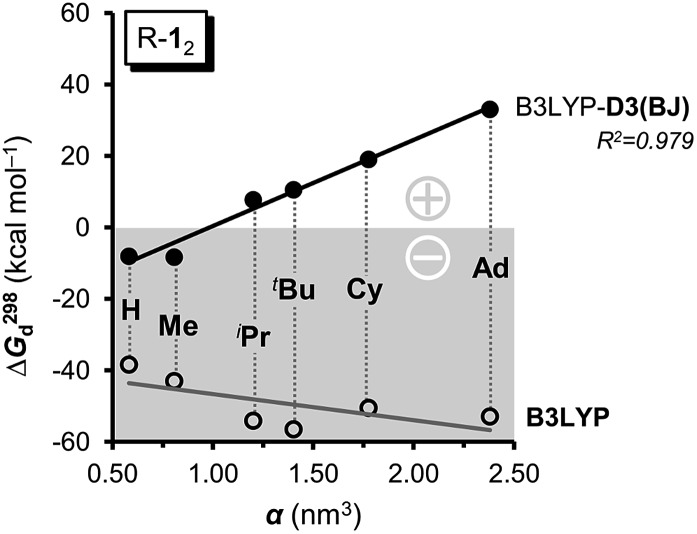
The computed free dissociation energies Δ*G*298d including dispersion corrections () correlate very well with the polarizability *α* and therefore the LD contributions of the R groups (R = H, Me, ^i^Pr, ^*t*^Bu, Cy, Ad) and gain stability with size. Neglecting LD leads to negative Δ*G*298d (○), bearing the opposite, negative trend. Basis set: cc-pVDZ.

First of all, the B3LYP-D3(BJ) and M06-2X results with a cc-pVDZ basis set show the same general trends but diverge significantly as the size of the DED increases (Table S3†). The M06-2X functional is in good agreement for ^*t*^Bu-**1**_2_ but the trend is not as smooth as with B3LYP-D3(BJ).^49^ Still, the LD contributions are not merely minor corrections but are quite large, exceeding the magnitude of carbon–carbon bond energies for R = 1-Ad (*cf.* ESI†). There is a large dispersion correction even in the hitherto unobserved parent system H-**1**_2_ that is predicted to dissociate with a Δ*G*298d of less than –5 kcal mol^–1^.

Non-covalent interactions can be visualized by plotting the reduced density gradient in regions of low electron density (NCI plot).^50^ This enables a qualitative analysis of the balance between repulsive and attractive contact areas. The NCI plots (Fig. 5) of H-**1**_2_, ^*t*^Bu-**1**_2_ and Ad-**1**_2_ visually support the general trend obtained for Δ*G*298d. Parent H-**1**_2_ features repulsive (red) areas between phenyl moieties within and between the trityl groups and few attractive (C–H···π) interactions (blue). Substitution with ^*t*^Bu groups leads to significant growth of both attractive and repulsive regions in the periphery of the HPE core structure but scattered weak interactions (green) also appear. Finally, Ad-**1**_2_ clearly shows a dense, weakly interacting region between the substituted trityl moieties. Hence, although the 1-adamantly moieties are even larger than the ^*t*^Bu groups, they exhibit a huge noncovalent contact area that maximizes attractive LD interactions visually outweighing the repulsive regions.

**Fig. 5:**
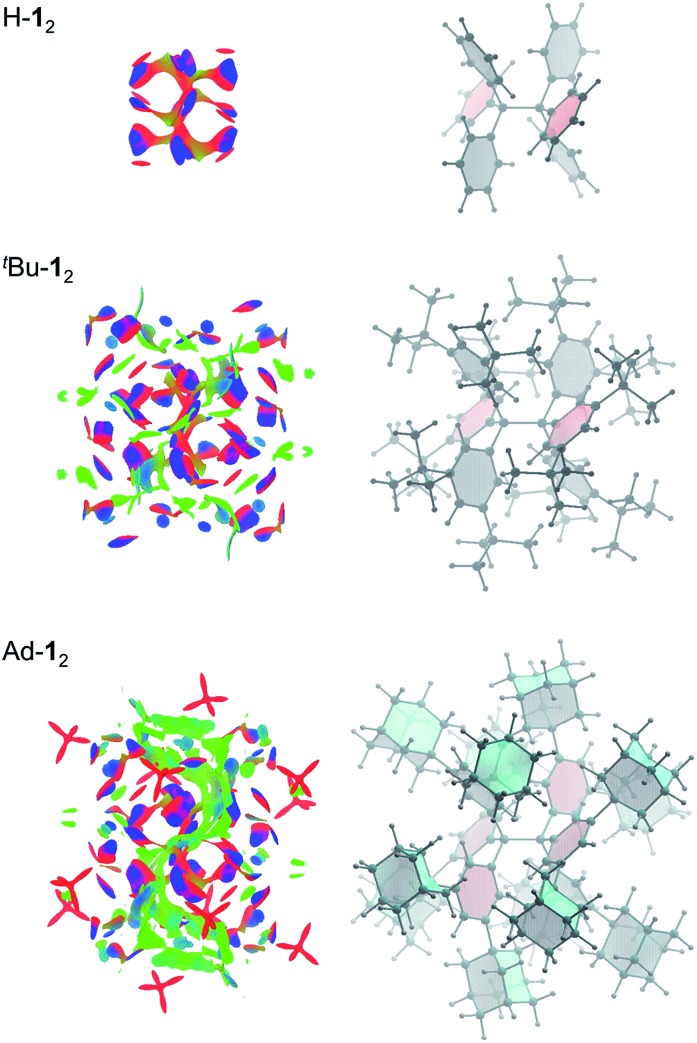
Non-covalent interaction (NCI) plots (*s* = 0.5 au/–0.01 < *ρ* < +0.01 au) are depicted separately on the left from the molecular structure on the right for clarity. Repulsion is colour-coded red, “strong” attraction blue and weak interactions in green.

## Conclusions

We re-synthesized ^*t*^Bu-**1**_2_, a member of the elusive class of HPE derivatives, and were able to obtain its dynamic NMR spectrum in equilibrium with the corresponding free radical ^*t*^Bu-**1·**. The dissociation free energy of Δ*G*298d (exp.) = –1.60(6) kcal mol^–1^ confirms computational pre-dictions and is composed of nearly equally large enthalpy and entropy contributions at 298 K. Computations suggest that substitutions with isopropyl and cyclohexyl DEDs also are accompanied by large unfavourable entropy contributions so that it is imperative to use rigid hydrocarbon moieties as DEDs. As a consequence, the introduction of sterically even more demanding 1-adamantyl substituents predicts an even further reduction the central bond length and a significant increase in stability as compared to ^*t*^Bu-**1**_2_. Counterintuitively, Ad-**1**_2_ should therefore be an isolable HPE derivative despite the use of even bulkier groups. Hence, large, highly polarizable and rigid hydrocarbon moieties are the most effective DEDs.
