# Supplementary material for: Sizing the role of London dispersion in the dissociation of all-meta tert-butyl hexaphenylethane
Source: Chem Sci. 2016 Aug 23;8(1):405–10. doi: 10.1039/c6sc02727j (PMC5365070; doi:10.1039/c6sc02727j)
Supplement: Supplementary file 1 [file SC-008-C6SC02727J-s001.pdf]

Electronic Supplementary Material (ESI) for Chemical Science.
This journal is © The Royal Society of Chemistry 2016

# Sizing the Role of London Dispersion in the Dissociation of all-*meta* *tert*-Butyl Hexaphenylethane

S. Rösel,[a] C. Balestrieri,[b] and P. R. Schreiner*[a]

[a)] Institute of Organic Chemistry, Justus-Liebig University, Heinrich-Buff-Ring 17, 35392 Giessen (Germany), E-mail: prs@uni-giessen.de.
[b)] Department of Chemical Sciences, University of Padova, Via Marzolo 1, 35131 Padova (Italy).

## Supporting information

### Table of contents

**Sizing the Role of London Dispersion in the Dissociation of all-*meta* *tert*-Butyl Hexaphenylethane** **1**

**Experiments** **2**

Determination of the dissociation energy 2

Experimental procedures 5

Spectra 8

**Computations** **28**

Overview 28

Dissociation Energies 29

Energies 30

Cartesian coordinates 33

*Radical Monomers* *33*

*Dimers* *55*

NMR computations 97

**Estimation of the covalent contribution of the overall dissociation energy** **101**

Estimation from heat of formation 101

Estimation from enthalpy of hydrogenation 101

Estimation from bond vibration 102

Estimation via ISAPT computations 102

Conclusions 102

Computational details for estimations of covalent C–C bond contribution 103

*Dissociation energies, heats of formation and isodesmic reactions* *103*

*Energies* *104*

*Cartesian coordinates* *105*

**Appendix** **107**

Thermodynamic data of C–C bond dissociations equilibria 107

**References** **114**

## Experiments

### Determination of the dissociation energy

The free dissociation energy ($\Delta G_d^{298}$) is connected to the equilibrium constant $K$ *via* the van't Hoff plot. To determine $K$ the dynamic $^1$H-NMR technique was used. For the equilibrium between a radical **1•** and a symmetric dimer **1**$_2$ the equilibrium constant $K$ is defined by

$$K_x = \frac{X_{\mathbf{1}\cdot}^2}{X_{\mathbf{1}_2}} \tag{1}$$

where $X_i$ is the molar fraction. The molar fraction can be derived from the intensity of the corresponding peaks of **1•** and **1**$_2$ because the Intensity $I_i$ is proportional to the amount of substance $n_i$ and the number of hydrogen atoms $H_i$ in the molecule

$$I_i \sim n_i H_i \Rightarrow n_i \sim \frac{I_i}{H_i} \tag{2}$$

The molar fraction becomes

$$X_i = \frac{n_i}{\sum n_i} = \frac{\frac{I_i}{H_i}}{\sum \frac{I_i}{H_i}} \tag{3}$$

Dimer **1**$_2$ carries the double amount of hydrogen atoms than radical **1•** so that the molar fraction becomes

$$X_{\mathbf{1}_2} = \frac{I_{\mathbf{1}_2}}{I_{\mathbf{1}_2} + 2I_{\mathbf{1}\cdot}} \tag{4.1}$$

and

$$X_{\mathbf{1}\cdot} = \frac{2I_{\mathbf{1}\cdot}}{I_{\mathbf{1}_2} + 2I_{\mathbf{1}\cdot}} \tag{4.2}$$

By plugging (4.1) and (4.2) in (1) and final transformation $K$is obtained as

$$K = \frac{4I_{\mathbf{1}\cdot}{}^2}{I_{\mathbf{1}_2}\left(I_{\mathbf{1}_2} + 2I_{\mathbf{1}\cdot}\right)} \tag{5}$$

The van't Hoff plot (6) delivers now the enthalpy $\Delta H_\mathrm{d}$ and the entropy $\Delta S_\mathrm{d}$ of the dissociation as the slope and the y-interception, respectively, by plotting $lnK$ versus $\frac{1}{T}$ with $R$, the gas constant and $T$, the temperature.

$$lnK = -\frac{\Delta H_\mathrm{d}}{R}\left(\frac{1}{T}\right) + \frac{\Delta S_\mathrm{d}}{R} \tag{6}$$

The intensities of the $^1$H-NMR spectra at temperatures from 4 °C to 65 °C (Table S1) where obtained by integration of the corresponding peaks. The final values and errors where determined by linear regression within the van't Hoff plot (Figure S1 and Figure S2).

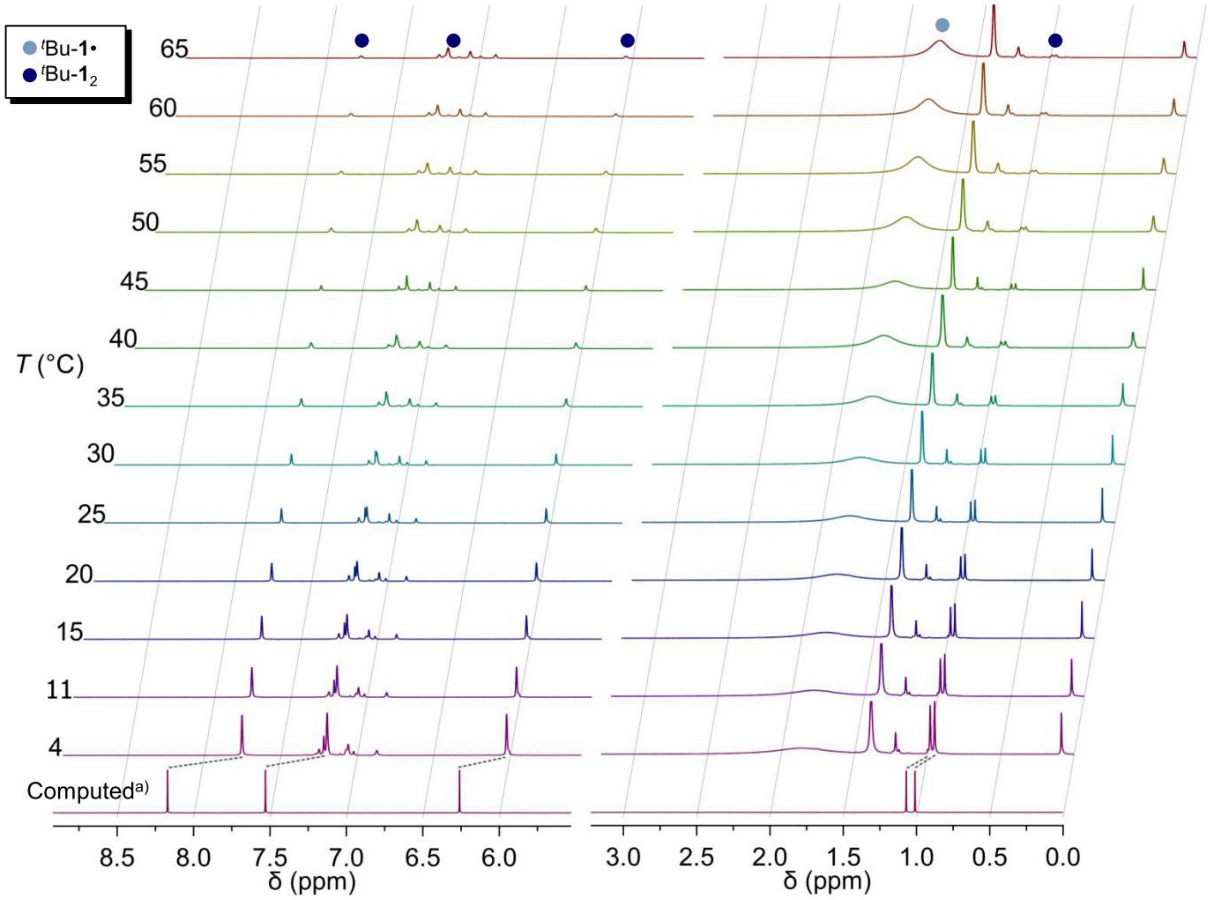


**Figure S1.** The equilibrium constant was obtained by the temperature dependence of the equilibrium between $^{t}$Bu-**1**$_2$ and $^{t}$Bu-**1•**. To determine the molar fraction the broad $^{t}$Bu-**1•** peak at 1.88 ppm and the two $^{t}$Bu-**1**$_2$ at 0.95; 0.98 ppm were used. On the bottom is depicted the [a)]B3LYP-D3(BJ)/6-31G(d,p)/C-PCM:cyclohexane $^{1}$H-NMR spectrum of $^{t}$Bu-**1**$_2$ at 25 °C.

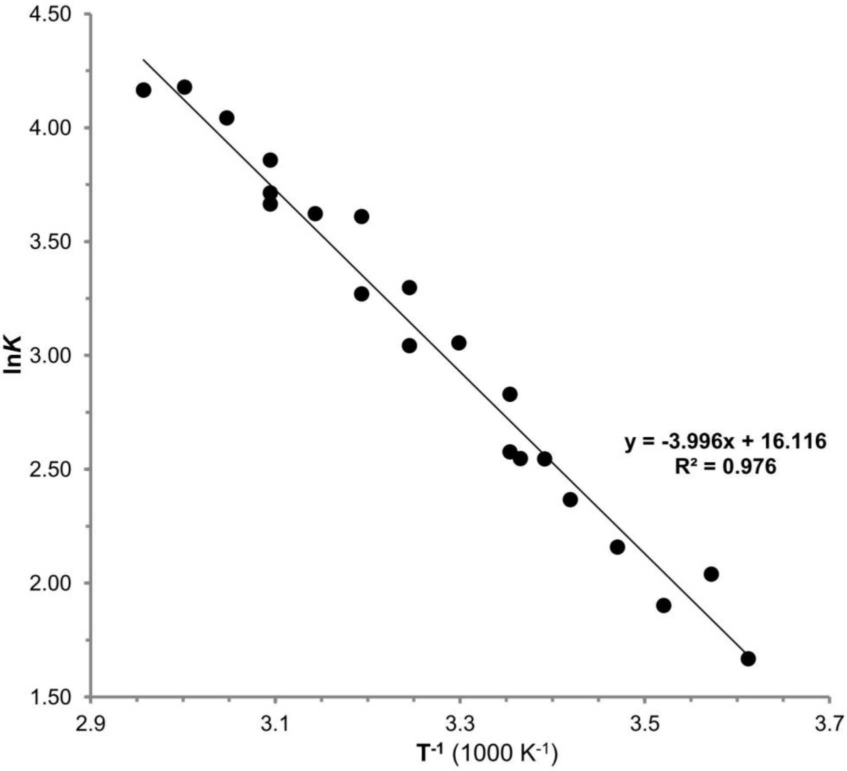


Figure S2. Van't Hoff plot of the equilibrium between $^{t}$Bu-**1**$_2$ and $^{t}$Bu-**1•** derived from VT $^{1}$H-NMR.

**Table S1.** Measured intensities (integrals ∫) of the in Figure S1 indicated peaks and the resulting equilibrium constant *K*.

| ***T*** [°C][a] | ∫( $^t$Bu-**1**•)[b] | ∫($^t$Bu-**1**$_2$)[b] | ln(***K***)[c] |
|---|---|---|---|
| 65.0 | 32.7 | 1 | 4.17 |
| 65.0 | 32.7 | 1 | 4.17 |
| 60.0 | 33.1 | 1 | 4.18 |
| 55.0 | 29.0 | 1 | 4.04 |
| 50.0 | 24.2 | 1 | 3.86 |
| 50.0 | 20.0 | 1 | 3.66 |
| 50.0 | 21.0 | 1 | 3.71 |
| 45.0 | 19.2 | 1 | 3.62 |
| 40.0 | 19.0 | 1 | 3.61 |
| 40.0 | 13.6 | 1 | 3.27 |
| 35.0 | 14.0 | 1 | 3.30 |
| 35.0 | 11.0 | 1 | 3.04 |
| 30.0 | 11.1 | 1 | 3.06 |
| 25.0 | 8.94 | 1 | 2.83 |
| 25.0 | 7.04 | 1 | 2.58 |
| 24.0 | 6.85 | 1 | 2.55 |
| 21.7 | 6.84 | 1 | 2.55 |
| 19.3 | 5.79 | 1 | 2.37 |
| 15.0 | 4.78 | 1 | 2.16 |
| 10.9 | 3.79 | 1 | 1.90 |
| 6.8 | 4.29 | 1 | 2.04 |
| 3.7 | 3.08 | 1 | 1.67 |

[a] Multiple listing of temperature entries result from temperature cycles to show the reversibility of the intensity change. Temperatures were determined with the internal NMR temperature sensor. [b] Manual integration. [c] Determined by equation (5).

**Table S2**. Thermodynamic data derived from the van't Hoff plot from the equilibrium between $^t$Bu-1$_2$ and $^t$Bu-1•.

| | | |
|---|---|---|
| $\Delta H_d^{298}$ | 7.9(3) | kcal mol$^{-1}$ |
| $\Delta S_d^{298}$ | 32.0(9) | cal K$^{-1}$ mol$^{-1}$ |
| $\Delta G_d^{298}$ | –1.6(5) | kcal mol$^{-1}$ |

### Experimental procedures

**General information:** All chemicals were purchased from Aldrich, Alfa Aesar, Acros Organics or Tokyo Chemical Industry in reagent grade or better quality and used without further purification if not mentioned different. For reactions under inert atmosphere nitrogen was used without purification. If not mentioned otherwise, dry solvents were obtained from drying system MBraun MB-SRS 800 and contained less than 15 ppm water. For reactions in the glovebox (MBraun Unilab Glovebox, $N_2$ filled; $O_2$ and $H_2O$ levels were kept below 0.1 ppm) benzene-*d*$_6$ and cyclohexane-*d*$_{12}$ were dried by distillation from Na/benzophenone in a nitrogen atmosphere onto sodium and were then degassed with 15 to 20 freeze-pump-thaw cycles. The solvent then was brought into the glove box and the piece of sodium was scratched at the glass wall to permanently show a shiny surface. In case the surface got stained, scratching was repeated daily till it stayed shiny and were further stored and used so. For glovebox reaction grease was exchanged by Glindemann PTFE Sealing Rings. All solvents and hydroscopic chemicals used in the glove box were dried previously and all used materials and chemicals were stored at least two days before usage inside the glove box to lose traces of water and oxygen. Analytical thin-layer chromatography (TLC) was performed on plastic-backed silica gel 60 coated with a fluorescence indicator. Visualization of TLC plate was performed by UV (254 nm), iodine or phosphomolybdic acid stain. Column chromatography was performed using Merck silica gel 60 (0.040–0.063 mm). $^1$H and $^{13}$C NMR spectra were measured with Bruker spectrometer Avance II 200 Hz (AV 200), Avance II 400 MHz (AV 400) and Avance III 600 MHz (AV 600), with and without TMS as the internal standard. Chemical shifts are reported in parts per million (ppm). Used GC-MS analyses were performed with a Quadrupol-MS HP MSD 5971(EI) and HP 5890A GC equipped with a J & W Scientific fused silica GC column (30 m × 0.250 mm, 0.25 micron DB–5MS stationary phase: 5% phenyl and 95% methyl silicone) using He (4.6 grade) as carrier gas. The EPR spectrum was recorded with a Varian combination of an E-101 Microwave Bridge, VHF-3400 Magnet and E-9 EPR Spectrometer.

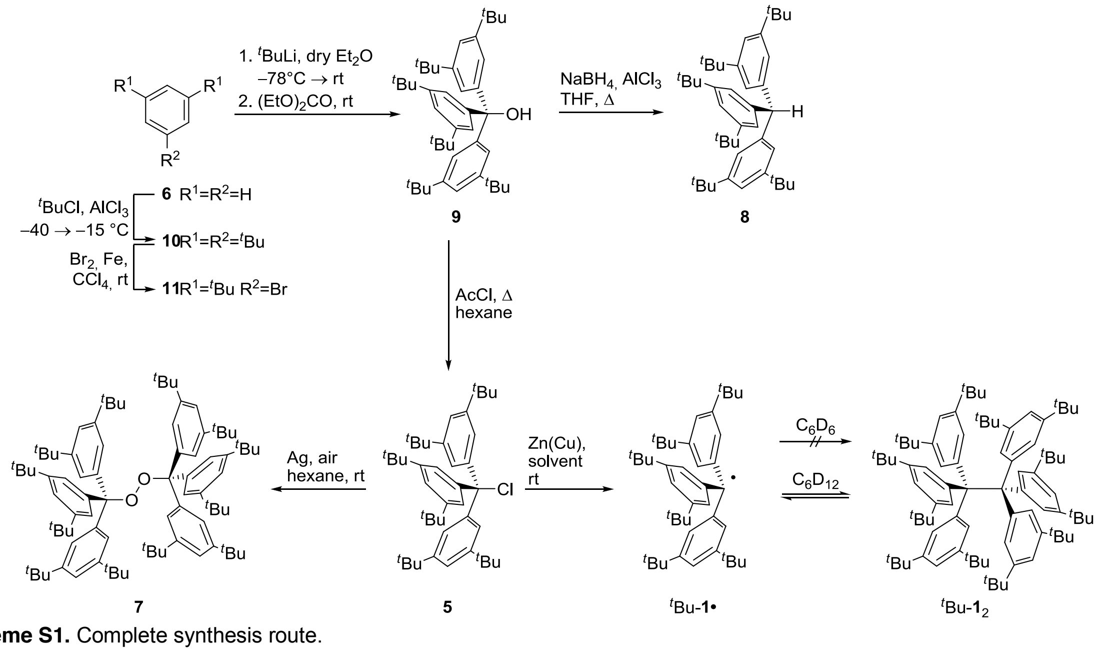


**Scheme S1.** Complete synthesis route.

### 1,3,5-Tri-*tert*-butylbenzene 10[1]

In a 500 mL one necked flask a mixture of 15.6 g (0.2 mol; 1 equiv.) benzene and 200 mL (1.8 mol; 9 equiv.) was cooled to –40 °C with a stirred acetone/liq. nitrogen bath. Then over 40 min 13.3 g (0.1 mol; 0.5 equiv.) yellow aluminium(III)chloride was added in portions. This yellow suspension was warmed to –15 °C, stirred for further 2 h at this temperature and put on 200 mL ice. Thereafter, 50 mL of dichloromethane were added, the organic phase separated, washed with 250 mL brine, dried over $Na_2SO_4$ and concentrated *in vacuo* to give 50.3 g of a pale yellow, solid crude product (the product was a liquid at 55 °C/15 mbar and congealed while cooling to rt). The product was recrystallized from 135 mL methanol at max. 60 °C (otherwise the product melts to give a clear emulsion that do not result in white crystals but in a congealed pale yellow block) to give 34.9 g (71%) of 1,3,5-tri-*tert*-butylbenzene as white needles.

m.p. 71–72 °C. IR (KBr): 714 (m), 873 (m), 900 (m), 1249 (s), 1362 (s), 1598 (s), 1761 (w), 1780 (w), 2865 (s), 2901 (s), 2964 (vs), 3076 (w) $cm^{-1}$. $^1H$ NMR (400 MHz, $CDCl_3$): δ = 7.27 (s, 3H), 1.35 (s, 27H) ppm. $^{13}C$ NMR (100 MHz, $CDCl_3$): δ = 149.9, 119.5, 35.0, 31.6 ppm. MS (EI): m/z = 246 (10%, $M^+$), 231 (100%, $[M^+] – CH_3$), 57 (32%, $C_4H_9^+$).

**1-Bromo-3,5-di-*tert*-butlybenzene 11**[1]

In a 100 mL one necked flask where dissolved 10.0 g (41 mmol; 1 equiv.) 1,3,5-tri-*tert*-butlybenzene in 25 mL $CCl_4$ and then added 2.8 g (50 mmol; 1.3 equiv.) iron powder. Thereafter a solution of 4.4 mL (85 mmol; 2.1 equiv.) $Br_2$ in 8 mL $CCl_4$ was added in 30 min at rt and the brown solution stirred for 2 h, poured on 50 mL ice, decanted in a separation funnel and separated between 100 mL aqueous $Na_2S_2O_3$ solution and 50 mL $CHCl_3$. The aqueous layer than was again extracted with 50 mL $CHCl_3$, the combined organic layers dried over $Na_2SO_4$ and concentrated *in vacuo* to give 21.4 g of a brown oil. After distillation over a 15 cm Vigreux column 9.4 g (86%) of 1-bromo-3,5-di-*tert*-butlybenzene were collected as a yellow oil with b.p. 115 °C/30 mbar.
IR (ATR): 549 (w), 703 (m), 751 (m), 841 (m), 863 (m), 1247 (m), 1280 (m), 1364 (m), 1407 (m), 1476 (m), 1564 (s), 1598 (s), 2716 (vw), 2746 (vw), 2869 (s), 2905 (s), 2964 (vs), 3080 (w) $cm^{-1}$. $^1H$ NMR (400 MHz, $CDCl_3$): δ = 7.32 (s, 3H), 1.30 (s, 18H) ppm. $^{13}C$ NMR (100 MHz, $CDCl_3$): δ = 153.2, 125.9, 122.3, 121.2, 35.2, 31.4 ppm. MS (EI): m/z = 270.0 (16%, $M^+$), 268.0 (16%, $M^+$), 255.0 (94%, $[M^+] – CH_3$), 253.0 (100%, $[M^+] – CH_3$).

**Tri(3,5-di-*tert*-butylphenyl)methanol 9**[2]

(*0.25 g to 10 g scale*) In a dried, argon flushed 250 mL Schlenk flask with septum were placed 37 mmol (3 equiv.) 1-bromo-3,5-di-*tert*-butylbenzene and dissolved with 50 mL dry diethylether. After cooling to –78 °C (acetone/liq. nitrogen) 44.6 mL (76 mmol; 6.1 equiv.) of a 1.7 M *tert*-butyllithium solution in pentane were added dropwise over 30 min, the white suspension was further stirred for 10 min and then allowed to warm to rt during 35 min. To the yellow reaction mixture was added a solution of 1.47 g (12 mmol; 1 equiv.) diethylcarbonate in 3 mL dry diethylether dropwise within 10 min and further stirred for 2 h at rt to result in a cloudy brown suspension. After addition of 100 mL water and 30 mL 2 M aqueous HCl, the layers were separated and the aqueous layer extracted with diethylether (3x30 mL). The combined organic layers were dried over $Na_2SO_4$ and concentrated carefully *in vacuo* to give 7.72 g of a yellow, glassy solid. Purification by column chromatography (silica gel, hexane/diethylether 20:1→10:1, $r_f$(10:1) = 0.28) gave pure product as a white solid in 78–84% yield.
m.p. 162 °C. IR (KBr): 727 (m), 880 (m), 1248 (m), 1362 (m), 1477 (m), 1595 (m), 2866 (m), 2903 (m), 2963 (s), 3438 (m, broad), 3581 (w) $cm^{-1}$. $^1H$ NMR (400 MHz, $CDCl_3$): δ = 7.28 (t, $^4J$ = 1.7 Hz, 3H), 7.04 (d, $^4J$ = 1.7 Hz, 6H), 2.76 (s, 1H), 1.23 (s, 54H) ppm. $^{13}C$ NMR (100 MHz, $CDCl_3$): δ = 149.7, 146.8, 122.8, 120.5, 83.6, 35.0, 31.6 ppm. $^1H$ NMR (400 MHz, $C_6D_6$): δ = 7.53 (d, $^4J$ = 1.8 Hz, 6H), 7.48 (t, $^4J$ = 1.8 Hz, 3H), 2.63 (s, 1H), 1.26 (s, 54H) ppm. $^{13}C$ NMR (100 MHz, $C_6D_6$): δ = 150.2, 147.7, 123.4, 120.7, 83.7, 35.1, 31.7 ppm. $^1H$ NMR (200 MHz, $C_6D_{12}$): δ = 7.25 (t, $^4J$ = 1.7 Hz, 3H), 7.06 (d, $^4J$ = 1.7 Hz, 6H), 2.34 (s, 1H), 1.21 (s, 54H) ppm. $^{13}C$ NMR (50 MHz, $C_6D_{12}$): δ = 145.0, 148.2, 123.5, 120.4, 83.7, 35.4, 31.9 ppm.

**Tris(3,5-di-*tert*-butylphenyl)methyl chloride 5**[3]

In a nitrogen flushed 25 mL two-necked Schlenk flask with condenser and septum were solved 504 mg (1 equiv./0.8 mmol) tris(3,5-di-*tert*-butylphenyl)methanol in 8 ml dry benzene (stored over 3 Å molecular sieves) and refluxed. Than 0.2 mL (3.5 equiv./2.9 mmol) acetyl chloride where in two steps added within 5 min and the yellow solution was refluxed for 30 min. After cooling to rt the solvent and reactants were removed *in vacuo* and the remaining yellow solid was recrystallized from dry hexane (freshly distilled from sodium/benzophenone) with 3 drops acetyl chloride (rt to –20 °C). Schlenk filtration gave 433 mg (83%) white needles which contained traces of the carbinol and were used without further purification.
$^1H$ NMR (600 MHz, $C_6D_6$): δ = 7.49 (s, 9H), 1.23 (s, 54H) ppm. $^{13}C$ NMR (150 MHz, $C_6D_6$): δ = 150.3, 146.2, 125.4, 121.3, 84.4, 35.1, 31.6 ppm. $^1H$ NMR (400 MHz, $C_6D_{12}$): δ = 7.28 (t, $^4J$ = 1.8 Hz, 3H), 6.99 (d, $^4J$ = 1.9 Hz, 6H), 1.20 (s, 54H) ppm. $^{13}C$ NMR (100 MHz, $C_6D_{12}$): δ = 149.9, 146.7, 125.5, 120.9, 82.8, 35.4, 31.8 ppm.

**Bis(tri(3,5-di-*tert*-butylphenyl)methyl)peroxide 7**[4]

In a 25 mL round bottom flask were placed 49 mg (1 equiv./0.08 mmol) tris(3,5-di-tert-butylphenyl)methyl chloride and 56 mg (0.52 mmol; 6.4 equiv.) silver powder, suspended in 3 mL dry hexane (stored over 3 Å MS) and stirred for 65 min in air. Then the colorless suspension was filtered through celite and the filtrate was concentrated *in*

*vacuo* to give crude white product. Recrystallization from chloroform gave 23.2 mg (49%) of bis(tri(3,5-dialkylphenyl)methyl)peroxides as colorless crystals.
m.p. 246–252 °C (decomp.). IR (KBr): 3076 (w), 2962 (s), 2865 (s), 1596 (m), 1477 (m), 1463 (m), 1431 (m), 1362 (m), 11248 (m), 1205 (m), 880 (m) $cm^{-1}$. $^1H$ NMR (400 MHz, $C_6D_6$): δ = 7.48 (t, $^3J$ = 1.7 Hz, 6H), 7.29 (broad), 1.21 (s, 108H) ppm. $^{13}C$ NMR (100 MHz, $C_6D_6$): δ = 149.4, 144.0, 125.8 (broad), 120.7, 95.1, 35.0, 31.7 ppm. $^1H$ NMR (400 MHz, $C_6D_{12}$): δ = 8.25-5.50 (d, very broad), 7.25 (t, $^3J$ = 1.7 Hz, 2H), 1.21 (s, 36H) ppm. $^{13}C$ NMR (100 MHz, $C_6D_{12}$): δ = 149.5, 144.4, 126.1 (broad), 120.7, 35.2, 31.9 ppm. MS (ESI): m/z = 1214 (100%; [$M^+$] + Na), 1215 (93%; [$M^+$] + Na), 1216 (39%; [$M^+$] + Na). HRMS (ESI): m/z (found) = 1213,9656u ([$M^+$] + Na) ≡ 1190,9758 ($M^+$); m/z (calc.) = 1190,9758 ($M^+$).

### Tri(3,5-di-*tert*-butylphenyl)methane 8[5]

In an argon flushed 10 mL two neck round bottom flask with condenser and septum were suspended 203 mg (1 equiv./0.29 mmol) tris(3,5-dialkylphenyl)methanol, 54.6 mg (1.45 mmol; 5 equiv.) $NaBH_4$ and 111.4 mg (0.81 mmol; 2.8 equiv.) yellow $AlCl_3$ suspended with 3 mL dry THF (distilled from Na/benzophenone) at rt. The suspension turned brown and got colorless again after 5 min. After decrease of the vigorous hydrogen development the mixture was heated to reflux for 2 h. The cooled mixture was then quenched by dropwise addition of water, then further 10 mL water were added and the resulting colorless clear two layers were extracted with hexane (3x30 mL). The combined organic layers were dried with $Na_2SO_4$ and the solvent removed *in vacuo* to give crude product as a colorless film. Further purification by column filtration over silica gel with hexane gave 173 mg (89%) of pure tris(3,5-dialkylphenyl)methane as colorless film that slowly solidified. Large crystals were grown from isothermal evaporation of pentane.
m.p. 184.5 °C. IR (KBr): 717 (m), 874 (m), 1246 (m), 1362 (m), 1430 (m), 1460 (m), 1478 (m), 1596 (m), 2865 (s), 2903 (s), 2963 (s), 3086 (w) $cm^{-1}$. $^1H$ NMR (400 MHz, $C_6D_6$): δ = 7.43 (t, $^4J$ = 1.7 Hz, 3H), 7.36 (d, $^4J$ = 1.7 Hz, 6H), 5.79 (s, 1H), 1.27 (s, 54H) ppm. $^{13}C$ NMR (100 MHz, $CDCl_3$): δ = 150.7 (q), 144.6 (q), 124.5, 120.0, 59.0, 35.0 (q), 31.6 ppm. $^1H$ NMR (400 MHz, $C_6D_{12}$): δ = 7.19 (t, $^4J$ = 1.7 Hz, 3H), 6.88 (d, $^4J$ = 1.7 Hz, 6H), 5.41 (s, 1H), 1.22 (s, 54H) ppm. $^{13}C$ NMR (100 MHz, $CDCl_3$): δ = 150.4, 145.0, 124.7, 119.7, 58.7, 35.3, 31.9 ppm. MS (EI): m/z = 580 (10%, $M^+$), 581 (4.8%, $M^+$), 582 (1.3%, $M^+$). HRMS E.A.: (found) C 88.83 H 11.22 (calc.) C 88.90 H 11.10.

### Tri(3,5-di-*tert*-butylphenyl)methyl radical $^t$Bu-1•[6]

Inside a glovebox in a tube-like 10 mL glass flask with a joint and a glass stirring bar where placed 16.7 mg (1 equiv./ 27 µmol) tris(3,5-dialkylphenyl)methyl chloride and 35.5 mg (20 equiv./543 µmol) Zn(Cu). These were suspended with 0.9 mL dry, degassed benzene-$d_6$, then stirred 2 h at rt and finally the stirring was stopped for the solids to settle down. After 0.65 mL of the supernatant, clear orange solution was transferred *via* syringe to a flame sealable NMR test tube, a Schlenk attachment with plug were attached, the valve closed and the set up brought out of the glove box. Now the solution was frozen in a cooling bath (–20 °C), vacuum applied and then immediately the NMR test tube flame sealed.
$^1H$ NMR (600 MHz, $C_6D_6$): δ = 1.83 (s, 108H, very broad) ppm.

### Hexa(3,5-di-*tert*-butylphenyl)ethane $^t$Bu-$1_2$[7]

Inside a glovebox in a tube like 10 mL glass flask with a joint and a glass stirring bar where placed 16.7 mg (1 equiv./27 µmol) tris(3,5-di-tert-butylphenyl)methylchlorid and 35.5 mg (20 equiv./540 µmol) Zn(Cu). These were suspended with 0.8 mL dry, degassed cyclohexane-$d_{12}$, then stirred 6 h at rt and finally the stirring was stopped for the solids to settle down. After 0.65 mL of the supernatant, clear orange solution were transferred *via* a 1 mL syringe to a flame-sealable NMR test tube, a Schlenk attachment with stopper was attached, the valve closed and the set up brought out of the glove box. Now the solution was frozen in a cooling bath (–20 °C), vacuum applied and then immediately the NMR test tube flame sealed. The NMR test tube was kept away from light and did not show signs of decomposition within 5 weeks.
$^1H$ NMR (600 MHz, $C_6D_{12}$): δ = 7.75 (s, 3H), 7.19 (s, 3H), 6.02 (s, 3H), 0.98 (s, 27H), 0.95 (s, 27H) ppm. $^{13}C$ NMR (150 MHz, $C_6D_6$): δ = 151.1 (q), 149.9 (q), 149.5 (q), 128.6 (t), 128.1 (t), 120.3 (t), 71.5 (q), 35.3 (q), 35.2 (q), 32.1 (p), 31.8 (p) ppm.

## Spectra

### 1,3,5-Tri-*tert*-butylbenzene 10:

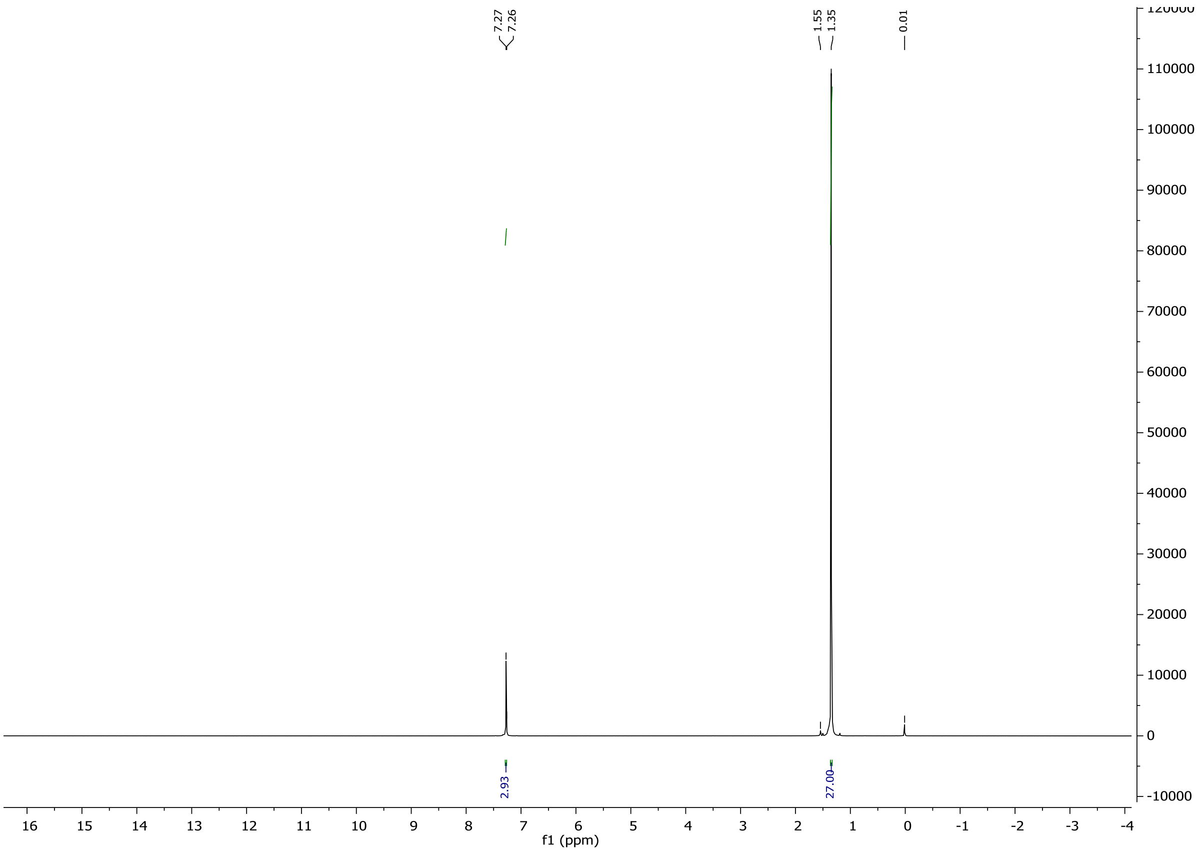


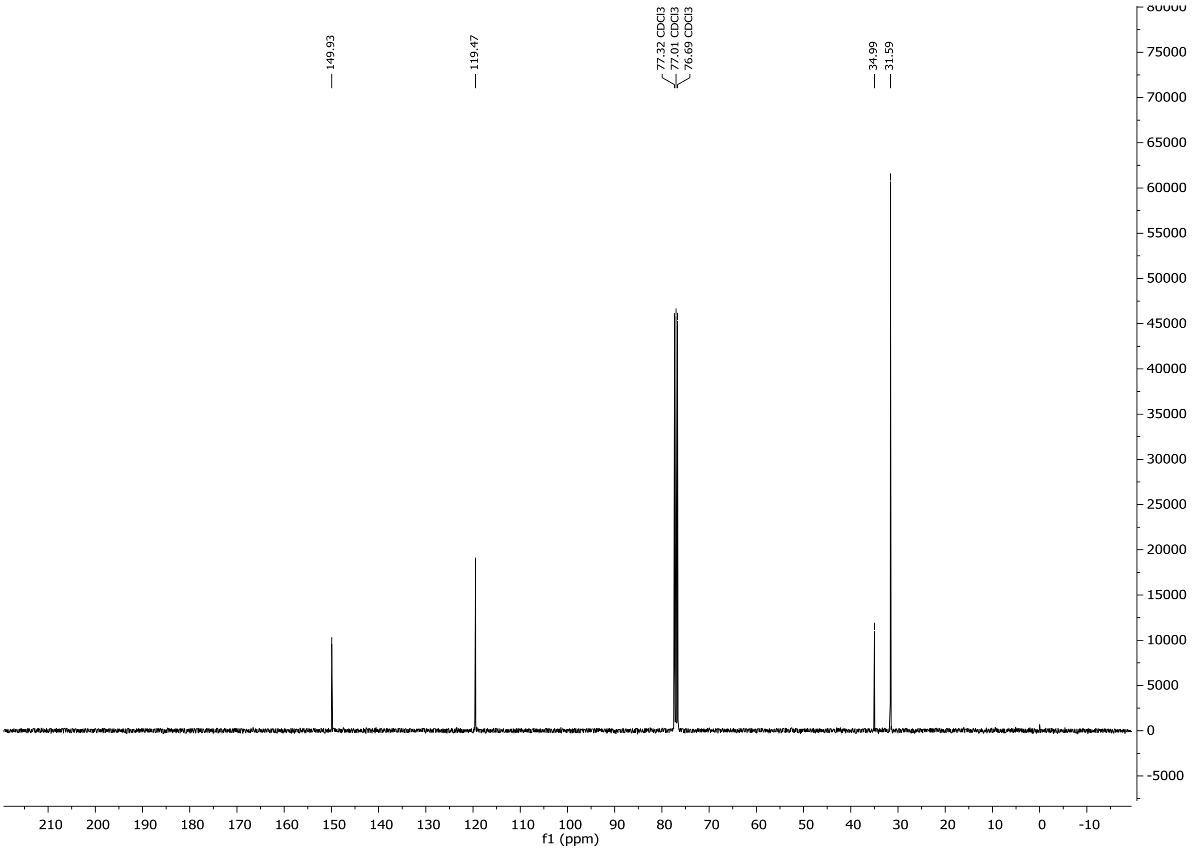

**1-Bromo-3,5-di-*tert*-butlybenzene 11:**

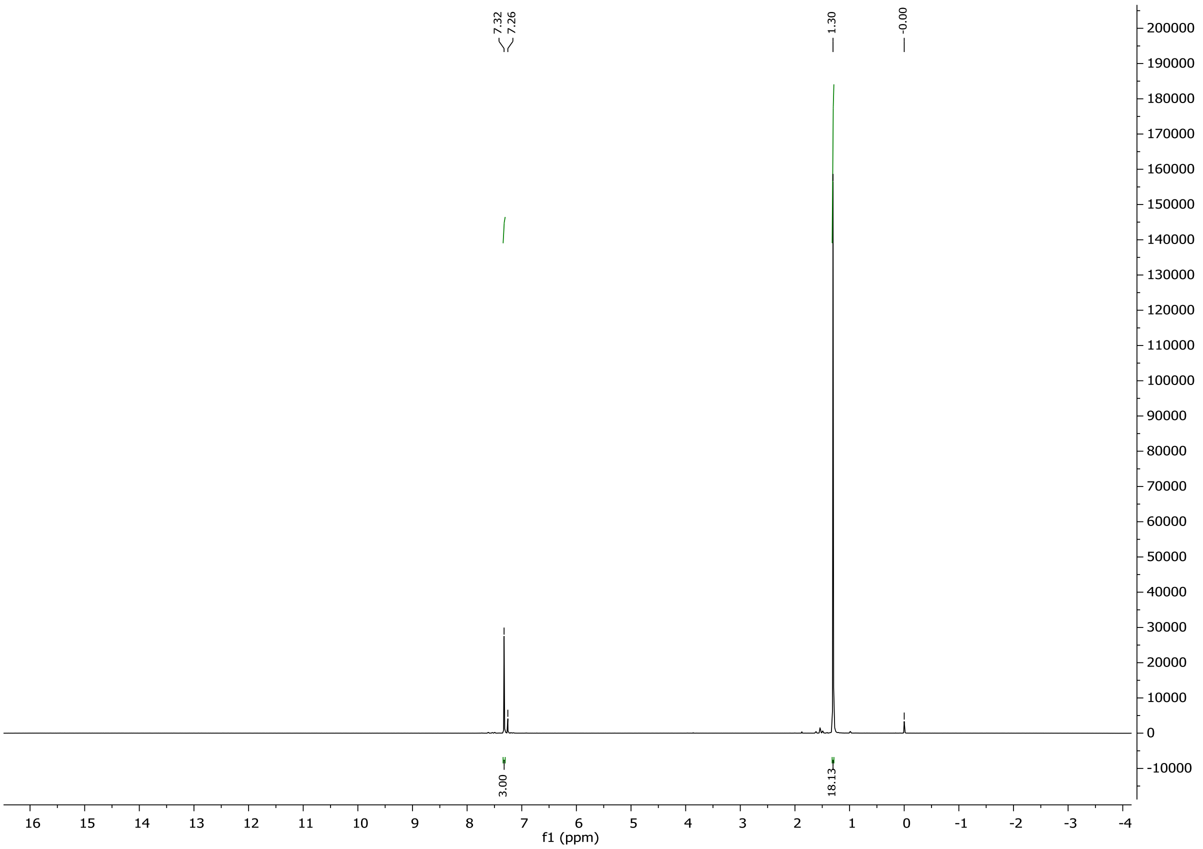


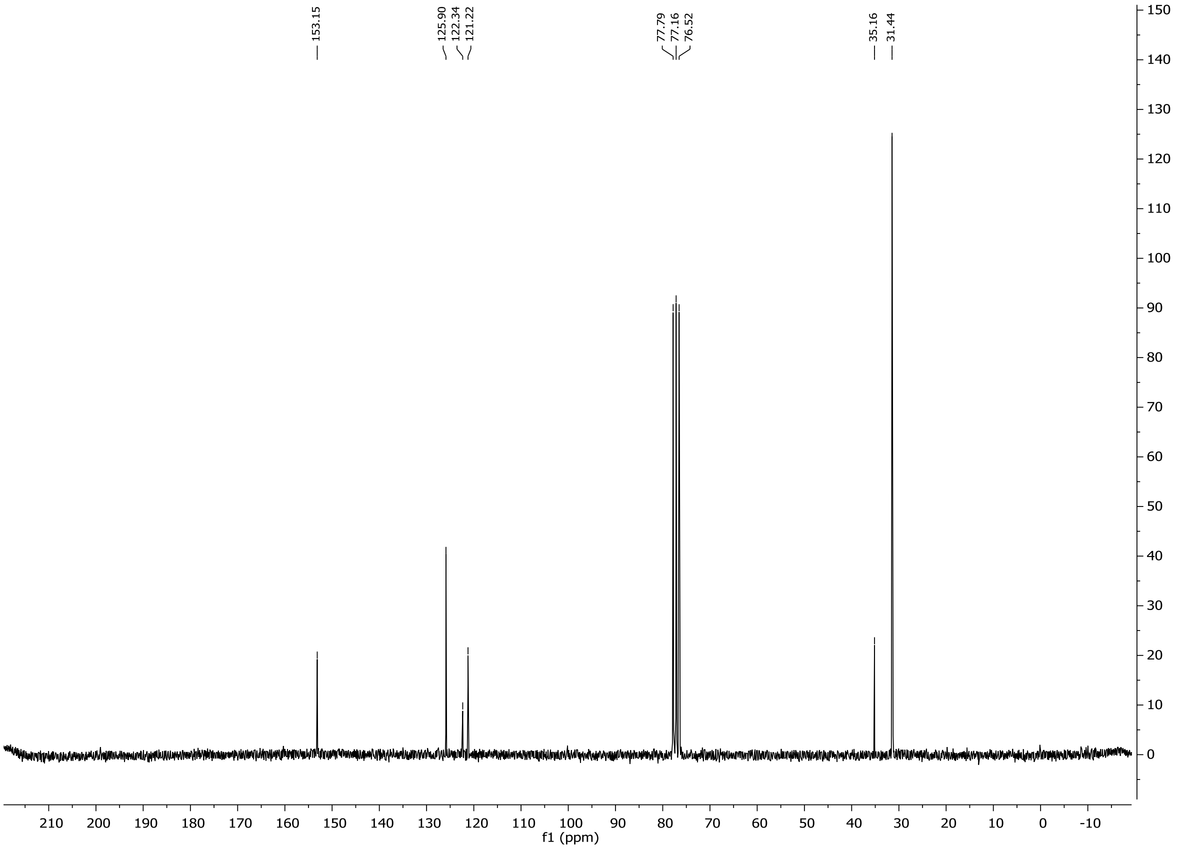

**Tri(3,5-di-*tert*-butylphenyl)methanol 9:**
In benzene-$d_6$

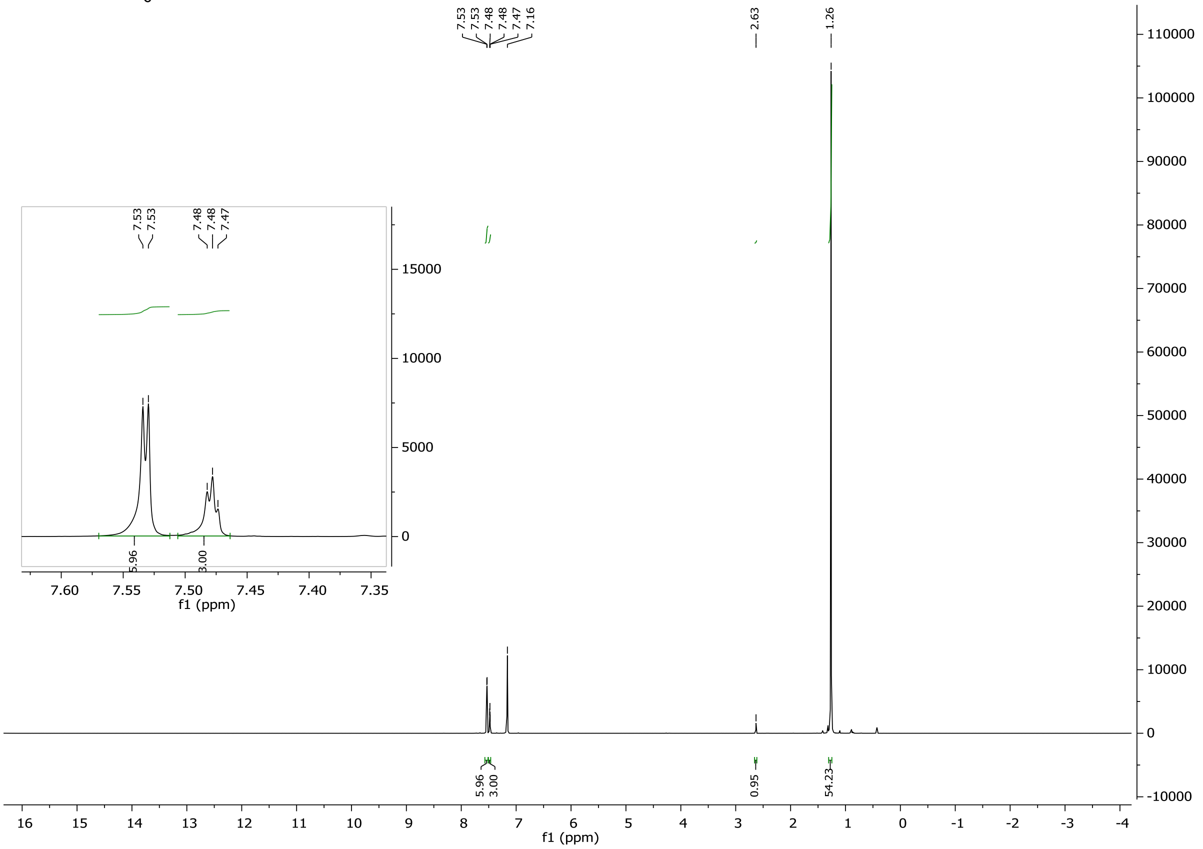


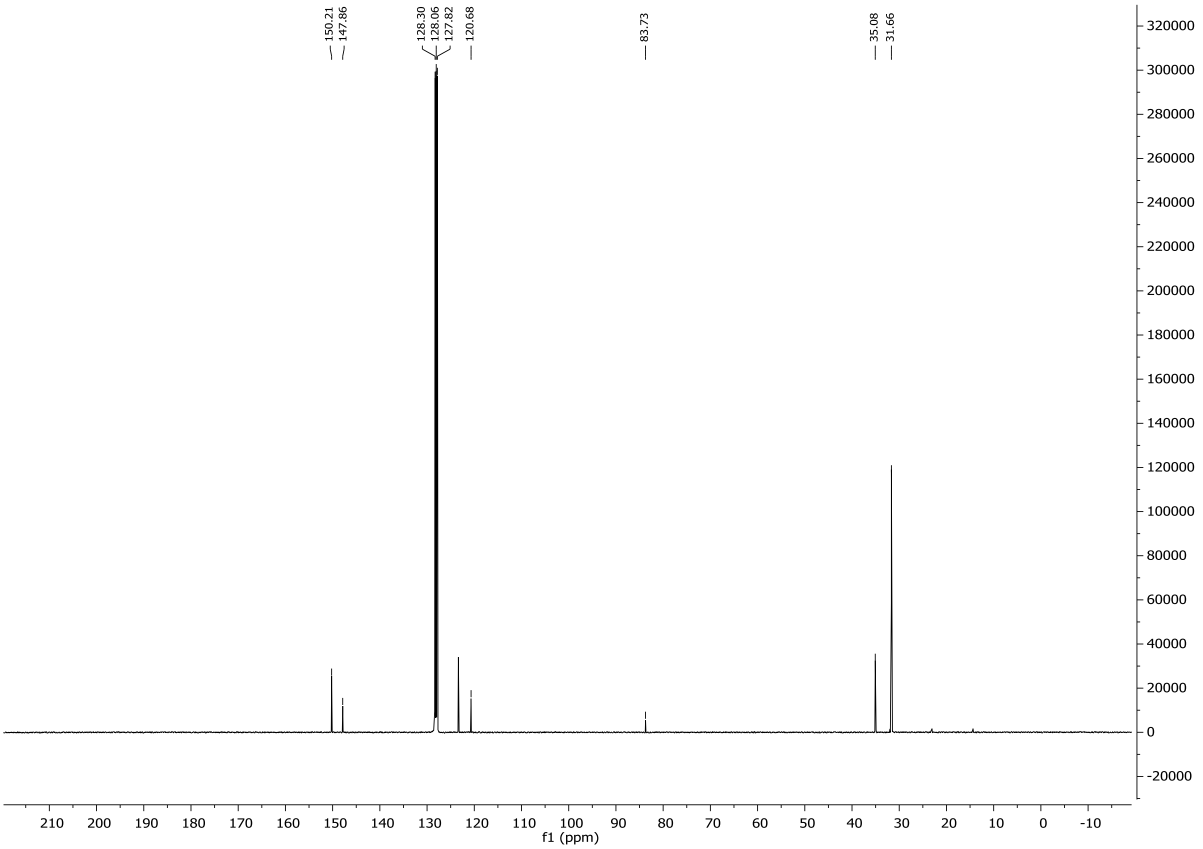

In cyclohexane-$d_{12}$

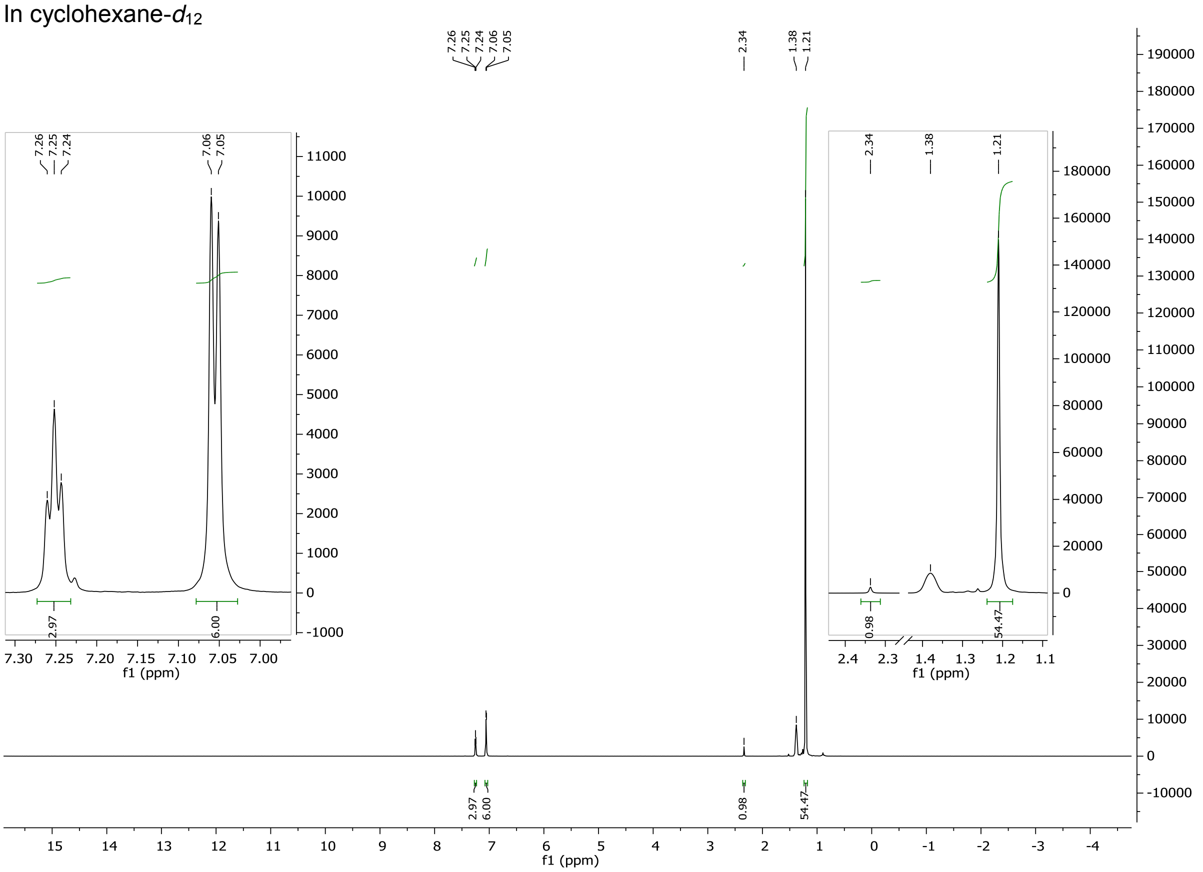


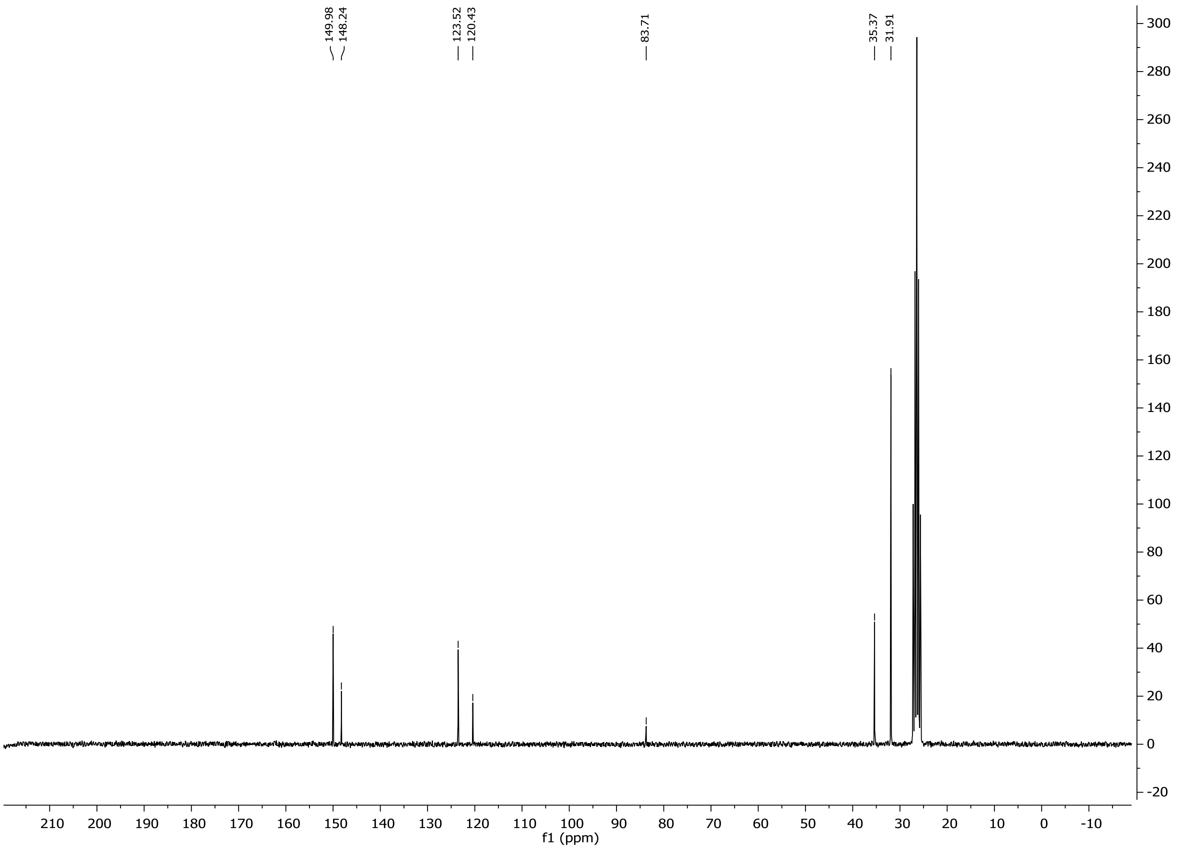

**Tri(3,5-di-*tert*-butylphenyl)methyl chloride 5:**
In benzene-$d_6$

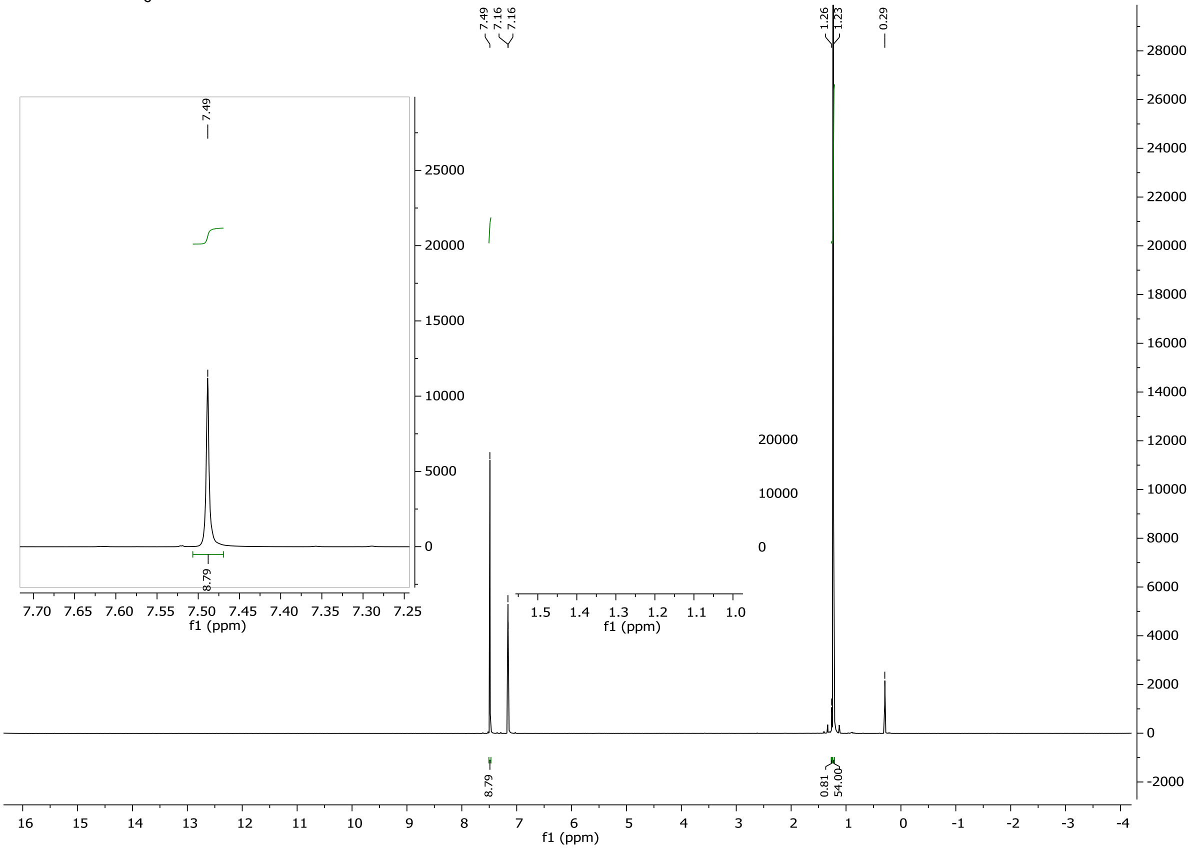

In cyclohexane-$d_{12}$

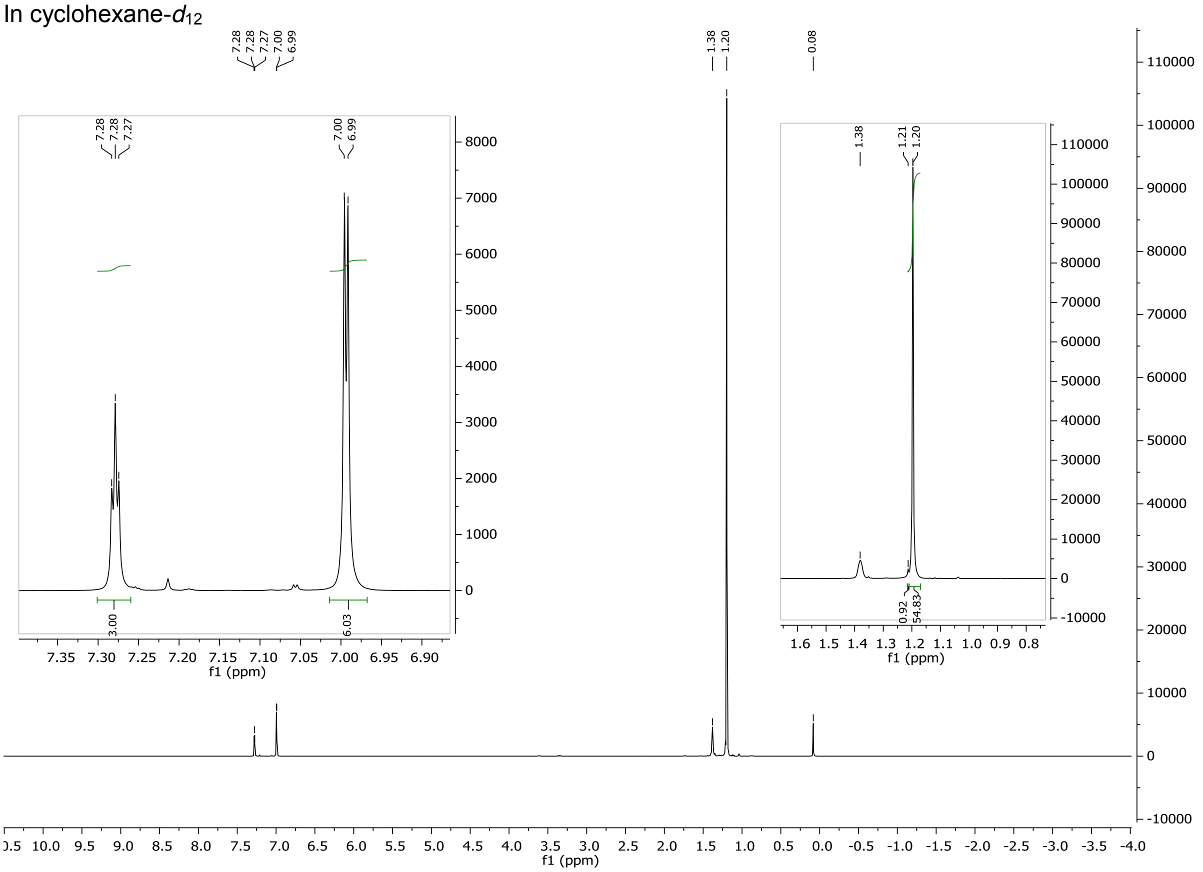

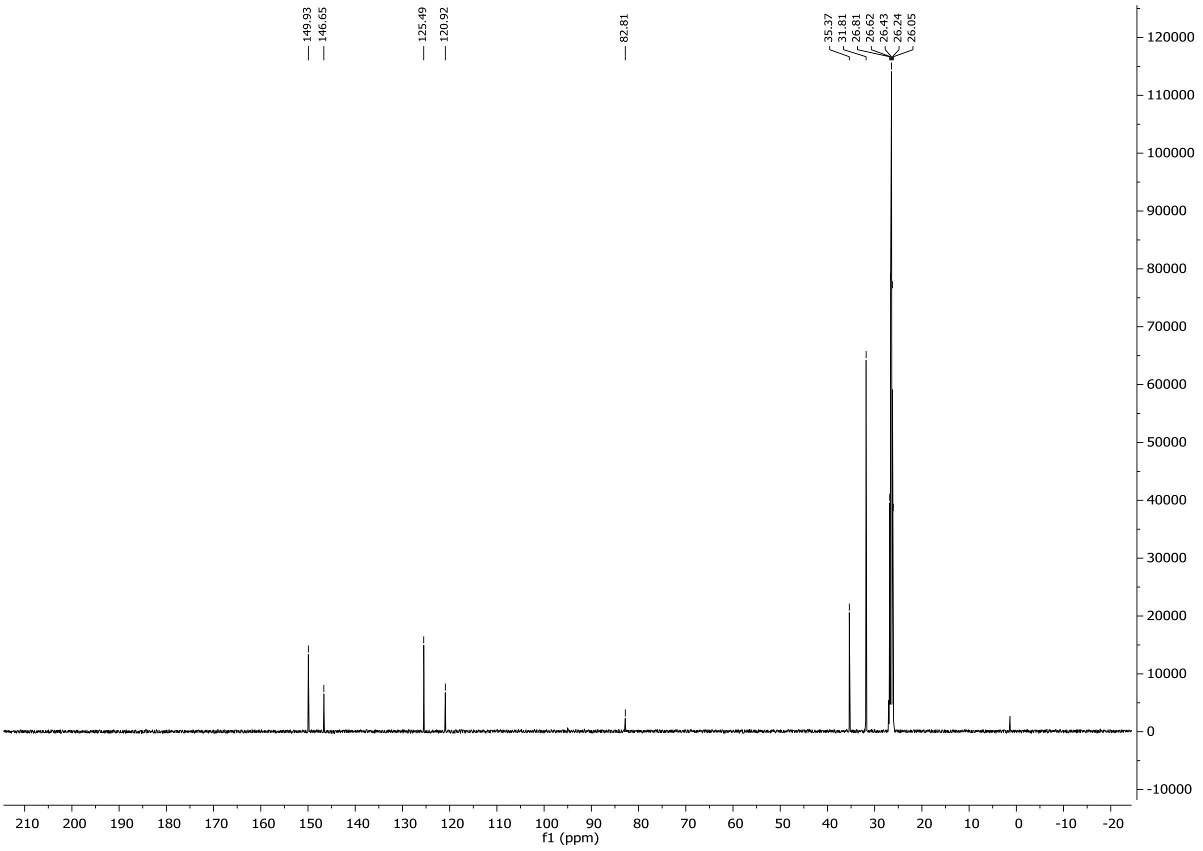

**Bis(tri(3,5-di-*tert*-butylphenyl)methyl)peroxide 7:**

In benzene-$d_6$

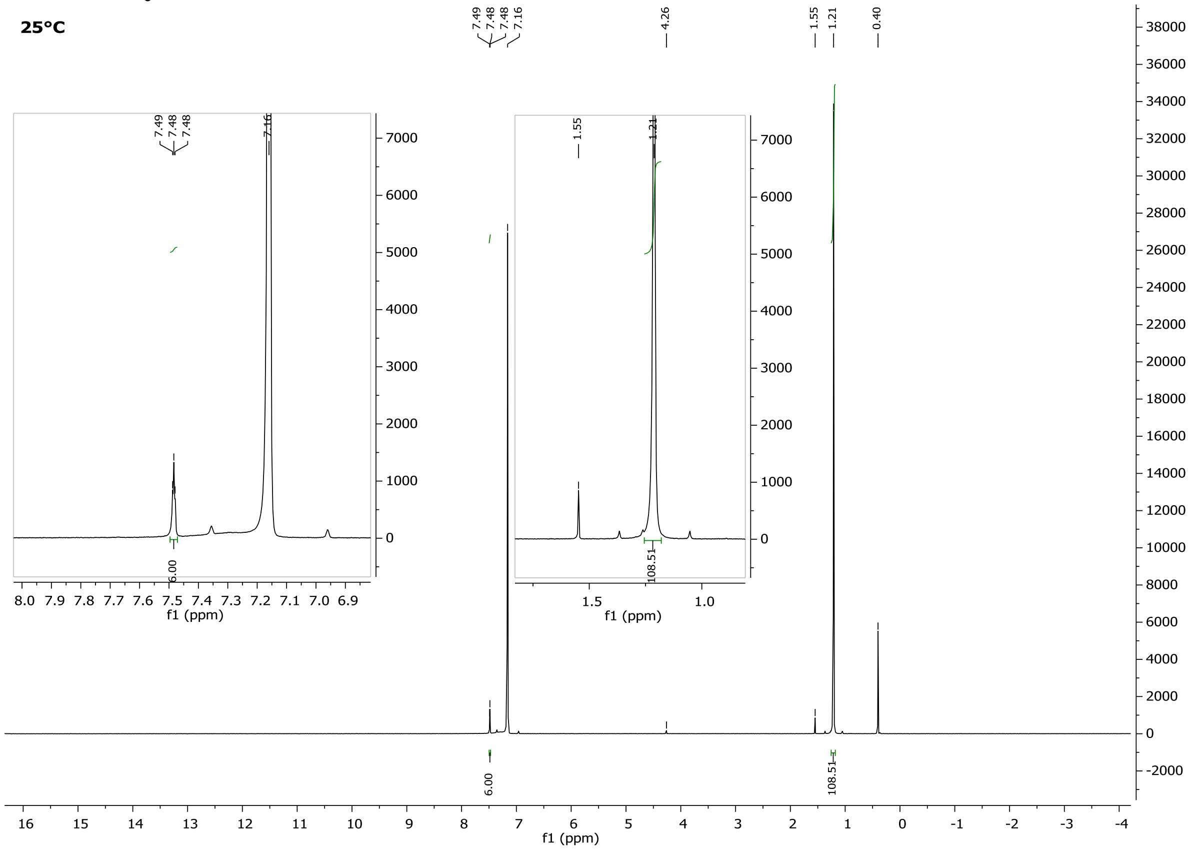


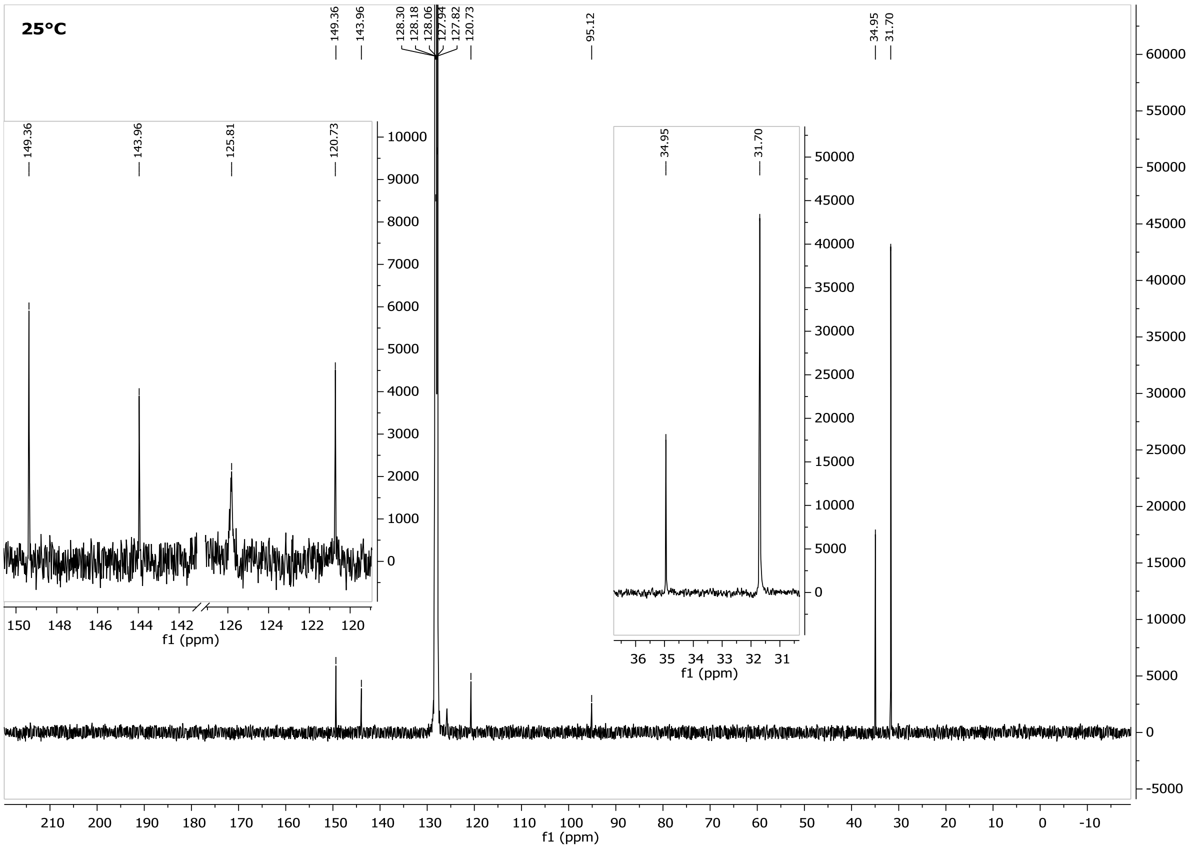

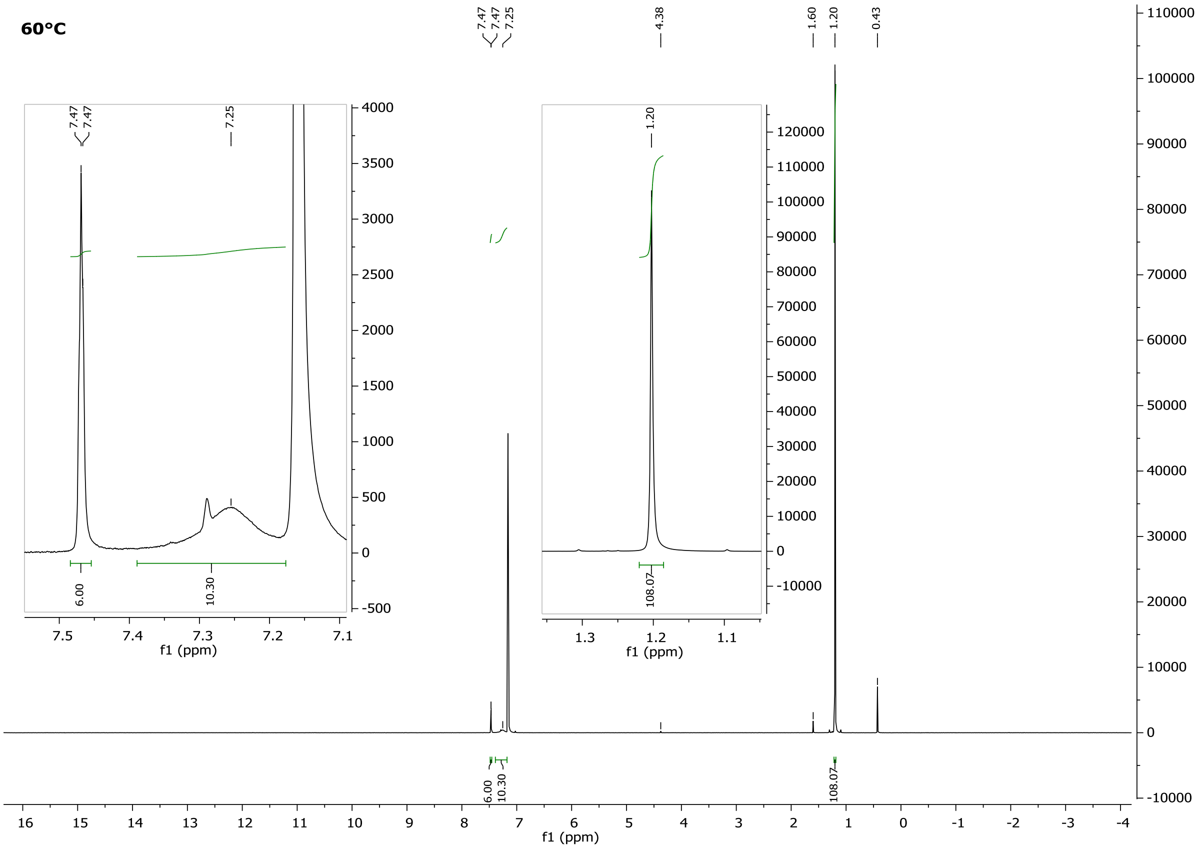
60°C
7.47
7.47
7.25
4.38
1.60
1.20
0.43
6.00
10.30
108.07
f1 (ppm)

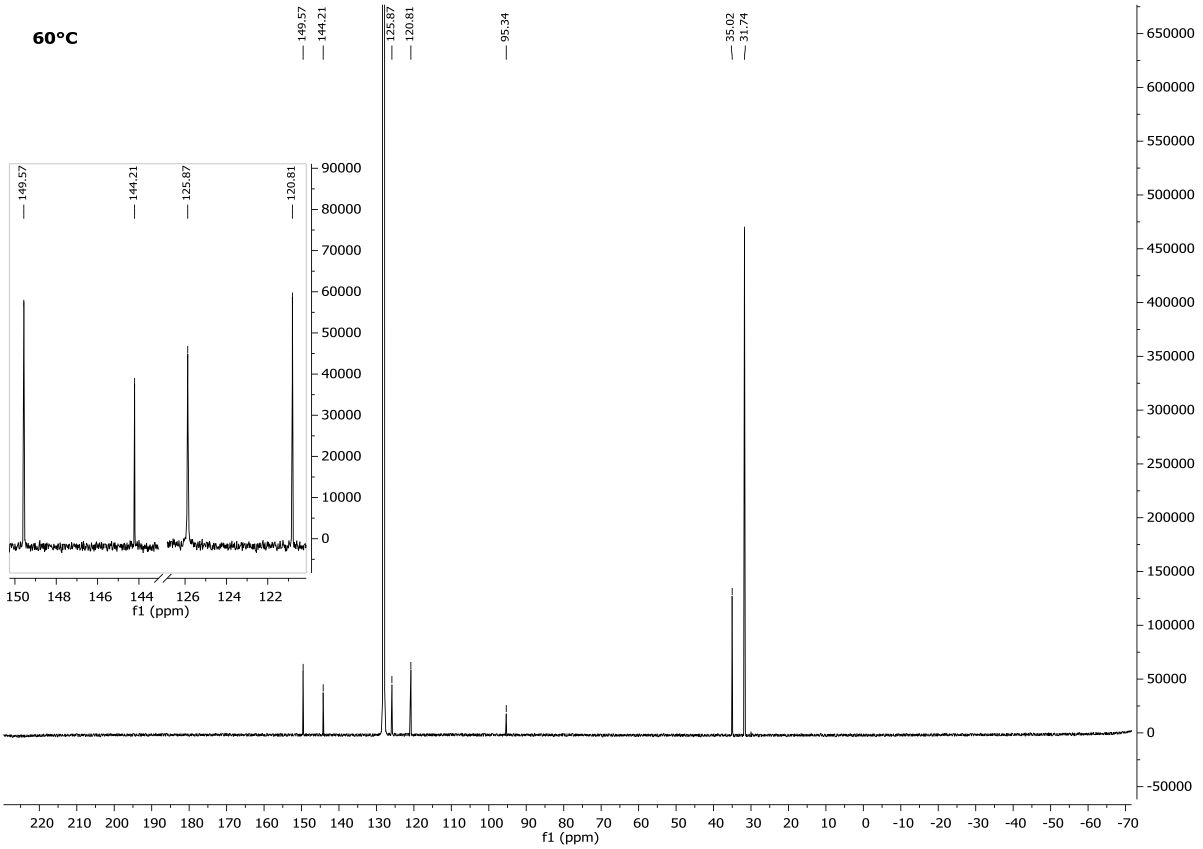
60°C
149.57
144.21
125.87
120.81
95.34
35.02
31.74
f1 (ppm)

In cyclohexane-$d_{12}$

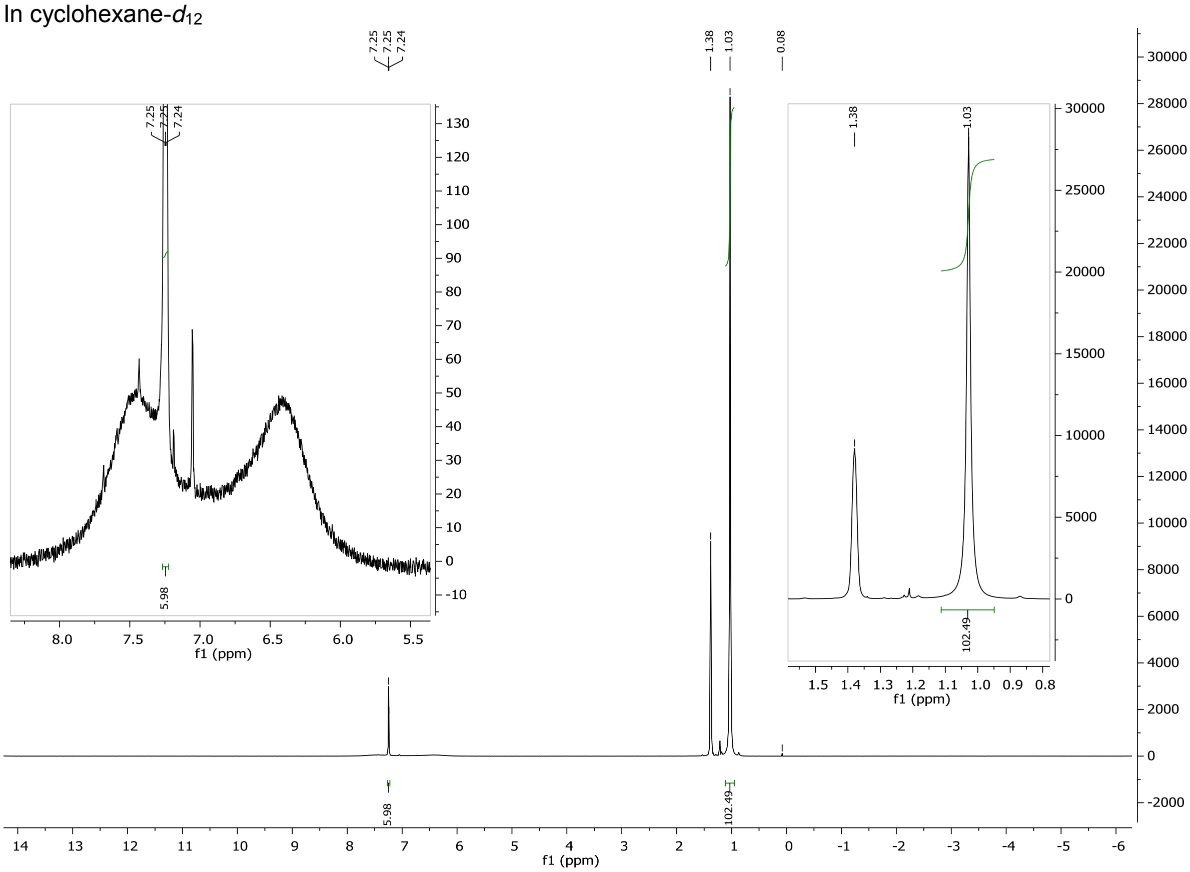


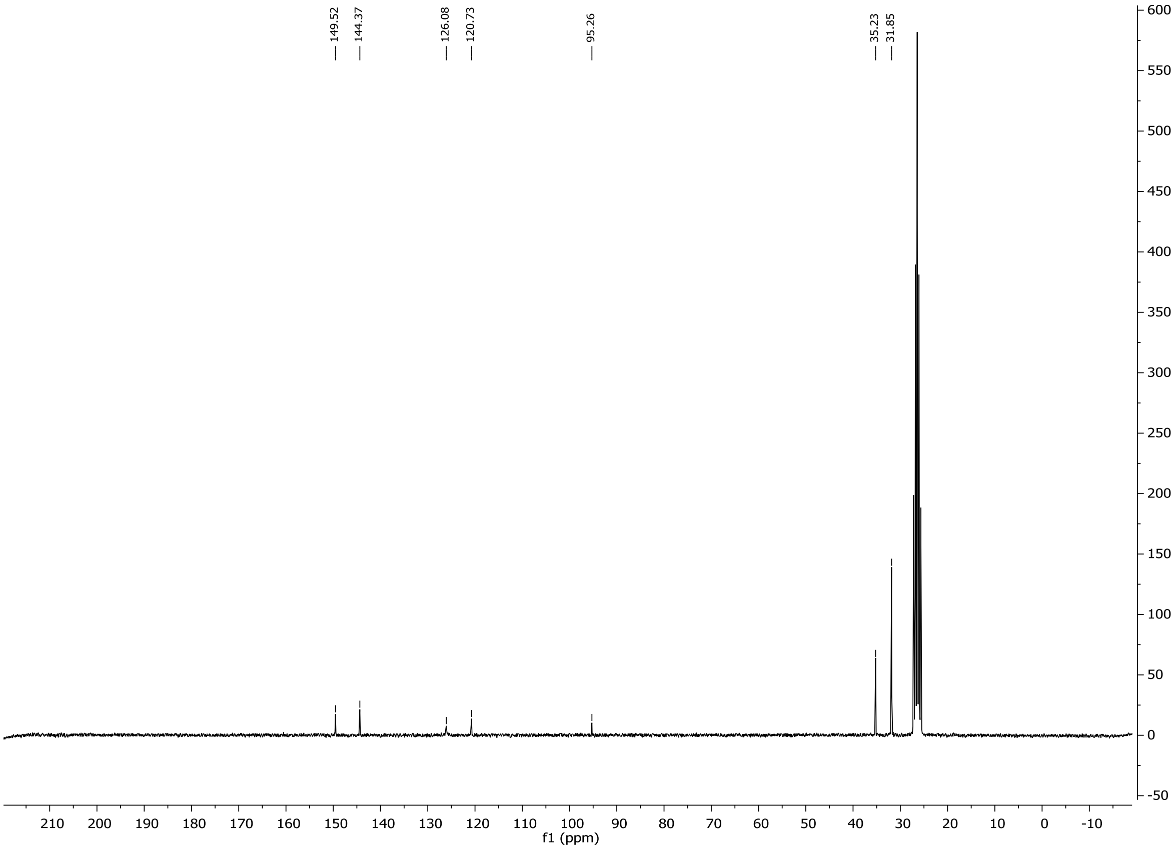

**Tri(3,5-di-*tert*-butylphenyl)methane 8:**

In benzene-$d_6$

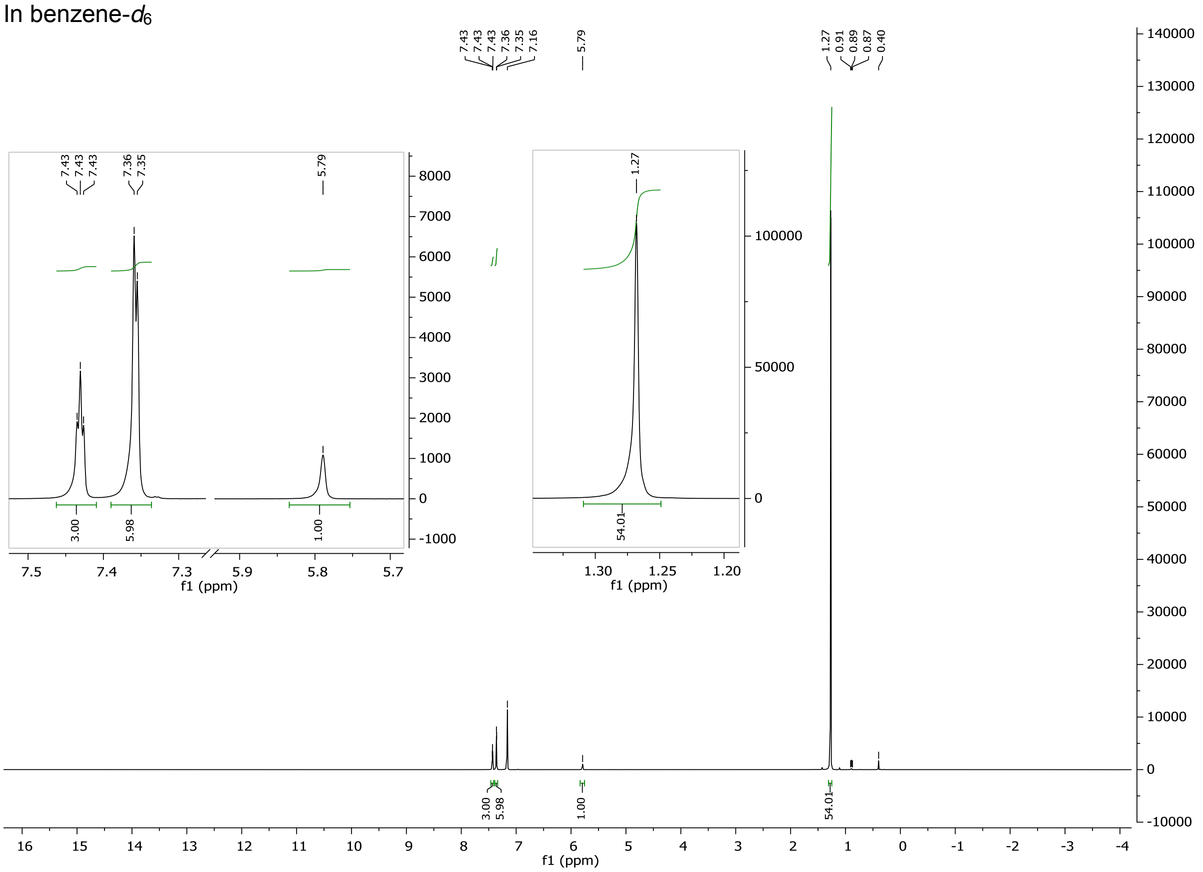


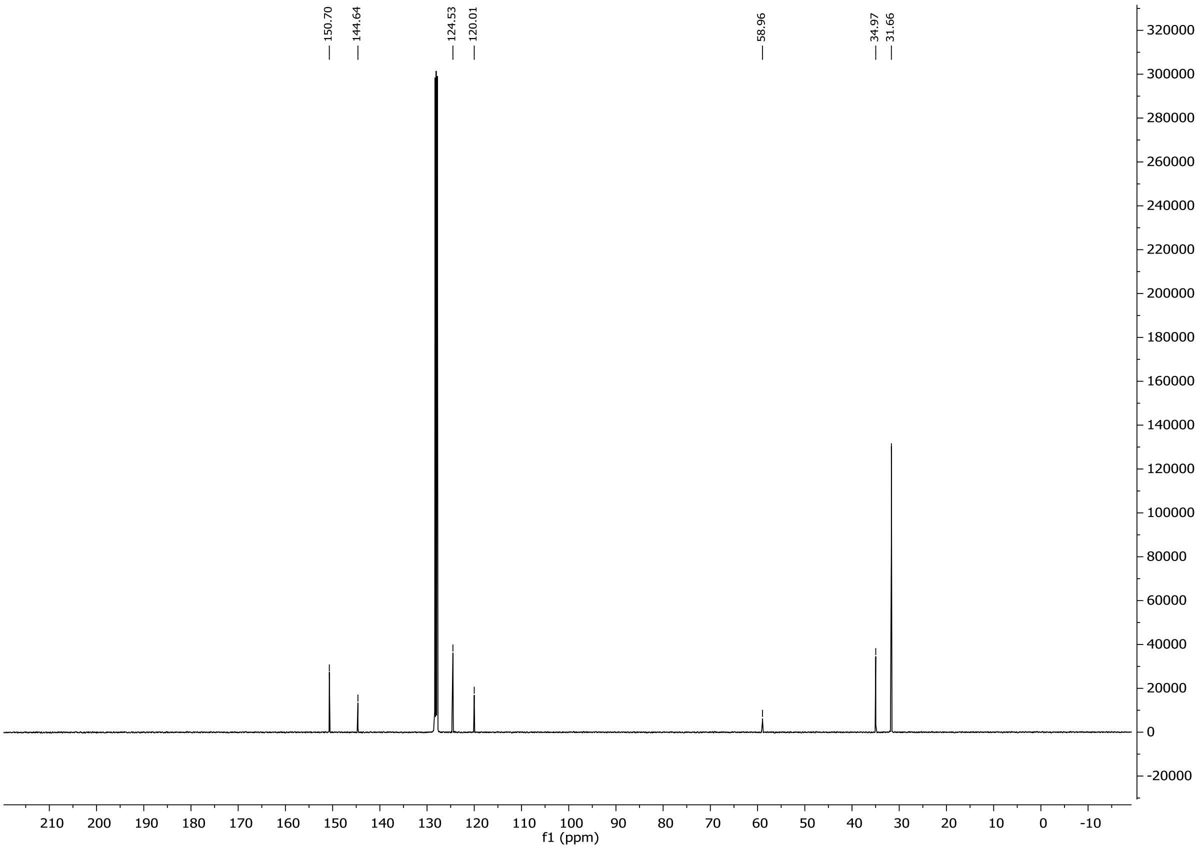

In cyclohexane-$d_{12}$

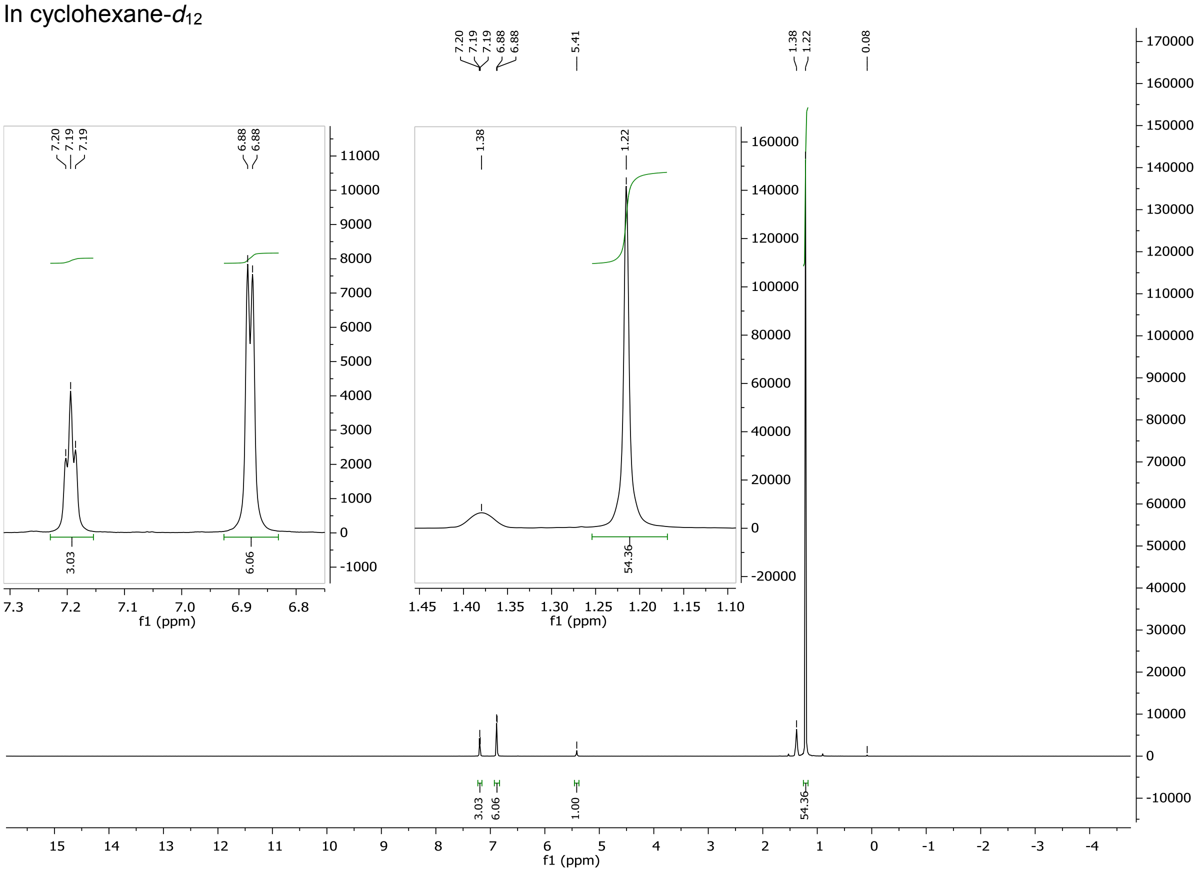

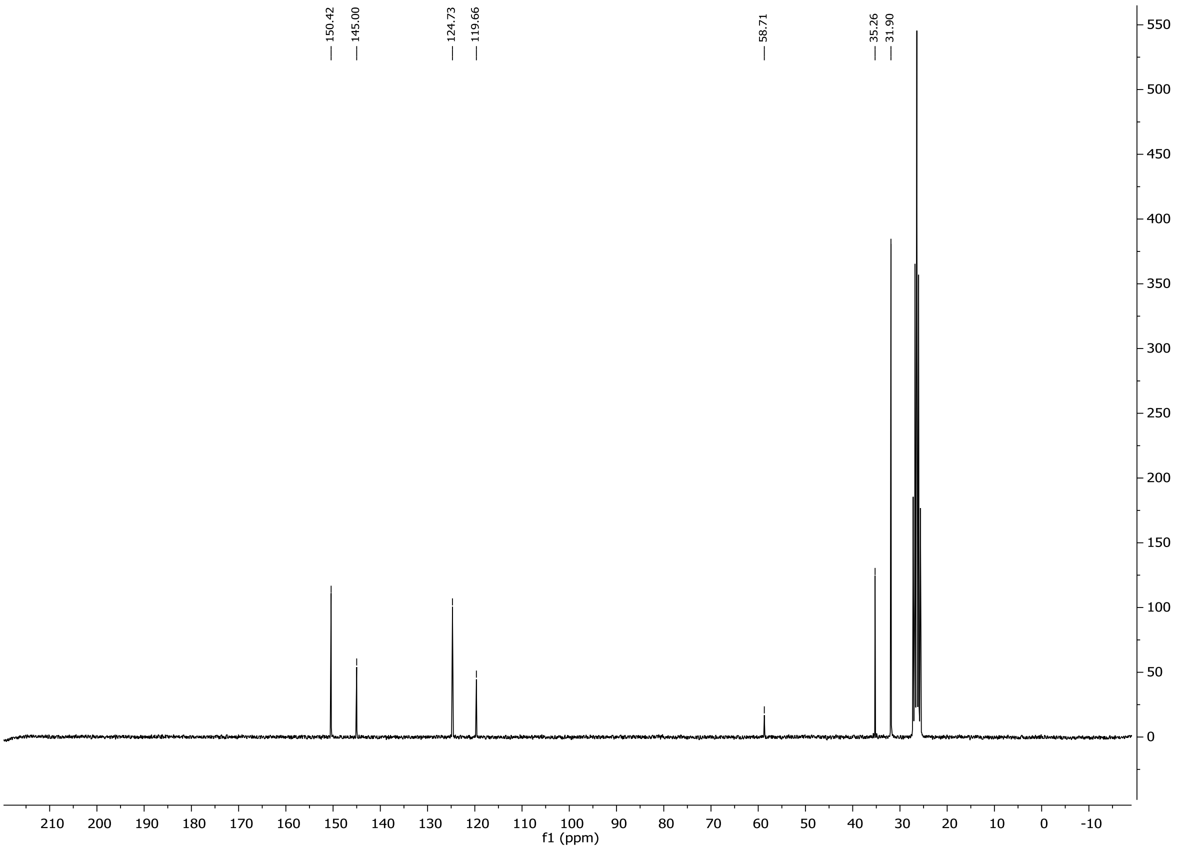

**Tri(3,5-di-*tert*-butylphenyl)methyl radical $^{t}$Bu-1•:**

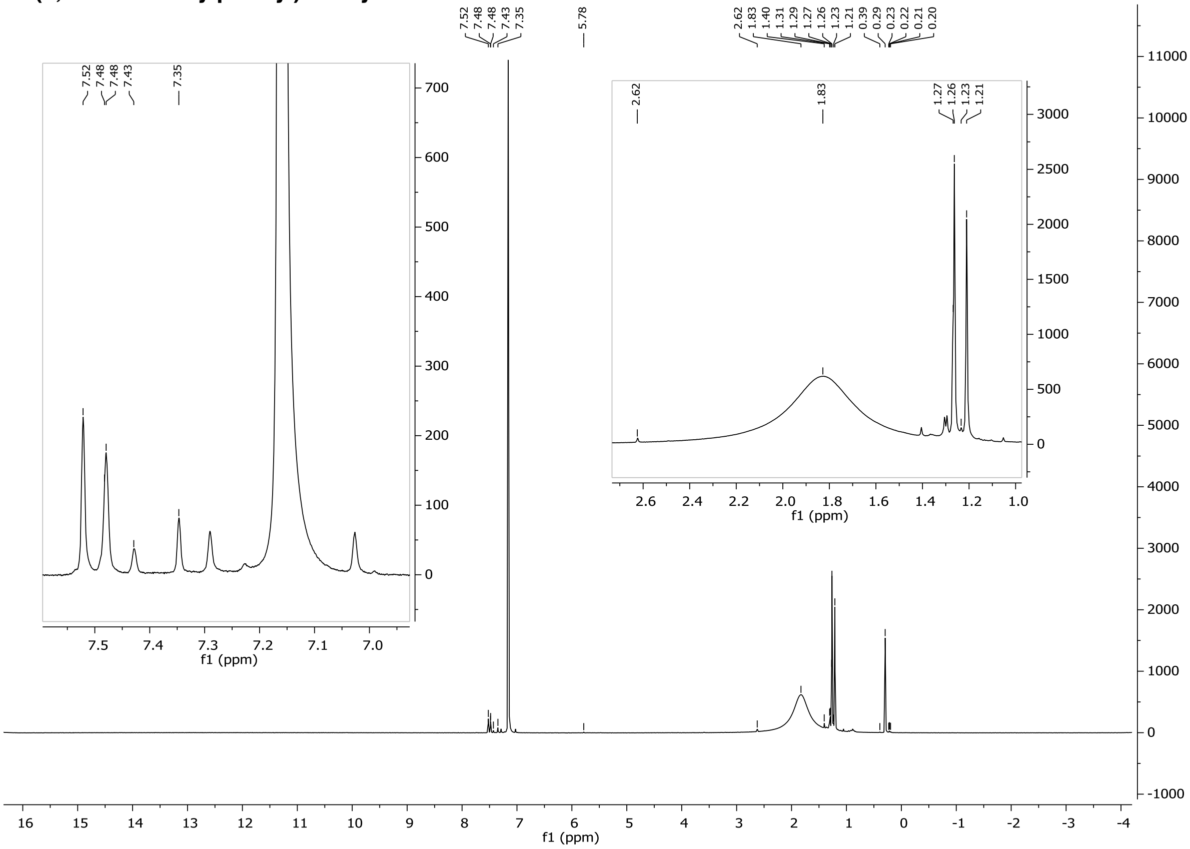


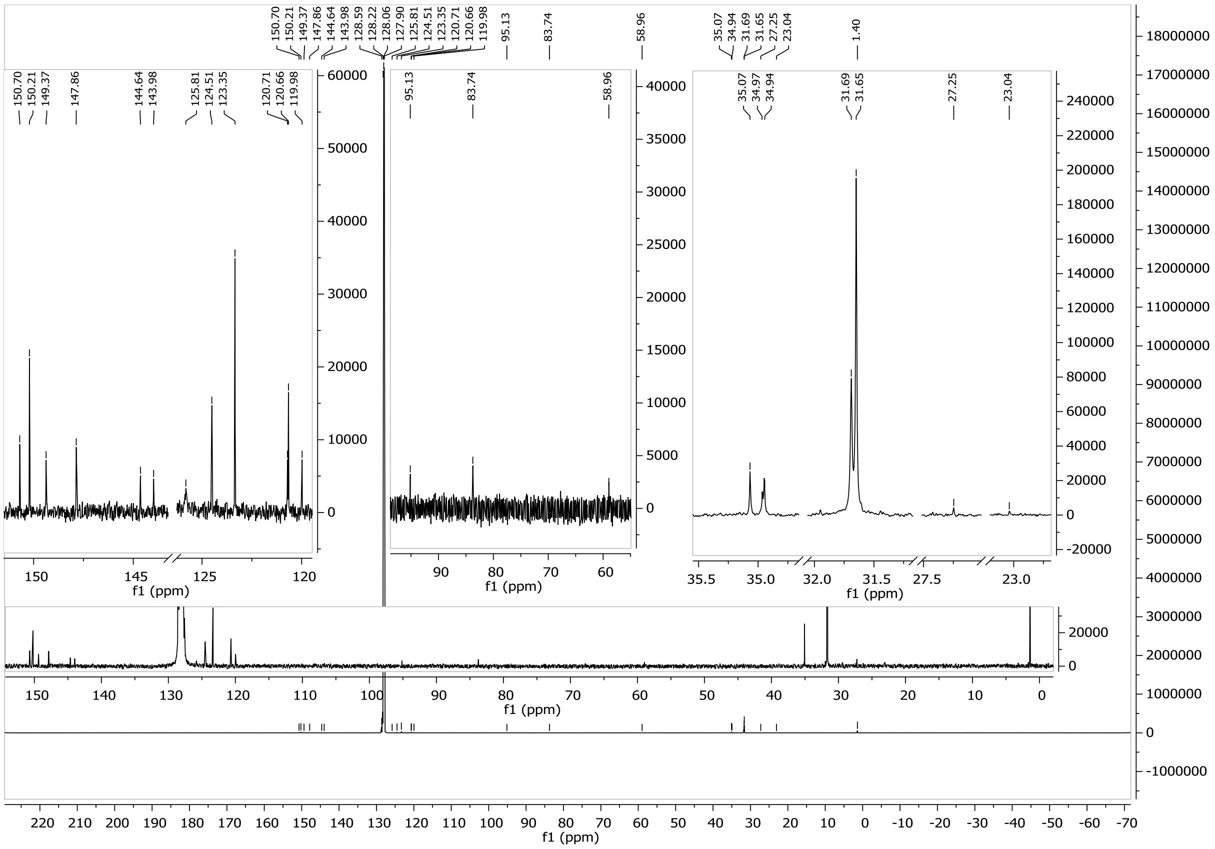

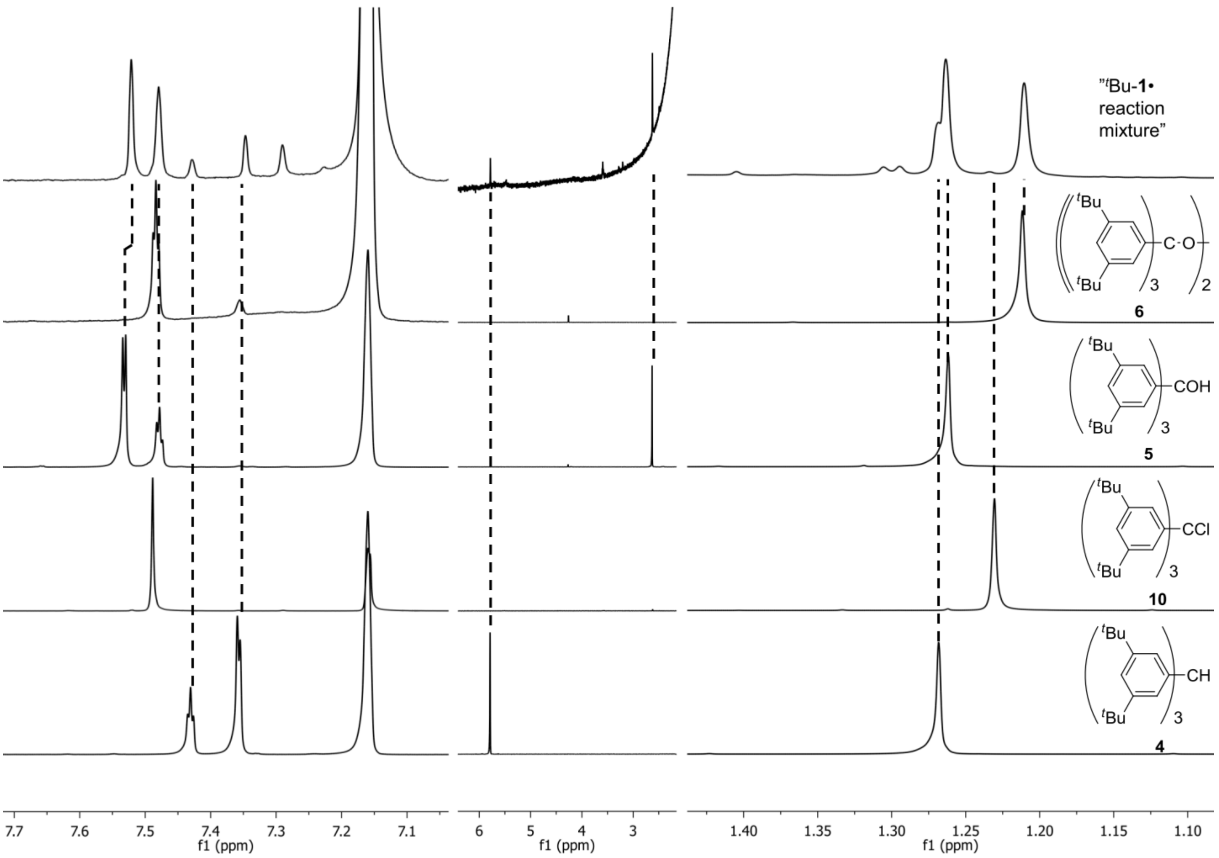
"$^t$Bu-**1**• reaction mixture"
$^t$Bu
C-O
3
2
6
COH
5
CCl
10
CH
4
7.7 7.6 7.5 7.4 7.3 7.2 7.1
f1 (ppm)
6 5 4 3
f1 (ppm)
1.40 1.35 1.30 1.25 1.20 1.15 1.10
f1 (ppm)

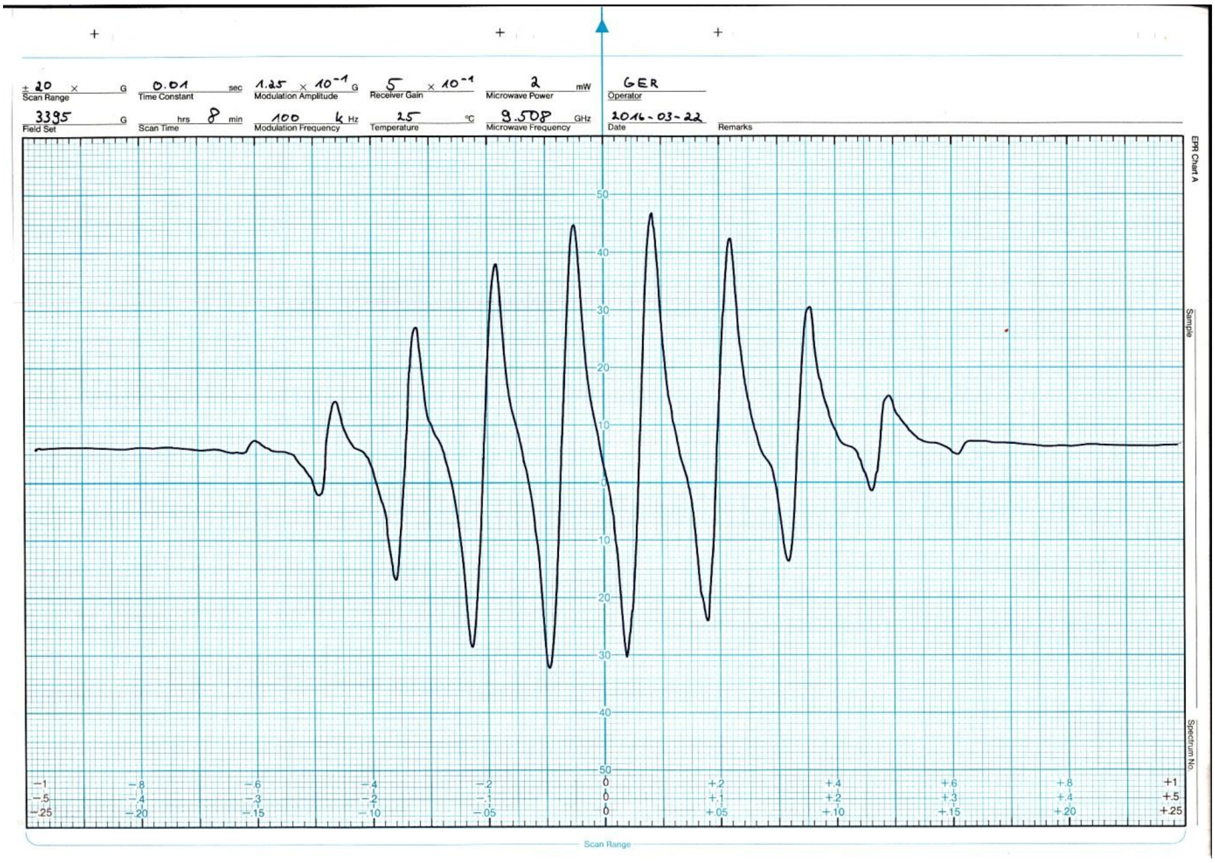
± 20 × G
Scan Range
0.01 sec
Time Constant
1.25 × 10⁻¹ G
Modulation Amplitude
5 × 10⁻¹
Receiver Gain
2 mW
Microwave Power
GER
Operator
3395 G
Field Set
8 min
Scan Time
100 kHz
Modulation Frequency
25 °C
Temperature
9.508 GHz
Microwave Frequency
2016-03-22
Date
Remarks
EPR Chart A
Sample
Spectrum No
Scan Range

**Hexa(3,5-di-*tert*-butylphenyl)ethane $^t$Bu-1$_2$:**

At 3.8 °C

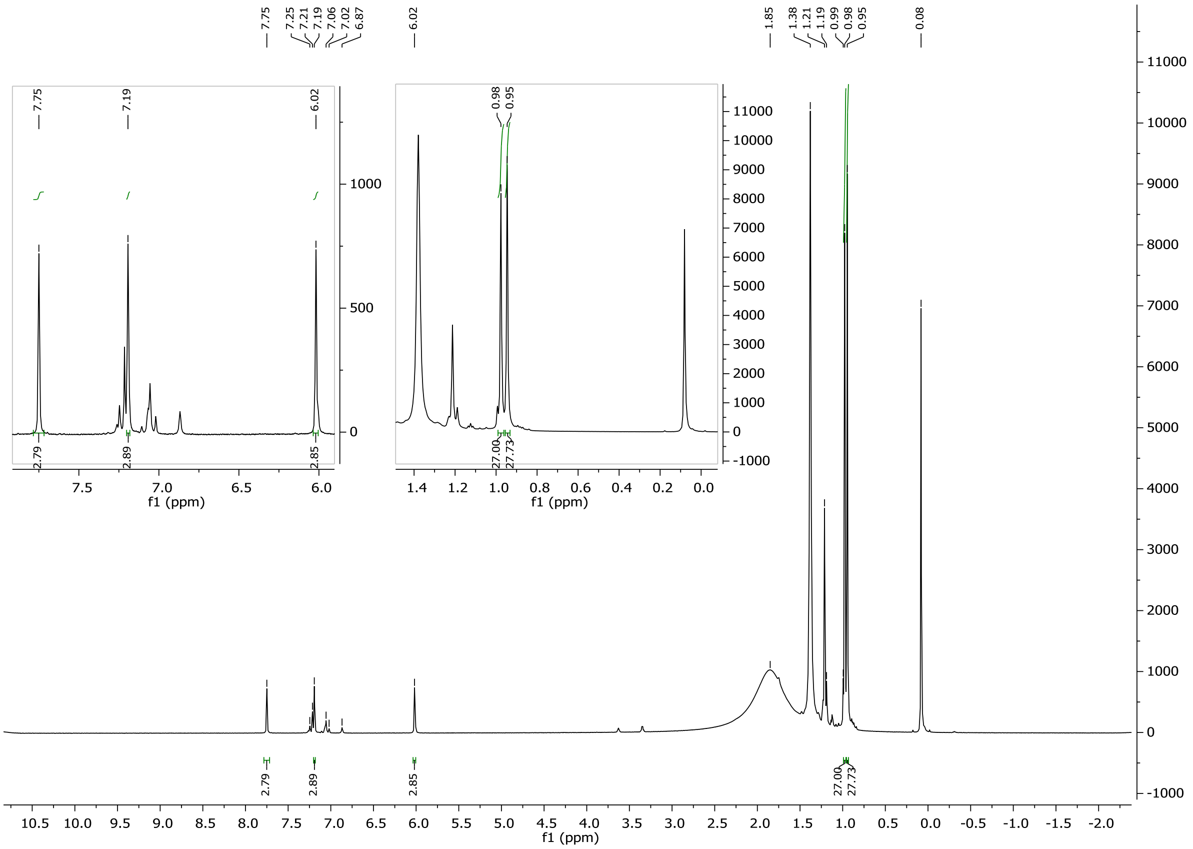

At 25 °C

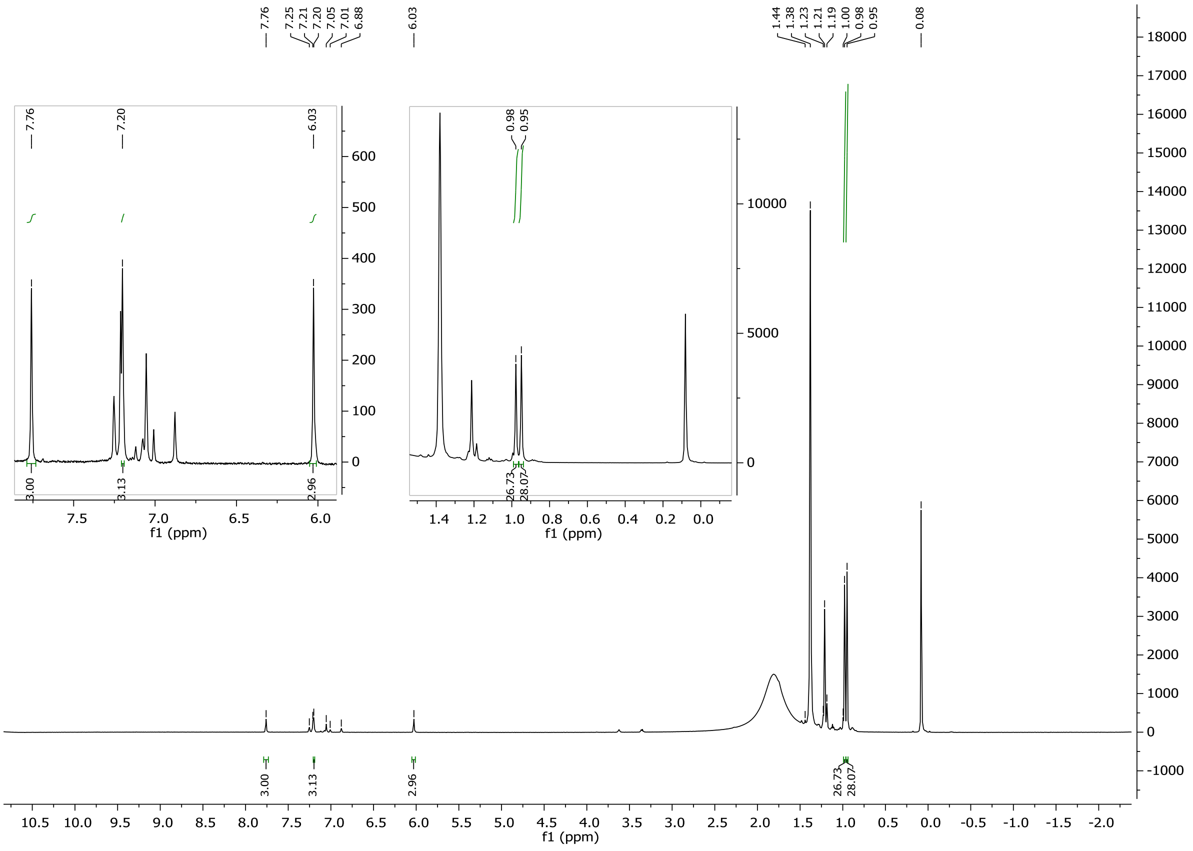

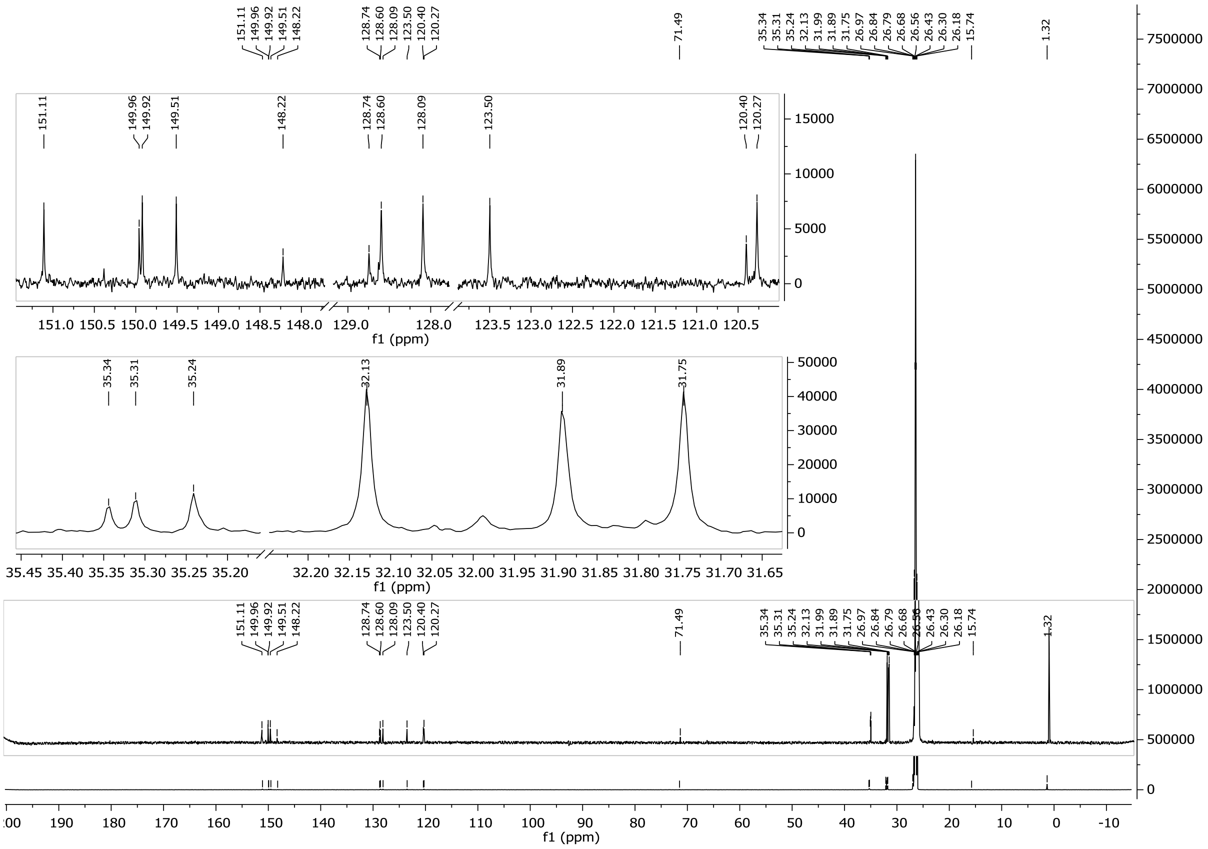

151.11
149.96
149.92
149.51
148.22
128.74
128.60
128.09
123.50
120.40
120.27
71.49
35.34
35.31
35.24
32.13
31.99
31.89
31.75
26.97
26.84
26.79
26.68
26.56
26.43
26.30
26.18
15.74
1.32
f1 (ppm)


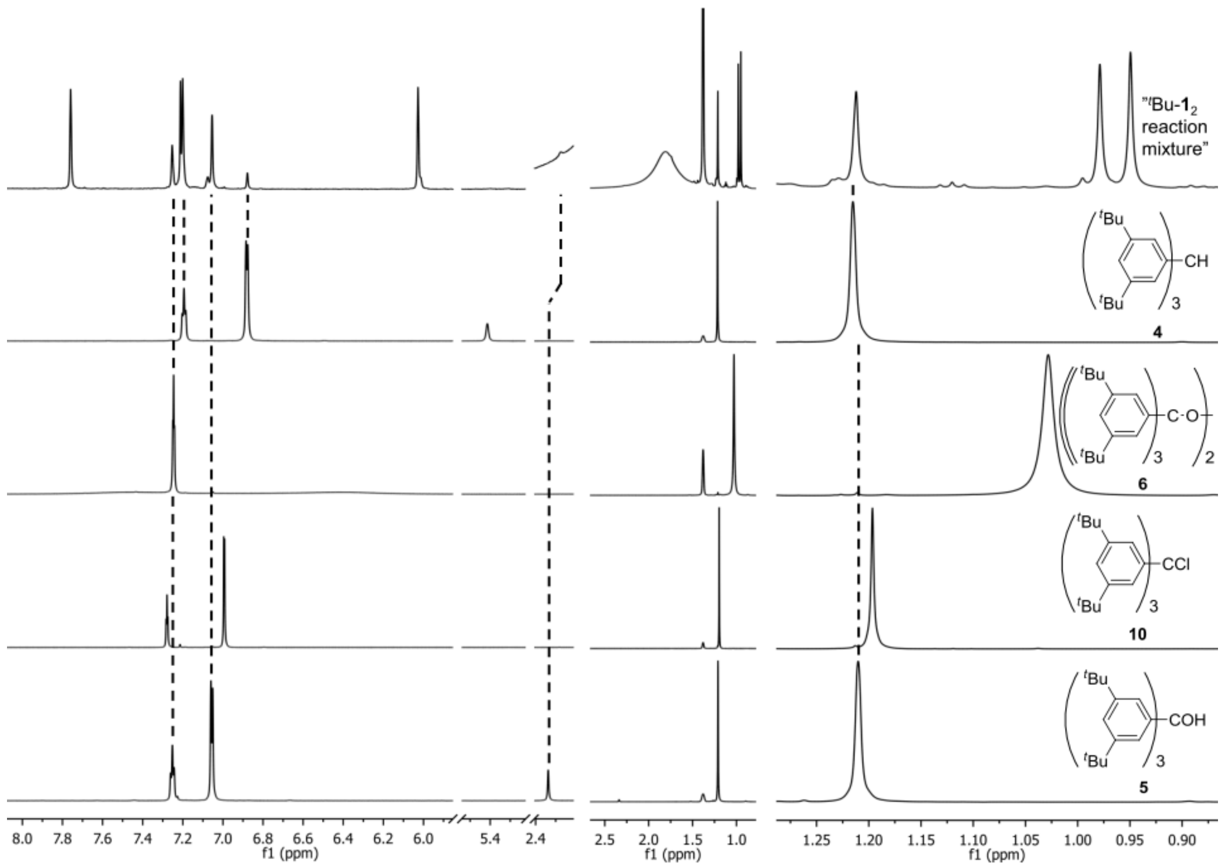

"ᵗBu-1₂ reaction mixture"
ᵗBu
CH
4
C-O
6
CCl
10
COH
5
f1 (ppm)

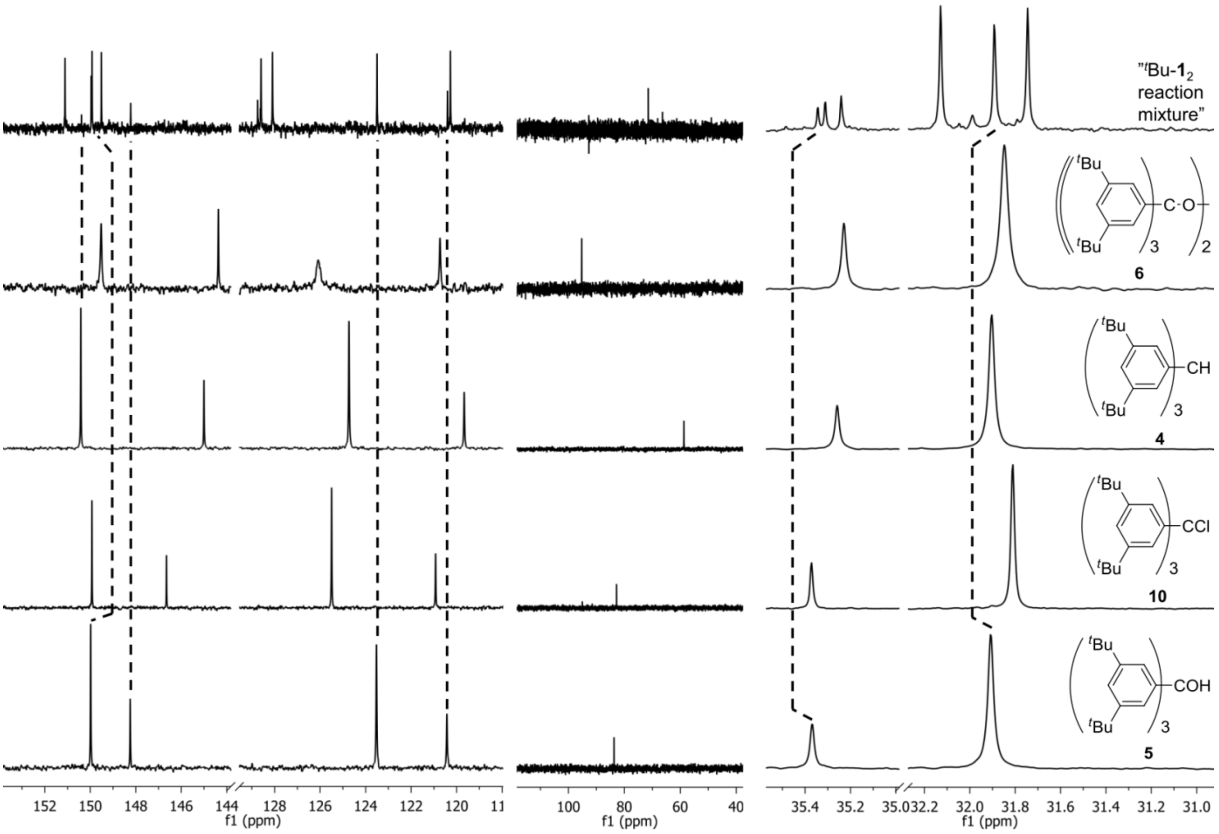
"$^t$Bu-$\mathbf{1}_2$ reaction mixture"
6
4
CH
10
CCl
5
COH
C-O
f1 (ppm)
f1 (ppm)
f1 (ppm)

**HSQC**

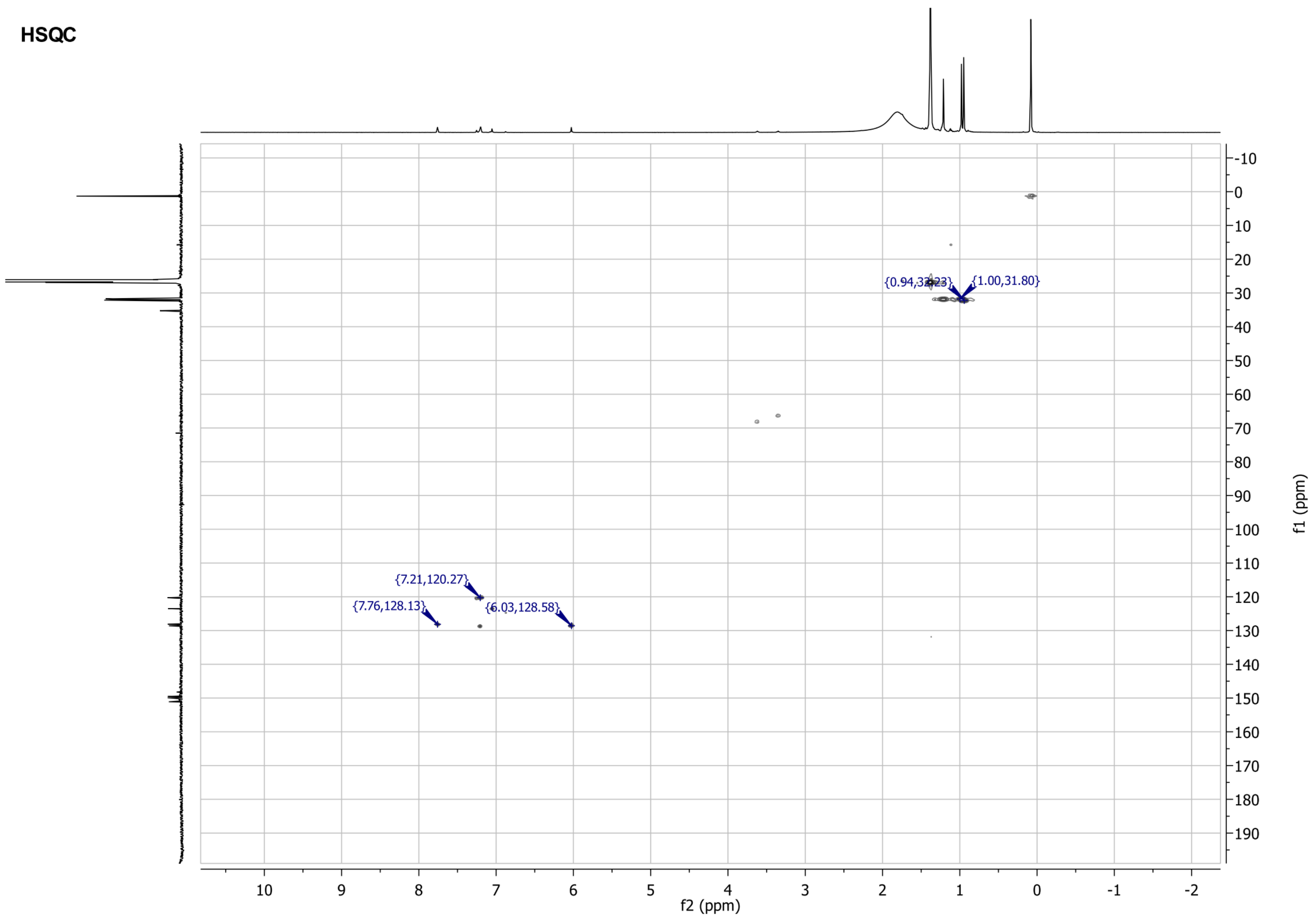

**HSQC**

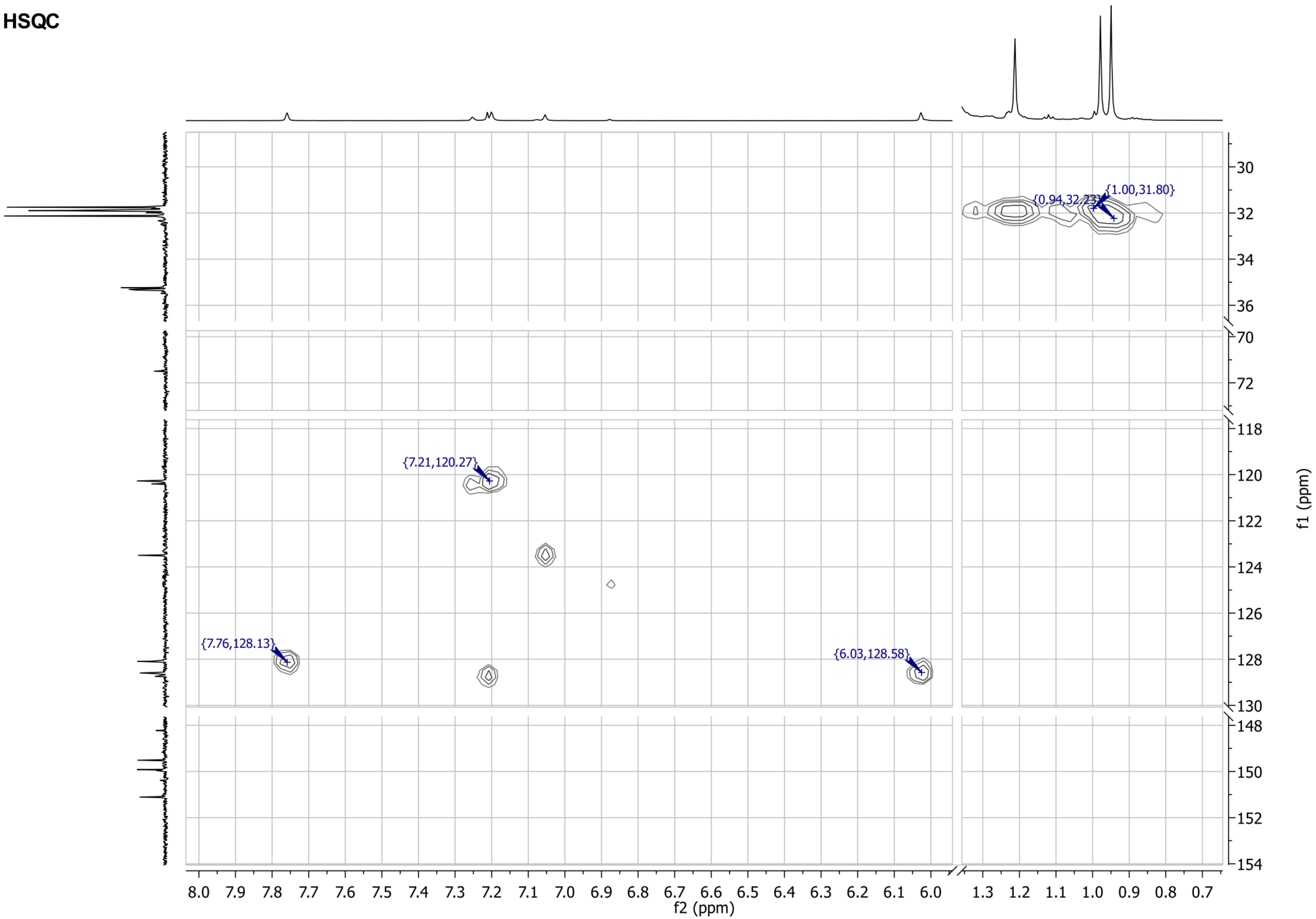

HMBC

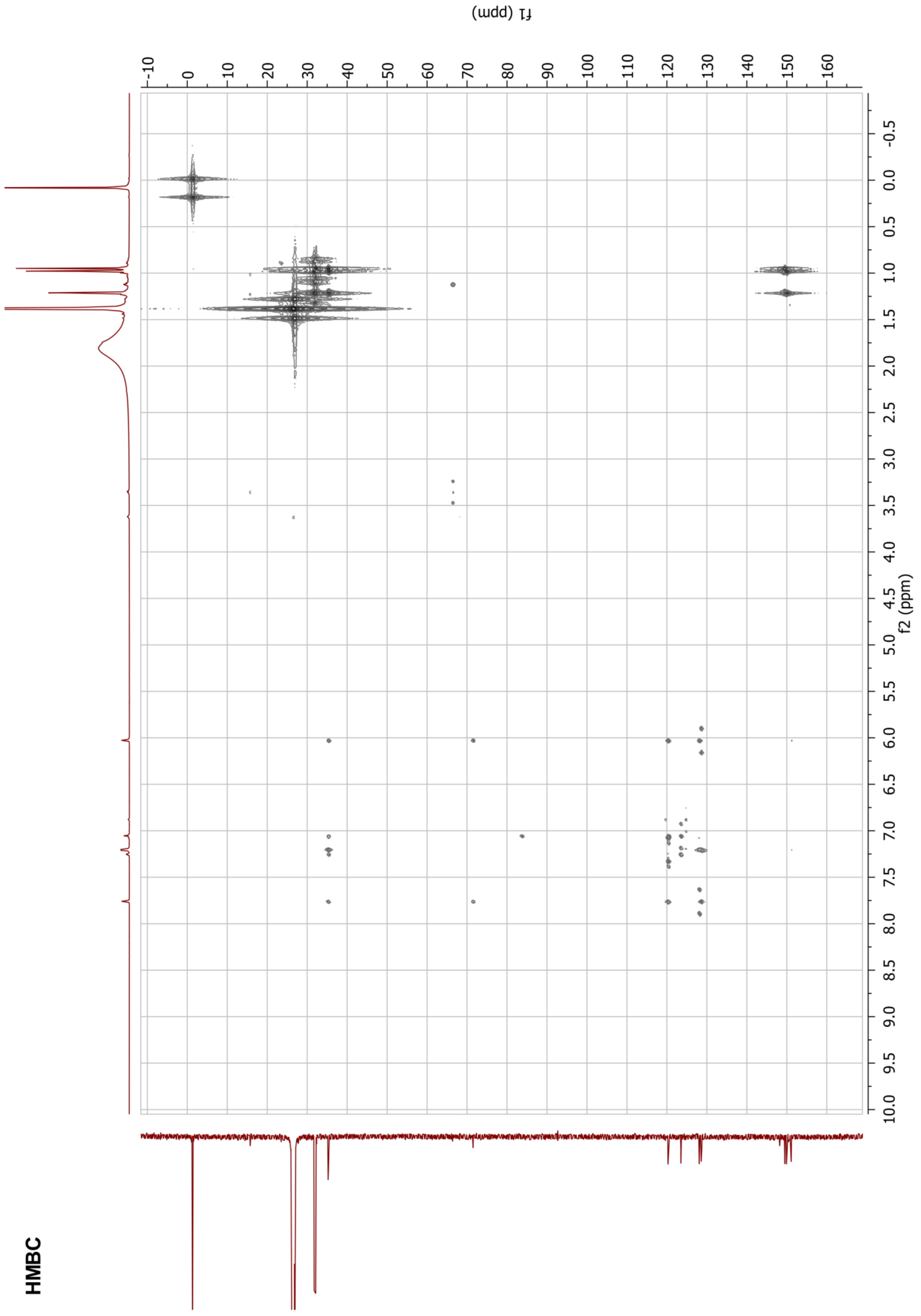

## HMBC

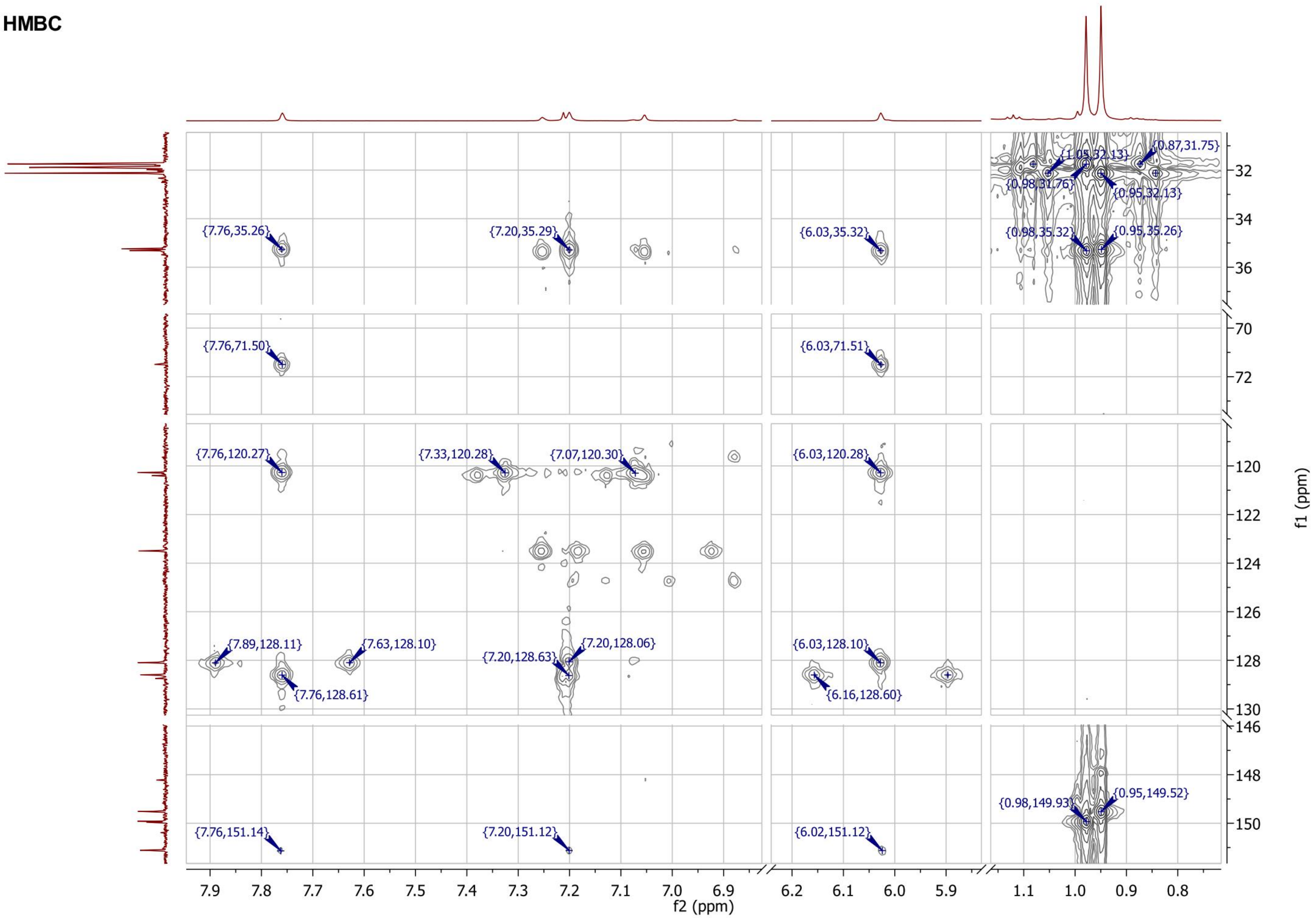

{7.76,35.26}
{7.20,35.29}
{6.03,35.32}
{0.98,35.32}
{0.95,35.26}
{1.05,32.13}
{0.87,31.75}
{0.98,31.76}
{0.95,32.13}
{7.76,71.50}
{6.03,71.51}
{7.76,120.27}
{7.33,120.28}
{7.07,120.30}
{6.03,120.28}
{7.89,128.11}
{7.63,128.10}
{7.20,128.63}
{7.20,128.06}
{7.76,128.61}
{6.03,128.10}
{6.16,128.60}
{0.98,149.93}
{0.95,149.52}
{7.76,151.14}
{7.20,151.12}
{6.02,151.12}
f2 (ppm)
f1 (ppm)

## Computations

For the computations Gaussian 09, Revision D.01 was used.[8] Dissociation energies were derived through $\Delta E = 2 \cdot E(\text{R-}\mathbf{1}\bullet) - E(\text{R-}\mathbf{1}_2)$ with $E = D_e$, $H_d^{298}$, $S_d^{298}$, $G_d^{298}$.

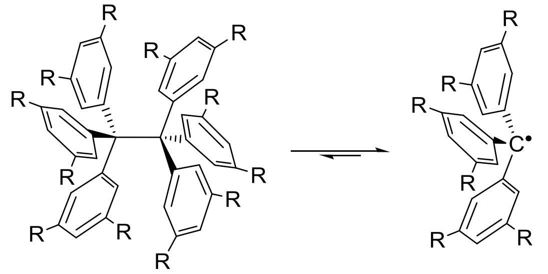


**Figure S3.** Nomenclature of the computed compounds.

### Overview

**Table S3.** Computed free energies $\Delta G_d^{298}$ for the equilibrium between R-$\mathbf{1}_2$ and R-$\mathbf{1}\bullet$ with C-PCM:cyclohexane as solvent model in kcal mol$^{-1}$. Dispersion corrections were obtained *via* $E_{disp} = D_e$(B3LYP-D3(BJ))–$D_e$(B3LYP). The isotropic polarizability $\alpha$ of R-$\mathbf{1}_2$ is the average value of B3LYP-D3(BJ) and M06-2X; basis set: cc-pVDZ.

| Prop. | R = | H | Me | $^i$Pr | $^t$Bu | Cy | 1-Ad |
|---|---|---|---|---|---|---|---|
| $\Delta G_{d298}$[a] | M06-2X | –5.2 | –8.3 | –0.3 | –5.1 | 3.1 | 12.8 |
| | B3LYP-D3(BJ) | –8.1 | –8.3 | 7.8 | 10.5 | 19.1 | 33.0 |
| $E_{disp}$[a] | | 31.1 | 41.5 | 61.2 | 67.7 | 67.3 | 98.3 |
| $\alpha$[b] | averaged | 0.7 | 1.0 | 1.4 | 1.7 | 2.1 | 2.8 |
| $R_{CC}$[c] | M06-2X | 1.692 | 1.712 | 1.669 | 1.666 | 1.700 | 1.663 |
| | B3LYP-D3(BJ) | 1.701 | 1.725 | 1.665 | 1.662 | 1.703 | 1.662 |

[a]Kcal mol$^{-1}$ [b]nm$^3$; max. deviation $\Delta\alpha$ = 0.02 nm$^3$; [c]Å.

## Dissociation Energies

**Table S4.** H-**1**$_2$ → 2 H-**1**•

| | | $D_e$ kcal mol$^{-1}$ | $\Delta H_d^0$ kcal mol$^{-1}$ | $\Delta H_d^{298}$ kcal mol$^{-1}$ | $\Delta S_d^{298}$ kcal mol$^{-1}$ K$^{-1}$ | $T\Delta S_d^{298}$ kcal mol$^{-1}$ | $\Delta G_d^{298}$ kcal mol$^{-1}$ | $\alpha$ nm$^3$ |
|---|---|---|---|---|---|---|---|---|
| | B3LYP | –18.22 | –22.18 | –21.58 | 55.19 | 16.45 | –38.04 | 0.59 |
| *Vacuum* | B3LYP-D3(BJ) | 12.99 | 8.46 | 9.05 | 55.17 | 16.45 | –7.40 | 0.58 |
| | M06-2X | 17.22 | 12.31 | 12.90 | 57.61 | 17.18 | –4.28 | 0.57 |
| | B3LYP | –18.98 | –23.05 | –22.46 | 53.42 | 15.93 | –38.39 | 0.69 |
| *C-PCM:CyH* | B3LYP-D3(BJ) | 12.16 | 7.70 | 8.29 | 55.09 | 16.43 | –8.14 | 0.69 |
| | M06-2X | 16.18 | 11.39 | 11.98 | 57.48 | 17.14 | –5.15 | 0.67 |

**Table S5.** Me-**1**$_2$ → 2 Me-**1**•

| | | $D_e$ kcal mol$^{-1}$ | $\Delta H_d^0$ kcal mol$^{-1}$ | $\Delta H_d^{298}$ kcal mol$^{-1}$ | $\Delta S_d^{298}$ kcal mol$^{-1}$ K$^{-1}$ | $T\Delta S_d^{298}$ kcal mol$^{-1}$ | $\Delta G_d^{298}$ kcal mol$^{-1}$ | $\alpha$ nm$^3$ |
|---|---|---|---|---|---|---|---|---|
| | B3LYP | –17.97 | 6.98 | –25.25 | 34.33 | 10.24 | –35.49 | 0.81 |
| *Vacuum* | B3LYP-D3(BJ) | 20.61 | 12.09 | 12.68 | 37.84 | 11.28 | 1.40 | 0.81 |
| | M06-2X | 21.47 | 16.21 | 16.80 | 81.45 | 24.28 | –7.48 | 0.79 |
| | B3LYP | –19.05 | –23.26 | –22.67 | 68.23 | 20.34 | –43.01 | 0.96 |
| *C-PCM:CyH* | B3LYP-D3(BJ) | 19.40 | 14.54 | 15.13 | 78.74 | 23.48 | -8.34 | 0.95 |
| | M06-2X | 20.03 | 15.08 | 15.67 | 80.33 | 23.95 | -8.28 | 0.93 |

**Table S6.** $^i$Pr-**1**$_2$ → 2 $^i$Pr-**1**•

| | | $D_e$ kcal mol$^{-1}$ | $\Delta H_d^0$ kcal mol$^{-1}$ | $\Delta H_d^{298}$ kcal mol$^{-1}$ | $\Delta S_d^{298}$ kcal mol$^{-1}$ K$^{-1}$ | $T\Delta S_d^{298}$ kcal mol$^{-1}$ | $\Delta G_d^{298}$ kcal mol$^{-1}$ | $\alpha$ nm$^3$ |
|---|---|---|---|---|---|---|---|---|
| | B3LYP | –26.10 | –30.73 | –30.14 | 82.13 | 24.49 | –54.63 | 1.21 |
| *Vacuum* | B3LYP-D3(BJ) | 35.16 | 27.49 | 28.08 | 51.06 | 15.22 | 12.86 | 1.20 |
| | M06-2X | 30.91 | 22.92 | 23.51 | 38.54 | 11.49 | 12.02 | 1.18 |
| | B3LYP | –27.26 | –31.87 | –31.27 | 76.68 | 22.86 | –54.14 | 1.43 |
| *C-PCM:CyH* | B3LYP-D3(BJ) | 33.93 | 28.03 | 28.62 | 70.01 | 20.87 | 7.75 | 1.42 |
| | M06-2X | 28.39 | 22.08 | 22.67 | 77.13 | 23.00 | –0.32 | 1.40 |

**Table S7.** $^t$Bu-**1**$_2$ → 2 $^t$Bu-**1**•

| | | $D_e$ kcal mol$^{-1}$ | $\Delta H_d^0$ kcal mol$^{-1}$ | $\Delta H_d^{298}$ kcal mol$^{-1}$ | $\Delta S_d^{298}$ kcal mol$^{-1}$ K$^{-1}$ | $T\Delta S_d^{298}$ kcal mol$^{-1}$ | $\Delta G_d^{298}$ kcal mol$^{-1}$ | $\alpha$ nm$^3$ |
|---|---|---|---|---|---|---|---|---|
| | B3LYP | –28.44 | –30.49 | –29.89 | 86.07 | 25.66 | –55.56 | 1.41 |
| *Vacuum* | B3LYP-D3(BJ) | 39.40 | 37.37 | 37.96 | 111.22 | 33.16 | 4.81 | 1.40 |
| | M06-2X | 28.94 | 30.15 | 30.75 | 110.46 | 32.93 | –2.19 | 1.38 |
| | B3LYP | –29.81 | –31.81 | –31.21 | 84.65 | 25.24 | –56.45 | 1.67 |
| *C-PCM:CyH* | B3LYP-D3(BJ) | 37.90 | 34.23 | 34.82 | 81.63 | 24.34 | 10.49 | 1.66 |
| | M06-2X | 27.30 | 29.14 | 29.74 | 116.71 | 34.80 | -5.06 | 1.64 |

**Table S8.** Cy-**1**$_2$ → 2 Cy-**1**•

| | | $D_e$ kcal mol$^{-1}$ | $\Delta H_d^0$ kcal mol$^{-1}$ | $\Delta H_d^{298}$ kcal mol$^{-1}$ | $\Delta S_d^{298}$ kcal mol$^{-1}$ K$^{-1}$ | $T\Delta S_d^{298}$ kcal mol$^{-1}$ | $\Delta G_d^{298}$ kcal mol$^{-1}$ | $\alpha$ nm$^3$ |
|---|---|---|---|---|---|---|---|---|
| | B3LYP | –19.84 | –24.00 | –23.40 | 53.78 | 16.03 | –39.44 | 1.79 |
| *Vacuum* | B3LYP-D3(BJ) | 47.74 | 41.87 | 42.46 | 86.15 | 25.69 | 16.77 | 1.78 |
| | M06-2X | 34.18 | 34.81 | 35.40 | 110.82 | 33.04 | 2.36 | 1.75 |

| | | | | | | | | |
|---|---|---|---|---|---|---|---|---|
| | B3LYP | –21.15 | –16.67 | –16.08 | 115.52 | 34.44 | –50.52 | 2.09 |
| *C-PCM:CyH* | B3LYP-D3(BJ) | 46.12 | 41.86 | 42.46 | 78.51 | 23.41 | 19.05 | 2.08 |
| | M06-2X | 33.46 | 29.57 | 30.16 | 90.64 | 27.03 | 3.14 | 2.05 |

**Table S9.** Ad-$\mathbf{1}_2$ → 2 Ad-**1**•

| | | $D_e$ kcal mol$^{-1}$ | $\Delta H_d^0$ kcal mol$^{-1}$ | $\Delta H_d^{298}$ kcal mol$^{-1}$ | $\Delta S_d^{298}$ kcal mol$^{-1}$ K$^{-1}$ | $T\Delta S_d^{298}$ kcal mol$^{-1}$ | $\Delta G_d^{298}$ kcal mol$^{-1}$ | $\alpha$ nm$^3$ |
|---|---|---|---|---|---|---|---|---|
| | B3LYP | –28.82 | –41.36 | –40.76 | –2.63 | –0.78 | –39.98 | 2.40 |
| *Vacuum* | B3LYP-D3(BJ) | 60.70 | 55.48 | 56.07 | 104.44 | 31.14 | 24.93 | 2.38 |
| | M06-2X | 40.99 | 37.49 | 38.08 | 77.61 | 23.14 | 14.94 | 2.36 |
| | B3LYP | –32.85 | –36.61 | –36.02 | 56.57 | 16.87 | –52.89 | 2.85 |
| *C-PCM:CyH* | B3LYP-D3(BJ) | 65.42 | 59.03 | 59.63 | 89.31 | 26.63 | 33.00 | 2.83 |
| | M06-2X | 38.68 | 38.23 | 38.83 | 87.32 | 26.04 | 12.79 | 2.81 |

## Energies

Energies of the optimized structures are given in hartree. Throughout cc-pVDZ was used as basis set. Symmetry for dimers was restricted to $S_6$, for radical monomers to $C_3$.

**Table S10.** Radical monomers R-1• in gas phase in hartree.

| | | |
|---|---|---|
| | H-**1**• | |
| method | electronic energy | ZPVE |
| B3LYP | –733.0764598 | 0.277351 |
| B3YLP-D3(BJ) | –733.1568231 | 0.277795 |
| M06-2X | –732.7935605 | 0.278935 |
| | Me-**1**• | |
| method | electronic energy | ZPVE |
| B3LYP | –968.9813788 | 0.438957 |
| B3YLP-D3(BJ) | –969.0952808 | 0.439616 |
| M06-2X | –968.5820561 | 0.442479 |
| | iPr-**1**• | |
| method | electronic energy | ZPVE |
| B3LYP | –1440.746265 | 0.778034 |
| B3YLP-D3(BJ) | –1440.940249 | 0.780041 |
| M06-2X | –1440.128500 | 0.783835 |
| | tBu-**1**• | |
| method | electronic energy | ZPVE |
| B3LYP | –1676.609727 | 0.944188 |
| B3YLP-D3(BJ) | –1676.854105 | 0.946712 |
| M06-2X | –1675.902194 | 0.952281 |
| | Cy-**1**• | |
| method | electronic energy | ZPVE |
| B3LYP | –2141.201519 | 1.176190 |
| B3YLP-D3(BJ) | –2141.493126 | 1.177676 |
| M06-2X | –2140.318802 | 1.183848 |
| | Ad-**1**• | |
| method | electronic energy | ZPVE |
| B3LYP | –3070.299518 | 1.608311 |
| B3YLP-D3(BJ) | –3070.782260 | 1.615265 |
| M06-2X | –3069.168355 | 1.625587 |

**Table S11.** Radical monomers R-**1**• with C–PCM:cyclohexane in hartree.

| | | |
|---|---|---|
| | H-**1**• | |
| method | electronic energy | ZPVE |
| B3LYP | –733.0787485 | 0.277293 |
| B3YLP-D3(BJ) | –733.1590829 | 0.277742 |
| M06-2X | –732.7964368 | 0.278896 |

| | Me-**1**• | |
|---|---|---|
| method | electronic energy | ZPVE |
| B3LYP | –968.9838193 | 0.438955 |
| B3YLP-D3(BJ) | –969.0976825 | 0.439616 |
| M06-2X | –968.5851891 | 0.439509 |
| | $^{i}$Pr-**1**• | |
| method | electronic energy | ZPVE |
| B3LYP | –1440.748523 | 0.777475 |
| B3YLP-D3(BJ) | –1440.942455 | 0.779511 |
| M06-2X | –1440.13230 | 0.782526 |
| | $^{t}$Bu-**1**• | |
| method | electronic energy | ZPVE |
| B3LYP | –1676.612119 | 0.943492 |
| B3YLP-D3(BJ) | –1676.856469 | 0.946265 |
| M06-2X | –1675.905340 | 0.951729 |
| | Cy-**1**• | |
| method | electronic energy | ZPVE |
| B3LYP | –2141.204065 | 1.175433 |
| B3YLP-D3(BJ) | –2141.495535 | 1.176557 |
| M06-2X | –2140.322113 | 1.182930 |
| | Ad-**1**• | |
| method | electronic energy | ZPVE |
| B3LYP | –3070.304520 | 1.610998 |
| B3YLP-D3(BJ) | –3070.779891 | 1.612403 |
| M06-2X | –3069.172026 | 1.623864 |

**Table S12.** Dimers R-$1_2$ in gas phase in hartree.

| | H-$\mathbf{1}_2$ | |
|---|---|---|
| method | electronic energy | ZPVE |
| B3LYP | –1466.123877 | 0.560584 |
| B3YLP-D3(BJ) | –1466.334350 | 0.562632 |
| M06-2X | –1465.614561 | 0.565697 |
| | Me-$\mathbf{1}_2$ | |
| method | electronic energy | ZPVE |
| B3LYP | –1937.934124 | 0.88536 |
| B3YLP-D3(BJ) | –1938.223412 | 0.88806 |
| M06-2X | –1937.198322 | 0.89488 |
| | $^{i}$Pr-$\mathbf{1}_2$ | |
| method | electronic energy | ZPVE |
| B3LYP | –2881.450942 | 1.563903 |
| B3YLP-D3(BJ) | –2881.936535 | 1.569315 |
| M06-2X | –2880.306254 | 1.575694 |
| | $^{t}$Bu-$\mathbf{1}_2$ | |
| method | electronic energy | ZPVE |
| B3LYP | –3353.17413 | 1.893794 |
| B3YLP-D3(BJ) | –3353.770992 | 1.900866 |
| M06-2X | –3351.850503 | 1.909038 |
| | Cy-$\mathbf{1}_2$ | |
| method | electronic energy | ZPVE |
| B3LYP | –4282.371419 | 2.357848 |
| B3YLP-D3(BJ) | –4283.062331 | 2.365418 |
| M06-2X | –4280.69208 | 2.374363 |
| | Ad-$\mathbf{1}_2$ | |
| method | electronic energy | ZPVE |
| B3LYP | –6140.553102 | 3.225925 |
| B3YLP-D3(BJ) | –6141.661258 | 3.241213 |
| M06-2X | –6138.402039 | 3.256664 |

**Table S13.** Dimers R-$1_2$ with C–PCM:cyclohexane in hartree.

| | | |
|---|---|---|
| | H-$\mathbf{1}_2$ | |
| method | electronic energy | ZPVE |
| B3LYP | –1466.127253 | 0.560676 |
| B3YLP-D3(BJ) | –1466.337552 | 0.562399 |
| M06-2X | –1465.618663 | 0.565398 |
| | Me-$\mathbf{1}_2$ | |
| method | electronic energy | ZPVE |
| B3LYP | –1937.937282 | 0.88472 |
| B3YLP-D3(BJ) | –1938.226278 | 0.88733 |
| M06-2X | –1937.202304 | 0.89283 |
| | $^i$Pr-$\mathbf{1}_2$ | |
| method | electronic energy | ZPVE |
| B3LYP | –2881.453611 | 1.562604 |
| B3YLP-D3(BJ) | –2881.93898 | 1.568258 |
| M06-2X | –2880.309852 | 1.576509 |
| | $^t$Bu-$\mathbf{1}_2$ | |
| method | electronic energy | ZPVE |
| B3LYP | –3353.176729 | 1.892288 |
| B3YLP-D3(BJ) | –3353.773333 | 1.899777 |
| M06-2X | –3351.854193 | 1.907694 |
| | Cy-$\mathbf{1}_2$ | |
| method | electronic energy | ZPVE |
| B3LYP | –4282.374429 | 2.352386 |
| B3YLP-D3(BJ) | –4283.064564 | 2.361481 |
| M06-2X | –4280.697544 | 2.375467 |
| | Ad-$\mathbf{1}_2$ | |
| method | electronic energy | ZPVE |
| B3LYP | –6140.556687 | 3.226542 |
| B3YLP-D3(BJ) | –6141.664032 | 3.239013 |
| M06-2X | –6138.405688 | 3.252795 |

## Cartesian coordinates

Cartesian coordinates of the optimized structures in Å. Throughout cc-pVDZ was used as basis set. Symmetry for dimers was restricted to $S_6$, for radical monomers to $C_3$.

*Radical Monomers*

**Table S14.** Radical monomers R-**1**• in gas phase.

H-1•

B3LYP

| | | | |
|---|---|---|---|
| C | –0.0041320000 | 4.306242000 | 0.005173000 |
| C | –0.994923000 | 3.599193000 | –0.685961000 |
| C | –0.997353000 | 2.205724000 | –0.688678000 |
| C | 0.000000000 | 1.466248000 | –0.004476000 |
| C | 0.996037000 | 2.204112000 | 0.683638000 |
| C | 0.989352000 | 3.597352000 | 0.690927000 |
| C | –1.269808000 | –0.733124000 | –0.004476000 |
| C | –2.406835000 | –0.239462000 | 0.683638000 |
| C | –3.610074000 | –0.941872000 | 0.690927000 |
| C | –3.727249000 | –2.156700000 | 0.005173000 |
| C | –2.619531000 | –2.661225000 | –0.685961000 |
| C | –1.411537000 | –1.966595000 | –0.688678000 |
| C | 3.614454000 | –0.937968000 | –0.685961000 |
| C | 2.408890000 | –0.239129000 | –0.688678000 |
| C | 1.269808000 | –0.733124000 | –0.004476000 |
| C | 1.410798000 | –1.964650000 | 0.683638000 |
| C | 2.620723000 | –2.655480000 | 0.690927000 |
| C | 3.731381000 | –2.149543000 | 0.005173000 |
| C | 0.000000000 | 0.000000000 | –0.006407000 |
| H | –0.005805000 | 5.398457000 | 0.009307000 |
| H | –1.769817000 | 4.139524000 | –1.235219000 |
| H | –1.766906000 | 1.670231000 | –1.246951000 |
| H | 1.766957000 | 1.666735000 | 1.238109000 |
| H | 1.762539000 | 4.136433000 | 1.243833000 |
| H | –2.326913000 | 0.696862000 | 1.238109000 |
| H | –4.463525000 | –0.541813000 | 1.243833000 |
| H | –4.672299000 | –2.704256000 | 0.009307000 |
| H | –2.700024000 | –3.602468000 | –1.235219000 |
| H | –0.563010000 | –2.365301000 | –1.246951000 |
| H | 4.469841000 | –0.537056000 | –1.235219000 |
| H | 2.329915000 | 0.695070000 | –1.246951000 |
| H | 0.559956000 | –2.363597000 | 1.238109000 |
| H | 2.700987000 | –3.594620000 | 1.243833000 |
| H | 4.678104000 | –2.694201000 | 0.009307000 |

B3LYP-D3(BJ)

| | | | |
|---|---|---|---|
| C | –0.0050170000 | 4.295913000 | 0.005953000 |
| C | –1.004913000 | 3.589678000 | –0.672302000 |
| C | –1.006436000 | 2.197331000 | –0.676402000 |
| C | 0.000000000 | 1.461201000 | –0.005152000 |
| C | 1.004607000 | 2.195716000 | 0.670647000 |
| C | 0.997995000 | 3.587908000 | 0.677971000 |
| C | –1.265437000 | –0.730601000 | –0.005152000 |
| C | –2.403850000 | –0.227843000 | 0.670647000 |
| C | –3.606217000 | –0.929665000 | 0.677971000 |
| C | –3.717861000 | –2.152301000 | 0.005953000 |
| C | –2.606296000 | –2.665119000 | –0.672302000 |
| C | –1.399727000 | –1.970265000 | –0.676402000 |
| C | 3.611209000 | –0.924559000 | –0.672302000 |
| C | 2.406163000 | –0.227066000 | –0.676402000 |
| C | 1.265437000 | –0.730601000 | –0.005152000 |
| C | 1.399242000 | –1.967874000 | 0.670647000 |
| C | 2.608222000 | –2.658243000 | 0.677971000 |
| C | 3.722878000 | –2.143612000 | 0.005953000 |
| C | 0.000000000 | 0.000000000 | –0.007370000 |
| H | –0.007018000 | 5.387722000 | 0.010721000 |
| H | –1.786781000 | 4.130447000 | –1.210288000 |
| H | –1.780819000 | 1.658438000 | –1.223571000 |
| H | 1.780844000 | 1.654984000 | 1.213346000 |
| H | 1.777791000 | 4.127429000 | 1.220240000 |
| H | –2.323680000 | 0.714764000 | 1.213346000 |
| H | –4.463354000 | –0.524103000 | 1.220240000 |
| H | –4.662395000 | –2.699939000 | 0.010721000 |
| H | –2.683681000 | –3.612621000 | –1.210288000 |
| H | –0.545840000 | –2.371454000 | –1.223571000 |
| H | 4.470462000 | –0.517826000 | –1.210288000 |
| H | 2.326659000 | 0.713015000 | –1.223571000 |
| H | 0.542837000 | –2.369748000 | 1.213346000 |
| H | 2.685563000 | –3.603326000 | 1.220240000 |
| H | 4.669413000 | –2.687783000 | 0.010721000 |

M06-2X

| | | | |
|---|---|---|---|
| C | –0.009746000 | 4.287674000 | 0.012981000 |
| C | –1.010430000 | 3.585615000 | –0.660633000 |
| C | –1.009460000 | 2.195627000 | –0.671177000 |
| C | 0.000000000 | 1.462588000 | –0.011568000 |
| C | 1.005698000 | 2.191010000 | 0.658872000 |
| C | 0.996592000 | 3.580879000 | 0.672898000 |
| C | –1.266638000 | –0.731294000 | –0.011568000 |
| C | –2.400319000 | –0.224545000 | 0.658872000 |
| C | –3.599428000 | –0.927365000 | 0.672898000 |
| C | –3.708362000 | –2.152277000 | 0.012981000 |
| C | –2.600019000 | –2.667865000 | –0.660633000 |
| C | –1.396739000 | –1.972031000 | –0.671177000 |
| C | 3.610448000 | –0.917750000 | –0.660633000 |
| C | 2.406198000 | –0.223595000 | –0.671177000 |
| C | 1.266638000 | –0.731294000 | –0.011568000 |
| C | 1.394621000 | –1.966465000 | 0.658872000 |
| C | 2.602836000 | –2.653513000 | 0.672898000 |
| C | 3.718108000 | –2.135397000 | 0.012981000 |
| C | 0.000000000 | 0.000000000 | –0.016846000 |
| H | –0.013283000 | 5.378171000 | 0.023589000 |
| H | –1.796783000 | 4.126758000 | –1.189067000 |
| H | –1.788371000 | 1.656231000 | –1.212386000 |
| H | 1.787582000 | 1.648233000 | 1.192251000 |
| H | 1.778850000 | 4.118248000 | 1.211075000 |
| H | –2.321202000 | 0.723975000 | 1.192251000 |
| H | –4.455932000 | –0.518595000 | 1.211075000 |
| H | –4.650992000 | –2.700589000 | 0.023589000 |
| H | –2.675486000 | –3.619439000 | –1.189067000 |
| H | –0.540153000 | –2.376890000 | –1.212386000 |
| H | 4.472269000 | –0.507319000 | –1.189067000 |
| H | 2.328523000 | 0.720659000 | –1.212386000 |
| H | 0.533620000 | –2.372208000 | 1.192251000 |
| H | 2.677082000 | –3.599653000 | 1.211075000 |
| H | 4.664275000 | –2.677582000 | 0.023589000 |

Me-**1**•

B3LYP

| | | | |
|---|---|---|---|
| C | 0.000000000 | 4.286130000 | –0.003990000 |
| C | 0.999001000 | 3.603003000 | 0.700056000 |
| C | 0.990246000 | 2.202433000 | 0.693477000 |
| C | –0.001489000 | 1.467099000 | 0.007034000 |
| C | –0.997453000 | 2.200645000 | –0.687450000 |
| C | –1.004513000 | 3.594773000 | –0.706131000 |
| C | 1.271289000 | –0.732260000 | 0.007034000 |
| C | 2.404540000 | –0.236503000 | –0.687450000 |
| C | 3.615421000 | –0.927453000 | –0.706131000 |
| C | 3.711898000 | –2.143065000 | –0.003990000 |
| C | 2.620791000 | –2.666661000 | 0.700056000 |
| C | 1.412240000 | –1.958795000 | 0.693477000 |
| C | –3.619792000 | –0.936341000 | 0.700056000 |
| C | –2.402486000 | –0.243638000 | 0.693477000 |
| C | –1.269800000 | –0.734839000 | 0.007034000 |
| C | –1.407088000 | –1.964142000 | –0.687450000 |
| C | –2.610908000 | –2.667321000 | –0.706131000 |
| C | –3.711898000 | –2.143065000 | –0.003990000 |
| C | 0.000000000 | 0.000000000 | 0.007276000 |
| C | 2.067494000 | 4.351435000 | 1.463704000 |
| C | –2.060762000 | 4.360061000 | –1.469008000 |
| C | 4.806304000 | –0.395358000 | –1.469008000 |
| C | 2.734706000 | –3.966219000 | 1.463704000 |
| C | –4.802200000 | –0.385215000 | 1.463704000 |
| C | –2.745543000 | –3.964702000 | –1.469008000 |
| H | 0.000285000 | 5.380419000 | –0.008165000 |
| H | 1.756675000 | 1.662662000 | 1.253608000 |

| | | | |
|---|---|---|---|
| H | –1.762767000 | 1.655971000 | –1.243394000 |
| H | 2.315497000 | 0.698616000 | –1.243394000 |
| H | 4.659437000 | –2.690457000 | –0.008165000 |
| H | 0.561570000 | –2.352656000 | 1.253608000 |
| H | –2.318245000 | 0.689995000 | 1.253608000 |
| H | –0.552729000 | –2.354587000 | –1.243394000 |
| H | –4.659723000 | –2.689962000 | –0.008165000 |
| H | 2.046088000 | 4.095477000 | 2.536393000 |
| H | 1.940449000 | 5.440626000 | 1.375569000 |
| H | 3.075438000 | 4.098182000 | 1.094104000 |
| H | –1.609233000 | 5.003017000 | –2.243482000 |
| H | –2.636760000 | 5.021336000 | –0.799473000 |
| H | –2.770731000 | 3.682609000 | –1.965866000 |
| H | 5.137356000 | –1.107872000 | –2.243482000 |
| H | 5.666985000 | –0.227167000 | –0.799473000 |
| H | 4.574598000 | 0.558219000 | –1.965866000 |
| H | 2.523743000 | –3.819703000 | 2.536393000 |
| H | 3.741496000 | –4.400791000 | 1.375569000 |
| H | 2.011410000 | –4.712498000 | 1.094104000 |
| H | –4.569832000 | –0.275774000 | 2.536393000 |
| H | –5.681944000 | –1.039835000 | 1.375569000 |
| H | –5.086848000 | 0.614317000 | 1.094104000 |
| H | –3.528123000 | –3.895145000 | –2.243482000 |
| H | –3.030225000 | –4.794169000 | –0.799473000 |
| H | –1.803867000 | –4.240828000 | –1.965866000 |

B3LYP-D3(BJ)

| | | | |
|---|---|---|---|
| C | 0.000000000 | 4.276471000 | 0.005309000 |
| C | –1.012098000 | 3.593662000 | –0.678299000 |
| C | –1.002135000 | 2.194630000 | –0.676159000 |
| C | 0.002070000 | 1.461687000 | –0.008972000 |
| C | 1.010812000 | 2.191785000 | 0.667269000 |
| C | 1.018350000 | 3.584307000 | 0.684938000 |
| C | –1.266894000 | –0.729051000 | –0.008972000 |
| C | –2.403548000 | –0.220503000 | 0.667269000 |
| C | –3.613276000 | –0.910236000 | 0.684938000 |
| C | –3.703533000 | –2.138236000 | 0.005309000 |
| C | –2.606153000 | –2.673333000 | –0.678299000 |
| C | –1.399537000 | –1.965189000 | –0.676159000 |
| C | 3.618251000 | –0.920329000 | –0.678299000 |
| C | 2.401673000 | –0.229440000 | –0.676159000 |
| C | 1.264823000 | –0.732637000 | –0.008972000 |
| C | 1.392735000 | –1.971282000 | 0.667269000 |
| C | 2.594926000 | –2.674071000 | 0.684938000 |
| C | 3.703533000 | –2.138236000 | 0.005309000 |
| C | 0.000000000 | 0.000000000 | –0.010315000 |
| C | –2.097289000 | 4.340097000 | –1.415878000 |
| C | 2.090055000 | 4.347642000 | 1.423842000 |
| C | –4.810196000 | –0.363780000 | 1.423842000 |
| C | –2.709990000 | –3.986354000 | –1.415878000 |
| C | 4.807279000 | –0.353744000 | –1.415878000 |
| C | 2.720141000 | –3.983862000 | 1.423842000 |
| H | –0.000529000 | 5.370272000 | 0.011109000 |
| H | –1.777149000 | 1.653796000 | –1.222064000 |
| H | 1.785217000 | 1.644950000 | 1.207069000 |
| H | –2.317178000 | 0.723568000 | 1.207069000 |
| H | –4.650528000 | –2.685594000 | 0.011109000 |
| H | –0.543655000 | –2.365954000 | –1.222064000 |
| H | 2.320804000 | 0.712158000 | –1.222064000 |
| H | 0.531960000 | –2.368519000 | 1.207069000 |
| H | 4.651056000 | –2.684678000 | 0.011109000 |
| H | –2.097602000 | 4.085007000 | –2.488636000 |
| H | –1.971046000 | 5.429038000 | –1.327597000 |
| H | –3.094972000 | 4.081229000 | –1.023910000 |
| H | 1.654935000 | 4.989898000 | 2.207729000 |
| H | 2.650228000 | 5.008389000 | 0.741039000 |
| H | 2.809656000 | 3.668476000 | 1.903588000 |
| H | –5.148846000 | –1.061734000 | 2.207729000 |
| H | –5.662506000 | –0.209030000 | 0.741039000 |
| H | –4.581822000 | 0.598996000 | 1.903588000 |
| H | –2.488919000 | –3.859080000 | –2.488636000 |
| H | –3.716162000 | –4.421495000 | –1.327597000 |
| H | –1.986962000 | –4.720939000 | –1.023910000 |
| H | 4.586521000 | –0.225927000 | –2.488636000 |
| H | 5.687208000 | –1.007543000 | –1.327597000 |
| H | 5.081934000 | 0.639710000 | –1.023910000 |
| H | 3.493911000 | –3.928165000 | 2.207729000 |
| H | 3.012279000 | –4.799359000 | 0.741039000 |
| H | 1.772165000 | –4.267472000 | 1.903588000 |

M06-2X

| | | | |
|---|---|---|---|
| C | 0.000000000 | 4.268756000 | –0.009395000 |
| C | 1.010324000 | 3.591864000 | 0.674094000 |
| C | 1.000024000 | 2.195039000 | 0.679607000 |
| C | –0.003686000 | 1.463257000 | 0.020652000 |
| C | –1.011963000 | 2.186029000 | –0.655367000 |
| C | –1.017270000 | 3.575689000 | –0.681539000 |
| C | 1.269060000 | –0.728437000 | 0.020652000 |
| C | 2.399138000 | –0.216629000 | –0.655367000 |
| C | 3.605273000 | –0.906863000 | –0.681539000 |
| C | 3.696851000 | –2.134378000 | –0.009395000 |
| C | 2.605484000 | –2.670898000 | 0.674094000 |
| C | 1.400948000 | –1.963565000 | 0.679607000 |
| C | –3.615807000 | –0.920966000 | 0.674094000 |
| C | –2.400971000 | –0.231474000 | 0.679607000 |
| C | –1.265375000 | –0.734820000 | 0.020652000 |
| C | –1.387175000 | –1.969400000 | –0.655367000 |
| C | –2.588003000 | –2.668826000 | –0.681539000 |
| C | –3.696851000 | –2.134378000 | –0.009395000 |
| C | 0.000000000 | 0.000000000 | 0.025526000 |
| C | 2.100323000 | 4.340234000 | 1.398125000 |
| C | –2.085675000 | 4.337286000 | –1.423204000 |
| C | 4.799038000 | –0.362396000 | –1.423204000 |
| C | 2.708591000 | –3.989050000 | 1.398125000 |
| C | –4.808914000 | –0.351184000 | 1.398125000 |
| C | –2.713363000 | –3.974891000 | –1.423204000 |
| H | –0.000135000 | 5.362238000 | –0.021928000 |
| H | 1.778830000 | 1.655831000 | 1.223743000 |
| H | –1.789664000 | 1.635755000 | –1.188714000 |
| H | 2.311437000 | 0.732017000 | –1.188714000 |
| H | 4.643902000 | –2.681002000 | –0.021928000 |
| H | 0.544577000 | –2.368427000 | 1.223743000 |
| H | –2.323406000 | 0.712596000 | 1.223743000 |
| H | –0.521773000 | –2.367772000 | –1.188714000 |
| H | –4.643767000 | –2.681236000 | –0.021928000 |
| H | 2.149819000 | 4.038762000 | 2.454679000 |
| H | 1.935248000 | 5.424901000 | 1.359085000 |
| H | 3.083918000 | 4.128910000 | 0.951968000 |
| H | –1.644376000 | 4.964702000 | –2.212039000 |
| H | –2.635652000 | 5.005112000 | –0.743269000 |
| H | –2.807912000 | 3.656545000 | –1.892424000 |
| H | 5.121746000 | –1.058280000 | –2.212039000 |
| H | 5.652380000 | –0.220015000 | –0.743269000 |
| H | 4.570617000 | 0.603450000 | –1.892424000 |
| H | 2.422761000 | –3.881179000 | 2.454679000 |
| H | 3.730478000 | –4.388424000 | 1.359085000 |
| H | 2.033782000 | –4.735206000 | 0.951968000 |
| H | –4.572580000 | –0.157583000 | 2.454679000 |
| H | –5.665726000 | –1.036477000 | 1.359085000 |
| H | –5.117700000 | 0.606296000 | 0.951968000 |
| H | –3.477370000 | –3.906423000 | –2.212039000 |
| H | –3.016728000 | –4.785098000 | –0.743269000 |
| H | –1.762705000 | –4.259995000 | –1.892424000 |

$^{i}$Pr-**1•**

B3LYP

| | | | |
|---|---|---|---|
| C | 0.007898000 | 4.279797000 | –0.017282000 |
| C | 0.989078000 | 3.599970000 | 0.721498000 |
| C | 0.980125000 | 2.201578000 | 0.712001000 |
| C | 0.000000000 | 1.467970000 | 0.001653000 |
| C | –0.977861000 | 2.197711000 | –0.715668000 |
| C | –0.978964000 | 3.595773000 | –0.744579000 |
| C | 1.271300000 | –0.733985000 | 0.001653000 |
| C | 2.392204000 | –0.252002000 | –0.715668000 |
| C | 3.603513000 | –0.950079000 | –0.744579000 |
| C | 3.702464000 | –2.146739000 | –0.017282000 |
| C | 2.623127000 | –2.656552000 | 0.721498000 |
| C | 1.416560000 | –1.949602000 | 0.712001000 |
| C | –3.612205000 | –0.943419000 | 0.721498000 |
| C | –2.396684000 | –0.251976000 | 0.712001000 |
| C | –1.271300000 | –0.733985000 | 0.001653000 |
| C | –1.414343000 | –1.945708000 | –0.715668000 |
| C | –2.624549000 | –2.645694000 | –0.744579000 |
| C | –3.710362000 | –2.133058000 | –0.017282000 |
| C | 0.000000000 | 0.000000000 | 0.005231000 |
| C | –2.777845000 | –3.932951000 | –1.548479000 |
| C | –2.544237000 | –3.706375000 | –3.052535000 |
| C | –1.873731000 | –5.056261000 | –1.011066000 |
| C | –4.805269000 | –0.439865000 | 1.527522000 |
| C | –5.272673000 | 0.951855000 | 1.068664000 |
| C | –4.518734000 | –0.457477000 | 3.039872000 |
| C | 4.794958000 | –0.439209000 | –1.548479000 |
| C | 5.315716000 | 0.905431000 | –1.011066000 |
| C | 4.481933000 | –0.350187000 | –3.052535000 |
| C | 2.783569000 | –3.941552000 | 1.527522000 |
| C | 1.812006000 | –5.042197000 | 1.068664000 |
| C | 2.655554000 | –3.684600000 | 3.039872000 |
| C | 2.021700000 | 4.381417000 | 1.527522000 |
| C | 1.863180000 | 4.142077000 | 3.039872000 |
| C | 3.460668000 | 4.090341000 | 1.068664000 |
| C | –2.017113000 | 4.372160000 | –1.548479000 |

| | | | |
|---|---|---|---|
| C | –1.937696000 | 4.056561000 | –3.052535000 |
| C | –3.441984000 | 4.150829000 | –1.011066000 |
| H | 0.011807000 | 5.374149000 | –0.025173000 |
| H | 1.727470000 | 1.651023000 | 1.285999000 |
| H | –1.728831000 | 1.643570000 | –1.281075000 |
| H | 2.287789000 | 0.675427000 | –1.281075000 |
| H | 4.648246000 | –2.697299000 | –0.025173000 |
| H | 0.566093000 | –2.321544000 | 1.285999000 |
| H | –2.293563000 | 0.670521000 | 1.285999000 |
| H | –0.558957000 | –2.318997000 | –1.281075000 |
| H | –4.660052000 | –2.676850000 | –0.025173000 |
| H | –3.824214000 | –4.262663000 | –1.423295000 |
| H | –1.507016000 | –3.391660000 | –3.255243000 |
| H | –3.214334000 | –2.926804000 | –3.448371000 |
| H | –2.726527000 | –4.634903000 | –3.618879000 |
| H | –0.806975000 | –4.799921000 | –1.120746000 |
| H | –2.049266000 | –5.995756000 | –1.561374000 |
| H | –2.064193000 | –5.245629000 | 0.057455000 |
| H | –5.634351000 | –1.145014000 | 1.341360000 |
| H | –5.512200000 | 0.958873000 | –0.006698000 |
| H | –4.497376000 | 1.715858000 | 1.244486000 |
| H | –6.175311000 | 1.261331000 | 1.621474000 |
| H | –4.223327000 | –1.463258000 | 3.378570000 |
| H | –5.412097000 | –0.152448000 | 3.610099000 |
| H | –3.702566000 | 0.236681000 | 3.301348000 |
| H | 5.603682000 | –1.180535000 | –1.423295000 |
| H | 4.560341000 | 1.701099000 | –1.120746000 |
| H | 6.217111000 | 1.223162000 | –1.561374000 |
| H | 5.574944000 | 0.835171000 | 0.057455000 |
| H | 3.690772000 | 0.390716000 | –3.255243000 |
| H | 4.141853000 | –1.320293000 | –3.448371000 |
| H | 5.377208000 | –0.043790000 | –3.618879000 |
| H | 3.808787000 | –4.306984000 | 1.341360000 |
| H | 1.925692000 | –5.253141000 | –0.006698000 |
| H | 0.762711000 | –4.752771000 | 1.244486000 |
| H | 1.995311000 | –5.978642000 | 1.621474000 |
| H | 3.378882000 | –2.925880000 | 3.378570000 |
| H | 2.838073000 | –4.610790000 | 3.610099000 |
| H | 1.646311000 | –3.324857000 | 3.301348000 |
| H | 1.825564000 | 5.451998000 | 1.341360000 |
| H | 0.844445000 | 4.389138000 | 3.378570000 |
| H | 2.574025000 | 4.763238000 | 3.610099000 |
| H | 2.056255000 | 3.088176000 | 3.301348000 |
| H | 3.586508000 | 4.294269000 | –0.006698000 |
| H | 3.734665000 | 3.036913000 | 1.244486000 |
| H | 4.180000000 | 4.717311000 | 1.621474000 |
| H | –1.779468000 | 5.443198000 | –1.423295000 |
| H | –2.183756000 | 3.000944000 | –3.255243000 |
| H | –0.927519000 | 4.247097000 | –3.448371000 |
| H | –2.650680000 | 4.678694000 | –3.618879000 |
| H | –3.753366000 | 3.098822000 | –1.120746000 |
| H | –4.167844000 | 4.772595000 | –1.561374000 |
| H | –3.510751000 | 4.410457000 | 0.057455000 |

B3LYP-D3(BJ)

| | | | |
|---|---|---|---|
| C | 0.005978000 | –4.271011000 | 0.006705000 |
| C | 1.019270000 | –3.587971000 | –0.681550000 |
| C | 1.006784000 | –2.191944000 | –0.677896000 |
| C | 0.000000000 | –1.461496000 | –0.006617000 |
| C | –1.004723000 | –2.190079000 | 0.669708000 |
| C | –1.011514000 | –3.585992000 | 0.686864000 |
| C | 1.265693000 | 0.730748000 | –0.006617000 |
| C | 2.399026000 | 0.224924000 | 0.669708000 |
| C | 3.611317000 | 0.916999000 | 0.686864000 |
| C | 3.695815000 | 2.140683000 | 0.006705000 |
| C | 2.597639000 | 2.676700000 | –0.681550000 |
| C | 1.394888000 | 1.967873000 | –0.677896000 |
| C | –3.616909000 | 0.911272000 | –0.681550000 |
| C | –2.401672000 | 0.224072000 | –0.677896000 |
| C | –1.265693000 | 0.730748000 | –0.006617000 |
| C | –1.394303000 | 1.965155000 | 0.669708000 |
| C | –2.599803000 | 2.668992000 | 0.686864000 |
| C | –3.701793000 | 2.130329000 | 0.006705000 |
| C | 0.000000000 | 0.000000000 | –0.009235000 |
| C | –2.725870000 | 3.980608000 | 1.446125000 |
| C | –2.599877000 | 3.760612000 | 2.962012000 |
| C | –1.713434000 | 5.024946000 | 0.953633000 |
| C | –4.818055000 | 0.366261000 | –1.438473000 |
| C | –5.204767000 | –1.041219000 | –0.961644000 |
| C | –4.573978000 | 0.388033000 | –2.955803000 |
| C | 4.810243000 | 0.370368000 | 1.446125000 |
| C | 5.208448000 | –1.028596000 | 0.953633000 |
| C | 4.556724000 | 0.371253000 | 2.962012000 |
| C | 2.726219000 | 3.989428000 | –1.438473000 |
| C | 1.700662000 | 5.028070000 | –0.961644000 |
| C | 2.623035000 | 3.767165000 | –2.955803000 |
| C | 2.091836000 | –4.355689000 | –1.438473000 |
| C | 1.950943000 | –4.155198000 | –2.955803000 |
| C | 3.504106000 | –3.986851000 | –0.961644000 |
| C | –2.084373000 | –4.350976000 | 1.446125000 |
| C | –1.956848000 | –4.131865000 | 2.962012000 |
| C | –3.495014000 | –3.996350000 | 0.953633000 |
| H | 0.008714000 | –5.364806000 | 0.011717000 |
| H | 1.774133000 | –1.641353000 | –1.222723000 |
| H | –1.775314000 | –1.637546000 | 1.207754000 |
| H | 2.305813000 | –0.718694000 | 1.207754000 |
| H | 4.641702000 | 2.689950000 | 0.011717000 |
| H | 0.534387000 | 2.357121000 | –1.222723000 |
| H | –2.308520000 | –0.715767000 | –1.222723000 |
| H | –0.530499000 | 2.356240000 | 1.207754000 |
| H | –4.650416000 | 2.674857000 | 0.011717000 |
| H | –3.738209000 | 4.372721000 | 1.247200000 |
| H | –1.601799000 | 3.370790000 | 3.222165000 |
| H | –3.346441000 | 3.035199000 | 3.321393000 |
| H | –2.744990000 | 4.706654000 | 3.509197000 |
| H | –0.678659000 | 4.701616000 | 1.150312000 |
| H | –1.864827000 | 5.988699000 | 1.466684000 |
| H | –1.810106000 | 5.192644000 | –0.130518000 |
| H | –5.666410000 | 1.039367000 | –1.225491000 |
| H | –5.395609000 | –1.055791000 | 0.122877000 |
| H | –4.402641000 | –1.767476000 | –1.169162000 |
| H | –6.113963000 | –1.392340000 | –1.476425000 |
| H | –4.326126000 | 1.403122000 | –3.303544000 |
| H | –5.467272000 | 0.043163000 | –3.502201000 |
| H | –3.735375000 | –0.273701000 | –3.229145000 |
| H | 5.655993000 | 1.051023000 | 1.247200000 |
| H | 4.411048000 | –1.763072000 | 1.150312000 |
| H | 6.118779000 | –1.379363000 | 1.466684000 |
| H | 5.402015000 | –1.028724000 | –0.130518000 |
| H | 3.720089000 | –0.298196000 | 3.222165000 |
| H | 4.301780000 | 1.380504000 | 3.321393000 |
| H | 5.448577000 | 0.023904000 | 3.509197000 |
| H | 3.733324000 | 4.387572000 | –1.225491000 |
| H | 1.783463000 | 5.200630000 | 0.122877000 |
| H | 0.670641000 | 4.696537000 | –1.169162000 |
| H | 1.851180000 | 5.991018000 | –1.476425000 |
| H | 3.378202000 | 3.044974000 | –3.303544000 |
| H | 2.771016000 | 4.713214000 | –3.502201000 |
| H | 1.630656000 | 3.371780000 | –3.229145000 |
| H | 1.933087000 | –5.426939000 | –1.225491000 |
| H | 0.947924000 | –4.448096000 | –3.303544000 |
| H | 2.696255000 | –4.756378000 | –3.502201000 |
| H | 2.104719000 | –3.098079000 | –3.229145000 |
| H | 3.612146000 | –4.144839000 | 0.122877000 |
| H | 3.732000000 | –2.929061000 | –1.169162000 |
| H | 4.262784000 | –4.598677000 | –1.476425000 |
| H | –1.917783000 | –5.423745000 | 1.247200000 |
| H | –2.118290000 | –3.072594000 | 3.222165000 |
| H | –0.955339000 | –4.415703000 | 3.321393000 |
| H | –2.703587000 | –4.730558000 | 3.509197000 |
| H | –3.732389000 | –2.938544000 | 1.150312000 |
| H | –4.253953000 | –4.609337000 | 1.466684000 |
| H | –3.591909000 | –4.163920000 | –0.130518000 |

M06-2X

| | | | |
|---|---|---|---|
| C | –0.017450000 | 4.265052000 | 0.100908000 |
| C | 1.024271000 | 3.585767000 | 0.751930000 |
| C | 1.019405000 | 2.195136000 | 0.743279000 |
| C | 0.000000000 | 1.463430000 | 0.091615000 |
| C | –1.019294000 | 2.184514000 | –0.554622000 |
| C | –1.042200000 | 3.580970000 | –0.552474000 |
| C | 1.267367000 | –0.731715000 | 0.091615000 |
| C | 2.401491000 | –0.209522000 | –0.554622000 |
| C | 3.622311000 | –0.887913000 | –0.552474000 |
| C | 3.702368000 | –2.117414000 | 0.100908000 |
| C | 2.593230000 | –2.679929000 | 0.751930000 |
| C | 1.391341000 | –1.980399000 | 0.743279000 |
| C | –3.617501000 | –0.905839000 | 0.751930000 |
| C | –2.410746000 | –0.214737000 | 0.743279000 |
| C | –1.267367000 | –0.731715000 | 0.091615000 |
| C | –1.382197000 | –1.974992000 | –0.554622000 |
| C | –2.580111000 | –2.693057000 | –0.552474000 |
| C | –3.684918000 | –2.147638000 | 0.100908000 |
| C | 0.000000000 | 0.000000000 | 0.095868000 |
| C | –2.667337000 | –4.042061000 | –1.242056000 |
| C | –2.421966000 | –3.918492000 | –2.748614000 |
| C | –1.698069000 | –5.045281000 | –0.609643000 |
| C | –4.869903000 | –0.355890000 | 1.414819000 |
| C | –5.850088000 | 0.145575000 | 0.346698000 |
| C | –4.595572000 | 0.741992000 | 2.439230000 |
| C | 4.834196000 | –0.288951000 | –1.242056000 |
| C | 5.218376000 | 1.052069000 | –0.609643000 |
| C | 4.604497000 | –0.138238000 | –2.748614000 |
| C | 2.743161000 | –4.039515000 | 1.414819000 |

| | | | |
|---|---|---|---|
| C | 2.798972000 | –5.139112000 | 0.346698000 |
| C | 1.655202000 | –4.350878000 | 2.439230000 |
| C | 2.126742000 | 4.395405000 | 1.414819000 |
| C | 2.940370000 | 3.608886000 | 2.439230000 |
| C | 3.051116000 | 4.993537000 | 0.346698000 |
| C | –2.166859000 | 4.331012000 | –1.242056000 |
| C | –2.182530000 | 4.056730000 | –2.748614000 |
| C | –3.520307000 | 3.993211000 | –0.609643000 |
| H | –0.031146000 | 5.359318000 | 0.114208000 |
| H | 1.798094000 | 1.641128000 | 1.267689000 |
| H | –1.801501000 | 1.629977000 | –1.077034000 |
| H | 2.312353000 | 0.745157000 | –1.077034000 |
| H | 4.656879000 | –2.652685000 | 0.114208000 |
| H | 0.522212000 | –2.377759000 | 1.267689000 |
| H | –2.320306000 | 0.736631000 | 1.267689000 |
| H | –0.510851000 | –2.375135000 | –1.077034000 |
| H | –4.625732000 | –2.706632000 | 0.114208000 |
| H | –3.692884000 | –4.417497000 | –1.093392000 |
| H | –1.399760000 | –3.562126000 | –2.950312000 |
| H | –3.123798000 | –3.207153000 | –3.207290000 |
| H | –2.541140000 | –4.894369000 | –3.243162000 |
| H | –0.656600000 | –4.705657000 | –0.729763000 |
| H | –1.787937000 | –6.031968000 | –1.088741000 |
| H | –1.894091000 | –5.160642000 | 0.466423000 |
| H | –5.348388000 | –1.200533000 | 1.939361000 |
| H | –6.089033000 | –0.642736000 | –0.381653000 |
| H | –5.408080000 | 0.991165000 | –0.203721000 |
| H | –6.788846000 | 0.488386000 | 0.807948000 |
| H | –3.870889000 | 0.415545000 | 3.199052000 |
| H | –5.528039000 | 1.024316000 | 2.948849000 |
| H | –4.198050000 | 1.647038000 | 1.953379000 |
| H | 5.672107000 | –0.989382000 | –1.093392000 |
| H | 4.403519000 | 1.784196000 | –0.729763000 |
| H | 6.117806000 | 1.467585000 | –1.088741000 |
| H | 5.416293000 | 0.939990000 | 0.466423000 |
| H | 3.784772000 | 0.568835000 | –2.950312000 |
| H | 4.339374000 | –1.101712000 | –3.207290000 |
| H | 5.509217000 | 0.246493000 | –3.243162000 |
| H | 3.713886000 | –4.031573000 | 1.939361000 |
| H | 3.601142000 | –4.951889000 | –0.381653000 |
| H | 1.845666000 | –5.179117000 | –0.203721000 |
| H | 2.971468000 | –6.123506000 | 0.807948000 |
| H | 1.575572000 | –3.560061000 | 3.199052000 |
| H | 1.876936000 | –5.299581000 | 2.948849000 |
| H | 0.672648000 | –4.459137000 | 1.953379000 |
| H | 1.634502000 | 5.232106000 | 1.939361000 |
| H | 2.295317000 | 3.144516000 | 3.199052000 |
| H | 3.651104000 | 4.275264000 | 2.948849000 |
| H | 3.525401000 | 2.812099000 | 1.953379000 |
| H | 2.487891000 | 5.594625000 | –0.381653000 |
| H | 3.562414000 | 4.187953000 | –0.203721000 |
| H | 3.817378000 | 5.635120000 | 0.807948000 |
| H | –1.979223000 | 5.406880000 | –1.093392000 |
| H | –2.385012000 | 2.993291000 | –2.950312000 |
| H | –1.215577000 | 4.308864000 | –3.207290000 |
| H | –2.968078000 | 4.647876000 | –3.243162000 |
| H | –3.746918000 | 2.921461000 | –0.729763000 |
| H | –4.329869000 | 4.564382000 | –1.088741000 |
| H | –3.522201000 | 4.220652000 | 0.466423000 |

*ᵗ*Bu-**1•**

B3LYP

| | | | |
|---|---|---|---|
| C | 0.001959000 | 4.274715000 | –0.010861000 |
| C | –1.000557000 | 3.592829000 | –0.733232000 |
| C | –0.990376000 | 2.198445000 | –0.713799000 |
| C | 0.000000000 | 1.467875000 | –0.008270000 |
| C | 0.983756000 | 2.197854000 | 0.690636000 |
| C | 0.998105000 | 3.600582000 | 0.708375000 |
| C | –1.271217000 | –0.733937000 | –0.008270000 |
| C | –2.395276000 | –0.246970000 | 0.690636000 |
| C | –3.617248000 | –0.935907000 | 0.708375000 |
| C | –3.702991000 | –2.135661000 | –0.010861000 |
| C | –2.611203000 | –2.662922000 | –0.733232000 |
| C | –1.408722000 | –1.956913000 | –0.713799000 |
| C | 3.611760000 | –0.929907000 | –0.733232000 |
| C | 2.399098000 | –0.241532000 | –0.713799000 |
| C | 1.271217000 | –0.733937000 | –0.008270000 |
| C | 1.411520000 | –1.950885000 | 0.690636000 |
| C | 2.619143000 | –2.664675000 | 0.708375000 |
| C | 3.701032000 | –2.139054000 | –0.010861000 |
| C | 0.000000000 | 0.000000000 | –0.009825000 |
| C | –2.052814000 | 4.404395000 | –1.517161000 |
| C | 2.084315000 | 4.341761000 | 1.516199000 |
| C | –4.802233000 | –0.365811000 | 1.516199000 |
| C | –2.787911000 | –3.979987000 | –1.517161000 |
| C | 4.840725000 | –0.424408000 | –1.517161000 |
| C | 2.717918000 | –3.975950000 | 1.516199000 |
| C | –1.343809000 | 5.282971000 | –2.574307000 |
| C | –2.835791000 | 5.311627000 | –0.539913000 |
| C | –3.064859000 | 3.502737000 | –2.246913000 |
| C | 1.961058000 | 3.962114000 | 3.010308000 |
| C | 1.962673000 | 5.872118000 | 1.399843000 |
| C | 3.482743000 | 3.931710000 | 0.999128000 |
| C | –4.411820000 | –0.282731000 | 3.010308000 |
| C | –6.066740000 | –1.236334000 | 1.399843000 |
| C | –5.146332000 | 1.050289000 | 0.999128000 |
| C | –3.903283000 | –3.805258000 | –2.574307000 |
| C | –3.182108000 | –5.111680000 | –0.539913000 |
| C | –1.501030000 | –4.405614000 | –2.246913000 |
| C | 5.247092000 | –1.477713000 | –2.574307000 |
| C | 6.017899000 | –0.199946000 | –0.539913000 |
| C | 4.565889000 | 0.902877000 | –2.246913000 |
| C | 2.450762000 | –3.679383000 | 3.010308000 |
| C | 4.104067000 | –4.635784000 | 1.399843000 |
| C | 1.663589000 | –4.981999000 | 0.999128000 |
| H | –0.635242000 | 5.989495000 | –2.115092000 |
| H | –2.082644000 | 5.873313000 | –3.141963000 |
| H | –0.781203000 | 4.661746000 | –3.289901000 |
| H | –2.174243000 | 6.015526000 | –0.011752000 |
| H | –3.361435000 | 4.710435000 | 0.219508000 |
| H | –3.588086000 | 5.905907000 | –1.085239000 |
| H | –3.626239000 | 2.864203000 | –1.547050000 |
| H | –2.575746000 | 2.850272000 | –2.987205000 |
| H | –3.795499000 | 4.126214000 | –2.786486000 |
| H | 2.734721000 | 4.478424000 | 3.603274000 |
| H | 2.084074000 | 2.879879000 | 3.169391000 |
| H | 0.975494000 | 4.250359000 | 3.410374000 |
| H | 2.759589000 | 6.353487000 | 1.988628000 |
| H | 0.998970000 | 6.239453000 | 1.786525000 |
| H | 2.067564000 | 6.215253000 | 0.358322000 |
| H | 4.271149000 | 4.452553000 | 1.568017000 |
| H | 3.600963000 | 4.193183000 | –0.065088000 |
| H | 3.657500000 | 2.849630000 | 1.101507000 |
| H | –5.245789000 | 0.129125000 | 3.603274000 |
| H | –3.536085000 | 0.364921000 | 3.169391000 |
| H | –4.168665000 | –1.280377000 | 3.410374000 |
| H | –6.882076000 | –0.786870000 | 1.988628000 |
| H | –5.903010000 | –2.254593000 | 1.786525000 |
| H | –6.416349000 | –1.317063000 | 0.358322000 |
| H | –5.991599000 | 1.472647000 | 1.568017000 |
| H | –5.431884000 | 1.021934000 | –0.065088000 |
| H | –4.296602000 | 1.742673000 | 1.101507000 |
| H | –4.869434000 | –3.544883000 | –2.115092000 |
| H | –4.045116000 | –4.740279000 | –3.141963000 |
| H | –3.646589000 | –3.007414000 | –3.289901000 |
| H | –4.122477000 | –4.890713000 | –0.011752000 |
| H | –2.398639000 | –5.266306000 | 0.219508000 |
| H | –3.320623000 | –6.060327000 | –1.085239000 |
| H | –0.667353000 | –4.572516000 | –1.547050000 |
| H | –1.180535000 | –3.655798000 | –2.987205000 |
| H | –1.675657000 | –5.350106000 | –2.786486000 |
| H | 5.504676000 | –2.444612000 | –2.115092000 |
| H | 6.127760000 | –1.133034000 | –3.141963000 |
| H | 4.427792000 | –1.654331000 | –3.289901000 |
| H | 6.296720000 | –1.124814000 | –0.011752000 |
| H | 5.760074000 | 0.555870000 | 0.219508000 |
| H | 6.908708000 | 0.154420000 | –1.085239000 |
| H | 4.293592000 | 1.708313000 | –1.547050000 |
| H | 3.756281000 | 0.805526000 | –2.987205000 |
| H | 5.471156000 | 1.223892000 | –2.786486000 |
| H | 2.511069000 | –4.607550000 | 3.603274000 |
| H | 1.452011000 | –3.244800000 | 3.169391000 |
| H | 3.193172000 | –2.969981000 | 3.410374000 |
| H | 4.122487000 | –5.566617000 | 1.988628000 |
| H | 4.904040000 | –3.984860000 | 1.786525000 |
| H | 4.348785000 | –4.898189000 | 0.358322000 |
| H | 1.720450000 | –5.925200000 | 1.568017000 |
| H | 1.830921000 | –5.215117000 | –0.065088000 |
| H | 0.639102000 | –4.592303000 | 1.101507000 |
| H | –0.003862000 | 5.363166000 | –0.017740000 |
| H | –1.738221000 | 1.635751000 | –1.268754000 |
| H | 1.735617000 | 1.640717000 | 1.250177000 |
| H | –2.288711000 | 0.682730000 | 1.250177000 |
| H | –4.642707000 | –2.684927000 | –0.017740000 |
| H | –0.547491000 | –2.323219000 | –1.268754000 |
| H | 2.285713000 | 0.687468000 | –1.268754000 |
| H | 0.553094000 | –2.323447000 | 1.250177000 |
| H | 4.646569000 | –2.678238000 | –0.017740000 |

B3LYP-D3(BJ)

| | | | |
|---|---|---|---|
| C | 0.007152000 | 4.265177000 | –0.011644000 |
| C | –1.030604000 | 3.586445000 | –0.682852000 |
| C | –1.015558000 | 2.194791000 | –0.685544000 |

| | | | |
|---|---|---|---|
| C | 0.003399000 | 1.460995000 | –0.029441000 |
| C | 1.017092000 | 2.183456000 | 0.628998000 |
| C | 1.038574000 | 3.583325000 | 0.644277000 |
| C | –1.266959000 | –0.727554000 | –0.029441000 |
| C | –2.399474000 | –0.210901000 | 0.628998000 |
| C | –3.622537000 | –0.892231000 | 0.644277000 |
| C | –3.697328000 | –2.126394000 | –0.011644000 |
| C | –2.590650000 | –2.685752000 | –0.682852000 |
| C | –1.392965000 | –1.976894000 | –0.685544000 |
| C | 3.621254000 | –0.900693000 | –0.682852000 |
| C | 2.408523000 | –0.217896000 | –0.685544000 |
| C | 1.263560000 | –0.733441000 | –0.029441000 |
| C | 1.382383000 | –1.972555000 | 0.628998000 |
| C | 2.583964000 | –2.691094000 | 0.644277000 |
| C | 3.690175000 | –2.138783000 | –0.011644000 |
| C | 0.000000000 | 0.000000000 | –0.035440000 |
| C | –2.136099000 | 4.396868000 | –1.377654000 |
| C | 2.175301000 | 4.307742000 | 1.383053000 |
| C | –4.818265000 | –0.270005000 | 1.383053000 |
| C | –2.739750000 | –4.048350000 | –1.377654000 |
| C | 4.875849000 | –0.348518000 | –1.377654000 |
| C | 2.642964000 | –4.037737000 | 1.383053000 |
| C | –1.507279000 | 5.345160000 | –2.419517000 |
| C | –2.890629000 | 5.224424000 | –0.315476000 |
| C | –3.152744000 | 3.496955000 | –2.097286000 |
| C | 2.120598000 | 3.941956000 | 2.881104000 |
| C | 2.076072000 | 5.835266000 | 1.253494000 |
| C | 3.530168000 | 3.859384000 | 0.794600000 |
| C | –4.474133000 | –0.134486000 | 2.881104000 |
| C | –6.091524000 | –1.119702000 | 1.253494000 |
| C | –5.107408000 | 1.127523000 | 0.794600000 |
| C | –3.875405000 | –3.977922000 | –2.419517000 |
| C | –3.079169000 | –5.115570000 | –0.315476000 |
| C | –1.452080000 | –4.478834000 | –2.097286000 |
| C | 5.382684000 | –1.367238000 | –2.419517000 |
| C | 5.969798000 | –0.108853000 | –0.315476000 |
| C | 4.604824000 | 0.981879000 | –2.097286000 |
| C | 2.353535000 | –3.807470000 | 2.881104000 |
| C | 4.015452000 | –4.715564000 | 1.253494000 |
| C | 1.577240000 | –4.986907000 | 0.794600000 |
| H | –0.802923000 | 6.053474000 | –1.957829000 |
| H | –2.290919000 | 5.933292000 | –2.924785000 |
| H | –0.957979000 | 4.774691000 | –3.185177000 |
| H | –2.217851000 | 5.914739000 | 0.215818000 |
| H | –3.355829000 | 4.563507000 | 0.433242000 |
| H | –3.686593000 | 5.823903000 | –0.787260000 |
| H | –3.660825000 | 2.814683000 | –1.399387000 |
| H | –2.676216000 | 2.890298000 | –2.882831000 |
| H | –3.924193000 | 4.119349000 | –2.576924000 |
| H | 2.930668000 | 4.448919000 | 3.431107000 |
| H | 2.231866000 | 2.858570000 | 3.038359000 |
| H | 1.159454000 | 4.249352000 | 3.322836000 |
| H | 2.914680000 | 6.308324000 | 1.787689000 |
| H | 1.142886000 | 6.223007000 | 1.690597000 |
| H | 2.123905000 | 6.159152000 | 0.202101000 |
| H | 4.360996000 | 4.371808000 | 1.306903000 |
| H | 3.588214000 | 4.099340000 | –0.279082000 |
| H | 3.685175000 | 2.775666000 | 0.906199000 |
| H | –5.318210000 | 0.313574000 | 3.431107000 |
| H | –3.591527000 | 0.503568000 | 3.038359000 |
| H | –4.259774000 | –1.120559000 | 3.322836000 |
| H | –6.920509000 | –0.629976000 | 1.787689000 |
| H | –5.960726000 | –2.121735000 | 1.690597000 |
| H | –6.395935000 | –1.240220000 | 0.202101000 |
| H | –5.966595000 | 1.590829000 | 1.306903000 |
| H | –5.344240000 | 1.057815000 | –0.279082000 |
| H | –4.246385000 | 1.803622000 | 0.906199000 |
| H | –4.841000000 | –3.722089000 | –1.957829000 |
| H | –3.992922000 | –4.950640000 | –2.924785000 |
| H | –3.656014000 | –3.216979000 | –3.185177000 |
| H | –4.013389000 | –4.878084000 | 0.215818000 |
| H | –2.274198000 | –5.187987000 | 0.433242000 |
| H | –3.200351000 | –6.104634000 | –0.787260000 |
| H | –0.607174000 | –4.577709000 | –1.399387000 |
| H | –1.164963000 | –3.762820000 | –2.882831000 |
| H | –1.605364000 | –5.458126000 | –2.576924000 |
| H | 5.643924000 | –2.331385000 | –1.957829000 |
| H | 6.283841000 | –0.982652000 | –2.924785000 |
| H | 4.613993000 | –1.557711000 | –3.185177000 |
| H | 6.231239000 | –1.036654000 | 0.215818000 |
| H | 5.630027000 | 0.624480000 | 0.433242000 |
| H | 6.886944000 | 0.280732000 | –0.787260000 |
| H | 4.267999000 | 1.763026000 | –1.399387000 |
| H | 3.841180000 | 0.872522000 | –2.882831000 |
| H | 5.529558000 | 1.338777000 | –2.576924000 |
| H | 2.387542000 | –4.762492000 | 3.431107000 |
| H | 1.359661000 | –3.362138000 | 3.038359000 |
| H | 3.100319000 | –3.128793000 | 3.322836000 |
| H | 4.005829000 | –5.678349000 | 1.787689000 |
| H | 4.817839000 | –4.101272000 | 1.690597000 |
| H | 4.272030000 | –4.918932000 | 0.202101000 |
| H | 1.605599000 | –5.962638000 | 1.306903000 |
| H | 1.756026000 | –5.157155000 | –0.279082000 |
| H | 0.561210000 | –4.579288000 | 0.906199000 |
| H | 0.000000000 | 5.352981000 | –0.007884000 |
| H | –1.784962000 | 1.637283000 | –1.213251000 |
| H | 1.789936000 | 1.623346000 | 1.153766000 |
| H | –2.300827000 | 0.738457000 | 1.153766000 |
| H | –4.635817000 | –2.676490000 | –0.007884000 |
| H | –0.525447000 | –2.364464000 | –1.213251000 |
| H | 2.310410000 | 0.727181000 | –1.213251000 |
| H | 0.510891000 | –2.361803000 | 1.153766000 |
| H | 4.635817000 | –2.676490000 | –0.007884000 |

M06-2X

| | | | |
|---|---|---|---|
| C | 0.005623000 | 4.259709000 | –0.008824000 |
| C | –1.028806000 | 3.584374000 | –0.683527000 |
| C | –1.017839000 | 2.194825000 | –0.686389000 |
| C | –0.000009000 | 1.462832000 | –0.032962000 |
| C | 1.010061000 | 2.178979000 | 0.627224000 |
| C | 1.030584000 | 3.577625000 | 0.648283000 |
| C | –1.266845000 | –0.731424000 | –0.032962000 |
| C | –2.392081000 | –0.214751000 | 0.627224000 |
| C | –3.613606000 | –0.896300000 | 0.648283000 |
| C | –3.691828000 | –2.124985000 | –0.008824000 |
| C | –2.589756000 | –2.683159000 | –0.683527000 |
| C | –1.391854000 | –1.978887000 | –0.686389000 |
| C | 3.618562000 | –0.901215000 | –0.683527000 |
| C | 2.409694000 | –0.215938000 | –0.686389000 |
| C | 1.266854000 | –0.731408000 | –0.032962000 |
| C | 1.382020000 | –1.964228000 | 0.627224000 |
| C | 2.583022000 | –2.681324000 | 0.648283000 |
| C | 3.686205000 | –2.134724000 | –0.008824000 |
| C | 0.000000000 | 0.000000000 | –0.042633000 |
| C | –2.119143000 | 4.399289000 | –1.387281000 |
| C | 2.159910000 | 4.295044000 | 1.395304000 |
| C | –4.799572000 | –0.276985000 | 1.395304000 |
| C | –2.750325000 | –4.034877000 | –1.387281000 |
| C | 4.869468000 | –0.364413000 | –1.387281000 |
| C | 2.639662000 | –4.018059000 | 1.395304000 |
| C | –1.475235000 | 5.313119000 | –2.442285000 |
| C | –2.854681000 | 5.258284000 | –0.346081000 |
| C | –3.145861000 | 3.505031000 | –2.087344000 |
| C | 2.093571000 | 3.924900000 | 2.885610000 |
| C | 2.061200000 | 5.817406000 | 1.268770000 |
| C | 3.512946000 | 3.847402000 | 0.818119000 |
| C | –4.445848000 | –0.149364000 | 2.885610000 |
| C | –6.068622000 | –1.123651000 | 1.268770000 |
| C | –5.088421000 | 1.118599000 | 0.818119000 |
| C | –3.863679000 | –3.934151000 | –2.442285000 |
| C | –3.126467000 | –5.101368000 | –0.346081000 |
| C | –1.462515000 | –4.476911000 | –2.087344000 |
| C | 5.338914000 | –1.378968000 | –2.442285000 |
| C | 5.981148000 | –0.156916000 | –0.346081000 |
| C | 4.608377000 | 0.971880000 | –2.087344000 |
| C | 2.352278000 | –3.775535000 | 2.885610000 |
| C | 4.007422000 | –4.693755000 | 1.268770000 |
| C | 1.575475000 | –4.966001000 | 0.818119000 |
| H | –0.754738000 | 6.011048000 | –1.991083000 |
| H | –2.249263000 | 5.908718000 | –2.951315000 |
| H | –0.943066000 | 4.717378000 | –3.199047000 |
| H | –2.167960000 | 5.945610000 | 0.169554000 |
| H | –3.331725000 | 4.620473000 | 0.413913000 |
| H | –3.636888000 | 5.861168000 | –0.833540000 |
| H | –3.655082000 | 2.838078000 | –1.375527000 |
| H | –2.677014000 | 2.884832000 | –2.865722000 |
| H | –3.910826000 | 4.131809000 | –2.569544000 |
| H | 2.897920000 | 4.433054000 | 3.440434000 |
| H | 2.207400000 | 2.841574000 | 3.036227000 |
| H | 1.128042000 | 4.228020000 | 3.317761000 |
| H | 2.897433000 | 6.285669000 | 1.808776000 |
| H | 1.125647000 | 6.200296000 | 1.703303000 |
| H | 2.114201000 | 6.139748000 | 0.217957000 |
| H | 4.337706000 | 4.353333000 | 1.344277000 |
| H | 3.579984000 | 4.096951000 | –0.252069000 |
| H | 3.659401000 | 2.761840000 | 0.926010000 |
| H | –5.288097000 | 0.293145000 | 3.440434000 |
| H | –3.564575000 | 0.490877000 | 3.036227000 |
| H | –4.225594000 | –1.137097000 | 3.317761000 |
| H | –6.892266000 | –0.633584000 | 1.808776000 |
| H | –5.932437000 | –2.125309000 | 1.703303000 |
| H | –6.374278000 | –1.238922000 | 0.217957000 |
| H | –5.938950000 | 1.579897000 | 1.344277000 |
| H | –5.338056000 | 1.051882000 | –0.252069000 |
| H | –4.221524000 | 1.788214000 | 0.926010000 |

| | | | |
|---|---|---|---|
| H | –4.828352000 | –3.659147000 | –1.991083000 |
| H | –3.992469000 | –4.902278000 | –2.951315000 |
| H | –3.613836000 | –3.175408000 | –3.199047000 |
| H | –4.065070000 | –4.850314000 | 0.169554000 |
| H | –2.335585000 | –5.195595000 | 0.413913000 |
| H | –3.257476000 | –6.080221000 | –0.833540000 |
| H | –0.630307000 | –4.584433000 | –1.375527000 |
| H | –1.159831000 | –3.760778000 | –2.865722000 |
| H | –1.622839000 | –5.452779000 | –2.569544000 |
| H | 5.583090000 | –2.351902000 | –1.991083000 |
| H | 6.241731000 | –1.006441000 | –2.951315000 |
| H | 4.556902000 | –1.541970000 | –3.199047000 |
| H | 6.233030000 | –1.095297000 | 0.169554000 |
| H | 5.667310000 | 0.575122000 | 0.413913000 |
| H | 6.894364000 | 0.219054000 | –0.833540000 |
| H | 4.285389000 | 1.746355000 | –1.375527000 |
| H | 3.836845000 | 0.875946000 | –2.865722000 |
| H | 5.533665000 | 1.320970000 | –2.569544000 |
| H | 2.390178000 | –4.726199000 | 3.440434000 |
| H | 1.357175000 | –3.332451000 | 3.036227000 |
| H | 3.097552000 | –3.090923000 | 3.317761000 |
| H | 3.994832000 | –5.652085000 | 1.808776000 |
| H | 4.806791000 | –4.074987000 | 1.703303000 |
| H | 4.260077000 | –4.900826000 | 0.217957000 |
| H | 1.601244000 | –5.933231000 | 1.344277000 |
| H | 1.758072000 | –5.148833000 | –0.252069000 |
| H | 0.562124000 | –4.550054000 | 0.926010000 |
| H | 0.000000000 | 5.349250000 | –0.005317000 |
| H | –1.789586000 | 1.635360000 | –1.212961000 |
| H | 1.783596000 | 1.615275000 | 1.151474000 |
| H | –2.290668000 | 0.737002000 | 1.151474000 |
| H | –4.632586000 | –2.674625000 | –0.005317000 |
| H | –0.521470000 | –2.367507000 | –1.212961000 |
| H | 2.311056000 | 0.732147000 | –1.212961000 |
| H | 0.507071000 | –2.352277000 | 1.151474000 |
| H | 4.632586000 | –2.674625000 | –0.005317000 |

Cy-**1•**

B3LYP

| | | | |
|---|---|---|---|
| C | –1.452997000 | –0.200778000 | –0.085085000 |
| C | 0.000000000 | 0.000000000 | –0.095215000 |
| C | 0.552619000 | 1.358722000 | –0.085085000 |
| C | 1.741941000 | 1.663088000 | 0.619271000 |
| C | 2.269613000 | 2.956782000 | 0.658435000 |
| C | 3.529727000 | 3.243715000 | 1.461154000 |
| C | 5.963210000 | 4.004090000 | 1.414161000 |
| C | 5.699556000 | 4.958895000 | 2.586273000 |
| C | 4.542851000 | 4.459278000 | 3.462532000 |
| C | 4.693741000 | 3.759725000 | 0.586119000 |
| C | 3.273018000 | 4.214018000 | 2.635905000 |
| C | 1.600228000 | 3.976939000 | –0.038656000 |
| C | 0.420763000 | 3.721839000 | –0.758775000 |
| C | –0.300395000 | 4.830662000 | –1.510795000 |
| C | 0.565265000 | 5.466433000 | –2.620791000 |
| C | –0.194147000 | 6.562829000 | –3.381467000 |
| C | –0.737285000 | 7.638641000 | –2.431328000 |
| C | –1.601769000 | 7.018852000 | –1.325425000 |
| C | –0.844153000 | 5.923209000 | –0.562732000 |
| C | –0.088494000 | 2.420059000 | –0.767770000 |
| C | 0.900378000 | –1.157943000 | –0.085085000 |
| C | 2.140080000 | –1.133391000 | –0.767770000 |
| C | 3.012826000 | –2.225311000 | –0.758775000 |
| C | 4.333673000 | –2.155181000 | –1.510795000 |
| C | 6.879388000 | –2.122253000 | –1.325425000 |
| C | 6.983900000 | –3.180813000 | –2.431328000 |
| C | 5.780650000 | –3.113278000 | –3.381467000 |
| C | 5.551725000 | –2.230547000 | –0.562732000 |
| C | 4.451438000 | –3.222750000 | –2.620791000 |
| C | 2.644016000 | –3.374308000 | –0.038656000 |
| C | 1.425842000 | –3.443934000 | 0.658435000 |
| C | 1.044276000 | –4.678691000 | 1.461154000 |
| C | 2.012937000 | –4.941525000 | 2.635905000 |
| C | 1.590422000 | –6.163863000 | 3.462532000 |
| C | 0.486039000 | –7.166336000 | 1.414161000 |
| C | 1.444751000 | –7.415408000 | 2.586273000 |
| C | 0.909147000 | –5.944762000 | 0.586119000 |
| C | 0.569306000 | –2.340109000 | 0.619271000 |
| C | –2.051586000 | –1.286668000 | –0.767770000 |
| C | –3.433589000 | –1.496528000 | –0.758775000 |
| C | –4.033278000 | –2.675481000 | –1.510795000 |
| C | –5.277619000 | –4.896598000 | –1.325425000 |
| C | –6.246614000 | –4.457828000 | –2.431328000 |
| C | –5.586503000 | –3.449551000 | –3.381467000 |
| C | –4.707573000 | –3.692662000 | –0.562732000 |
| C | –5.016703000 | –2.243683000 | –2.620791000 |
| C | –4.244245000 | –0.602631000 | –0.038656000 |
| C | –3.695455000 | 0.487151000 | 0.658435000 |
| C | –4.574003000 | 1.434976000 | 1.461154000 |
| C | –6.449249000 | 3.162246000 | 1.414161000 |
| C | –7.144307000 | 2.456513000 | 2.586273000 |
| C | –6.133273000 | 1.704586000 | 3.462532000 |
| C | –5.602888000 | 2.185037000 | 0.586119000 |
| C | –5.285955000 | 0.727508000 | 2.635905000 |
| C | –2.311247000 | 0.677021000 | 0.619271000 |
| H | 2.245435000 | 0.868168000 | 1.173001000 |
| H | 3.856285000 | 2.283312000 | 1.900768000 |
| H | 6.330882000 | 3.037861000 | 1.808652000 |
| H | 6.764909000 | 4.400841000 | 0.768355000 |
| H | 6.613342000 | 5.083473000 | 3.191803000 |
| H | 5.449038000 | 5.961389000 | 2.189703000 |
| H | 4.842721000 | 3.514899000 | 3.955057000 |
| H | 4.334420000 | 5.179439000 | 4.271922000 |
| H | 4.391223000 | 4.704295000 | 0.097270000 |
| H | 4.894843000 | 3.040281000 | –0.225294000 |
| H | 2.904646000 | 5.177493000 | 2.237215000 |
| H | 2.468087000 | 3.814902000 | 3.275440000 |
| H | 2.004127000 | 4.991853000 | –0.016544000 |
| H | –1.173908000 | 4.368311000 | –2.006287000 |
| H | 0.908393000 | 4.682252000 | –3.316254000 |
| H | 1.475901000 | 5.901570000 | –2.169181000 |
| H | –1.038116000 | 6.105376000 | –3.931611000 |
| H | 0.462187000 | 7.017900000 | –4.142593000 |
| H | 0.111334000 | 8.178799000 | –1.969897000 |
| H | –1.315769000 | 8.390623000 | –2.994370000 |
| H | –1.945797000 | 7.798189000 | –0.624326000 |
| H | –2.512750000 | 6.583224000 | –1.777881000 |
| H | 0.000000000 | 6.378653000 | –0.012608000 |
| H | –1.496340000 | 5.461334000 | 0.197353000 |
| H | –0.997307000 | 2.209893000 | –1.335415000 |
| H | 2.412477000 | –0.241254000 | –1.335415000 |
| H | 4.370022000 | –1.167521000 | –2.006287000 |
| H | 7.726329000 | –2.213985000 | –0.624326000 |
| H | 6.957614000 | –1.115507000 | –1.777881000 |
| H | 7.027380000 | –4.185818000 | –1.969897000 |
| H | 7.924377000 | –3.055822000 | –2.994370000 |
| H | 5.806469000 | –2.153654000 | –3.931611000 |
| H | 5.846586000 | –3.909216000 | –4.142593000 |
| H | 5.524076000 | –3.189327000 | –0.012608000 |
| H | 5.477824000 | –1.434799000 | 0.197353000 |
| H | 3.600753000 | –3.127817000 | –3.316254000 |
| H | 4.372959000 | –4.228953000 | –2.169181000 |
| H | 3.321008000 | –4.231551000 | –0.016544000 |
| H | 0.049264000 | –4.481297000 | 1.900768000 |
| H | 3.031517000 | –5.104244000 | 2.237215000 |
| H | 2.069759000 | –4.044877000 | 3.275440000 |
| H | 0.622631000 | –5.951369000 | 3.955057000 |
| H | 2.318316000 | –6.343438000 | 4.271922000 |
| H | –0.534577000 | –7.001635000 | 1.808652000 |
| H | 0.428786000 | –8.059003000 | 0.768355000 |
| H | 1.095746000 | –8.269059000 | 3.191803000 |
| H | 2.438195000 | –7.699700000 | 2.189703000 |
| H | 1.878427000 | –6.155058000 | 0.097270000 |
| H | 0.185540000 | –5.759199000 | –0.225294000 |
| H | –0.370862000 | –2.378688000 | 1.173001000 |
| H | –1.415170000 | –1.968640000 | –1.335415000 |
| H | –3.196114000 | –3.200790000 | –2.006287000 |
| H | –5.780532000 | –5.584205000 | –0.624326000 |
| H | –4.444864000 | –5.467717000 | –1.777881000 |
| H | –7.138715000 | –3.992981000 | –1.969897000 |
| H | –6.608608000 | –5.334801000 | –2.994370000 |
| H | –4.768353000 | –3.951723000 | –3.931611000 |
| H | –6.308773000 | –3.108684000 | –4.142593000 |
| H | –5.524076000 | –3.189327000 | –0.012608000 |
| H | –3.981484000 | –4.026535000 | 0.197353000 |
| H | –4.509146000 | –1.554435000 | –3.316254000 |
| H | –5.848860000 | –1.672617000 | –2.169181000 |
| H | –5.325135000 | –0.760302000 | –0.016544000 |
| H | –3.905549000 | 2.197984000 | 1.900768000 |
| H | –5.796306000 | 3.963775000 | 1.808652000 |
| H | –7.193694000 | 3.658162000 | 0.768355000 |
| H | –7.709088000 | 3.185586000 | 3.191803000 |
| H | –7.887233000 | 1.738311000 | 2.189703000 |
| H | –5.465352000 | 2.436470000 | 3.955057000 |
| H | –6.652736000 | 1.163999000 | 4.271922000 |
| H | –6.269650000 | 1.450763000 | 0.097270000 |
| H | –5.080382000 | 2.718917000 | –0.225294000 |
| H | –5.936164000 | –0.073249000 | 2.237215000 |
| H | –4.537846000 | 0.229975000 | 3.275440000 |
| H | –1.874573000 | 1.510520000 | 1.173001000 |

B3LYP-D3(BJ)

| | | | |
|---|---|---|---|
| C | –1.261627000 | –0.736230000 | 0.085598000 |
| C | 0.000000000 | 0.000000000 | 0.094110000 |

| | | | |
|---|---|---|---|
| C | –0.006780000 | 1.460716000 | 0.085598000 |
| C | 1.007123000 | 2.203234000 | 0.732740000 |
| C | 1.022571000 | 3.598553000 | 0.716334000 |
| C | 2.152060000 | 4.352300000 | 1.389796000 |
| C | 4.260956000 | 5.729217000 | 1.019361000 |
| C | 3.770178000 | 6.763862000 | 2.038529000 |
| C | 2.841334000 | 6.122057000 | 3.076001000 |
| C | 3.088722000 | 5.003611000 | 0.351308000 |
| C | 1.665696000 | 5.403397000 | 2.404886000 |
| C | –0.003779000 | 4.277347000 | 0.041523000 |
| C | –1.034252000 | 3.579930000 | –0.608366000 |
| C | –2.145715000 | 4.317437000 | –1.327621000 |
| C | –1.623222000 | 5.226719000 | –2.456323000 |
| C | –2.768811000 | 5.939040000 | –3.183595000 |
| C | –3.641831000 | 6.735186000 | –2.206736000 |
| C | –4.168508000 | 5.840057000 | –1.079093000 |
| C | –3.024422000 | 5.125585000 | –0.351870000 |
| C | –1.022760000 | 2.185177000 | –0.579759000 |
| C | 1.268407000 | –0.724486000 | 0.085598000 |
| C | 2.403799000 | –0.206852000 | –0.579759000 |
| C | 3.617437000 | –0.894277000 | –0.608366000 |
| C | 4.811867000 | –0.300475000 | –1.327621000 |
| C | 7.141892000 | 0.690005000 | –1.079093000 |
| C | 7.653757000 | –0.213675000 | –2.206736000 |
| C | 6.527765000 | –0.571660000 | –3.183595000 |
| C | 5.951098000 | 0.056434000 | –0.351870000 |
| C | 5.338082000 | –1.207608000 | –2.456323000 |
| C | 3.706180000 | –2.135401000 | 0.041523000 |
| C | 2.605153000 | –2.684848000 | 0.716334000 |
| C | 2.693172000 | –4.039888000 | 1.389796000 |
| C | 3.846631000 | –4.144233000 | 2.404886000 |
| C | 3.881190000 | –5.521696000 | 3.076001000 |
| C | 2.831170000 | –6.554704000 | 1.019361000 |
| C | 3.972588000 | –6.647001000 | 2.038529000 |
| C | 2.788894000 | –5.176717000 | 0.351308000 |
| C | 1.404495000 | –1.973811000 | 0.732740000 |
| C | –1.381039000 | –1.978325000 | –0.579759000 |
| C | –2.583185000 | –2.685653000 | –0.608366000 |
| C | –2.666152000 | –4.016962000 | –1.327621000 |
| C | –2.973384000 | –6.530062000 | –1.079093000 |
| C | –4.011926000 | –6.521511000 | –2.206736000 |
| C | –3.758955000 | –5.367381000 | –3.183595000 |
| C | –2.926676000 | –5.182019000 | –0.351870000 |
| C | –3.714860000 | –4.019111000 | –2.456323000 |
| C | –3.702402000 | –2.141946000 | 0.041523000 |
| C | –3.627723000 | –0.913704000 | 0.716334000 |
| C | –4.845232000 | –0.312411000 | 1.389796000 |
| C | –7.092126000 | 0.825487000 | 1.019361000 |
| C | –7.742766000 | –0.116861000 | 2.038529000 |
| C | –6.722524000 | –0.600361000 | 3.076001000 |
| C | –5.877615000 | 0.173106000 | 0.351308000 |
| C | –5.512327000 | –1.259164000 | 2.404886000 |
| C | –2.411619000 | –0.229423000 | 0.732740000 |
| H | 1.790679000 | 1.669360000 | 1.272556000 |
| H | 2.751225000 | 3.610817000 | 1.948617000 |
| H | 4.898832000 | 4.987207000 | 1.534926000 |
| H | 4.894669000 | 6.210679000 | 0.256025000 |
| H | 4.626053000 | 7.248775000 | 2.536871000 |
| H | 3.220726000 | 7.563261000 | 1.506594000 |
| H | 3.417028000 | 5.393234000 | 3.676565000 |
| H | 2.468300000 | 6.883349000 | 3.781123000 |
| H | 2.505286000 | 5.721450000 | –0.254048000 |
| H | 3.454911000 | 4.232714000 | –0.346628000 |
| H | 1.036276000 | 6.148936000 | 1.886364000 |
| H | 1.022390000 | 4.919834000 | 3.158018000 |
| H | 0.000000000 | 5.368689000 | 0.020224000 |
| H | –2.794140000 | 3.554517000 | –1.795243000 |
| H | –1.024157000 | 4.629672000 | –3.163125000 |
| H | –0.940261000 | 5.981718000 | –2.026369000 |
| H | –3.394741000 | 5.186106000 | –3.697934000 |
| H | –2.366916000 | 6.601274000 | –3.968331000 |
| H | –3.040451000 | 7.553852000 | –1.768420000 |
| H | –4.479811000 | 7.212476000 | –2.741452000 |
| H | –4.762770000 | 6.431880000 | –0.363106000 |
| H | –4.853482000 | 5.084213000 | –1.506784000 |
| H | –2.394156000 | 5.872695000 | 0.164269000 |
| H | –3.417657000 | 4.455211000 | 0.429945000 |
| H | –1.805891000 | 1.636646000 | –1.105156000 |
| H | 2.320322000 | 0.745625000 | –1.105156000 |
| H | 4.475373000 | 0.642538000 | –1.795243000 |
| H | 7.951556000 | 0.908740000 | –0.363106000 |
| H | 6.829799000 | 1.661132000 | –1.506784000 |
| H | 8.062053000 | –1.143818000 | –1.768420000 |
| H | 8.486093000 | 0.273392000 | –2.741452000 |
| H | 6.188670000 | 0.346879000 | –3.697934000 |
| H | 6.900329000 | –1.250827000 | –3.968331000 |
| H | 6.282981000 | –0.862948000 | 0.164269000 |
| H | 5.567155000 | 0.732172000 | 0.429945000 |
| H | 4.521492000 | –1.427890000 | –3.163125000 |
| H | 5.650450000 | –2.176570000 | –2.026369000 |
| H | 4.649421000 | –2.684344000 | 0.020224000 |
| H | 1.751447000 | –4.188039000 | 1.948617000 |
| H | 4.806997000 | –3.971910000 | 1.886364000 |
| H | 3.749507000 | –3.345333000 | 3.158018000 |
| H | 2.962164000 | –5.655850000 | 3.676565000 |
| H | 4.727005000 | –5.579285000 | 3.781123000 |
| H | 1.869632000 | –6.736116000 | 1.534926000 |
| H | 2.931272000 | –7.344247000 | 0.256025000 |
| H | 3.964597000 | –7.630667000 | 2.536871000 |
| H | 4.939613000 | –6.570861000 | 1.506594000 |
| H | 3.702278000 | –5.030366000 | –0.254048000 |
| H | 1.938182000 | –5.108398000 | –0.346628000 |
| H | 0.550369000 | –2.385454000 | 1.272556000 |
| H | –0.514431000 | –2.382270000 | –1.105156000 |
| H | –1.681232000 | –4.197055000 | –1.795243000 |
| H | –3.188786000 | –7.340619000 | –0.363106000 |
| H | –1.976317000 | –6.745345000 | –1.506784000 |
| H | –5.021602000 | –6.410034000 | –1.768420000 |
| H | –4.006283000 | –7.485868000 | –2.741452000 |
| H | –2.793929000 | –5.532985000 | –3.697934000 |
| H | –4.533412000 | –5.350447000 | –3.968331000 |
| H | –3.888826000 | –5.009747000 | 0.164269000 |
| H | –2.149498000 | –5.187384000 | 0.429945000 |
| H | –3.497335000 | –3.201782000 | –3.163125000 |
| H | –4.710190000 | –3.805149000 | –2.026369000 |
| H | –4.649421000 | –2.684344000 | 0.020224000 |
| H | –4.502672000 | 0.577222000 | 1.948617000 |
| H | –6.768463000 | 1.748909000 | 1.534926000 |
| H | –7.825940000 | 1.133568000 | 0.256025000 |
| H | –8.590649000 | 0.381892000 | 2.536871000 |
| H | –8.160339000 | –0.992400000 | 1.506594000 |
| H | –6.379192000 | 0.262616000 | 3.676565000 |
| H | –7.195306000 | –1.304064000 | 3.781123000 |
| H | –6.207564000 | –0.691084000 | –0.254048000 |
| H | –5.393094000 | 0.875684000 | –0.346628000 |
| H | –5.843273000 | –2.177026000 | 1.886364000 |
| H | –4.771897000 | –1.574501000 | 3.158018000 |
| H | –2.341048000 | 0.716093000 | 1.272556000 |

M06-2X

| | | | |
|---|---|---|---|
| C | –0.934731000 | –1.124864000 | –0.041411000 |
| C | 0.000000000 | 0.000000000 | –0.041224000 |
| C | –0.506796000 | 1.371933000 | –0.041411000 |
| C | 0.181583000 | 2.404656000 | 0.627507000 |
| C | –0.296959000 | 3.712906000 | 0.641596000 |
| C | 0.444902000 | 4.795596000 | 1.397010000 |
| C | 1.647632000 | 7.022086000 | 1.254627000 |
| C | 0.808364000 | 7.556675000 | 2.415054000 |
| C | 0.371047000 | 6.421704000 | 3.340891000 |
| C | 0.892075000 | 5.942933000 | 0.478399000 |
| C | –0.386230000 | 5.341643000 | 2.568409000 |
| C | –1.487509000 | 4.001994000 | –0.036591000 |
| C | –2.198229000 | 3.006713000 | –0.718215000 |
| C | –3.486381000 | 3.332278000 | –1.443493000 |
| C | –3.288559000 | 4.379731000 | –2.549316000 |
| C | –4.597852000 | 4.672979000 | –3.282329000 |
| C | –5.687988000 | 5.121893000 | –2.308543000 |
| C | –5.895401000 | 4.083764000 | –1.205768000 |
| C | –4.587088000 | 3.788170000 | –0.472382000 |
| C | –1.702604000 | 1.704686000 | –0.709252000 |
| C | 1.441526000 | –0.247068000 | –0.041411000 |
| C | 2.327603000 | 0.622155000 | –0.709252000 |
| C | 3.703004000 | 0.400366000 | –0.718215000 |
| C | 4.629028000 | 1.353156000 | –1.443493000 |
| C | 6.484344000 | 3.063685000 | –1.205768000 |
| C | 7.279684000 | 2.364996000 | –2.308543000 |
| C | 6.345845000 | 1.645367000 | –3.282329000 |
| C | 5.574196000 | 2.078450000 | –0.472382000 |
| C | 5.437238000 | 0.658110000 | –2.549316000 |
| C | 4.209583000 | –0.712777000 | –0.036591000 |
| C | 3.363950000 | –1.599279000 | 0.641596000 |
| C | 3.930656000 | –2.783094000 | 1.397010000 |
| C | 4.819113000 | –2.336337000 | 2.568409000 |
| C | 5.375835000 | –3.532188000 | 3.340891000 |
| C | 5.257489000 | –4.937934000 | 1.254627000 |
| C | 6.140090000 | –4.478401000 | 2.415054000 |
| C | 4.700693000 | –3.744026000 | 0.478399000 |
| C | 1.991701000 | –1.359584000 | 0.627507000 |
| C | –0.624999000 | –2.326841000 | –0.709252000 |
| C | –1.504775000 | –3.407078000 | –0.718215000 |
| C | –1.142647000 | –4.685434000 | –1.443493000 |
| C | –0.588943000 | –7.147449000 | –1.205768000 |
| C | –1.591696000 | –7.486889000 | –2.308543000 |
| C | –1.747993000 | –6.318346000 | –3.282329000 |
| C | –0.987107000 | –5.866620000 | –0.472382000 |

| | | | |
|---|---|---|---|
| C | –2.148678000 | –5.037841000 | –2.549316000 |
| C | –2.722074000 | –3.289217000 | –0.036591000 |
| C | –3.066991000 | –2.113627000 | 0.641596000 |
| C | –4.375559000 | –2.012501000 | 1.397010000 |
| C | –6.905121000 | –2.084152000 | 1.254627000 |
| C | –6.948455000 | –3.078274000 | 2.415054000 |
| C | –5.746882000 | –2.889515000 | 3.340891000 |
| C | –5.592768000 | –2.198907000 | 0.478399000 |
| C | –4.432884000 | –3.005306000 | 2.568409000 |
| C | –2.173285000 | –1.045072000 | 0.627507000 |
| H | 1.099511000 | 2.166037000 | 1.168907000 |
| H | 1.355974000 | 4.338195000 | 1.822464000 |
| H | 2.582744000 | 6.591574000 | 1.654738000 |
| H | 1.939264000 | 7.840732000 | 0.579192000 |
| H | 1.370947000 | 8.317293000 | 2.977509000 |
| H | –0.088836000 | 8.058753000 | 2.010512000 |
| H | 1.265852000 | 5.972563000 | 3.807435000 |
| H | –0.252034000 | 6.810339000 | 4.160755000 |
| H | 0.000000000 | 6.392912000 | 0.006949000 |
| H | 1.511844000 | 5.540992000 | –0.338302000 |
| H | –1.324577000 | 5.768472000 | 2.170909000 |
| H | –0.673169000 | 4.512645000 | 3.233700000 |
| H | –1.872790000 | 5.025208000 | –0.029665000 |
| H | –3.837142000 | 2.402998000 | –1.926574000 |
| H | –2.515674000 | 4.033378000 | –3.252658000 |
| H | –2.910073000 | 5.313147000 | –2.095761000 |
| H | –4.932998000 | 3.757433000 | –3.801263000 |
| H | –4.435230000 | 5.435849000 | –4.058865000 |
| H | –5.388973000 | 6.081473000 | –1.849743000 |
| H | –6.630569000 | 5.306594000 | –2.845965000 |
| H | –6.661468000 | 4.424810000 | –0.492914000 |
| H | –6.274324000 | 3.149785000 | –1.657206000 |
| H | –4.246541000 | 4.700912000 | 0.049090000 |
| H | –4.738128000 | 3.019445000 | 0.301636000 |
| H | –2.242341000 | 0.924001000 | –1.249576000 |
| H | 1.921379000 | 1.479924000 | –1.249576000 |
| H | 3.999629000 | 2.121563000 | –1.926574000 |
| H | 7.162731000 | 3.556596000 | –0.492914000 |
| H | 5.864956000 | 3.858832000 | –1.657206000 |
| H | 7.961197000 | 1.626251000 | –1.849743000 |
| H | 7.910929000 | 3.088944000 | –2.845965000 |
| H | 5.720531000 | 2.393385000 | –3.801263000 |
| H | 6.925198000 | 1.123097000 | –4.058865000 |
| H | 6.194380000 | 1.327156000 | 0.049090000 |
| H | 4.983980000 | 2.593617000 | 0.301636000 |
| H | 4.750845000 | 0.161948000 | –3.252658000 |
| H | 6.056357000 | –0.136376000 | –2.095761000 |
| H | 5.288353000 | –0.890720000 | –0.029665000 |
| H | 3.079000000 | –3.343405000 | 1.822464000 |
| H | 5.657932000 | –1.737118000 | 2.170909000 |
| H | 4.244649000 | –1.673341000 | 3.233700000 |
| H | 4.539466000 | –4.082542000 | 3.807435000 |
| H | 6.023944000 | –3.186901000 | 4.160755000 |
| H | 4.417098000 | –5.532509000 | 1.654738000 |
| H | 5.820641000 | –5.599818000 | 0.579192000 |
| H | 6.517513000 | –5.345922000 | 2.977509000 |
| H | 7.023503000 | –3.952443000 | 2.010512000 |
| H | 5.536424000 | –3.196456000 | 0.006949000 |
| H | 4.042718000 | –4.079791000 | –0.338302000 |
| H | 1.326088000 | –2.035223000 | 1.168907000 |
| H | 0.320963000 | –2.403925000 | –1.249576000 |
| H | –0.162487000 | –4.524562000 | –1.926574000 |
| H | –0.501264000 | –7.981405000 | –0.492914000 |
| H | 0.409368000 | –7.008617000 | –1.657206000 |
| H | –2.572224000 | –7.707724000 | –1.849743000 |
| H | –1.280361000 | –8.395538000 | –2.845965000 |
| H | –0.787533000 | –6.150818000 | –3.801263000 |
| H | –2.489968000 | –6.558946000 | –4.058865000 |
| H | –1.947839000 | –6.028069000 | 0.049090000 |
| H | –0.245852000 | –5.613062000 | 0.301636000 |
| H | –2.235171000 | –4.195327000 | –3.252658000 |
| H | –3.146284000 | –5.176771000 | –2.095761000 |
| H | –3.415562000 | –4.134488000 | –0.029665000 |
| H | –4.434974000 | –0.994789000 | 1.822464000 |
| H | –6.999842000 | –1.059065000 | 1.654738000 |
| H | –7.759905000 | –2.240914000 | 0.579192000 |
| H | –7.888460000 | –2.971371000 | 2.977509000 |
| H | –6.934667000 | –4.106311000 | 2.010512000 |
| H | –5.805318000 | –1.890021000 | 3.807435000 |
| H | –5.771909000 | –3.623437000 | 4.160755000 |
| H | –5.536424000 | –3.196456000 | 0.006949000 |
| H | –5.554562000 | –1.461200000 | –0.338302000 |
| H | –4.333355000 | –4.031354000 | 2.170909000 |
| H | –3.571481000 | –2.839304000 | 3.233700000 |
| H | –2.425599000 | –0.130814000 | 1.168907000 |

Ad-**1**•

B3LYP

| | | | |
|---|---|---|---|
| C | 0.000000000 | 0.000000000 | 0.006580000 |
| C | 1.959594000 | 1.404301000 | –0.690910000 |
| C | 2.669879000 | 2.608915000 | –0.712020000 |
| C | 0.734613000 | 1.270856000 | 0.005270000 |
| C | 2.135892000 | 3.698510000 | –0.000460000 |
| C | 0.238360000 | 2.400717000 | 0.698510000 |
| C | 0.926263000 | 3.618764000 | 0.712910000 |
| C | –0.944141000 | 4.595710000 | 2.182330000 |
| C | 0.416564000 | 4.846036000 | 1.485270000 |
| C | 1.441741000 | 5.250960000 | 2.584600000 |
| C | 0.235502000 | 6.054392000 | 0.520970000 |
| C | –1.419843000 | 5.848558000 | 2.944230000 |
| C | –0.383117000 | 6.220991000 | 4.021510000 |
| C | –1.584196000 | 7.023718000 | 1.962540000 |
| C | 0.970995000 | 6.506373000 | 3.345560000 |
| C | –0.231629000 | 7.309565000 | 1.284280000 |
| C | 0.813716000 | 7.678026000 | 2.356230000 |
| C | –6.214455000 | –3.855379000 | 1.284280000 |
| C | –5.361008000 | –2.823246000 | 0.520970000 |
| C | –5.290620000 | –4.883814000 | 1.962540000 |
| C | –7.056223000 | –3.134315000 | 2.356230000 |
| C | –4.270950000 | 0.000482000 | –0.000460000 |
| C | –4.405072000 | –2.062263000 | 1.485270000 |
| C | –3.594326000 | 1.007725000 | –0.712020000 |
| C | –4.355078000 | –4.153899000 | 2.944230000 |
| C | –3.597073000 | –1.007214000 | 0.712910000 |
| C | –3.507931000 | –3.115505000 | 2.182330000 |
| C | –2.195958000 | 0.994908000 | –0.690910000 |
| C | –2.198262000 | –0.993932000 | 0.698510000 |
| C | –1.467900000 | 0.000766000 | 0.005270000 |
| C | –6.120182000 | –2.412280000 | 3.345560000 |
| C | –5.268335000 | –1.376896000 | 2.584600000 |
| C | –5.195978000 | –3.442284000 | 4.021510000 |
| C | 5.149187000 | –4.094093000 | 3.345560000 |
| C | 3.826595000 | –3.874064000 | 2.584600000 |
| C | 6.242508000 | –4.543711000 | 2.356230000 |
| C | 5.579095000 | –2.778707000 | 4.021510000 |
| C | 2.135058000 | –3.698992000 | –0.000460000 |
| C | 3.988509000 | –2.783773000 | 1.485270000 |
| C | 0.924447000 | –3.616641000 | –0.712020000 |
| C | 6.446084000 | –3.454186000 | 1.284280000 |
| C | 2.670810000 | –2.611550000 | 0.712910000 |
| C | 5.125506000 | –3.231146000 | 0.520970000 |
| C | 0.236364000 | –2.399209000 | –0.690910000 |
| C | 1.959901000 | –1.406785000 | 0.698510000 |
| C | 0.733287000 | –1.271621000 | 0.005270000 |
| C | 5.774921000 | –1.694659000 | 2.944230000 |
| C | 4.452072000 | –1.480205000 | 2.182330000 |
| C | 6.874817000 | –2.139905000 | 1.962540000 |
| C | –0.383117000 | –6.215701000 | –4.024670000 |
| C | 0.965890000 | –6.507588000 | –3.341500000 |
| C | 1.439588000 | –5.253277000 | –2.580560000 |
| C | –1.422559000 | –5.835984000 | –2.952720000 |
| C | –0.944141000 | –4.585138000 | –2.189370000 |
| C | 0.412254000 | –4.841856000 | –1.485650000 |
| C | 0.797631000 | –7.676953000 | –2.351440000 |
| C | –1.598119000 | –7.008535000 | –1.969760000 |
| C | –0.250290000 | –7.301301000 | –1.284570000 |
| C | 0.220786000 | –6.047912000 | –0.520760000 |
| C | 5.127253000 | 3.215162000 | –0.520760000 |
| C | 3.987043000 | 2.777951000 | –1.485650000 |
| C | 3.829677000 | 3.873358000 | –2.580560000 |
| C | 6.448257000 | 3.433893000 | –1.284570000 |
| C | 4.442916000 | 1.474919000 | –2.189370000 |
| C | 6.249621000 | 4.529246000 | –2.351440000 |
| C | 5.152791000 | 4.090279000 | –3.341500000 |
| C | 6.868629000 | 2.120256000 | –1.969760000 |
| C | 5.765390000 | 1.686020000 | –2.952720000 |
| C | 5.574513000 | 2.776062000 | –4.024670000 |
| C | –3.498776000 | 3.110219000 | –2.189370000 |
| C | –4.399297000 | 2.063905000 | –1.485650000 |
| C | –5.348038000 | 2.832750000 | –0.520760000 |
| C | –4.342831000 | 4.149964000 | –2.952720000 |
| C | –5.269265000 | 1.379918000 | –2.580560000 |
| C | –5.270510000 | 4.888279000 | –1.969760000 |
| C | –6.197967000 | 3.867408000 | –1.284570000 |
| C | –5.191396000 | 3.439639000 | –4.024670000 |
| C | –6.118681000 | 2.417309000 | –3.341500000 |
| C | –7.047252000 | 3.147708000 | –2.351440000 |
| H | 2.328664000 | 0.539835000 | –1.238470000 |
| H | 2.682088000 | 4.642884000 | –0.000560000 |
| H | –0.693528000 | 2.290245000 | 1.248860000 |
| H | –0.855731000 | 3.751553000 | 2.886900000 |
| H | –1.701740000 | 4.307851000 | 1.433590000 |

| | | | |
|---|---|---|---|
| H | 2.427035000 | 5.442152000 | 2.127810000 |
| H | 1.577686000 | 4.407494000 | 3.283560000 |
| H | 1.184822000 | 6.269893000 | 0.003710000 |
| H | –0.496156000 | 5.787939000 | –0.261640000 |
| H | –2.388719000 | 5.627987000 | 3.423620000 |
| H | –0.722288000 | 7.107095000 | 4.586260000 |
| H | –0.278570000 | 5.397239000 | 4.749300000 |
| H | –2.346921000 | 6.780854000 | 1.201740000 |
| H | –1.940924000 | 7.920874000 | 2.498440000 |
| H | 1.721752000 | 6.766126000 | 4.111450000 |
| H | –0.341634000 | 8.144621000 | 0.571280000 |
| H | 0.501159000 | 8.590159000 | 2.894150000 |
| H | 1.783369000 | 7.905481000 | 1.879000000 |
| H | –6.882632000 | –4.368174000 | 0.571280000 |
| H | –6.022298000 | –2.108861000 | 0.003710000 |
| H | –4.764425000 | –3.323653000 | –0.261640000 |
| H | –4.698931000 | –5.422920000 | 1.201740000 |
| H | –5.889216000 | –5.641326000 | 2.498440000 |
| H | –7.689875000 | –3.861063000 | 2.894150000 |
| H | –7.738031000 | –2.408298000 | 1.879000000 |
| H | –5.361900000 | 0.001315000 | –0.000560000 |
| H | –3.679620000 | –4.882685000 | 3.423620000 |
| H | –2.821075000 | –2.616862000 | 2.886900000 |
| H | –2.879839000 | –3.627675000 | 1.433590000 |
| H | –1.631843000 | 1.746764000 | –1.238470000 |
| H | –1.636647000 | –1.745735000 | 1.248860000 |
| H | –6.720513000 | –1.891982000 | 4.111450000 |
| H | –5.926560000 | –0.619202000 | 2.127810000 |
| H | –4.605844000 | –0.837431000 | 3.283560000 |
| H | –5.793781000 | –4.179067000 | 4.586260000 |
| H | –4.534861000 | –2.939869000 | 4.749300000 |
| H | 4.998761000 | –4.874144000 | 4.111450000 |
| H | 3.499524000 | –4.822950000 | 2.127810000 |
| H | 3.028158000 | –3.570063000 | 3.283560000 |
| H | 7.188716000 | –4.729096000 | 2.894150000 |
| H | 5.954663000 | –5.497183000 | 1.879000000 |
| H | 6.516069000 | –2.928028000 | 4.586260000 |
| H | 4.813432000 | –2.457371000 | 4.749300000 |
| H | 2.679811000 | –4.644199000 | –0.000560000 |
| H | 7.224266000 | –3.776447000 | 0.571280000 |
| H | 4.837476000 | –4.161032000 | 0.003710000 |
| H | 5.260580000 | –2.464286000 | –0.261640000 |
| H | –0.696821000 | –2.286599000 | –1.238470000 |
| H | 2.330175000 | –0.544510000 | 1.248860000 |
| H | 6.068340000 | –0.745302000 | 3.423620000 |
| H | 3.676806000 | –1.134692000 | 2.886900000 |
| H | 4.581578000 | –0.680176000 | 1.433590000 |
| H | 7.045852000 | –1.357934000 | 1.201740000 |
| H | 7.830140000 | –2.279547000 | 2.498440000 |
| H | –0.724450000 | –7.100617000 | –4.590010000 |
| H | –0.270304000 | –5.393497000 | –4.753010000 |
| H | 1.719183000 | –6.772459000 | –4.103160000 |
| H | 1.581679000 | –4.411710000 | –3.280630000 |
| H | 2.422409000 | –5.448094000 | –2.120180000 |
| H | –2.388108000 | –5.610839000 | –3.436660000 |
| H | –1.704452000 | –4.294308000 | –1.444560000 |
| H | –0.848096000 | –3.741219000 | –2.893140000 |
| H | 1.764007000 | –7.909285000 | –1.869860000 |
| H | 0.482296000 | –8.588177000 | –2.889320000 |
| H | –1.957782000 | –7.904634000 | –2.505470000 |
| H | –2.362732000 | –6.759506000 | –1.212890000 |
| H | –0.368490000 | –8.135013000 | –0.571340000 |
| H | 1.166858000 | –6.267571000 | 0.000870000 |
| H | –0.512653000 | –5.775996000 | 0.258300000 |
| H | 4.844447000 | 4.144314000 | 0.000870000 |
| H | 5.258486000 | 2.444027000 | 0.258300000 |
| H | 3.029813000 | 3.575629000 | –3.280630000 |
| H | 3.506984000 | 4.821915000 | –2.120180000 |
| H | 7.229373000 | 3.748385000 | –0.571340000 |
| H | 4.571206000 | 0.671055000 | –1.444560000 |
| H | 3.664038000 | 1.136137000 | –2.893140000 |
| H | 5.967638000 | 5.482317000 | –1.869860000 |
| H | 7.196431000 | 4.711769000 | –2.889320000 |
| H | 5.005530000 | 4.875086000 | –4.103160000 |
| H | 7.824505000 | 2.256828000 | –2.505470000 |
| H | 7.035271000 | 1.333567000 | –1.212890000 |
| H | 6.053183000 | 0.737258000 | –3.436660000 |
| H | 6.511540000 | 2.922916000 | –4.590010000 |
| H | 4.806058000 | 2.462658000 | –4.753010000 |
| H | –2.866753000 | 3.623253000 | –1.444560000 |
| H | –2.815943000 | 2.605082000 | –2.893140000 |
| H | –6.011305000 | 2.123257000 | 0.000870000 |
| H | –4.745833000 | 3.331969000 | 0.258300000 |
| H | –3.665076000 | 4.873582000 | –3.436660000 |
| H | –4.611492000 | 0.836081000 | –3.280630000 |
| H | –5.929393000 | 0.626180000 | –2.120180000 |
| H | –5.866723000 | 5.647806000 | –2.505470000 |
| H | –4.672538000 | 5.425939000 | –1.212890000 |
| H | –6.860883000 | 4.386628000 | –0.571340000 |
| H | –5.787089000 | 4.177701000 | –4.590010000 |
| H | –4.535754000 | 2.930839000 | –4.753010000 |
| H | –6.724713000 | 1.897373000 | –4.103160000 |
| H | –7.731645000 | 2.426968000 | –1.869860000 |
| H | –7.678727000 | 3.876408000 | –2.889320000 |

B3LYP-D3(BJ)

| | | | |
|---|---|---|---|
| C | 0.000000000 | 0.000000000 | 0.081460000 |
| C | 1.054740000 | 2.180420000 | –0.554070000 |
| C | 1.080540000 | 3.575040000 | –0.570890000 |
| C | 0.010470000 | 1.461220000 | 0.070410000 |
| C | 0.000000000 | 4.264800000 | 0.004730000 |
| C | –1.030690000 | 2.201490000 | 0.674290000 |
| C | –1.067610000 | 3.595670000 | 0.626210000 |
| C | –3.257490000 | 3.499350000 | 1.944630000 |
| C | –2.243890000 | 4.391610000 | 1.192430000 |
| C | –1.754070000 | 5.486280000 | 2.175580000 |
| C | –2.999170000 | 5.082870000 | 0.025140000 |
| C | –4.438420000 | 4.328390000 | 2.476050000 |
| C | –3.920680000 | 5.405530000 | 3.443230000 |
| C | –5.162410000 | 5.000240000 | 1.297420000 |
| C | –2.934780000 | 6.321170000 | 2.699600000 |
| C | –4.179010000 | 5.917380000 | 0.551270000 |
| C | –3.651930000 | 6.994670000 | 1.515590000 |
| C | –3.035096000 | –6.577819000 | 0.551270000 |
| C | –2.902309000 | –5.138792000 | 0.025140000 |
| C | –1.749130000 | –6.970898000 | 1.297420000 |
| C | –4.231597000 | –6.659999000 | 1.515590000 |
| C | –3.693425000 | –2.132400000 | 0.004730000 |
| C | –2.681301000 | –4.139071000 | 1.192430000 |
| C | –3.636345000 | –0.851745000 | –0.570890000 |
| C | –1.529285000 | –6.007979000 | 2.476050000 |
| C | –2.580136000 | –2.722412000 | 0.626210000 |
| C | –1.401781000 | –4.570744000 | 1.944630000 |
| C | –2.415669000 | –0.176778000 | –0.554070000 |
| C | –1.391201000 | –1.993349000 | 0.674290000 |
| C | –1.270689000 | –0.721543000 | 0.070410000 |
| C | –4.006904000 | –5.702179000 | 2.699600000 |
| C | –3.874223000 | –4.262209000 | 2.175580000 |
| C | –2.720986000 | –6.098173000 | 3.443230000 |
| C | 6.941684000 | –0.618991000 | 2.699600000 |
| C | 5.628293000 | –1.224071000 | 2.175580000 |
| C | 7.883527000 | –0.334671000 | 1.515590000 |
| C | 6.641666000 | 0.692644000 | 3.443230000 |
| C | 3.693425000 | –2.132400000 | 0.004730000 |
| C | 4.925191000 | –0.252539000 | 1.192430000 |
| C | 2.555805000 | –2.723295000 | –0.570890000 |
| C | 7.214106000 | 0.660439000 | 0.551270000 |
| C | 3.647746000 | –0.873258000 | 0.626210000 |
| C | 5.901479000 | 0.055923000 | 0.025140000 |
| C | 1.360929000 | –2.003642000 | –0.554070000 |
| C | 2.421891000 | –0.208141000 | 0.674290000 |
| C | 1.260218000 | –0.739677000 | 0.070410000 |
| C | 5.967705000 | 1.679590000 | 2.476050000 |
| C | 4.659271000 | 1.071394000 | 1.944630000 |
| C | 6.911540000 | 1.970658000 | 1.297420000 |
| C | 2.461720000 | –6.050890000 | –3.442870000 |
| C | 3.820380000 | –5.590620000 | –2.887970000 |
| C | 3.689080000 | –4.167370000 | –2.319300000 |
| C | 1.422450000 | –6.043400000 | –2.309400000 |
| C | 1.297240000 | –4.620630000 | –1.738760000 |
| C | 2.647310000 | –4.126340000 | –1.171200000 |
| C | 4.269990000 | –6.548650000 | –1.770700000 |
| C | 1.867710000 | –7.007850000 | –1.197770000 |
| C | 3.224940000 | –6.549200000 | –0.640620000 |
| C | 3.089220000 | –5.127970000 | –0.070370000 |
| C | 2.896342000 | 5.239328000 | –0.070370000 |
| C | 2.249860000 | 4.355808000 | –1.171200000 |
| C | 1.764508000 | 5.278522000 | –2.319300000 |
| C | 4.059303000 | 6.067480000 | –0.640620000 |
| C | 3.352963000 | 3.433758000 | –1.738760000 |
| C | 3.536302000 | 6.972245000 | –1.770700000 |
| C | 2.931429000 | 6.103856000 | –2.887970000 |
| C | 5.135121000 | 5.121410000 | –1.197770000 |
| C | 4.522513000 | 4.253578000 | –2.309400000 |
| C | 4.009364000 | 5.157357000 | –3.442870000 |
| C | –4.650203000 | 1.186872000 | –1.738760000 |
| C | –4.897170000 | –0.229468000 | –1.171200000 |
| C | –5.985562000 | –0.111358000 | –0.070370000 |
| C | –5.944963000 | 1.789822000 | –2.309400000 |
| C | –5.453588000 | –1.111152000 | –2.319300000 |
| C | –7.002831000 | 1.886440000 | –1.197770000 |
| C | –7.284244000 | 0.481720000 | –0.640620000 |
| C | –6.471085000 | 0.893533000 | –3.442870000 |
| C | –6.751809000 | –0.513236000 | –2.887970000 |
| C | –7.806292000 | –0.423595000 | –1.770700000 |

| | | | |
|---|---|---|---|
| H | 1.844170000 | 1.614910000 | –1.040040000 |
| H | –0.008690000 | 5.354450000 | –0.030020000 |
| H | –1.813010000 | 1.652090000 | 1.189360000 |
| H | –2.753420000 | 2.986180000 | 2.780540000 |
| H | –3.639310000 | 2.715110000 | 1.270850000 |
| H | –1.031680000 | 6.148930000 | 1.673250000 |
| H | –1.218420000 | 5.007300000 | 3.012490000 |
| H | –2.302940000 | 5.726110000 | –0.537380000 |
| H | –3.357340000 | 4.312000000 | –0.678430000 |
| H | –5.136170000 | 3.658410000 | 3.005200000 |
| H | –4.763570000 | 5.995980000 | 3.841510000 |
| H | –3.420610000 | 4.931930000 | 4.305440000 |
| H | –5.558650000 | 4.233380000 | 0.610190000 |
| H | –6.024710000 | 5.583550000 | 1.663210000 |
| H | –2.553280000 | 7.093430000 | 3.388210000 |
| H | –4.690580000 | 6.397990000 | –0.299280000 |
| H | –4.486870000 | 7.615460000 | 1.883050000 |
| H | –2.956630000 | 7.668900000 | 0.986260000 |
| H | –3.195532000 | –7.261156000 | –0.299280000 |
| H | –3.807487000 | –4.857459000 | –0.537380000 |
| H | –2.055631000 | –5.063542000 | –0.678430000 |
| H | –0.886889000 | –6.930622000 | 0.610190000 |
| H | –1.823141000 | –8.009327000 | 1.663210000 |
| H | –4.351747000 | –7.693473000 | 1.883050000 |
| H | –5.163147000 | –6.394967000 | 0.986260000 |
| H | –4.632745000 | –2.684751000 | –0.030020000 |
| H | –0.600191000 | –6.277259000 | 3.005200000 |
| H | –1.209398000 | –3.877622000 | 2.780540000 |
| H | –0.531699000 | –4.509290000 | 1.270850000 |
| H | –2.320638000 | 0.789643000 | –1.040040000 |
| H | –0.524247000 | –2.396158000 | 1.189360000 |
| H | –4.866450000 | –5.757920000 | 3.388210000 |
| H | –4.809289000 | –3.967926000 | 1.673250000 |
| H | –3.727239000 | –3.558833000 | 3.012490000 |
| H | –2.810886000 | –7.123363000 | 3.841510000 |
| H | –2.560871000 | –5.428300000 | 4.305440000 |
| H | 7.419730000 | –1.335509000 | 3.388210000 |
| H | 5.840969000 | –2.181004000 | 1.673250000 |
| H | 4.945659000 | –1.448467000 | 3.012490000 |
| H | 8.838617000 | 0.078014000 | 1.883050000 |
| H | 8.119777000 | –1.273933000 | 0.986260000 |
| H | 7.574456000 | 1.127383000 | 3.841510000 |
| H | 5.981481000 | 0.496370000 | 4.305440000 |
| H | 4.641435000 | –2.669699000 | –0.030020000 |
| H | 7.886112000 | 0.863167000 | –0.299280000 |
| H | 6.110427000 | –0.868650000 | –0.537380000 |
| H | 5.412971000 | 0.751542000 | –0.678430000 |
| H | 0.476468000 | –2.404553000 | –1.040040000 |
| H | 2.337257000 | 0.744068000 | 1.189360000 |
| H | 5.736361000 | 2.618849000 | 3.005200000 |
| H | 3.962818000 | 0.891442000 | 2.780540000 |
| H | 4.171009000 | 1.794180000 | 1.270850000 |
| H | 6.445539000 | 2.697242000 | 0.610190000 |
| H | 7.847851000 | 2.425777000 | 1.663210000 |
| H | 2.549490000 | –7.063720000 | –3.872170000 |
| H | 2.139210000 | –5.380140000 | –4.257670000 |
| H | 4.569770000 | –5.588660000 | –3.697030000 |
| H | 3.382260000 | –3.462370000 | –3.110190000 |
| H | 4.668490000 | –3.823730000 | –1.949160000 |
| H | 0.441430000 | –6.358370000 | –2.702140000 |
| H | 0.531240000 | –4.606930000 | –0.945970000 |
| H | 0.950480000 | –3.935800000 | –2.530080000 |
| H | 5.252810000 | –6.236450000 | –1.377020000 |
| H | 4.392730000 | –7.568630000 | –2.173420000 |
| H | 1.943700000 | –8.035250000 | –1.593090000 |
| H | 1.115940000 | –7.026900000 | –0.390370000 |
| H | 3.547230000 | –7.232380000 | 0.162820000 |
| H | 4.049540000 | –4.807110000 | 0.363740000 |
| H | 2.349860000 | –5.111200000 | 0.748520000 |
| H | 2.138309000 | 5.910560000 | 0.363740000 |
| H | 3.251499000 | 4.590639000 | 0.748520000 |
| H | 1.307370000 | 4.660308000 | –3.110190000 |
| H | 0.977202000 | 5.954896000 | –1.949160000 |
| H | 4.489810000 | 6.688182000 | 0.162820000 |
| H | 3.724098000 | 2.763533000 | –0.945970000 |
| H | 2.933263000 | 2.791040000 | –2.530080000 |
| H | 2.774519000 | 7.667292000 | –1.377020000 |
| H | 4.358261000 | 7.588531000 | –2.173420000 |
| H | 2.555036000 | 6.751867000 | –3.697030000 |
| H | 5.986881000 | 5.700919000 | –1.593090000 |
| H | 5.527504000 | 4.479883000 | –0.390370000 |
| H | 5.285795000 | 3.561475000 | –2.702140000 |
| H | 4.842616000 | 5.739783000 | –3.872170000 |
| H | 3.589733000 | 4.542680000 | –4.257670000 |
| H | –4.255339000 | 1.843397000 | –0.945970000 |
| H | –3.883743000 | 1.144760000 | –2.530080000 |
| H | –6.187850000 | –1.103450000 | 0.363740000 |
| H | –5.601359000 | 0.520561000 | 0.748520000 |
| H | –5.727225000 | 2.796895000 | –2.702140000 |
| H | –4.689631000 | –1.197938000 | –3.110190000 |
| H | –5.645692000 | –2.131166000 | –1.949160000 |
| H | –7.930581000 | 2.334331000 | –1.593090000 |
| H | –6.643444000 | 2.547017000 | –0.390370000 |
| H | –8.037040000 | 0.544198000 | 0.162820000 |
| H | –7.392106000 | 1.323937000 | –3.872170000 |
| H | –5.728943000 | 0.837460000 | –4.257670000 |
| H | –7.124807000 | –1.163207000 | –3.697030000 |
| H | –8.027329000 | –1.430842000 | –1.377020000 |
| H | –8.750991000 | –0.019901000 | –2.173420000 |

M06-2X

| | | | |
|---|---|---|---|
| C | 0.000000000 | 0.000000000 | 0.220480000 |
| C | 1.045940000 | 2.160111000 | –0.477590000 |
| C | 1.057730000 | 3.552171000 | –0.546690000 |
| C | 0.022180000 | 1.463121000 | 0.194770000 |
| C | 0.011240000 | 4.253321000 | 0.068600000 |
| C | –0.998640000 | 2.210761000 | 0.813270000 |
| C | –1.025950000 | 3.603681000 | 0.751320000 |
| C | –3.150730000 | 3.545251000 | 2.155650000 |
| C | –2.163310000 | 4.420461000 | 1.360780000 |
| C | –1.613020000 | 5.506131000 | 2.313720000 |
| C | –2.953100000 | 5.118051000 | 0.228440000 |
| C | –4.291000000 | 4.390701000 | 2.737040000 |
| C | –3.715660000 | 5.460591000 | 3.673090000 |
| C | –5.055090000 | 5.066871000 | 1.591710000 |
| C | –2.754310000 | 6.358361000 | 2.884880000 |
| C | –4.096140000 | 5.966691000 | 0.801170000 |
| C | –3.514030000 | 7.036181000 | 1.735470000 |
| C | –3.119236000 | –6.530707000 | 0.801170000 |
| C | –2.955812000 | –5.116485000 | 0.228440000 |
| C | –1.860494000 | –6.911272000 | 1.591710000 |
| C | –4.336496000 | –6.561330000 | 1.735470000 |
| C | –3.689104000 | –2.116926000 | 0.068600000 |
| C | –2.746576000 | –4.083712000 | 1.360780000 |
| C | –3.605135000 | –0.860064000 | –0.546690000 |
| C | –1.656958000 | –5.911465000 | 2.737040000 |
| C | –2.607904000 | –2.690339000 | 0.751320000 |
| C | –1.494912000 | –4.501237000 | 2.155650000 |
| C | –2.393681000 | –0.174245000 | –0.477590000 |
| C | –1.415255000 | –1.970228000 | 0.813270000 |
| C | –1.278190000 | –0.712352000 | 0.194770000 |
| C | –4.129347000 | –5.564483000 | 2.884880000 |
| C | –3.961939000 | –4.149982000 | 2.313720000 |
| C | –2.871180000 | –5.948151000 | 3.673090000 |
| C | 6.883657000 | –0.793878000 | 2.884880000 |
| C | 5.574959000 | –1.356149000 | 2.313720000 |
| C | 7.850526000 | –0.474851000 | 1.735470000 |
| C | 6.586840000 | 0.487561000 | 3.673090000 |
| C | 3.677864000 | –2.136394000 | 0.068600000 |
| C | 4.909886000 | –0.336749000 | 1.360780000 |
| C | 2.547405000 | –2.692106000 | –0.546690000 |
| C | 7.215376000 | 0.564016000 | 0.801170000 |
| C | 3.633854000 | –0.913342000 | 0.751320000 |
| C | 5.908912000 | –0.001566000 | 0.228440000 |
| C | 1.347741000 | –1.985866000 | –0.477590000 |
| C | 2.413895000 | –0.240533000 | 0.813270000 |
| C | 1.256010000 | –0.750769000 | 0.194770000 |
| C | 5.947958000 | 1.520765000 | 2.737040000 |
| C | 4.645642000 | 0.955987000 | 2.155650000 |
| C | 6.915584000 | 1.844401000 | 1.591710000 |
| C | 2.556250000 | –5.672439000 | –3.751920000 |
| C | 3.893490000 | –5.262859000 | –3.121710000 |
| C | 3.727170000 | –3.920389000 | –2.397680000 |
| C | 1.497280000 | –5.807819000 | –2.650800000 |
| C | 1.338280000 | –4.465109000 | –1.925010000 |
| C | 2.665860000 | –4.026249000 | –1.279280000 |
| C | 4.333510000 | –6.333789000 | –2.113620000 |
| C | 1.934910000 | –6.885209000 | –1.650850000 |
| C | 3.270140000 | –6.476309000 | –1.016420000 |
| C | 3.101750000 | –5.133729000 | –0.291790000 |
| C | 2.895065000 | 5.253059000 | –0.291790000 |
| C | 2.153904000 | 4.321827000 | –1.279280000 |
| C | 1.531572000 | 5.188019000 | –2.397680000 |
| C | 3.973578000 | 6.070179000 | –1.016420000 |
| C | 3.197758000 | 3.391539000 | –1.925010000 |
| C | 3.318467000 | 6.919825000 | –2.113620000 |
| C | 2.611025000 | 6.003291000 | –3.121710000 |
| C | 4.995311000 | 5.118286000 | –1.650850000 |
| C | 4.281079000 | 4.200592000 | –2.650800000 |
| C | 3.634351000 | 5.049997000 | –3.751920000 |
| C | –4.536038000 | 1.073570000 | –1.925010000 |
| C | –4.819764000 | –0.295578000 | –1.279280000 |
| C | –5.996815000 | –0.119330000 | –0.291790000 |
| C | –5.778359000 | 1.607227000 | –2.650800000 |
| C | –5.258742000 | –1.267629000 | –2.397680000 |

| | | | |
|---|---|---|---|
| C | –6.930221000 | 1.766923000 | –1.650850000 |
| C | –7.243718000 | 0.406130000 | –1.016420000 |
| C | –6.190602000 | 0.622442000 | –3.751920000 |
| C | –6.504515000 | –0.740432000 | –3.121710000 |
| C | –7.651977000 | –0.586035000 | –2.113620000 |
| H | 1.820400000 | 1.577531000 | –0.972770000 |
| H | 0.000000000 | 5.343321000 | 0.006110000 |
| H | –1.770530000 | 1.665761000 | 1.353260000 |
| H | –2.615360000 | 3.025041000 | 2.967550000 |
| H | –3.573740000 | 2.769101000 | 1.495300000 |
| H | –0.904160000 | 6.155581000 | 1.775590000 |
| H | –1.050620000 | 5.020181000 | 3.128550000 |
| H | –2.272330000 | 5.754051000 | –0.361490000 |
| H | –3.350760000 | 4.351091000 | –0.458150000 |
| H | –4.972200000 | 3.734141000 | 3.299720000 |
| H | –4.531450000 | 6.064311000 | 4.103910000 |
| H | –3.183650000 | 4.982451000 | 4.512060000 |
| H | –5.489100000 | 4.302381000 | 0.925640000 |
| H | –5.890000000 | 5.664371000 | 1.993240000 |
| H | –2.332470000 | 7.125671000 | 3.552310000 |
| H | –4.637390000 | 6.451281000 | –0.026200000 |
| H | –4.324160000 | 7.665191000 | 2.139640000 |
| H | –2.835550000 | 7.699181000 | 1.173210000 |
| H | –3.268278000 | –7.241738000 | –0.026200000 |
| H | –3.846989000 | –4.844921000 | –0.361490000 |
| H | –2.092775000 | –5.077389000 | –0.458150000 |
| H | –0.981421000 | –6.904890000 | 0.925640000 |
| H | –1.960489000 | –7.933075000 | 1.993240000 |
| H | –4.476170000 | –7.577428000 | 2.139640000 |
| H | –5.249911000 | –6.305249000 | 1.173210000 |
| H | –4.627451000 | –2.671660000 | 0.006110000 |
| H | –0.747761000 | –6.173122000 | 3.299720000 |
| H | –1.312082000 | –3.777488000 | 2.967550000 |
| H | –0.611242000 | –4.479500000 | 1.495300000 |
| H | –2.276382000 | 0.787747000 | –0.972770000 |
| H | –0.557326000 | –2.366204000 | 1.353260000 |
| H | –5.004777000 | –5.582814000 | 3.552310000 |
| H | –4.878809000 | –3.860816000 | 1.775590000 |
| H | –3.822294000 | –3.419954000 | 3.128550000 |
| H | –2.986122000 | –6.956506000 | 4.103910000 |
| H | –2.723104000 | –5.248347000 | 4.512060000 |
| H | 7.337247000 | –1.542857000 | 3.552310000 |
| H | 5.782969000 | –2.294765000 | 1.775590000 |
| H | 4.872914000 | –1.600227000 | 3.128550000 |
| H | 8.800330000 | –0.087763000 | 2.139640000 |
| H | 8.085461000 | –1.393932000 | 1.173210000 |
| H | 7.517572000 | 0.892196000 | 4.103910000 |
| H | 5.906754000 | 0.265896000 | 4.512060000 |
| H | 4.627451000 | –2.671660000 | 0.006110000 |
| H | 7.905668000 | 0.790457000 | –0.026200000 |
| H | 6.119319000 | –0.909130000 | –0.361490000 |
| H | 5.443535000 | 0.726298000 | –0.458150000 |
| H | 0.455982000 | –2.365278000 | –0.972770000 |
| H | 2.327856000 | 0.700444000 | 1.353260000 |
| H | 5.719961000 | 2.438981000 | 3.299720000 |
| H | 3.927442000 | 0.752448000 | 2.967550000 |
| H | 4.184981000 | 1.710399000 | 1.495300000 |
| H | 6.470521000 | 2.602510000 | 0.925640000 |
| H | 7.850489000 | 2.268704000 | 1.993240000 |
| H | 2.667110000 | –6.629299000 | –4.288440000 |
| H | 2.239960000 | –4.917349000 | –4.490230000 |
| H | 4.657560000 | –5.158109000 | –3.907550000 |
| H | 3.423230000 | –3.134579000 | –3.109320000 |
| H | 4.692950000 | –3.608679000 | –1.967940000 |
| H | 0.531550000 | –6.088969000 | –3.098620000 |
| H | 0.557770000 | –4.550659000 | –1.148950000 |
| H | 1.001020000 | –3.696919000 | –2.640530000 |
| H | 5.302170000 | –6.054929000 | –1.665950000 |
| H | 4.477130000 | –7.298969000 | –2.626810000 |
| H | 2.040620000 | –7.855439000 | –2.163970000 |
| H | 1.167360000 | –7.010549000 | –0.868920000 |
| H | 3.586290000 | –7.241379000 | –0.290520000 |
| H | 4.050610000 | –4.849299000 | 0.191800000 |
| H | 2.346430000 | –5.219429000 | 0.508140000 |
| H | 2.174311000 | 5.932581000 | 0.191800000 |
| H | 3.346943000 | 4.641783000 | 0.508140000 |
| H | 1.003010000 | 4.531894000 | –3.109320000 |
| H | 0.778733000 | 5.868554000 | –1.967940000 |
| H | 4.478073000 | 6.726508000 | –0.290520000 |
| H | 3.662101000 | 2.758373000 | –1.148950000 |
| H | 2.701116000 | 2.715369000 | –2.640530000 |
| H | 2.592637000 | 7.619279000 | –1.665950000 |
| H | 4.082528000 | 7.526793000 | –2.626810000 |
| H | 2.138273000 | 6.612620000 | –3.907550000 |
| H | 5.782700000 | 5.694949000 | –2.163970000 |
| H | 5.487634000 | 4.516238000 | –0.868920000 |
| H | 5.007427000 | 3.504821000 | –3.098620000 |
| H | 4.407586000 | 5.624435000 | –4.288440000 |
| H | 3.138569000 | 4.398537000 | –4.490230000 |
| H | –4.219872000 | 1.792286000 | –1.148950000 |
| H | –3.702136000 | 0.981551000 | –2.640530000 |
| H | –6.224921000 | –1.083282000 | 0.191800000 |
| H | –5.693373000 | 0.577646000 | 0.508140000 |
| H | –5.538977000 | 2.584149000 | –3.098620000 |
| H | –4.426240000 | –1.397315000 | –3.109320000 |
| H | –5.471683000 | –2.259874000 | –1.967940000 |
| H | –7.823320000 | 2.160491000 | –2.163970000 |
| H | –6.654994000 | 2.494311000 | –0.868920000 |
| H | –8.064364000 | 0.514871000 | –0.290520000 |
| H | –7.074697000 | 1.004864000 | –4.288440000 |
| H | –5.378529000 | 0.518812000 | –4.490230000 |
| H | –6.795834000 | –1.454511000 | –3.907550000 |
| H | –7.894808000 | –1.564350000 | –1.665950000 |
| H | –8.559658000 | –0.227824000 | –2.626810000 |

**Table S15.** Radical monomers R-**1**• with C–PCM:cyclohexane.

H-**1**•

B3LYP

| | | | |
|---|---|---|---|
| C | –0.004010000 | 4.307178000 | 0.005427000 |
| C | –0.996663000 | 3.600012000 | –0.683838000 |
| C | –0.999020000 | 2.206174000 | –0.686651000 |
| C | 0.000000000 | 1.466289000 | –0.004707000 |
| C | 0.997743000 | 2.204276000 | 0.681372000 |
| C | 0.991278000 | 3.597917000 | 0.689051000 |
| C | –1.269844000 | –0.733145000 | –0.004707000 |
| C | –2.407830000 | –0.238068000 | 0.681372000 |
| C | –3.611526000 | –0.940487000 | 0.689051000 |
| C | –3.728121000 | –2.157062000 | 0.005427000 |
| C | –2.619370000 | –2.663142000 | –0.683838000 |
| C | –1.411093000 | –1.968263000 | –0.686651000 |
| C | 3.616034000 | –0.936871000 | –0.683838000 |
| C | 2.410113000 | –0.237910000 | –0.686651000 |
| C | 1.269844000 | –0.733145000 | –0.004707000 |
| C | 1.410088000 | –1.966209000 | 0.681372000 |
| C | 2.620249000 | –2.657430000 | 0.689051000 |
| C | 3.732131000 | –2.150116000 | 0.005427000 |
| C | 0.000000000 | 0.000000000 | –0.006735000 |
| H | –0.005650000 | 5.399338000 | 0.009750000 |
| H | –1.772932000 | 4.140279000 | –1.231082000 |
| H | –1.770479000 | 1.671848000 | –1.243410000 |
| H | 1.770531000 | 1.667840000 | 1.234155000 |
| H | 1.765885000 | 4.136693000 | 1.240133000 |
| H | –2.329657000 | 0.699405000 | 1.234155000 |
| H | –4.465424000 | –0.539046000 | 1.240133000 |
| H | –4.673139000 | –2.704562000 | 0.009750000 |
| H | –2.699121000 | –3.605544000 | –1.231082000 |
| H | –0.562623000 | –2.369203000 | –1.243410000 |
| H | 4.472053000 | –0.534735000 | –1.231082000 |
| H | 2.333102000 | 0.697356000 | –1.243410000 |
| H | 0.559126000 | –2.367245000 | 1.234155000 |
| H | 2.699539000 | –3.597648000 | 1.240133000 |
| H | 4.678789000 | –2.694776000 | 0.009750000 |

B3LYP-D3(BJ)

| | | | |
|---|---|---|---|
| C | –0.004917000 | 4.296865000 | 0.006150000 |
| C | –1.006721000 | 3.590478000 | –0.670055000 |
| C | –1.008149000 | 2.197761000 | –0.674210000 |
| C | 0.000000000 | 1.461255000 | –0.005351000 |
| C | 1.006337000 | 2.195937000 | 0.668266000 |
| C | 0.999950000 | 3.588521000 | 0.675921000 |
| C | –1.265484000 | –0.730627000 | –0.005351000 |
| C | –2.404906000 | –0.226455000 | 0.668266000 |
| C | –3.607725000 | –0.928279000 | 0.675921000 |
| C | –3.718736000 | –2.152691000 | 0.006150000 |
| C | –2.606085000 | –2.667085000 | –0.670055000 |
| C | –1.399242000 | –1.971963000 | –0.674210000 |
| C | 3.612806000 | –0.923393000 | –0.670055000 |
| C | 2.407391000 | –0.225798000 | –0.674210000 |
| C | 1.265484000 | –0.730627000 | –0.005351000 |

| | | | |
|---|---|---|---|
| C | 1.398569000 | –1.969482000 | 0.668266000 |
| C | 2.607775000 | –2.660242000 | 0.675921000 |
| C | 3.723653000 | –2.144174000 | 0.006150000 |
| C | 0.000000000 | 0.000000000 | –0.007596000 |
| H | –0.006898000 | 5.388621000 | 0.011083000 |
| H | –1.790033000 | 4.131142000 | –1.205907000 |
| H | –1.784513000 | 1.660025000 | –1.219698000 |
| H | 1.784507000 | 1.656180000 | 1.209165000 |
| H | 1.781211000 | 4.127762000 | 1.216223000 |
| H | –2.326548000 | 0.717338000 | 1.209165000 |
| H | –4.465352000 | –0.521307000 | 1.216223000 |
| H | –4.663234000 | –2.700285000 | 0.011083000 |
| H | –2.682657000 | –3.615785000 | –1.205907000 |
| H | –0.545368000 | –2.375446000 | –1.219698000 |
| H | 4.472690000 | –0.515357000 | –1.205907000 |
| H | 2.329881000 | 0.715421000 | –1.219698000 |
| H | 0.542041000 | –2.373519000 | 1.209165000 |
| H | 2.684141000 | –3.606455000 | 1.216223000 |
| H | 4.670132000 | –2.688337000 | 0.011083000 |

M06-2X

| | | | |
|---|---|---|---|
| C | –0.009750000 | 4.288822000 | 0.012618000 |
| C | –1.012991000 | 3.586257000 | –0.657696000 |
| C | –1.011871000 | 2.195859000 | –0.667674000 |
| C | 0.000000000 | 1.462601000 | –0.011210000 |
| C | 1.008043000 | 2.191642000 | 0.655737000 |
| C | 0.999108000 | 3.581961000 | 0.669554000 |
| C | –1.266649000 | –0.731300000 | –0.011210000 |
| C | –2.402039000 | –0.222830000 | 0.655737000 |
| C | –3.601623000 | –0.925728000 | 0.669554000 |
| C | –3.709354000 | –2.152854000 | 0.012618000 |
| C | –2.599294000 | –2.670405000 | –0.657696000 |
| C | –1.395734000 | –1.974236000 | –0.667674000 |
| C | 3.612285000 | –0.915853000 | –0.657696000 |
| C | 2.407605000 | –0.221623000 | –0.667674000 |
| C | 1.266649000 | –0.731300000 | –0.011210000 |
| C | 1.393996000 | –1.968812000 | 0.655737000 |
| C | 2.602515000 | –2.656234000 | 0.669554000 |
| C | 3.719104000 | –2.135968000 | 0.012618000 |
| C | 0.000000000 | 0.000000000 | –0.016349000 |
| H | –0.013320000 | 5.379321000 | 0.022898000 |
| H | –1.801381000 | 4.126989000 | –1.183462000 |
| H | –1.793448000 | 1.657704000 | –1.206270000 |
| H | 1.792714000 | 1.650611000 | 1.186787000 |
| H | 1.783454000 | 4.119209000 | 1.204777000 |
| H | –2.325828000 | 0.727230000 | 1.186787000 |
| H | –4.459067000 | –0.515088000 | 1.204777000 |
| H | –4.651969000 | –2.701196000 | 0.022898000 |
| H | –2.673387000 | –3.623536000 | –1.183462000 |
| H | –0.538890000 | –2.382024000 | –1.206270000 |
| H | 4.474768000 | –0.503453000 | –1.183462000 |
| H | 2.332338000 | 0.724320000 | –1.206270000 |
| H | 0.533114000 | –2.377841000 | 1.186787000 |
| H | 2.675612000 | –3.604121000 | 1.204777000 |
| H | 4.665289000 | –2.678125000 | 0.022898000 |

Me-**1**•

B3LYP

| | | | |
|---|---|---|---|
| C | –0.006662000 | 4.287189000 | 0.006765000 |
| C | –1.004004000 | 3.602184000 | –0.699047000 |
| C | –0.993409000 | 2.201336000 | –0.691426000 |
| C | 0.000000000 | 1.467202000 | –0.005013000 |
| C | 0.995313000 | 2.202665000 | 0.688949000 |
| C | 0.999515000 | 3.597351000 | 0.708605000 |
| C | –1.270634000 | –0.733601000 | –0.005013000 |
| C | –2.405220000 | –0.239366000 | 0.688949000 |
| C | –3.615155000 | –0.933070000 | 0.708605000 |
| C | –3.709484000 | –2.149364000 | 0.006765000 |
| C | –2.617581000 | –2.670585000 | –0.699047000 |
| C | –1.409709000 | –1.960986000 | –0.691426000 |
| C | 3.621585000 | –0.931599000 | –0.699047000 |
| C | 2.403118000 | –0.240350000 | –0.691426000 |
| C | 1.270634000 | –0.733601000 | –0.005013000 |
| C | 1.409908000 | –1.963299000 | 0.688949000 |
| C | 2.615640000 | –2.664281000 | 0.708605000 |
| C | 3.716146000 | –2.137825000 | 0.006765000 |
| C | 0.000000000 | 0.000000000 | –0.004517000 |
| C | –2.064450000 | 4.349161000 | –1.475296000 |
| C | 2.053671000 | 4.364080000 | 1.473005000 |
| C | –4.806240000 | –0.403509000 | 1.473005000 |
| C | –2.734259000 | –3.962447000 | –1.475296000 |
| C | 4.798709000 | –0.386714000 | –1.475296000 |
| C | 2.752569000 | –3.960571000 | 1.473005000 |
| H | –0.010088000 | 5.381323000 | 0.012870000 |
| H | –1.760514000 | 1.661232000 | –1.250226000 |
| H | 1.761907000 | 1.660501000 | 1.245711000 |
| H | –2.318990000 | 0.695605000 | 1.245711000 |
| H | –4.655319000 | –2.699398000 | 0.012870000 |
| H | –0.558412000 | –2.355266000 | –1.250226000 |
| H | 2.318926000 | 0.694034000 | –1.250226000 |
| H | 0.557083000 | –2.356107000 | 1.245711000 |
| H | 4.665407000 | –2.681925000 | 0.012870000 |
| H | –1.932507000 | 4.211658000 | –2.562378000 |
| H | –2.030917000 | 5.429127000 | –1.268894000 |
| H | –3.074779000 | 3.984773000 | –1.227329000 |
| H | 1.599271000 | 5.009938000 | 2.243239000 |
| H | 2.631228000 | 5.023397000 | 0.803010000 |
| H | 2.761600000 | 3.687444000 | 1.973785000 |
| H | –5.138369000 | –1.119959000 | 2.243239000 |
| H | –5.666004000 | –0.232988000 | 0.803010000 |
| H | –4.574220000 | 0.547894000 | 1.973785000 |
| H | –2.681150000 | –3.779429000 | –2.562378000 |
| H | –3.686303000 | –4.473389000 | –1.268894000 |
| H | –1.913525000 | –4.655223000 | –1.227329000 |
| H | 4.613657000 | –0.432229000 | –2.562378000 |
| H | 5.717220000 | –0.955738000 | –1.268894000 |
| H | 4.988304000 | 0.670450000 | –1.227329000 |
| H | 3.539098000 | –3.889978000 | 2.243239000 |
| H | 3.034776000 | –4.790409000 | 0.803010000 |
| H | 1.812620000 | –4.235338000 | 1.973785000 |

B3LYP-D3(BJ)

symmetry c3

| | | | |
|---|---|---|---|
| C | 0.006897000 | 4.277324000 | –0.006857000 |
| C | 1.018470000 | 3.592326000 | 0.676755000 |
| C | 1.006558000 | 2.193059000 | 0.673134000 |
| C | 0.000000000 | 1.461710000 | 0.006723000 |
| C | –1.008356000 | 2.193915000 | –0.668262000 |
| C | –1.013502000 | 3.587115000 | –0.685942000 |
| C | 1.265878000 | –0.730855000 | 0.006723000 |
| C | 2.404164000 | –0.223696000 | –0.668262000 |
| C | 3.613284000 | –0.915839000 | –0.685942000 |
| C | 3.700823000 | –2.144635000 | –0.006857000 |
| C | 2.601811000 | –2.678184000 | 0.676755000 |
| C | 1.395966000 | –1.968235000 | 0.673134000 |
| C | –3.620281000 | –0.914142000 | 0.676755000 |
| C | –2.402524000 | –0.224825000 | 0.673134000 |
| C | –1.265878000 | –0.730855000 | 0.006723000 |
| C | –1.395808000 | –1.970219000 | –0.668262000 |
| C | –2.599782000 | –2.671276000 | –0.685942000 |
| C | –3.707720000 | –2.132689000 | –0.006857000 |
| C | 0.000000000 | 0.000000000 | 0.007022000 |
| C | 2.098217000 | 4.337646000 | 1.423566000 |
| C | –2.084153000 | 4.352231000 | –1.424652000 |
| C | 4.811219000 | –0.371186000 | –1.424652000 |
| C | 2.707403000 | –3.985933000 | 1.423566000 |
| C | –4.805621000 | –0.351714000 | 1.423566000 |
| C | –2.727066000 | –3.981045000 | –1.424652000 |
| H | 0.010081000 | 5.371015000 | –0.013414000 |
| H | 1.782784000 | 1.651722000 | 1.216665000 |
| H | –1.784467000 | 1.649737000 | –1.208234000 |
| H | 2.320947000 | 0.720525000 | –1.208234000 |
| H | 4.646395000 | –2.694238000 | –0.013414000 |
| H | 0.539041000 | –2.369798000 | 1.216665000 |
| H | –2.321826000 | 0.718075000 | 1.216665000 |
| H | –0.536481000 | –2.370262000 | –1.208234000 |
| H | –4.656476000 | –2.676777000 | –0.013414000 |
| H | 2.010938000 | 4.176988000 | 2.511443000 |
| H | 2.043519000 | 5.420259000 | 1.238347000 |
| H | 3.100939000 | 3.989299000 | 1.127207000 |
| H | –1.647310000 | 4.996199000 | –2.206017000 |
| H | –2.643616000 | 5.012243000 | –0.740753000 |
| H | –2.803564000 | 3.674202000 | –1.906083000 |
| H | 5.150490000 | –1.071487000 | –2.206017000 |
| H | 5.662538000 | –0.216683000 | –0.740753000 |
| H | 4.583735000 | 0.590857000 | –1.906083000 |
| H | 2.611909000 | –3.830018000 | 2.511443000 |
| H | 3.672323000 | –4.479869000 | 1.238347000 |
| H | 1.904364000 | –4.680141000 | 1.127207000 |
| H | –4.622847000 | –0.346971000 | 2.511443000 |
| H | –5.715842000 | –0.940390000 | 1.238347000 |
| H | –5.005304000 | 0.690843000 | 1.127207000 |
| H | –3.503180000 | –3.924712000 | –2.206017000 |
| H | –3.018922000 | –4.795560000 | –0.740753000 |
| H | –1.780170000 | –4.265059000 | –1.906083000 |

M06-2X

| | | | |
|---|---|---|---|
| C | 0.000000000 | 4.269860000 | –0.009813000 |

| | | | |
|---|---|---|---|
| C | 1.011971000 | 3.592854000 | 0.672272000 |
| C | 1.001196000 | 2.195567000 | 0.678242000 |
| C | –0.003885000 | 1.463178000 | 0.021111000 |
| C | –1.013619000 | 2.186300000 | –0.653289000 |
| C | –1.019123000 | 3.576473000 | –0.679920000 |
| C | 1.269092000 | –0.728224000 | 0.021111000 |
| C | 2.400201000 | –0.215330000 | –0.653289000 |
| C | 3.606878000 | –0.905650000 | –0.679920000 |
| C | 3.697807000 | –2.134930000 | –0.009813000 |
| C | 2.605517000 | –2.672820000 | 0.672272000 |
| C | 1.400819000 | –1.964844000 | 0.678242000 |
| C | –3.617489000 | –0.920034000 | 0.672272000 |
| C | –2.402014000 | –0.230723000 | 0.678242000 |
| C | –1.265207000 | –0.734954000 | 0.021111000 |
| C | –1.386582000 | –1.970970000 | –0.653289000 |
| C | –2.587755000 | –2.670823000 | –0.679920000 |
| C | –3.697807000 | –2.134930000 | –0.009813000 |
| C | 0.000000000 | 0.000000000 | 0.026193000 |
| C | 2.104025000 | 4.341366000 | 1.393187000 |
| C | –2.089637000 | 4.337839000 | –1.418869000 |
| C | 4.801497000 | –0.359241000 | –1.418869000 |
| C | 2.707721000 | –3.992822000 | 1.393187000 |
| C | –4.811745000 | –0.348544000 | 1.393187000 |
| C | –2.711861000 | –3.978598000 | –1.418869000 |
| H | –0.000148000 | 5.363268000 | –0.022851000 |
| H | 1.781787000 | 1.657929000 | 1.221310000 |
| H | –1.793490000 | 1.637380000 | –1.184862000 |
| H | 2.314758000 | 0.734518000 | –1.184862000 |
| H | 4.644800000 | –2.681506000 | –0.022851000 |
| H | 0.544915000 | –2.372038000 | 1.221310000 |
| H | –2.326703000 | 0.714108000 | 1.221310000 |
| H | –0.521268000 | –2.371898000 | –1.184862000 |
| H | –4.644653000 | –2.681762000 | –0.022851000 |
| H | 2.158896000 | 4.036855000 | 2.448539000 |
| H | 1.936937000 | 5.425677000 | 1.356609000 |
| H | 3.085627000 | 4.131409000 | 0.942135000 |
| H | –1.649581000 | 4.968548000 | –2.205656000 |
| H | –2.638952000 | 5.003465000 | –0.736390000 |
| H | –2.811160000 | 3.656919000 | –1.888754000 |
| H | 5.127679000 | –1.055695000 | –2.205656000 |
| H | 5.652604000 | –0.216333000 | –0.736390000 |
| H | 4.572564000 | 0.606076000 | –1.888754000 |
| H | 2.416572000 | –3.888086000 | 2.448539000 |
| H | 3.730306000 | –4.390275000 | 1.356609000 |
| H | 2.035092000 | –4.737936000 | 0.942135000 |
| H | –4.575467000 | –0.148769000 | 2.448539000 |
| H | –5.667242000 | –1.035402000 | 1.356609000 |
| H | –5.120719000 | 0.606527000 | 0.942135000 |
| H | –3.478098000 | –3.912852000 | –2.205656000 |
| H | –3.013652000 | –4.787132000 | –0.736390000 |
| H | –1.761404000 | –4.262995000 | –1.888754000 |

$^{i}$Pr-**1•**

B3LYP

| | | | |
|---|---|---|---|
| C | 0.007609000 | 4.280661000 | –0.017743000 |
| C | 0.988661000 | 3.600725000 | 0.721828000 |
| C | 0.979822000 | 2.201917000 | 0.712179000 |
| C | 0.000000000 | 1.468070000 | 0.001073000 |
| C | –0.977738000 | 2.197923000 | –0.716994000 |
| C | –0.979076000 | 3.596304000 | –0.745920000 |
| C | 1.271386000 | –0.734035000 | 0.001073000 |
| C | 2.392326000 | –0.252215000 | –0.716994000 |
| C | 3.604029000 | –0.950248000 | –0.745920000 |
| C | 3.703356000 | –2.146920000 | –0.017743000 |
| C | 2.623989000 | –2.656568000 | 0.721828000 |
| C | 1.417005000 | –1.949510000 | 0.712179000 |
| C | –3.612650000 | –0.944157000 | 0.721828000 |
| C | –2.396827000 | –0.252408000 | 0.712179000 |
| C | –1.271386000 | –0.734035000 | 0.001073000 |
| C | –1.414588000 | –1.945707000 | –0.716994000 |
| C | –2.624953000 | –2.646056000 | –0.745920000 |
| C | –3.710965000 | –2.133741000 | –0.017743000 |
| C | 0.000000000 | 0.000000000 | 0.004508000 |
| C | –2.779098000 | –3.934503000 | –1.547809000 |
| C | –2.547890000 | –3.707911000 | –3.052386000 |
| C | –1.872851000 | –5.056886000 | –1.012085000 |
| C | –4.806097000 | –0.439789000 | 1.527699000 |
| C | –5.271369000 | 0.953294000 | 1.070952000 |
| C | –4.519988000 | –0.459705000 | 3.040221000 |
| C | 4.796928000 | –0.439518000 | –1.547809000 |
| C | 5.315817000 | 0.906506000 | –1.012085000 |
| C | 4.485090000 | –0.352582000 | –3.052386000 |
| C | 2.783917000 | –3.942308000 | 1.527699000 |
| C | 1.810108000 | –5.041786000 | 1.070952000 |
| C | 2.658110000 | –3.684572000 | 3.040221000 |
| C | 2.022180000 | 4.382097000 | 1.527699000 |
| C | 1.861878000 | 4.144277000 | 3.040221000 |
| C | 3.461261000 | 4.088492000 | 1.070952000 |
| C | –2.017830000 | 4.374021000 | –1.547809000 |
| C | –1.937200000 | 4.060493000 | –3.052386000 |
| C | –3.442966000 | 4.150380000 | –1.012085000 |
| H | 0.011238000 | 5.374921000 | –0.025424000 |
| H | 1.727365000 | 1.652247000 | 1.286794000 |
| H | –1.728752000 | 1.644600000 | –1.283175000 |
| H | 2.288642000 | 0.674843000 | –1.283175000 |
| H | 4.649199000 | –2.697193000 | –0.025424000 |
| H | 0.567206000 | –2.322065000 | 1.286794000 |
| H | –2.294571000 | 0.669818000 | 1.286794000 |
| H | –0.559890000 | –2.319443000 | –1.283175000 |
| H | –4.660437000 | –2.677728000 | –0.025424000 |
| H | –3.824786000 | –4.264740000 | –1.420657000 |
| H | –1.511048000 | –3.392820000 | –3.256743000 |
| H | –3.220058000 | –2.929679000 | –3.447688000 |
| H | –2.730583000 | –4.637096000 | –3.617343000 |
| H | –0.806478000 | –4.800170000 | –1.125124000 |
| H | –2.050187000 | –5.996065000 | –1.562157000 |
| H | –2.060854000 | –5.246560000 | 0.056902000 |
| H | –5.635580000 | –1.143679000 | 1.340011000 |
| H | –5.510012000 | 0.962821000 | –0.004667000 |
| H | –4.495852000 | 1.716376000 | 1.250054000 |
| H | –6.174510000 | 1.261170000 | 1.623641000 |
| H | –4.227034000 | –1.466597000 | 3.378118000 |
| H | –5.413635000 | –0.154182000 | 3.609555000 |
| H | –3.703327000 | 0.233556000 | 3.302800000 |
| H | 5.605766000 | –1.179992000 | –1.420657000 |
| H | 4.560308000 | 1.701655000 | –1.125124000 |
| H | 6.217839000 | 1.222518000 | –1.562157000 |
| H | 5.574081000 | 0.838528000 | 0.056902000 |
| H | 3.693793000 | 0.387804000 | –3.256743000 |
| H | 4.147206000 | –1.323813000 | –3.447688000 |
| H | 5.381135000 | –0.046206000 | –3.617343000 |
| H | 3.808245000 | –4.308716000 | 1.340011000 |
| H | 1.921179000 | –5.253221000 | –0.004667000 |
| H | 0.761500000 | –4.751710000 | 1.250054000 |
| H | 1.995049000 | –5.977868000 | 1.623641000 |
| H | 3.383628000 | –2.927421000 | 3.378118000 |
| H | 2.840343000 | –4.611255000 | 3.609555000 |
| H | 1.649398000 | –3.323953000 | 3.302800000 |
| H | 1.827335000 | 5.452395000 | 1.340011000 |
| H | 0.843407000 | 4.394018000 | 3.378118000 |
| H | 2.573292000 | 4.765436000 | 3.609555000 |
| H | 2.053929000 | 3.090397000 | 3.302800000 |
| H | 3.588834000 | 4.290400000 | –0.004667000 |
| H | 3.734352000 | 3.035334000 | 1.250054000 |
| H | 4.179461000 | 4.716697000 | 1.623641000 |
| H | –1.780980000 | 5.444731000 | –1.420657000 |
| H | –2.182744000 | 3.005016000 | –3.256743000 |
| H | –0.927147000 | 4.253492000 | –3.447688000 |
| H | –2.650552000 | 4.683302000 | –3.617343000 |
| H | –3.753830000 | 3.098515000 | –1.125124000 |
| H | –4.167651000 | 4.773547000 | –1.562157000 |
| H | –3.513227000 | 4.408032000 | 0.056902000 |

B3LYP-D3(BJ)

| | | | |
|---|---|---|---|
| C | 0.000000000 | 4.272238000 | –0.006373000 |
| C | 1.015047000 | 3.590363000 | 0.681302000 |
| C | 1.004413000 | 2.193966000 | 0.677565000 |
| C | –0.002050000 | 1.461949000 | 0.006843000 |
| C | –1.008492000 | 2.189361000 | –0.668878000 |
| C | –1.017330000 | 3.585616000 | –0.686007000 |
| C | 1.267110000 | –0.729199000 | 0.006843000 |
| C | 2.400288000 | –0.221301000 | –0.668878000 |
| C | 3.613899000 | –0.911774000 | –0.686007000 |
| C | 3.699867000 | –2.136119000 | –0.006373000 |
| C | 2.601822000 | –2.674238000 | 0.681302000 |
| C | 1.397824000 | –1.966830000 | 0.677565000 |
| C | –3.616869000 | –0.916125000 | 0.681302000 |
| C | –2.402237000 | –0.227136000 | 0.677565000 |
| C | –1.265060000 | –0.732750000 | 0.006843000 |
| C | –1.391796000 | –1.968060000 | –0.668878000 |
| C | –2.596569000 | –2.673841000 | –0.686007000 |
| C | –3.699867000 | –2.136119000 | –0.006373000 |
| C | 0.000000000 | 0.000000000 | 0.009431000 |
| C | –2.720442000 | –3.985921000 | –1.445467000 |
| C | –2.595244000 | –3.766371000 | –2.961539000 |
| C | –1.705588000 | –5.028116000 | –0.953250000 |
| C | –4.819332000 | –0.373165000 | 1.437236000 |
| C | –5.207133000 | 1.034271000 | 0.961022000 |
| C | –4.576264000 | –0.395135000 | 2.954788000 |
| C | 4.812130000 | –0.363012000 | –1.445467000 |
| C | 5.207270000 | 1.036975000 | –0.953250000 |
| C | 4.559395000 | –0.364362000 | –2.961539000 |
| C | 2.732836000 | –3.987082000 | 1.437236000 |

| | | | |
|---|---|---|---|
| C | 1.707862000 | –5.026645000 | 0.961022000 |
| C | 2.630329000 | –3.765594000 | 2.954788000 |
| C | 2.086496000 | 4.360246000 | 1.437236000 |
| C | 1.945935000 | 4.160728000 | 2.954788000 |
| C | 3.499271000 | 3.992374000 | 0.961022000 |
| C | –2.091688000 | 4.348932000 | –1.445467000 |
| C | –1.964151000 | 4.130733000 | –2.961539000 |
| C | –3.501682000 | 3.991141000 | –0.953250000 |
| H | 0.001202000 | 5.365949000 | –0.011337000 |
| H | 1.773674000 | 1.645342000 | 1.221671000 |
| H | –1.779433000 | 1.636686000 | –1.206270000 |
| H | 2.307128000 | 0.722691000 | –1.206270000 |
| H | 4.646447000 | –2.684015000 | –0.011337000 |
| H | 0.538071000 | –2.358718000 | 1.221671000 |
| H | –2.311745000 | 0.713376000 | 1.221671000 |
| H | –0.527695000 | –2.359377000 | –1.206270000 |
| H | –4.647649000 | –2.681933000 | –0.011337000 |
| H | –3.731821000 | –4.379492000 | –1.245920000 |
| H | –1.597624000 | –3.375562000 | –3.222282000 |
| H | –3.343923000 | –3.043205000 | –3.321414000 |
| H | –2.739128000 | –4.713401000 | –3.507144000 |
| H | –0.671563000 | –4.703601000 | –1.152078000 |
| H | –1.857216000 | –5.992056000 | –1.465721000 |
| H | –1.800813000 | –5.195416000 | 0.131168000 |
| H | –5.666403000 | –1.047174000 | 1.223271000 |
| H | –5.396750000 | 1.049599000 | –0.123767000 |
| H | –4.406625000 | 1.761652000 | 1.171014000 |
| H | –6.117753000 | 1.382389000 | 1.475160000 |
| H | –4.328713000 | –1.410343000 | 3.302765000 |
| H | –5.470826000 | –0.051283000 | 3.499560000 |
| H | –3.738878000 | 0.267903000 | 3.229013000 |
| H | 5.658662000 | –1.042105000 | –1.245920000 |
| H | 4.409219000 | 1.770210000 | –1.152078000 |
| H | 6.117881000 | 1.387632000 | –1.465721000 |
| H | 5.399769000 | 1.038158000 | 0.131168000 |
| H | 3.722134000 | 0.304198000 | –3.222282000 |
| H | 4.307455000 | –1.374320000 | –3.321414000 |
| H | 5.451489000 | –0.015454000 | –3.507144000 |
| H | 3.740081000 | –4.383662000 | 1.223271000 |
| H | 1.789396000 | –5.198522000 | –0.123767000 |
| H | 0.677677000 | –4.697076000 | 1.171014000 |
| H | 1.861692000 | –5.989324000 | 1.475160000 |
| H | 3.385750000 | –3.043604000 | 3.302765000 |
| H | 2.779826000 | –4.712232000 | 3.499560000 |
| H | 1.637428000 | –3.371915000 | 3.229013000 |
| H | 1.926322000 | 5.430836000 | 1.223271000 |
| H | 0.942963000 | 4.453947000 | 3.302765000 |
| H | 2.691000000 | 4.763516000 | 3.499560000 |
| H | 2.101450000 | 3.104012000 | 3.229013000 |
| H | 3.607354000 | 4.148923000 | –0.123767000 |
| H | 3.728948000 | 2.935423000 | 1.171014000 |
| H | 4.256060000 | 4.606935000 | 1.475160000 |
| H | –1.926841000 | 5.421598000 | –1.245920000 |
| H | –2.124511000 | 3.071364000 | –3.222282000 |
| H | –0.963531000 | 4.417525000 | –3.321414000 |
| H | –2.712361000 | 4.728855000 | –3.507144000 |
| H | –3.737656000 | 2.933391000 | –1.152078000 |
| H | –4.260665000 | 4.604425000 | –1.465721000 |
| H | –3.598956000 | 4.157258000 | 0.131168000 |

M06-2X

| | | | |
|---|---|---|---|
| C | 0.000000000 | 4.266751000 | –0.005760000 |
| C | 1.010990000 | 3.589341000 | 0.685080000 |
| C | 1.001050000 | 2.194701000 | 0.686920000 |
| C | –0.003620000 | 1.463511000 | 0.021450000 |
| C | –1.007980000 | 2.184421000 | –0.655850000 |
| C | –1.014070000 | 3.578621000 | –0.681510000 |
| C | 1.269247000 | –0.728620000 | 0.021450000 |
| C | 2.395754000 | –0.219274000 | –0.655850000 |
| C | 3.606211000 | –0.911100000 | –0.681510000 |
| C | 3.695114000 | –2.133375000 | –0.005760000 |
| C | 2.602965000 | –2.670213000 | 0.685080000 |
| C | 1.400142000 | –1.964285000 | 0.686920000 |
| C | –3.613955000 | –0.919127000 | 0.685080000 |
| C | –2.401191000 | –0.230416000 | 0.686920000 |
| C | –1.265627000 | –0.734890000 | 0.021450000 |
| C | –1.387774000 | –1.965147000 | –0.655850000 |
| C | –2.592141000 | –2.667521000 | –0.681510000 |
| C | –3.695114000 | –2.133375000 | –0.005760000 |
| C | 0.000000000 | 0.000000000 | 0.027300000 |
| C | –2.714750000 | –3.968539000 | –1.455690000 |
| C | –2.599380000 | –3.722559000 | –2.963470000 |
| C | –1.685221000 | –5.001829000 | –0.992050000 |
| C | –4.813790000 | –0.378859000 | 1.441300000 |
| C | –5.190120000 | 1.029021000 | 0.973360000 |
| C | –4.563490000 | –0.402239000 | 2.952550000 |
| C | 4.794231000 | –0.366773000 | –1.455690000 |
| C | 5.174321000 | 1.041471000 | –0.992050000 |
| C | 4.523521000 | –0.389850000 | –2.963470000 |
| C | 2.734997000 | –3.979435000 | 1.441300000 |
| C | 1.703902000 | –5.009286000 | 0.973360000 |
| C | 2.630094000 | –3.750979000 | 2.952550000 |
| C | 2.078794000 | 4.358294000 | 1.441300000 |
| C | 1.933396000 | 4.153218000 | 2.952550000 |
| C | 3.486218000 | 3.980265000 | 0.973360000 |
| C | –2.079481000 | 4.335313000 | –1.455690000 |
| C | –1.924141000 | 4.112409000 | –2.963470000 |
| C | –3.489101000 | 3.960358000 | –0.992050000 |
| H | 0.001150000 | 5.360391000 | –0.017010000 |
| H | 1.772930000 | 1.646771000 | 1.232100000 |
| H | –1.780250000 | 1.628271000 | –1.191870000 |
| H | 2.300249000 | 0.727606000 | –1.191870000 |
| H | 4.641659000 | –2.681191000 | –0.017010000 |
| H | 0.539680000 | –2.358788000 | 1.232100000 |
| H | –2.312610000 | 0.712017000 | 1.232100000 |
| H | –0.519999000 | –2.355877000 | –1.191870000 |
| H | –4.642809000 | –2.679199000 | –0.017010000 |
| H | –3.719861000 | –4.371929000 | –1.252260000 |
| H | –1.606610000 | –3.316029000 | –3.213780000 |
| H | –3.356820000 | –3.003139000 | –3.307010000 |
| H | –2.732061000 | –4.661709000 | –3.521690000 |
| H | –0.660201000 | –4.654349000 | –1.196150000 |
| H | –1.827101000 | –5.954899000 | –1.523500000 |
| H | –1.770531000 | –5.190569000 | 0.088270000 |
| H | –5.661400000 | –1.049269000 | 1.225890000 |
| H | –5.386450000 | 1.048911000 | –0.108790000 |
| H | –4.378330000 | 1.742841000 | 1.184480000 |
| H | –6.091420000 | 1.383011000 | 1.496160000 |
| H | –4.319450000 | –1.417209000 | 3.298380000 |
| H | –5.452080000 | –0.051779000 | 3.499140000 |
| H | –3.721440000 | 0.257701000 | 3.215080000 |
| H | 5.646132000 | –1.035529000 | –1.252260000 |
| H | 4.360885000 | 1.755424000 | –1.196150000 |
| H | 6.070644000 | 1.395134000 | –1.523500000 |
| H | 5.380430000 | 1.061960000 | 0.088270000 |
| H | 3.675071000 | 0.266649000 | –3.213780000 |
| H | 4.279205000 | –1.405522000 | –3.307010000 |
| H | 5.403189000 | –0.035179000 | –3.521690000 |
| H | 3.739394000 | –4.378282000 | 1.225890000 |
| H | 1.784842000 | –5.189258000 | –0.108790000 |
| H | 0.679821000 | –4.663166000 | 1.184480000 |
| H | 1.847987000 | –5.966830000 | 1.496160000 |
| H | 3.387064000 | –3.032149000 | 3.298380000 |
| H | 2.770882000 | –4.695750000 | 3.499140000 |
| H | 1.637545000 | –3.351712000 | 3.215080000 |
| H | 1.922007000 | 5.427551000 | 1.225890000 |
| H | 0.932386000 | 4.449358000 | 3.298380000 |
| H | 2.681198000 | 4.747530000 | 3.499140000 |
| H | 2.083896000 | 3.094011000 | 3.215080000 |
| H | 3.601609000 | 4.140347000 | –0.108790000 |
| H | 3.698510000 | 2.920325000 | 1.184480000 |
| H | 4.243433000 | 4.583819000 | 1.496160000 |
| H | –1.926271000 | 5.407458000 | –1.252260000 |
| H | –2.068460000 | 3.049380000 | –3.213780000 |
| H | –0.922385000 | 4.408661000 | –3.307010000 |
| H | –2.671128000 | 4.696888000 | –3.521690000 |
| H | –3.700684000 | 2.898925000 | –1.196150000 |
| H | –4.243544000 | 4.559765000 | –1.523500000 |
| H | –3.609900000 | 4.128609000 | 0.088270000 |

$^t$Bu-**1**•

B3LYP

| | | | |
|---|---|---|---|
| C | –0.001279000 | 4.275620000 | 0.009791000 |
| C | 1.001111000 | 3.593809000 | 0.733291000 |
| C | 0.990680000 | 2.199129000 | 0.714451000 |
| C | 0.000000000 | 1.467990000 | 0.009371000 |
| C | –0.984000000 | 2.197800000 | –0.689889000 |
| C | –0.998009000 | 3.600930000 | –0.708869000 |
| C | 1.271316000 | –0.733995000 | 0.009371000 |
| C | 2.395351000 | –0.246731000 | –0.689889000 |
| C | 3.617502000 | –0.936164000 | –0.708869000 |
| C | 3.703435000 | –2.136702000 | 0.009791000 |
| C | 2.611775000 | –2.663892000 | 0.733291000 |
| C | 1.409162000 | –1.957519000 | 0.714451000 |
| C | –3.612886000 | –0.929917000 | 0.733291000 |
| C | –2.399842000 | –0.241610000 | 0.714451000 |
| C | –1.271316000 | –0.733995000 | 0.009371000 |
| C | –1.411351000 | –1.951069000 | –0.689889000 |
| C | –2.619492000 | –2.664766000 | –0.708869000 |
| C | –3.702156000 | –2.138917000 | 0.009791000 |
| C | 0.000000000 | 0.000000000 | 0.011481000 |
| C | 2.053511000 | 4.405449000 | 1.517091000 |
| C | –2.084849000 | 4.341531000 | –1.516549000 |

| | | | |
|---|---|---|---|
| C | 4.802300000 | –0.365233000 | –1.516549000 |
| C | 2.788475000 | –3.981117000 | 1.517091000 |
| C | –4.841986000 | –0.424332000 | 1.517091000 |
| C | –2.717451000 | –3.976297000 | –1.516549000 |
| C | 1.343952000 | 5.287149000 | 2.571361000 |
| C | 2.838302000 | 5.310159000 | 0.538831000 |
| C | 3.063341000 | 3.503788000 | 2.250091000 |
| C | –1.963719000 | 3.958761000 | –3.010089000 |
| C | –1.961718000 | 5.871961000 | –1.402749000 |
| C | –3.482559000 | 3.932631000 | –0.996529000 |
| C | 4.410247000 | –0.278750000 | –3.010089000 |
| C | 6.066126000 | –1.237083000 | –1.402749000 |
| C | 5.147038000 | 1.049669000 | –0.996529000 |
| C | 3.906830000 | –3.807471000 | 2.571361000 |
| C | 3.179581000 | –5.113121000 | 0.538831000 |
| C | 1.502699000 | –4.404825000 | 2.250091000 |
| C | –5.250781000 | –1.479678000 | 2.571361000 |
| C | –6.017883000 | –0.197038000 | 0.538831000 |
| C | –4.566040000 | 0.901037000 | 2.250091000 |
| C | –2.446528000 | –3.680011000 | –3.010089000 |
| C | –4.104408000 | –4.634878000 | –1.402749000 |
| C | –1.664479000 | –4.982300000 | –0.996529000 |
| H | 0.636602000 | 5.993069000 | 2.109381000 |
| H | 2.083422000 | 5.877969000 | 3.137511000 |
| H | 0.781231000 | 4.667999000 | 3.288861000 |
| H | 2.177682000 | 6.014129000 | 0.009531000 |
| H | 3.364581000 | 4.706998000 | –0.218749000 |
| H | 3.590432000 | 5.903948000 | 1.084731000 |
| H | 3.625541000 | 2.863548000 | 1.552341000 |
| H | 2.572141000 | 2.853349000 | 2.990871000 |
| H | 3.793211000 | 4.128158000 | 2.789531000 |
| H | –2.737609000 | 4.475221000 | –3.602399000 |
| H | –2.088919000 | 2.876401000 | –3.167129000 |
| H | –0.978489000 | 4.245930000 | –3.412049000 |
| H | –2.758948000 | 6.352111000 | –1.992009000 |
| H | –0.997888000 | 6.237870000 | –1.790449000 |
| H | –2.065778000 | 6.216851000 | –0.361739000 |
| H | –4.270999000 | 4.452122000 | –1.566419000 |
| H | –3.599849000 | 4.197801000 | 0.066941000 |
| H | –3.657369000 | 2.850251000 | –1.095689000 |
| H | 5.244459000 | 0.133228000 | –3.602399000 |
| H | 3.535496000 | 0.370857000 | –3.167129000 |
| H | 4.166328000 | –1.275569000 | –3.412049000 |
| H | 6.880563000 | –0.786736000 | –1.992009000 |
| H | 5.901098000 | –2.254739000 | –1.790449000 |
| H | 6.416840000 | –1.319409000 | –0.361739000 |
| H | 5.991150000 | 1.472733000 | –1.566419000 |
| H | 5.435327000 | 1.018660000 | 0.066941000 |
| H | 4.297075000 | 1.742249000 | –1.095689000 |
| H | 4.871849000 | –3.547848000 | 2.109381000 |
| H | 4.048759000 | –4.743281000 | 3.137511000 |
| H | 3.651990000 | –3.010566000 | 3.288861000 |
| H | 4.119547000 | –4.892992000 | 0.009531000 |
| H | 2.394089000 | –5.267312000 | –0.218749000 |
| H | 3.317753000 | –6.061379000 | 1.084731000 |
| H | 0.667135000 | –4.571584000 | 1.552341000 |
| H | 1.185002000 | –3.654213000 | 2.990871000 |
| H | 1.678484000 | –5.349096000 | 2.789531000 |
| H | –5.508451000 | –2.445221000 | 2.109381000 |
| H | –6.132181000 | –1.134688000 | 3.137511000 |
| H | –4.433222000 | –1.657434000 | 3.288861000 |
| H | –6.297229000 | –1.121137000 | 0.009531000 |
| H | –5.758671000 | 0.560314000 | –0.218749000 |
| H | –6.908185000 | 0.157431000 | 1.084731000 |
| H | –4.292676000 | 1.708036000 | 1.552341000 |
| H | –3.757143000 | 0.800865000 | 2.990871000 |
| H | –5.471695000 | 1.220938000 | 2.789531000 |
| H | –2.506851000 | –4.608449000 | –3.602399000 |
| H | –1.446576000 | –3.247258000 | –3.167129000 |
| H | –3.187839000 | –2.970361000 | –3.412049000 |
| H | –4.121615000 | –5.565374000 | –1.992009000 |
| H | –4.903210000 | –3.983131000 | –1.790449000 |
| H | –4.351062000 | –4.897442000 | –0.361739000 |
| H | –1.720151000 | –5.924854000 | –1.566419000 |
| H | –1.835478000 | –5.216461000 | 0.066941000 |
| H | –0.639705000 | –4.592500000 | –1.095689000 |
| H | 0.004922000 | 5.364160000 | 0.015221000 |
| H | 1.739070000 | 1.637519000 | 1.269751000 |
| H | –1.736930000 | 1.641270000 | –1.248609000 |
| H | 2.289847000 | 0.683590000 | –1.248609000 |
| H | 4.643038000 | –2.686342000 | 0.015221000 |
| H | 0.548598000 | –2.324838000 | 1.269751000 |
| H | –2.287668000 | 0.687319000 | 1.269751000 |
| H | –0.552917000 | –2.324861000 | –1.248609000 |
| H | –4.647959000 | –2.677818000 | 0.015221000 |

B3LYP-D3(BJ)

| | | | |
|---|---|---|---|
| C | –0.007040000 | 4.266470000 | 0.012270000 |
| C | 1.033940000 | 3.586060000 | 0.677550000 |
| C | 1.018740000 | 2.194200000 | 0.677010000 |
| C | –0.003150000 | 1.461180000 | 0.023450000 |
| C | –1.019820000 | 2.185630000 | –0.629180000 |
| C | –1.041340000 | 3.585840000 | –0.641220000 |
| C | 1.266994000 | –0.727862000 | 0.023450000 |
| C | 2.402721000 | –0.209625000 | –0.629180000 |
| C | 3.626099000 | –0.891093000 | –0.641220000 |
| C | 3.698392000 | –2.127138000 | 0.012270000 |
| C | 2.588649000 | –2.688448000 | 0.677550000 |
| C | 1.390863000 | –1.979355000 | 0.677010000 |
| C | –3.622589000 | –0.897612000 | 0.677550000 |
| C | –2.409603000 | –0.214845000 | 0.677010000 |
| C | –1.263844000 | –0.733318000 | 0.023450000 |
| C | –1.382901000 | –1.976005000 | –0.629180000 |
| C | –2.584759000 | –2.694747000 | –0.641220000 |
| C | –3.691351000 | –2.139332000 | 0.012270000 |
| C | 0.000000000 | 0.000000000 | 0.027630000 |
| C | 2.142040000 | 4.394990000 | 1.370010000 |
| C | –2.181490000 | 4.312290000 | –1.373060000 |
| C | 4.825298000 | –0.266919000 | –1.373060000 |
| C | 2.735153000 | –4.052556000 | 1.370010000 |
| C | –4.877193000 | –0.342434000 | 1.370010000 |
| C | –2.643808000 | –4.045371000 | –1.373060000 |
| C | 1.516440000 | 5.338960000 | 2.417870000 |
| C | 2.890230000 | 5.227790000 | 0.307320000 |
| C | 3.163170000 | 3.493530000 | 2.081390000 |
| C | –2.134340000 | 3.949600000 | –2.872210000 |
| C | –2.080820000 | 5.839510000 | –1.240660000 |
| C | –3.533610000 | 3.862450000 | –0.779190000 |
| C | 4.487624000 | –0.126407000 | –2.872210000 |
| C | 6.097574000 | –1.117712000 | –1.240660000 |
| C | 5.111785000 | 1.128971000 | –0.779190000 |
| C | 3.865455000 | –3.982756000 | 2.417870000 |
| C | 3.082284000 | –5.116908000 | 0.307320000 |
| C | 1.443901000 | –4.486151000 | 2.081390000 |
| C | –5.381895000 | –1.356204000 | 2.417870000 |
| C | –5.972514000 | –0.110882000 | 0.307320000 |
| C | –4.607071000 | 0.992621000 | 2.081390000 |
| C | –2.353284000 | –3.823193000 | –2.872210000 |
| C | –4.016754000 | –4.721798000 | –1.240660000 |
| C | –1.578175000 | –4.991421000 | –0.779190000 |
| H | 0.808330000 | 6.046970000 | 1.961370000 |
| H | 2.302090000 | 5.926970000 | 2.919960000 |
| H | 0.973250000 | 4.765090000 | 3.185540000 |
| H | 2.214270000 | 5.920710000 | –0.216490000 |
| H | 3.352380000 | 4.570330000 | –0.446440000 |
| H | 3.688060000 | 5.824990000 | 0.778610000 |
| H | 3.666970000 | 2.812930000 | 1.378730000 |
| H | 2.691680000 | 2.885290000 | 2.868810000 |
| H | 3.937080000 | 4.115590000 | 2.557330000 |
| H | –2.946930000 | 4.458910000 | –3.416050000 |
| H | –2.248080000 | 2.866720000 | –3.031590000 |
| H | –1.175470000 | 4.258220000 | –3.318310000 |
| H | –2.921160000 | 6.313150000 | –1.771490000 |
| H | –1.148660000 | 6.227530000 | –1.679670000 |
| H | –2.125350000 | 6.161320000 | –0.188500000 |
| H | –4.365950000 | 4.376760000 | –1.286920000 |
| H | –3.586890000 | 4.100460000 | 0.295210000 |
| H | –3.689570000 | 2.779050000 | –0.893080000 |
| H | 5.334995000 | 0.322661000 | –3.416050000 |
| H | 3.606693000 | 0.513534000 | –3.031590000 |
| H | 4.275462000 | –1.111123000 | –3.318310000 |
| H | 6.927928000 | –0.626776000 | –1.771490000 |
| H | 5.967529000 | –2.118996000 | –1.679670000 |
| H | 6.398535000 | –1.240053000 | –0.188500000 |
| H | 5.973361000 | 1.592644000 | –1.286920000 |
| H | 5.344548000 | 1.056108000 | 0.295210000 |
| H | 4.251513000 | 1.805736000 | –0.893080000 |
| H | 4.832665000 | –3.723519000 | 1.961370000 |
| H | 3.981862000 | –4.957153000 | 2.919960000 |
| H | 3.640064000 | –3.225404000 | 3.185540000 |
| H | 4.020350000 | –4.877969000 | –0.216490000 |
| H | 2.281832000 | –5.188411000 | –0.446440000 |
| H | 3.200559000 | –6.106449000 | 0.778610000 |
| H | 0.602584000 | –4.582154000 | 1.378730000 |
| H | 1.152895000 | –3.773708000 | 2.868810000 |
| H | 1.595666000 | –5.467406000 | 2.557330000 |
| H | –5.640995000 | –2.323451000 | 1.961370000 |
| H | –6.283952000 | –0.969817000 | 2.919960000 |
| H | –4.613314000 | –1.539686000 | 3.185540000 |
| H | –6.234620000 | –1.042741000 | –0.216490000 |
| H | –5.634212000 | 0.618081000 | –0.446440000 |
| H | –6.888619000 | 0.281459000 | 0.778610000 |

| | | | |
|---|---|---|---|
| H | –4.269554000 | 1.769224000 | 1.378730000 |
| H | –3.844574000 | 0.888418000 | 2.868810000 |
| H | –5.532745000 | 1.351816000 | 2.557330000 |
| H | –2.388065000 | –4.781571000 | –3.416050000 |
| H | –1.358612000 | –3.380255000 | –3.031590000 |
| H | –3.099992000 | –3.147097000 | –3.318310000 |
| H | –4.006768000 | –5.686374000 | –1.771490000 |
| H | –4.818869000 | –4.108534000 | –1.679670000 |
| H | –4.273185000 | –4.921267000 | –0.188500000 |
| H | –1.607411000 | –5.969404000 | –1.286920000 |
| H | –1.757658000 | –5.156568000 | 0.295210000 |
| H | –0.561943000 | –4.584787000 | –0.893080000 |
| H | 0.000000000 | 5.354250000 | 0.011050000 |
| H | 1.790950000 | 1.636410000 | 1.200250000 |
| H | –1.795870000 | 1.627840000 | –1.151660000 |
| H | 2.307686000 | 0.741349000 | –1.151660000 |
| H | 4.636917000 | –2.677125000 | 0.011050000 |
| H | 0.521698000 | –2.369213000 | 1.200250000 |
| H | –2.312648000 | 0.732803000 | 1.200250000 |
| H | –0.511816000 | –2.369189000 | –1.151660000 |
| H | –4.636917000 | –2.677125000 | 0.011050000 |

M06-2X

| | | | |
|---|---|---|---|
| C | –0.005822000 | 4.260544000 | 0.009526000 |
| C | 1.030142000 | 3.584626000 | 0.682117000 |
| C | 1.018836000 | 2.194720000 | 0.684325000 |
| C | –0.000463000 | 1.462640000 | 0.032463000 |
| C | –1.012067000 | 2.179540000 | –0.625502000 |
| C | –1.032449000 | 3.578644000 | –0.646101000 |
| C | 1.266915000 | –0.730919000 | 0.032463000 |
| C | 2.393571000 | –0.213294000 | –0.625502000 |
| C | 3.615421000 | –0.895195000 | –0.646101000 |
| C | 3.692650000 | –2.125230000 | 0.009526000 |
| C | 2.589306000 | –2.684442000 | 0.682117000 |
| C | 1.391265000 | –1.979698000 | 0.684325000 |
| C | –3.619448000 | –0.900184000 | 0.682117000 |
| C | –2.410101000 | –0.215022000 | 0.684325000 |
| C | –1.266452000 | –0.731721000 | 0.032463000 |
| C | –1.381503000 | –1.966246000 | –0.625502000 |
| C | –2.582972000 | –2.683449000 | –0.646101000 |
| C | –3.686828000 | –2.135314000 | 0.009526000 |
| C | 0.000000000 | 0.000000000 | 0.041647000 |
| C | 2.122726000 | 4.399095000 | 1.383030000 |
| C | –2.162913000 | 4.297120000 | –1.390671000 |
| C | 4.802872000 | –0.275422000 | –1.390671000 |
| C | 2.748365000 | –4.037882000 | 1.383030000 |
| C | –4.871091000 | –0.361213000 | 1.383030000 |
| C | –2.639959000 | –4.021698000 | –1.390671000 |
| C | 1.482054000 | 5.313347000 | 2.439768000 |
| C | 2.854787000 | 5.258299000 | 0.339362000 |
| C | 3.151352000 | 3.504506000 | 2.079860000 |
| C | –2.100511000 | 3.926278000 | –2.881066000 |
| C | –2.061914000 | 5.819401000 | –1.264539000 |
| C | –3.515203000 | 3.850686000 | –0.810592000 |
| C | 4.450512000 | –0.144043000 | –2.881066000 |
| C | 6.070706000 | –1.124031000 | –1.264539000 |
| C | 5.092393000 | 1.118912000 | –0.810592000 |
| C | 3.860466000 | –3.940170000 | 2.439768000 |
| C | 3.126427000 | –5.101468000 | 0.339362000 |
| C | 1.459315000 | –4.481404000 | 2.079860000 |
| C | –5.342520000 | –1.373177000 | 2.439768000 |
| C | –5.981214000 | –0.156831000 | 0.339362000 |
| C | –4.610667000 | 0.976898000 | 2.079860000 |
| C | –2.350001000 | –3.782235000 | –2.881066000 |
| C | –4.008792000 | –4.695370000 | –1.264539000 |
| C | –1.577190000 | –4.969598000 | –0.810592000 |
| H | 0.760203000 | 6.011172000 | 1.990521000 |
| H | 2.258600000 | 5.908838000 | 2.944892000 |
| H | 0.953839000 | 4.717844000 | 3.199700000 |
| H | 2.166301000 | 5.946441000 | –0.172924000 |
| H | 3.329871000 | 4.620438000 | –0.421989000 |
| H | 3.638495000 | 5.860241000 | 0.825400000 |
| H | 3.658847000 | 2.838216000 | 1.366085000 |
| H | 2.684881000 | 2.884005000 | 2.859518000 |
| H | 3.917169000 | 4.131844000 | 2.559818000 |
| H | –2.906276000 | 4.435349000 | –3.432806000 |
| H | –2.216319000 | 2.842988000 | –3.031242000 |
| H | –1.136368000 | 4.229834000 | –3.316346000 |
| H | –2.899080000 | 6.287440000 | –1.803165000 |
| H | –1.126738000 | 6.201134000 | –1.700884000 |
| H | –2.112381000 | 6.142019000 | –0.213683000 |
| H | –4.339517000 | 4.358574000 | –1.335361000 |
| H | –3.580169000 | 4.100517000 | 0.259730000 |
| H | –3.663831000 | 2.765467000 | –0.919203000 |
| H | 5.294263000 | 0.299234000 | –3.432806000 |
| H | 3.570259000 | 0.497895000 | –3.031242000 |
| H | 4.231328000 | –1.130793000 | –3.316346000 |
| H | 6.894623000 | –0.633043000 | –1.803165000 |
| H | 5.933709000 | –2.124783000 | –1.700884000 |
| H | 6.375335000 | –1.241634000 | –0.213683000 |
| H | 5.944394000 | 1.578845000 | –1.335361000 |
| H | 5.341236000 | 1.050259000 | 0.259730000 |
| H | 4.226880000 | 1.790237000 | –0.919203000 |
| H | 4.825726000 | –3.663941000 | 1.990521000 |
| H | 3.987904000 | –4.910424000 | 2.944892000 |
| H | 3.608853000 | –3.184971000 | 3.199700000 |
| H | 4.066618000 | –4.849292000 | –0.172924000 |
| H | 2.336481000 | –5.193972000 | –0.421989000 |
| H | 3.255870000 | –6.081150000 | 0.825400000 |
| H | 0.628544000 | –4.587762000 | 1.366085000 |
| H | 1.155181000 | –3.767178000 | 2.859518000 |
| H | 1.619697000 | –5.458290000 | 2.559818000 |
| H | –5.585929000 | –2.347231000 | 1.990521000 |
| H | –6.246504000 | –0.998414000 | 2.944892000 |
| H | –4.562692000 | –1.532873000 | 3.199700000 |
| H | –6.232919000 | –1.097149000 | –0.172924000 |
| H | –5.666352000 | 0.573534000 | –0.421989000 |
| H | –6.894365000 | 0.220909000 | 0.825400000 |
| H | –4.287391000 | 1.749546000 | 1.366085000 |
| H | –3.840062000 | 0.883173000 | 2.859518000 |
| H | –5.536866000 | 1.326446000 | 2.559818000 |
| H | –2.387987000 | –4.734583000 | –3.432806000 |
| H | –1.353940000 | –3.340883000 | –3.031242000 |
| H | –3.094960000 | –3.099041000 | –3.316346000 |
| H | –3.995543000 | –5.654397000 | –1.803165000 |
| H | –4.806971000 | –4.076351000 | –1.700884000 |
| H | –4.262954000 | –4.900385000 | –0.213683000 |
| H | –1.604877000 | –5.937419000 | –1.335361000 |
| H | –1.761067000 | –5.150776000 | 0.259730000 |
| H | –0.563049000 | –4.555704000 | –0.919203000 |
| H | 0.000000000 | 5.350093000 | 0.006180000 |
| H | 1.792504000 | 1.636100000 | 1.208937000 |
| H | –1.787725000 | 1.617467000 | –1.148363000 |
| H | 2.294630000 | 0.739482000 | –1.148363000 |
| H | 4.633316000 | –2.675046000 | 0.006180000 |
| H | 0.520652000 | –2.370404000 | 1.208937000 |
| H | –2.313156000 | 0.734304000 | 1.208937000 |
| H | –0.506905000 | –2.356949000 | –1.148363000 |
| H | –4.633316000 | –2.675046000 | 0.006180000 |

Cy-**1**•

B3LYP

| | | | |
|---|---|---|---|
| C | 1.452980000 | –0.201320000 | 0.091051000 |
| C | 0.000000000 | 0.000000000 | 0.100661000 |
| C | –0.552142000 | 1.358978000 | 0.091051000 |
| C | –1.742850000 | 1.662010000 | –0.613599000 |
| C | –2.271560000 | 2.955460000 | –0.654679000 |
| C | –3.532300000 | 3.240100000 | –1.457699000 |
| C | –5.963870000 | 4.005050000 | –1.413059000 |
| C | –5.697930000 | 4.957630000 | –2.586459000 |
| C | –4.542840000 | 4.453700000 | –3.462129000 |
| C | –4.695400000 | 3.759890000 | –0.583749000 |
| C | –3.273580000 | 4.207270000 | –2.634949000 |
| C | –1.601430000 | 3.977000000 | 0.040701000 |
| C | –0.420720000 | 3.723550000 | 0.760491000 |
| C | 0.300250000 | 4.833330000 | 1.510661000 |
| C | –0.565390000 | 5.473360000 | 2.618551000 |
| C | 0.196040000 | 6.571060000 | 3.375761000 |
| C | 0.740630000 | 7.643500000 | 2.422501000 |
| C | 1.603650000 | 7.019400000 | 1.318611000 |
| C | 0.843970000 | 5.923070000 | 0.559251000 |
| C | 0.088930000 | 2.421480000 | 0.771531000 |
| C | –0.900839000 | –1.157657000 | 0.091051000 |
| C | –2.141528000 | –1.133724000 | 0.771531000 |
| C | –3.014329000 | –2.226129000 | 0.760491000 |
| C | –4.335911000 | –2.156641000 | 1.510661000 |
| C | –6.880803000 | –2.120898000 | 1.318611000 |
| C | –6.989780000 | –3.180345000 | 2.422501000 |
| C | –5.788725000 | –3.115754000 | 3.375761000 |
| C | –5.551514000 | –2.230635000 | 0.559251000 |
| C | –4.457374000 | –3.226322000 | 2.618551000 |
| C | –2.643468000 | –3.375379000 | 0.040701000 |
| C | –1.423723000 | –3.444958000 | –0.654679000 |
| C | –1.039859000 | –4.679111000 | –1.457699000 |
| C | –2.006812000 | –4.938638000 | –2.634949000 |
| C | –1.585597000 | –6.161065000 | –3.462129000 |
| C | –0.486540000 | –7.167388000 | –1.413059000 |
| C | –1.444468000 | –7.413367000 | –2.586459000 |
| C | –0.908460000 | –5.946280000 | –0.583749000 |
| C | –0.567918000 | –2.340357000 | –0.613599000 |
| C | 2.052598000 | –1.287755000 | 0.771531000 |
| C | 3.435049000 | –1.497421000 | 0.760491000 |
| C | 4.035661000 | –2.676689000 | 1.510661000 |

| | | | |
|---|---|---|---|
| C | 5.277153000 | −4.898501000 | 1.318611000 |
| C | 6.249150000 | −4.463154000 | 2.422501000 |
| C | 5.592685000 | −3.455305000 | 3.375761000 |
| C | 4.707544000 | −3.692434000 | 0.559251000 |
| C | 5.022763000 | −2.247038000 | 2.618551000 |
| C | 4.244898000 | −0.601621000 | 0.040701000 |
| C | 3.695283000 | 0.489499000 | −0.654679000 |
| C | 4.572159000 | 1.439012000 | −1.457699000 |
| C | 6.450410000 | 3.162338000 | −1.413059000 |
| C | 7.142398000 | 2.455737000 | −2.586459000 |
| C | 6.128437000 | 1.707365000 | −3.462129000 |
| C | 5.603860000 | 2.186391000 | −0.583749000 |
| C | 5.280392000 | 0.731369000 | −2.634949000 |
| C | 2.310768000 | 0.678348000 | −0.613599000 |
| H | −2.247620000 | 0.866150000 | −1.164999000 |
| H | −3.859350000 | 2.278550000 | −1.894529000 |
| H | −6.332730000 | 3.038740000 | −1.806249000 |
| H | −6.765130000 | 4.404390000 | −0.768279000 |
| H | −6.611580000 | 5.083170000 | −3.192059000 |
| H | −5.444700000 | 5.960110000 | −2.191559000 |
| H | −4.844990000 | 3.509210000 | −3.952879000 |
| H | −4.332060000 | 5.172500000 | −4.272049000 |
| H | −4.391810000 | 4.705080000 | −0.096499000 |
| H | −4.898980000 | 3.041730000 | 0.228311000 |
| H | −2.903380000 | 5.171190000 | −2.239029000 |
| H | −2.470230000 | 3.804200000 | −3.274239000 |
| H | −2.005590000 | 4.991780000 | 0.018081000 |
| H | 1.173600000 | 4.372080000 | 2.007351000 |
| H | −0.909040000 | 4.691840000 | 3.317001000 |
| H | −1.475170000 | 5.908550000 | 2.165221000 |
| H | 1.039280000 | 6.113430000 | 3.927051000 |
| H | −0.459760000 | 7.028980000 | 4.135771000 |
| H | −0.107050000 | 8.183820000 | 1.959531000 |
| H | 1.320730000 | 8.395620000 | 2.983661000 |
| H | 1.947920000 | 7.795770000 | 0.614401000 |
| H | 2.514150000 | 6.583490000 | 1.771651000 |
| H | 0.000000000 | 6.378570000 | 0.008521000 |
| H | 1.496050000 | 5.458870000 | −0.199839000 |
| H | 0.998990000 | 2.212980000 | 1.337651000 |
| H | −2.415992000 | −0.241339000 | 1.337651000 |
| H | −4.373132000 | −1.169672000 | 2.007351000 |
| H | −7.725295000 | −2.210937000 | 0.614401000 |
| H | −6.958544000 | −1.114427000 | 1.771651000 |
| H | −7.033871000 | −4.184618000 | 1.959531000 |
| H | −7.931185000 | −3.054024000 | 2.983661000 |
| H | −5.814025000 | −2.156672000 | 3.927051000 |
| H | −5.857395000 | −3.912654000 | 4.135771000 |
| H | −5.524003000 | −3.189285000 | 0.008521000 |
| H | −5.475545000 | −1.433818000 | −0.199839000 |
| H | −3.608732000 | −3.133172000 | 3.317001000 |
| H | −4.379369000 | −4.231810000 | 2.165221000 |
| H | −3.320213000 | −4.232782000 | 0.018081000 |
| H | −0.043607000 | −4.481570000 | −1.894529000 |
| H | −3.026692000 | −5.099996000 | −2.239029000 |
| H | −2.059419000 | −4.041382000 | −3.274239000 |
| H | −0.616570000 | −5.950489000 | −3.952879000 |
| H | −2.313486000 | −6.337924000 | −4.272049000 |
| H | 0.534739000 | −7.003675000 | −1.806249000 |
| H | −0.431748000 | −8.060969000 | −0.768279000 |
| H | −1.096364000 | −8.267381000 | −3.192059000 |
| H | −2.439256000 | −7.695303000 | −2.191559000 |
| H | −1.878813000 | −6.155959000 | −0.096499000 |
| H | −0.184725000 | −5.763506000 | 0.228311000 |
| H | 0.373702000 | −2.379571000 | −1.164999000 |
| H | 1.417002000 | −1.971641000 | 1.337651000 |
| H | 3.199532000 | −3.202407000 | 2.007351000 |
| H | 5.777375000 | −5.584833000 | 0.614401000 |
| H | 4.444394000 | −5.469063000 | 1.771651000 |
| H | 7.140921000 | −3.999202000 | 1.959531000 |
| H | 6.610455000 | −5.341596000 | 2.983661000 |
| H | 4.774745000 | −3.956758000 | 3.927051000 |
| H | 6.317155000 | −3.116326000 | 4.135771000 |
| H | 5.524003000 | −3.189285000 | 0.008521000 |
| H | 3.979495000 | −4.025052000 | −0.199839000 |
| H | 4.517772000 | −1.558668000 | 3.317001000 |
| H | 5.854539000 | −1.676740000 | 2.165221000 |
| H | 5.325803000 | −0.758998000 | 0.018081000 |
| H | 3.902957000 | 2.203020000 | −1.894529000 |
| H | 5.797991000 | 3.964935000 | −1.806249000 |
| H | 7.196878000 | 3.656580000 | −0.768279000 |
| H | 7.707944000 | 3.184211000 | −3.192059000 |
| H | 7.883956000 | 1.735194000 | −2.191559000 |
| H | 5.461560000 | 2.441280000 | −3.952879000 |
| H | 6.645546000 | 1.165424000 | −4.272049000 |
| H | 6.270623000 | 1.450879000 | −0.096499000 |
| H | 5.083705000 | 2.721776000 | 0.228311000 |
| H | 5.930072000 | −0.071194000 | −2.239029000 |
| H | 4.529649000 | 0.237182000 | −3.274239000 |
| H | 1.873918000 | 1.513421000 | −1.164999000 |

B3LYP-D3(BJ)

| | | | |
|---|---|---|---|
| C | −0.925130000 | −1.130601000 | −0.063005000 |
| C | 0.000000000 | 0.000000000 | −0.069149000 |
| C | −0.516564000 | 1.366487000 | −0.063005000 |
| C | 0.164733000 | 2.405312000 | 0.612384000 |
| C | −0.325464000 | 3.711659000 | 0.637819000 |
| C | 0.416776000 | 4.792576000 | 1.398481000 |
| C | 1.646295000 | 7.015003000 | 1.264345000 |
| C | 0.812282000 | 7.555319000 | 2.431447000 |
| C | 0.350009000 | 6.420745000 | 3.352581000 |
| C | 0.882520000 | 5.940714000 | 0.481777000 |
| C | −0.414651000 | 5.345723000 | 2.572812000 |
| C | −1.524317000 | 3.996121000 | −0.035399000 |
| C | −2.230459000 | 2.996490000 | −0.723109000 |
| C | −3.525889000 | 3.311124000 | −1.443721000 |
| C | −3.344051000 | 4.365210000 | −2.553134000 |
| C | −4.661105000 | 4.648508000 | −3.283719000 |
| C | −5.761998000 | 5.075915000 | −2.306183000 |
| C | −5.952727000 | 4.033608000 | −1.198296000 |
| C | −4.636498000 | 3.748005000 | −0.467336000 |
| C | −1.720869000 | 1.697136000 | −0.727137000 |
| C | 1.441694000 | −0.235885000 | −0.063005000 |
| C | 2.330197000 | 0.641749000 | −0.727137000 |
| C | 3.710266000 | 0.433389000 | −0.723109000 |
| C | 4.630462000 | 1.397947000 | −1.443721000 |
| C | 6.469570000 | 3.138409000 | −1.198296000 |
| C | 7.276870000 | 2.452080000 | −2.306183000 |
| C | 6.356279000 | 1.712381000 | −3.283719000 |
| C | 5.564116000 | 2.141322000 | −0.467336000 |
| C | 5.452408000 | 0.713428000 | −2.553134000 |
| C | 4.222901000 | −0.677963000 | −0.035399000 |
| C | 3.377123000 | −1.573970000 | 0.637819000 |
| C | 3.942105000 | −2.757227000 | 1.398481000 |
| C | 4.836857000 | −2.313763000 | 2.572812000 |
| C | 5.385524000 | −3.513489000 | 3.352581000 |
| C | 5.252024000 | −4.933235000 | 1.264345000 |
| C | 6.136957000 | −4.481116000 | 2.431447000 |
| C | 4.703549000 | −3.734641000 | 0.481777000 |
| C | 2.000695000 | −1.345319000 | 0.612384000 |
| C | −0.609328000 | −2.338884000 | −0.727137000 |
| C | −1.479807000 | −3.429880000 | −0.723109000 |
| C | −1.104574000 | −4.709071000 | −1.443721000 |
| C | −0.516843000 | −7.172016000 | −1.198296000 |
| C | −1.514872000 | −7.527994000 | −2.306183000 |
| C | −1.695174000 | −6.360889000 | −3.283719000 |
| C | −0.927618000 | −5.889327000 | −0.467336000 |
| C | −2.108357000 | −5.078638000 | −2.553134000 |
| C | −2.698583000 | −3.318158000 | −0.035399000 |
| C | −3.051659000 | −2.137689000 | 0.637819000 |
| C | −4.358881000 | −2.035349000 | 1.398481000 |
| C | −6.898319000 | −2.081769000 | 1.264345000 |
| C | −6.949239000 | −3.074202000 | 2.431447000 |
| C | −5.735533000 | −2.907256000 | 3.352581000 |
| C | −5.586069000 | −2.206073000 | 0.481777000 |
| C | −4.422206000 | −3.031959000 | 2.572812000 |
| C | −2.165428000 | −1.059993000 | 0.612384000 |
| H | 1.084803000 | 2.172732000 | 1.150737000 |
| H | 1.322685000 | 4.328403000 | 1.828072000 |
| H | 2.582328000 | 6.578125000 | 1.659559000 |
| H | 1.942861000 | 7.835644000 | 0.590136000 |
| H | 1.389562000 | 8.301975000 | 3.001724000 |
| H | −0.074646000 | 8.080908000 | 2.030203000 |
| H | 1.234142000 | 5.960719000 | 3.832053000 |
| H | −0.278355000 | 6.817566000 | 4.167224000 |
| H | 0.000000000 | 6.399372000 | 0.000071000 |
| H | 1.506800000 | 5.533154000 | −0.330453000 |
| H | −1.351898000 | 5.778416000 | 2.178207000 |
| H | −0.707814000 | 4.516891000 | 3.237916000 |
| H | −1.917653000 | 5.014482000 | −0.020087000 |
| H | −3.865844000 | 2.378753000 | −1.929286000 |
| H | −2.571239000 | 4.024916000 | −3.261839000 |
| H | −2.968321000 | 5.302178000 | −2.103500000 |
| H | −4.983873000 | 3.734049000 | −3.815396000 |
| H | −4.508326000 | 5.422586000 | −4.054013000 |
| H | −5.484475000 | 6.044316000 | −1.848896000 |
| H | −6.710017000 | 5.241890000 | −2.844405000 |
| H | −6.719749000 | 4.369680000 | −0.480850000 |
| H | −6.329150000 | 3.094740000 | −1.645568000 |
| H | −4.308535000 | 4.658835000 | 0.065970000 |
| H | −4.779108000 | 2.969186000 | 0.299880000 |
| H | −2.255913000 | 0.918326000 | −1.272799000 |
| H | 1.923250000 | 1.494515000 | −1.272799000 |
| H | 3.992983000 | 2.158543000 | −1.929286000 |
| H | 7.144128000 | 3.634633000 | −0.480850000 |
| H | 5.844698000 | 3.933834000 | −1.645568000 |
| H | 7.976768000 | 1.727537000 | −1.848896000 |

| | | | |
|---|---|---|---|
| H | 7.894619000 | 3.190101000 | –2.844405000 |
| H | 5.725718000 | 2.449137000 | –3.815396000 |
| H | 6.950260000 | 1.193032000 | –4.054013000 |
| H | 6.188937000 | 1.401883000 | 0.065970000 |
| H | 4.960945000 | 2.654236000 | 0.299880000 |
| H | 4.771299000 | 0.214300000 | –3.261839000 |
| H | 6.075982000 | –0.080448000 | –2.103500000 |
| H | 5.301495000 | –0.846505000 | –0.020087000 |
| H | 3.087164000 | –3.309680000 | 1.828072000 |
| H | 5.680204000 | –1.718430000 | 2.178207000 |
| H | 4.265650000 | –1.645460000 | 3.237916000 |
| H | 4.545063000 | –4.049158000 | 3.832053000 |
| H | 6.043363000 | –3.167721000 | 4.167224000 |
| H | 4.405660000 | –5.525424000 | 1.659559000 |
| H | 5.814436000 | –5.600389000 | 0.590136000 |
| H | 6.494941000 | –5.354384000 | 3.001724000 |
| H | 7.035594000 | –3.975808000 | 2.030203000 |
| H | 5.542019000 | –3.199686000 | 0.000071000 |
| H | 4.038452000 | –4.071504000 | –0.330453000 |
| H | 1.339240000 | –2.025833000 | 1.150737000 |
| H | 0.332663000 | –2.412841000 | –1.272799000 |
| H | –0.127139000 | –4.537296000 | –1.929286000 |
| H | –0.424379000 | –8.004313000 | –0.480850000 |
| H | 0.484452000 | –7.028574000 | –1.645568000 |
| H | –2.492293000 | –7.771853000 | –1.848896000 |
| H | –1.184601000 | –8.431991000 | –2.844405000 |
| H | –0.741844000 | –6.183185000 | –3.815396000 |
| H | –2.441934000 | –6.615618000 | –4.054013000 |
| H | –1.880402000 | –6.060718000 | 0.065970000 |
| H | –0.181837000 | –5.623422000 | 0.299880000 |
| H | –2.200060000 | –4.239216000 | –3.261839000 |
| H | –3.107660000 | –5.221731000 | –2.103500000 |
| H | –3.383843000 | –4.167977000 | –0.020087000 |
| H | –4.409849000 | –1.018722000 | 1.828072000 |
| H | –6.987988000 | –1.052701000 | 1.659559000 |
| H | –7.757298000 | –2.235255000 | 0.590136000 |
| H | –7.884503000 | –2.947592000 | 3.001724000 |
| H | –6.960948000 | –4.105099000 | 2.030203000 |
| H | –5.779205000 | –1.911561000 | 3.832053000 |
| H | –5.765008000 | –3.649845000 | 4.167224000 |
| H | –5.542019000 | –3.199686000 | 0.000071000 |
| H | –5.545252000 | –1.461650000 | –0.330453000 |
| H | –4.328306000 | –4.059986000 | 2.178207000 |
| H | –3.557835000 | –2.871431000 | 3.237916000 |
| H | –2.424043000 | –0.146899000 | 1.150737000 |

M06-2X

| | | | |
|---|---|---|---|
| C | 0.931630000 | –1.127940000 | 0.051840000 |
| C | 0.000000000 | 0.000000000 | 0.054160000 |
| C | 0.511009000 | 1.370786000 | 0.051840000 |
| C | –0.174830000 | 2.403330000 | –0.620370000 |
| C | 0.306660000 | 3.711100000 | –0.638290000 |
| C | –0.430180000 | 4.790090000 | –1.403330000 |
| C | –1.640620000 | 7.012540000 | –1.284270000 |
| C | –0.794630000 | 7.543290000 | –2.441350000 |
| C | –0.345690000 | 6.403720000 | –3.355910000 |
| C | –0.887210000 | 5.941370000 | –0.494590000 |
| C | 0.410290000 | 5.332650000 | –2.569610000 |
| C | 1.496590000 | 4.000120000 | 0.042260000 |
| C | 2.205130000 | 3.004540000 | 0.726750000 |
| C | 3.492900000 | 3.328050000 | 1.453710000 |
| C | 3.297330000 | 4.380240000 | 2.555310000 |
| C | 4.606960000 | 4.666440000 | 3.290840000 |
| C | 5.701030000 | 5.107940000 | 2.318010000 |
| C | 5.905400000 | 4.066430000 | 1.217880000 |
| C | 4.596980000 | 3.776380000 | 0.482700000 |
| C | 1.707620000 | 1.702510000 | 0.719000000 |
| C | –1.442640000 | –0.242846000 | 0.051840000 |
| C | –2.328227000 | 0.627587000 | 0.719000000 |
| C | –3.704573000 | 0.407429000 | 0.726750000 |
| C | –4.628626000 | 1.360915000 | 1.453710000 |
| C | –6.474332000 | 3.081011000 | 1.217880000 |
| C | –7.274121000 | 2.383267000 | 2.318010000 |
| C | –6.344736000 | 1.656524000 | 3.290840000 |
| C | –5.568931000 | 2.092911000 | 0.482700000 |
| C | –5.442064000 | 0.665452000 | 2.555310000 |
| C | –4.212501000 | –0.703975000 | 0.042260000 |
| C | –3.367237000 | –1.589975000 | –0.638290000 |
| C | –3.933250000 | –2.767592000 | –1.403330000 |
| C | –4.823355000 | –2.311003000 | –2.569610000 |
| C | –5.372939000 | –3.501236000 | –3.355910000 |
| C | –5.252728000 | –4.927089000 | –1.284270000 |
| C | –6.135366000 | –4.459815000 | –2.441350000 |
| C | –4.701772000 | –3.739031000 | –0.494590000 |
| C | –1.993930000 | –1.353072000 | –0.620370000 |
| C | 0.620607000 | –2.330098000 | 0.719000000 |
| C | 1.499443000 | –3.411969000 | 0.726750000 |
| C | 1.135726000 | –4.688965000 | 1.453710000 |
| C | 0.568932000 | –7.147442000 | 1.217880000 |
| C | 1.573091000 | –7.491207000 | 2.318010000 |
| C | 1.737776000 | –6.322965000 | 3.290840000 |
| C | 0.971951000 | –5.869292000 | 0.482700000 |
| C | 2.144734000 | –5.045692000 | 2.555310000 |
| C | 2.715911000 | –3.296145000 | 0.042260000 |
| C | 3.060577000 | –2.121126000 | –0.638290000 |
| C | 4.363430000 | –2.022498000 | –1.403330000 |
| C | 6.893348000 | –2.085451000 | –1.284270000 |
| C | 6.929996000 | –3.083475000 | –2.441350000 |
| C | 5.718629000 | –2.902484000 | –3.355910000 |
| C | 5.588982000 | –2.202339000 | –0.494590000 |
| C | 4.413065000 | –3.021647000 | –2.569610000 |
| C | 2.168760000 | –1.050258000 | –0.620370000 |
| H | –1.093670000 | 2.165900000 | –1.160750000 |
| H | –1.336230000 | 4.328900000 | –1.834890000 |
| H | –2.570180000 | 6.574410000 | –1.688870000 |
| H | –1.941280000 | 7.834130000 | –0.616550000 |
| H | –1.356630000 | 8.297200000 | –3.013250000 |
| H | 0.097210000 | 8.051470000 | –2.033300000 |
| H | –1.234930000 | 5.946520000 | –3.824970000 |
| H | 0.282180000 | 6.788840000 | –4.173630000 |
| H | 0.000000000 | 6.397260000 | –0.019640000 |
| H | –1.513710000 | 5.543300000 | 0.318840000 |
| H | 1.342320000 | 5.767530000 | –2.165980000 |
| H | 0.707060000 | 4.501420000 | –3.227850000 |
| H | 1.883470000 | 5.022560000 | 0.034160000 |
| H | 3.838250000 | 2.399210000 | 1.941030000 |
| H | 2.521090000 | 4.040820000 | 3.258350000 |
| H | 2.927550000 | 5.315330000 | 2.098210000 |
| H | 4.935720000 | 3.749290000 | 3.810680000 |
| H | 4.446950000 | 5.431100000 | 4.066060000 |
| H | 5.406920000 | 6.067430000 | 1.856530000 |
| H | 6.643820000 | 5.288880000 | 2.856210000 |
| H | 6.674150000 | 4.401700000 | 0.505370000 |
| H | 6.277340000 | 3.131020000 | 1.671980000 |
| H | 4.261090000 | 4.689800000 | –0.040570000 |
| H | 4.746110000 | 3.004500000 | –0.288550000 |
| H | 2.248020000 | 0.922330000 | 1.259630000 |
| H | –1.922772000 | 1.485678000 | 1.259630000 |
| H | –3.996902000 | 2.124417000 | 1.941030000 |
| H | –7.149059000 | 3.579133000 | 0.505370000 |
| H | –5.850213000 | 3.870826000 | 1.671980000 |
| H | –7.958009000 | 1.648815000 | 1.856530000 |
| H | –7.902215000 | 3.109277000 | 2.856210000 |
| H | –5.714841000 | 2.399814000 | 3.810680000 |
| H | –6.926946000 | 1.135622000 | 4.066060000 |
| H | –6.192031000 | 1.345312000 | –0.040570000 |
| H | –4.975029000 | 2.608002000 | –0.288550000 |
| H | –4.759998000 | 0.162918000 | 3.258350000 |
| H | –6.066986000 | –0.122332000 | 2.098210000 |
| H | –5.291400000 | –0.880147000 | 0.034160000 |
| H | –3.080822000 | –3.321659000 | –1.834890000 |
| H | –5.665988000 | –1.721282000 | –2.165980000 |
| H | –4.251874000 | –1.638378000 | –3.227850000 |
| H | –4.532372000 | –4.042741000 | –3.824970000 |
| H | –6.020398000 | –3.150045000 | –4.173630000 |
| H | –4.408516000 | –5.513046000 | –1.688870000 |
| H | –5.813915000 | –5.598263000 | –0.616550000 |
| H | –6.507271000 | –5.323476000 | –3.013250000 |
| H | –7.021383000 | –3.941549000 | –2.033300000 |
| H | –5.540190000 | –3.198630000 | –0.019640000 |
| H | –4.043784000 | –4.082561000 | 0.318840000 |
| H | –1.328890000 | –2.030096000 | –1.160750000 |
| H | –0.325249000 | –2.408008000 | 1.259630000 |
| H | 0.158652000 | –4.523627000 | 1.941030000 |
| H | 0.474909000 | –7.980834000 | 0.505370000 |
| H | –0.427127000 | –7.001846000 | 1.671980000 |
| H | 2.551089000 | –7.716245000 | 1.856530000 |
| H | 1.258395000 | –8.398157000 | 2.856210000 |
| H | 0.779121000 | –6.149104000 | 3.810680000 |
| H | 2.479996000 | –6.566722000 | 4.066060000 |
| H | 1.930941000 | –6.035112000 | –0.040570000 |
| H | 0.228919000 | –5.612502000 | –0.288550000 |
| H | 2.238908000 | –4.203738000 | 3.258350000 |
| H | 3.139436000 | –5.192998000 | 2.098210000 |
| H | 3.407930000 | –4.142413000 | 0.034160000 |
| H | 4.417052000 | –1.007241000 | –1.834890000 |
| H | 6.978696000 | –1.061364000 | –1.688870000 |
| H | 7.755196000 | –2.235867000 | –0.616550000 |
| H | 7.863901000 | –2.973724000 | –3.013250000 |
| H | 6.924173000 | –4.109921000 | –2.033300000 |
| H | 5.767302000 | –1.903779000 | –3.824970000 |
| H | 5.738218000 | –3.638795000 | –4.173630000 |
| H | 5.540190000 | –3.198630000 | –0.019640000 |
| H | 5.557494000 | –1.460739000 | 0.318840000 |
| H | 4.323668000 | –4.046248000 | –2.165980000 |

| | | | |
|---|---|---|---|
| H | 3.544814000 | –2.863042000 | –3.227850000 |
| H | 2.422559000 | –0.135804000 | –1.160750000 |

Ad-**1**•

B3LYP

| | | | |
|---|---|---|---|
| C | 0.000000000 | 0.000000000 | 0.008100000 |
| C | –2.196483000 | 0.995153000 | –0.689000000 |
| C | –3.595236000 | 1.007781000 | –0.710150000 |
| C | –1.467810000 | 0.000831000 | 0.006950000 |
| C | –4.271870000 | –0.001045000 | 0.000090000 |
| C | –2.198367000 | –0.994138000 | 0.700020000 |
| C | –3.597303000 | –1.008470000 | 0.714200000 |
| C | –3.506527000 | –3.116908000 | 2.183430000 |
| C | –4.404615000 | –2.065038000 | 1.485250000 |
| C | –5.270422000 | –1.380609000 | 2.583440000 |
| C | –5.357978000 | –2.826781000 | 0.518970000 |
| C | –4.353523000 | –4.156565000 | 2.944000000 |
| C | –5.196887000 | –3.446211000 | 4.020200000 |
| C | –5.286324000 | –4.887523000 | 1.960390000 |
| C | –6.121638000 | –2.417558000 | 3.342920000 |
| C | –6.210775000 | –3.860219000 | 1.281160000 |
| C | –7.055162000 | –3.140456000 | 2.351890000 |
| C | 6.448436000 | –3.448580000 | 1.281160000 |
| C | 5.127053000 | –3.226755000 | 0.518970000 |
| C | 6.875881000 | –2.134329000 | 1.960390000 |
| C | 6.247296000 | –4.539721000 | 2.351890000 |
| C | 2.136840000 | –3.699025000 | 0.000090000 |
| C | 3.990682000 | –2.781989000 | 1.485250000 |
| C | 0.924855000 | –3.617456000 | –0.710150000 |
| C | 5.776452000 | –1.691979000 | 2.944000000 |
| C | 2.672012000 | –2.611121000 | 0.714200000 |
| C | 4.452585000 | –1.478288000 | 2.183430000 |
| C | 0.236414000 | –2.399787000 | –0.689000000 |
| C | 1.960132000 | –1.406772000 | 0.700020000 |
| C | 0.733185000 | –1.271576000 | 0.006950000 |
| C | 5.154486000 | –4.092716000 | 3.342920000 |
| C | 3.830854000 | –3.874015000 | 2.583440000 |
| C | 5.582950000 | –2.777530000 | 4.020200000 |
| C | 0.967153000 | 6.510273000 | 3.342920000 |
| C | 1.439568000 | 5.254624000 | 2.583440000 |
| C | 0.807866000 | 7.680177000 | 2.351890000 |
| C | –0.386063000 | 6.223742000 | 4.020200000 |
| C | 2.135030000 | 3.700070000 | 0.000090000 |
| C | 0.413932000 | 4.847027000 | 1.485250000 |
| C | 2.670382000 | 2.609676000 | –0.710150000 |
| C | –0.237660000 | 7.308799000 | 1.281160000 |
| C | 0.925291000 | 3.619591000 | 0.714200000 |
| C | 0.230925000 | 6.053536000 | 0.518970000 |
| C | 1.960069000 | 1.404634000 | –0.689000000 |
| C | 0.238235000 | 2.400910000 | 0.700020000 |
| C | 0.734625000 | 1.270745000 | 0.006950000 |
| C | –1.422930000 | 5.848544000 | 2.944000000 |
| C | –0.946058000 | 4.595196000 | 2.183430000 |
| C | –1.589557000 | 7.021853000 | 1.960390000 |
| C | 5.575902000 | 2.774125000 | –4.022810000 |
| C | 5.150180000 | 4.089530000 | –3.344270000 |
| C | 3.827273000 | 3.871947000 | –2.583220000 |
| C | 5.769397000 | 1.687902000 | –2.947330000 |
| C | 4.446649000 | 1.475558000 | –2.184470000 |
| C | 3.987410000 | 2.779786000 | –1.485500000 |
| C | 6.245091000 | 4.534768000 | –2.354750000 |
| C | 6.870870000 | 2.128381000 | –1.965070000 |
| C | 6.446338000 | 3.442978000 | –1.284640000 |
| C | 5.125682000 | 3.223104000 | –0.520670000 |
| C | –5.354131000 | 2.827418000 | –0.520670000 |
| C | –4.401070000 | 2.063306000 | –1.485500000 |
| C | –5.266841000 | 1.378542000 | –2.583220000 |
| C | –6.204875000 | 3.861204000 | –1.284640000 |
| C | –3.501196000 | 3.113132000 | –2.184470000 |
| C | –7.049770000 | 3.141023000 | –2.354750000 |
| C | –6.116727000 | 2.415421000 | –3.344270000 |
| C | –5.278667000 | 4.886157000 | –1.965070000 |
| C | –4.346465000 | 4.152494000 | –2.947330000 |
| C | –5.190413000 | 3.441810000 | –4.022810000 |
| C | –0.945454000 | –4.588690000 | –2.184470000 |
| C | 0.413660000 | –4.843092000 | –1.485500000 |
| C | 0.228449000 | –6.050523000 | –0.520670000 |
| C | –1.422933000 | –5.840396000 | –2.947330000 |
| C | 1.439568000 | –5.250489000 | –2.583220000 |
| C | –1.592202000 | –7.014538000 | –1.965070000 |
| C | –0.241463000 | –7.304181000 | –1.284640000 |
| C | –0.385488000 | –6.215935000 | –4.022810000 |
| C | 0.966547000 | –6.504952000 | –3.344270000 |
| C | 0.804679000 | –7.675792000 | –2.354750000 |
| H | –1.633828000 | 1.748161000 | –1.236600000 |
| H | –5.362720000 | –0.001472000 | –0.001430000 |
| H | –1.637792000 | –1.746591000 | 1.250570000 |
| H | –2.821689000 | –2.617670000 | 2.889580000 |
| H | –2.877192000 | –3.628624000 | 1.435310000 |
| H | –5.929557000 | –0.624331000 | 2.125760000 |
| H | –4.609454000 | –0.841398000 | 3.284240000 |
| H | –6.019963000 | –2.113313000 | 0.001390000 |
| H | –4.759366000 | –3.327424000 | –0.262010000 |
| H | –3.677525000 | –4.884310000 | 3.424090000 |
| H | –5.794676000 | –4.184018000 | 4.583560000 |
| H | –4.537480000 | –2.942660000 | 4.748780000 |
| H | –4.692323000 | –5.424898000 | 1.200210000 |
| H | –5.884839000 | –5.645760000 | 2.495290000 |
| H | –6.723726000 | –1.897925000 | 4.107840000 |
| H | –6.877030000 | –4.373583000 | 0.566860000 |
| H | –7.688163000 | –3.868551000 | 2.888720000 |
| H | –7.736799000 | –2.414963000 | 1.873710000 |
| H | 7.226149000 | –3.768891000 | 0.566860000 |
| H | 4.840164000 | –4.156784000 | 0.001390000 |
| H | 5.261317000 | –2.458019000 | –0.262010000 |
| H | 7.044261000 | –1.351222000 | 1.200210000 |
| H | 7.831791000 | –2.273540000 | 2.495290000 |
| H | 7.194345000 | –4.723869000 | 2.888720000 |
| H | 5.959819000 | –5.492783000 | 1.873710000 |
| H | 2.682634000 | –4.643515000 | –0.001430000 |
| H | 6.068699000 | –0.742675000 | 3.424090000 |
| H | 3.677814000 | –1.134820000 | 2.889580000 |
| H | 4.581076000 | –0.677409000 | 1.435310000 |
| H | –0.697038000 | –2.289016000 | –1.236600000 |
| H | 2.331488000 | –0.545075000 | 1.250570000 |
| H | 5.005514000 | –4.873955000 | 4.107840000 |
| H | 3.505465000 | –4.822982000 | 2.125760000 |
| H | 3.033399000 | –3.571205000 | 3.284240000 |
| H | 6.520804000 | –2.926328000 | 4.583560000 |
| H | 4.817158000 | –2.458243000 | 4.748780000 |
| H | 1.718212000 | 6.771880000 | 4.107840000 |
| H | 2.424092000 | 5.447312000 | 2.125760000 |
| H | 1.576055000 | 4.412603000 | 3.284240000 |
| H | 0.493818000 | 8.592420000 | 2.888720000 |
| H | 1.776980000 | 7.907746000 | 1.873710000 |
| H | –0.726128000 | 7.110346000 | 4.583560000 |
| H | –0.279678000 | 5.400903000 | 4.748780000 |
| H | 2.680085000 | 4.644987000 | –0.001430000 |
| H | –0.349119000 | 8.142474000 | 0.566860000 |
| H | 1.179799000 | 6.270097000 | 0.001390000 |
| H | –0.501951000 | 5.785444000 | –0.262010000 |
| H | 2.330865000 | 0.540856000 | –1.236600000 |
| H | –0.693696000 | 2.291665000 | 1.250570000 |
| H | –2.391174000 | 5.626985000 | 3.424090000 |
| H | –0.856124000 | 3.752490000 | 2.889580000 |
| H | –1.703884000 | 4.306033000 | 1.435310000 |
| H | –2.351938000 | 6.776120000 | 1.200210000 |
| H | –1.946952000 | 7.919300000 | 2.495290000 |
| H | 6.513176000 | 2.921864000 | –4.587430000 |
| H | 4.808639000 | 2.456227000 | –4.750440000 |
| H | 5.000968000 | 4.871334000 | –4.108560000 |
| H | 3.028997000 | 3.569941000 | –3.283410000 |
| H | 3.502991000 | 4.821037000 | –2.125040000 |
| H | 6.059720000 | 0.738383000 | –3.428180000 |
| H | 4.576575000 | 0.674380000 | –1.436970000 |
| H | 3.669683000 | 1.132918000 | –2.888610000 |
| H | 5.959648000 | 5.488059000 | –1.875830000 |
| H | 7.191516000 | 4.717750000 | –2.893050000 |
| H | 7.826196000 | 2.266485000 | –2.501280000 |
| H | 7.039261000 | 1.344703000 | –1.205480000 |
| H | 7.225650000 | 3.761918000 | –0.571460000 |
| H | 4.840744000 | 4.153695000 | –0.002970000 |
| H | 5.259420000 | 2.454317000 | 0.260370000 |
| H | –6.017577000 | 2.115360000 | –0.002970000 |
| H | –4.755211000 | 3.327633000 | 0.260370000 |
| H | –4.606158000 | 0.838217000 | –3.283410000 |
| H | –5.926636000 | 0.623160000 | –2.125040000 |
| H | –6.870742000 | 4.376637000 | –0.571460000 |
| H | –2.872318000 | 3.626241000 | –1.436970000 |
| H | –2.815977000 | 2.611580000 | –2.888610000 |
| H | –7.732622000 | 2.417177000 | –1.875830000 |
| H | –7.681449000 | 3.869161000 | –2.893050000 |
| H | –6.719183000 | 1.895299000 | –4.108560000 |
| H | –5.875932000 | 5.644442000 | –2.501280000 |
| H | –4.684177000 | 5.423828000 | –1.205480000 |
| H | –3.669318000 | 4.878680000 | –3.428180000 |
| H | –5.786996000 | 4.179643000 | –4.587430000 |
| H | –4.531475000 | 2.936290000 | –4.750440000 |
| H | –1.704257000 | –4.300621000 | –1.436970000 |
| H | –0.853706000 | –3.744498000 | –2.888610000 |
| H | 1.176833000 | –6.269055000 | –0.002970000 |
| H | –0.504209000 | –5.781950000 | 0.260370000 |
| H | –2.390401000 | –5.617063000 | –3.428180000 |
| H | 1.577162000 | –4.408159000 | –3.283410000 |

| | | | |
|---|---|---|---|
| H | 2.423646000 | –5.444198000 | –2.125040000 |
| H | –1.950264000 | –7.910927000 | –2.501280000 |
| H | –2.355084000 | –6.768531000 | –1.205480000 |
| H | –0.354908000 | –8.138556000 | –0.571460000 |
| H | –0.726179000 | –7.101507000 | –4.587430000 |
| H | –0.277165000 | –5.392517000 | –4.750440000 |
| H | 1.718215000 | –6.766633000 | –4.108560000 |
| H | 1.772974000 | –7.905236000 | –1.875830000 |
| H | 0.489933000 | –8.586911000 | –2.893050000 |

B3LYP-D3(BJ)

| | | | |
|---|---|---|---|
| C | 0.000000000 | 0.000000000 | 0.028259000 |
| C | –1.668700000 | 1.742520000 | 0.720989000 |
| C | –2.147510000 | 3.056740000 | 0.734059000 |
| C | –0.489480000 | 1.383800000 | 0.025369000 |
| C | –1.424710000 | 4.025870000 | 0.014999000 |
| C | 0.203060000 | 2.399510000 | –0.675911000 |
| C | –0.251560000 | 3.721860000 | –0.698931000 |
| C | 1.751010000 | 4.329110000 | –2.193241000 |
| C | 0.469910000 | 4.830650000 | –1.480741000 |
| C | –0.474190000 | 5.418080000 | –2.570311000 |
| C | 0.885620000 | 5.984230000 | –0.521961000 |
| C | 2.442340000 | 5.469920000 | –2.966241000 |
| C | 1.480740000 | 6.028920000 | –4.032341000 |
| C | 2.835950000 | 6.594010000 | –1.989481000 |
| C | 0.212730000 | 6.563110000 | –3.340371000 |
| C | 1.569350000 | 7.128960000 | –1.295521000 |
| C | 0.598480000 | 7.685140000 | –2.356471000 |
| C | 5.389185000 | –4.923577000 | –1.295521000 |
| C | 4.739685000 | –3.759084000 | –0.521961000 |
| C | 4.292605000 | –5.753010000 | –1.989481000 |
| C | 6.356286000 | –4.360869000 | –2.356471000 |
| C | 4.198861000 | –0.779100000 | 0.014999000 |
| C | 3.948510000 | –2.822279000 | –1.480741000 |
| C | 3.720969000 | 0.331428000 | 0.734059000 |
| C | 3.515920000 | –4.850088000 | –2.966241000 |
| C | 3.349005000 | –1.643073000 | –0.698931000 |
| C | 2.873614000 | –3.680974000 | –2.193241000 |
| C | 2.343416000 | 0.573877000 | 0.720989000 |
| C | 1.976506000 | –1.375610000 | –0.675911000 |
| C | 1.443146000 | –0.267998000 | 0.025369000 |
| C | 5.577455000 | –3.465785000 | –3.340371000 |
| C | 4.929290000 | –2.298379000 | –2.570311000 |
| C | 4.480828000 | –4.296818000 | –4.032341000 |
| C | –5.790185000 | –3.097325000 | –3.340371000 |
| C | –4.455100000 | –3.119700000 | –2.570311000 |
| C | –6.954766000 | –3.324271000 | –2.356471000 |
| C | –5.961568000 | –1.732101000 | –4.032341000 |
| C | –2.774151000 | –3.246770000 | 0.014999000 |
| C | –4.418421000 | –2.008371000 | –1.480741000 |
| C | –1.573459000 | –3.388168000 | 0.734059000 |
| C | –6.958535000 | –2.205383000 | –1.295521000 |
| C | –3.097445000 | –2.078787000 | –0.698931000 |
| C | –5.625305000 | –2.225146000 | –0.521961000 |
| C | –0.674716000 | –2.316396000 | 0.720989000 |
| C | –2.179567000 | –1.023900000 | –0.675911000 |
| C | –0.953666000 | –1.115802000 | 0.025369000 |
| C | –5.958260000 | –0.619831000 | –2.966241000 |
| C | –4.624624000 | –0.648136000 | –2.193241000 |
| C | –7.128555000 | –0.841000000 | –1.989481000 |
| C | –0.773090000 | –6.221820000 | 4.017579000 |
| C | –2.150490000 | –6.253300000 | 3.329349000 |
| C | –2.384010000 | –4.923870000 | 2.584799000 |
| C | 0.322150000 | –6.024870000 | 2.952019000 |
| C | 0.083280000 | –4.697480000 | 2.204799000 |
| C | –1.294910000 | –4.693230000 | 1.496649000 |
| C | –2.195590000 | –7.420710000 | 2.323729000 |
| C | 0.283380000 | –7.197190000 | 1.953639000 |
| C | –1.092780000 | –7.229460000 | 1.263329000 |
| C | –1.323670000 | –5.901330000 | 0.515909000 |
| C | –4.448867000 | 4.096997000 | 0.515909000 |
| C | –3.417002000 | 3.468040000 | 1.496649000 |
| C | –3.072192000 | 4.526548000 | 2.584799000 |
| C | –5.714506000 | 4.561105000 | 1.263329000 |
| C | –4.109777000 | 2.276617000 | 2.204799000 |
| C | –5.328729000 | 5.611792000 | 2.323729000 |
| C | –4.340272000 | 4.989029000 | 3.329349000 |
| C | –6.374640000 | 3.353181000 | 1.953639000 |
| C | –5.378766000 | 2.733445000 | 2.952019000 |
| C | –5.001709000 | 3.780425000 | 4.017579000 |
| C | 4.026497000 | 2.420863000 | 2.204799000 |
| C | 4.711911000 | 1.225190000 | 1.496649000 |
| C | 5.772537000 | 1.804333000 | 0.515909000 |
| C | 5.056615000 | 3.291425000 | 2.952019000 |
| C | 5.456202000 | 0.397322000 | 2.584799000 |
| C | 6.091259000 | 3.844010000 | 1.953639000 |
| C | 6.807286000 | 2.668355000 | 1.263329000 |
| C | 5.774799000 | 2.441395000 | 4.017579000 |
| C | 6.490762000 | 1.264271000 | 3.329349000 |
| C | 7.524318000 | 1.808919000 | 2.323729000 |
| H | –2.189580000 | 0.962960000 | 1.272679000 |
| H | –1.790400000 | 5.053910000 | 0.007429000 |
| H | 1.096640000 | 2.117150000 | –1.228491000 |
| H | 1.497310000 | 3.513610000 | –2.891521000 |
| H | 2.451910000 | 3.908230000 | –1.452321000 |
| H | –1.402450000 | 5.788450000 | –2.104651000 |
| H | –0.770640000 | 4.613560000 | –3.265621000 |
| H | 0.000000000 | 6.372990000 | 0.007159000 |
| H | 1.564920000 | 5.585970000 | 0.251869000 |
| H | 3.347010000 | 5.071500000 | –3.456181000 |
| H | 1.972920000 | 6.835360000 | –4.603881000 |
| H | 1.214470000 | 5.238940000 | –4.756391000 |
| H | 3.548420000 | 6.212210000 | –1.237051000 |
| H | 3.348080000 | 7.408210000 | –2.531751000 |
| H | –0.486040000 | 6.958150000 | –4.097611000 |
| H | 1.842940000 | 7.929610000 | –0.587051000 |
| H | 1.069580000 | 8.522340000 | –2.900761000 |
| H | –0.305870000 | 8.090510000 | –1.868951000 |
| H | 5.945774000 | –5.560838000 | –0.587051000 |
| H | 5.519171000 | –3.186495000 | 0.007159000 |
| H | 4.055132000 | –4.148245000 | 0.251869000 |
| H | 3.605722000 | –6.179127000 | –1.237051000 |
| H | 4.741658000 | –6.603627000 | –2.531751000 |
| H | 6.845773000 | –5.187453000 | –2.900761000 |
| H | 7.159522000 | –3.780364000 | –1.868951000 |
| H | 5.272014000 | –0.976423000 | 0.007429000 |
| H | 2.718543000 | –5.434346000 | –3.456181000 |
| H | 2.294220000 | –3.053513000 | –2.891521000 |
| H | 2.158671000 | –4.077531000 | –1.452321000 |
| H | 1.928738000 | 1.414752000 | 1.272679000 |
| H | 1.285186000 | –2.008293000 | –1.228491000 |
| H | 6.268955000 | –3.058152000 | –4.097611000 |
| H | 5.714170000 | –1.679668000 | –2.104651000 |
| H | 4.380780000 | –1.639386000 | –3.265621000 |
| H | 4.933135000 | –5.126279000 | –4.603881000 |
| H | 3.929820000 | –3.671232000 | –4.756391000 |
| H | –5.782915000 | –3.899998000 | –4.097611000 |
| H | –4.311720000 | –4.108782000 | –2.104651000 |
| H | –3.610140000 | –2.974174000 | –3.265621000 |
| H | –7.915353000 | –3.334887000 | –2.900761000 |
| H | –6.853652000 | –4.310146000 | –1.868951000 |
| H | –6.906055000 | –1.709081000 | –4.603881000 |
| H | –5.144290000 | –1.567708000 | –4.756391000 |
| H | –3.481614000 | –4.077487000 | 0.007429000 |
| H | –7.788714000 | –2.368772000 | –0.587051000 |
| H | –5.519171000 | –3.186495000 | 0.007159000 |
| H | –5.620052000 | –1.437724000 | 0.251869000 |
| H | 0.260842000 | –2.377712000 | 1.272679000 |
| H | –2.381826000 | –0.108857000 | –1.228491000 |
| H | –6.065553000 | 0.362846000 | –3.456181000 |
| H | –3.791530000 | –0.460096000 | –2.891521000 |
| H | –4.610581000 | 0.169301000 | –1.452321000 |
| H | –7.154142000 | –0.033083000 | –1.237051000 |
| H | –8.089738000 | –0.804583000 | –2.531751000 |
| H | –0.601120000 | –7.161570000 | 4.571209000 |
| H | –0.736270000 | –5.402410000 | 4.756729000 |
| H | –2.941870000 | –6.385850000 | 4.086889000 |
| H | –2.372080000 | –4.079600000 | 3.295819000 |
| H | –3.384390000 | –4.930160000 | 2.121419000 |
| H | 1.310810000 | –5.986440000 | 3.440119000 |
| H | 0.886530000 | –4.541730000 | 1.464629000 |
| H | 0.140890000 | –3.858580000 | 2.918869000 |
| H | –3.186300000 | –7.465780000 | 1.837669000 |
| H | –2.054520000 | –8.381110000 | 2.849809000 |
| H | 0.470930000 | –8.150740000 | 2.477949000 |
| H | 1.083320000 | –7.083060000 | 1.200889000 |
| H | –1.126630000 | –8.061260000 | 0.538929000 |
| H | –2.292010000 | –5.936810000 | –0.010041000 |
| H | –0.549950000 | –5.758290000 | –0.258501000 |
| H | –3.995424000 | 4.953344000 | –0.010041000 |
| H | –4.711851000 | 3.355416000 | –0.258501000 |
| H | –2.346997000 | 4.094081000 | 3.295819000 |
| H | –2.577449000 | 5.396048000 | 2.121419000 |
| H | –6.417941000 | 5.006320000 | 0.538929000 |
| H | –4.376519000 | 1.503107000 | 1.464629000 |
| H | –3.412074000 | 1.807276000 | 2.918869000 |
| H | –4.872405000 | 6.492307000 | 1.837669000 |
| H | –6.230994000 | 5.969821000 | 2.849809000 |
| H | –4.059374000 | 5.740659000 | 4.086889000 |
| H | –7.294213000 | 3.667532000 | 2.477949000 |
| H | –6.675770000 | 2.603347000 | 1.200889000 |
| H | –5.839814000 | 1.858025000 | 3.440119000 |
| H | –5.901542000 | 4.101370000 | 4.571209000 |
| H | –4.310490000 | 3.338833000 | 4.756729000 |
| H | 3.489989000 | 3.038623000 | 1.464629000 |

| | | | |
|---|---|---|---|
| H | 3.271183000 | 2.051305000 | 2.918869000 |
| H | 6.287433000 | 0.983466000 | –0.010041000 |
| H | 5.261800000 | 2.402875000 | –0.258501000 |
| H | 4.529004000 | 4.128415000 | 3.440119000 |
| H | 4.719077000 | –0.014481000 | 3.295819000 |
| H | 5.961839000 | –0.465887000 | 2.121419000 |
| H | 6.823283000 | 4.483208000 | 2.477949000 |
| H | 5.592450000 | 4.479713000 | 1.200889000 |
| H | 7.544571000 | 3.054940000 | 0.538929000 |
| H | 6.502662000 | 3.060200000 | 4.571209000 |
| H | 5.046759000 | 2.063577000 | 4.756729000 |
| H | 7.001243000 | 0.645191000 | 4.086889000 |
| H | 8.058705000 | 0.973474000 | 1.837669000 |
| H | 8.285514000 | 2.411289000 | 2.849809000 |

M06-2X

| | | | |
|---|---|---|---|
| C | 0.000000000 | 0.000000000 | 0.022280000 |
| C | 1.019490000 | 2.182330000 | –0.641170000 |
| C | 1.031640000 | 3.576460000 | –0.661270000 |
| C | 0.003020000 | 1.462690000 | 0.019140000 |
| C | 0.000000000 | 4.255290000 | 0.002390000 |
| C | –1.014190000 | 2.188000000 | 0.671740000 |
| C | –1.029970000 | 3.582460000 | 0.674700000 |
| C | –3.170050000 | 3.484520000 | 2.057880000 |
| C | –2.111980000 | 4.384920000 | 1.393080000 |
| C | –1.468530000 | 5.258100000 | 2.495160000 |
| C | –2.838370000 | 5.314850000 | 0.393420000 |
| C | –4.236370000 | 4.325750000 | 2.771750000 |
| C | –3.569720000 | 5.180250000 | 3.857870000 |
| C | –4.936370000 | 5.240630000 | 1.759380000 |
| C | –2.530230000 | 6.104130000 | 3.210410000 |
| C | –3.898530000 | 6.163740000 | 1.108830000 |
| C | –3.223460000 | 7.018390000 | 2.190850000 |
| C | –3.388690000 | –6.458096000 | 1.108830000 |
| C | –3.183610000 | –5.115526000 | 0.393420000 |
| C | –2.070334000 | –6.895337000 | 1.759380000 |
| C | –4.466374000 | –6.300793000 | 2.190850000 |
| C | –3.685189000 | –2.127645000 | 0.002390000 |
| C | –2.741462000 | –4.021488000 | 1.393080000 |
| C | –3.613125000 | –0.894804000 | –0.661270000 |
| C | –1.628024000 | –5.831679000 | 2.771750000 |
| C | –2.587516000 | –2.683210000 | 0.674700000 |
| C | –1.432658000 | –4.487604000 | 2.057880000 |
| C | –2.399698000 | –0.208261000 | –0.641170000 |
| C | –1.387769000 | –1.972314000 | 0.671740000 |
| C | –1.268237000 | –0.728730000 | 0.019140000 |
| C | –4.021217000 | –5.243308000 | 3.210410000 |
| C | –3.819383000 | –3.900834000 | 2.495160000 |
| C | –2.701368000 | –5.681593000 | 3.857870000 |
| C | 6.551447000 | –0.860821000 | 3.210410000 |
| C | 5.287913000 | –1.357266000 | 2.495160000 |
| C | 7.689834000 | –0.717597000 | 2.190850000 |
| C | 6.271088000 | 0.501343000 | 3.857870000 |
| C | 3.685189000 | –2.127645000 | 0.002390000 |
| C | 4.853442000 | –0.363432000 | 1.393080000 |
| C | 2.581485000 | –2.681656000 | –0.661270000 |
| C | 7.287220000 | 0.294356000 | 1.108830000 |
| C | 3.617486000 | –0.899250000 | 0.674700000 |
| C | 6.021980000 | –0.199324000 | 0.393420000 |
| C | 1.380208000 | –1.974069000 | –0.641170000 |
| C | 2.401959000 | –0.215686000 | 0.671740000 |
| C | 1.265217000 | –0.733960000 | 0.019140000 |
| C | 5.864394000 | 1.505929000 | 2.771750000 |
| C | 4.602708000 | 1.003084000 | 2.057880000 |
| C | 7.006704000 | 1.654707000 | 1.759380000 |
| C | 2.682660000 | –5.660580000 | –3.864820000 |
| C | 4.004830000 | –5.226680000 | –3.219230000 |
| C | 3.805640000 | –3.888070000 | –2.496230000 |
| C | 1.613080000 | –5.816290000 | –2.775940000 |
| C | 1.420430000 | –4.476410000 | –2.053250000 |
| C | 2.731990000 | –4.014650000 | –1.391120000 |
| C | 4.453100000 | –6.289920000 | –2.206980000 |
| C | 2.058550000 | –6.885880000 | –1.771050000 |
| C | 3.379190000 | –6.452940000 | –1.122160000 |
| C | 3.177160000 | –5.114150000 | –0.398720000 |
| C | 2.840404000 | 5.308576000 | –0.398720000 |
| C | 2.110794000 | 4.373298000 | –1.391120000 |
| C | 1.464347000 | 5.239816000 | –2.496230000 |
| C | 3.898815000 | 6.152935000 | –1.122160000 |
| C | 3.166470000 | 3.468334000 | –2.053250000 |
| C | 3.220680000 | 7.001458000 | –2.206980000 |
| C | 2.524022000 | 6.081625000 | –3.219230000 |
| C | 4.934072000 | 5.225697000 | –1.771050000 |
| C | 4.230515000 | 4.305114000 | –2.775940000 |
| C | 3.560876000 | 5.153542000 | –3.864820000 |
| C | –4.586900000 | 1.008076000 | –2.053250000 |
| C | –4.842784000 | –0.358648000 | –1.391120000 |

| | | | |
|---|---|---|---|
| C | –6.017564000 | –0.194427000 | –0.398720000 |
| C | –5.843595000 | 1.511176000 | –2.775940000 |
| C | –5.269987000 | –1.351746000 | –2.496230000 |
| C | –6.992622000 | 1.660183000 | –1.771050000 |
| C | –7.278005000 | 0.300005000 | –1.122160000 |
| C | –6.243536000 | 0.507038000 | –3.864820000 |
| C | –6.528853000 | –0.854945000 | –3.219230000 |
| C | –7.673781000 | –0.711538000 | –2.206980000 |
| H | 1.791820000 | 1.616430000 | –1.159350000 |
| H | –0.000150000 | 5.347220000 | –0.003490000 |
| H | –1.785860000 | 1.626100000 | 1.195040000 |
| H | –2.683440000 | 2.814780000 | 2.786260000 |
| H | –3.646840000 | 2.844650000 | 1.295130000 |
| H | –0.705760000 | 5.917440000 | 2.049310000 |
| H | –0.949400000 | 4.604220000 | 3.216150000 |
| H | –2.107970000 | 5.975460000 | –0.101440000 |
| H | –3.306280000 | 4.700490000 | –0.395020000 |
| H | –4.974390000 | 3.651370000 | 3.232940000 |
| H | –4.329840000 | 5.778430000 | 4.387260000 |
| H | –3.083810000 | 4.530710000 | 4.604630000 |
| H | –5.439970000 | 4.635130000 | 0.986930000 |
| H | –5.712040000 | 5.839430000 | 2.265210000 |
| H | –2.042770000 | 6.715940000 | 3.985560000 |
| H | –4.392200000 | 6.818060000 | 0.373570000 |
| H | –3.974430000 | 7.646950000 | 2.697570000 |
| H | –2.485570000 | 7.696310000 | 1.730020000 |
| H | –3.708513000 | –7.212787000 | 0.373570000 |
| H | –4.120915000 | –4.813286000 | –0.101440000 |
| H | –2.417604000 | –5.213567000 | –0.395020000 |
| H | –1.294155000 | –7.028717000 | 0.986930000 |
| H | –2.201075000 | –7.866487000 | 2.265210000 |
| H | –4.635238000 | –7.265432000 | 2.697570000 |
| H | –5.422415000 | –6.000722000 | 1.730020000 |
| H | –4.630753000 | –2.673740000 | –0.003490000 |
| H | –0.674984000 | –6.133633000 | 3.232940000 |
| H | –1.095951000 | –3.731317000 | 2.786260000 |
| H | –0.640119000 | –4.580581000 | 1.295130000 |
| H | –2.295780000 | 0.743547000 | –1.159350000 |
| H | –0.515314000 | –2.359650000 | 1.195040000 |
| H | –4.794790000 | –5.127061000 | 3.985560000 |
| H | –4.771773000 | –3.569926000 | 2.049310000 |
| H | –3.512671000 | –3.124315000 | 3.216150000 |
| H | –2.839347000 | –6.638966000 | 4.387260000 |
| H | –2.381805000 | –4.936013000 | 4.604630000 |
| H | 6.837560000 | –1.588879000 | 3.985560000 |
| H | 5.477533000 | –2.347514000 | 2.049310000 |
| H | 4.462071000 | –1.479905000 | 3.216150000 |
| H | 8.609668000 | –0.381517000 | 2.697570000 |
| H | 7.907985000 | –1.695588000 | 1.730020000 |
| H | 7.169187000 | 0.860537000 | 4.387260000 |
| H | 5.465615000 | 0.405303000 | 4.604630000 |
| H | 4.630903000 | –2.673480000 | –0.003490000 |
| H | 8.100713000 | 0.394727000 | 0.373570000 |
| H | 6.228885000 | –1.162174000 | –0.101440000 |
| H | 5.723884000 | 0.513078000 | –0.395020000 |
| H | 0.503959000 | –2.359977000 | –1.159350000 |
| H | 2.301174000 | 0.733550000 | 1.195040000 |
| H | 5.649374000 | 2.482263000 | 3.232940000 |
| H | 3.779391000 | 0.916537000 | 2.786260000 |
| H | 4.286959000 | 1.735931000 | 1.295130000 |
| H | 6.734125000 | 2.393587000 | 0.986930000 |
| H | 7.913115000 | 2.027057000 | 2.265210000 |
| H | 2.818350000 | –6.615050000 | –4.400010000 |
| H | 2.360900000 | –4.910680000 | –4.606290000 |
| H | 4.775880000 | –5.106450000 | –3.996260000 |
| H | 3.496200000 | –3.107980000 | –3.212210000 |
| H | 4.759650000 | –3.559220000 | –2.052410000 |
| H | 0.658330000 | –6.115350000 | –3.235510000 |
| H | 0.631020000 | –4.575100000 | –1.288020000 |
| H | 1.080570000 | –3.714970000 | –2.774830000 |
| H | 5.410830000 | –5.992780000 | –1.747710000 |
| H | 4.619960000 | –7.251780000 | –2.719620000 |
| H | 2.187380000 | –7.854040000 | –2.283060000 |
| H | 1.284790000 | –7.023680000 | –0.996940000 |
| H | 3.701180000 | –7.211940000 | –0.392280000 |
| H | 4.116150000 | –4.814440000 | 0.094640000 |
| H | 2.413610000 | –5.215550000 | 0.391680000 |
| H | 2.111352000 | 5.971911000 | 0.094640000 |
| H | 3.309994000 | 4.698023000 | 0.391680000 |
| H | 0.943489000 | 4.581788000 | –3.212210000 |
| H | 0.702550000 | 5.901588000 | –2.052410000 |
| H | 4.395133000 | 6.811286000 | –0.392280000 |
| H | 3.646643000 | 2.834030000 | –1.288020000 |
| H | 2.676973000 | 2.793286000 | –2.774830000 |
| H | 2.484484000 | 7.682306000 | –1.747710000 |
| H | 3.970245000 | 7.626893000 | –2.719620000 |
| H | 2.034375000 | 6.689259000 | –3.996260000 |
| H | 5.708108000 | 5.821347000 | –2.283060000 |

| | | | |
|---|---|---|---|
| H | 5.440290000 | 4.624501000 | –0.996940000 |
| H | 4.966883000 | 3.627806000 | –3.235510000 |
| H | 4.319626000 | 5.748288000 | –4.400010000 |
| H | 3.072323000 | 4.499940000 | –4.606290000 |
| H | –4.277663000 | 1.741070000 | –1.288020000 |
| H | –3.757543000 | 0.921684000 | –2.774830000 |
| H | –6.227502000 | –1.157471000 | 0.094640000 |
| H | –5.723604000 | 0.517527000 | 0.391680000 |
| H | –5.625214000 | 2.487544000 | –3.235510000 |
| H | –4.439690000 | –1.473808000 | –3.212210000 |
| H | –5.462200000 | –2.342368000 | –2.052410000 |
| H | –7.895488000 | 2.032693000 | –2.283060000 |
| H | –6.725080000 | 2.399179000 | –0.996940000 |
| H | –8.096313000 | 0.400654000 | –0.392280000 |
| H | –7.137976000 | 0.866762000 | –4.400010000 |
| H | –5.433224000 | 0.410740000 | –4.606290000 |
| H | –6.810255000 | –1.582809000 | –3.996260000 |
| H | –7.895315000 | –1.689527000 | –1.747710000 |
| H | –8.590206000 | –0.375113000 | –2.719620000 |

## *Dimers*

**Table S16.** Dimers R-$\mathbf{1}_2$ in gas phase.

H-$\mathbf{1}_2$

B3LYP

| | | | |
|---|---|---|---|
| C | –2.228037000 | 3.438986000 | 2.543080000 |
| C | –1.198184000 | 3.636963000 | 1.619150000 |
| C | –0.497253000 | 2.552231000 | 1.089870000 |
| C | –0.807850000 | 1.218562000 | 1.436900000 |
| C | –1.819381000 | 1.050420000 | 2.400150000 |
| C | –2.521048000 | 2.135936000 | 2.939160000 |
| C | 1.459231000 | 0.090338000 | 1.436900000 |
| C | 2.458924000 | –0.845482000 | 1.089870000 |
| C | 3.748795000 | –0.780824000 | 1.619150000 |
| C | 4.092268000 | 0.210044000 | 2.543080000 |
| C | 3.110299000 | 1.115324000 | 2.939160000 |
| C | 1.819381000 | 1.050420000 | 2.400150000 |
| C | –0.589251000 | –3.251260000 | 2.939160000 |
| C | 0.000000000 | –2.100840000 | 2.400150000 |
| C | –0.651380000 | –1.308900000 | 1.436900000 |
| C | –1.961670000 | –1.706750000 | 1.089870000 |
| C | –2.550611000 | –2.856140000 | 1.619150000 |
| C | –1.864231000 | –3.649030000 | 2.543080000 |
| C | 0.000000000 | 0.000000000 | 0.864810000 |
| C | 2.228037000 | –3.438986000 | –2.543080000 |
| C | 2.521048000 | –2.135936000 | –2.939160000 |
| C | 1.819381000 | –1.050420000 | –2.400150000 |
| C | 0.807850000 | –1.218562000 | –1.436900000 |
| C | 0.497253000 | –2.552231000 | –1.089870000 |
| C | 1.198184000 | –3.636963000 | –1.619150000 |
| C | 0.651380000 | 1.308900000 | –1.436900000 |
| C | 1.961670000 | 1.706750000 | –1.089870000 |
| C | 2.550611000 | 2.856140000 | –1.619150000 |
| C | 1.864231000 | 3.649030000 | –2.543080000 |
| C | 0.589251000 | 3.251260000 | –2.939160000 |
| C | 0.000000000 | 2.100840000 | –2.400150000 |
| C | –3.110299000 | –1.115324000 | –2.939160000 |
| C | –1.819381000 | –1.050420000 | –2.400150000 |
| C | –1.459231000 | –0.090338000 | –1.436900000 |
| C | –2.458924000 | 0.845482000 | –1.089870000 |
| C | –3.748795000 | 0.780824000 | –1.619150000 |
| C | –4.092268000 | –0.210044000 | –2.543080000 |
| C | 0.000000000 | 0.000000000 | –0.864810000 |
| H | –2.777916000 | 4.287287000 | 2.957110000 |
| H | –0.923981000 | 4.647937000 | 1.308070000 |
| H | 0.314513000 | 2.763191000 | 0.402090000 |
| H | –2.079463000 | 0.056744000 | 2.754520000 |
| H | –3.302601000 | 1.946827000 | 3.679100000 |
| H | 2.235737000 | –1.653972000 | 0.402090000 |
| H | 4.487222000 | –1.523777000 | 1.308070000 |
| H | 5.101857000 | 0.262103000 | 2.957110000 |
| H | 3.337302000 | 1.886723000 | 3.679100000 |
| H | 1.088874000 | 1.772496000 | 2.754520000 |
| H | –0.034701000 | –3.833550000 | 3.679100000 |
| H | 0.990590000 | –1.829240000 | 2.754520000 |
| H | –2.550250000 | –1.109220000 | 0.402090000 |
| H | –3.563241000 | –3.124159000 | 1.308070000 |
| H | –2.323941000 | –4.549390000 | 2.957110000 |
| H | 2.777916000 | –4.287287000 | –2.957110000 |
| H | 3.302601000 | –1.946827000 | –3.679100000 |
| H | 2.079463000 | –0.056744000 | –2.754520000 |
| H | –0.314513000 | –2.763191000 | –0.402090000 |
| H | 0.923981000 | –4.647937000 | –1.308070000 |
| H | 2.550250000 | 1.109220000 | –0.402090000 |
| H | 3.563241000 | 3.124159000 | –1.308070000 |
| H | 2.323941000 | 4.549390000 | –2.957110000 |
| H | 0.034701000 | 3.833550000 | –3.679100000 |
| H | –0.990590000 | 1.829240000 | –2.754520000 |
| H | –3.337302000 | –1.886723000 | –3.679100000 |
| H | –1.088874000 | –1.772496000 | –2.754520000 |
| H | –2.235737000 | 1.653972000 | –0.402090000 |
| H | –4.487222000 | 1.523777000 | –1.308070000 |
| H | –5.101857000 | –0.262103000 | –2.957110000 |

B3LYP-D3(BJ)

| | | | |
|---|---|---|---|
| C | –2.822218000 | 3.001211000 | 2.417917000 |
| C | –1.802221000 | 3.372556000 | 1.537696000 |
| C | –0.900580000 | 2.425545000 | 1.054752000 |
| C | –0.995971000 | 1.062371000 | 1.405227000 |
| C | –1.991839000 | 0.724027000 | 2.334568000 |
| C | –2.895756000 | 1.671866000 | 2.826882000 |
| C | 1.418026000 | 0.331350000 | 1.405227000 |
| C | 2.550874000 | –0.432847000 | 1.054752000 |
| C | 3.821829000 | –0.125509000 | 1.537696000 |
| C | 4.010234000 | 0.943507000 | 2.417917000 |
| C | 2.895756000 | 1.671866000 | 2.826882000 |
| C | 1.622945000 | 1.362969000 | 2.334568000 |
| C | 0.000000000 | –3.343731000 | 2.826882000 |
| C | 0.368893000 | –2.086996000 | 2.334568000 |
| C | –0.422055000 | –1.393722000 | 1.405227000 |
| C | –1.650294000 | –1.992698000 | 1.054752000 |
| C | –2.019608000 | –3.247047000 | 1.537696000 |
| C | –1.188016000 | –3.944718000 | 2.417917000 |
| C | 0.000000000 | 0.000000000 | 0.850251000 |
| C | 2.822218000 | –3.001211000 | –2.417917000 |
| C | 2.895756000 | –1.671866000 | –2.826882000 |
| C | 1.991839000 | –0.724027000 | –2.334568000 |
| C | 0.995971000 | –1.062371000 | –1.405227000 |
| C | 0.900580000 | –2.425545000 | –1.054752000 |
| C | 1.802221000 | –3.372556000 | –1.537696000 |
| C | 0.422055000 | 1.393722000 | –1.405227000 |
| C | 1.650294000 | 1.992698000 | –1.054752000 |
| C | 2.019608000 | 3.247047000 | –1.537696000 |
| C | 1.188016000 | 3.944718000 | –2.417917000 |
| C | 0.000000000 | 3.343731000 | –2.826882000 |
| C | –0.368893000 | 2.086996000 | –2.334568000 |
| C | –2.895756000 | –1.671866000 | –2.826882000 |
| C | –1.622945000 | –1.362969000 | –2.334568000 |
| C | –1.418026000 | –0.331350000 | –1.405227000 |
| C | –2.550874000 | 0.432847000 | –1.054752000 |
| C | –3.821829000 | 0.125509000 | –1.537696000 |
| C | –4.010234000 | –0.943507000 | –2.417917000 |
| C | 0.000000000 | 0.000000000 | –0.850251000 |
| H | –3.532634000 | 3.741198000 | 2.792385000 |
| H | –1.696561000 | 4.413188000 | 1.223392000 |
| H | –0.109155000 | 2.768038000 | 0.399198000 |
| H | –2.082027000 | –0.297879000 | 2.690825000 |
| H | –3.661725000 | 1.355379000 | 3.538310000 |
| H | 2.451769000 | –1.289488000 | 0.399198000 |
| H | 4.670213000 | –0.737329000 | 1.223392000 |
| H | 5.006289000 | 1.188752000 | 2.792385000 |
| H | 3.004655000 | 2.493458000 | 3.538310000 |
| H | 0.783043000 | 1.952028000 | 2.690825000 |
| H | 0.657070000 | –3.848837000 | 3.538310000 |
| H | 1.298984000 | –1.654149000 | 2.690825000 |
| H | –2.342614000 | –1.478550000 | 0.399198000 |
| H | –2.973653000 | –3.675859000 | 1.223392000 |
| H | –1.473655000 | –4.929949000 | 2.792385000 |
| H | 3.532634000 | –3.741198000 | –2.792385000 |
| H | 3.661725000 | –1.355379000 | –3.538310000 |
| H | 2.082027000 | 0.297879000 | –2.690825000 |
| H | 0.109155000 | –2.768038000 | –0.399198000 |
| H | 1.696561000 | –4.413188000 | –1.223392000 |
| H | 2.342614000 | 1.478550000 | –0.399198000 |
| H | 2.973653000 | 3.675859000 | –1.223392000 |
| H | 1.473655000 | 4.929949000 | –2.792385000 |
| H | –0.657070000 | 3.848837000 | –3.538310000 |
| H | –1.298984000 | 1.654149000 | –2.690825000 |
| H | –3.004655000 | –2.493458000 | –3.538310000 |
| H | –0.783043000 | –1.952028000 | –2.690825000 |
| H | –2.451769000 | 1.289488000 | –0.399198000 |
| H | –4.670213000 | 0.737329000 | –1.223392000 |
| H | –5.006289000 | –1.188752000 | –2.792385000 |

M06-2X

| | | | |
|---|---|---|---|
| C | 1.855256000 | 3.692895000 | 2.370669000 |
| C | 2.560400000 | 2.837163000 | 1.524637000 |
| C | 1.981792000 | 1.659495000 | 1.063183000 |
| C | 0.660911000 | 1.297758000 | 1.399596000 |
| C | 0.000000000 | 2.139032000 | 2.304112000 |
| C | 0.580305000 | 3.320115000 | 2.774856000 |
| C | 0.793436000 | –1.221244000 | 1.399596000 |
| C | 0.446268000 | –2.546030000 | 1.063183000 |
| C | 1.176856000 | –3.635953000 | 1.524637000 |
| C | 2.270513000 | –3.453146000 | 2.370669000 |
| C | 2.585151000 | –2.162617000 | 2.774856000 |
| C | 1.852456000 | –1.069516000 | 2.304112000 |

| | | | |
|---|---|---|---|
| C | –3.165457000 | –1.157498000 | 2.774856000 |
| C | –1.852456000 | –1.069516000 | 2.304112000 |
| C | –1.454346000 | –0.076513000 | 1.399596000 |
| C | –2.428061000 | 0.886535000 | 1.063183000 |
| C | –3.737256000 | 0.798790000 | 1.524637000 |
| C | –4.125769000 | –0.239749000 | 2.370669000 |
| C | 0.000000000 | 0.000000000 | 0.845722000 |
| C | –1.855256000 | –3.692895000 | –2.370669000 |
| C | –0.580305000 | –3.320115000 | –2.774856000 |
| C | 0.000000000 | –2.139032000 | –2.304112000 |
| C | –0.660911000 | –1.297758000 | –1.399596000 |
| C | –1.981792000 | –1.659495000 | –1.063183000 |
| C | –2.560400000 | –2.837163000 | –1.524637000 |
| C | 1.454346000 | 0.076513000 | –1.399596000 |
| C | 2.428061000 | –0.886535000 | –1.063183000 |
| C | 3.737256000 | –0.798790000 | –1.524637000 |
| C | 4.125769000 | 0.239749000 | –2.370669000 |
| C | 3.165457000 | 1.157498000 | –2.774856000 |
| C | 1.852456000 | 1.069516000 | –2.304112000 |
| C | –2.585151000 | 2.162617000 | –2.774856000 |
| C | –1.852456000 | 1.069516000 | –2.304112000 |
| C | –0.793436000 | 1.221244000 | –1.399596000 |
| C | –0.446268000 | 2.546030000 | –1.063183000 |
| C | –1.176856000 | 3.635953000 | –1.524637000 |
| C | –2.270513000 | 3.453146000 | –2.370669000 |
| C | 0.000000000 | 0.000000000 | –0.845722000 |
| H | 2.305711000 | 4.620604000 | 2.726166000 |
| H | 3.580109000 | 3.079018000 | 1.221401000 |
| H | 2.592070000 | 1.012755000 | 0.440650000 |
| H | –0.993940000 | 1.890026000 | 2.665372000 |
| H | 0.016790000 | 3.946364000 | 3.467955000 |
| H | –0.418963000 | –2.751176000 | 0.440650000 |
| H | 0.876453000 | –4.639974000 | 1.221401000 |
| H | 2.848705000 | –4.307106000 | 2.726166000 |
| H | 3.409257000 | –1.987723000 | 3.467955000 |
| H | 2.133780000 | –0.084236000 | 2.665372000 |
| H | –3.426047000 | –1.958642000 | 3.467955000 |
| H | –1.139841000 | –1.805790000 | 2.665372000 |
| H | –2.173106000 | 1.738421000 | 0.440650000 |
| H | –4.456562000 | 1.560956000 | 1.221401000 |
| H | –5.154416000 | –0.313498000 | 2.726166000 |
| H | –2.305711000 | –4.620604000 | –2.726166000 |
| H | –0.016790000 | –3.946364000 | –3.467955000 |
| H | 0.993940000 | –1.890026000 | –2.665372000 |
| H | –2.592070000 | –1.012755000 | –0.440650000 |
| H | –3.580109000 | –3.079018000 | –1.221401000 |
| H | 2.173106000 | –1.738421000 | –0.440650000 |
| H | 4.456562000 | –1.560956000 | –1.221401000 |
| H | 5.154416000 | 0.313498000 | –2.726166000 |
| H | 3.426047000 | 1.958642000 | –3.467955000 |
| H | 1.139841000 | 1.805790000 | –2.665372000 |
| H | –3.409257000 | 1.987723000 | –3.467955000 |
| H | –2.133780000 | 0.084236000 | –2.665372000 |
| H | 0.418963000 | 2.751176000 | –0.440650000 |
| H | –0.876453000 | 4.639974000 | –1.221401000 |
| H | –2.848705000 | 4.307106000 | –2.726166000 |

Me-**1**$_2$

B3LYP

| | | | |
|---|---|---|---|
| C | –4.072567000 | –0.030376000 | 2.550822000 |
| C | –3.717729000 | 0.948367000 | 1.607861000 |
| C | –2.424343000 | 0.942186000 | 1.079978000 |
| C | –1.461706000 | –0.028315000 | 1.440499000 |
| C | –1.848413000 | –0.957882000 | 2.415530000 |
| C | –3.140710000 | –0.976962000 | 2.973349000 |
| C | 0.706332000 | 1.280032000 | 1.440499000 |
| C | 2.028129000 | 1.628449000 | 1.079978000 |
| C | 2.680174000 | 2.745464000 | 1.607861000 |
| C | 2.009977000 | 3.542134000 | 2.550822000 |
| C | 0.724280000 | 3.208416000 | 2.973349000 |
| C | 0.094656000 | 2.079714000 | 2.415530000 |
| C | 2.416429000 | –2.231453000 | 2.973349000 |
| C | 1.753757000 | –1.121832000 | 2.415530000 |
| C | 0.755375000 | –1.251717000 | 1.440499000 |
| C | 0.396214000 | –2.570636000 | 1.079978000 |
| C | 1.037555000 | –3.693831000 | 1.607861000 |
| C | 2.062590000 | –3.511758000 | 2.550822000 |
| C | 0.000000000 | 0.000000000 | 0.864097000 |
| C | 4.072567000 | 0.030376000 | –2.550822000 |
| C | 3.140710000 | 0.976962000 | –2.973349000 |
| C | 1.848413000 | 0.957882000 | –2.415530000 |
| C | 1.461706000 | 0.028315000 | –1.440499000 |
| C | 2.424343000 | –0.942186000 | –1.079978000 |
| C | 3.717729000 | –0.948367000 | –1.607861000 |
| C | –0.755375000 | 1.251717000 | –1.440499000 |
| C | –0.396214000 | 2.570636000 | –1.079978000 |
| C | –1.037555000 | 3.693831000 | –1.607861000 |
| C | –2.062590000 | 3.511758000 | –2.550822000 |
| C | –2.416429000 | 2.231453000 | –2.973349000 |
| C | –1.753757000 | 1.121832000 | –2.415530000 |
| C | –0.724280000 | –3.208416000 | –2.973349000 |
| C | –0.094656000 | –2.079714000 | –2.415530000 |
| C | –0.706332000 | –1.280032000 | –1.440499000 |
| C | –2.028129000 | –1.628449000 | –1.079978000 |
| C | –2.680174000 | –2.745464000 | –1.607861000 |
| C | –2.009977000 | –3.542134000 | –2.550822000 |
| C | 0.000000000 | 0.000000000 | –0.864097000 |
| C | –4.719805000 | 1.998012000 | 1.184939000 |
| C | –3.490631000 | –2.015317000 | 4.014287000 |
| C | 4.090232000 | 3.088465000 | 1.184939000 |
| C | 0.000000000 | 4.030634000 | 4.014287000 |
| C | 3.490631000 | –2.015317000 | 4.014287000 |
| C | 0.629573000 | –5.086477000 | 1.184939000 |
| C | 3.490631000 | 2.015317000 | –4.014287000 |
| C | 4.719805000 | –1.998012000 | –1.184939000 |
| C | –0.629573000 | 5.086477000 | –1.184939000 |
| C | –3.490631000 | 2.015317000 | –4.014287000 |
| C | 0.000000000 | –4.030634000 | –4.014287000 |
| C | –4.090232000 | –3.088465000 | –1.184939000 |
| H | –5.083342000 | –0.038010000 | 2.970549000 |
| H | –2.165570000 | 1.727112000 | 0.377290000 |
| H | –1.140145000 | –1.702370000 | 2.771309000 |
| H | 2.578508000 | 1.011882000 | 0.377290000 |
| H | 2.508753000 | 4.421308000 | 2.970549000 |
| H | –0.904223000 | 1.838579000 | 2.771309000 |
| H | 2.044368000 | –0.136210000 | 2.771309000 |
| H | –0.412938000 | –2.738995000 | 0.377290000 |
| H | 2.574589000 | –4.383298000 | 2.970549000 |
| H | 5.083342000 | 0.038010000 | –2.970549000 |
| H | 1.140145000 | 1.702370000 | –2.771309000 |
| H | 2.165570000 | –1.727112000 | –0.377290000 |
| H | 0.412938000 | 2.738995000 | –0.377290000 |
| H | –2.574589000 | 4.383298000 | –2.970549000 |
| H | –2.044368000 | 0.136210000 | –2.771309000 |
| H | 0.904223000 | –1.838579000 | –2.771309000 |
| H | –2.578508000 | –1.011882000 | –0.377290000 |
| H | –2.508753000 | –4.421308000 | –2.970549000 |
| H | –4.306232000 | 2.662794000 | 0.412784000 |
| H | –5.030981000 | 2.622625000 | 2.040012000 |
| H | –5.635319000 | 1.536095000 | 0.777947000 |
| H | –4.530827000 | –1.910360000 | 4.357355000 |
| H | –2.834254000 | –1.932567000 | 4.897117000 |
| H | –3.366388000 | –3.037787000 | 3.619517000 |
| H | 4.459163000 | 2.397910000 | 0.412784000 |
| H | 4.786750000 | 3.045645000 | 2.040012000 |
| H | 4.147957000 | 4.112281000 | 0.777947000 |
| H | 0.610993000 | 4.878992000 | 4.357355000 |
| H | –0.256525000 | 3.420819000 | 4.897117000 |
| H | –0.947607000 | 4.434271000 | 3.619517000 |
| H | 3.919834000 | –2.968631000 | 4.357355000 |
| H | 3.090779000 | –1.488252000 | 4.897117000 |
| H | 4.313995000 | –1.396484000 | 3.619517000 |
| H | –0.152931000 | –5.060703000 | 0.412784000 |
| H | 0.244231000 | –5.668270000 | 2.040012000 |
| H | 1.487362000 | –5.648377000 | 0.777947000 |
| H | 2.834254000 | 1.932567000 | –4.897117000 |
| H | 4.530827000 | 1.910360000 | –4.357355000 |
| H | 3.366388000 | 3.037787000 | –3.619517000 |
| H | 5.635319000 | –1.536095000 | –0.777947000 |
| H | 5.030981000 | –2.622625000 | –2.040012000 |
| H | 4.306232000 | –2.662794000 | –0.412784000 |
| H | –1.487362000 | 5.648377000 | –0.777947000 |
| H | –0.244231000 | 5.668270000 | –2.040012000 |
| H | 0.152931000 | 5.060703000 | –0.412784000 |
| H | –3.090779000 | 1.488252000 | –4.897117000 |
| H | –3.919834000 | 2.968631000 | –4.357355000 |
| H | –4.313995000 | 1.396484000 | –3.619517000 |
| H | 0.256525000 | –3.420819000 | –4.897117000 |
| H | –0.610993000 | –4.878992000 | –4.357355000 |
| H | 0.947607000 | –4.434271000 | –3.619517000 |
| H | –4.147957000 | –4.112281000 | –0.777947000 |
| H | –4.786750000 | –3.045645000 | –2.040012000 |
| H | –4.459163000 | –2.397910000 | –0.412784000 |

B3LYP-D3(BJ)

| | | | |
|---|---|---|---|
| C | –4.161896000 | –0.052619000 | 2.231077000 |
| C | –3.707999000 | 0.999915000 | 1.420453000 |
| C | –2.369296000 | 1.023440000 | 1.032092000 |
| C | –1.460329000 | 0.006453000 | 1.394620000 |
| C | –1.935199000 | –0.981774000 | 2.262330000 |
| C | –3.277037000 | –1.032888000 | 2.677539000 |
| C | 0.735753000 | 1.261455000 | 1.394620000 |
| C | 2.070973000 | 1.540150000 | 1.032092000 |

| | | | |
|---|---|---|---|
| C | 2.719952000 | 2.711264000 | 1.420453000 |
| C | 2.035378000 | 3.630617000 | 2.231077000 |
| C | 0.744011000 | 3.354442000 | 2.677539000 |
| C | 0.117358000 | 2.166819000 | 2.262330000 |
| C | 2.533026000 | –2.321553000 | 2.677539000 |
| C | 1.817841000 | –1.185045000 | 2.262330000 |
| C | 0.724576000 | –1.267908000 | 1.394620000 |
| C | 0.298323000 | –2.563590000 | 1.032092000 |
| C | 0.988047000 | –3.711179000 | 1.420453000 |
| C | 2.126517000 | –3.577998000 | 2.231077000 |
| C | 0.000000000 | 0.000000000 | 0.862265000 |
| C | 4.161896000 | 0.052619000 | –2.231077000 |
| C | 3.277037000 | 1.032888000 | –2.677539000 |
| C | 1.935199000 | 0.981774000 | –2.262330000 |
| C | 1.460329000 | –0.006453000 | –1.394620000 |
| C | 2.369296000 | –1.023440000 | –1.032092000 |
| C | 3.707999000 | –0.999915000 | –1.420453000 |
| C | –0.724576000 | 1.267908000 | –1.394620000 |
| C | –0.298323000 | 2.563590000 | –1.032092000 |
| C | –0.988047000 | 3.711179000 | –1.420453000 |
| C | –2.126517000 | 3.577998000 | –2.231077000 |
| C | –2.533026000 | 2.321553000 | –2.677539000 |
| C | –1.817841000 | 1.185045000 | –2.262330000 |
| C | –0.744011000 | –3.354442000 | –2.677539000 |
| C | –0.117358000 | –2.166819000 | –2.262330000 |
| C | –0.735753000 | –1.261455000 | –1.394620000 |
| C | –2.070973000 | –1.540150000 | –1.032092000 |
| C | –2.719952000 | –2.711264000 | –1.420453000 |
| C | –2.035378000 | –3.630617000 | –2.231077000 |
| C | 0.000000000 | 0.000000000 | –0.862265000 |
| C | –4.647380000 | 2.093622000 | 0.974780000 |
| C | –3.734489000 | –2.156108000 | 3.575657000 |
| C | 4.136820000 | 2.977938000 | 0.974780000 |
| C | 0.000000000 | 4.312217000 | 3.575657000 |
| C | 3.734489000 | –2.156108000 | 3.575657000 |
| C | 0.510560000 | –5.071560000 | 0.974780000 |
| C | 3.734489000 | 2.156108000 | –3.575657000 |
| C | 4.647380000 | –2.093622000 | –0.974780000 |
| C | –0.510560000 | 5.071560000 | –0.974780000 |
| C | –3.734489000 | 2.156108000 | –3.575657000 |
| C | 0.000000000 | –4.312217000 | –3.575657000 |
| C | –4.136820000 | –2.977938000 | –0.974780000 |
| H | –5.213093000 | –0.092981000 | 2.531180000 |
| H | –2.039287000 | 1.856695000 | 0.424424000 |
| H | –1.265663000 | –1.758868000 | 2.621915000 |
| H | 2.627588000 | 0.837727000 | 0.424424000 |
| H | 2.526022000 | 4.561161000 | 2.531180000 |
| H | –0.890392000 | 1.975530000 | 2.621915000 |
| H | 2.156056000 | –0.216663000 | 2.621915000 |
| H | –0.588301000 | –2.694422000 | 0.424424000 |
| H | 2.687070000 | –4.468180000 | 2.531180000 |
| H | 5.213093000 | 0.092981000 | –2.531180000 |
| H | 1.265663000 | 1.758868000 | –2.621915000 |
| H | 2.039287000 | –1.856695000 | –0.424424000 |
| H | 0.588301000 | 2.694422000 | –0.424424000 |
| H | –2.687070000 | 4.468180000 | –2.531180000 |
| H | –2.156056000 | 0.216663000 | –2.621915000 |
| H | 0.890392000 | –1.975530000 | –2.621915000 |
| H | –2.627588000 | –0.837727000 | –0.424424000 |
| H | –2.526022000 | –4.561161000 | –2.531180000 |
| H | –4.189774000 | 2.713080000 | 0.189661000 |
| H | –4.920070000 | 2.755168000 | 1.815363000 |
| H | –5.586559000 | 1.675264000 | 0.577359000 |
| H | –4.797774000 | –2.056568000 | 3.839027000 |
| H | –3.152583000 | –2.179702000 | 4.511981000 |
| H | –3.596197000 | –3.135505000 | 3.087635000 |
| H | 4.444483000 | 2.271910000 | 0.189661000 |
| H | 4.846080000 | 2.883321000 | 1.815363000 |
| H | 4.244101000 | 4.000470000 | 0.577359000 |
| H | 0.617847000 | 5.183278000 | 3.839027000 |
| H | –0.311386000 | 3.820068000 | 4.511981000 |
| H | –0.917328000 | 4.682151000 | 3.087635000 |
| H | 4.179927000 | –3.126710000 | 3.839027000 |
| H | 3.463969000 | –1.640366000 | 4.511981000 |
| H | 4.513525000 | –1.546646000 | 3.087635000 |
| H | –0.254709000 | –4.984990000 | 0.189661000 |
| H | 0.073989000 | –5.638489000 | 1.815363000 |
| H | 1.342458000 | –5.675734000 | 0.577359000 |
| H | 3.152583000 | 2.179702000 | –4.511981000 |
| H | 4.797774000 | 2.056568000 | –3.839027000 |
| H | 3.596197000 | 3.135505000 | –3.087635000 |
| H | 5.586559000 | –1.675264000 | –0.577359000 |
| H | 4.920070000 | –2.755168000 | –1.815363000 |
| H | 4.189774000 | –2.713080000 | –0.189661000 |
| H | –1.342458000 | 5.675734000 | –0.577359000 |
| H | –0.073989000 | 5.638489000 | –1.815363000 |
| H | 0.254709000 | 4.984990000 | –0.189661000 |
| H | –3.463969000 | 1.640366000 | –4.511981000 |
| H | –4.179927000 | 3.126710000 | –3.839027000 |
| H | –4.513525000 | 1.546646000 | –3.087635000 |
| H | 0.311386000 | –3.820068000 | –4.511981000 |
| H | –0.617847000 | –5.183278000 | –3.839027000 |
| H | 0.917328000 | –4.682151000 | –3.087635000 |
| H | –4.244101000 | –4.000470000 | –0.577359000 |
| H | –4.846080000 | –2.883321000 | –1.815363000 |
| H | –4.444483000 | –2.271910000 | –0.189661000 |

M06-2X

| | | | |
|---|---|---|---|
| C | –4.171129000 | –0.055195000 | 2.195095000 |
| C | –3.705539000 | 1.008146000 | 1.410201000 |
| C | –2.365936000 | 1.035926000 | 1.037317000 |
| C | –1.461477000 | 0.011178000 | 1.389914000 |
| C | –1.948767000 | –0.983236000 | 2.239669000 |
| C | –3.294381000 | –1.037865000 | 2.637405000 |
| C | 0.740419000 | 1.260088000 | 1.389914000 |
| C | 2.080106000 | 1.530998000 | 1.037317000 |
| C | 2.725850000 | 2.705017000 | 1.410201000 |
| C | 2.037764000 | 3.639901000 | 2.195095000 |
| C | 0.748373000 | 3.371950000 | 2.637405000 |
| C | 0.122876000 | 2.179300000 | 2.239669000 |
| C | 2.546008000 | –2.334085000 | 2.637405000 |
| C | 1.825891000 | –1.196063000 | 2.239669000 |
| C | 0.721058000 | –1.271266000 | 1.389914000 |
| C | 0.285830000 | –2.566924000 | 1.037317000 |
| C | 0.979689000 | –3.713164000 | 1.410201000 |
| C | 2.133364000 | –3.584706000 | 2.195095000 |
| C | 0.000000000 | 0.000000000 | 0.856065000 |
| C | 4.171129000 | 0.055195000 | –2.195095000 |
| C | 3.294381000 | 1.037865000 | –2.637405000 |
| C | 1.948767000 | 0.983236000 | –2.239669000 |
| C | 1.461477000 | –0.011178000 | –1.389914000 |
| C | 2.365936000 | –1.035926000 | –1.037317000 |
| C | 3.705539000 | –1.008146000 | –1.410201000 |
| C | –0.721058000 | 1.271266000 | –1.389914000 |
| C | –0.285830000 | 2.566924000 | –1.037317000 |
| C | –0.979689000 | 3.713164000 | –1.410201000 |
| C | –2.133364000 | 3.584706000 | –2.195095000 |
| C | –2.546008000 | 2.334085000 | –2.637405000 |
| C | –1.825891000 | 1.196063000 | –2.239669000 |
| C | –0.748373000 | –3.371950000 | –2.637405000 |
| C | –0.122876000 | –2.179300000 | –2.239669000 |
| C | –0.740419000 | –1.260088000 | –1.389914000 |
| C | –2.080106000 | –1.530998000 | –1.037317000 |
| C | –2.725850000 | –2.705017000 | –1.410201000 |
| C | –2.037764000 | –3.639901000 | –2.195095000 |
| C | 0.000000000 | 0.000000000 | –0.856065000 |
| C | –4.640157000 | 2.108506000 | 0.977375000 |
| C | –3.760832000 | –2.171317000 | 3.514838000 |
| C | 4.146098000 | 2.964241000 | 0.977375000 |
| C | 0.000000000 | 4.342635000 | 3.514838000 |
| C | 3.760832000 | –2.171317000 | 3.514838000 |
| C | 0.494059000 | –5.072747000 | 0.977375000 |
| C | 3.760832000 | 2.171317000 | –3.514838000 |
| C | 4.640157000 | –2.108506000 | –0.977375000 |
| C | –0.494059000 | 5.072747000 | –0.977375000 |
| C | –3.760832000 | 2.171317000 | –3.514838000 |
| C | 0.000000000 | –4.342635000 | –3.514838000 |
| C | –4.146098000 | –2.964241000 | –0.977375000 |
| H | –5.225881000 | –0.097575000 | 2.481786000 |
| H | –2.033446000 | 1.888586000 | 0.453005000 |
| H | –1.289900000 | –1.766142000 | 2.609052000 |
| H | 2.652287000 | 0.816723000 | 0.453005000 |
| H | 2.528438000 | 4.574533000 | 2.481786000 |
| H | –0.884574000 | 2.000157000 | 2.609052000 |
| H | 2.174474000 | –0.234015000 | 2.609052000 |
| H | –0.618841000 | –2.705309000 | 0.453005000 |
| H | 2.697442000 | –4.476958000 | 2.481786000 |
| H | 5.225881000 | 0.097575000 | –2.481786000 |
| H | 1.289900000 | 1.766142000 | –2.609052000 |
| H | 2.033446000 | –1.888586000 | –0.453005000 |
| H | 0.618841000 | 2.705309000 | –0.453005000 |
| H | –2.697442000 | 4.476958000 | –2.481786000 |
| H | –2.174474000 | 0.234015000 | –2.609052000 |
| H | 0.884574000 | –2.000157000 | –2.609052000 |
| H | –2.652287000 | –0.816723000 | –0.453005000 |
| H | –2.528438000 | –4.574533000 | –2.481786000 |
| H | –4.159224000 | 2.764863000 | 0.238909000 |
| H | –4.951421000 | 2.723972000 | 1.835824000 |
| H | –5.553731000 | 1.693743000 | 0.525940000 |
| H | –4.822507000 | –2.066888000 | 3.774729000 |
| H | –3.180608000 | –2.208824000 | 4.448909000 |
| H | –3.625666000 | –3.139427000 | 3.008658000 |
| H | 4.474054000 | 2.219562000 | 0.238909000 |
| H | 4.834740000 | 2.926071000 | 1.835824000 |
| H | 4.243690000 | 3.962801000 | 0.525940000 |

| | | | |
|---|---|---|---|
| H | 0.621276000 | 5.209857000 | 3.774729000 |
| H | –0.322593000 | 3.858899000 | 4.448909000 |
| H | –0.905991000 | 4.709632000 | 3.008658000 |
| H | 4.201231000 | –3.142970000 | 3.774729000 |
| H | 3.503202000 | –1.650076000 | 4.448909000 |
| H | 4.531656000 | –1.570205000 | 3.008658000 |
| H | –0.314830000 | –4.984425000 | 0.238909000 |
| H | 0.116682000 | –5.650043000 | 1.835824000 |
| H | 1.310042000 | –5.656544000 | 0.525940000 |
| H | 3.180608000 | 2.208824000 | –4.448909000 |
| H | 4.822507000 | 2.066888000 | –3.774729000 |
| H | 3.625666000 | 3.139427000 | –3.008658000 |
| H | 5.553731000 | –1.693743000 | –0.525940000 |
| H | 4.951421000 | –2.723972000 | –1.835824000 |
| H | 4.159224000 | –2.764863000 | –0.238909000 |
| H | –1.310042000 | 5.656544000 | –0.525940000 |
| H | –0.116682000 | 5.650043000 | –1.835824000 |
| H | 0.314830000 | 4.984425000 | –0.238909000 |
| H | –3.503202000 | 1.650076000 | –4.448909000 |
| H | –4.201231000 | 3.142970000 | –3.774729000 |
| H | –4.531656000 | 1.570205000 | –3.008658000 |
| H | 0.322593000 | –3.858899000 | –4.448909000 |
| H | –0.621276000 | –5.209857000 | –3.774729000 |
| H | 0.905991000 | –4.709632000 | –3.008658000 |
| H | –4.243690000 | –3.962801000 | –0.525940000 |
| H | –4.834740000 | –2.926071000 | –1.835824000 |
| H | –4.474054000 | –2.219562000 | –0.238909000 |

$^{i}$Pr-**1**$_2$

B3LYP

| | | | |
|---|---|---|---|
| C | –3.935112000 | –0.521870000 | 2.770405000 |
| C | –3.797856000 | 0.421377000 | 1.739988000 |
| C | –2.554958000 | 0.545727000 | 1.104413000 |
| C | –1.440164000 | –0.248990000 | 1.458320000 |
| C | –1.630718000 | –1.167191000 | 2.504357000 |
| C | –2.860859000 | –1.316498000 | 3.169400000 |
| C | 0.504450000 | 1.371713000 | 1.458320000 |
| C | 1.750093000 | 1.939795000 | 1.104413000 |
| C | 2.263851000 | 3.078351000 | 1.739988000 |
| C | 1.515603000 | 3.668842000 | 2.770405000 |
| C | 0.290309000 | 3.135825000 | 3.169400000 |
| C | –0.195458000 | 1.995839000 | 2.504357000 |
| C | 2.570550000 | –1.819328000 | 3.169400000 |
| C | 1.826176000 | –0.828647000 | 2.504357000 |
| C | 0.935713000 | –1.122723000 | 1.458320000 |
| C | 0.804865000 | –2.485522000 | 1.104413000 |
| C | 1.534005000 | –3.499728000 | 1.739988000 |
| C | 2.419509000 | –3.146972000 | 2.770405000 |
| C | 0.000000000 | 0.000000000 | 0.853480000 |
| C | 3.935112000 | 0.521870000 | –2.770405000 |
| C | 2.860859000 | 1.316498000 | –3.169400000 |
| C | 1.630718000 | 1.167191000 | –2.504357000 |
| C | 1.440164000 | 0.248990000 | –1.458320000 |
| C | 2.554958000 | –0.545727000 | –1.104413000 |
| C | 3.797856000 | –0.421377000 | –1.739988000 |
| C | –0.935713000 | 1.122723000 | –1.458320000 |
| C | –0.804865000 | 2.485522000 | –1.104413000 |
| C | –1.534005000 | 3.499728000 | –1.739988000 |
| C | –2.419509000 | 3.146972000 | –2.770405000 |
| C | –2.570550000 | 1.819328000 | –3.169400000 |
| C | –1.826176000 | 0.828647000 | –2.504357000 |
| C | –0.290309000 | –3.135825000 | –3.169400000 |
| C | 0.195458000 | –1.995839000 | –2.504357000 |
| C | –0.504450000 | –1.371713000 | –1.458320000 |
| C | –1.750093000 | –1.939795000 | –1.104413000 |
| C | –2.263851000 | –3.078351000 | –1.739988000 |
| C | –1.515603000 | –3.668842000 | –2.770405000 |
| C | 0.000000000 | 0.000000000 | –0.853480000 |
| C | –3.608023000 | –3.702903000 | –1.365016000 |
| C | –3.435871000 | –4.820681000 | –0.319308000 |
| C | –4.660720000 | –2.690868000 | –0.899388000 |
| C | 0.469706000 | –3.744178000 | –4.344258000 |
| C | 1.906232000 | –4.153362000 | –3.979414000 |
| C | 0.458579000 | –2.804603000 | –5.564625000 |
| C | 3.608023000 | 3.702903000 | 1.365016000 |
| C | 4.660720000 | 2.690868000 | 0.899388000 |
| C | 3.435871000 | 4.820681000 | 0.319308000 |
| C | –0.469706000 | 3.744178000 | 4.344258000 |
| C | –1.906232000 | 4.153362000 | 3.979414000 |
| C | –0.458579000 | 2.804603000 | 5.564625000 |
| C | –1.402797000 | 4.976091000 | –1.365016000 |
| C | 0.000000000 | 5.381736000 | –0.899388000 |
| C | –2.456897000 | 5.385892000 | –0.319308000 |
| C | –3.477406000 | 1.465311000 | –4.344258000 |
| C | –4.550033000 | 0.425836000 | –3.979414000 |
| C | –2.658147000 | 1.005160000 | –5.564625000 |
| C | 3.477406000 | –1.465311000 | 4.344258000 |
| C | 2.658147000 | –1.005160000 | 5.564625000 |
| C | 4.550033000 | –0.425836000 | 3.979414000 |
| C | 1.402797000 | –4.976091000 | 1.365016000 |
| C | 2.456897000 | –5.385892000 | 0.319308000 |
| C | 0.000000000 | –5.381736000 | 0.899388000 |
| C | –5.010819000 | 1.273188000 | 1.365016000 |
| C | –4.660720000 | 2.690868000 | 0.899388000 |
| C | –5.892768000 | 0.565211000 | 0.319308000 |
| C | –3.007700000 | –2.278866000 | 4.344258000 |
| C | –2.199568000 | –1.799443000 | 5.564625000 |
| C | –2.643801000 | –3.727526000 | 3.979414000 |
| C | 3.007700000 | 2.278866000 | –4.344258000 |
| C | 2.199568000 | 1.799443000 | –5.564625000 |
| C | 2.643801000 | 3.727526000 | –3.979414000 |
| C | 5.010819000 | –1.273188000 | –1.365016000 |
| C | 5.892768000 | –0.565211000 | –0.319308000 |
| C | 4.660720000 | –2.690868000 | –0.899388000 |
| H | –4.896098000 | –0.617245000 | 3.286210000 |
| H | –2.446034000 | 1.303472000 | 0.337024000 |
| H | –0.797706000 | –1.774400000 | 2.845219000 |
| H | 2.351856000 | 1.466591000 | 0.337024000 |
| H | 1.913499000 | 4.548767000 | 3.286210000 |
| H | –1.137822000 | 1.578033000 | 2.845219000 |
| H | 1.935528000 | 0.196366000 | 2.845219000 |
| H | 0.094177000 | –2.770063000 | 0.337024000 |
| H | 2.982598000 | –3.931523000 | 3.286210000 |
| H | 4.896098000 | 0.617245000 | –3.286210000 |
| H | 0.797706000 | 1.774400000 | –2.845219000 |
| H | 2.446034000 | –1.303472000 | –0.337024000 |
| H | –0.094177000 | 2.770063000 | –0.337024000 |
| H | –2.982598000 | 3.931523000 | –3.286210000 |
| H | –1.935528000 | –0.196366000 | –2.845219000 |
| H | 1.137822000 | –1.578033000 | –2.845219000 |
| H | –2.351856000 | –1.466591000 | –0.337024000 |
| H | –1.913499000 | –4.548767000 | –3.286210000 |
| H | –3.991556000 | –4.178149000 | –2.286228000 |
| H | –3.053520000 | –4.410317000 | 0.629618000 |
| H | –2.727278000 | –5.590831000 | –0.661577000 |
| H | –4.401489000 | –5.312763000 | –0.113464000 |
| H | –4.388937000 | –2.230174000 | 0.063304000 |
| H | –5.633153000 | –3.190812000 | –0.759295000 |
| H | –4.798658000 | –1.881973000 | –1.632970000 |
| H | –0.072895000 | –4.662617000 | –4.629939000 |
| H | 1.920287000 | –4.849365000 | –3.125754000 |
| H | 2.519358000 | –3.277353000 | –3.711328000 |
| H | 2.395963000 | –4.651344000 | –4.832729000 |
| H | –0.571043000 | –2.550139000 | –5.863081000 |
| H | 0.957479000 | –3.277292000 | –6.427354000 |
| H | 0.987298000 | –1.861752000 | –5.345775000 |
| H | 3.991556000 | 4.178149000 | 2.286228000 |
| H | 4.388937000 | 2.230174000 | –0.063304000 |
| H | 4.798658000 | 1.881973000 | 1.632970000 |
| H | 5.633153000 | 3.190812000 | 0.759295000 |
| H | 3.053520000 | 4.410317000 | –0.629618000 |
| H | 4.401489000 | 5.312763000 | 0.113464000 |
| H | 2.727278000 | 5.590831000 | 0.661577000 |
| H | 0.072895000 | 4.662617000 | 4.629939000 |
| H | –1.920287000 | 4.849365000 | 3.125754000 |
| H | –2.395963000 | 4.651344000 | 4.832729000 |
| H | –2.519358000 | 3.277353000 | 3.711328000 |
| H | 0.571043000 | 2.550139000 | 5.863081000 |
| H | –0.987298000 | 1.861752000 | 5.345775000 |
| H | –0.957479000 | 3.277292000 | 6.427354000 |
| H | –1.622605000 | 5.545863000 | –2.286228000 |
| H | 0.263081000 | 4.916018000 | 0.063304000 |
| H | 0.053252000 | 6.473859000 | –0.759295000 |
| H | 0.769492000 | 5.096746000 | –1.632970000 |
| H | –2.292686000 | 4.849584000 | 0.629618000 |
| H | –3.478162000 | 5.157307000 | –0.661577000 |
| H | –2.400243000 | 6.468183000 | –0.113464000 |
| H | –4.001498000 | 2.394438000 | –4.629939000 |
| H | –5.159817000 | 0.761665000 | –3.125754000 |
| H | –4.097950000 | –0.543152000 | –3.711328000 |
| H | –5.226164000 | 0.250708000 | –4.832729000 |
| H | –1.922963000 | 1.769607000 | –5.863081000 |
| H | –3.316957000 | 0.809445000 | –6.427354000 |
| H | –2.105973000 | 0.075851000 | –5.345775000 |
| H | 4.001498000 | –2.394438000 | 4.629939000 |
| H | 1.922963000 | –1.769607000 | 5.863081000 |
| H | 2.105973000 | –0.075851000 | 5.345775000 |
| H | 3.316957000 | –0.809445000 | 6.427354000 |
| H | 5.159817000 | –0.761665000 | 3.125754000 |
| H | 5.226164000 | –0.250708000 | 4.832729000 |
| H | 4.097950000 | 0.543152000 | 3.711328000 |
| H | 1.622605000 | –5.545863000 | 2.286228000 |
| H | 2.292686000 | –4.849584000 | –0.629618000 |
| H | 2.400243000 | –6.468183000 | 0.113464000 |

| | | | |
|---|---|---|---|
| H | 3.478162000 | –5.157307000 | 0.661577000 |
| H | –0.263081000 | –4.916018000 | –0.063304000 |
| H | –0.769492000 | –5.096746000 | 1.632970000 |
| H | –0.053252000 | –6.473859000 | 0.759295000 |
| H | –5.614161000 | 1.367715000 | 2.286228000 |
| H | –4.125856000 | 2.685844000 | –0.063304000 |
| H | –4.029166000 | 3.214773000 | 1.632970000 |
| H | –5.579901000 | 3.283047000 | 0.759295000 |
| H | –5.346206000 | 0.439268000 | –0.629618000 |
| H | –6.801732000 | 1.155420000 | 0.113464000 |
| H | –6.205440000 | –0.433523000 | 0.661577000 |
| H | –4.074393000 | –2.268180000 | 4.629939000 |
| H | –2.494007000 | –0.780531000 | 5.863081000 |
| H | –1.118675000 | –1.785901000 | 5.345775000 |
| H | –2.359478000 | –2.467847000 | 6.427354000 |
| H | –3.239530000 | –4.087700000 | 3.125754000 |
| H | –2.830201000 | –4.400637000 | 4.832729000 |
| H | –1.578592000 | –3.820505000 | 3.711328000 |
| H | 4.074393000 | 2.268180000 | –4.629939000 |
| H | 2.494007000 | 0.780531000 | –5.863081000 |
| H | 2.359478000 | 2.467847000 | –6.427354000 |
| H | 1.118675000 | 1.785901000 | –5.345775000 |
| H | 3.239530000 | 4.087700000 | –3.125754000 |
| H | 1.578592000 | 3.820505000 | –3.711328000 |
| H | 2.830201000 | 4.400637000 | –4.832729000 |
| H | 5.614161000 | –1.367715000 | –2.286228000 |
| H | 5.346206000 | –0.439268000 | 0.629618000 |
| H | 6.205440000 | 0.433523000 | –0.661577000 |
| H | 6.801732000 | –1.155420000 | –0.113464000 |
| H | 4.125856000 | –2.685844000 | 0.063304000 |
| H | 5.579901000 | –3.283047000 | –0.759295000 |
| H | 4.029166000 | –3.214773000 | –1.632970000 |

B3LYP-D3(BJ)

| | | | |
|---|---|---|---|
| C | –3.934060000 | –0.562145000 | 2.682856000 |
| C | –3.778643000 | 0.405214000 | 1.677566000 |
| C | –2.529643000 | 0.548793000 | 1.069212000 |
| C | –1.425828000 | –0.254097000 | 1.421978000 |
| C | –1.622051000 | –1.190515000 | 2.441997000 |
| C | –2.861291000 | –1.356990000 | 3.080648000 |
| C | 0.492860000 | 1.361852000 | 1.421978000 |
| C | 1.740090000 | 1.916339000 | 1.069212000 |
| C | 2.240247000 | 3.069794000 | 1.677566000 |
| C | 1.480198000 | 3.688069000 | 2.682856000 |
| C | 0.255457000 | 3.156446000 | 3.080648000 |
| C | –0.219990000 | 1.999995000 | 2.441997000 |
| C | 2.605834000 | –1.799456000 | 3.080648000 |
| C | 1.842042000 | –0.809480000 | 2.441997000 |
| C | 0.932969000 | –1.107755000 | 1.421978000 |
| C | 0.789553000 | –2.465132000 | 1.069212000 |
| C | 1.538396000 | –3.475008000 | 1.677566000 |
| C | 2.453862000 | –3.125924000 | 2.682856000 |
| C | 0.000000000 | 0.000000000 | 0.831860000 |
| C | 3.934060000 | 0.562145000 | –2.682856000 |
| C | 2.861291000 | 1.356990000 | –3.080648000 |
| C | 1.622051000 | 1.190515000 | –2.441997000 |
| C | 1.425828000 | 0.254097000 | –1.421978000 |
| C | 2.529643000 | –0.548793000 | –1.069212000 |
| C | 3.778643000 | –0.405214000 | –1.677566000 |
| C | –0.932969000 | 1.107755000 | –1.421978000 |
| C | –0.789553000 | 2.465132000 | –1.069212000 |
| C | –1.538396000 | 3.475008000 | –1.677566000 |
| C | –2.453862000 | 3.125924000 | –2.682856000 |
| C | –2.605834000 | 1.799456000 | –3.080648000 |
| C | –1.842042000 | 0.809480000 | –2.441997000 |
| C | –0.255457000 | –3.156446000 | –3.080648000 |
| C | 0.219990000 | –1.999995000 | –2.441997000 |
| C | –0.492860000 | –1.361852000 | –1.421978000 |
| C | –1.740090000 | –1.916339000 | –1.069212000 |
| C | –2.240247000 | –3.069794000 | –1.677566000 |
| C | –1.480198000 | –3.688069000 | –2.682856000 |
| C | 0.000000000 | 0.000000000 | –0.831860000 |
| C | –3.578741000 | –3.675364000 | –1.275976000 |
| C | –3.376295000 | –4.799521000 | –0.246797000 |
| C | –4.582781000 | –2.645870000 | –0.752330000 |
| C | 0.542278000 | –3.773327000 | –4.217990000 |
| C | 1.940159000 | –4.216251000 | –3.760739000 |
| C | 0.633366000 | –2.807570000 | –5.411059000 |
| C | 3.578741000 | 3.675364000 | 1.275976000 |
| C | 4.582781000 | 2.645870000 | 0.752330000 |
| C | 3.376295000 | 4.799521000 | 0.246797000 |
| C | –0.542278000 | 3.773327000 | 4.217990000 |
| C | –1.940159000 | 4.216251000 | 3.760739000 |
| C | –0.633366000 | 2.807570000 | 5.411059000 |
| C | –1.393588000 | 4.936963000 | –1.275976000 |
| C | 0.000000000 | 5.291740000 | –0.752330000 |
| C | –2.468360000 | 5.323718000 | –0.246797000 |
| C | –3.538936000 | 1.417037000 | –4.217990000 |
| C | –4.621460000 | 0.427899000 | –3.760739000 |
| C | –2.748110000 | 0.855274000 | –5.411059000 |
| C | 3.538936000 | –1.417037000 | 4.217990000 |
| C | 2.748110000 | –0.855274000 | 5.411059000 |
| C | 4.621460000 | –0.427899000 | 3.760739000 |
| C | 1.393588000 | –4.936963000 | 1.275976000 |
| C | 2.468360000 | –5.323718000 | 0.246797000 |
| C | 0.000000000 | –5.291740000 | 0.752330000 |
| C | –4.972329000 | 1.261599000 | 1.275976000 |
| C | –4.582781000 | 2.645870000 | 0.752330000 |
| C | –5.844655000 | 0.524197000 | 0.246797000 |
| C | –2.996658000 | –2.356290000 | 4.217990000 |
| C | –2.114744000 | –1.952296000 | 5.411059000 |
| C | –2.681301000 | –3.788352000 | 3.760739000 |
| C | 2.996658000 | 2.356290000 | –4.217990000 |
| C | 2.114744000 | 1.952296000 | –5.411059000 |
| C | 2.681301000 | 3.788352000 | –3.760739000 |
| C | 4.972329000 | –1.261599000 | –1.275976000 |
| C | 5.844655000 | –0.524197000 | –0.246797000 |
| C | 4.582781000 | –2.645870000 | –0.752330000 |
| H | –4.903707000 | –0.675611000 | 3.177126000 |
| H | –2.407044000 | 1.324216000 | 0.325480000 |
| H | –0.790788000 | –1.799793000 | 2.780570000 |
| H | 2.350327000 | 1.422453000 | 0.325480000 |
| H | 1.866757000 | 4.584541000 | 3.177126000 |
| H | –1.163273000 | 1.584739000 | 2.780570000 |
| H | 1.954060000 | 0.215054000 | 2.780570000 |
| H | 0.056717000 | –2.746669000 | 0.325480000 |
| H | 3.036950000 | –3.908929000 | 3.177126000 |
| H | 4.903707000 | 0.675611000 | –3.177126000 |
| H | 0.790788000 | 1.799793000 | –2.780570000 |
| H | 2.407044000 | –1.324216000 | –0.325480000 |
| H | –0.056717000 | 2.746669000 | –0.325480000 |
| H | –3.036950000 | 3.908929000 | –3.177126000 |
| H | –1.954060000 | –0.215054000 | –2.780570000 |
| H | 1.163273000 | –1.584739000 | –2.780570000 |
| H | –2.350327000 | –1.422453000 | –0.325480000 |
| H | –1.866757000 | –4.584541000 | –3.177126000 |
| H | –4.004632000 | –4.131487000 | –2.187696000 |
| H | –2.939374000 | –4.392134000 | 0.677991000 |
| H | –2.695046000 | –5.576886000 | –0.624707000 |
| H | –4.337635000 | –5.275860000 | 0.007570000 |
| H | –4.258556000 | –2.211618000 | 0.206043000 |
| H | –5.563441000 | –3.118466000 | –0.583418000 |
| H | –4.720678000 | –1.819082000 | –1.464247000 |
| H | –0.005588000 | –4.671818000 | –4.550752000 |
| H | 1.877010000 | –4.932680000 | –2.927393000 |
| H | 2.536200000 | –3.354296000 | –3.419052000 |
| H | 2.489044000 | –4.696175000 | –4.587333000 |
| H | –0.368901000 | –2.507365000 | –5.755408000 |
| H | 1.163918000 | –3.275991000 | –6.256322000 |
| H | 1.181069000 | –1.891192000 | –5.135314000 |
| H | 4.004632000 | 4.131487000 | 2.187696000 |
| H | 4.258556000 | 2.211618000 | –0.206043000 |
| H | 4.720678000 | 1.819082000 | 1.464247000 |
| H | 5.563441000 | 3.118466000 | 0.583418000 |
| H | 2.939374000 | 4.392134000 | –0.677991000 |
| H | 4.337635000 | 5.275860000 | –0.007570000 |
| H | 2.695046000 | 5.576886000 | 0.624707000 |
| H | 0.005588000 | 4.671818000 | 4.550752000 |
| H | –1.877010000 | 4.932680000 | 2.927393000 |
| H | –2.489044000 | 4.696175000 | 4.587333000 |
| H | –2.536200000 | 3.354296000 | 3.419052000 |
| H | 0.368901000 | 2.507365000 | 5.755408000 |
| H | –1.181069000 | 1.891192000 | 5.135314000 |
| H | –1.163918000 | 3.275991000 | 6.256322000 |
| H | –1.575657000 | 5.533857000 | –2.187696000 |
| H | 0.213960000 | 4.793826000 | 0.206043000 |
| H | 0.081050000 | 6.377314000 | –0.583418000 |
| H | 0.784968000 | 4.997768000 | –1.464247000 |
| H | –2.334012000 | 4.741639000 | 0.677991000 |
| H | –3.482202000 | 5.122422000 | –0.624707000 |
| H | –2.400212000 | 6.394432000 | 0.007570000 |
| H | –4.043119000 | 2.340749000 | –4.550752000 |
| H | –5.210331000 | 0.840801000 | –2.927393000 |
| H | –4.173005000 | –0.519266000 | –3.419052000 |
| H | –5.311529000 | 0.192512000 | –4.587333000 |
| H | –1.986991000 | 1.573161000 | –5.755408000 |
| H | –3.419051000 | 0.630013000 | –6.256322000 |
| H | –2.228355000 | –0.077240000 | –5.135314000 |
| H | 4.043119000 | –2.340749000 | 4.550752000 |
| H | 1.986991000 | –1.573161000 | 5.755408000 |
| H | 2.228355000 | 0.077240000 | 5.135314000 |
| H | 3.419051000 | –0.630013000 | 6.256322000 |
| H | 5.210331000 | –0.840801000 | 2.927393000 |
| H | 5.311529000 | –0.192512000 | 4.587333000 |
| H | 4.173005000 | 0.519266000 | 3.419052000 |

| | | | |
|---|---|---|---|
| H | 1.575657000 | –5.533857000 | 2.187696000 |
| H | 2.334012000 | –4.741639000 | –0.677991000 |
| H | 2.400212000 | –6.394432000 | –0.007570000 |
| H | 3.482202000 | –5.122422000 | 0.624707000 |
| H | –0.213960000 | –4.793826000 | –0.206043000 |
| H | –0.784968000 | –4.997768000 | 1.464247000 |
| H | –0.081050000 | –6.377314000 | 0.583418000 |
| H | –5.580289000 | 1.402369000 | 2.187696000 |
| H | –4.044595000 | 2.582208000 | –0.206043000 |
| H | –3.935710000 | 3.178686000 | 1.464247000 |
| H | –5.482391000 | 3.258848000 | 0.583418000 |
| H | –5.273386000 | 0.349505000 | –0.677991000 |
| H | –6.737846000 | 1.118571000 | –0.007570000 |
| H | –6.177248000 | –0.454465000 | 0.624707000 |
| H | –4.048708000 | –2.331070000 | 4.550752000 |
| H | –2.355893000 | –0.934205000 | 5.755408000 |
| H | –1.047286000 | –1.968432000 | 5.135314000 |
| H | –2.255133000 | –2.645978000 | 6.256322000 |
| H | –3.333321000 | –4.091878000 | 2.927393000 |
| H | –2.822485000 | –4.503663000 | 4.587333000 |
| H | –1.636805000 | –3.873562000 | 3.419052000 |
| H | 4.048708000 | 2.331070000 | –4.550752000 |
| H | 2.355893000 | 0.934205000 | –5.755408000 |
| H | 2.255133000 | 2.645978000 | –6.256322000 |
| H | 1.047286000 | 1.968432000 | –5.135314000 |
| H | 3.333321000 | 4.091878000 | –2.927393000 |
| H | 1.636805000 | 3.873562000 | –3.419052000 |
| H | 2.822485000 | 4.503663000 | –4.587333000 |
| H | 5.580289000 | –1.402369000 | –2.187696000 |
| H | 5.273386000 | –0.349505000 | 0.677991000 |
| H | 6.177248000 | 0.454465000 | –0.624707000 |
| H | 6.737846000 | –1.118571000 | 0.007570000 |
| H | 4.044595000 | –2.582208000 | 0.206043000 |
| H | 5.482391000 | –3.258848000 | –0.583418000 |
| H | 3.935710000 | –3.178686000 | –1.464247000 |

M06-2X

| | | | |
|---|---|---|---|
| C | –2.844330000 | –2.795010000 | 2.657900000 |
| C | –3.286990000 | –1.897750000 | 1.675970000 |
| C | –2.365680000 | –1.035150000 | 1.085780000 |
| C | –0.997560000 | –1.048680000 | 1.425440000 |
| C | –0.607490000 | –1.939430000 | 2.427860000 |
| C | –1.512270000 | –2.813490000 | 3.051350000 |
| C | –0.409404000 | 1.388252000 | 1.425440000 |
| C | 0.286374000 | 2.566314000 | 1.085780000 |
| C | –0.000005000 | 3.795492000 | 1.675970000 |
| C | –0.998385000 | 3.860767000 | 2.657900000 |
| C | –1.680419000 | 2.716409000 | 3.051350000 |
| C | –1.375851000 | 1.495817000 | 2.427860000 |
| C | 3.192689000 | 0.097081000 | 3.051350000 |
| C | 1.983341000 | 0.443613000 | 2.427860000 |
| C | 1.406964000 | –0.339572000 | 1.425440000 |
| C | 2.079306000 | –1.531164000 | 1.085780000 |
| C | 3.286995000 | –1.897742000 | 1.675970000 |
| C | 3.842715000 | –1.065757000 | 2.657900000 |
| C | 0.000000000 | 0.000000000 | 0.834410000 |
| C | 2.844330000 | 2.795010000 | –2.657900000 |
| C | 1.512270000 | 2.813490000 | –3.051350000 |
| C | 0.607490000 | 1.939430000 | –2.427860000 |
| C | 0.997560000 | 1.048680000 | –1.425440000 |
| C | 2.365680000 | 1.035150000 | –1.085780000 |
| C | 3.286990000 | 1.897750000 | –1.675970000 |
| C | –1.406964000 | 0.339572000 | –1.425440000 |
| C | –2.079306000 | 1.531164000 | –1.085780000 |
| C | –3.286995000 | 1.897742000 | –1.675970000 |
| C | –3.842715000 | 1.065757000 | –2.657900000 |
| C | –3.192689000 | –0.097081000 | –3.051350000 |
| C | –1.983341000 | –0.443613000 | –2.427860000 |
| C | 1.680419000 | –2.716409000 | –3.051350000 |
| C | 1.375851000 | –1.495817000 | –2.427860000 |
| C | 0.409404000 | –1.388252000 | –1.425440000 |
| C | –0.286374000 | –2.566314000 | –1.085780000 |
| C | 0.000005000 | –3.795492000 | –1.675970000 |
| C | 0.998385000 | –3.860767000 | –2.657900000 |
| C | 0.000000000 | 0.000000000 | –0.834410000 |
| C | –0.730000000 | –5.065590000 | –1.272240000 |
| C | 0.116780000 | –5.859300000 | –0.271700000 |
| C | –2.121600000 | –4.810960000 | –0.699930000 |
| C | 2.693260000 | –2.751500000 | –4.181180000 |
| C | 4.046440000 | –2.172140000 | –3.761250000 |
| C | 2.146850000 | –2.016970000 | –5.411130000 |
| C | 0.730000000 | 5.065590000 | 1.272240000 |
| C | 2.121600000 | 4.810960000 | 0.699930000 |
| C | –0.116780000 | 5.859300000 | 0.271700000 |
| C | –2.693260000 | 2.751500000 | 4.181180000 |
| C | –4.046440000 | 2.172140000 | 3.761250000 |
| C | –2.146850000 | 2.016970000 | 5.411130000 |
| C | –4.021930000 | 3.164994000 | –1.272240000 |
| C | –3.105614000 | 4.242839000 | –0.699930000 |
| C | –5.132693000 | 2.828516000 | –0.271700000 |
| C | –3.729499000 | –0.956682000 | –4.181180000 |
| C | –3.904348000 | –2.418250000 | –3.761250000 |
| C | –2.820172000 | –0.850742000 | –5.411130000 |
| C | 3.729499000 | 0.956682000 | 4.181180000 |
| C | 2.820172000 | 0.850742000 | 5.411130000 |
| C | 3.904348000 | 2.418250000 | 3.761250000 |
| C | 4.021930000 | –3.164994000 | 1.272240000 |
| C | 5.132693000 | –2.828516000 | 0.271700000 |
| C | 3.105614000 | –4.242839000 | 0.699930000 |
| C | –4.751930000 | –1.900596000 | 1.272240000 |
| C | –5.227214000 | –0.568121000 | 0.699930000 |
| C | –5.015913000 | –3.030784000 | 0.271700000 |
| C | –1.036239000 | –3.708182000 | 4.181180000 |
| C | –0.673322000 | –2.867712000 | 5.411130000 |
| C | 0.142092000 | –4.590390000 | 3.761250000 |
| C | 1.036239000 | 3.708182000 | –4.181180000 |
| C | 0.673322000 | 2.867712000 | –5.411130000 |
| C | –0.142092000 | 4.590390000 | –3.761250000 |
| C | 4.751930000 | 1.900596000 | –1.272240000 |
| C | 5.015913000 | 3.030784000 | –0.271700000 |
| C | 5.227214000 | 0.568121000 | –0.699930000 |
| H | –3.562470000 | –3.466920000 | 3.138580000 |
| H | –2.735070000 | –0.305510000 | 0.374000000 |
| H | 0.421790000 | –1.953200000 | 2.777600000 |
| H | 1.102956000 | 2.521395000 | 0.374000000 |
| H | –1.221206000 | 4.818650000 | 3.138580000 |
| H | –1.902416000 | 0.611319000 | 2.777600000 |
| H | 1.480626000 | 1.341881000 | 2.777600000 |
| H | 1.632114000 | –2.215885000 | 0.374000000 |
| H | 4.783676000 | –1.351730000 | 3.138580000 |
| H | 3.562470000 | 3.466920000 | –3.138580000 |
| H | –0.421790000 | 1.953200000 | –2.777600000 |
| H | 2.735070000 | 0.305510000 | –0.374000000 |
| H | –1.632114000 | 2.215885000 | –0.374000000 |
| H | –4.783676000 | 1.351730000 | –3.138580000 |
| H | –1.480626000 | –1.341881000 | –2.777600000 |
| H | 1.902416000 | –0.611319000 | –2.777600000 |
| H | –1.102956000 | –2.521395000 | –0.374000000 |
| H | 1.221206000 | –4.818650000 | –3.138580000 |
| H | –0.839000000 | –5.675240000 | –2.185480000 |
| H | 0.248980000 | –5.265280000 | 0.646350000 |
| H | 1.115730000 | –6.085280000 | –0.673950000 |
| H | –0.376000000 | –6.806990000 | –0.005010000 |
| H | –2.066350000 | –4.298500000 | 0.275020000 |
| H | –2.649430000 | –5.763500000 | –0.543130000 |
| H | –2.726130000 | –4.188640000 | –1.375800000 |
| H | 2.842120000 | –3.809370000 | –4.452790000 |
| H | 4.457860000 | –2.711850000 | –2.895730000 |
| H | 3.950980000 | –1.108580000 | –3.488450000 |
| H | 4.770200000 | –2.241050000 | –4.587270000 |
| H | 1.190170000 | –2.450730000 | –5.737010000 |
| H | 2.858950000 | –2.072390000 | –6.248500000 |
| H | 1.974840000 | –0.953450000 | –5.179580000 |
| H | 0.839000000 | 5.675240000 | 2.185480000 |
| H | 2.066350000 | 4.298500000 | –0.275020000 |
| H | 2.726130000 | 4.188640000 | 1.375800000 |
| H | 2.649430000 | 5.763500000 | 0.543130000 |
| H | –0.248980000 | 5.265280000 | –0.646350000 |
| H | 0.376000000 | 6.806990000 | 0.005010000 |
| H | –1.115730000 | 6.085280000 | 0.673950000 |
| H | –2.842120000 | 3.809370000 | 4.452790000 |
| H | –4.457860000 | 2.711850000 | 2.895730000 |
| H | –4.770200000 | 2.241050000 | 4.587270000 |
| H | –3.950980000 | 1.108580000 | 3.488450000 |
| H | –1.190170000 | 2.450730000 | 5.737010000 |
| H | –1.974840000 | 0.953450000 | 5.179580000 |
| H | –2.858950000 | 2.072390000 | 6.248500000 |
| H | –4.495402000 | 3.564215000 | –2.185480000 |
| H | –2.689435000 | 3.938762000 | 0.275020000 |
| H | –3.666622000 | 5.176224000 | –0.543130000 |
| H | –2.264404000 | 4.455218000 | –1.375800000 |
| H | –4.684356000 | 2.417017000 | 0.646350000 |
| H | –5.827872000 | 2.076389000 | –0.673950000 |
| H | –5.707026000 | 3.729121000 | –0.005010000 |
| H | –4.720071000 | –0.556663000 | –4.452790000 |
| H | –4.577461000 | –2.504695000 | –2.895730000 |
| H | –2.935548000 | –2.867359000 | –3.488450000 |
| H | –4.325906000 | –3.010589000 | –4.587270000 |
| H | –2.717479000 | 0.194648000 | –5.737010000 |
| H | –3.224217000 | –1.439728000 | –6.248500000 |
| H | –1.813132000 | –1.233537000 | –5.179580000 |
| H | 4.720071000 | 0.556663000 | 4.452790000 |
| H | 2.717479000 | –0.194648000 | 5.737010000 |
| H | 1.813132000 | 1.233537000 | 5.179580000 |
| H | 3.224217000 | 1.439728000 | 6.248500000 |

| | | | |
|---|---|---|---|
| H | 4.577461000 | 2.504695000 | 2.895730000 |
| H | 4.325906000 | 3.010589000 | 4.587270000 |
| H | 2.935548000 | 2.867359000 | 3.488450000 |
| H | 4.495402000 | –3.564215000 | 2.185480000 |
| H | 4.684356000 | –2.417017000 | –0.646350000 |
| H | 5.707026000 | –3.729121000 | 0.005010000 |
| H | 5.827872000 | –2.076389000 | 0.673950000 |
| H | 2.689435000 | –3.938762000 | –0.275020000 |
| H | 2.264404000 | –4.455218000 | 1.375800000 |
| H | 3.666622000 | –5.176224000 | 0.543130000 |
| H | –5.334402000 | –2.111025000 | 2.185480000 |
| H | –4.755785000 | –0.359738000 | –0.275020000 |
| H | –4.990534000 | 0.266578000 | 1.375800000 |
| H | –6.316052000 | –0.587276000 | 0.543130000 |
| H | –4.435376000 | –2.848263000 | –0.646350000 |
| H | –6.083026000 | –3.077869000 | 0.005010000 |
| H | –4.712142000 | –4.008891000 | 0.673950000 |
| H | –1.877951000 | –4.366033000 | 4.452790000 |
| H | –1.527309000 | –2.256082000 | 5.737010000 |
| H | 0.161708000 | –2.186987000 | 5.179580000 |
| H | –0.365267000 | –3.512118000 | 6.248500000 |
| H | –0.119601000 | –5.216545000 | 2.895730000 |
| H | 0.444294000 | –5.251639000 | 4.587270000 |
| H | 1.015432000 | –3.975939000 | 3.488450000 |
| H | 1.877951000 | 4.366033000 | –4.452790000 |
| H | 1.527309000 | 2.256082000 | –5.737010000 |
| H | 0.365267000 | 3.512118000 | –6.248500000 |
| H | –0.161708000 | 2.186987000 | –5.179580000 |
| H | 0.119601000 | 5.216545000 | –2.895730000 |
| H | –1.015432000 | 3.975939000 | –3.488450000 |
| H | –0.444294000 | 5.251639000 | –4.587270000 |
| H | 5.334402000 | 2.111025000 | –2.185480000 |
| H | 4.435376000 | 2.848263000 | 0.646350000 |
| H | 4.712142000 | 4.008891000 | –0.673950000 |
| H | 6.083026000 | 3.077869000 | –0.005010000 |
| H | 4.755785000 | 0.359738000 | 0.275020000 |
| H | 6.316052000 | 0.587276000 | –0.543130000 |
| H | 4.990534000 | –0.266578000 | –1.375800000 |

$^t$Bu-**1**$_2$

B3LYP

| | | | |
|---|---|---|---|
| C | 1.413190000 | –3.701170000 | 2.788120000 |
| C | 0.504770000 | –3.800890000 | 1.728820000 |
| C | 0.089490000 | –2.613660000 | 1.094900000 |
| C | 0.572090000 | –1.345770000 | 1.466190000 |
| C | 1.476820000 | –1.308020000 | 2.547810000 |
| C | 1.897230000 | –2.459810000 | 3.227310000 |
| C | –1.451516000 | 0.177441000 | 1.466190000 |
| C | –2.308241000 | 1.229329000 | 1.094900000 |
| C | –3.544052000 | 1.463301000 | 1.728820000 |
| C | –3.911902000 | 0.626727000 | 2.788120000 |
| C | –3.078873000 | –0.413144000 | 3.227310000 |
| C | –1.871189000 | –0.624954000 | 2.547810000 |
| C | 1.181643000 | 2.872954000 | 3.227310000 |
| C | 0.394369000 | 1.932974000 | 2.547810000 |
| C | 0.879426000 | 1.168329000 | 1.466190000 |
| C | 2.218751000 | 1.384331000 | 1.094900000 |
| C | 3.039282000 | 2.337589000 | 1.728820000 |
| C | 2.498712000 | 3.074443000 | 2.788120000 |
| C | 0.000000000 | 0.000000000 | 0.853720000 |
| C | –1.413190000 | 3.701170000 | –2.788120000 |
| C | –1.897230000 | 2.459810000 | –3.227310000 |
| C | –1.476820000 | 1.308020000 | –2.547810000 |
| C | –0.572090000 | 1.345770000 | –1.466190000 |
| C | –0.089490000 | 2.613660000 | –1.094900000 |
| C | –0.504770000 | 3.800890000 | –1.728820000 |
| C | –0.879426000 | –1.168329000 | –1.466190000 |
| C | –2.218751000 | –1.384331000 | –1.094900000 |
| C | –3.039282000 | –2.337589000 | –1.728820000 |
| C | –2.498712000 | –3.074443000 | –2.788120000 |
| C | –1.181643000 | –2.872954000 | –3.227310000 |
| C | –0.394369000 | –1.932974000 | –2.547810000 |
| C | 3.078873000 | 0.413144000 | –3.227310000 |
| C | 1.871189000 | 0.624954000 | –2.547810000 |
| C | 1.451516000 | –0.177441000 | –1.466190000 |
| C | 2.308241000 | –1.229329000 | –1.094900000 |
| C | 3.544052000 | –1.463301000 | –1.728820000 |
| C | 3.911902000 | –0.626727000 | –2.788120000 |
| C | 0.000000000 | 0.000000000 | –0.853720000 |
| C | –0.094720000 | –5.160000000 | 1.302090000 |
| C | 2.834600000 | –2.402990000 | 4.453210000 |
| C | –4.421331000 | 2.662030000 | 1.302090000 |
| C | –3.498350000 | –1.253341000 | 4.453210000 |
| C | 0.663750000 | 3.656331000 | 4.453210000 |
| C | 4.516051000 | 2.497970000 | 1.302090000 |
| C | –2.834600000 | 2.402990000 | –4.453210000 |
| C | 0.094720000 | 5.160000000 | –1.302090000 |
| C | –4.516051000 | –2.497970000 | –1.302090000 |
| C | –0.663750000 | –3.656331000 | –4.453210000 |
| C | 3.498350000 | 1.253341000 | –4.453210000 |
| C | 4.421331000 | –2.662030000 | –1.302090000 |
| C | 0.625130000 | –6.352660000 | 1.960990000 |
| C | 0.000000000 | –5.339920000 | –0.227710000 |
| C | –1.580640000 | –5.203160000 | 1.725970000 |
| C | 3.259480000 | –0.966230000 | 4.804760000 |
| C | 4.112280000 | –3.228940000 | 4.178350000 |
| C | 2.100970000 | –2.996470000 | 5.679260000 |
| C | –5.814130000 | 2.634952000 | 1.960990000 |
| C | –4.624506000 | 2.669960000 | –0.227710000 |
| C | –3.715749000 | 3.970454000 | 1.725970000 |
| C | –2.466520000 | –2.339677000 | 4.804760000 |
| C | –4.852484000 | –1.946869000 | 4.178350000 |
| C | –3.645504000 | –0.321258000 | 5.679260000 |
| C | –0.792960000 | 3.305907000 | 4.804760000 |
| C | 0.740204000 | 5.175809000 | 4.178350000 |
| C | 1.544534000 | 3.317728000 | 5.679260000 |
| C | 5.189000000 | 3.717708000 | 1.960990000 |
| C | 4.624506000 | 2.669960000 | –0.227710000 |
| C | 5.296389000 | 1.232706000 | 1.725970000 |
| C | –2.100970000 | 2.996470000 | –5.679260000 |
| C | –4.112280000 | 3.228940000 | –4.178350000 |
| C | –3.259480000 | 0.966230000 | –4.804760000 |
| C | 0.000000000 | 5.339920000 | 0.227710000 |
| C | –0.625130000 | 6.352660000 | –1.960990000 |
| C | 1.580640000 | 5.203160000 | –1.725970000 |
| C | –4.624506000 | –2.669960000 | 0.227710000 |
| C | –5.189000000 | –3.717708000 | –1.960990000 |
| C | –5.296389000 | –1.232706000 | –1.725970000 |
| C | –1.544534000 | –3.317728000 | –5.679260000 |
| C | –0.740204000 | –5.175809000 | –4.178350000 |
| C | 0.792960000 | –3.305907000 | –4.804760000 |
| C | 3.645504000 | 0.321258000 | –5.679260000 |
| C | 4.852484000 | 1.946869000 | –4.178350000 |
| C | 2.466520000 | 2.339677000 | –4.804760000 |
| C | 4.624506000 | –2.669960000 | 0.227710000 |
| C | 5.814130000 | –2.634952000 | –1.960990000 |
| C | 3.715749000 | –3.970454000 | –1.725970000 |
| H | 0.514740000 | –6.353260000 | 3.056530000 |
| H | 1.700990000 | –6.364830000 | 1.723870000 |
| H | 0.191460000 | –7.295140000 | 1.590380000 |
| H | –0.514410000 | –4.538760000 | –0.776520000 |
| H | –0.460280000 | –6.296340000 | –0.526880000 |
| H | 1.051170000 | –5.355730000 | –0.556760000 |
| H | –1.680570000 | –5.118510000 | 2.820150000 |
| H | –2.045060000 | –6.154390000 | 1.414810000 |
| H | –2.151850000 | –4.381020000 | 1.269950000 |
| H | 3.926560000 | –0.980690000 | 5.681580000 |
| H | 2.395690000 | –0.331050000 | 5.055600000 |
| H | 3.804270000 | –0.485000000 | 3.978720000 |
| H | 4.773390000 | –3.217500000 | 5.061180000 |
| H | 4.675120000 | –2.815320000 | 3.326720000 |
| H | 3.881320000 | –4.280850000 | 3.949670000 |
| H | 2.750620000 | –2.961880000 | 6.569970000 |
| H | 1.810540000 | –4.046150000 | 5.518430000 |
| H | 1.184960000 | –2.426040000 | 5.903490000 |
| H | –5.759455000 | 2.730852000 | 3.056530000 |
| H | –6.362599000 | 1.709314000 | 1.723870000 |
| H | –6.413507000 | 3.481761000 | 1.590380000 |
| H | –3.673476000 | 2.714872000 | –0.776520000 |
| H | –5.222650000 | 3.546784000 | –0.526880000 |
| H | –5.163783000 | 1.767525000 | –0.556760000 |
| H | –3.592475000 | 4.014671000 | 2.820150000 |
| H | –4.307328000 | 4.848269000 | 1.414810000 |
| H | –2.718150000 | 4.054067000 | 1.269950000 |
| H | –2.812582000 | –2.910156000 | 5.681580000 |
| H | –1.484543000 | –1.909203000 | 5.055600000 |
| H | –2.322157000 | –3.052094000 | 3.978720000 |
| H | –5.173132000 | –2.525127000 | 5.061180000 |
| H | –4.775699000 | –2.641113000 | 3.326720000 |
| H | –5.647985000 | –1.220897000 | 3.949670000 |
| H | –3.940373000 | –0.901167000 | 6.569970000 |
| H | –4.409339000 | 0.455101000 | 5.518430000 |
| H | –2.693492000 | 0.186815000 | 5.903490000 |
| H | –1.113978000 | 3.890846000 | 5.681580000 |
| H | –0.911147000 | 2.240253000 | 5.055600000 |
| H | –1.482113000 | 3.537094000 | 3.978720000 |
| H | 0.399742000 | 5.742627000 | 5.061180000 |
| H | 0.100579000 | 5.456433000 | 3.326720000 |
| H | 1.766665000 | 5.501747000 | 3.949670000 |
| H | 1.189753000 | 3.863047000 | 6.569970000 |
| H | 2.598799000 | 3.591049000 | 5.518430000 |
| H | 1.508532000 | 2.239225000 | 5.903490000 |
| H | 5.244715000 | 3.622408000 | 3.056530000 |
| H | 4.661609000 | 4.655516000 | 1.723870000 |

| | | | |
|---|---|---|---|
| H | 6.222047000 | 3.813379000 | 1.590380000 |
| H | 4.187886000 | 1.823888000 | –0.776520000 |
| H | 5.682930000 | 2.749556000 | –0.526880000 |
| H | 4.112613000 | 3.588205000 | –0.556760000 |
| H | 5.273045000 | 1.103839000 | 2.820150000 |
| H | 6.352388000 | 1.306121000 | 1.414810000 |
| H | 4.870000000 | 0.326953000 | 1.269950000 |
| H | –1.810540000 | 4.046150000 | –5.518430000 |
| H | –2.750620000 | 2.961880000 | –6.569970000 |
| H | –1.184960000 | 2.426040000 | –5.903490000 |
| H | –3.881320000 | 4.280850000 | –3.949670000 |
| H | –4.675120000 | 2.815320000 | –3.326720000 |
| H | –4.773390000 | 3.217500000 | –5.061180000 |
| H | –3.804270000 | 0.485000000 | –3.978720000 |
| H | –2.395690000 | 0.331050000 | –5.055600000 |
| H | –3.926560000 | 0.980690000 | –5.681580000 |
| H | 0.460280000 | 6.296340000 | 0.526880000 |
| H | 0.514410000 | 4.538760000 | 0.776520000 |
| H | –1.051170000 | 5.355730000 | 0.556760000 |
| H | –0.191460000 | 7.295140000 | –1.590380000 |
| H | –1.700990000 | 6.364830000 | –1.723870000 |
| H | –0.514740000 | 6.353260000 | –3.056530000 |
| H | 2.045060000 | 6.154390000 | –1.414810000 |
| H | 1.680570000 | 5.118510000 | –2.820150000 |
| H | 2.151850000 | 4.381020000 | –1.269950000 |
| H | –5.682930000 | –2.749556000 | 0.526880000 |
| H | –4.187886000 | –1.823888000 | 0.776520000 |
| H | –4.112613000 | –3.588205000 | 0.556760000 |
| H | –6.222047000 | –3.813379000 | –1.590380000 |
| H | –4.661609000 | –4.655516000 | –1.723870000 |
| H | –5.244715000 | –3.622408000 | –3.056530000 |
| H | –6.352388000 | –1.306121000 | –1.414810000 |
| H | –5.273045000 | –1.103839000 | –2.820150000 |
| H | –4.870000000 | –0.326953000 | –1.269950000 |
| H | –2.598799000 | –3.591049000 | –5.518430000 |
| H | –1.189753000 | –3.863047000 | –6.569970000 |
| H | –1.508532000 | –2.239225000 | –5.903490000 |
| H | –1.766665000 | –5.501747000 | –3.949670000 |
| H | –0.100579000 | –5.456433000 | –3.326720000 |
| H | –0.399742000 | –5.742627000 | –5.061180000 |
| H | 1.482113000 | –3.537094000 | –3.978720000 |
| H | 0.911147000 | –2.240253000 | –5.055600000 |
| H | 1.113978000 | –3.890846000 | –5.681580000 |
| H | 4.409339000 | –0.455101000 | –5.518430000 |
| H | 3.940373000 | 0.901167000 | –6.569970000 |
| H | 2.693492000 | –0.186815000 | –5.903490000 |
| H | 5.647985000 | 1.220897000 | –3.949670000 |
| H | 4.775699000 | 2.641113000 | –3.326720000 |
| H | 5.173132000 | 2.525127000 | –5.061180000 |
| H | 2.322157000 | 3.052094000 | –3.978720000 |
| H | 1.484543000 | 1.909203000 | –5.055600000 |
| H | 2.812582000 | 2.910156000 | –5.681580000 |
| H | 5.222650000 | –3.546784000 | 0.526880000 |
| H | 3.673476000 | –2.714872000 | 0.776520000 |
| H | 5.163783000 | –1.767525000 | 0.556760000 |
| H | 6.413507000 | –3.481761000 | –1.590380000 |
| H | 6.362599000 | –1.709314000 | –1.723870000 |
| H | 5.759455000 | –2.730852000 | –3.056530000 |
| H | 4.307328000 | –4.848269000 | –1.414810000 |
| H | 3.592475000 | –4.014671000 | –2.820150000 |
| H | 2.718150000 | –4.054067000 | –1.269950000 |
| H | 1.738290000 | –4.604030000 | 3.302290000 |
| H | –0.649070000 | –2.687310000 | 0.304620000 |
| H | 1.844340000 | –0.348980000 | 2.891120000 |
| H | –2.002744000 | 1.905766000 | 0.304620000 |
| H | –4.856352000 | 0.796612000 | 3.302290000 |
| H | –1.224396000 | –1.422755000 | 2.891120000 |
| H | –0.619944000 | 1.771735000 | 2.891120000 |
| H | 2.651814000 | 0.781544000 | 0.304620000 |
| H | 3.118062000 | 3.807418000 | 3.302290000 |
| H | –1.738290000 | 4.604030000 | –3.302290000 |
| H | –1.844340000 | 0.348980000 | –2.891120000 |
| H | 0.649070000 | 2.687310000 | –0.304620000 |
| H | –2.651814000 | –0.781544000 | –0.304620000 |
| H | –3.118062000 | –3.807418000 | –3.302290000 |
| H | 0.619944000 | –1.771735000 | –2.891120000 |
| H | 1.224396000 | 1.422755000 | –2.891120000 |
| H | 2.002744000 | –1.905766000 | –0.304620000 |
| H | 4.856352000 | –0.796612000 | –3.302290000 |

B3LYP-D3(BJ)

| | | | |
|---|---|---|---|
| C | –3.431720000 | –1.981304000 | 2.697076000 |
| C | –3.655269000 | –1.062617000 | 1.667202000 |
| C | –2.544716000 | –0.456070000 | 1.060946000 |
| C | –1.228329000 | –0.764859000 | 1.426698000 |
| C | –1.054859000 | –1.678449000 | 2.479281000 |
| C | –2.133047000 | –2.283677000 | 3.132245000 |
| C | –0.048223000 | 1.446194000 | 1.426698000 |
| C | 0.877390000 | 2.431824000 | 1.060946000 |
| C | 0.907381000 | 3.696864000 | 1.667202000 |
| C | 0.000000000 | 3.962609000 | 2.697076000 |
| C | –0.911199000 | 2.989111000 | 3.132245000 |
| C | –0.926150000 | 1.752759000 | 2.479281000 |
| C | 3.044245000 | –0.705434000 | 3.132245000 |
| C | 1.981008000 | –0.074310000 | 2.479281000 |
| C | 1.276552000 | –0.681335000 | 1.426698000 |
| C | 1.667327000 | –1.975754000 | 1.060946000 |
| C | 2.747888000 | –2.634247000 | 1.667202000 |
| C | 3.431720000 | –1.981304000 | 2.697076000 |
| C | 0.000000000 | 0.000000000 | 0.830798000 |
| C | 3.431720000 | 1.981304000 | –2.697076000 |
| C | 2.133047000 | 2.283677000 | –3.132245000 |
| C | 1.054859000 | 1.678449000 | –2.479281000 |
| C | 1.228329000 | 0.764859000 | –1.426698000 |
| C | 2.544716000 | 0.456070000 | –1.060946000 |
| C | 3.655269000 | 1.062617000 | –1.667202000 |
| C | –1.276552000 | 0.681335000 | –1.426698000 |
| C | –1.667327000 | 1.975754000 | –1.060946000 |
| C | –2.747888000 | 2.634247000 | –1.667202000 |
| C | –3.431720000 | 1.981304000 | –2.697076000 |
| C | –3.044245000 | 0.705434000 | –3.132245000 |
| C | –1.981008000 | 0.074310000 | –2.479281000 |
| C | 0.911199000 | –2.989111000 | –3.132245000 |
| C | 0.926150000 | –1.752759000 | –2.479281000 |
| C | 0.048223000 | –1.446194000 | –1.426698000 |
| C | –0.877390000 | –2.431824000 | –1.060946000 |
| C | –0.907381000 | –3.696864000 | –1.667202000 |
| C | 0.000000000 | –3.962609000 | –2.697076000 |
| C | 0.000000000 | 0.000000000 | –0.830798000 |
| C | –5.066400000 | –0.639149000 | 1.227183000 |
| C | –1.924826000 | –3.227427000 | 4.327199000 |
| C | 1.979681000 | 4.707206000 | 1.227183000 |
| C | –1.832621000 | 3.280661000 | 4.327199000 |
| C | 3.757446000 | –0.053235000 | 4.327199000 |
| C | 3.086719000 | –4.068057000 | 1.227183000 |
| C | 1.924826000 | 3.227427000 | –4.327199000 |
| C | 5.066400000 | 0.639149000 | –1.227183000 |
| C | –3.086719000 | 4.068057000 | –1.227183000 |
| C | –3.757446000 | 0.053235000 | –4.327199000 |
| C | 1.832621000 | –3.280661000 | –4.327199000 |
| C | –1.979681000 | –4.707206000 | –1.227183000 |
| C | –6.162976000 | –1.489779000 | 1.885289000 |
| C | –5.205455000 | –0.767882000 | –0.302204000 |
| C | –5.278026000 | 0.835485000 | 1.629182000 |
| C | –0.437121000 | –3.425034000 | 4.659205000 |
| C | –2.538300000 | –4.608258000 | 4.017538000 |
| C | –2.620268000 | –2.620205000 | 5.564489000 |
| C | 1.791301000 | 6.082183000 | 1.885289000 |
| C | 1.937723000 | 4.891997000 | –0.302204000 |
| C | 3.362565000 | 4.153162000 | 1.629182000 |
| C | –2.747606000 | 2.091075000 | 4.659205000 |
| C | –2.721719000 | 4.502361000 | 4.017538000 |
| C | –0.959030000 | 3.579321000 | 5.564489000 |
| C | 3.184727000 | 1.333959000 | 4.659205000 |
| C | 5.260018000 | 0.105897000 | 4.017538000 |
| C | 3.579298000 | –0.959116000 | 5.564489000 |
| C | 4.371674000 | –4.592404000 | 1.885289000 |
| C | 3.267732000 | –4.124115000 | –0.302204000 |
| C | 1.915461000 | –4.988647000 | 1.629182000 |
| C | 2.620268000 | 2.620205000 | –5.564489000 |
| C | 2.538300000 | 4.608258000 | –4.017538000 |
| C | 0.437121000 | 3.425034000 | –4.659205000 |
| C | 5.205455000 | 0.767882000 | 0.302204000 |
| C | 6.162976000 | 1.489779000 | –1.885289000 |
| C | 5.278026000 | –0.835485000 | –1.629182000 |
| C | –3.267732000 | 4.124115000 | 0.302204000 |
| C | –4.371674000 | 4.592404000 | –1.885289000 |
| C | –1.915461000 | 4.988647000 | –1.629182000 |
| C | –3.579298000 | 0.959116000 | –5.564489000 |
| C | –5.260018000 | –0.105897000 | –4.017538000 |
| C | –3.184727000 | –1.333959000 | –4.659205000 |
| C | 0.959030000 | –3.579321000 | –5.564489000 |
| C | 2.721719000 | –4.502361000 | –4.017538000 |
| C | 2.747606000 | –2.091075000 | –4.659205000 |
| C | –1.937723000 | –4.891997000 | 0.302204000 |
| C | –1.791301000 | –6.082183000 | –1.885289000 |
| C | –3.362565000 | –4.153162000 | –1.629182000 |
| H | –6.169111000 | –1.378658000 | 2.980605000 |
| H | –6.042429000 | –2.558605000 | 1.648393000 |
| H | –7.150649000 | –1.172897000 | 1.515703000 |
| H | –4.452482000 | –0.175374000 | –0.838706000 |
| H | –6.198996000 | –0.417038000 | –0.625639000 |
| H | –5.097621000 | –1.817004000 | –0.618214000 |
| H | –5.218095000 | 0.955833000 | 2.722395000 |
| H | –6.268180000 | 1.190404000 | 1.298210000 |

| | | | |
|---|---|---|---|
| H | –4.514383000 | 1.482955000 | 1.175085000 |
| H | –0.336132000 | –4.114899000 | 5.511542000 |
| H | 0.051996000 | –2.477886000 | 4.934736000 |
| H | 0.115211000 | –3.854389000 | 3.810672000 |
| H | –2.438419000 | –5.276245000 | 4.888874000 |
| H | –2.027888000 | –5.079477000 | 3.164109000 |
| H | –3.608421000 | –4.534030000 | 3.771396000 |
| H | –2.472374000 | –3.265784000 | 6.445974000 |
| H | –3.703689000 | –2.506007000 | 5.408040000 |
| H | –2.207007000 | –1.624672000 | 5.793052000 |
| H | 1.890603000 | 6.031936000 | 2.980605000 |
| H | 0.805398000 | 6.512199000 | 1.648393000 |
| H | 2.559566000 | 6.779092000 | 1.515703000 |
| H | 2.074363000 | 3.943650000 | –0.838706000 |
| H | 2.738333000 | 5.577007000 | –0.625639000 |
| H | 0.975238000 | 5.323171000 | –0.618214000 |
| H | 3.436823000 | 4.041086000 | 2.722395000 |
| H | 4.165010000 | 4.833201000 | 1.298210000 |
| H | 3.541468000 | 3.168093000 | 1.175085000 |
| H | –3.395541000 | 2.348548000 | 5.511542000 |
| H | –2.171910000 | 1.193914000 | 4.934736000 |
| H | –3.395604000 | 1.827419000 | 3.810672000 |
| H | –3.350153000 | 4.749855000 | 4.888874000 |
| H | –3.385012000 | 4.295942000 | 3.164109000 |
| H | –2.122374000 | 5.391999000 | 3.771396000 |
| H | –1.592065000 | 3.774031000 | 6.445974000 |
| H | –0.318421000 | 4.460492000 | 5.408040000 |
| H | –0.303503000 | 2.723660000 | 5.793052000 |
| H | 3.731673000 | 1.766351000 | 5.511542000 |
| H | 2.119915000 | 1.283973000 | 4.934736000 |
| H | 3.280394000 | 2.026970000 | 3.810672000 |
| H | 5.788572000 | 0.526390000 | 4.888874000 |
| H | 5.412900000 | 0.783536000 | 3.164109000 |
| H | 5.730795000 | –0.857969000 | 3.771396000 |
| H | 4.064439000 | –0.508247000 | 6.445974000 |
| H | 4.022110000 | –1.954485000 | 5.408040000 |
| H | 2.510510000 | –1.098988000 | 5.793052000 |
| H | 4.278508000 | –4.653278000 | 2.980605000 |
| H | 5.237031000 | –3.953594000 | 1.648393000 |
| H | 4.591083000 | –5.606195000 | 1.515703000 |
| H | 2.378120000 | –3.768276000 | –0.838706000 |
| H | 3.460663000 | –5.159970000 | –0.625639000 |
| H | 4.122382000 | –3.506167000 | –0.618214000 |
| H | 1.781272000 | –4.996919000 | 2.722395000 |
| H | 2.103170000 | –6.023605000 | 1.298210000 |
| H | 0.972915000 | –4.651048000 | 1.175085000 |
| H | 3.703689000 | 2.506007000 | –5.408040000 |
| H | 2.472374000 | 3.265784000 | –6.445974000 |
| H | 2.207007000 | 1.624672000 | –5.793052000 |
| H | 3.608421000 | 4.534030000 | –3.771396000 |
| H | 2.027888000 | 5.079477000 | –3.164109000 |
| H | 2.438419000 | 5.276245000 | –4.888874000 |
| H | –0.115211000 | 3.854389000 | –3.810672000 |
| H | –0.051996000 | 2.477886000 | –4.934736000 |
| H | 0.336132000 | 4.114899000 | –5.511542000 |
| H | 6.198996000 | 0.417038000 | 0.625639000 |
| H | 4.452482000 | 0.175374000 | 0.838706000 |
| H | 5.097621000 | 1.817004000 | 0.618214000 |
| H | 7.150649000 | 1.172897000 | –1.515703000 |
| H | 6.042429000 | 2.558605000 | –1.648393000 |
| H | 6.169111000 | 1.378658000 | –2.980605000 |
| H | 6.268180000 | –1.190404000 | –1.298210000 |
| H | 5.218095000 | –0.955833000 | –2.722395000 |
| H | 4.514383000 | –1.482955000 | –1.175085000 |
| H | –3.460663000 | 5.159970000 | 0.625639000 |
| H | –2.378120000 | 3.768276000 | 0.838706000 |
| H | –4.122382000 | 3.506167000 | 0.618214000 |
| H | –4.591083000 | 5.606195000 | –1.515703000 |
| H | –5.237031000 | 3.953594000 | –1.648393000 |
| H | –4.278508000 | 4.653278000 | –2.980605000 |
| H | –2.103170000 | 6.023605000 | –1.298210000 |
| H | –1.781272000 | 4.996919000 | –2.722395000 |
| H | –0.972915000 | 4.651048000 | –1.175085000 |
| H | –4.022110000 | 1.954485000 | –5.408040000 |
| H | –4.064439000 | 0.508247000 | –6.445974000 |
| H | –2.510510000 | 1.098988000 | –5.793052000 |
| H | –5.730795000 | 0.857969000 | –3.771396000 |
| H | –5.412900000 | –0.783536000 | –3.164109000 |
| H | –5.788572000 | –0.526390000 | –4.888874000 |
| H | –3.280394000 | –2.026970000 | –3.810672000 |
| H | –2.119915000 | –1.283973000 | –4.934736000 |
| H | –3.731673000 | –1.766351000 | –5.511542000 |
| H | 0.318421000 | –4.460492000 | –5.408040000 |
| H | 1.592065000 | –3.774031000 | –6.445974000 |
| H | 0.303503000 | –2.723660000 | –5.793052000 |
| H | 2.122374000 | –5.391999000 | –3.771396000 |
| H | 3.385012000 | –4.295942000 | –3.164109000 |
| H | 3.350153000 | –4.749855000 | –4.888874000 |
| H | 3.395604000 | –1.827419000 | –3.810672000 |
| H | 2.171910000 | –1.193914000 | –4.934736000 |
| H | 3.395541000 | –2.348548000 | –5.511542000 |
| H | –2.738333000 | –5.577007000 | 0.625639000 |
| H | –2.074363000 | –3.943650000 | 0.838706000 |
| H | –0.975238000 | –5.323171000 | 0.618214000 |
| H | –2.559566000 | –6.779092000 | –1.515703000 |
| H | –0.805398000 | –6.512199000 | –1.648393000 |
| H | –1.890603000 | –6.031936000 | –2.980605000 |
| H | –4.165010000 | –4.833201000 | –1.298210000 |
| H | –3.436823000 | –4.041086000 | –2.722395000 |
| H | –3.541468000 | –3.168093000 | –1.175085000 |
| H | –4.278889000 | –2.453561000 | 3.190599000 |
| H | –2.721075000 | 0.293996000 | 0.301476000 |
| H | –0.052859000 | –1.906398000 | 2.815913000 |
| H | 1.615146000 | 2.209522000 | 0.301476000 |
| H | 0.014598000 | 4.932407000 | 3.190599000 |
| H | –1.624560000 | 0.998976000 | 2.815913000 |
| H | 1.677418000 | 0.907422000 | 2.815913000 |
| H | 1.105929000 | –2.503518000 | 0.301476000 |
| H | 4.264291000 | –2.478846000 | 3.190599000 |
| H | 4.278889000 | 2.453561000 | –3.190599000 |
| H | 0.052859000 | 1.906398000 | –2.815913000 |
| H | 2.721075000 | –0.293996000 | –0.301476000 |
| H | –1.105929000 | 2.503518000 | –0.301476000 |
| H | –4.264291000 | 2.478846000 | –3.190599000 |
| H | –1.677418000 | –0.907422000 | –2.815913000 |
| H | 1.624560000 | –0.998976000 | –2.815913000 |
| H | –1.615146000 | –2.209522000 | –0.301476000 |
| H | –0.014598000 | –4.932407000 | –3.190599000 |

M06-2X

| | | | |
|---|---|---|---|
| C | 1.522580000 | –3.663230000 | 2.698180000 |
| C | 0.567670000 | –3.757410000 | 1.684590000 |
| C | 0.112340000 | –2.576150000 | 1.082920000 |
| C | 0.604320000 | –1.313650000 | 1.433370000 |
| C | 1.542700000 | –1.268040000 | 2.477340000 |
| C | 1.999740000 | –2.418510000 | 3.126800000 |
| C | –1.439814000 | 0.133469000 | 1.433370000 |
| C | –2.287181000 | 1.190786000 | 1.082920000 |
| C | –3.537848000 | 1.387088000 | 1.684590000 |
| C | –3.933740000 | 0.513022000 | 2.698180000 |
| C | –3.094361000 | –0.522571000 | 3.126800000 |
| C | –1.869505000 | –0.701997000 | 2.477340000 |
| C | 1.094621000 | 2.941081000 | 3.126800000 |
| C | 0.326805000 | 1.970037000 | 2.477340000 |
| C | 0.835494000 | 1.180181000 | 1.433370000 |
| C | 2.174841000 | 1.385364000 | 1.082920000 |
| C | 2.970178000 | 2.370322000 | 1.684590000 |
| C | 2.411160000 | 3.150208000 | 2.698180000 |
| C | 0.000000000 | 0.000000000 | 0.832810000 |
| C | –1.522580000 | 3.663230000 | –2.698180000 |
| C | –1.999740000 | 2.418510000 | –3.126800000 |
| C | –1.542700000 | 1.268040000 | –2.477340000 |
| C | –0.604320000 | 1.313650000 | –1.433370000 |
| C | –0.112340000 | 2.576150000 | –1.082920000 |
| C | –0.567670000 | 3.757410000 | –1.684590000 |
| C | –0.835494000 | –1.180181000 | –1.433370000 |
| C | –2.174841000 | –1.385364000 | –1.082920000 |
| C | –2.970178000 | –2.370322000 | –1.684590000 |
| C | –2.411160000 | –3.150208000 | –2.698180000 |
| C | –1.094621000 | –2.941081000 | –3.126800000 |
| C | –0.326805000 | –1.970037000 | –2.477340000 |
| C | 3.094361000 | 0.522571000 | –3.126800000 |
| C | 1.869505000 | 0.701997000 | –2.477340000 |
| C | 1.439814000 | –0.133469000 | –1.433370000 |
| C | 2.287181000 | –1.190786000 | –1.082920000 |
| C | 3.537848000 | –1.387088000 | –1.684590000 |
| C | 3.933740000 | –0.513022000 | –2.698180000 |
| C | 0.000000000 | 0.000000000 | –0.832810000 |
| C | –0.064290000 | –5.089930000 | 1.264800000 |
| C | 2.987290000 | –2.345200000 | 4.297520000 |
| C | –4.375864000 | 2.600642000 | 1.264800000 |
| C | –3.524648000 | –1.414469000 | 4.297520000 |
| C | 0.537358000 | 3.759669000 | 4.297520000 |
| C | 4.440154000 | 2.489288000 | 1.264800000 |
| C | –2.987290000 | 2.345200000 | –4.297520000 |
| C | 0.064290000 | 5.089930000 | –1.264800000 |
| C | –4.440154000 | –2.489288000 | –1.264800000 |
| C | –0.537358000 | –3.759669000 | –4.297520000 |
| C | 3.524648000 | 1.414469000 | –4.297520000 |
| C | 4.375864000 | –2.600642000 | –1.264800000 |
| C | 0.637110000 | –6.290550000 | 1.905020000 |
| C | 0.000000000 | –5.248730000 | –0.260990000 |
| C | –1.536210000 | –5.086170000 | 1.707860000 |
| C | 3.305200000 | –0.899990000 | 4.692290000 |
| C | 4.300090000 | –3.041940000 | 3.906130000 |

| | | | |
|---|---|---|---|
| C | 2.379250000 | –3.053910000 | 5.519170000 |
| C | –5.766331000 | 2.593522000 | 1.905020000 |
| C | –4.545534000 | 2.624365000 | –0.260990000 |
| C | –3.636647000 | 3.873482000 | 1.707860000 |
| C | –2.432014000 | –2.412392000 | 4.692290000 |
| C | –4.784442000 | –2.203017000 | 3.906130000 |
| C | –3.834389000 | –0.533536000 | 5.519170000 |
| C | –0.873186000 | 3.312382000 | 4.692290000 |
| C | 0.484352000 | 5.244957000 | 3.906130000 |
| C | 1.455139000 | 3.587446000 | 5.519170000 |
| C | 5.129221000 | 3.697028000 | 1.905020000 |
| C | 4.545534000 | 2.624365000 | –0.260990000 |
| C | 5.172857000 | 1.212688000 | 1.707860000 |
| C | –2.379250000 | 3.053910000 | –5.519170000 |
| C | –4.300090000 | 3.041940000 | –3.906130000 |
| C | –3.305200000 | 0.899990000 | –4.692290000 |
| C | 0.000000000 | 5.248730000 | 0.260990000 |
| C | –0.637110000 | 6.290550000 | –1.905020000 |
| C | 1.536210000 | 5.086170000 | –1.707860000 |
| C | –4.545534000 | –2.624365000 | 0.260990000 |
| C | –5.129221000 | –3.697028000 | –1.905020000 |
| C | –5.172857000 | –1.212688000 | –1.707860000 |
| C | –1.455139000 | –3.587446000 | –5.519170000 |
| C | –0.484352000 | –5.244957000 | –3.906130000 |
| C | 0.873186000 | –3.312382000 | –4.692290000 |
| C | 3.834389000 | 0.533536000 | –5.519170000 |
| C | 4.784442000 | 2.203017000 | –3.906130000 |
| C | 2.432014000 | 2.412392000 | –4.692290000 |
| C | 4.545534000 | –2.624365000 | 0.260990000 |
| C | 5.766331000 | –2.593522000 | –1.905020000 |
| C | 3.636647000 | –3.873482000 | –1.707860000 |
| H | 0.553940000 | –6.274330000 | 3.001840000 |
| H | 1.704580000 | –6.321830000 | 1.638130000 |
| H | 0.170670000 | –7.221350000 | 1.549290000 |
| H | –0.495810000 | –4.418210000 | –0.786460000 |
| H | –0.497660000 | –6.183670000 | –0.564480000 |
| H | 1.046390000 | –5.295770000 | –0.600980000 |
| H | –1.610650000 | –5.041630000 | 2.805320000 |
| H | –2.043560000 | –6.002120000 | 1.365120000 |
| H | –2.069530000 | –4.217640000 | 1.291360000 |
| H | 4.006000000 | –0.895630000 | 5.540490000 |
| H | 2.397960000 | –0.355500000 | 4.996630000 |
| H | 3.773000000 | –0.348430000 | 3.863560000 |
| H | 4.996780000 | –3.044560000 | 4.759380000 |
| H | 4.782800000 | –2.516410000 | 3.068290000 |
| H | 4.127890000 | –4.085500000 | 3.602080000 |
| H | 3.070600000 | –2.993380000 | 6.374050000 |
| H | 2.184280000 | –4.117330000 | 5.318340000 |
| H | 1.428350000 | –2.581110000 | 5.808750000 |
| H | –5.710699000 | 2.657439000 | 3.001840000 |
| H | –6.327155000 | 1.684705000 | 1.638130000 |
| H | –6.339208000 | 3.462870000 | 1.549290000 |
| H | –3.578377000 | 2.638489000 | –0.786460000 |
| H | –5.106385000 | 3.522821000 | –0.564480000 |
| H | –5.109466000 | 1.741685000 | –0.600980000 |
| H | –3.560855000 | 3.915679000 | 2.805320000 |
| H | –4.176208000 | 4.770835000 | 1.365120000 |
| H | –2.617818000 | 3.901086000 | 1.291360000 |
| H | –2.778638000 | –3.021483000 | 5.540490000 |
| H | –1.506852000 | –1.898944000 | 4.996630000 |
| H | –2.188249000 | –3.093299000 | 3.863560000 |
| H | –5.135056000 | –2.805058000 | 4.759380000 |
| H | –4.570675000 | –2.883821000 | 3.068290000 |
| H | –5.602092000 | –1.532108000 | 3.602080000 |
| H | –4.127643000 | –1.162528000 | 6.374050000 |
| H | –4.657852000 | 0.167023000 | 5.318340000 |
| H | –2.949482000 | 0.053568000 | 5.808750000 |
| H | –1.227362000 | 3.917113000 | 5.540490000 |
| H | –0.891108000 | 2.254444000 | 4.996630000 |
| H | –1.584751000 | 3.441729000 | 3.863560000 |
| H | 0.138276000 | 5.849618000 | 4.759380000 |
| H | –0.212125000 | 5.400231000 | 3.068290000 |
| H | 1.474202000 | 5.617608000 | 3.602080000 |
| H | 1.057043000 | 4.155908000 | 6.374050000 |
| H | 2.473572000 | 3.950307000 | 5.318340000 |
| H | 1.521132000 | 2.527542000 | 5.808750000 |
| H | 5.156759000 | 3.616891000 | 3.001840000 |
| H | 4.622575000 | 4.637125000 | 1.638130000 |
| H | 6.168538000 | 3.758480000 | 1.549290000 |
| H | 4.074187000 | 1.779721000 | –0.786460000 |
| H | 5.604045000 | 2.660849000 | –0.564480000 |
| H | 4.063076000 | 3.554085000 | –0.600980000 |
| H | 5.171505000 | 1.125951000 | 2.805320000 |
| H | 6.219768000 | 1.231285000 | 1.365120000 |
| H | 4.687348000 | 0.316554000 | 1.291360000 |
| H | –2.184280000 | 4.117330000 | –5.318340000 |
| H | –3.070600000 | 2.993380000 | –6.374050000 |
| H | –1.428350000 | 2.581110000 | –5.808750000 |
| H | –4.127890000 | 4.085500000 | –3.602080000 |
| H | –4.782800000 | 2.516410000 | –3.068290000 |
| H | –4.996780000 | 3.044560000 | –4.759380000 |
| H | –3.773000000 | 0.348430000 | –3.863560000 |
| H | –2.397960000 | 0.355500000 | –4.996630000 |
| H | –4.006000000 | 0.895630000 | –5.540490000 |
| H | 0.497660000 | 6.183670000 | 0.564480000 |
| H | 0.495810000 | 4.418210000 | 0.786460000 |
| H | –1.046390000 | 5.295770000 | 0.600980000 |
| H | –0.170670000 | 7.221350000 | –1.549290000 |
| H | –1.704580000 | 6.321830000 | –1.638130000 |
| H | –0.553940000 | 6.274330000 | –3.001840000 |
| H | 2.043560000 | 6.002120000 | –1.365120000 |
| H | 1.610650000 | 5.041630000 | –2.805320000 |
| H | 2.069530000 | 4.217640000 | –1.291360000 |
| H | –5.604045000 | –2.660849000 | 0.564480000 |
| H | –4.074187000 | –1.779721000 | 0.786460000 |
| H | –4.063076000 | –3.554085000 | 0.600980000 |
| H | –6.168538000 | –3.758480000 | –1.549290000 |
| H | –4.622575000 | –4.637125000 | –1.638130000 |
| H | –5.156759000 | –3.616891000 | –3.001840000 |
| H | –6.219768000 | –1.231285000 | –1.365120000 |
| H | –5.171505000 | –1.125951000 | –2.805320000 |
| H | –4.687348000 | –0.316554000 | –1.291360000 |
| H | –2.473572000 | –3.950307000 | –5.318340000 |
| H | –1.057043000 | –4.155908000 | –6.374050000 |
| H | –1.521132000 | –2.527542000 | –5.808750000 |
| H | –1.474202000 | –5.617608000 | –3.602080000 |
| H | 0.212125000 | –5.400231000 | –3.068290000 |
| H | –0.138276000 | –5.849618000 | –4.759380000 |
| H | 1.584751000 | –3.441729000 | –3.863560000 |
| H | 0.891108000 | –2.254444000 | –4.996630000 |
| H | 1.227362000 | –3.917113000 | –5.540490000 |
| H | 4.657852000 | –0.167023000 | –5.318340000 |
| H | 4.127643000 | 1.162528000 | –6.374050000 |
| H | 2.949482000 | –0.053568000 | –5.808750000 |
| H | 5.602092000 | 1.532108000 | –3.602080000 |
| H | 4.570675000 | 2.883821000 | –3.068290000 |
| H | 5.135056000 | 2.805058000 | –4.759380000 |
| H | 2.188249000 | 3.093299000 | –3.863560000 |
| H | 1.506852000 | 1.898944000 | –4.996630000 |
| H | 2.778638000 | 3.021483000 | –5.540490000 |
| H | 5.106385000 | –3.522821000 | 0.564480000 |
| H | 3.578377000 | –2.638489000 | 0.786460000 |
| H | 5.109466000 | –1.741685000 | 0.600980000 |
| H | 6.339208000 | –3.462870000 | –1.549290000 |
| H | 6.327155000 | –1.684705000 | –1.638130000 |
| H | 5.710699000 | –2.657439000 | –3.001840000 |
| H | 4.176208000 | –4.770835000 | –1.365120000 |
| H | 3.560855000 | –3.915679000 | –2.805320000 |
| H | 2.617818000 | –3.901086000 | –1.291360000 |
| H | 1.887550000 | –4.567810000 | 3.184880000 |
| H | –0.680110000 | –2.657400000 | 0.346210000 |
| H | 1.910830000 | –0.308100000 | 2.820440000 |
| H | –1.961321000 | 1.917693000 | 0.346210000 |
| H | –4.899614000 | 0.649239000 | 3.184880000 |
| H | –1.222237000 | –1.500777000 | 2.820440000 |
| H | –0.688593000 | 1.808877000 | 2.820440000 |
| H | 2.641431000 | 0.739707000 | 0.346210000 |
| H | 3.012064000 | 3.918571000 | 3.184880000 |
| H | –1.887550000 | 4.567810000 | –3.184880000 |
| H | –1.910830000 | 0.308100000 | –2.820440000 |
| H | 0.680110000 | 2.657400000 | –0.346210000 |
| H | –2.641431000 | –0.739707000 | –0.346210000 |
| H | –3.012064000 | –3.918571000 | –3.184880000 |
| H | 0.688593000 | –1.808877000 | –2.820440000 |
| H | 1.222237000 | 1.500777000 | –2.820440000 |
| H | 1.961321000 | –1.917693000 | –0.346210000 |
| H | 4.899614000 | –0.649239000 | –3.184880000 |

Cy-**1**$_2$

B3LYP

| | | | |
|---|---|---|---|
| C | 0.000000000 | 0.000000000 | 0.858503000 |
| C | –1.630958000 | –1.256154000 | 2.445661000 |
| C | –2.881949000 | –1.486548000 | 3.042706000 |
| C | –1.431499000 | –0.290552000 | 1.444848000 |
| C | –3.975753000 | –0.718999000 | 2.630615000 |
| C | –2.557026000 | 0.480514000 | 1.089741000 |
| C | –3.822284000 | 0.278317000 | 1.655899000 |
| C | –4.992060000 | 1.153244000 | 1.229519000 |
| C | –6.159302000 | 0.342244000 | 0.624044000 |
| C | –5.505079000 | 2.048145000 | 2.381001000 |
| C | –7.314271000 | 1.249466000 | 0.176708000 |
| C | –6.660585000 | 2.953641000 | 1.932741000 |
| C | –7.810479000 | 2.140284000 | 1.323700000 |
| C | 5.888221000 | 4.291416000 | 1.932741000 |

| | | | |
|---|---|---|---|
| C | 4.526285000 | 3.743466000 | 2.381001000 |
| C | 5.758780000 | 5.693931000 | 1.323700000 |
| C | 1.365205000 | 3.802603000 | 2.630615000 |
| C | 3.494768000 | 3.746629000 | 1.229519000 |
| C | 0.153586000 | 3.239115000 | 3.042706000 |
| C | 2.152171000 | 3.171037000 | 1.655899000 |
| C | –0.272382000 | 2.040529000 | 2.445661000 |
| C | 1.694650000 | 1.974192000 | 1.089741000 |
| C | 0.464124000 | 1.384991000 | 1.444848000 |
| C | 4.739204000 | 5.709611000 | 0.176708000 |
| C | 3.376043000 | 5.162990000 | 0.624044000 |
| C | 2.575066000 | –6.959077000 | 0.176708000 |
| C | 2.783259000 | –5.505234000 | 0.624044000 |
| C | 2.051699000 | –7.834215000 | 1.323700000 |
| C | 2.610548000 | –3.083604000 | 2.630615000 |
| C | 1.497292000 | –4.899873000 | 1.229519000 |
| C | 2.728363000 | –1.752567000 | 3.042706000 |
| C | 0.772365000 | –7.245057000 | 1.932741000 |
| C | 1.670113000 | –3.449353000 | 1.655899000 |
| C | 0.978794000 | –5.791611000 | 2.381001000 |
| C | 1.903341000 | –0.784374000 | 2.445661000 |
| C | 0.862375000 | –2.454706000 | 1.089741000 |
| C | 0.967375000 | –1.094439000 | 1.444848000 |
| C | 4.277881000 | –1.454763000 | 6.619368000 |
| C | 3.314581000 | –1.913674000 | 5.515805000 |
| C | 3.704959000 | –1.339956000 | 4.134585000 |
| C | 5.733582000 | –1.800400000 | 6.278316000 |
| C | 6.133298000 | –1.235419000 | 4.908693000 |
| C | 5.171094000 | –1.695656000 | 3.804215000 |
| C | –4.054028000 | –3.630470000 | 3.804215000 |
| C | –3.012916000 | –2.538610000 | 4.134585000 |
| C | –3.314581000 | –1.913674000 | 5.515805000 |
| C | –4.136553000 | –4.693882000 | 4.908693000 |
| C | –4.425983000 | –4.065228000 | 6.278316000 |
| C | –3.398802000 | –2.977372000 | 6.619368000 |
| C | –0.692043000 | 3.878566000 | 4.134585000 |
| C | –1.117065000 | 5.326127000 | 3.804215000 |
| C | 0.000000000 | 3.827349000 | 5.515805000 |
| C | –1.996745000 | 5.929301000 | 4.908693000 |
| C | –0.879078000 | 4.432135000 | 6.619368000 |
| C | –1.307599000 | 5.865628000 | 6.278316000 |
| C | –0.464124000 | –1.384991000 | –1.444848000 |
| C | 0.000000000 | 0.000000000 | –0.858503000 |
| C | –0.967375000 | 1.094439000 | –1.444848000 |
| C | –0.862375000 | 2.454706000 | –1.089741000 |
| C | –1.670113000 | 3.449353000 | –1.655899000 |
| C | –1.497292000 | 4.899873000 | –1.229519000 |
| C | –0.772365000 | 7.245057000 | –1.932741000 |
| C | –2.051699000 | 7.834215000 | –1.323700000 |
| C | –2.575066000 | 6.959077000 | –0.176708000 |
| C | –0.978794000 | 5.791611000 | –2.381001000 |
| C | –2.783259000 | 5.505234000 | –0.624044000 |
| C | –2.610548000 | 3.083604000 | –2.630615000 |
| C | –2.728363000 | 1.752567000 | –3.042706000 |
| C | –3.704959000 | 1.339956000 | –4.134585000 |
| C | –3.314581000 | 1.913674000 | –5.515805000 |
| C | –4.277881000 | 1.454763000 | –6.619368000 |
| C | –5.733582000 | 1.800400000 | –6.278316000 |
| C | –6.133298000 | 1.235419000 | –4.908693000 |
| C | –5.171094000 | 1.695656000 | –3.804215000 |
| C | –1.903341000 | 0.784374000 | –2.445661000 |
| C | 1.431499000 | 0.290552000 | –1.444848000 |
| C | 1.630958000 | 1.256154000 | –2.445661000 |
| C | 2.881949000 | 1.486548000 | –3.042706000 |
| C | 3.012916000 | 2.538610000 | –4.134585000 |
| C | 4.136553000 | 4.693882000 | –4.908693000 |
| C | 4.425983000 | 4.065228000 | –6.278316000 |
| C | 3.398802000 | 2.977372000 | –6.619368000 |
| C | 4.054028000 | 3.630470000 | –3.804215000 |
| C | 3.314581000 | 1.913674000 | –5.515805000 |
| C | 3.975753000 | 0.718999000 | –2.630615000 |
| C | 3.822284000 | –0.278317000 | –1.655899000 |
| C | 4.992060000 | –1.153244000 | –1.229519000 |
| C | 6.159302000 | –0.342244000 | –0.624044000 |
| C | 7.314271000 | –1.249466000 | –0.176708000 |
| C | 6.660585000 | –2.953641000 | –1.932741000 |
| C | 7.810479000 | –2.140284000 | –1.323700000 |
| C | 5.505079000 | –2.048145000 | –2.381001000 |
| C | 2.557026000 | –0.480514000 | –1.089741000 |
| C | 0.272382000 | –2.040529000 | –2.445661000 |
| C | –0.153586000 | –3.239115000 | –3.042706000 |
| C | 0.692043000 | –3.878566000 | –4.134585000 |
| C | 1.996745000 | –5.929301000 | –4.908693000 |
| C | 1.307599000 | –5.865628000 | –6.278316000 |
| C | 0.879078000 | –4.432135000 | –6.619368000 |
| C | 1.117065000 | –5.326127000 | –3.804215000 |
| C | 0.000000000 | –3.827349000 | –5.515805000 |
| C | –1.365205000 | –3.802603000 | –2.630615000 |
| C | –2.152171000 | –3.171037000 | –1.655899000 |
| C | –3.494768000 | –3.746629000 | –1.229519000 |
| C | –5.888221000 | –4.291416000 | –1.932741000 |
| C | –5.758780000 | –5.693931000 | –1.323700000 |
| C | –4.739204000 | –5.709611000 | –0.176708000 |
| C | –4.526285000 | –3.743466000 | –2.381001000 |
| C | –3.376043000 | –5.162990000 | –0.624044000 |
| C | –1.694650000 | –1.974192000 | –1.089741000 |
| H | –0.795499000 | –1.856219000 | 2.796670000 |
| H | –4.955153000 | –0.885000000 | 3.085628000 |
| H | –2.458135000 | 1.278960000 | 0.361897000 |
| H | –4.619550000 | 1.826067000 | 0.436049000 |
| H | –6.533682000 | –0.377692000 | 1.375220000 |
| H | –5.790319000 | –0.257421000 | –0.225150000 |
| H | –5.846022000 | 1.407296000 | 3.215333000 |
| H | –4.672397000 | 2.654429000 | 2.775997000 |
| H | –6.970130000 | 1.889469000 | –0.657486000 |
| H | –8.142514000 | 0.638565000 | –0.221075000 |
| H | –6.286833000 | 3.673448000 | 1.180284000 |
| H | –7.023109000 | 3.553966000 | 2.784574000 |
| H | –8.610541000 | 2.812165000 | 0.969163000 |
| H | –8.261460000 | 1.504005000 | 2.109014000 |
| H | 6.324715000 | 3.607833000 | 1.180284000 |
| H | 6.589379000 | 4.305208000 | 2.784574000 |
| H | 4.141765000 | 4.359156000 | 3.215333000 |
| H | 4.635002000 | 2.719200000 | 2.775997000 |
| H | 6.740677000 | 6.050865000 | 0.969163000 |
| H | 5.433236000 | 6.402632000 | 2.109014000 |
| H | 1.711144000 | 4.733788000 | 3.085628000 |
| H | 3.891196000 | 3.087614000 | 0.436049000 |
| H | –1.209784000 | 1.617032000 | 2.796670000 |
| H | 2.336680000 | 1.489327000 | 0.361897000 |
| H | 5.121393000 | 5.091575000 | –0.657486000 |
| H | 4.624270000 | 6.732342000 | –0.221075000 |
| H | 2.939750000 | 5.847181000 | 1.375220000 |
| H | 2.672227000 | 5.143274000 | –0.225150000 |
| H | 1.848737000 | –6.981044000 | –0.657486000 |
| H | 3.518244000 | –7.370907000 | –0.221075000 |
| H | 3.593932000 | –5.469489000 | 1.375220000 |
| H | 3.118093000 | –4.885853000 | –0.225150000 |
| H | 1.869864000 | –8.863030000 | 0.969163000 |
| H | 2.828224000 | –7.906636000 | 2.109014000 |
| H | 3.244009000 | –3.848788000 | 3.085628000 |
| H | 0.728354000 | –4.913681000 | 0.436049000 |
| H | –0.037883000 | –7.281281000 | 1.180284000 |
| H | 0.433730000 | –7.859174000 | 2.784574000 |
| H | 1.704257000 | –5.766451000 | 3.215333000 |
| H | 0.037396000 | –5.373629000 | 2.775997000 |
| H | 2.005282000 | 0.239188000 | 2.796670000 |
| H | 0.121456000 | –2.768288000 | 0.361897000 |
| H | 4.185537000 | –0.359795000 | 6.748893000 |
| H | 3.991177000 | –1.906999000 | 7.584065000 |
| H | 2.280542000 | –1.617245000 | 5.759196000 |
| H | 3.317466000 | –3.018470000 | 5.462849000 |
| H | 3.650102000 | –0.238777000 | 4.215756000 |
| H | 5.852760000 | –2.900677000 | 6.266607000 |
| H | 6.411756000 | –1.421970000 | 7.061975000 |
| H | 7.166125000 | –1.531078000 | 4.657477000 |
| H | 6.128944000 | –0.129936000 | 4.956561000 |
| H | 5.262903000 | –2.790228000 | 3.677417000 |
| H | 5.452242000 | –1.245564000 | 2.837206000 |
| H | –5.047860000 | –3.162693000 | 3.677417000 |
| H | –3.804811000 | –4.098998000 | 2.837206000 |
| H | –2.031837000 | –3.041692000 | 4.215756000 |
| H | –2.540846000 | –1.166385000 | 5.759196000 |
| H | –4.272804000 | –1.363775000 | 5.462849000 |
| H | –4.909015000 | –5.440507000 | 4.657477000 |
| H | –3.177000000 | –5.242853000 | 4.956561000 |
| H | –5.438440000 | –3.618300000 | 6.266607000 |
| H | –4.437340000 | –4.841758000 | 7.061975000 |
| H | –2.404360000 | –3.444884000 | 6.748893000 |
| H | –3.647098000 | –2.502961000 | 7.584065000 |
| H | –1.618264000 | 3.280469000 | 4.215756000 |
| H | –0.215043000 | 5.952922000 | 3.677417000 |
| H | –1.647431000 | 5.344562000 | 2.837206000 |
| H | 0.260304000 | 2.783630000 | 5.759196000 |
| H | 0.955338000 | 4.382244000 | 5.462849000 |
| H | –2.257110000 | 6.971585000 | 4.657477000 |
| H | –2.951944000 | 5.372789000 | 4.956561000 |
| H | –1.781177000 | 3.804679000 | 6.748893000 |
| H | –0.344079000 | 4.409960000 | 7.584065000 |
| H | –0.414320000 | 6.518977000 | 6.266607000 |
| H | –1.974415000 | 6.263728000 | 7.061975000 |
| H | –0.121456000 | 2.768288000 | –0.361897000 |
| H | –0.728354000 | 4.913681000 | –0.436049000 |
| H | 0.037883000 | 7.281281000 | –1.180284000 |
| H | –0.433730000 | 7.859174000 | –2.784574000 |
| H | –1.869864000 | 8.863030000 | –0.969163000 |

| | | | |
|---|---|---|---|
| H | –2.828224000 | 7.906636000 | –2.109014000 |
| H | –1.848737000 | 6.981044000 | 0.657486000 |
| H | –3.518244000 | 7.370907000 | 0.221075000 |
| H | –1.704257000 | 5.766451000 | –3.215333000 |
| H | –0.037396000 | 5.373629000 | –2.775997000 |
| H | –3.593932000 | 5.469489000 | –1.375220000 |
| H | –3.118093000 | 4.885853000 | 0.225150000 |
| H | –3.244009000 | 3.848788000 | –3.085628000 |
| H | –3.650102000 | 0.238777000 | –4.215756000 |
| H | –2.280542000 | 1.617245000 | –5.759196000 |
| H | –3.317466000 | 3.018470000 | –5.462849000 |
| H | –4.185537000 | 0.359795000 | –6.748893000 |
| H | –3.991177000 | 1.906999000 | –7.584065000 |
| H | –5.852760000 | 2.900677000 | –6.266607000 |
| H | –6.411756000 | 1.421970000 | –7.061975000 |
| H | –7.166125000 | 1.531078000 | –4.657477000 |
| H | –6.128944000 | 0.129936000 | –4.956561000 |
| H | –5.262903000 | 2.790228000 | –3.677417000 |
| H | –5.452242000 | 1.245564000 | –2.837206000 |
| H | –2.005282000 | –0.239188000 | –2.796670000 |
| H | 0.795499000 | 1.856219000 | –2.796670000 |
| H | 2.031837000 | 3.041692000 | –4.215756000 |
| H | 4.909015000 | 5.440507000 | –4.657477000 |
| H | 3.177000000 | 5.242853000 | –4.956561000 |
| H | 5.438440000 | 3.618300000 | –6.266607000 |
| H | 4.437340000 | 4.841758000 | –7.061975000 |
| H | 2.404360000 | 3.444884000 | –6.748893000 |
| H | 3.647098000 | 2.502961000 | –7.584065000 |
| H | 5.047860000 | 3.162693000 | –3.677417000 |
| H | 3.804811000 | 4.098998000 | –2.837206000 |
| H | 2.540846000 | 1.166385000 | –5.759196000 |
| H | 4.272804000 | 1.363775000 | –5.462849000 |
| H | 4.955153000 | 0.885000000 | –3.085628000 |
| H | 4.619550000 | –1.826067000 | –0.436049000 |
| H | 6.533682000 | 0.377692000 | –1.375220000 |
| H | 5.790319000 | 0.257421000 | 0.225150000 |
| H | 6.970130000 | –1.889469000 | 0.657486000 |
| H | 8.142514000 | –0.638565000 | 0.221075000 |
| H | 6.286833000 | –3.673448000 | –1.180284000 |
| H | 7.023109000 | –3.553966000 | –2.784574000 |
| H | 8.610541000 | –2.812165000 | –0.969163000 |
| H | 8.261460000 | –1.504005000 | –2.109014000 |
| H | 5.846022000 | –1.407296000 | –3.215333000 |
| H | 4.672397000 | –2.654429000 | –2.775997000 |
| H | 2.458135000 | –1.278960000 | –0.361897000 |
| H | 1.209784000 | –1.617032000 | –2.796670000 |
| H | 1.618264000 | –3.280469000 | –4.215756000 |
| H | 2.257110000 | –6.971585000 | –4.657477000 |
| H | 2.951944000 | –5.372789000 | –4.956561000 |
| H | 0.414320000 | –6.518977000 | –6.266607000 |
| H | 1.974415000 | –6.263728000 | –7.061975000 |
| H | 1.781177000 | –3.804679000 | –6.748893000 |
| H | 0.344079000 | –4.409960000 | –7.584065000 |
| H | 0.215043000 | –5.952922000 | –3.677417000 |
| H | 1.647431000 | –5.344562000 | –2.837206000 |
| H | –0.260304000 | –2.783630000 | –5.759196000 |
| H | –0.955338000 | –4.382244000 | –5.462849000 |
| H | –1.711144000 | –4.733788000 | –3.085628000 |
| H | –3.891196000 | –3.087614000 | –0.436049000 |
| H | –6.324715000 | –3.607833000 | –1.180284000 |
| H | –6.589379000 | –4.305208000 | –2.784574000 |
| H | –6.740677000 | –6.050865000 | –0.969163000 |
| H | –5.433236000 | –6.402632000 | –2.109014000 |
| H | –5.121393000 | –5.091575000 | 0.657486000 |
| H | –4.624270000 | –6.732342000 | 0.221075000 |
| H | –4.141765000 | –4.359156000 | –3.215333000 |
| H | –4.635002000 | –2.719200000 | –2.775997000 |
| H | –2.939750000 | –5.847181000 | –1.375220000 |
| H | –2.672227000 | –5.143274000 | 0.225150000 |
| H | –2.336680000 | –1.489327000 | –0.361897000 |

B3LYP-D3(BJ)

| | | | |
|---|---|---|---|
| C | 0.000000000 | 0.000000000 | 0.854920000 |
| C | –1.810650000 | 1.205810000 | 2.254320000 |
| C | –2.498230000 | 2.347870000 | 2.685930000 |
| C | –0.710260000 | 1.272360000 | 1.389730000 |
| C | –2.045160000 | 3.608250000 | 2.279230000 |
| C | –0.254460000 | 2.557510000 | 1.053960000 |
| C | –0.897610000 | 3.721470000 | 1.483990000 |
| C | –0.257840000 | 5.046010000 | 1.114090000 |
| C | –1.211100000 | 6.247470000 | 1.081510000 |
| C | 0.940990000 | 5.338210000 | 2.042040000 |
| C | –0.479180000 | 7.527210000 | 0.658670000 |
| C | 1.677640000 | 6.613250000 | 1.623280000 |
| C | 0.724190000 | 7.812690000 | 1.565970000 |
| C | 4.888423000 | –4.759504000 | 1.623280000 |
| C | 4.152530000 | –3.484026000 | 2.042040000 |
| C | 6.403893000 | –4.533512000 | 1.565970000 |
| C | 4.147416000 | –0.032964000 | 2.279230000 |
| C | 4.498893000 | –2.299709000 | 1.114090000 |
| C | 3.282430000 | 0.989596000 | 2.685930000 |
| C | 3.671693000 | –1.083382000 | 1.483990000 |
| C | 1.949587000 | 0.965164000 | 2.254320000 |
| C | 2.342099000 | –1.058386000 | 1.053960000 |
| C | 1.457026000 | –0.021077000 | 1.389730000 |
| C | 6.758345000 | –3.348623000 | 0.658670000 |
| C | 6.016018000 | –2.074892000 | 1.081510000 |
| C | –6.279165000 | –4.178587000 | 0.658670000 |
| C | –4.804918000 | –4.172578000 | 1.081510000 |
| C | –7.128083000 | –3.279178000 | 1.565970000 |
| C | –2.102256000 | –3.575286000 | 2.279230000 |
| C | –4.241053000 | –2.746301000 | 1.114090000 |
| C | –0.784200000 | –3.337466000 | 2.685930000 |
| C | –6.566063000 | –1.853746000 | 1.623280000 |
| C | –2.774083000 | –2.638088000 | 1.483990000 |
| C | –5.093520000 | –1.854184000 | 2.042040000 |
| C | –0.138937000 | –2.170974000 | 2.254320000 |
| C | –2.087639000 | –1.499124000 | 1.053960000 |
| C | –0.746766000 | –1.251283000 | 1.389730000 |
| C | 0.075560000 | –5.924890000 | 5.490970000 |
| C | –0.799310000 | –4.970010000 | 4.670110000 |
| C | 0.000000000 | –4.328640000 | 3.524270000 |
| C | 0.702020000 | –7.009500000 | 4.606270000 |
| C | 1.490240000 | –6.393140000 | 3.444520000 |
| C | 0.623000000 | –5.424700000 | 2.635960000 |
| C | –5.009428000 | 2.172816000 | 2.635960000 |
| C | –3.748712000 | 2.164320000 | 3.524270000 |
| C | –3.904500000 | 3.177228000 | 4.670110000 |
| C | –6.281742000 | 1.905984000 | 3.444520000 |
| C | –6.421415000 | 2.896783000 | 4.606270000 |
| C | –5.168885000 | 2.897008000 | 5.490970000 |
| C | 3.748712000 | 2.164320000 | 3.524270000 |
| C | 4.386428000 | 3.251884000 | 2.635960000 |
| C | 4.703810000 | 1.792782000 | 4.670110000 |
| C | 4.791502000 | 4.487156000 | 3.444520000 |
| C | 5.093325000 | 3.027882000 | 5.490970000 |
| C | 5.719395000 | 4.112717000 | 4.606270000 |
| C | –1.457026000 | 0.021077000 | –1.389730000 |
| C | 0.000000000 | 0.000000000 | –0.854920000 |
| C | 0.746766000 | 1.251283000 | –1.389730000 |
| C | 2.087639000 | 1.499124000 | –1.053960000 |
| C | 2.774083000 | 2.638088000 | –1.483990000 |
| C | 4.241053000 | 2.746301000 | –1.114090000 |
| C | 6.566063000 | 1.853746000 | –1.623280000 |
| C | 7.128083000 | 3.279178000 | –1.565970000 |
| C | 6.279165000 | 4.178587000 | –0.658670000 |
| C | 5.093520000 | 1.854184000 | –2.042040000 |
| C | 4.804918000 | 4.172578000 | –1.081510000 |
| C | 2.102256000 | 3.575286000 | –2.279230000 |
| C | 0.784200000 | 3.337466000 | –2.685930000 |
| C | 0.000000000 | 4.328640000 | –3.524270000 |
| C | 0.799310000 | 4.970010000 | –4.670110000 |
| C | –0.075560000 | 5.924890000 | –5.490970000 |
| C | –0.702020000 | 7.009500000 | –4.606270000 |
| C | –1.490240000 | 6.393140000 | –3.444520000 |
| C | –0.623000000 | 5.424700000 | –2.635960000 |
| C | 0.138937000 | 2.170974000 | –2.254320000 |
| C | 0.710260000 | –1.272360000 | –1.389730000 |
| C | 1.810650000 | –1.205810000 | –2.254320000 |
| C | 2.498230000 | –2.347870000 | –2.685930000 |
| C | 3.748712000 | –2.164320000 | –3.524270000 |
| C | 6.281742000 | –1.905984000 | –3.444520000 |
| C | 6.421415000 | –2.896783000 | –4.606270000 |
| C | 5.168885000 | –2.897008000 | –5.490970000 |
| C | 5.009428000 | –2.172816000 | –2.635960000 |
| C | 3.904500000 | –3.177228000 | –4.670110000 |
| C | 2.045160000 | –3.608250000 | –2.279230000 |
| C | 0.897610000 | –3.721470000 | –1.483990000 |
| C | 0.257840000 | –5.046010000 | –1.114090000 |
| C | 1.211100000 | –6.247470000 | –1.081510000 |
| C | 0.479180000 | –7.527210000 | –0.658670000 |
| C | –1.677640000 | –6.613250000 | –1.623280000 |
| C | –0.724190000 | –7.812690000 | –1.565970000 |
| C | –0.940990000 | –5.338210000 | –2.042040000 |
| C | 0.254460000 | –2.557510000 | –1.053960000 |
| C | –1.949587000 | –0.965164000 | –2.254320000 |
| C | –3.282430000 | –0.989596000 | –2.685930000 |
| C | –3.748712000 | –2.164320000 | –3.524270000 |
| C | –4.791502000 | –4.487156000 | –3.444520000 |
| C | –5.719395000 | –4.112717000 | –4.606270000 |
| C | –5.093325000 | –3.027882000 | –5.490970000 |
| C | –4.386428000 | –3.251884000 | –2.635960000 |
| C | –4.703810000 | –1.792782000 | –4.670110000 |
| C | –4.147416000 | 0.032964000 | –2.279230000 |
| C | –3.671693000 | 1.083382000 | –1.483990000 |

| | | | |
|---|---|---|---|
| C | –4.498893000 | 2.299709000 | –1.114090000 |
| C | –4.888423000 | 4.759504000 | –1.623280000 |
| C | –6.403893000 | 4.533512000 | –1.565970000 |
| C | –6.758345000 | 3.348623000 | –0.658670000 |
| C | –4.152530000 | 3.484026000 | –2.042040000 |
| C | –6.016018000 | 2.074892000 | –1.081510000 |
| C | –2.342099000 | 1.058386000 | –1.053960000 |
| H | –2.173090000 | 0.242590000 | 2.601540000 |
| H | –2.575010000 | 4.504300000 | 2.602570000 |
| H | 0.634820000 | 2.676470000 | 0.446370000 |
| H | 0.155770000 | 4.924670000 | 0.098240000 |
| H | –1.646400000 | 6.403510000 | 2.085100000 |
| H | –2.053730000 | 6.040100000 | 0.400600000 |
| H | 0.572200000 | 5.436870000 | 3.079960000 |
| H | 1.627630000 | 4.477160000 | 2.033650000 |
| H | –0.129200000 | 7.416690000 | –0.383330000 |
| H | –1.175420000 | 8.382210000 | 0.665900000 |
| H | 2.126510000 | 6.455210000 | 0.624560000 |
| H | 2.513090000 | 6.815360000 | 2.314270000 |
| H | 1.258480000 | 8.713420000 | 1.220560000 |
| H | 0.361810000 | 8.033620000 | 2.587530000 |
| H | 4.527121000 | –5.069217000 | 0.624560000 |
| H | 4.645730000 | –5.584080000 | 2.314270000 |
| H | 4.422368000 | –3.213975000 | 3.079960000 |
| H | 3.063519000 | –3.648149000 | 2.033650000 |
| H | 6.916803000 | –5.446586000 | 1.220560000 |
| H | 6.776414000 | –4.330147000 | 2.587530000 |
| H | 5.188343000 | –0.022126000 | 2.602570000 |
| H | 4.187004000 | –2.597236000 | 0.098240000 |
| H | 1.296634000 | 1.760656000 | 2.601540000 |
| H | 2.000481000 | –1.888005000 | 0.446370000 |
| H | 6.487642000 | –3.596455000 | –0.383330000 |
| H | 7.846917000 | –3.173161000 | 0.665900000 |
| H | 6.368802000 | –1.775931000 | 2.085100000 |
| H | 6.257745000 | –1.241468000 | 0.400600000 |
| H | –6.358442000 | –3.820235000 | –0.383330000 |
| H | –6.671497000 | –5.209049000 | 0.665900000 |
| H | –4.722402000 | –4.627579000 | 2.085100000 |
| H | –4.204015000 | –4.798632000 | 0.400600000 |
| H | –8.175283000 | –3.266834000 | 1.220560000 |
| H | –7.138224000 | –3.703473000 | 2.587530000 |
| H | –2.613333000 | –4.482174000 | 2.602570000 |
| H | –4.342774000 | –2.327434000 | 0.098240000 |
| H | –6.653631000 | –1.385993000 | 0.624560000 |
| H | –7.158820000 | –1.231280000 | 2.314270000 |
| H | –4.994568000 | –2.222895000 | 3.079960000 |
| H | –4.691149000 | –0.829011000 | 2.033650000 |
| H | 0.876456000 | –2.003246000 | 2.601540000 |
| H | –2.635301000 | –0.788465000 | 0.446370000 |
| H | 0.880830000 | –5.347050000 | 5.981630000 |
| H | –0.517400000 | –6.384640000 | 6.298900000 |
| H | –1.227870000 | –4.183360000 | 5.312350000 |
| H | –1.651220000 | –5.537480000 | 4.254220000 |
| H | 0.837990000 | –3.771100000 | 3.981320000 |
| H | –0.101790000 | –7.650090000 | 4.196980000 |
| H | 1.352580000 | –7.666870000 | 5.206740000 |
| H | 1.891080000 | –7.182330000 | 2.786430000 |
| H | 2.362910000 | –5.844270000 | 3.844720000 |
| H | –0.194860000 | –5.979450000 | 2.141500000 |
| H | 1.211620000 | –4.956840000 | 1.831490000 |
| H | –5.080926000 | 3.158479000 | 2.141500000 |
| H | –4.898559000 | 1.429126000 | 1.831490000 |
| H | –3.684863000 | 1.159829000 | 3.981320000 |
| H | –3.008961000 | 3.155047000 | 5.312350000 |
| H | –3.969988000 | 4.198738000 | 4.254220000 |
| H | –7.165620000 | 1.953442000 | 2.786430000 |
| H | –6.242741000 | 0.875795000 | 3.844720000 |
| H | –6.574277000 | 3.913198000 | 4.196980000 |
| H | –7.315994000 | 2.662066000 | 5.206740000 |
| H | –5.071096000 | 1.910704000 | 5.981630000 |
| H | –5.270560000 | 3.640402000 | 6.298900000 |
| H | 2.846873000 | 2.611271000 | 3.981320000 |
| H | 5.275786000 | 2.820971000 | 2.141500000 |
| H | 3.686939000 | 3.527714000 | 1.831490000 |
| H | 4.236831000 | 1.028313000 | 5.312350000 |
| H | 5.621208000 | 1.338742000 | 4.254220000 |
| H | 5.274540000 | 5.228888000 | 2.786430000 |
| H | 3.879831000 | 4.968475000 | 3.844720000 |
| H | 4.190266000 | 3.436346000 | 5.981630000 |
| H | 5.787960000 | 2.744238000 | 6.298900000 |
| H | 6.676067000 | 3.736892000 | 4.196980000 |
| H | 5.963414000 | 5.004804000 | 5.206740000 |
| H | 2.635301000 | 0.788465000 | –0.446370000 |
| H | 4.342774000 | 2.327434000 | –0.098240000 |
| H | 6.653631000 | 1.385993000 | –0.624560000 |
| H | 7.158820000 | 1.231280000 | –2.314270000 |
| H | 8.175283000 | 3.266834000 | –1.220560000 |
| H | 7.138224000 | 3.703473000 | –2.587530000 |
| H | 6.358442000 | 3.820235000 | 0.383330000 |
| H | 6.671497000 | 5.209049000 | –0.665900000 |
| H | 4.994568000 | 2.222895000 | –3.079960000 |
| H | 4.691149000 | 0.829011000 | –2.033650000 |
| H | 4.722402000 | 4.627579000 | –2.085100000 |
| H | 4.204015000 | 4.798632000 | –0.400600000 |
| H | 2.613333000 | 4.482174000 | –2.602570000 |
| H | –0.837990000 | 3.771100000 | –3.981320000 |
| H | 1.227870000 | 4.183360000 | –5.312350000 |
| H | 1.651220000 | 5.537480000 | –4.254220000 |
| H | –0.880830000 | 5.347050000 | –5.981630000 |
| H | 0.517400000 | 6.384640000 | –6.298900000 |
| H | 0.101790000 | 7.650090000 | –4.196980000 |
| H | –1.352580000 | 7.666870000 | –5.206740000 |
| H | –1.891080000 | 7.182330000 | –2.786430000 |
| H | –2.362910000 | 5.844270000 | –3.844720000 |
| H | 0.194860000 | 5.979450000 | –2.141500000 |
| H | –1.211620000 | 4.956840000 | –1.831490000 |
| H | –0.876456000 | 2.003246000 | –2.601540000 |
| H | 2.173090000 | –0.242590000 | –2.601540000 |
| H | 3.684863000 | –1.159829000 | –3.981320000 |
| H | 7.165620000 | –1.953442000 | –2.786430000 |
| H | 6.242741000 | –0.875795000 | –3.844720000 |
| H | 6.574277000 | –3.913198000 | –4.196980000 |
| H | 7.315994000 | –2.662066000 | –5.206740000 |
| H | 5.071096000 | –1.910704000 | –5.981630000 |
| H | 5.270560000 | –3.640402000 | –6.298900000 |
| H | 5.080926000 | –3.158479000 | –2.141500000 |
| H | 4.898559000 | –1.429126000 | –1.831490000 |
| H | 3.008961000 | –3.155047000 | –5.312350000 |
| H | 3.969988000 | –4.198738000 | –4.254220000 |
| H | 2.575010000 | –4.504300000 | –2.602570000 |
| H | –0.155770000 | –4.924670000 | –0.098240000 |
| H | 1.646400000 | –6.403510000 | –2.085100000 |
| H | 2.053730000 | –6.040100000 | –0.400600000 |
| H | 0.129200000 | –7.416690000 | 0.383330000 |
| H | 1.175420000 | –8.382210000 | –0.665900000 |
| H | –2.126510000 | –6.455210000 | –0.624560000 |
| H | –2.513090000 | –6.815360000 | –2.314270000 |
| H | –1.258480000 | –8.713420000 | –1.220560000 |
| H | –0.361810000 | –8.033620000 | –2.587530000 |
| H | –0.572200000 | –5.436870000 | –3.079960000 |
| H | –1.627630000 | –4.477160000 | –2.033650000 |
| H | –0.634820000 | –2.676470000 | –0.446370000 |
| H | –1.296634000 | –1.760656000 | –2.601540000 |
| H | –2.846873000 | –2.611271000 | –3.981320000 |
| H | –5.274540000 | –5.228888000 | –2.786430000 |
| H | –3.879831000 | –4.968475000 | –3.844720000 |
| H | –6.676067000 | –3.736892000 | –4.196980000 |
| H | –5.963414000 | –5.004804000 | –5.206740000 |
| H | –4.190266000 | –3.436346000 | –5.981630000 |
| H | –5.787960000 | –2.744238000 | –6.298900000 |
| H | –5.275786000 | –2.820971000 | –2.141500000 |
| H | –3.686939000 | –3.527714000 | –1.831490000 |
| H | –4.236831000 | –1.028313000 | –5.312350000 |
| H | –5.621208000 | –1.338742000 | –4.254220000 |
| H | –5.188343000 | 0.022126000 | –2.602570000 |
| H | –4.187004000 | 2.597236000 | –0.098240000 |
| H | –4.527121000 | 5.069217000 | –0.624560000 |
| H | –4.645730000 | 5.584080000 | –2.314270000 |
| H | –6.916803000 | 5.446586000 | –1.220560000 |
| H | –6.776414000 | 4.330147000 | –2.587530000 |
| H | –6.487642000 | 3.596455000 | 0.383330000 |
| H | –7.846917000 | 3.173161000 | –0.665900000 |
| H | –4.422368000 | 3.213975000 | –3.079960000 |
| H | –3.063519000 | 3.648149000 | –2.033650000 |
| H | –6.368802000 | 1.775931000 | –2.085100000 |
| H | –6.257745000 | 1.241468000 | –0.400600000 |
| H | –2.000481000 | 1.888005000 | –0.446370000 |

M06-2X

| | | | |
|---|---|---|---|
| C | 0.000000000 | 0.000000000 | 0.855220000 |
| C | –1.825956000 | 1.212508000 | 2.238710000 |
| C | –2.519998000 | 2.356518000 | 2.647670000 |
| C | –0.713930000 | 1.271647000 | 1.388710000 |
| C | –2.076956000 | 3.611505000 | 2.220660000 |
| C | –0.254355000 | 2.554576000 | 1.057570000 |
| C | –0.914719000 | 3.719441000 | 1.451730000 |
| C | –0.280581000 | 5.041278000 | 1.068150000 |
| C | –1.235258000 | 6.236563000 | 1.070730000 |
| C | 0.937289000 | 5.333335000 | 1.962050000 |
| C | –0.523106000 | 7.515770000 | 0.626880000 |
| C | 1.652572000 | 6.610566000 | 1.522480000 |
| C | 0.695578000 | 7.802952000 | 1.504960000 |
| C | 4.898633000 | –4.736452000 | 1.522480000 |
| C | 4.150159000 | –3.478384000 | 1.962050000 |
| C | 6.409766000 | –4.503864000 | 1.504960000 |

| | | | |
|---|---|---|---|
| C | 4.166133000 | –0.007056000 | 2.220660000 |
| C | 4.506165000 | –2.277649000 | 1.068150000 |
| C | 3.300803000 | 1.004123000 | 2.647670000 |
| C | 3.678490000 | –1.067551000 | 1.451730000 |
| C | 1.963040000 | 0.975070000 | 2.238710000 |
| C | 2.339505000 | –1.057010000 | 1.057570000 |
| C | 1.458244000 | –0.017542000 | 1.388710000 |
| C | 6.770401000 | –3.304862000 | 0.626880000 |
| C | 6.018651000 | –2.048517000 | 1.070730000 |
| C | –6.247294000 | –4.210908000 | 0.626880000 |
| C | –4.783393000 | –4.188047000 | 1.070730000 |
| C | –7.105344000 | –3.299088000 | 1.504960000 |
| C | –2.089177000 | –3.604450000 | 2.220660000 |
| C | –4.225584000 | –2.763629000 | 1.068150000 |
| C | –0.780805000 | –3.360641000 | 2.647670000 |
| C | –6.551204000 | –1.874114000 | 1.522480000 |
| C | –2.763771000 | –2.651890000 | 1.451730000 |
| C | –5.087448000 | –1.854951000 | 1.962050000 |
| C | –0.137085000 | –2.187578000 | 2.238710000 |
| C | –2.085150000 | –1.497566000 | 1.057570000 |
| C | –0.744314000 | –1.254105000 | 1.388710000 |
| C | 0.000000000 | –5.852775000 | 5.540810000 |
| C | –0.829111000 | –4.884680000 | 4.695910000 |
| C | –0.021600000 | –4.352842000 | 3.504860000 |
| C | 0.521305000 | –7.014620000 | 4.694400000 |
| C | 1.326258000 | –6.503097000 | 3.499610000 |
| C | 0.507139000 | –5.523422000 | 2.660790000 |
| C | –5.036993000 | 2.322515000 | 2.660790000 |
| C | –3.758872000 | 2.195127000 | 3.504860000 |
| C | –3.815701000 | 3.160371000 | 4.695910000 |
| C | –6.294976000 | 2.102975000 | 3.499610000 |
| C | –6.335492000 | 3.055846000 | 4.694400000 |
| C | –5.068652000 | 2.926387000 | 5.540810000 |
| C | 3.780472000 | 2.157715000 | 3.504860000 |
| C | 4.529854000 | 3.200907000 | 2.660790000 |
| C | 4.644812000 | 1.724308000 | 4.695910000 |
| C | 4.968718000 | 4.400122000 | 3.499610000 |
| C | 5.068652000 | 2.926387000 | 5.540810000 |
| C | 5.814186000 | 3.958774000 | 4.694400000 |
| C | –1.458244000 | 0.017542000 | –1.388710000 |
| C | 0.000000000 | 0.000000000 | –0.855220000 |
| C | 0.744314000 | 1.254105000 | –1.388710000 |
| C | 2.085150000 | 1.497566000 | –1.057570000 |
| C | 2.763771000 | 2.651890000 | –1.451730000 |
| C | 4.225584000 | 2.763629000 | –1.068150000 |
| C | 6.551204000 | 1.874114000 | –1.522480000 |
| C | 7.105344000 | 3.299088000 | –1.504960000 |
| C | 6.247294000 | 4.210908000 | –0.626880000 |
| C | 5.087448000 | 1.854951000 | –1.962050000 |
| C | 4.783393000 | 4.188047000 | –1.070730000 |
| C | 2.089177000 | 3.604450000 | –2.220660000 |
| C | 0.780805000 | 3.360641000 | –2.647670000 |
| C | 0.021600000 | 4.352842000 | –3.504860000 |
| C | 0.829111000 | 4.884680000 | –4.695910000 |
| C | 0.000000000 | 5.852775000 | –5.540810000 |
| C | –0.521305000 | 7.014620000 | –4.694400000 |
| C | –1.326258000 | 6.503097000 | –3.499610000 |
| C | –0.507139000 | 5.523422000 | –2.660790000 |
| C | 0.137085000 | 2.187578000 | –2.238710000 |
| C | 0.713930000 | –1.271647000 | –1.388710000 |
| C | 1.825956000 | –1.212508000 | –2.238710000 |
| C | 2.519998000 | –2.356518000 | –2.647670000 |
| C | 3.758872000 | –2.195127000 | –3.504860000 |
| C | 6.294976000 | –2.102975000 | –3.499610000 |
| C | 6.335492000 | –3.055846000 | –4.694400000 |
| C | 5.068652000 | –2.926387000 | –5.540810000 |
| C | 5.036993000 | –2.322515000 | –2.660790000 |
| C | 3.815701000 | –3.160371000 | –4.695910000 |
| C | 2.076956000 | –3.611505000 | –2.220660000 |
| C | 0.914719000 | –3.719441000 | –1.451730000 |
| C | 0.280581000 | –5.041278000 | –1.068150000 |
| C | 1.235258000 | –6.236563000 | –1.070730000 |
| C | 0.523106000 | –7.515770000 | –0.626880000 |
| C | –1.652572000 | –6.610566000 | –1.522480000 |
| C | –0.695578000 | –7.802952000 | –1.504960000 |
| C | –0.937289000 | –5.333335000 | –1.962050000 |
| C | 0.254355000 | –2.554576000 | –1.057570000 |
| C | –1.963040000 | –0.975070000 | –2.238710000 |
| C | –3.300803000 | –1.004123000 | –2.647670000 |
| C | –3.780472000 | –2.157715000 | –3.504860000 |
| C | –4.968718000 | –4.400122000 | –3.499610000 |
| C | –5.814186000 | –3.958774000 | –4.694400000 |
| C | –5.068652000 | –2.926387000 | –5.540810000 |
| C | –4.529854000 | –3.200907000 | –2.660790000 |
| C | –4.644812000 | –1.724308000 | –4.695910000 |
| C | –4.166133000 | 0.007056000 | –2.220660000 |
| C | –3.678490000 | 1.067551000 | –1.451730000 |
| C | –4.506165000 | 2.277649000 | –1.068150000 |
| C | –4.898633000 | 4.736452000 | –1.522480000 |
| C | –6.409766000 | 4.503864000 | –1.504960000 |
| C | –6.770401000 | 3.304862000 | –0.626880000 |
| C | –4.150159000 | 3.478384000 | –1.962050000 |
| C | –6.018651000 | 2.048517000 | –1.070730000 |
| C | –2.339505000 | 1.057010000 | –1.057570000 |
| H | –2.189765000 | 0.254966000 | 2.605410000 |
| H | –2.624005000 | 4.508911000 | 2.515850000 |
| H | 0.657645000 | 2.681119000 | 0.480410000 |
| H | 0.107981000 | 4.923348000 | 0.040210000 |
| H | –1.629991000 | 6.390171000 | 2.090970000 |
| H | –2.101703000 | 6.027820000 | 0.421130000 |
| H | 0.593936000 | 5.430848000 | 3.008490000 |
| H | 1.628514000 | 4.474290000 | 1.931590000 |
| H | –0.195363000 | 7.401195000 | –0.421180000 |
| H | –1.221836000 | 8.365779000 | 0.649700000 |
| H | 2.057301000 | 6.456634000 | 0.505580000 |
| H | 2.511723000 | 6.814112000 | 2.180460000 |
| H | 1.215398000 | 8.708070000 | 1.155610000 |
| H | 0.356460000 | 8.008453000 | 2.535990000 |
| H | 4.562958000 | –5.009992000 | 0.505580000 |
| H | 4.645332000 | –5.582272000 | 2.180460000 |
| H | 4.406284000 | –3.229788000 | 3.008490000 |
| H | 3.060591000 | –3.647480000 | 1.931590000 |
| H | 6.933711000 | –5.406600000 | 1.155610000 |
| H | 6.757293000 | –4.312930000 | 2.535990000 |
| H | 5.216834000 | 0.017999000 | 2.515850000 |
| H | 4.209754000 | –2.555189000 | 0.040210000 |
| H | 1.315689000 | 1.768909000 | 2.605410000 |
| H | 1.993094000 | –1.910097000 | 0.480410000 |
| H | 6.507305000 | –3.531408000 | –0.421180000 |
| H | 7.855895000 | –3.124748000 | 0.649700000 |
| H | 6.349046000 | –1.783472000 | 2.090970000 |
| H | 6.271097000 | –1.193782000 | 0.421130000 |
| H | –6.311941000 | –3.869787000 | –0.421180000 |
| H | –6.634059000 | –5.241031000 | 0.649700000 |
| H | –4.719055000 | –4.606699000 | 2.090970000 |
| H | –4.169394000 | –4.834038000 | 0.421130000 |
| H | –8.149108000 | –3.301470000 | 1.155610000 |
| H | –7.113753000 | –3.695523000 | 2.535990000 |
| H | –2.592829000 | –4.526910000 | 2.515850000 |
| H | –4.317735000 | –2.368160000 | 0.040210000 |
| H | –6.620260000 | –1.446642000 | 0.505580000 |
| H | –7.157056000 | –1.231840000 | 2.180460000 |
| H | –5.000220000 | –2.201060000 | 3.008490000 |
| H | –4.689106000 | –0.826810000 | 1.931590000 |
| H | 0.874075000 | –2.023875000 | 2.605410000 |
| H | –2.650740000 | –0.771022000 | 0.480410000 |
| H | 0.857375000 | –5.309960000 | 5.976540000 |
| H | –0.598671000 | –6.229477000 | 6.383900000 |
| H | –1.189741000 | –4.041799000 | 5.305450000 |
| H | –1.723684000 | –5.415031000 | 4.324220000 |
| H | 0.857794000 | –3.822175000 | 3.912910000 |
| H | –0.336539000 | –7.604614000 | 4.324610000 |
| H | 1.133089000 | –7.693747000 | 5.307570000 |
| H | 1.670163000 | –7.342640000 | 2.874920000 |
| H | 2.231980000 | –5.987782000 | 3.867300000 |
| H | –0.359375000 | –6.047223000 | 2.214350000 |
| H | 1.108159000 | –5.132930000 | 1.822580000 |
| H | –5.057362000 | 3.334840000 | 2.214350000 |
| H | –4.999327000 | 1.606771000 | 1.822580000 |
| H | –3.738998000 | 1.168216000 | 3.912910000 |
| H | –2.905430000 | 3.051246000 | 5.305450000 |
| H | –3.827712000 | 4.200270000 | 4.324220000 |
| H | –7.193994000 | 2.224917000 | 2.874920000 |
| H | –6.301561000 | 1.060940000 | 3.867300000 |
| H | –6.417519000 | 4.093759000 | 4.324610000 |
| H | –7.229525000 | 2.865590000 | 5.307570000 |
| H | –5.027248000 | 1.912471000 | 5.976540000 |
| H | –5.095549000 | 3.633203000 | 6.383900000 |
| H | 2.881204000 | 2.653959000 | 3.912910000 |
| H | 5.416737000 | 2.712384000 | 2.214350000 |
| H | 3.891168000 | 3.526159000 | 1.822580000 |
| H | 4.095171000 | 0.990554000 | 5.305450000 |
| H | 5.551396000 | 1.214761000 | 4.324220000 |
| H | 5.523831000 | 5.117724000 | 2.874920000 |
| H | 4.069581000 | 4.926842000 | 3.867300000 |
| H | 4.169873000 | 3.397489000 | 5.976540000 |
| H | 5.694221000 | 2.596274000 | 6.383900000 |
| H | 6.754058000 | 3.510855000 | 4.324610000 |
| H | 6.096436000 | 4.828157000 | 5.307570000 |
| H | 2.650740000 | 0.771022000 | –0.480410000 |
| H | 4.317735000 | 2.368160000 | –0.040210000 |
| H | 6.620260000 | 1.446642000 | –0.505580000 |
| H | 7.157056000 | 1.231840000 | –2.180460000 |
| H | 8.149108000 | 3.301470000 | –1.155610000 |
| H | 7.113753000 | 3.695523000 | –2.535990000 |
| H | 6.311941000 | 3.869787000 | 0.421180000 |

| | | | |
|---|---|---|---|
| H | 6.634059000 | 5.241031000 | −0.649700000 |
| H | 5.000220000 | 2.201060000 | −3.008490000 |
| H | 4.689106000 | 0.826810000 | −1.931590000 |
| H | 4.719055000 | 4.606699000 | −2.090970000 |
| H | 4.169394000 | 4.834038000 | −0.421130000 |
| H | 2.592829000 | 4.526910000 | −2.515850000 |
| H | −0.857794000 | 3.822175000 | −3.912910000 |
| H | 1.189741000 | 4.041799000 | −5.305450000 |
| H | 1.723684000 | 5.415031000 | −4.324220000 |
| H | −0.857375000 | 5.309960000 | −5.976540000 |
| H | 0.598671000 | 6.229477000 | −6.383900000 |
| H | 0.336539000 | 7.604614000 | −4.324610000 |
| H | −1.133089000 | 7.693747000 | −5.307570000 |
| H | −1.670163000 | 7.342640000 | −2.874920000 |
| H | −2.231980000 | 5.987782000 | −3.867300000 |
| H | 0.359375000 | 6.047223000 | −2.214350000 |
| H | −1.108159000 | 5.132930000 | −1.822580000 |
| H | −0.874075000 | 2.023875000 | −2.605410000 |
| H | 2.189765000 | −0.254966000 | −2.605410000 |
| H | 3.738998000 | −1.168216000 | −3.912910000 |
| H | 7.193994000 | −2.224917000 | −2.874920000 |
| H | 6.301561000 | −1.060940000 | −3.867300000 |
| H | 6.417519000 | −4.093759000 | −4.324610000 |
| H | 7.229525000 | −2.865590000 | −5.307570000 |
| H | 5.027248000 | −1.912471000 | −5.976540000 |
| H | 5.095549000 | −3.633203000 | −6.383900000 |
| H | 5.057362000 | −3.334840000 | −2.214350000 |
| H | 4.999327000 | −1.606771000 | −1.822580000 |
| H | 2.905430000 | −3.051246000 | −5.305450000 |
| H | 3.827712000 | −4.200270000 | −4.324220000 |
| H | 2.624005000 | −4.508911000 | −2.515850000 |
| H | −0.107981000 | −4.923348000 | −0.040210000 |
| H | 1.629991000 | −6.390171000 | −2.090970000 |
| H | 2.101703000 | −6.027820000 | −0.421130000 |
| H | 0.195363000 | −7.401195000 | 0.421180000 |
| H | 1.221836000 | −8.365779000 | −0.649700000 |
| H | −2.057301000 | −6.456634000 | −0.505580000 |
| H | −2.511723000 | −6.814112000 | −2.180460000 |
| H | −1.215398000 | −8.708070000 | −1.155610000 |
| H | −0.356460000 | −8.008453000 | −2.535990000 |
| H | −0.593936000 | −5.430848000 | −3.008490000 |
| H | −1.628514000 | −4.474290000 | −1.931590000 |
| H | −0.657645000 | −2.681119000 | −0.480410000 |
| H | −1.315689000 | −1.768909000 | −2.605410000 |
| H | −2.881204000 | −2.653959000 | −3.912910000 |
| H | −5.523831000 | −5.117724000 | −2.874920000 |
| H | −4.069581000 | −4.926842000 | −3.867300000 |
| H | −6.754058000 | −3.510855000 | −4.324610000 |
| H | −6.096436000 | −4.828157000 | −5.307570000 |
| H | −4.169873000 | −3.397489000 | −5.976540000 |
| H | −5.694221000 | −2.596274000 | −6.383900000 |
| H | −5.416737000 | −2.712384000 | −2.214350000 |
| H | −3.891168000 | −3.526159000 | −1.822580000 |
| H | −4.095171000 | −0.990554000 | −5.305450000 |
| H | −5.551396000 | −1.214761000 | −4.324220000 |
| H | −5.216834000 | −0.017999000 | −2.515850000 |
| H | −4.209754000 | 2.555189000 | −0.040210000 |
| H | −4.562958000 | 5.009992000 | −0.505580000 |
| H | −4.645332000 | 5.582272000 | −2.180460000 |
| H | −6.933711000 | 5.406600000 | −1.155610000 |
| H | −6.757293000 | 4.312930000 | −2.535990000 |
| H | −6.507305000 | 3.531408000 | 0.421180000 |
| H | −7.855895000 | 3.124748000 | −0.649700000 |
| H | −4.406284000 | 3.229788000 | −3.008490000 |
| H | −3.060591000 | 3.647480000 | −1.931590000 |
| H | −6.349046000 | 1.783472000 | −2.090970000 |
| H | −6.271097000 | 1.193782000 | −0.421130000 |
| H | −1.993094000 | 1.910097000 | −0.480410000 |

Ad-**1**$_2$

B3LYP

| | | | |
|---|---|---|---|
| C | 0.000000000 | 0.000000000 | 0.853240000 |
| C | −0.961212000 | −1.706731000 | 2.555570000 |
| C | −1.997419000 | −2.348775000 | 3.255590000 |
| C | −1.193080000 | −0.844302000 | 1.468910000 |
| C | −3.311125000 | −2.118780000 | 2.834770000 |
| C | −2.538526000 | −0.639095000 | 1.096620000 |
| C | −3.606806000 | −1.266955000 | 1.759130000 |
| C | −5.251186000 | −0.109608000 | 0.152440000 |
| C | −5.081073000 | −1.016036000 | 1.393730000 |
| C | −5.813571000 | −2.357864000 | 1.099900000 |
| C | −5.804976000 | −0.322402000 | 2.585370000 |
| C | −6.741111000 | 0.127621000 | −0.166840000 |
| C | −7.435337000 | −1.218210000 | −0.447730000 |
| C | −7.424503000 | 0.813949000 | 1.030960000 |
| C | −7.305815000 | −2.122154000 | 0.791920000 |

| | | | |
|---|---|---|---|
| C | −7.295820000 | −0.087622000 | 2.272740000 |
| C | −7.980725000 | −1.440580000 | 1.998300000 |
| C | 3.572027000 | 6.362177000 | 2.272740000 |
| C | 2.623280000 | 5.188458000 | 2.585370000 |
| C | 4.417152000 | 6.022834000 | 1.030960000 |
| C | 2.742784000 | 7.631801000 | 1.998300000 |
| C | −0.179354000 | 3.926908000 | 2.834770000 |
| C | 1.660624000 | 4.908357000 | 1.393730000 |
| C | −1.035389000 | 2.904203000 | 3.255590000 |
| C | 3.481078000 | 5.774163000 | −0.166840000 |
| C | 0.706188000 | 3.757063000 | 1.759130000 |
| C | 2.530669000 | 4.602465000 | 0.152440000 |
| C | −0.997466000 | 1.685800000 | 2.555570000 |
| C | 0.715791000 | 2.517975000 | 1.096620000 |
| C | −0.134646000 | 1.455389000 | 1.468910000 |
| C | 1.815068000 | 7.388098000 | 0.791920000 |
| C | 0.864815000 | 6.213632000 | 1.099900000 |
| C | 2.662668000 | 7.048296000 | −0.447730000 |
| C | 5.490747000 | −5.265944000 | 0.791920000 |
| C | 4.948756000 | −3.855768000 | 1.099900000 |
| C | 5.237941000 | −6.191221000 | 1.998300000 |
| C | 4.772669000 | −5.830086000 | −0.447730000 |
| C | 3.490480000 | −1.808129000 | 2.834770000 |
| C | 3.420450000 | −3.892321000 | 1.393730000 |
| C | 3.032808000 | −0.555429000 | 3.255590000 |
| C | 3.723793000 | −6.274555000 | 2.272740000 |
| C | 2.900618000 | −2.490108000 | 1.759130000 |
| C | 3.181696000 | −4.866056000 | 2.585370000 |
| C | 1.958678000 | 0.020931000 | 2.555570000 |
| C | 1.822735000 | −1.878881000 | 1.096620000 |
| C | 1.327727000 | −0.611087000 | 1.468910000 |
| C | 3.260033000 | −5.901784000 | −0.166840000 |
| C | 2.720517000 | −4.492856000 | 0.152440000 |
| C | 3.007351000 | −6.836782000 | 1.030960000 |
| C | 3.595821000 | 1.195180000 | 7.266980000 |
| C | 4.292026000 | −0.137582000 | 6.935360000 |
| C | 3.604288000 | −0.789846000 | 5.719710000 |
| C | 3.695075000 | 2.133355000 | 6.049610000 |
| C | 3.012888000 | 1.476286000 | 4.833230000 |
| C | 3.687300000 | 0.130977000 | 4.466760000 |
| C | 5.775581000 | 0.123955000 | 6.607950000 |
| C | 5.177374000 | 2.403308000 | 5.728800000 |
| C | 5.874968000 | 1.071035000 | 5.396130000 |
| C | 5.189245000 | 0.420994000 | 4.178070000 |
| C | −2.230031000 | −4.704514000 | 4.178070000 |
| C | −1.730220000 | −3.258784000 | 4.466760000 |
| C | −2.486170000 | −2.726482000 | 5.719710000 |
| C | −2.009940000 | −5.623390000 | 5.396130000 |
| C | −0.227943000 | −3.347381000 | 4.833230000 |
| C | −2.780442000 | −5.063777000 | 6.607950000 |
| C | −2.265162000 | −3.648213000 | 6.935360000 |
| C | −0.507362000 | −5.685391000 | 5.728800000 |
| C | 0.000003000 | −4.266706000 | 6.049610000 |
| C | −0.762855000 | −3.711663000 | 7.266980000 |
| C | −2.784945000 | 1.871095000 | 4.833230000 |
| C | −1.957079000 | 3.127807000 | 4.466760000 |
| C | −2.959213000 | 4.283521000 | 4.178070000 |
| C | −3.695077000 | 2.133351000 | 6.049610000 |
| C | −1.118117000 | 3.516328000 | 5.719710000 |
| C | −4.670012000 | 3.282084000 | 5.728800000 |
| C | −3.865028000 | 4.552354000 | 5.396130000 |
| C | −2.832967000 | 2.516483000 | 7.266980000 |
| C | −2.026864000 | 3.785794000 | 6.935360000 |
| C | −2.995139000 | 4.939822000 | 6.607950000 |
| C | 0.134646000 | −1.455389000 | −1.468910000 |
| C | 0.000000000 | 0.000000000 | −0.853240000 |
| C | −1.327727000 | 0.611087000 | −1.468910000 |
| C | −1.822735000 | 1.878881000 | −1.096620000 |
| C | −2.900618000 | 2.490108000 | −1.759130000 |
| C | −3.420450000 | 3.892321000 | −1.393730000 |
| C | −2.720517000 | 4.492856000 | −0.152440000 |
| C | −3.260033000 | 5.901784000 | 0.166840000 |
| C | −3.007351000 | 6.836782000 | −1.030960000 |
| C | −3.723793000 | 6.274555000 | −2.272740000 |
| C | −5.237941000 | 6.191221000 | −1.998300000 |
| C | −5.490747000 | 5.265944000 | −0.791920000 |
| C | −4.772669000 | 5.830086000 | 0.447730000 |
| C | −3.181696000 | 4.866056000 | −2.585370000 |
| C | −4.948756000 | 3.855768000 | −1.099900000 |
| C | −3.490480000 | 1.808129000 | −2.834770000 |
| C | −3.032808000 | 0.555429000 | −3.255590000 |
| C | −3.687300000 | −0.130977000 | −4.466760000 |
| C | −3.604288000 | 0.789846000 | −5.719710000 |
| C | −4.292026000 | 0.137582000 | −6.935360000 |
| C | −5.775581000 | −0.123955000 | −6.607950000 |
| C | −5.874968000 | −1.071035000 | −5.396130000 |
| C | −5.177374000 | −2.403308000 | −5.728800000 |
| C | −3.695075000 | −2.133355000 | −6.049610000 |

| | | | |
|---|---|---|---|
| C | –3.595821000 | –1.195180000 | –7.266980000 |
| C | –5.189245000 | –0.420994000 | –4.178070000 |
| C | –3.012888000 | –1.476286000 | –4.833230000 |
| C | –1.958678000 | –0.020931000 | –2.555570000 |
| C | 1.193080000 | 0.844302000 | –1.468910000 |
| C | 0.961212000 | 1.706731000 | –2.555570000 |
| C | 1.997419000 | 2.348775000 | –3.255590000 |
| C | 1.730220000 | 3.258784000 | –4.466760000 |
| C | 0.227943000 | 3.347381000 | –4.833230000 |
| C | –0.000003000 | 4.266706000 | –6.049610000 |
| C | 0.507362000 | 5.685391000 | –5.728800000 |
| C | 2.009940000 | 5.623390000 | –5.396130000 |
| C | 2.780442000 | 5.063777000 | –6.607950000 |
| C | 2.265162000 | 3.648213000 | –6.935360000 |
| C | 0.762855000 | 3.711663000 | –7.266980000 |
| C | 2.230031000 | 4.704514000 | –4.178070000 |
| C | 2.486170000 | 2.726482000 | –5.719710000 |
| C | 3.311125000 | 2.118780000 | –2.834770000 |
| C | 3.606806000 | 1.266955000 | –1.759130000 |
| C | 5.081073000 | 1.016036000 | –1.393730000 |
| C | 5.813571000 | 2.357864000 | –1.099900000 |
| C | 7.305815000 | 2.122154000 | –0.791920000 |
| C | 7.435337000 | 1.218210000 | 0.447730000 |
| C | 6.741111000 | –0.127621000 | 0.166840000 |
| C | 7.424503000 | –0.813949000 | –1.030960000 |
| C | 7.295820000 | 0.087622000 | –2.272740000 |
| C | 7.980725000 | 1.440580000 | –1.998300000 |
| C | 5.251186000 | 0.109608000 | –0.152440000 |
| C | 5.804976000 | 0.322402000 | –2.585370000 |
| C | 2.538526000 | 0.639095000 | –1.096620000 |
| C | 0.997466000 | –1.685800000 | –2.555570000 |
| C | 1.035389000 | –2.904203000 | –3.255590000 |
| C | 1.957079000 | –3.127807000 | –4.466760000 |
| C | 2.784945000 | –1.871095000 | –4.833230000 |
| C | 3.695077000 | –2.133351000 | –6.049610000 |
| C | 4.670012000 | –3.282084000 | –5.728800000 |
| C | 3.865028000 | –4.552354000 | –5.396130000 |
| C | 2.995139000 | –4.939822000 | –6.607950000 |
| C | 2.026864000 | –3.785794000 | –6.935360000 |
| C | 2.832967000 | –2.516483000 | –7.266980000 |
| C | 2.959213000 | –4.283521000 | –4.178070000 |
| C | 1.118117000 | –3.516328000 | –5.719710000 |
| C | 0.179354000 | –3.926908000 | –2.834770000 |
| C | –0.706188000 | –3.757063000 | –1.759130000 |
| C | –1.660624000 | –4.908357000 | –1.393730000 |
| C | –2.530669000 | –4.602465000 | –0.152440000 |
| C | –3.481078000 | –5.774163000 | 0.166840000 |
| C | –4.417152000 | –6.022834000 | –1.030960000 |
| C | –3.572027000 | –6.362177000 | –2.272740000 |
| C | –2.742784000 | –7.631801000 | –1.998300000 |
| C | –1.815068000 | –7.388098000 | –0.791920000 |
| C | –2.662668000 | –7.048296000 | 0.447730000 |
| C | –2.623280000 | –5.188458000 | –2.585370000 |
| C | –0.864815000 | –6.213632000 | –1.099900000 |
| C | –0.715791000 | –2.517975000 | –1.096620000 |
| H | 0.055563000 | –1.864116000 | 2.891380000 |
| H | –4.131283000 | –2.601380000 | 3.368110000 |
| H | –2.757943000 | 0.048036000 | 0.289430000 |
| H | –4.751080000 | –0.564221000 | –0.718430000 |
| H | –4.756592000 | 0.859504000 | 0.325520000 |
| H | –5.719687000 | –3.039914000 | 1.960600000 |
| H | –5.325555000 | –2.858589000 | 0.246320000 |
| H | –5.708514000 | –0.937242000 | 3.495490000 |
| H | –5.307457000 | 0.639290000 | 2.800060000 |
| H | –6.814459000 | 0.775551000 | –1.056430000 |
| H | –8.499743000 | –1.054597000 | –0.692160000 |
| H | –6.976478000 | –1.707911000 | –1.324510000 |
| H | –6.958619000 | 1.796185000 | 1.224540000 |
| H | –8.488744000 | 1.003671000 | 0.805350000 |
| H | –7.790295000 | –3.094226000 | 0.596440000 |
| H | –7.773220000 | 0.400932000 | 3.139570000 |
| H | –9.055428000 | –1.288254000 | 1.796140000 |
| H | –7.912223000 | –2.089252000 | 2.889520000 |
| H | 4.233827000 | 6.531340000 | 3.139570000 |
| H | 2.042581000 | 5.412340000 | 3.495490000 |
| H | 3.207370000 | 4.276747000 | 2.800060000 |
| H | 5.034851000 | 5.128248000 | 1.224540000 |
| H | 5.113576000 | 6.849632000 | 0.805350000 |
| H | 3.412053000 | 8.486358000 | 1.796140000 |
| H | 2.146767000 | 7.896812000 | 2.889520000 |
| H | –0.187220000 | 4.878486000 | 3.368110000 |
| H | 4.078876000 | 5.513719000 | –1.056430000 |
| H | 1.886910000 | 4.396667000 | –0.718430000 |
| H | 3.122649000 | 3.689578000 | 0.325520000 |
| H | –1.642154000 | 0.883939000 | 2.891380000 |
| H | 1.420572000 | 2.364431000 | 0.289430000 |
| H | 1.215469000 | 8.293706000 | 0.596440000 |
| H | 0.227201000 | 6.473351000 | 1.960600000 |
| H | 0.187167000 | 6.041361000 | 0.246320000 |
| H | 3.336564000 | 7.888292000 | –0.692160000 |
| H | 2.009145000 | 6.895762000 | –1.324510000 |
| H | 6.574826000 | –5.199480000 | 0.596440000 |
| H | 5.492486000 | –3.433437000 | 1.960600000 |
| H | 5.138388000 | –3.182772000 | 0.246320000 |
| H | 5.643375000 | –7.198104000 | 1.796140000 |
| H | 5.765457000 | –5.807561000 | 2.889520000 |
| H | 5.163179000 | –6.833695000 | –0.692160000 |
| H | 4.967333000 | –5.187851000 | –1.324510000 |
| H | 4.318503000 | –2.277106000 | 3.368110000 |
| H | 3.539393000 | –6.932271000 | 3.139570000 |
| H | 3.665933000 | –4.475097000 | 3.495490000 |
| H | 2.100087000 | –4.916038000 | 2.800060000 |
| H | 1.586590000 | 0.980178000 | 2.891380000 |
| H | 1.337371000 | –2.412467000 | 0.289430000 |
| H | 2.735582000 | –6.289270000 | –1.056430000 |
| H | 2.864170000 | –3.832446000 | –0.718430000 |
| H | 1.633944000 | –4.549082000 | 0.325520000 |
| H | 1.923768000 | –6.924433000 | 1.224540000 |
| H | 3.375167000 | –7.853303000 | 0.805350000 |
| H | 4.065864000 | 1.663351000 | 8.149740000 |
| H | 2.536952000 | 1.016394000 | 7.524040000 |
| H | 4.218599000 | –0.820873000 | 7.798890000 |
| H | 2.544052000 | –0.997086000 | 5.947480000 |
| H | 4.076700000 | –1.763039000 | 5.508210000 |
| H | 3.184779000 | 3.085744000 | 6.272280000 |
| H | 3.054189000 | 2.164798000 | 3.973490000 |
| H | 1.944760000 | 1.312200000 | 5.054040000 |
| H | 6.290468000 | –0.828418000 | 6.389320000 |
| H | 6.284683000 | 0.569066000 | 7.480790000 |
| H | 5.671861000 | 2.889703000 | 6.588050000 |
| H | 5.261133000 | 3.099047000 | 4.875390000 |
| H | 6.936014000 | 1.256278000 | 5.156080000 |
| H | 5.709206000 | –0.514759000 | 3.913090000 |
| H | 5.267338000 | 1.084368000 | 3.299250000 |
| H | –3.300397000 | –4.686937000 | 3.913090000 |
| H | –1.694579000 | –5.103832000 | 3.299250000 |
| H | –2.135528000 | –1.704670000 | 5.947480000 |
| H | –3.565187000 | –2.649006000 | 5.508210000 |
| H | –2.380038000 | –6.634903000 | 5.156080000 |
| H | 0.347676000 | –3.727404000 | 3.973490000 |
| H | 0.164019000 | –2.340311000 | 5.054040000 |
| H | –3.862664000 | –5.033496000 | 6.389320000 |
| H | –2.649516000 | –5.727228000 | 7.480790000 |
| H | –2.820196000 | –3.242978000 | 7.798890000 |
| H | –0.333375000 | –6.356827000 | 6.588050000 |
| H | 0.053287000 | –6.105798000 | 4.875390000 |
| H | 1.079943000 | –4.300971000 | 6.272280000 |
| H | –0.592428000 | –4.352817000 | 8.149740000 |
| H | –0.388253000 | –2.705262000 | 7.524040000 |
| H | –3.401864000 | 1.562606000 | 3.973490000 |
| H | –2.108778000 | 1.028111000 | 5.054040000 |
| H | –2.408808000 | 5.201697000 | 3.913090000 |
| H | –3.572759000 | 4.019465000 | 3.299250000 |
| H | –4.264722000 | 1.215227000 | 6.272280000 |
| H | –0.408524000 | 2.701757000 | 5.947480000 |
| H | –0.511513000 | 4.412045000 | 5.508210000 |
| H | –5.338487000 | 3.467125000 | 6.588050000 |
| H | –5.314420000 | 3.006751000 | 4.875390000 |
| H | –4.555976000 | 5.378625000 | 5.156080000 |
| H | –3.473436000 | 2.689466000 | 8.149740000 |
| H | –2.148699000 | 1.688868000 | 7.524040000 |
| H | –1.398403000 | 4.063850000 | 7.798890000 |
| H | –2.427803000 | 5.861914000 | 6.389320000 |
| H | –3.635167000 | 5.158162000 | 7.480790000 |
| H | –1.337371000 | 2.412467000 | –0.289430000 |
| H | –2.864170000 | 3.832446000 | 0.718430000 |
| H | –1.633944000 | 4.549082000 | –0.325520000 |
| H | –2.735582000 | 6.289270000 | 1.056430000 |
| H | –1.923768000 | 6.924433000 | –1.224540000 |
| H | –3.375167000 | 7.853303000 | –0.805350000 |
| H | –3.539393000 | 6.932271000 | –3.139570000 |
| H | –5.643375000 | 7.198104000 | –1.796140000 |
| H | –5.765457000 | 5.807561000 | –2.889520000 |
| H | –6.574826000 | 5.199480000 | –0.596440000 |
| H | –5.163179000 | 6.833695000 | 0.692160000 |
| H | –4.967333000 | 5.187851000 | 1.324510000 |
| H | –3.665933000 | 4.475097000 | –3.495490000 |
| H | –2.100087000 | 4.916038000 | –2.800060000 |
| H | –5.492486000 | 3.433437000 | –1.960600000 |
| H | –5.138388000 | 3.182772000 | –0.246320000 |
| H | –4.318503000 | 2.277106000 | –3.368110000 |
| H | –2.544052000 | 0.997086000 | –5.947480000 |
| H | –4.076700000 | 1.763039000 | –5.508210000 |
| H | –4.218599000 | 0.820873000 | –7.798890000 |
| H | –6.290468000 | 0.828418000 | –6.389320000 |
| H | –6.284683000 | –0.569066000 | –7.480790000 |

| | | | |
|---|---|---|---|
| H | –6.936014000 | –1.256278000 | –5.156080000 |
| H | –5.671861000 | –2.889703000 | –6.588050000 |
| H | –5.261133000 | –3.099047000 | –4.875390000 |
| H | –3.184779000 | –3.085744000 | –6.272280000 |
| H | –4.065864000 | –1.663351000 | –8.149740000 |
| H | –2.536952000 | –1.016394000 | –7.524040000 |
| H | –5.709206000 | 0.514759000 | –3.913090000 |
| H | –5.267338000 | –1.084368000 | –3.299250000 |
| H | –3.054189000 | –2.164798000 | –3.973490000 |
| H | –1.944760000 | –1.312200000 | –5.054040000 |
| H | –1.586590000 | –0.980178000 | –2.891380000 |
| H | –0.055563000 | 1.864116000 | –2.891380000 |
| H | –0.347676000 | 3.727404000 | –3.973490000 |
| H | –0.164019000 | 2.340311000 | –5.054040000 |
| H | –1.079943000 | 4.300971000 | –6.272280000 |
| H | 0.333375000 | 6.356827000 | –6.588050000 |
| H | –0.053287000 | 6.105798000 | –4.875390000 |
| H | 2.380038000 | 6.634903000 | –5.156080000 |
| H | 3.862664000 | 5.033496000 | –6.389320000 |
| H | 2.649516000 | 5.727228000 | –7.480790000 |
| H | 2.820196000 | 3.242978000 | –7.798890000 |
| H | 0.592428000 | 4.352817000 | –8.149740000 |
| H | 0.388253000 | 2.705262000 | –7.524040000 |
| H | 3.300397000 | 4.686937000 | –3.913090000 |
| H | 1.694579000 | 5.103832000 | –3.299250000 |
| H | 2.135528000 | 1.704670000 | –5.947480000 |
| H | 3.565187000 | 2.649006000 | –5.508210000 |
| H | 4.131283000 | 2.601380000 | –3.368110000 |
| H | 5.719687000 | 3.039914000 | –1.960600000 |
| H | 5.325555000 | 2.858589000 | –0.246320000 |
| H | 7.790295000 | 3.094226000 | –0.596440000 |
| H | 8.499743000 | 1.054597000 | 0.692160000 |
| H | 6.976478000 | 1.707911000 | 1.324510000 |
| H | 6.814459000 | –0.775551000 | 1.056430000 |
| H | 6.958619000 | –1.796185000 | –1.224540000 |
| H | 8.488744000 | –1.003671000 | –0.805350000 |
| H | 7.773220000 | –0.400932000 | –3.139570000 |
| H | 9.055428000 | 1.288254000 | –1.796140000 |
| H | 7.912223000 | 2.089252000 | –2.889520000 |
| H | 4.751080000 | 0.564221000 | 0.718430000 |
| H | 4.756592000 | –0.859504000 | –0.325520000 |
| H | 5.708514000 | 0.937242000 | –3.495490000 |
| H | 5.307457000 | –0.639290000 | –2.800060000 |
| H | 2.757943000 | –0.048036000 | –0.289430000 |
| H | 1.642154000 | –0.883939000 | –2.891380000 |
| H | 3.401864000 | –1.562606000 | –3.973490000 |
| H | 2.108778000 | –1.028111000 | –5.054040000 |
| H | 4.264722000 | –1.215227000 | –6.272280000 |
| H | 5.338487000 | –3.467125000 | –6.588050000 |
| H | 5.314420000 | –3.006751000 | –4.875390000 |
| H | 4.555976000 | –5.378625000 | –5.156080000 |
| H | 2.427803000 | –5.861914000 | –6.389320000 |
| H | 3.635167000 | –5.158162000 | –7.480790000 |
| H | 1.398403000 | –4.063850000 | –7.798890000 |
| H | 3.473436000 | –2.689466000 | –8.149740000 |
| H | 2.148699000 | –1.688868000 | –7.524040000 |
| H | 2.408808000 | –5.201697000 | –3.913090000 |
| H | 3.572759000 | –4.019465000 | –3.299250000 |
| H | 0.408524000 | –2.701757000 | –5.947480000 |
| H | 0.511513000 | –4.412045000 | –5.508210000 |
| H | 0.187220000 | –4.878486000 | –3.368110000 |
| H | –1.886910000 | –4.396667000 | 0.718430000 |
| H | –3.122649000 | –3.689578000 | –0.325520000 |
| H | –4.078876000 | –5.513719000 | 1.056430000 |
| H | –5.034851000 | –5.128248000 | –1.224540000 |
| H | –5.113576000 | –6.849632000 | –0.805350000 |
| H | –4.233827000 | –6.531340000 | –3.139570000 |
| H | –3.412053000 | –8.486358000 | –1.796140000 |
| H | –2.146767000 | –7.896812000 | –2.889520000 |
| H | –1.215469000 | –8.293706000 | –0.596440000 |
| H | –3.336564000 | –7.888292000 | 0.692160000 |
| H | –2.009145000 | –6.895762000 | 1.324510000 |
| H | –2.042581000 | –5.412340000 | –3.495490000 |
| H | –3.207370000 | –4.276747000 | –2.800060000 |
| H | –0.227201000 | –6.473351000 | –1.960600000 |
| H | –0.187167000 | –6.041361000 | –0.246320000 |
| H | –1.420572000 | –2.364431000 | –0.289430000 |

B3LYP-D3(BJ)

| | | | |
|---|---|---|---|
| C | 0.000000000 | 0.000000000 | 0.831840000 |
| C | –2.012410000 | –0.204010000 | 2.424790000 |
| C | –3.171790000 | 0.271430000 | 3.054870000 |
| C | –1.354500000 | 0.510290000 | 1.418840000 |
| C | –3.669390000 | 1.517440000 | 2.666510000 |
| C | –1.871980000 | 1.774460000 | 1.085310000 |
| C | –3.015830000 | 2.294660000 | 1.697390000 |
| C | –2.674210000 | 4.413340000 | 0.310730000 |
| C | –3.517500000 | 3.705780000 | 1.392680000 |
| C | –4.989000000 | 3.678460000 | 0.906730000 |
| C | –3.441710000 | 4.563070000 | 2.684220000 |
| C | –3.191190000 | 5.837110000 | 0.048900000 |
| C | –4.656480000 | 5.786290000 | –0.416870000 |
| C | –3.099040000 | 6.658670000 | 1.345520000 |
| C | –5.510960000 | 5.105560000 | 0.664420000 |
| C | –3.957140000 | 5.988060000 | 2.432360000 |
| C | –5.422770000 | 5.918250000 | 1.968020000 |
| C | 7.164382000 | 0.432954000 | 2.432360000 |
| C | 5.672590000 | 0.699073000 | 2.684220000 |
| C | 7.316097000 | –0.645488000 | 1.345520000 |
| C | 7.836740000 | 1.737132000 | 1.968020000 |
| C | 3.148837000 | 2.419065000 | 2.666510000 |
| C | 4.968050000 | 1.193354000 | 1.392680000 |
| C | 1.820960000 | 2.611136000 | 3.054870000 |
| C | 6.650681000 | –0.154903000 | 0.048900000 |
| C | 3.495149000 | 1.464455000 | 1.697390000 |
| C | 5.159170000 | 0.109264000 | 0.310730000 |
| C | 0.829527000 | 1.844803000 | 2.424790000 |
| C | 2.472717000 | 0.733952000 | 1.085310000 |
| C | 1.119174000 | 0.917886000 | 1.418840000 |
| C | 7.177025000 | 2.219851000 | 0.664420000 |
| C | 5.680140000 | 2.481371000 | 0.906730000 |
| C | 7.339314000 | 1.139485000 | –0.416870000 |
| C | –1.666065000 | –7.325411000 | 0.664420000 |
| C | –0.691140000 | –6.159831000 | 0.906730000 |
| C | –2.413970000 | –7.655382000 | 1.968020000 |
| C | –2.682834000 | –6.925775000 | –0.416870000 |
| C | 0.520553000 | –3.936505000 | 2.666510000 |
| C | –1.450550000 | –4.899134000 | 1.392680000 |
| C | 1.350830000 | –2.882566000 | 3.054870000 |
| C | –3.207242000 | –6.421014000 | 2.432360000 |
| C | –0.479319000 | –3.759115000 | 1.697390000 |
| C | –2.230880000 | –5.262143000 | 2.684220000 |
| C | 1.182883000 | –1.640793000 | 2.424790000 |
| C | –0.600737000 | –2.508412000 | 1.085310000 |
| C | 0.235326000 | –1.428176000 | 1.418840000 |
| C | –3.459491000 | –5.682207000 | 0.048900000 |
| C | –2.484960000 | –4.522604000 | 0.310730000 |
| C | –4.217057000 | –6.013182000 | 1.345520000 |
| C | 3.592000000 | –2.768480000 | 6.843470000 |
| C | 3.021150000 | –4.112350000 | 6.362160000 |
| C | 1.901260000 | –3.859900000 | 5.338000000 |
| C | 4.155750000 | –1.998860000 | 5.638340000 |
| C | 3.038130000 | –1.754060000 | 4.611250000 |
| C | 2.441020000 | –3.088130000 | 4.107680000 |
| C | 4.139260000 | –4.940120000 | 5.702640000 |
| C | 5.279430000 | –2.820580000 | 4.982220000 |
| C | 4.712180000 | –4.166990000 | 4.502060000 |
| C | 3.593230000 | –3.913360000 | 3.480340000 |
| C | –5.185684000 | –1.155148000 | 3.480340000 |
| C | –3.894909000 | –0.569920000 | 4.107680000 |
| C | –4.293401000 | 0.283411000 | 5.338000000 |
| C | –5.964809000 | –1.997373000 | 4.502060000 |
| C | –3.038126000 | –1.754068000 | 4.611250000 |
| C | –6.347899000 | –1.114644000 | 5.702640000 |
| C | –5.071975000 | –0.560218000 | 6.362160000 |
| C | –5.082409000 | –3.161830000 | 4.982220000 |
| C | –3.808939000 | –2.599555000 | 5.638340000 |
| C | –4.193574000 | –1.726523000 | 6.843470000 |
| C | –0.000004000 | 3.508128000 | 4.611250000 |
| C | 1.453889000 | 3.658050000 | 4.107680000 |
| C | 1.592454000 | 5.068508000 | 3.480340000 |
| C | –0.346811000 | 4.598415000 | 5.638340000 |
| C | 2.392141000 | 3.576489000 | 5.338000000 |
| C | –0.197021000 | 5.982410000 | 4.982220000 |
| C | 1.252629000 | 6.164363000 | 4.502060000 |
| C | 0.601574000 | 4.495003000 | 6.843470000 |
| C | 2.050825000 | 4.672568000 | 6.362160000 |
| C | 2.208639000 | 6.054764000 | 5.702640000 |
| C | –1.119174000 | –0.917886000 | –1.418840000 |
| C | 0.000000000 | 0.000000000 | –0.831840000 |
| C | –0.235326000 | 1.428176000 | –1.418840000 |
| C | 0.600737000 | 2.508412000 | –1.085310000 |
| C | 0.479319000 | 3.759115000 | –1.697390000 |
| C | 1.450550000 | 4.899134000 | –1.392680000 |
| C | 2.484960000 | 4.522604000 | –0.310730000 |
| C | 3.459491000 | 5.682207000 | –0.048900000 |
| C | 4.217057000 | 6.013182000 | –1.345520000 |
| C | 3.207242000 | 6.421014000 | –2.432360000 |
| C | 2.413970000 | 7.655382000 | –1.968020000 |
| C | 1.666065000 | 7.325411000 | –0.664420000 |
| C | 2.682834000 | 6.925775000 | 0.416870000 |
| C | 2.230880000 | 5.262143000 | –2.684220000 |
| C | 0.691140000 | 6.159831000 | –0.906730000 |
| C | –0.520553000 | 3.936505000 | –2.666510000 |
| C | –1.350830000 | 2.882566000 | –3.054870000 |

| | | | |
|---|---|---|---|
| C | –2.441020000 | 3.088130000 | –4.107680000 |
| C | –1.901260000 | 3.859900000 | –5.338000000 |
| C | –3.021150000 | 4.112350000 | –6.362160000 |
| C | –4.139260000 | 4.940120000 | –5.702640000 |
| C | –4.712180000 | 4.166990000 | –4.502060000 |
| C | –5.279430000 | 2.820580000 | –4.982220000 |
| C | –4.155750000 | 1.998860000 | –5.638340000 |
| C | –3.592000000 | 2.768480000 | –6.843470000 |
| C | –3.593230000 | 3.913360000 | –3.480340000 |
| C | –3.038130000 | 1.754060000 | –4.611250000 |
| C | –1.182883000 | 1.640793000 | –2.424790000 |
| C | 1.354500000 | –0.510290000 | –1.418840000 |
| C | 2.012410000 | 0.204010000 | –2.424790000 |
| C | 3.171790000 | –0.271430000 | –3.054870000 |
| C | 3.894909000 | 0.569920000 | –4.107680000 |
| C | 3.038126000 | 1.754068000 | –4.611250000 |
| C | 3.808939000 | 2.599555000 | –5.638340000 |
| C | 5.082409000 | 3.161830000 | –4.982220000 |
| C | 5.964809000 | 1.997373000 | –4.502060000 |
| C | 6.347899000 | 1.114644000 | –5.702640000 |
| C | 5.071975000 | 0.560218000 | –6.362160000 |
| C | 4.193574000 | 1.726523000 | –6.843470000 |
| C | 5.185684000 | 1.155148000 | –3.480340000 |
| C | 4.293401000 | –0.283411000 | –5.338000000 |
| C | 3.669390000 | –1.517440000 | –2.666510000 |
| C | 3.015830000 | –2.294660000 | –1.697390000 |
| C | 3.517500000 | –3.705780000 | –1.392680000 |
| C | 4.989000000 | –3.678460000 | –0.906730000 |
| C | 5.510960000 | –5.105560000 | –0.664420000 |
| C | 4.656480000 | –5.786290000 | 0.416870000 |
| C | 3.191190000 | –5.837110000 | –0.048900000 |
| C | 3.099040000 | –6.658670000 | –1.345520000 |
| C | 3.957140000 | –5.988060000 | –2.432360000 |
| C | 5.422770000 | –5.918250000 | –1.968020000 |
| C | 2.674210000 | –4.413340000 | –0.310730000 |
| C | 3.441710000 | –4.563070000 | –2.684220000 |
| C | 1.871980000 | –1.774460000 | –1.085310000 |
| C | –0.829527000 | –1.844803000 | –2.424790000 |
| C | –1.820960000 | –2.611136000 | –3.054870000 |
| C | –1.453889000 | –3.658050000 | –4.107680000 |
| C | 0.000004000 | –3.508128000 | –4.611250000 |
| C | 0.346811000 | –4.598415000 | –5.638340000 |
| C | 0.197021000 | –5.982410000 | –4.982220000 |
| C | –1.252629000 | –6.164363000 | –4.502060000 |
| C | –2.208639000 | –6.054764000 | –5.702640000 |
| C | –2.050825000 | –4.672568000 | –6.362160000 |
| C | –0.601574000 | –4.495003000 | –6.843470000 |
| C | –1.592454000 | –5.068508000 | –3.480340000 |
| C | –2.392141000 | –3.576489000 | –5.338000000 |
| C | –3.148837000 | –2.419065000 | –2.666510000 |
| C | –3.495149000 | –1.464455000 | –1.697390000 |
| C | –4.968050000 | –1.193354000 | –1.392680000 |
| C | –5.159170000 | –0.109264000 | –0.310730000 |
| C | –6.650681000 | 0.154903000 | –0.048900000 |
| C | –7.316097000 | 0.645488000 | –1.345520000 |
| C | –7.164382000 | –0.432954000 | –2.432360000 |
| C | –7.836740000 | –1.737132000 | –1.968020000 |
| C | –7.177025000 | –2.219851000 | –0.664420000 |
| C | –7.339314000 | –1.139485000 | 0.416870000 |
| C | –5.672590000 | –0.699073000 | –2.684220000 |
| C | –5.680140000 | –2.481371000 | –0.906730000 |
| C | –2.472717000 | –0.733952000 | –1.085310000 |
| H | –1.608180000 | –1.154020000 | 2.743160000 |
| H | –4.570000000 | 1.907670000 | 3.140410000 |
| H | –1.345040000 | 2.381250000 | 0.363620000 |
| H | –2.688110000 | 3.830050000 | –0.621530000 |
| H | –1.621530000 | 4.461890000 | 0.631770000 |
| H | –5.624010000 | 3.171090000 | 1.650760000 |
| H | –5.052350000 | 3.085380000 | –0.021020000 |
| H | –4.028630000 | 4.088060000 | 3.486640000 |
| H | –2.396540000 | 4.591290000 | 3.031440000 |
| H | –2.567680000 | 6.302230000 | –0.732350000 |
| H | –5.028510000 | 6.807910000 | –0.606960000 |
| H | –4.736930000 | 5.231400000 | –1.363860000 |
| H | –2.048660000 | 6.722660000 | 1.679410000 |
| H | –3.446460000 | 7.691130000 | 1.169240000 |
| H | –6.560660000 | 5.053760000 | 0.330290000 |
| H | –3.890360000 | 6.567690000 | 3.368360000 |
| H | –5.819430000 | 6.934960000 | 1.805790000 |
| H | –6.043690000 | 5.449620000 | 2.751120000 |
| H | 7.632966000 | 0.085306000 | 3.368360000 |
| H | 5.554679000 | 1.444866000 | 3.486640000 |
| H | 5.174444000 | –0.220180000 | 3.031440000 |
| H | 6.846324000 | –1.587138000 | 1.679410000 |
| H | 8.383944000 | –0.860843000 | 1.169240000 |
| H | 8.915567000 | 1.572294000 | 1.805790000 |
| H | 7.741354000 | 2.509179000 | 2.751120000 |
| H | 3.937091000 | 3.003901000 | 3.140410000 |
| H | 6.741731000 | –0.927439000 | –0.732350000 |
| H | 4.660976000 | 0.412947000 | –0.621530000 |
| H | 4.674875000 | –0.826659000 | 0.631770000 |
| H | –0.195321000 | 1.969735000 | 2.743160000 |
| H | 2.734743000 | –0.025786000 | 0.363620000 |
| H | 7.657015000 | 3.154818000 | 0.330290000 |
| H | 5.558249000 | 3.284991000 | 1.650760000 |
| H | 5.198192000 | 2.832773000 | –0.021020000 |
| H | 8.410078000 | 0.950862000 | –0.606960000 |
| H | 6.898990000 | 1.486602000 | –1.363860000 |
| H | –1.096355000 | –8.208578000 | 0.330290000 |
| H | 0.065761000 | –6.456081000 | 1.650760000 |
| H | –0.145842000 | –5.918153000 | –0.021020000 |
| H | –3.096137000 | –8.507254000 | 1.805790000 |
| H | –1.697664000 | –7.958799000 | 2.751120000 |
| H | –3.381568000 | –7.758772000 | –0.606960000 |
| H | –2.162060000 | –6.718002000 | –1.363860000 |
| H | 0.632909000 | –4.911571000 | 3.140410000 |
| H | –3.742606000 | –6.652996000 | 3.368360000 |
| H | –1.526049000 | –5.532926000 | 3.486640000 |
| H | –2.777904000 | –4.371110000 | 3.031440000 |
| H | 1.803501000 | –0.815715000 | 2.743160000 |
| H | –1.389703000 | –2.355464000 | 0.363620000 |
| H | –4.174051000 | –5.374791000 | –0.732350000 |
| H | –1.972866000 | –4.242997000 | –0.621530000 |
| H | –3.053345000 | –3.635231000 | 0.631770000 |
| H | –4.797664000 | –5.135522000 | 1.679410000 |
| H | –4.937484000 | –6.830287000 | 1.169240000 |
| H | 4.383510000 | –2.936830000 | 7.593850000 |
| H | 2.800790000 | –2.176090000 | 7.334300000 |
| H | 2.606640000 | –4.668260000 | 7.219580000 |
| H | 1.081110000 | –3.281780000 | 5.796350000 |
| H | 1.475210000 | –4.825770000 | 5.025140000 |
| H | 4.554840000 | –1.025770000 | 5.969210000 |
| H | 3.450620000 | –1.184120000 | 3.765890000 |
| H | 2.241770000 | –1.132730000 | 5.053980000 |
| H | 3.742170000 | –5.914730000 | 5.369720000 |
| H | 4.937730000 | –5.149260000 | 6.434840000 |
| H | 6.098410000 | –2.987860000 | 5.702590000 |
| H | 5.707550000 | –2.265050000 | 4.130040000 |
| H | 5.510740000 | –4.759240000 | 4.024420000 |
| H | 3.196310000 | –4.869820000 | 3.102970000 |
| H | 3.989290000 | –3.368010000 | 2.607510000 |
| H | –5.815543000 | –0.333176000 | 3.102970000 |
| H | –4.911427000 | –1.770821000 | 2.607510000 |
| H | –3.382660000 | 0.704621000 | 5.796350000 |
| H | –4.916844000 | 1.135316000 | 5.025140000 |
| H | –6.876993000 | –2.392821000 | 4.024420000 |
| H | –2.750788000 | –2.396265000 | 3.765890000 |
| H | –2.101858000 | –1.375065000 | 5.053980000 |
| H | –6.993391000 | –0.283449000 | 5.369720000 |
| H | –6.928255000 | –1.701570000 | 6.434840000 |
| H | –5.346152000 | 0.076714000 | 7.219580000 |
| H | –5.636768000 | –3.787448000 | 5.702590000 |
| H | –4.815366000 | –3.810358000 | 4.130040000 |
| H | –3.165763000 | –3.431722000 | 5.969210000 |
| H | –4.735124000 | –2.327816000 | 7.593850000 |
| H | –3.284944000 | –1.337510000 | 7.334300000 |
| H | –0.699832000 | 3.580385000 | 3.765890000 |
| H | –0.139912000 | 2.507795000 | 5.053980000 |
| H | 2.619233000 | 5.202996000 | 3.102970000 |
| H | 0.922137000 | 5.138831000 | 2.607510000 |
| H | –1.389077000 | 4.457492000 | 5.969210000 |
| H | 2.301550000 | 2.577159000 | 5.796350000 |
| H | 3.441634000 | 3.690454000 | 5.025140000 |
| H | –0.461642000 | 6.775308000 | 5.702590000 |
| H | –0.892184000 | 6.075408000 | 4.130040000 |
| H | 1.366253000 | 7.152061000 | 4.024420000 |
| H | 0.351614000 | 5.264646000 | 7.593850000 |
| H | 0.484154000 | 3.513600000 | 7.334300000 |
| H | 2.739512000 | 4.591546000 | 7.219580000 |
| H | 3.251221000 | 6.198179000 | 5.369720000 |
| H | 1.990525000 | 6.850830000 | 6.434840000 |
| H | 1.389703000 | 2.355464000 | –0.363620000 |
| H | 1.972866000 | 4.242997000 | 0.621530000 |
| H | 3.053345000 | 3.635231000 | –0.631770000 |
| H | 4.174051000 | 5.374791000 | 0.732350000 |
| H | 4.797664000 | 5.135522000 | –1.679410000 |
| H | 4.937484000 | 6.830287000 | –1.169240000 |
| H | 3.742606000 | 6.652996000 | –3.368360000 |
| H | 3.096137000 | 8.507254000 | –1.805790000 |
| H | 1.697664000 | 7.958799000 | –2.751120000 |
| H | 1.096355000 | 8.208578000 | –0.330290000 |
| H | 3.381568000 | 7.758772000 | 0.606960000 |
| H | 2.162060000 | 6.718002000 | 1.363860000 |
| H | 1.526049000 | 5.532926000 | –3.486640000 |
| H | 2.777904000 | 4.371110000 | –3.031440000 |
| H | –0.065761000 | 6.456081000 | –1.650760000 |

| | | | |
|---|---|---|---|
| H | 0.145842000 | 5.918153000 | 0.021020000 |
| H | –0.632909000 | 4.911571000 | –3.140410000 |
| H | –1.081110000 | 3.281780000 | –5.796350000 |
| H | –1.475210000 | 4.825770000 | –5.025140000 |
| H | –2.606640000 | 4.668260000 | –7.219580000 |
| H | –3.742170000 | 5.914730000 | –5.369720000 |
| H | –4.937730000 | 5.149260000 | –6.434840000 |
| H | –5.510740000 | 4.759240000 | –4.024420000 |
| H | –6.098410000 | 2.987860000 | –5.702590000 |
| H | –5.707550000 | 2.265050000 | –4.130040000 |
| H | –4.554840000 | 1.025770000 | –5.969210000 |
| H | –4.383510000 | 2.936830000 | –7.593850000 |
| H | –2.800790000 | 2.176090000 | –7.334300000 |
| H | –3.196310000 | 4.869820000 | –3.102970000 |
| H | –3.989290000 | 3.368010000 | –2.607510000 |
| H | –3.450620000 | 1.184120000 | –3.765890000 |
| H | –2.241770000 | 1.132730000 | –5.053980000 |
| H | –1.803501000 | 0.815715000 | –2.743160000 |
| H | 1.608180000 | 1.154020000 | –2.743160000 |
| H | 2.750788000 | 2.396265000 | –3.765890000 |
| H | 2.101858000 | 1.375065000 | –5.053980000 |
| H | 3.165763000 | 3.431722000 | –5.969210000 |
| H | 5.636768000 | 3.787448000 | –5.702590000 |
| H | 4.815366000 | 3.810358000 | –4.130040000 |
| H | 6.876993000 | 2.392821000 | –4.024420000 |
| H | 6.993391000 | 0.283449000 | –5.369720000 |
| H | 6.928255000 | 1.701570000 | –6.434840000 |
| H | 5.346152000 | –0.076714000 | –7.219580000 |
| H | 4.735124000 | 2.327816000 | –7.593850000 |
| H | 3.284944000 | 1.337510000 | –7.334300000 |
| H | 5.815543000 | 0.333176000 | –3.102970000 |
| H | 4.911427000 | 1.770821000 | –2.607510000 |
| H | 3.382660000 | –0.704621000 | –5.796350000 |
| H | 4.916844000 | –1.135316000 | –5.025140000 |
| H | 4.570000000 | –1.907670000 | –3.140410000 |
| H | 5.624010000 | –3.171090000 | –1.650760000 |
| H | 5.052350000 | –3.085380000 | 0.021020000 |
| H | 6.560660000 | –5.053760000 | –0.330290000 |
| H | 5.028510000 | –6.807910000 | 0.606960000 |
| H | 4.736930000 | –5.231400000 | 1.363860000 |
| H | 2.567680000 | –6.302230000 | 0.732350000 |
| H | 2.048660000 | –6.722660000 | –1.679410000 |
| H | 3.446460000 | –7.691130000 | –1.169240000 |
| H | 3.890360000 | –6.567690000 | –3.368360000 |
| H | 5.819430000 | –6.934960000 | –1.805790000 |
| H | 6.043690000 | –5.449620000 | –2.751120000 |
| H | 2.688110000 | –3.830050000 | 0.621530000 |
| H | 1.621530000 | –4.461890000 | –0.631770000 |
| H | 4.028630000 | –4.088060000 | –3.486640000 |
| H | 2.396540000 | –4.591290000 | –3.031440000 |
| H | 1.345040000 | –2.381250000 | –0.363620000 |
| H | 0.195321000 | –1.969735000 | –2.743160000 |
| H | 0.699832000 | –3.580385000 | –3.765890000 |
| H | 0.139912000 | –2.507795000 | –5.053980000 |
| H | 1.389077000 | –4.457492000 | –5.969210000 |
| H | 0.461642000 | –6.775308000 | –5.702590000 |
| H | 0.892184000 | –6.075408000 | –4.130040000 |
| H | –1.366253000 | –7.152061000 | –4.024420000 |
| H | –3.251221000 | –6.198179000 | –5.369720000 |
| H | –1.990525000 | –6.850830000 | –6.434840000 |
| H | –2.739512000 | –4.591546000 | –7.219580000 |
| H | –0.351614000 | –5.264646000 | –7.593850000 |
| H | –0.484154000 | –3.513600000 | –7.334300000 |
| H | –2.619233000 | –5.202996000 | –3.102970000 |
| H | –0.922137000 | –5.138831000 | –2.607510000 |
| H | –2.301550000 | –2.577159000 | –5.796350000 |
| H | –3.441634000 | –3.690454000 | –5.025140000 |
| H | –3.937091000 | –3.003901000 | –3.140410000 |
| H | –4.660976000 | –0.412947000 | 0.621530000 |
| H | –4.674875000 | 0.826659000 | –0.631770000 |
| H | –6.741731000 | 0.927439000 | 0.732350000 |
| H | –6.846324000 | 1.587138000 | –1.679410000 |
| H | –8.383944000 | 0.860843000 | –1.169240000 |
| H | –7.632966000 | –0.085306000 | –3.368360000 |
| H | –8.915567000 | –1.572294000 | –1.805790000 |
| H | –7.741354000 | –2.509179000 | –2.751120000 |
| H | –7.657015000 | –3.154818000 | –0.330290000 |
| H | –8.410078000 | –0.950862000 | 0.606960000 |
| H | –6.898990000 | –1.486602000 | 1.363860000 |
| H | –5.554679000 | –1.444866000 | –3.486640000 |
| H | –5.174444000 | 0.220180000 | –3.031440000 |
| H | –5.558249000 | –3.284991000 | –1.650760000 |
| H | –5.198192000 | –2.832773000 | 0.021020000 |
| H | –2.734743000 | 0.025786000 | –0.363620000 |

M06-2X

| | | | |
|---|---|---|---|
| C | 0.000000000 | 0.000000000 | 0.830200000 |
| C | –1.625780000 | –1.183720000 | 2.442080000 |
| C | –2.863480000 | –1.361270000 | 3.075610000 |
| C | –1.425800000 | –0.245830000 | 1.425810000 |
| C | –3.928600000 | –0.552020000 | 2.683060000 |
| C | –2.518670000 | 0.571590000 | 1.090010000 |
| C | –3.766010000 | 0.438420000 | 1.704070000 |
| C | –4.567690000 | 2.392630000 | 0.279010000 |
| C | –4.929350000 | 1.371670000 | 1.372830000 |
| C | –6.151820000 | 0.562330000 | 0.883970000 |
| C | –5.341050000 | 2.157540000 | 2.640440000 |
| C | –5.751990000 | 3.320910000 | –0.020910000 |
| C | –6.958140000 | 2.496080000 | –0.491200000 |
| C | –6.130780000 | 4.088140000 | 1.252880000 |
| C | –7.342380000 | 1.489310000 | 0.599640000 |
| C | –6.525040000 | 3.088280000 | 2.348800000 |
| C | –7.720720000 | 2.248360000 | 1.878470000 |
| C | 5.937049000 | 4.106710000 | 2.348800000 |
| C | 4.539009000 | 3.546715000 | 2.640440000 |
| C | 6.605823000 | 3.265341000 | 1.252880000 |
| C | 5.807497000 | 5.562160000 | 1.878470000 |
| C | 1.486237000 | 3.678277000 | 2.683060000 |
| C | 3.652576000 | 3.583107000 | 1.372830000 |
| C | 0.252846000 | 3.160481000 | 3.075610000 |
| C | 5.751987000 | 3.320914000 | –0.020910000 |
| C | 2.262688000 | 3.042250000 | 1.704070000 |
| C | 4.355923000 | 2.759421000 | 0.279010000 |
| C | –0.212242000 | 1.999827000 | 2.442080000 |
| C | 1.754346000 | 1.895437000 | 1.090010000 |
| C | 0.500005000 | 1.357694000 | 1.425810000 |
| C | 4.960970000 | 5.614033000 | 0.599640000 |
| C | 3.562902000 | 5.046467000 | 0.883970000 |
| C | 5.640739000 | 4.777886000 | –0.491200000 |
| C | 2.381410000 | –7.103343000 | 0.599640000 |
| C | 2.588918000 | –5.608797000 | 0.883970000 |
| C | 1.913223000 | –7.810520000 | 1.878470000 |
| C | 1.317401000 | –7.273966000 | –0.491200000 |
| C | 2.442363000 | –3.126257000 | 2.683060000 |
| C | 1.276774000 | –4.954777000 | 1.372830000 |
| C | 2.610634000 | –1.799211000 | 3.075610000 |
| C | 0.587991000 | –7.194990000 | 2.348800000 |
| C | 1.503322000 | –3.480670000 | 1.704070000 |
| C | 0.802041000 | –5.704255000 | 2.640440000 |
| C | 1.838022000 | –0.816107000 | 2.442080000 |
| C | 0.764324000 | –2.467027000 | 1.090010000 |
| C | 0.925795000 | –1.111864000 | 1.425810000 |
| C | 0.000003000 | –6.641824000 | –0.020910000 |
| C | 0.211767000 | –5.152051000 | 0.279010000 |
| C | –0.475043000 | –7.353481000 | 1.252880000 |
| C | 4.457590000 | –0.498530000 | 6.844150000 |
| C | 4.605050000 | –1.964350000 | 6.418230000 |
| C | 3.519690000 | –2.313580000 | 5.389630000 |
| C | 4.604140000 | 0.402690000 | 5.612130000 |
| C | 3.523850000 | 0.048470000 | 4.582080000 |
| C | 3.645280000 | –1.421450000 | 4.134200000 |
| C | 5.989230000 | –2.178680000 | 5.788850000 |
| C | 5.991410000 | 0.195320000 | 4.988500000 |
| C | 6.142620000 | –1.270360000 | 4.561160000 |
| C | 5.055960000 | –1.610000000 | 3.533890000 |
| C | –3.922281000 | –3.573590000 | 3.533890000 |
| C | –3.053652000 | –2.446180000 | 4.134200000 |
| C | –3.763464000 | –1.891351000 | 5.389630000 |
| C | –4.171474000 | –4.684485000 | 4.561160000 |
| C | –1.719949000 | –3.075979000 | 4.582080000 |
| C | –4.881407000 | –4.097485000 | 5.788850000 |
| C | –4.003702000 | –3.005915000 | 6.418230000 |
| C | –2.826553000 | –5.286373000 | 4.988500000 |
| C | –1.953330000 | –4.188647000 | 5.612130000 |
| C | –2.660535000 | –3.611121000 | 6.844150000 |
| C | –1.803901000 | 3.027509000 | 4.582080000 |
| C | –0.591628000 | 3.867630000 | 4.134200000 |
| C | –1.133679000 | 5.183590000 | 3.533890000 |
| C | –2.650810000 | 3.785957000 | 5.612130000 |
| C | 0.243774000 | 4.204931000 | 5.389630000 |
| C | –3.164857000 | 5.091053000 | 4.988500000 |
| C | –1.971146000 | 5.954845000 | 4.561160000 |
| C | –1.797055000 | 4.109651000 | 6.844150000 |
| C | –0.601348000 | 4.970265000 | 6.418230000 |
| C | –1.107823000 | 6.276165000 | 5.788850000 |
| C | –0.500005000 | –1.357694000 | –1.425810000 |
| C | 0.000000000 | 0.000000000 | –0.830200000 |
| C | –0.925795000 | 1.111864000 | –1.425810000 |
| C | –0.764324000 | 2.467027000 | –1.090010000 |
| C | –1.503322000 | 3.480670000 | –1.704070000 |
| C | –1.276774000 | 4.954777000 | –1.372830000 |
| C | –0.211767000 | 5.152051000 | –0.279010000 |

| | | | | | | | |
|---|---|---|---|---|---|---|---|
| C | –0.000003000 | 6.641824000 | 0.020910000 | H | –8.589500000 | 2.899550000 | 1.686490000 |
| C | 0.475043000 | 7.353481000 | –1.252880000 | H | –8.013480000 | 1.537170000 | 2.669140000 |
| C | –0.587991000 | 7.194990000 | –2.348800000 | H | 6.542008000 | 4.066770000 | 3.268410000 |
| C | –1.913223000 | 7.810520000 | –1.878470000 | H | 4.063469000 | 4.127714000 | 3.447920000 |
| C | –2.381410000 | 7.103343000 | –0.599640000 | H | 4.614205000 | 2.504797000 | 2.995000000 |
| C | –1.317401000 | 7.273966000 | 0.491200000 | H | 6.710791000 | 2.220171000 | 1.591890000 |
| C | –0.802041000 | 5.704255000 | –2.640440000 | H | 7.619840000 | 3.647330000 | 1.047990000 |
| C | –2.588918000 | 5.608797000 | –0.883970000 | H | 6.805834000 | 5.988950000 | 1.686490000 |
| C | –2.442363000 | 3.126257000 | –2.683060000 | H | 5.337968000 | 6.171292000 | 2.669140000 |
| C | –2.610634000 | 1.799211000 | –3.075610000 | H | 1.866489000 | 4.585414000 | 3.157820000 |
| C | –3.645280000 | 1.421450000 | –4.134200000 | H | 6.218609000 | 2.708726000 | –0.810040000 |
| C | –3.519690000 | 2.313580000 | –5.389630000 | H | 3.743273000 | 2.750859000 | –0.638650000 |
| C | –4.605050000 | 1.964350000 | –6.418230000 | H | 4.444919000 | 1.709085000 | 0.608410000 |
| C | –5.989230000 | 2.178680000 | –5.788850000 | H | –1.151018000 | 1.579779000 | 2.777650000 |
| C | –6.142620000 | 1.270360000 | –4.561160000 | H | 2.369977000 | 1.385400000 | 0.360740000 |
| C | –5.991410000 | –0.195320000 | –4.988500000 | H | 4.864644000 | 6.657551000 | 0.260920000 |
| C | –4.604140000 | –0.402690000 | –5.612130000 | H | 3.058225000 | 5.666701000 | 1.643420000 |
| C | –4.457590000 | 0.498530000 | –6.844150000 | H | 2.943205000 | 5.084421000 | –0.028850000 |
| C | –5.055960000 | 1.610000000 | –3.533890000 | H | 6.644092000 | 5.182185000 | –0.706750000 |
| C | –3.523850000 | –0.048470000 | –4.582080000 | H | 5.058422000 | 4.832363000 | –1.424000000 |
| C | –1.838022000 | 0.816107000 | –2.442080000 | H | 3.333286000 | –7.541681000 | 0.260920000 |
| C | 1.425800000 | 0.245830000 | –1.425810000 | H | 3.378395000 | –5.481851000 | 1.643420000 |
| C | 1.625780000 | 1.183720000 | –2.442080000 | H | 2.931635000 | –5.091101000 | –0.028850000 |
| C | 2.863480000 | 1.361270000 | –3.075610000 | H | 1.783666000 | –8.888500000 | 1.686490000 |
| C | 3.053652000 | 2.446180000 | –4.134200000 | H | 2.675512000 | –7.708462000 | 2.669140000 |
| C | 1.719949000 | 3.075979000 | –4.582080000 | H | 1.165858000 | –8.345045000 | –0.706750000 |
| C | 1.953330000 | 4.188647000 | –5.612130000 | H | 1.655738000 | –6.796903000 | –1.424000000 |
| C | 2.826553000 | 5.286373000 | –4.988500000 | H | 3.037841000 | –3.909134000 | 3.157820000 |
| C | 4.171474000 | 4.684485000 | –4.561160000 | H | 0.250922000 | –7.698930000 | 3.268410000 |
| C | 4.881407000 | 4.097485000 | –5.788850000 | H | 1.542971000 | –5.582924000 | 3.447920000 |
| C | 4.003702000 | 3.005915000 | –6.418230000 | H | –0.137885000 | –5.248417000 | 2.995000000 |
| C | 2.660535000 | 3.611121000 | –6.844150000 | H | 1.943638000 | 0.206921000 | 2.777650000 |
| C | 3.922281000 | 3.573590000 | –3.533890000 | H | 0.014803000 | –2.745160000 | 0.360740000 |
| C | 3.763464000 | 1.891351000 | –5.389630000 | H | –0.763479000 | –6.739836000 | –0.810040000 |
| C | 3.928600000 | 0.552020000 | –2.683060000 | H | 0.510677000 | –4.617199000 | –0.638650000 |
| C | 3.766010000 | –0.438420000 | –1.704070000 | H | –0.742349000 | –4.703955000 | 0.608410000 |
| C | 4.929350000 | –1.371670000 | –1.372830000 | H | –1.432671000 | –6.921801000 | 1.591890000 |
| C | 6.151820000 | –0.562330000 | –0.883970000 | H | –0.651240000 | –8.422640000 | 1.047990000 |
| C | 7.342380000 | –1.489310000 | –0.599640000 | H | 5.223490000 | –0.243140000 | 7.595280000 |
| C | 6.958140000 | –2.496080000 | 0.491200000 | H | 3.472310000 | –0.339520000 | 7.313000000 |
| C | 5.751990000 | –3.320910000 | 0.020910000 | H | 4.494180000 | –2.619310000 | 7.296420000 |
| C | 6.130780000 | –4.088140000 | –1.252880000 | H | 2.516000000 | –2.179970000 | 5.827720000 |
| C | 6.525040000 | –3.088280000 | –2.348800000 | H | 3.612350000 | –3.375550000 | 5.112330000 |
| C | 7.720720000 | –2.248360000 | –1.878470000 | H | 4.485100000 | 1.457730000 | 5.904690000 |
| C | 4.567690000 | –2.392630000 | –0.279010000 | H | 3.629150000 | 0.713730000 | 3.710110000 |
| C | 5.341050000 | –2.157540000 | –2.640440000 | H | 2.519620000 | 0.229090000 | 5.002780000 |
| C | 2.518670000 | –0.571590000 | –1.090010000 | H | 6.111030000 | –3.234760000 | 5.495640000 |
| C | 0.212242000 | –1.999827000 | –2.442080000 | H | 6.777640000 | –1.951410000 | 6.525450000 |
| C | –0.252846000 | –3.160481000 | –3.075610000 | H | 6.777020000 | 0.461420000 | 5.715030000 |
| C | 0.591628000 | –3.867630000 | –4.134200000 | H | 6.115540000 | 0.857620000 | 4.114410000 |
| C | 1.803901000 | –3.027509000 | –4.582080000 | H | 7.133930000 | –1.425290000 | 4.105930000 |
| C | 2.650810000 | –3.785957000 | –5.612130000 | H | 5.172030000 | –2.649230000 | 3.178860000 |
| C | 3.164857000 | –5.091053000 | –4.988500000 | H | 5.150010000 | –0.953820000 | 2.650400000 |
| C | 1.971146000 | –5.954845000 | –4.561160000 | H | –4.880315000 | –3.154494000 | 3.178860000 |
| C | 1.107823000 | –6.276165000 | –5.788850000 | H | –3.401037000 | –3.983129000 | 2.650400000 |
| C | 0.601348000 | –4.970265000 | –6.418230000 | H | –3.145909000 | –1.088935000 | 5.827720000 |
| C | 1.797055000 | –4.109651000 | –6.844150000 | H | –4.729487000 | –1.440612000 | 5.112330000 |
| C | 1.133679000 | –5.183590000 | –3.533890000 | H | –4.801302000 | –5.465520000 | 4.105930000 |
| C | –0.243774000 | –4.204931000 | –5.389630000 | H | –1.196467000 | –3.499801000 | 3.710110000 |
| C | –1.486237000 | –3.678277000 | –2.683060000 | H | –1.061412000 | –2.296600000 | 5.002780000 |
| C | –2.262688000 | –3.042250000 | –1.704070000 | H | –5.856899000 | –3.674927000 | 5.495640000 |
| C | –3.652576000 | –3.583107000 | –1.372830000 | H | –5.078791000 | –4.893903000 | 6.525450000 |
| C | –4.355923000 | –2.759421000 | –0.279010000 | H | –4.515479000 | –2.582419000 | 7.296420000 |
| C | –5.751987000 | –3.320914000 | 0.020910000 | H | –2.988909000 | –6.099781000 | 5.715030000 |
| C | –6.605823000 | –3.265341000 | –1.252880000 | H | –2.315049000 | –5.725023000 | 4.114410000 |
| C | –5.937049000 | –4.106710000 | –2.348800000 | H | –0.980119000 | –4.613076000 | 5.904690000 |
| C | –5.807497000 | –5.562160000 | –1.878470000 | H | –2.822310000 | –4.402105000 | 7.595280000 |
| C | –4.960970000 | –5.614033000 | –0.599640000 | H | –2.030188000 | –2.837349000 | 7.313000000 |
| C | –5.640739000 | –4.777886000 | 0.491200000 | H | –2.432683000 | 2.786071000 | 3.710110000 |
| C | –4.539009000 | –3.546715000 | –2.640440000 | H | –1.458208000 | 2.067510000 | 5.002780000 |
| C | –3.562902000 | –5.046467000 | –0.883970000 | H | –0.291715000 | 5.803724000 | 3.178860000 |
| C | –1.754346000 | –1.895437000 | –1.090010000 | H | –1.748973000 | 4.936949000 | 2.650400000 |
| H | –0.792620000 | –1.786700000 | 2.777650000 | H | –3.504981000 | 3.155346000 | 5.904690000 |
| H | –4.904330000 | –0.676280000 | 3.157820000 | H | 0.629909000 | 3.268905000 | 5.827720000 |
| H | –2.384780000 | 1.359760000 | 0.360740000 | H | 1.117137000 | 4.816162000 | 5.112330000 |
| H | –4.253950000 | 1.866340000 | –0.638650000 | H | –3.788111000 | 5.638361000 | 5.715030000 |
| H | –3.702570000 | 2.994870000 | 0.608410000 | H | –3.800491000 | 4.867403000 | 4.114410000 |
| H | –6.436620000 | –0.184850000 | 1.643420000 | H | –2.332628000 | 6.890810000 | 4.105930000 |
| H | –5.874840000 | 0.006680000 | –0.028850000 | H | –2.401180000 | 4.645245000 | 7.595280000 |
| H | –5.606440000 | 1.455210000 | 3.447920000 | H | –1.442122000 | 3.176869000 | 7.313000000 |
| H | –4.476320000 | 2.743620000 | 2.995000000 | H | 0.021299000 | 5.201729000 | 7.296420000 |
| H | –5.455130000 | 4.031110000 | –0.810040000 | H | –0.254131000 | 6.909687000 | 5.495640000 |
| H | –7.809950000 | 3.162860000 | –0.706750000 | H | –1.698849000 | 6.845313000 | 6.525450000 |
| H | –6.714160000 | 1.964540000 | –1.424000000 | H | –0.014803000 | 2.745160000 | –0.360740000 |
| H | –5.278120000 | 4.701630000 | 1.591890000 | H | –0.510677000 | 4.617199000 | 0.638650000 |
| H | –6.968600000 | 4.775310000 | 1.047990000 | H | 0.742349000 | 4.703955000 | –0.608410000 |
| H | –8.197930000 | 0.884130000 | 0.260920000 | H | 0.763479000 | 6.739836000 | 0.810040000 |
| H | –6.792930000 | 3.632160000 | 3.268410000 | H | 1.432671000 | 6.921801000 | –1.591890000 |

| | | | |
|---|---|---|---|
| H | 0.651240000 | 8.422640000 | –1.047990000 |
| H | –0.250922000 | 7.698930000 | –3.268410000 |
| H | –1.783666000 | 8.888500000 | –1.686490000 |
| H | –2.675512000 | 7.708462000 | –2.669140000 |
| H | –3.333286000 | 7.541681000 | –0.260920000 |
| H | –1.165858000 | 8.345045000 | 0.706750000 |
| H | –1.655738000 | 6.796903000 | 1.424000000 |
| H | –1.542971000 | 5.582924000 | –3.447920000 |
| H | 0.137885000 | 5.248417000 | –2.995000000 |
| H | –3.378395000 | 5.481851000 | –1.643420000 |
| H | –2.931635000 | 5.091101000 | 0.028850000 |
| H | –3.037841000 | 3.909134000 | –3.157820000 |
| H | –2.516000000 | 2.179970000 | –5.827720000 |
| H | –3.612350000 | 3.375550000 | –5.112330000 |
| H | –4.494180000 | 2.619310000 | –7.296420000 |
| H | –6.111030000 | 3.234760000 | –5.495640000 |
| H | –6.777640000 | 1.951410000 | –6.525450000 |
| H | –7.133930000 | 1.425290000 | –4.105930000 |
| H | –6.777020000 | –0.461420000 | –5.715030000 |
| H | –6.115540000 | –0.857620000 | –4.114410000 |
| H | –4.485100000 | –1.457730000 | –5.904690000 |
| H | –5.223490000 | 0.243140000 | –7.595280000 |
| H | –3.472310000 | 0.339520000 | –7.313000000 |
| H | –5.172030000 | 2.649230000 | –3.178860000 |
| H | –5.150010000 | 0.953820000 | –2.650400000 |
| H | –3.629150000 | –0.713730000 | –3.710110000 |
| H | –2.519620000 | –0.229090000 | –5.002780000 |
| H | –1.943638000 | –0.206921000 | –2.777650000 |
| H | 0.792620000 | 1.786700000 | –2.777650000 |
| H | 1.196467000 | 3.499801000 | –3.710110000 |
| H | 1.061412000 | 2.296600000 | –5.002780000 |
| H | 0.980119000 | 4.613076000 | –5.904690000 |
| H | 2.988909000 | 6.099781000 | –5.715030000 |
| H | 2.315049000 | 5.725023000 | –4.114410000 |
| H | 4.801302000 | 5.465520000 | –4.105930000 |
| H | 5.856899000 | 3.674927000 | –5.495640000 |
| H | 5.078791000 | 4.893903000 | –6.525450000 |
| H | 4.515479000 | 2.582419000 | –7.296420000 |
| H | 2.822310000 | 4.402105000 | –7.595280000 |
| H | 2.030188000 | 2.837349000 | –7.313000000 |
| H | 4.880315000 | 3.154494000 | –3.178860000 |
| H | 3.401037000 | 3.983129000 | –2.650400000 |
| H | 3.145909000 | 1.088935000 | –5.827720000 |
| H | 4.729487000 | 1.440612000 | –5.112330000 |
| H | 4.904330000 | 0.676280000 | –3.157820000 |
| H | 6.436620000 | 0.184850000 | –1.643420000 |
| H | 5.874840000 | –0.006680000 | 0.028850000 |

| | | | |
|---|---|---|---|
| H | 8.197930000 | –0.884130000 | –0.260920000 |
| H | 7.809950000 | –3.162860000 | 0.706750000 |
| H | 6.714160000 | –1.964540000 | 1.424000000 |
| H | 5.455130000 | –4.031110000 | 0.810040000 |
| H | 5.278120000 | –4.701630000 | –1.591890000 |
| H | 6.968600000 | –4.775310000 | –1.047990000 |
| H | 6.792930000 | –3.632160000 | –3.268410000 |
| H | 8.589500000 | –2.899550000 | –1.686490000 |
| H | 8.013480000 | –1.537170000 | –2.669140000 |
| H | 4.253950000 | –1.866340000 | 0.638650000 |
| H | 3.702570000 | –2.994870000 | –0.608410000 |
| H | 5.606440000 | –1.455210000 | –3.447920000 |
| H | 4.476320000 | –2.743620000 | –2.995000000 |
| H | 2.384780000 | –1.359760000 | –0.360740000 |
| H | 1.151018000 | –1.579779000 | –2.777650000 |
| H | 2.432683000 | –2.786071000 | –3.710110000 |
| H | 1.458208000 | –2.067510000 | –5.002780000 |
| H | 3.504981000 | –3.155346000 | –5.904690000 |
| H | 3.788111000 | –5.638361000 | –5.715030000 |
| H | 3.800491000 | –4.867403000 | –4.114410000 |
| H | 2.332628000 | –6.890810000 | –4.105930000 |
| H | 0.254131000 | –6.909687000 | –5.495640000 |
| H | 1.698849000 | –6.845313000 | –6.525450000 |
| H | –0.021299000 | –5.201729000 | –7.296420000 |
| H | 2.401180000 | –4.645245000 | –7.595280000 |
| H | 1.442122000 | –3.176869000 | –7.313000000 |
| H | 0.291715000 | –5.803724000 | –3.178860000 |
| H | 1.748973000 | –4.936949000 | –2.650400000 |
| H | –0.629909000 | –3.268905000 | –5.827720000 |
| H | –1.117137000 | –4.816162000 | –5.112330000 |
| H | –1.866489000 | –4.585414000 | –3.157820000 |
| H | –3.743273000 | –2.750859000 | 0.638650000 |
| H | –4.444919000 | –1.709085000 | –0.608410000 |
| H | –6.218609000 | –2.708726000 | 0.810040000 |
| H | –6.710791000 | –2.220171000 | –1.591890000 |
| H | –7.619840000 | –3.647330000 | –1.047990000 |
| H | –6.542008000 | –4.066770000 | –3.268410000 |
| H | –6.805834000 | –5.988950000 | –1.686490000 |
| H | –5.337968000 | –6.171292000 | –2.669140000 |
| H | –4.864644000 | –6.657551000 | –0.260920000 |
| H | –6.644092000 | –5.182185000 | 0.706750000 |
| H | –5.058422000 | –4.832363000 | 1.424000000 |
| H | –4.063469000 | –4.127714000 | –3.447920000 |
| H | –4.614205000 | –2.504797000 | –2.995000000 |
| H | –3.058225000 | –5.666701000 | –1.643420000 |
| H | –2.943205000 | –5.084421000 | 0.028850000 |
| H | –2.369977000 | –1.385400000 | –0.360740000 |

**Table S17.** Dimers R-$\mathbf{1}_2$ with C–PCM:cyclohexane.

H-$\mathbf{1}_2$

B3LYP

| | | | |
|---|---|---|---|
| C | –2.806325000 | 2.988527000 | 2.541520000 |
| C | –1.826088000 | 3.366606000 | 1.619210000 |
| C | –0.943037000 | 2.423275000 | 1.090900000 |
| C | –1.012283000 | 1.055308000 | 1.436980000 |
| C | –1.979721000 | 0.710098000 | 2.398610000 |
| C | –2.864115000 | 1.653593000 | 2.936950000 |
| C | 1.420065000 | 0.349008000 | 1.436980000 |
| C | 2.570136000 | –0.394943000 | 1.090900000 |
| C | 3.828610000 | –0.101865000 | 1.619210000 |
| C | 3.991303000 | 0.936085000 | 2.541520000 |
| C | 2.864111000 | 1.653600000 | 2.936950000 |
| C | 1.604824000 | 1.359439000 | 2.398610000 |
| C | 0.000004000 | –3.307193000 | 2.936950000 |
| C | 0.374897000 | –2.069538000 | 2.398610000 |
| C | –0.407782000 | –1.404316000 | 1.436980000 |
| C | –1.627099000 | –2.028332000 | 1.090900000 |
| C | –2.002522000 | –3.264741000 | 1.619210000 |
| C | –1.184978000 | –3.924612000 | 2.541520000 |
| C | 0.000000000 | 0.000000000 | 0.865340000 |
| C | 2.806325000 | –2.988527000 | –2.541520000 |
| C | 2.864115000 | –1.653593000 | –2.936950000 |
| C | 1.979721000 | –0.710098000 | –2.398610000 |
| C | 1.012283000 | –1.055308000 | –1.436980000 |
| C | 0.943037000 | –2.423275000 | –1.090900000 |
| C | 1.826088000 | –3.366606000 | –1.619210000 |
| C | 0.407782000 | 1.404316000 | –1.436980000 |
| C | 1.627099000 | 2.028332000 | –1.090900000 |
| C | 2.002522000 | 3.264741000 | –1.619210000 |
| C | 1.184978000 | 3.924612000 | –2.541520000 |
| C | –0.000004000 | 3.307193000 | –2.936950000 |
| C | –0.374897000 | 2.069538000 | –2.398610000 |
| C | –2.864111000 | –1.653600000 | –2.936950000 |
| C | –1.604824000 | –1.359439000 | –2.398610000 |
| C | –1.420065000 | –0.349008000 | –1.436980000 |
| C | –2.570136000 | 0.394943000 | –1.090900000 |
| C | –3.828610000 | 0.101865000 | –1.619210000 |
| C | –3.991303000 | –0.936085000 | –2.541520000 |
| C | 0.000000000 | 0.000000000 | –0.865340000 |
| H | –3.498647000 | 3.725560000 | 2.954680000 |
| H | –1.735085000 | 4.410487000 | 1.309380000 |
| H | –0.180170000 | 2.775154000 | 0.404750000 |
| H | –2.060934000 | –0.314070000 | 2.752380000 |
| H | –3.601024000 | 1.328353000 | 3.675320000 |
| H | 2.493439000 | –1.231546000 | 0.404750000 |
| H | 4.687136000 | –0.702616000 | 1.309380000 |
| H | 4.975753000 | 1.167137000 | 2.954680000 |
| H | 2.950900000 | 2.454402000 | 3.675320000 |
| H | 0.758474000 | 1.941856000 | 2.752380000 |
| H | 0.650124000 | –3.782755000 | 3.675320000 |
| H | 1.302460000 | –1.627786000 | 2.752380000 |

| | | | |
|---|---|---|---|
| H | –2.313269000 | –1.543608000 | 0.404750000 |
| H | –2.952051000 | –3.707871000 | 1.309380000 |
| H | –1.477106000 | –4.892698000 | 2.954680000 |
| H | 3.498647000 | –3.725560000 | –2.954680000 |
| H | 3.601024000 | –1.328353000 | –3.675320000 |
| H | 2.060934000 | 0.314070000 | –2.752380000 |
| H | 0.180170000 | –2.775154000 | –0.404750000 |
| H | 1.735085000 | –4.410487000 | –1.309380000 |
| H | 2.313269000 | 1.543608000 | –0.404750000 |
| H | 2.952051000 | 3.707871000 | –1.309380000 |
| H | 1.477106000 | 4.892698000 | –2.954680000 |
| H | –0.650124000 | 3.782755000 | –3.675320000 |
| H | –1.302460000 | 1.627786000 | –2.752380000 |
| H | –2.950900000 | –2.454402000 | –3.675320000 |
| H | –0.758474000 | –1.941856000 | –2.752380000 |
| H | –2.493439000 | 1.231546000 | –0.404750000 |
| H | –4.687136000 | 0.702616000 | –1.309380000 |
| H | –4.975753000 | –1.167137000 | –2.954680000 |

B3LYP-D3(BJ)

| | | | |
|---|---|---|---|
| C | –2.822925000 | 3.003227000 | 2.416083000 |
| C | –1.800733000 | 3.374162000 | 1.537668000 |
| C | –0.899186000 | 2.426188000 | 1.055759000 |
| C | –0.996060000 | 1.062736000 | 1.405189000 |
| C | –1.994059000 | 0.725001000 | 2.332709000 |
| C | –2.898361000 | 1.673370000 | 2.824276000 |
| C | 1.418386000 | 0.331245000 | 1.405189000 |
| C | 2.550734000 | –0.434376000 | 1.055759000 |
| C | 3.822476000 | –0.127600000 | 1.537668000 |
| C | 4.012333000 | 0.943111000 | 2.416083000 |
| C | 2.898361000 | 1.673370000 | 2.824276000 |
| C | 1.624898000 | 1.364405000 | 2.332709000 |
| C | 0.000000000 | –3.346739000 | 2.824276000 |
| C | 0.369160000 | –2.089406000 | 2.332709000 |
| C | –0.422327000 | –1.393981000 | 1.405189000 |
| C | –1.651548000 | –1.991812000 | 1.055759000 |
| C | –2.021743000 | –3.246561000 | 1.537668000 |
| C | –1.189408000 | –3.946338000 | 2.416083000 |
| C | 0.000000000 | 0.000000000 | 0.850679000 |
| C | 2.822925000 | –3.003227000 | –2.416083000 |
| C | 2.898361000 | –1.673370000 | –2.824276000 |
| C | 1.994059000 | –0.725001000 | –2.332709000 |
| C | 0.996060000 | –1.062736000 | –1.405189000 |
| C | 0.899186000 | –2.426188000 | –1.055759000 |
| C | 1.800733000 | –3.374162000 | –1.537668000 |
| C | 0.422327000 | 1.393981000 | –1.405189000 |
| C | 1.651548000 | 1.991812000 | –1.055759000 |
| C | 2.021743000 | 3.246561000 | –1.537668000 |
| C | 1.189408000 | 3.946338000 | –2.416083000 |
| C | 0.000000000 | 3.346739000 | –2.824276000 |
| C | –0.369160000 | 2.089406000 | –2.332709000 |
| C | –2.898361000 | –1.673370000 | –2.824276000 |
| C | –1.624898000 | –1.364405000 | –2.332709000 |
| C | –1.418386000 | –0.331245000 | –1.405189000 |
| C | –2.550734000 | 0.434376000 | –1.055759000 |
| C | –3.822476000 | 0.127600000 | –1.537668000 |
| C | –4.012333000 | –0.943111000 | –2.416083000 |
| C | 0.000000000 | 0.000000000 | –0.850679000 |
| H | –3.533315000 | 3.743624000 | 2.789579000 |
| H | –1.693640000 | 4.415045000 | 1.224743000 |
| H | –0.106057000 | 2.768182000 | 0.401947000 |
| H | –2.086811000 | –0.296937000 | 2.688237000 |
| H | –3.665959000 | 1.357050000 | 3.533961000 |
| H | 2.450344000 | –1.292243000 | 0.401947000 |
| H | 4.670361000 | –0.740788000 | 1.224743000 |
| H | 5.008731000 | 1.188129000 | 2.789579000 |
| H | 3.008220000 | 2.496289000 | 3.533961000 |
| H | 0.786251000 | 1.955700000 | 2.688237000 |
| H | 0.657740000 | –3.853339000 | 3.533961000 |
| H | 1.300560000 | –1.658763000 | 2.688237000 |
| H | –2.344287000 | –1.475939000 | 0.401947000 |
| H | –2.976721000 | –3.674257000 | 1.224743000 |
| H | –1.475415000 | –4.931753000 | 2.789579000 |
| H | 3.533315000 | –3.743624000 | –2.789579000 |
| H | 3.665959000 | –1.357050000 | –3.533961000 |
| H | 2.086811000 | 0.296937000 | –2.688237000 |
| H | 0.106057000 | –2.768182000 | –0.401947000 |
| H | 1.693640000 | –4.415045000 | –1.224743000 |
| H | 2.344287000 | 1.475939000 | –0.401947000 |
| H | 2.976721000 | 3.674257000 | –1.224743000 |
| H | 1.475415000 | 4.931753000 | –2.789579000 |
| H | –0.657740000 | 3.853339000 | –3.533961000 |
| H | –1.300560000 | 1.658763000 | –2.688237000 |
| H | –3.008220000 | –2.496289000 | –3.533961000 |
| H | –0.786251000 | –1.955700000 | –2.688237000 |
| H | –2.450344000 | 1.292243000 | –0.401947000 |
| H | –4.670361000 | 0.740788000 | –1.224743000 |
| H | –5.008731000 | –1.188129000 | –2.789579000 |

M06-2X

| | | | |
|---|---|---|---|
| C | 1.856614000 | 3.695539000 | 2.367085000 |
| C | 2.562163000 | 2.837270000 | 1.523315000 |
| C | 1.982680000 | 1.659069000 | 1.063478000 |
| C | 0.661148000 | 1.298440000 | 1.399127000 |
| C | 0.000000000 | 2.142474000 | 2.301207000 |
| C | 0.580544000 | 3.324444000 | 2.770645000 |
| C | 0.793908000 | –1.221791000 | 1.399127000 |
| C | 0.445456000 | –2.546586000 | 1.063478000 |
| C | 1.176066000 | –3.637533000 | 1.523315000 |
| C | 2.272124000 | –3.455645000 | 2.367085000 |
| C | 2.588781000 | –2.164988000 | 2.770645000 |
| C | 1.855437000 | –1.071237000 | 2.301207000 |
| C | –3.169325000 | –1.159456000 | 2.770645000 |
| C | –1.855437000 | –1.071237000 | 2.301207000 |
| C | –1.455056000 | –0.076649000 | 1.399127000 |
| C | –2.428136000 | 0.887517000 | 1.063478000 |
| C | –3.738229000 | 0.800263000 | 1.523315000 |
| C | –4.128738000 | –0.239894000 | 2.367085000 |
| C | 0.000000000 | 0.000000000 | 0.846113000 |
| C | –1.856614000 | –3.695539000 | –2.367085000 |
| C | –0.580544000 | –3.324444000 | –2.770645000 |
| C | 0.000000000 | –2.142474000 | –2.301207000 |
| C | –0.661148000 | –1.298440000 | –1.399127000 |
| C | –1.982680000 | –1.659069000 | –1.063478000 |
| C | –2.562163000 | –2.837270000 | –1.523315000 |
| C | 1.455056000 | 0.076649000 | –1.399127000 |
| C | 2.428136000 | –0.887517000 | –1.063478000 |
| C | 3.738229000 | –0.800263000 | –1.523315000 |
| C | 4.128738000 | 0.239894000 | –2.367085000 |
| C | 3.169325000 | 1.159456000 | –2.770645000 |
| C | 1.855437000 | 1.071237000 | –2.301207000 |
| C | –2.588781000 | 2.164988000 | –2.770645000 |
| C | –1.855437000 | 1.071237000 | –2.301207000 |
| C | –0.793908000 | 1.221791000 | –1.399127000 |
| C | –0.445456000 | 2.546586000 | –1.063478000 |
| C | –1.176066000 | 3.637533000 | –1.523315000 |
| C | –2.272124000 | 3.455645000 | –2.367085000 |
| C | 0.000000000 | 0.000000000 | –0.846113000 |
| H | 2.307410000 | 4.623610000 | 2.721097000 |
| H | 3.582644000 | 3.077951000 | 1.221640000 |
| H | 2.592964000 | 1.010191000 | 0.443110000 |
| H | –0.994958000 | 1.896474000 | 2.661699000 |
| H | 0.016484000 | 3.952615000 | 3.461571000 |
| H | –0.421631000 | –2.750668000 | 0.443110000 |
| H | 0.874262000 | –4.641636000 | 1.221640000 |
| H | 2.850459000 | –4.310081000 | 2.721097000 |
| H | 3.414823000 | –1.990583000 | 3.461571000 |
| H | 2.139874000 | –0.086578000 | 2.661699000 |
| H | –3.431307000 | –1.962032000 | 3.461571000 |
| H | –1.144915000 | –1.809896000 | 2.661699000 |
| H | –2.171333000 | 1.740477000 | 0.443110000 |
| H | –4.456905000 | 1.563685000 | 1.221640000 |
| H | –5.157869000 | –0.313529000 | 2.721097000 |
| H | –2.307410000 | –4.623610000 | –2.721097000 |
| H | –0.016484000 | –3.952615000 | –3.461571000 |
| H | 0.994958000 | –1.896474000 | –2.661699000 |
| H | –2.592964000 | –1.010191000 | –0.443110000 |
| H | –3.582644000 | –3.077951000 | –1.221640000 |
| H | 2.171333000 | –1.740477000 | –0.443110000 |
| H | 4.456905000 | –1.563685000 | –1.221640000 |
| H | 5.157869000 | 0.313529000 | –2.721097000 |
| H | 3.431307000 | 1.962032000 | –3.461571000 |
| H | 1.144915000 | 1.809896000 | –2.661699000 |
| H | –3.414823000 | 1.990583000 | –3.461571000 |
| H | –2.139874000 | 0.086578000 | –2.661699000 |
| H | 0.421631000 | 2.750668000 | –0.443110000 |
| H | –0.874262000 | 4.641636000 | –1.221640000 |
| H | –2.850459000 | 4.310081000 | –2.721097000 |

Me-**1**$_2$

B3LYP

| | | | |
|---|---|---|---|
| C | –4.071351000 | –0.027826000 | 2.556695000 |
| C | –3.717117000 | 0.950589000 | 1.612496000 |
| C | –2.424522000 | 0.942694000 | 1.082093000 |
| C | –1.461897000 | –0.028708000 | 1.440984000 |
| C | –1.848390000 | –0.958421000 | 2.416206000 |
| C | –3.139961000 | –0.976255000 | 2.976979000 |
| C | 0.706087000 | 1.280394000 | 1.440984000 |
| C | 2.028658000 | 1.628350000 | 1.082093000 |
| C | 2.681793000 | 2.743823000 | 1.612496000 |

| | | | |
|---|---|---|---|
| C | 2.011578000 | 3.539806000 | 2.556695000 |
| C | 0.724519000 | 3.207413000 | 2.976979000 |
| C | 0.094178000 | 2.079963000 | 2.416206000 |
| C | 2.415442000 | –2.231158000 | 2.976979000 |
| C | 1.754212000 | –1.121542000 | 2.416206000 |
| C | 0.755810000 | –1.251686000 | 1.440984000 |
| C | 0.395864000 | –2.571045000 | 1.082093000 |
| C | 1.035324000 | –3.694412000 | 1.612496000 |
| C | 2.059773000 | –3.511980000 | 2.556695000 |
| C | 0.000000000 | 0.000000000 | 0.863813000 |
| C | 4.071351000 | 0.027826000 | –2.556695000 |
| C | 3.139961000 | 0.976255000 | –2.976979000 |
| C | 1.848390000 | 0.958421000 | –2.416206000 |
| C | 1.461897000 | 0.028708000 | –1.440984000 |
| C | 2.424522000 | –0.942694000 | –1.082093000 |
| C | 3.717117000 | –0.950589000 | –1.612496000 |
| C | –0.755810000 | 1.251686000 | –1.440984000 |
| C | –0.395864000 | 2.571045000 | –1.082093000 |
| C | –1.035324000 | 3.694412000 | –1.612496000 |
| C | –2.059773000 | 3.511980000 | –2.556695000 |
| C | –2.415442000 | 2.231158000 | –2.976979000 |
| C | –1.754212000 | 1.121542000 | –2.416206000 |
| C | –0.724519000 | –3.207413000 | –2.976979000 |
| C | –0.094178000 | –2.079963000 | –2.416206000 |
| C | –0.706087000 | –1.280394000 | –1.440984000 |
| C | –2.028658000 | –1.628350000 | –1.082093000 |
| C | –2.681793000 | –2.743823000 | –1.612496000 |
| C | –2.011578000 | –3.539806000 | –2.556695000 |
| C | 0.000000000 | 0.000000000 | –0.863813000 |
| C | –4.720566000 | 1.999198000 | 1.190330000 |
| C | –3.489457000 | –2.014639000 | 4.018114000 |
| C | 4.091640000 | 3.088531000 | 1.190330000 |
| C | 0.000000000 | 4.029278000 | 4.018114000 |
| C | 3.489457000 | –2.014639000 | 4.018114000 |
| C | 0.628926000 | –5.087729000 | 1.190330000 |
| C | 3.489457000 | 2.014639000 | –4.018114000 |
| C | 4.720566000 | –1.999198000 | –1.190330000 |
| C | –0.628926000 | 5.087729000 | –1.190330000 |
| C | –3.489457000 | 2.014639000 | –4.018114000 |
| C | 0.000000000 | –4.029278000 | –4.018114000 |
| C | –4.091640000 | –3.088531000 | –1.190330000 |
| H | –5.081026000 | –0.033386000 | 2.978782000 |
| H | –2.165434000 | 1.727583000 | 0.379387000 |
| H | –1.141136000 | –1.704436000 | 2.770701000 |
| H | 2.578847000 | 1.011529000 | 0.379387000 |
| H | 2.511600000 | 4.416990000 | 2.978782000 |
| H | –0.905517000 | 1.840471000 | 2.770701000 |
| H | 2.046653000 | –0.136035000 | 2.770701000 |
| H | –0.413414000 | –2.739112000 | 0.379387000 |
| H | 2.569426000 | –4.383604000 | 2.978782000 |
| H | 5.081026000 | 0.033386000 | –2.978782000 |
| H | 1.141136000 | 1.704436000 | –2.770701000 |
| H | 2.165434000 | –1.727583000 | –0.379387000 |
| H | 0.413414000 | 2.739112000 | –0.379387000 |
| H | –2.569426000 | 4.383604000 | –2.978782000 |
| H | –2.046653000 | 0.136035000 | –2.770701000 |
| H | 0.905517000 | –1.840471000 | –2.770701000 |
| H | –2.578847000 | –1.011529000 | –0.379387000 |
| H | –2.511600000 | –4.416990000 | –2.978782000 |
| H | –4.295600000 | 2.689454000 | 0.447250000 |
| H | –5.063097000 | 2.596122000 | 2.052792000 |
| H | –5.618817000 | 1.536817000 | 0.746846000 |
| H | –4.527846000 | –1.906189000 | 4.365063000 |
| H | –2.828836000 | –1.935585000 | 4.898073000 |
| H | –3.369989000 | –3.036913000 | 3.621376000 |
| H | 4.476936000 | 2.375372000 | 0.447250000 |
| H | 4.779856000 | 3.086709000 | 2.052792000 |
| H | 4.140331000 | 4.097630000 | 0.746846000 |
| H | 0.613115000 | 4.874324000 | 4.365063000 |
| H | –0.261847000 | 3.417636000 | 4.898073000 |
| H | –0.945050000 | 4.436953000 | 3.621376000 |
| H | 3.914731000 | –2.968135000 | 4.365063000 |
| H | 3.090684000 | –1.482052000 | 4.898073000 |
| H | 4.315038000 | –1.400039000 | 3.621376000 |
| H | –0.181335000 | –5.064826000 | 0.447250000 |
| H | 0.283241000 | –5.682831000 | 2.052792000 |
| H | 1.478486000 | –5.634447000 | 0.746846000 |
| H | 2.828836000 | 1.935585000 | –4.898073000 |
| H | 4.527846000 | 1.906189000 | –4.365063000 |
| H | 3.369989000 | 3.036913000 | –3.621376000 |
| H | 5.618817000 | –1.536817000 | –0.746846000 |
| H | 5.063097000 | –2.596122000 | –2.052792000 |
| H | 4.295600000 | –2.689454000 | –0.447250000 |
| H | –1.478486000 | 5.634447000 | –0.746846000 |
| H | –0.283241000 | 5.682831000 | –2.052792000 |
| H | 0.181335000 | 5.064826000 | –0.447250000 |
| H | –3.090684000 | 1.482052000 | –4.898073000 |
| H | –3.914731000 | 2.968135000 | –4.365063000 |
| H | –4.315038000 | 1.400039000 | –3.621376000 |
| H | 0.261847000 | –3.417636000 | –4.898073000 |
| H | –0.613115000 | –4.874324000 | –4.365063000 |
| H | 0.945050000 | –4.436953000 | –3.621376000 |
| H | –4.140331000 | –4.097630000 | –0.746846000 |
| H | –4.779856000 | –3.086709000 | –2.052792000 |
| H | –4.476936000 | –2.375372000 | –0.447250000 |

B3LYP-D3(BJ)

| | | | |
|---|---|---|---|
| C | –4.162574000 | –0.051215000 | 2.232530000 |
| C | –3.707901000 | 1.001584000 | 1.422061000 |
| C | –2.369149000 | 1.024021000 | 1.033052000 |
| C | –1.460466000 | 0.006314000 | 1.394919000 |
| C | –1.936305000 | –0.982389000 | 2.261895000 |
| C | –3.278387000 | –1.033055000 | 2.677682000 |
| C | 0.735701000 | 1.261644000 | 1.394919000 |
| C | 2.071402000 | 1.539732000 | 1.033052000 |
| C | 2.721348000 | 2.710345000 | 1.422061000 |
| C | 2.036933000 | 3.630502000 | 2.232530000 |
| C | 0.744541000 | 3.355694000 | 2.677682000 |
| C | 0.117378000 | 2.168084000 | 2.261895000 |
| C | 2.533846000 | –2.322639000 | 2.677682000 |
| C | 1.818926000 | –1.185695000 | 2.261895000 |
| C | 0.724765000 | –1.267958000 | 1.394919000 |
| C | 0.297746000 | –2.563753000 | 1.033052000 |
| C | 0.986553000 | –3.711929000 | 1.422061000 |
| C | 2.125640000 | –3.579287000 | 2.232530000 |
| C | 0.000000000 | 0.000000000 | 0.862515000 |
| C | 4.162574000 | 0.051215000 | –2.232530000 |
| C | 3.278387000 | 1.033055000 | –2.677682000 |
| C | 1.936305000 | 0.982389000 | –2.261895000 |
| C | 1.460466000 | –0.006314000 | –1.394919000 |
| C | 2.369149000 | –1.024021000 | –1.033052000 |
| C | 3.707901000 | –1.001584000 | –1.422061000 |
| C | –0.724765000 | 1.267958000 | –1.394919000 |
| C | –0.297746000 | 2.563753000 | –1.033052000 |
| C | –0.986553000 | 3.711929000 | –1.422061000 |
| C | –2.125640000 | 3.579287000 | –2.232530000 |
| C | –2.533846000 | 2.322639000 | –2.677682000 |
| C | –1.818926000 | 1.185695000 | –2.261895000 |
| C | –0.744541000 | –3.355694000 | –2.677682000 |
| C | –0.117378000 | –2.168084000 | –2.261895000 |
| C | –0.735701000 | –1.261644000 | –1.394919000 |
| C | –2.071402000 | –1.539732000 | –1.033052000 |
| C | –2.721348000 | –2.710345000 | –1.422061000 |
| C | –2.036933000 | –3.630502000 | –2.232530000 |
| C | 0.000000000 | 0.000000000 | –0.862515000 |
| C | –4.647514000 | 2.095517000 | 0.977227000 |
| C | –3.737243000 | –2.157698000 | 3.573461000 |
| C | 4.138528000 | 2.977107000 | 0.977227000 |
| C | 0.000000000 | 4.315397000 | 3.573461000 |
| C | 3.737243000 | –2.157698000 | 3.573461000 |
| C | 0.508986000 | –5.072624000 | 0.977227000 |
| C | 3.737243000 | 2.157698000 | –3.573461000 |
| C | 4.647514000 | –2.095517000 | –0.977227000 |
| C | –0.508986000 | 5.072624000 | –0.977227000 |
| C | –3.737243000 | 2.157698000 | –3.573461000 |
| C | 0.000000000 | –4.315397000 | –3.573461000 |
| C | –4.138528000 | –2.977107000 | –0.977227000 |
| H | –5.213660000 | –0.090873000 | 2.532739000 |
| H | –2.038677000 | 1.857150000 | 0.425395000 |
| H | –1.268340000 | –1.761504000 | 2.620013000 |
| H | 2.627678000 | 0.836971000 | 0.425395000 |
| H | 2.528132000 | 4.560599000 | 2.532739000 |
| H | –0.891337000 | 1.979167000 | 2.620013000 |
| H | 2.159678000 | –0.217663000 | 2.620013000 |
| H | –0.589000000 | –2.694121000 | 0.425395000 |
| H | 2.685529000 | –4.469726000 | 2.532739000 |
| H | 5.213660000 | 0.090873000 | –2.532739000 |
| H | 1.268340000 | 1.761504000 | –2.620013000 |
| H | 2.038677000 | –1.857150000 | –0.425395000 |
| H | 0.589000000 | 2.694121000 | –0.425395000 |
| H | –2.685529000 | 4.469726000 | –2.532739000 |
| H | –2.159678000 | 0.217663000 | –2.620013000 |
| H | 0.891337000 | –1.979167000 | –2.620013000 |
| H | –2.627678000 | –0.836971000 | –0.425395000 |
| H | –2.528132000 | –4.560599000 | –2.532739000 |
| H | –4.185964000 | 2.723007000 | 0.200839000 |
| H | –4.929679000 | 2.749002000 | 1.820788000 |
| H | –5.581783000 | 1.676509000 | 0.569287000 |
| H | –4.798461000 | –2.053049000 | 3.842590000 |
| H | –3.149891000 | –2.189331000 | 4.506075000 |
| H | –3.606890000 | –3.135351000 | 3.079809000 |
| H | 4.451175000 | 2.263648000 | 0.200839000 |
| H | 4.845545000 | 2.894726000 | 1.820788000 |
| H | 4.242790000 | 3.995711000 | 0.569287000 |
| H | 0.621238000 | 5.182113000 | 3.842590000 |

| | | | |
|---|---|---|---|
| H | –0.321071000 | 3.822551000 | 4.506075000 |
| H | –0.911849000 | 4.691334000 | 3.079809000 |
| H | 4.177223000 | –3.129064000 | 3.842590000 |
| H | 3.470962000 | –1.633220000 | 4.506075000 |
| H | 4.518739000 | –1.555983000 | 3.079809000 |
| H | –0.265211000 | –4.986655000 | 0.200839000 |
| H | 0.084134000 | –5.643728000 | 1.820788000 |
| H | 1.338992000 | –5.672220000 | 0.569287000 |
| H | 3.149891000 | 2.189331000 | –4.506075000 |
| H | 4.798461000 | 2.053049000 | –3.842590000 |
| H | 3.606890000 | 3.135351000 | –3.079809000 |
| H | 5.581783000 | –1.676509000 | –0.569287000 |
| H | 4.929679000 | –2.749002000 | –1.820788000 |
| H | 4.185964000 | –2.723007000 | –0.200839000 |
| H | –1.338992000 | 5.672220000 | –0.569287000 |
| H | –0.084134000 | 5.643728000 | –1.820788000 |
| H | 0.265211000 | 4.986655000 | –0.200839000 |
| H | –3.470962000 | 1.633220000 | –4.506075000 |
| H | –4.177223000 | 3.129064000 | –3.842590000 |
| H | –4.518739000 | 1.555983000 | –3.079809000 |
| H | 0.321071000 | –3.822551000 | –4.506075000 |
| H | –0.621238000 | –5.182113000 | –3.842590000 |
| H | 0.911849000 | –4.691334000 | –3.079809000 |
| H | –4.242790000 | –3.995711000 | –0.569287000 |
| H | –4.845545000 | –2.894726000 | –1.820788000 |
| H | –4.451175000 | –2.263648000 | –0.200839000 |

M06-2X

| | | | |
|---|---|---|---|
| C | –2.132645000 | 3.585031000 | 2.199820000 |
| C | –0.979618000 | 3.713529000 | 1.413210000 |
| C | –0.287016000 | 2.566947000 | 1.038070000 |
| C | –0.721798000 | 1.270911000 | 1.390540000 |
| C | –1.826362000 | 1.195772000 | 2.241080000 |
| C | –2.545944000 | 2.333925000 | 2.641050000 |
| C | 1.461540000 | –0.010360000 | 1.390540000 |
| C | 2.366550000 | –1.034911000 | 1.038070000 |
| C | 3.705820000 | –1.008391000 | 1.413210000 |
| C | 4.171050000 | 0.054409000 | 2.199820000 |
| C | 3.294210000 | 1.037889000 | 2.641050000 |
| C | 1.948750000 | 0.983790000 | 2.241080000 |
| C | –0.748267000 | –3.371814000 | 2.641050000 |
| C | –0.122388000 | –2.179562000 | 2.241080000 |
| C | –0.739742000 | –1.260551000 | 1.390540000 |
| C | –2.079534000 | –1.532037000 | 1.038070000 |
| C | –2.726202000 | –2.705139000 | 1.413210000 |
| C | –2.038405000 | –3.639440000 | 2.199820000 |
| C | 0.000000000 | 0.000000000 | 0.856100000 |
| C | 2.132645000 | –3.585031000 | –2.199820000 |
| C | 2.545944000 | –2.333925000 | –2.641050000 |
| C | 1.826362000 | –1.195772000 | –2.241080000 |
| C | 0.721798000 | –1.270911000 | –1.390540000 |
| C | 0.287016000 | –2.566947000 | –1.038070000 |
| C | 0.979618000 | –3.713529000 | –1.413210000 |
| C | 0.739742000 | 1.260551000 | –1.390540000 |
| C | 2.079534000 | 1.532037000 | –1.038070000 |
| C | 2.726202000 | 2.705139000 | –1.413210000 |
| C | 2.038405000 | 3.639440000 | –2.199820000 |
| C | 0.748267000 | 3.371814000 | –2.641050000 |
| C | 0.122388000 | 2.179562000 | –2.241080000 |
| C | –3.294210000 | –1.037889000 | –2.641050000 |
| C | –1.948750000 | –0.983790000 | –2.241080000 |
| C | –1.461540000 | 0.010360000 | –1.390540000 |
| C | –2.366550000 | 1.034911000 | –1.038070000 |
| C | –3.705820000 | 1.008391000 | –1.413210000 |
| C | –4.171050000 | –0.054409000 | –2.199820000 |
| C | 0.000000000 | 0.000000000 | –0.856100000 |
| C | –0.494813000 | 5.073943000 | 0.981800000 |
| C | –3.760698000 | 2.171243000 | 3.518770000 |
| C | 4.641570000 | –2.108451000 | 0.981800000 |
| C | 3.760701000 | 2.171239000 | 3.518770000 |
| C | –0.000002000 | –4.342482000 | 3.518770000 |
| C | –4.146757000 | –2.965492000 | 0.981800000 |
| C | 3.760698000 | –2.171243000 | –3.518770000 |
| C | 0.494813000 | –5.073943000 | –0.981800000 |
| C | 4.146757000 | 2.965492000 | –0.981800000 |
| C | 0.000002000 | 4.342482000 | –3.518770000 |
| C | –3.760701000 | –2.171239000 | –3.518770000 |
| C | –4.641570000 | 2.108451000 | –0.981800000 |
| H | –2.695789000 | 4.477346000 | 2.487860000 |
| H | 0.616718000 | 2.705173000 | 0.452190000 |
| H | –2.176939000 | 0.233990000 | 2.609210000 |
| H | 2.034390000 | –1.886680000 | 0.452190000 |
| H | 5.225390000 | 0.095949000 | 2.487860000 |
| H | 1.291110000 | 1.768290000 | 2.609210000 |
| H | 0.885829000 | –2.002279000 | 2.609210000 |
| H | –2.651108000 | –0.818493000 | 0.452190000 |
| H | –2.529601000 | –4.573295000 | 2.487860000 |
| H | 2.695789000 | –4.477346000 | –2.487860000 |
| H | 2.176939000 | –0.233990000 | –2.609210000 |
| H | –0.616718000 | –2.705173000 | –0.452190000 |
| H | 2.651108000 | 0.818493000 | –0.452190000 |
| H | 2.529601000 | 4.573295000 | –2.487860000 |
| H | –0.885829000 | 2.002279000 | –2.609210000 |
| H | –1.291110000 | –1.768290000 | –2.609210000 |
| H | –2.034390000 | 1.886680000 | –0.452190000 |
| H | –5.225390000 | –0.095949000 | –2.487860000 |
| H | 0.321162000 | 4.987753000 | 0.250950000 |
| H | –0.127622000 | 5.654070000 | 1.842590000 |
| H | –1.309213000 | 5.653926000 | 0.522830000 |
| H | –4.199670000 | 3.143205000 | 3.779400000 |
| H | –3.503660000 | 1.647978000 | 4.451870000 |
| H | –4.531986000 | 1.571372000 | 3.011850000 |
| H | 4.158939000 | –2.772011000 | 0.250950000 |
| H | 4.960379000 | –2.716511000 | 1.842590000 |
| H | 5.551050000 | –1.693151000 | 0.522830000 |
| H | 4.821930000 | 2.065419000 | 3.779400000 |
| H | 3.179021000 | 2.210269000 | 4.451870000 |
| H | 3.626841000 | 3.139129000 | 3.011850000 |
| H | –0.622260000 | –5.208624000 | 3.779400000 |
| H | 0.324639000 | –3.858247000 | 4.451870000 |
| H | 0.905145000 | –4.710501000 | 3.011850000 |
| H | –4.480102000 | –2.215742000 | 0.250950000 |
| H | –4.832757000 | –2.937559000 | 1.842590000 |
| H | –4.241837000 | –3.960774000 | 0.522830000 |
| H | 3.503660000 | –1.647978000 | –4.451870000 |
| H | 4.199670000 | –3.143205000 | –3.779400000 |
| H | 4.531986000 | –1.571372000 | –3.011850000 |
| H | 1.309213000 | –5.653926000 | –0.522830000 |
| H | 0.127622000 | –5.654070000 | –1.842590000 |
| H | –0.321162000 | –4.987753000 | –0.250950000 |
| H | 4.241837000 | 3.960774000 | –0.522830000 |
| H | 4.832757000 | 2.937559000 | –1.842590000 |
| H | 4.480102000 | 2.215742000 | –0.250950000 |
| H | –0.324639000 | 3.858247000 | –4.451870000 |
| H | 0.622260000 | 5.208624000 | –3.779400000 |
| H | –0.905145000 | 4.710501000 | –3.011850000 |
| H | –3.179021000 | –2.210269000 | –4.451870000 |
| H | –4.821930000 | –2.065419000 | –3.779400000 |
| H | –3.626841000 | –3.139129000 | –3.011850000 |
| H | –5.551050000 | 1.693151000 | –0.522830000 |
| H | –4.960379000 | 2.716511000 | –1.842590000 |
| H | –4.158939000 | 2.772011000 | –0.250950000 |

$^{i}$Pr-**1**$_2$

B3LYP

| | | | |
|---|---|---|---|
| C | –3.794473000 | 1.164245000 | 2.770870000 |
| C | –3.272895000 | 1.968600000 | 1.744580000 |
| C | –2.091121000 | 1.563625000 | 1.109940000 |
| C | –1.414102000 | 0.371402000 | 1.457300000 |
| C | –1.972278000 | –0.386854000 | 2.499430000 |
| C | –3.152248000 | –0.008714000 | 3.165880000 |
| C | 1.028695000 | 1.038947000 | 1.457300000 |
| C | 2.399699000 | 1.029151000 | 1.109940000 |
| C | 3.341305000 | 1.850110000 | 1.744580000 |
| C | 2.905502000 | 2.703988000 | 2.770870000 |
| C | 1.568577000 | 2.734284000 | 3.165880000 |
| C | 0.651114000 | 1.901470000 | 2.499430000 |
| C | 1.583671000 | –2.725570000 | 3.165880000 |
| C | 1.321164000 | –1.514616000 | 2.499430000 |
| C | 0.385407000 | –1.410349000 | 1.457300000 |
| C | –0.308579000 | –2.592776000 | 1.109940000 |
| C | –0.068410000 | –3.818710000 | 1.744580000 |
| C | 0.888971000 | –3.868232000 | 2.770870000 |
| C | 0.000000000 | 0.000000000 | 0.852610000 |
| C | 3.794473000 | –1.164245000 | –2.770870000 |
| C | 3.152248000 | 0.008714000 | –3.165880000 |
| C | 1.972278000 | 0.386854000 | –2.499430000 |
| C | 1.414102000 | –0.371402000 | –1.457300000 |
| C | 2.091121000 | –1.563625000 | –1.109940000 |
| C | 3.272895000 | –1.968600000 | –1.744580000 |
| C | –0.385407000 | 1.410349000 | –1.457300000 |
| C | 0.308579000 | 2.592776000 | –1.109940000 |
| C | 0.068410000 | 3.818710000 | –1.744580000 |
| C | –0.888971000 | 3.868232000 | –2.770870000 |
| C | –1.583671000 | 2.725570000 | –3.165880000 |
| C | –1.321164000 | 1.514616000 | –2.499430000 |
| C | –1.568577000 | –2.734284000 | –3.165880000 |
| C | –0.651114000 | –1.901470000 | –2.499430000 |
| C | –1.028695000 | –1.038947000 | –1.457300000 |
| C | –2.399699000 | –1.029151000 | –1.109940000 |
| C | –3.341305000 | –1.850110000 | –1.744580000 |
| C | –2.905502000 | –2.703988000 | –2.770870000 |
| C | 0.000000000 | 0.000000000 | –0.852610000 |

| | | | |
|---|---|---|---|
| C | –4.826235000 | –1.846109000 | –1.383950000 |
| C | –5.160694000 | –2.968068000 | –0.382420000 |
| C | –5.346573000 | –0.498313000 | –0.873100000 |
| C | –1.127398000 | –3.612201000 | –4.333530000 |
| C | 0.000000000 | –4.586688000 | –3.954830000 |
| C | –0.732036000 | –2.762564000 | –5.555590000 |
| C | 4.826235000 | 1.846109000 | 1.383950000 |
| C | 5.346573000 | 0.498313000 | 0.873100000 |
| C | 5.160694000 | 2.968068000 | 0.382420000 |
| C | 1.127398000 | 3.612201000 | 4.333530000 |
| C | 0.000000000 | 4.586688000 | 3.954830000 |
| C | 0.732036000 | 2.762564000 | 5.555590000 |
| C | 0.814340000 | 5.102697000 | –1.383950000 |
| C | 2.241735000 | 4.879424000 | –0.873100000 |
| C | 0.009925000 | 5.953327000 | –0.382420000 |
| C | –2.564558000 | 2.782456000 | –4.333530000 |
| C | –3.972188000 | 2.293344000 | –3.954830000 |
| C | –2.026433000 | 2.015244000 | –5.555590000 |
| C | 2.564558000 | –2.782456000 | 4.333530000 |
| C | 2.026433000 | –2.015244000 | 5.555590000 |
| C | 3.972188000 | –2.293344000 | 3.954830000 |
| C | –0.814340000 | –5.102697000 | 1.383950000 |
| C | –0.009925000 | –5.953327000 | 0.382420000 |
| C | –2.241735000 | –4.879424000 | 0.873100000 |
| C | –4.011895000 | 3.256587000 | 1.383950000 |
| C | –3.104838000 | 4.381112000 | 0.873100000 |
| C | –5.150770000 | 2.985258000 | 0.382420000 |
| C | –3.691957000 | –0.829745000 | 4.333530000 |
| C | –2.758469000 | –0.747321000 | 5.555590000 |
| C | –3.972188000 | –2.293344000 | 3.954830000 |
| C | 3.691957000 | 0.829745000 | –4.333530000 |
| C | 2.758469000 | 0.747321000 | –5.555590000 |
| C | 3.972188000 | 2.293344000 | –3.954830000 |
| C | 4.011895000 | –3.256587000 | –1.383950000 |
| C | 5.150770000 | –2.985258000 | –0.382420000 |
| C | 3.104838000 | –4.381112000 | –0.873100000 |
| H | –4.707135000 | 1.478533000 | 3.287510000 |
| H | –1.668068000 | 2.210747000 | 0.349750000 |
| H | –1.472548000 | –1.290336000 | 2.836220000 |
| H | 2.748597000 | 0.339216000 | 0.349750000 |
| H | 3.634015000 | 3.337233000 | 3.287510000 |
| H | –0.381189000 | 1.920432000 | 2.836220000 |
| H | 1.853738000 | –0.630096000 | 2.836220000 |
| H | –1.080529000 | –2.549963000 | 0.349750000 |
| H | 1.073121000 | –4.815765000 | 3.287510000 |
| H | 4.707135000 | –1.478533000 | –3.287510000 |
| H | 1.472548000 | 1.290336000 | –2.836220000 |
| H | 1.668068000 | –2.210747000 | –0.349750000 |
| H | 1.080529000 | 2.549963000 | –0.349750000 |
| H | –1.073121000 | 4.815765000 | –3.287510000 |
| H | –1.853738000 | 0.630096000 | –2.836220000 |
| H | 0.381189000 | –1.920432000 | –2.836220000 |
| H | –2.748597000 | –0.339216000 | –0.349750000 |
| H | –3.634015000 | –3.337233000 | –3.287510000 |
| H | –5.369021000 | –2.076271000 | –2.318660000 |
| H | –4.653624000 | –2.793736000 | 0.580760000 |
| H | –4.842748000 | –3.953833000 | –0.756290000 |
| H | –6.246687000 | –3.008381000 | –0.193610000 |
| H | –4.906770000 | –0.230976000 | 0.100860000 |
| H | –6.439722000 | –0.539765000 | –0.738410000 |
| H | –5.122944000 | 0.316379000 | –1.578760000 |
| H | –2.004920000 | –4.216969000 | –4.622260000 |
| H | –0.289708000 | –5.224331000 | –3.104480000 |
| H | 0.921367000 | –4.048968000 | –3.676240000 |
| H | 0.244708000 | –5.244094000 | –4.805540000 |
| H | –1.554968000 | –2.097247000 | –5.862610000 |
| H | –0.476270000 | –3.408130000 | –6.412420000 |
| H | 0.146266000 | –2.132544000 | –5.335880000 |
| H | 5.369021000 | 2.076271000 | 2.318660000 |
| H | 4.906770000 | 0.230976000 | –0.100860000 |
| H | 5.122944000 | –0.316379000 | 1.578760000 |
| H | 6.439722000 | 0.539765000 | 0.738410000 |
| H | 4.653624000 | 2.793736000 | –0.580760000 |
| H | 6.246687000 | 3.008381000 | 0.193610000 |
| H | 4.842748000 | 3.953833000 | 0.756290000 |
| H | 2.004920000 | 4.216969000 | 4.622260000 |
| H | 0.289708000 | 5.224331000 | 3.104480000 |
| H | –0.244708000 | 5.244094000 | 4.805540000 |
| H | –0.921367000 | 4.048968000 | 3.676240000 |
| H | 1.554968000 | 2.097247000 | 5.862610000 |
| H | –0.146266000 | 2.132544000 | 5.335880000 |
| H | 0.476270000 | 3.408130000 | 6.412420000 |
| H | 0.886407000 | 5.687844000 | –2.318660000 |
| H | 2.253354000 | 4.364876000 | 0.100860000 |
| H | 2.752411000 | 5.846846000 | –0.738410000 |
| H | 2.835464000 | 4.278410000 | –1.578760000 |
| H | –0.092634000 | 5.427025000 | 0.580760000 |
| H | –1.002746000 | 6.170859000 | –0.756290000 |
| H | 0.518010000 | 6.913980000 | –0.193610000 |
| H | –2.649542000 | 3.844796000 | –4.622260000 |
| H | –4.379549000 | 2.863060000 | –3.104480000 |
| H | –3.967193000 | 1.226557000 | –3.676240000 |
| H | –4.663872000 | 2.410123000 | –4.805540000 |
| H | –1.038785000 | 2.395265000 | –5.862610000 |
| H | –2.713392000 | 2.116527000 | –6.412420000 |
| H | –1.919970000 | 0.939602000 | –5.335880000 |
| H | 2.649542000 | –3.844796000 | 4.622260000 |
| H | 1.038785000 | –2.395265000 | 5.862610000 |
| H | 1.919970000 | –0.939602000 | 5.335880000 |
| H | 2.713392000 | –2.116527000 | 6.412420000 |
| H | 4.379549000 | –2.863060000 | 3.104480000 |
| H | 4.663872000 | –2.410123000 | 4.805540000 |
| H | 3.967193000 | –1.226557000 | 3.676240000 |
| H | –0.886407000 | –5.687844000 | 2.318660000 |
| H | 0.092634000 | –5.427025000 | –0.580760000 |
| H | –0.518010000 | –6.913980000 | 0.193610000 |
| H | 1.002746000 | –6.170859000 | 0.756290000 |
| H | –2.253354000 | –4.364876000 | –0.100860000 |
| H | –2.835464000 | –4.278410000 | 1.578760000 |
| H | –2.752411000 | –5.846846000 | 0.738410000 |
| H | –4.482613000 | 3.611573000 | 2.318660000 |
| H | –2.653416000 | 4.133900000 | –0.100860000 |
| H | –2.287480000 | 4.594789000 | 1.578760000 |
| H | –3.687311000 | 5.307081000 | 0.738410000 |
| H | –4.746258000 | 2.633289000 | –0.580760000 |
| H | –5.728678000 | 3.905599000 | 0.193610000 |
| H | –5.845494000 | 2.217026000 | 0.756290000 |
| H | –4.654462000 | –0.372173000 | 4.622260000 |
| H | –2.593753000 | 0.298018000 | 5.862610000 |
| H | –1.773704000 | –1.192942000 | 5.335880000 |
| H | –3.189662000 | –1.291603000 | 6.412420000 |
| H | –4.669257000 | –2.361271000 | 3.104480000 |
| H | –4.419164000 | –2.833970000 | 4.805540000 |
| H | –3.045826000 | –2.822411000 | 3.676240000 |
| H | 4.654462000 | 0.372173000 | –4.622260000 |
| H | 2.593753000 | –0.298018000 | –5.862610000 |
| H | 3.189662000 | 1.291603000 | –6.412420000 |
| H | 1.773704000 | 1.192942000 | –5.335880000 |
| H | 4.669257000 | 2.361271000 | –3.104480000 |
| H | 3.045826000 | 2.822411000 | –3.676240000 |
| H | 4.419164000 | 2.833970000 | –4.805540000 |
| H | 4.482613000 | –3.611573000 | –2.318660000 |
| H | 4.746258000 | –2.633289000 | 0.580760000 |
| H | 5.845494000 | –2.217026000 | –0.756290000 |
| H | 5.728678000 | –3.905599000 | –0.193610000 |
| H | 2.653416000 | –4.133900000 | 0.100860000 |
| H | 3.687311000 | –5.307081000 | –0.738410000 |
| H | 2.287480000 | –4.594789000 | –1.578760000 |

B3LYP-D3(BJ)

| | | | |
|---|---|---|---|
| C | 2.531406000 | 3.067818000 | 2.678130000 |
| C | 3.068767000 | 2.242586000 | 1.677500000 |
| C | 2.241400000 | 1.294074000 | 1.071590000 |
| C | 0.883741000 | 1.147979000 | 1.421840000 |
| C | 0.398267000 | 1.974919000 | 2.439970000 |
| C | 1.202556000 | 2.934268000 | 3.075890000 |
| C | 0.552309000 | –1.339332000 | 1.421840000 |
| C | 0.000001000 | –2.588146000 | 1.071590000 |
| C | 0.407753000 | –3.778924000 | 1.677500000 |
| C | 1.391105000 | –3.726171000 | 2.678130000 |
| C | 1.939872000 | –2.508578000 | 3.075890000 |
| C | 1.511197000 | –1.332369000 | 2.439970000 |
| C | –3.142428000 | –0.425690000 | 3.075890000 |
| C | –1.909463000 | –0.642550000 | 2.439970000 |
| C | –1.436050000 | 0.191353000 | 1.421840000 |
| C | –2.241401000 | 1.294072000 | 1.071590000 |
| C | –3.476520000 | 1.536338000 | 1.677500000 |
| C | –3.922511000 | 0.658353000 | 2.678130000 |
| C | 0.000000000 | 0.000000000 | 0.832580000 |
| C | –2.531406000 | –3.067818000 | –2.678130000 |
| C | –1.202556000 | –2.934268000 | –3.075890000 |
| C | –0.398267000 | –1.974919000 | –2.439970000 |
| C | –0.883741000 | –1.147979000 | –1.421840000 |
| C | –2.241400000 | –1.294074000 | –1.071590000 |
| C | –3.068767000 | –2.242586000 | –1.677500000 |
| C | 1.436050000 | –0.191353000 | –1.421840000 |
| C | 2.241401000 | –1.294072000 | –1.071590000 |
| C | 3.476520000 | –1.536338000 | –1.677500000 |
| C | 3.922511000 | –0.658353000 | –2.678130000 |
| C | 3.142428000 | 0.425690000 | –3.075890000 |
| C | 1.909463000 | 0.642550000 | –2.439970000 |
| C | –1.939872000 | 2.508578000 | –3.075890000 |
| C | –1.511197000 | 1.332369000 | –2.439970000 |
| C | –0.552309000 | 1.339332000 | –1.421840000 |
| C | –0.000001000 | 2.588146000 | –1.071590000 |

| | | | |
|---|---|---|---|
| C | –0.407753000 | 3.778924000 | –1.677500000 |
| C | –1.391105000 | 3.726171000 | –2.678130000 |
| C | 0.000000000 | 0.000000000 | –0.832580000 |
| C | 0.179576000 | 5.126522000 | –1.277920000 |
| C | –0.713675000 | 5.823590000 | –0.239350000 |
| C | 1.620821000 | 5.037254000 | –0.769460000 |
| C | –2.935880000 | 2.429557000 | –4.221260000 |
| C | –4.238448000 | 1.728829000 | –3.808720000 |
| C | –2.301496000 | 1.738340000 | –5.440230000 |
| C | –0.179576000 | –5.126522000 | 1.277920000 |
| C | –1.620821000 | –5.037254000 | 0.769460000 |
| C | 0.713675000 | –5.823590000 | 0.239350000 |
| C | 2.935880000 | –2.429557000 | 4.221260000 |
| C | 4.238448000 | –1.728829000 | 3.808720000 |
| C | 2.301496000 | –1.738340000 | 5.440230000 |
| C | 4.349910000 | –2.718779000 | –1.277920000 |
| C | 3.551979000 | –3.922299000 | –0.769460000 |
| C | 5.400215000 | –2.293735000 | –0.239350000 |
| C | 3.571998000 | 1.327768000 | –4.221260000 |
| C | 3.616434000 | 2.806189000 | –3.808720000 |
| C | 2.656194000 | 1.123984000 | –5.440230000 |
| C | –3.571998000 | –1.327768000 | 4.221260000 |
| C | –2.656194000 | –1.123984000 | 5.440230000 |
| C | –3.616434000 | –2.806189000 | 3.808720000 |
| C | –4.349910000 | 2.718779000 | 1.277920000 |
| C | –5.400215000 | 2.293735000 | 0.239350000 |
| C | –3.551979000 | 3.922299000 | 0.769460000 |
| C | 4.529486000 | 2.407743000 | 1.277920000 |
| C | 5.172800000 | 1.114955000 | 0.769460000 |
| C | 4.686540000 | 3.529855000 | 0.239350000 |
| C | 0.636118000 | 3.757325000 | 4.221260000 |
| C | 0.354699000 | 2.862324000 | 5.440230000 |
| C | –0.622014000 | 4.535018000 | 3.808720000 |
| C | –0.636118000 | –3.757325000 | –4.221260000 |
| C | –0.354699000 | –2.862324000 | –5.440230000 |
| C | 0.622014000 | –4.535018000 | –3.808720000 |
| C | –4.529486000 | –2.407743000 | –1.277920000 |
| C | –4.686540000 | –3.529855000 | –0.239350000 |
| C | –5.172800000 | –1.114955000 | –0.769460000 |
| H | 3.172171000 | 3.806337000 | 3.169180000 |
| H | 2.671832000 | 0.633234000 | 0.331600000 |
| H | –0.626409000 | 1.868622000 | 2.780140000 |
| H | –0.787519000 | –2.630491000 | 0.331600000 |
| H | 1.710298000 | –4.650349000 | 3.169180000 |
| H | 1.931479000 | –0.391825000 | 2.780140000 |
| H | –1.305069000 | –1.476797000 | 2.780140000 |
| H | –1.884313000 | 1.997257000 | 0.331600000 |
| H | –4.882470000 | 0.844013000 | 3.169180000 |
| H | –3.172171000 | –3.806337000 | –3.169180000 |
| H | 0.626409000 | –1.868622000 | –2.780140000 |
| H | –2.671832000 | –0.633234000 | –0.331600000 |
| H | 1.884313000 | –1.997257000 | –0.331600000 |
| H | 4.882470000 | –0.844013000 | –3.169180000 |
| H | 1.305069000 | 1.476797000 | –2.780140000 |
| H | –1.931479000 | 0.391825000 | –2.780140000 |
| H | 0.787519000 | 2.630491000 | –0.331600000 |
| H | –1.710298000 | 4.650349000 | –3.169180000 |
| H | 0.179749000 | 5.751362000 | –2.188970000 |
| H | –0.751019000 | 5.229902000 | 0.687240000 |
| H | –1.745004000 | 5.939964000 | –0.605280000 |
| H | –0.318620000 | 6.822749000 | 0.007940000 |
| H | 1.680073000 | 4.503926000 | 0.192020000 |
| H | 2.034146000 | 6.045932000 | –0.610760000 |
| H | 2.268696000 | 4.510433000 | –1.485200000 |
| H | –3.184459000 | 3.465048000 | –4.510550000 |
| H | –4.707015000 | 2.235403000 | –2.950960000 |
| H | –4.053266000 | 0.680838000 | –3.521610000 |
| H | –4.959799000 | 1.723843000 | –4.641870000 |
| H | –1.381439000 | 2.255856000 | –5.755150000 |
| H | –3.000465000 | 1.725014000 | –6.292660000 |
| H | –2.035380000 | 0.694457000 | –5.204050000 |
| H | –0.179749000 | –5.751362000 | 2.188970000 |
| H | –1.680073000 | –4.503926000 | –0.192020000 |
| H | –2.268696000 | –4.510433000 | 1.485200000 |
| H | –2.034146000 | –6.045932000 | 0.610760000 |
| H | 0.751019000 | –5.229902000 | –0.687240000 |
| H | 0.318620000 | –6.822749000 | –0.007940000 |
| H | 1.745004000 | –5.939964000 | 0.605280000 |
| H | 3.184459000 | –3.465048000 | 4.510550000 |
| H | 4.707015000 | –2.235403000 | 2.950960000 |
| H | 4.959799000 | –1.723843000 | 4.641870000 |
| H | 4.053266000 | –0.680838000 | 3.521610000 |
| H | 1.381439000 | –2.255856000 | 5.755150000 |
| H | 2.035380000 | –0.694457000 | 5.204050000 |
| H | 3.000465000 | –1.725014000 | 6.292660000 |
| H | 4.890952000 | –3.031348000 | –2.188970000 |
| H | 3.060478000 | –3.706949000 | 0.192020000 |
| H | 4.218858000 | –4.784588000 | –0.610760000 |
| H | 2.771801000 | –4.219965000 | –1.485200000 |
| H | 4.904737000 | –1.964550000 | 0.687240000 |
| H | 6.016662000 | –1.458764000 | –0.605280000 |
| H | 6.067984000 | –3.135442000 | 0.007940000 |
| H | 4.593049000 | 1.025299000 | –4.510550000 |
| H | 4.289423000 | 2.958693000 | –2.950960000 |
| H | 2.616256000 | 3.169812000 | –3.521610000 |
| H | 3.972792000 | 3.433391000 | –4.641870000 |
| H | 2.644348000 | 0.068433000 | –5.755150000 |
| H | 2.994139000 | 1.735972000 | –6.292660000 |
| H | 1.619107000 | 1.415462000 | –5.204050000 |
| H | –4.593049000 | –1.025299000 | 4.510550000 |
| H | –2.644348000 | –0.068433000 | 5.755150000 |
| H | –1.619107000 | –1.415462000 | 5.204050000 |
| H | –2.994139000 | –1.735972000 | 6.292660000 |
| H | –4.289423000 | –2.958693000 | 2.950960000 |
| H | –3.972792000 | –3.433391000 | 4.641870000 |
| H | –2.616256000 | –3.169812000 | 3.521610000 |
| H | –4.890952000 | 3.031348000 | 2.188970000 |
| H | –4.904737000 | 1.964550000 | –0.687240000 |
| H | –6.067984000 | 3.135442000 | –0.007940000 |
| H | –6.016662000 | 1.458764000 | 0.605280000 |
| H | –3.060478000 | 3.706949000 | –0.192020000 |
| H | –2.771801000 | 4.219965000 | 1.485200000 |
| H | –4.218858000 | 4.784588000 | 0.610760000 |
| H | 5.070700000 | 2.720014000 | 2.188970000 |
| H | 4.740551000 | 0.796977000 | –0.192020000 |
| H | 5.040497000 | 0.290468000 | 1.485200000 |
| H | 6.253003000 | 1.261344000 | 0.610760000 |
| H | 4.153719000 | 3.265352000 | –0.687240000 |
| H | 5.749364000 | 3.687307000 | –0.007940000 |
| H | 4.271658000 | 4.481200000 | 0.605280000 |
| H | 1.408590000 | 4.490347000 | 4.510550000 |
| H | 1.262909000 | 2.324290000 | 5.755150000 |
| H | –0.416273000 | 2.109919000 | 5.204050000 |
| H | –0.006326000 | 3.460986000 | 6.292660000 |
| H | –0.417592000 | 5.194096000 | 2.950960000 |
| H | –0.987008000 | 5.157234000 | 4.641870000 |
| H | –1.437010000 | 3.850650000 | 3.521610000 |
| H | –1.408590000 | –4.490347000 | –4.510550000 |
| H | –1.262909000 | –2.324290000 | –5.755150000 |
| H | 0.006326000 | –3.460986000 | –6.292660000 |
| H | 0.416273000 | –2.109919000 | –5.204050000 |
| H | 0.417592000 | –5.194096000 | –2.950960000 |
| H | 1.437010000 | –3.850650000 | –3.521610000 |
| H | 0.987008000 | –5.157234000 | –4.641870000 |
| H | –5.070700000 | –2.720014000 | –2.188970000 |
| H | –4.153719000 | –3.265352000 | 0.687240000 |
| H | –4.271658000 | –4.481200000 | –0.605280000 |
| H | –5.749364000 | –3.687307000 | 0.007940000 |
| H | –4.740551000 | –0.796977000 | 0.192020000 |
| H | –6.253003000 | –1.261344000 | –0.610760000 |
| H | –5.040497000 | –0.290468000 | –1.485200000 |

M06-2X

| | | | |
|---|---|---|---|
| C | 2.514355000 | 3.099590000 | 2.654270000 |
| C | 3.055809000 | 2.250675000 | 1.679380000 |
| C | 2.235521000 | 1.290676000 | 1.089600000 |
| C | 0.874001000 | 1.154709000 | 1.424910000 |
| C | 0.385484000 | 2.001510000 | 2.423330000 |
| C | 1.186384000 | 2.972499000 | 3.044890000 |
| C | 0.563007000 | –1.334261000 | 1.424910000 |
| C | –0.000002000 | –2.581356000 | 1.089600000 |
| C | 0.421237000 | –3.771746000 | 1.679380000 |
| C | 1.427146000 | –3.727290000 | 2.654270000 |
| C | 1.981067000 | –2.513688000 | 3.044890000 |
| C | 1.540616000 | –1.334594000 | 2.423330000 |
| C | –3.167452000 | –0.458810000 | 3.044890000 |
| C | –1.926101000 | –0.666916000 | 2.423330000 |
| C | –1.437007000 | 0.179553000 | 1.424910000 |
| C | –2.235519000 | 1.290680000 | 1.089600000 |
| C | –3.477046000 | 1.521071000 | 1.679380000 |
| C | –3.941501000 | 0.627700000 | 2.654270000 |
| C | 0.000000000 | 0.000000000 | 0.834600000 |
| C | –2.514355000 | –3.099590000 | –2.654270000 |
| C | –1.186384000 | –2.972499000 | –3.044890000 |
| C | –0.385484000 | –2.001510000 | –2.423330000 |
| C | –0.874001000 | –1.154709000 | –1.424910000 |
| C | –2.235521000 | –1.290676000 | –1.089600000 |
| C | –3.055809000 | –2.250675000 | –1.679380000 |
| C | 1.437007000 | –0.179553000 | –1.424910000 |
| C | 2.235519000 | –1.290680000 | –1.089600000 |
| C | 3.477046000 | –1.521071000 | –1.679380000 |
| C | 3.941501000 | –0.627700000 | –2.654270000 |
| C | 3.167452000 | 0.458810000 | –3.044890000 |
| C | 1.926101000 | 0.666916000 | –2.423330000 |
| C | –1.981067000 | 2.513688000 | –3.044890000 |

| | | | |
|---|---|---|---|
| C | –1.540616000 | 1.334594000 | –2.423330000 |
| C | –0.563007000 | 1.334261000 | –1.424910000 |
| C | 0.000002000 | 2.581356000 | –1.089600000 |
| C | –0.421237000 | 3.771746000 | –1.679380000 |
| C | –1.427146000 | 3.727290000 | –2.654270000 |
| C | 0.000000000 | 0.000000000 | –0.834600000 |
| C | 0.166651000 | 5.112214000 | –1.273220000 |
| C | –0.710464000 | 5.767355000 | –0.201640000 |
| C | 1.610759000 | 5.016592000 | –0.786940000 |
| C | –2.977939000 | 2.438950000 | –4.188210000 |
| C | –4.226402000 | 1.629299000 | –3.830860000 |
| C | –2.302641000 | 1.861989000 | –5.438220000 |
| C | –0.166651000 | –5.112214000 | 1.273220000 |
| C | –1.610759000 | –5.016592000 | 0.786940000 |
| C | 0.710464000 | –5.767355000 | 0.201640000 |
| C | 2.977939000 | –2.438950000 | 4.188210000 |
| C | 4.226402000 | –1.629299000 | 3.830860000 |
| C | 2.302641000 | –1.861989000 | 5.438220000 |
| C | 4.343982000 | –2.700431000 | –1.273220000 |
| C | 3.539116000 | –3.903254000 | –0.786940000 |
| C | 5.349908000 | –2.268397000 | –0.201640000 |
| C | 3.601162000 | 1.359496000 | –4.188210000 |
| C | 3.524215000 | 2.845522000 | –3.830860000 |
| C | 2.763850000 | 1.063151000 | –5.438220000 |
| C | –3.601162000 | –1.359496000 | 4.188210000 |
| C | –2.763850000 | –1.063151000 | 5.438220000 |
| C | –3.524215000 | –2.845522000 | 3.830860000 |
| C | –4.343982000 | 2.700431000 | 1.273220000 |
| C | –5.349908000 | 2.268397000 | 0.201640000 |
| C | –3.539116000 | 3.903254000 | 0.786940000 |
| C | 4.510633000 | 2.411783000 | 1.273220000 |
| C | 5.149876000 | 1.113337000 | 0.786940000 |
| C | 4.639444000 | 3.498957000 | 0.201640000 |
| C | 0.623223000 | 3.798446000 | 4.188210000 |
| C | 0.461209000 | 2.925140000 | 5.438220000 |
| C | –0.702187000 | 4.474820000 | 3.830860000 |
| C | –0.623223000 | –3.798446000 | –4.188210000 |
| C | –0.461209000 | –2.925140000 | –5.438220000 |
| C | 0.702187000 | –4.474820000 | –3.830860000 |
| C | –4.510633000 | –2.411783000 | –1.273220000 |
| C | –4.639444000 | –3.498957000 | –0.201640000 |
| C | –5.149876000 | –1.113337000 | –0.786940000 |
| H | 3.152822000 | 3.848853000 | 3.133090000 |
| H | 2.684879000 | 0.605665000 | 0.379750000 |
| H | –0.638663000 | 1.902996000 | 2.773320000 |
| H | –0.817918000 | –2.628006000 | 0.379750000 |
| H | 1.756794000 | –4.654850000 | 3.133090000 |
| H | 1.967374000 | –0.398399000 | 2.773320000 |
| H | –1.328711000 | –1.504597000 | 2.773320000 |
| H | –1.866961000 | 2.022341000 | 0.379750000 |
| H | –4.909616000 | 0.805997000 | 3.133090000 |
| H | –3.152822000 | –3.848853000 | –3.133090000 |
| H | 0.638663000 | –1.902996000 | –2.773320000 |
| H | –2.684879000 | –0.605665000 | –0.379750000 |
| H | 1.866961000 | –2.022341000 | –0.379750000 |
| H | 4.909616000 | –0.805997000 | –3.133090000 |
| H | 1.328711000 | 1.504597000 | –2.773320000 |
| H | –1.967374000 | 0.398399000 | –2.773320000 |
| H | 0.817918000 | 2.628006000 | –0.379750000 |
| H | –1.756794000 | 4.654850000 | –3.133090000 |
| H | 0.145748000 | 5.753679000 | –2.170290000 |
| H | –0.726756000 | 5.128388000 | 0.695970000 |
| H | –1.747191000 | 5.893014000 | –0.547590000 |
| H | –0.312533000 | 6.754115000 | 0.080790000 |
| H | 1.676051000 | 4.476979000 | 0.172670000 |
| H | 2.025548000 | 6.023145000 | –0.627320000 |
| H | 2.246911000 | 4.491823000 | –1.515190000 |
| H | –3.292128000 | 3.470746000 | –4.414650000 |
| H | –4.725439000 | 2.035985000 | –2.939020000 |
| H | –3.970884000 | 0.575694000 | –3.631450000 |
| H | –4.945275000 | 1.643793000 | –4.663730000 |
| H | –1.424725000 | 2.459453000 | –5.725200000 |
| H | –3.003207000 | 1.840073000 | –6.286770000 |
| H | –1.963633000 | 0.830734000 | –5.247810000 |
| H | –0.145748000 | –5.753679000 | 2.170290000 |
| H | –1.676051000 | –4.476979000 | –0.172670000 |
| H | –2.246911000 | –4.491823000 | 1.515190000 |
| H | –2.025548000 | –6.023145000 | 0.627320000 |
| H | 0.726756000 | –5.128388000 | –0.695970000 |
| H | 0.312533000 | –6.754115000 | –0.080790000 |
| H | 1.747191000 | –5.893014000 | 0.547590000 |
| H | 3.292128000 | –3.470746000 | 4.414650000 |
| H | 4.725439000 | –2.035985000 | 2.939020000 |
| H | 4.945275000 | –1.643793000 | 4.663730000 |
| H | 3.970884000 | –0.575694000 | 3.631450000 |
| H | 1.424725000 | –2.459453000 | 5.725200000 |
| H | 1.963633000 | –0.830734000 | 5.247810000 |
| H | 3.003207000 | –1.840073000 | 6.286770000 |
| H | 4.909958000 | –3.003061000 | –2.170290000 |
| H | 3.039152000 | –3.689992000 | 0.172670000 |
| H | 4.203423000 | –4.765749000 | –0.627320000 |
| H | 2.766577000 | –4.191793000 | –1.515190000 |
| H | 4.804692000 | –1.934805000 | 0.695970000 |
| H | 5.977096000 | –1.433395000 | –0.547590000 |
| H | 6.005502000 | –3.106396000 | 0.080790000 |
| H | 4.651819000 | 1.115694000 | –4.414650000 |
| H | 4.125934000 | 3.074358000 | –2.939020000 |
| H | 2.484008000 | 3.151040000 | –3.631450000 |
| H | 3.896204000 | 3.460837000 | –4.663730000 |
| H | 2.842312000 | 0.004122000 | –5.725200000 |
| H | 3.095153000 | 1.680817000 | –6.286770000 |
| H | 1.701253000 | 1.285189000 | –5.247810000 |
| H | –4.651819000 | –1.115694000 | 4.414650000 |
| H | –2.842312000 | –0.004122000 | 5.725200000 |
| H | –1.701253000 | –1.285189000 | 5.247810000 |
| H | –3.095153000 | –1.680817000 | 6.286770000 |
| H | –4.125934000 | –3.074358000 | 2.939020000 |
| H | –3.896204000 | –3.460837000 | 4.663730000 |
| H | –2.484008000 | –3.151040000 | 3.631450000 |
| H | –4.909958000 | 3.003061000 | 2.170290000 |
| H | –4.804692000 | 1.934805000 | –0.695970000 |
| H | –6.005502000 | 3.106396000 | –0.080790000 |
| H | –5.977096000 | 1.433395000 | 0.547590000 |
| H | –3.039152000 | 3.689992000 | –0.172670000 |
| H | –2.766577000 | 4.191793000 | 1.515190000 |
| H | –4.203423000 | 4.765749000 | 0.627320000 |
| H | 5.055706000 | 2.750618000 | 2.170290000 |
| H | 4.715203000 | 0.786987000 | –0.172670000 |
| H | 5.013488000 | 0.300029000 | 1.515190000 |
| H | 6.228971000 | 1.257397000 | 0.627320000 |
| H | 4.077937000 | 3.193583000 | –0.695970000 |
| H | 5.692969000 | 3.647719000 | –0.080790000 |
| H | 4.229904000 | 4.459619000 | 0.547590000 |
| H | 1.359690000 | 4.586440000 | 4.414650000 |
| H | 1.417586000 | 2.463575000 | 5.725200000 |
| H | –0.262380000 | 2.115923000 | 5.247810000 |
| H | 0.091946000 | 3.520890000 | 6.286770000 |
| H | –0.599505000 | 5.110343000 | 2.939020000 |
| H | –1.049071000 | 5.104630000 | 4.663730000 |
| H | –1.486877000 | 3.726734000 | 3.631450000 |
| H | –1.359690000 | –4.586440000 | –4.414650000 |
| H | –1.417586000 | –2.463575000 | –5.725200000 |
| H | –0.091946000 | –3.520890000 | –6.286770000 |
| H | 0.262380000 | –2.115923000 | –5.247810000 |
| H | 0.599505000 | –5.110343000 | –2.939020000 |
| H | 1.486877000 | –3.726734000 | –3.631450000 |
| H | 1.049071000 | –5.104630000 | –4.663730000 |
| H | –5.055706000 | –2.750618000 | –2.170290000 |
| H | –4.077937000 | –3.193583000 | 0.695970000 |
| H | –4.229904000 | –4.459619000 | –0.547590000 |
| H | –5.692969000 | –3.647719000 | 0.080790000 |
| H | –4.715203000 | –0.786987000 | 0.172670000 |
| H | –6.228971000 | –1.257397000 | –0.627320000 |
| H | –5.013488000 | –0.300029000 | –1.515190000 |

$^{t}$Bu-**1**$_2$

B3LYP

| | | | |
|---|---|---|---|
| C | 1.413840000 | –3.700720000 | 2.790040000 |
| C | 0.507010000 | –3.800810000 | 1.729050000 |
| C | 0.091910000 | –2.613480000 | 1.094730000 |
| C | 0.572880000 | –1.345140000 | 1.467000000 |
| C | 1.476160000 | –1.307230000 | 2.550030000 |
| C | 1.896520000 | –2.458900000 | 3.230190000 |
| C | –1.451365000 | 0.176441000 | 1.467000000 |
| C | –2.309295000 | 1.227144000 | 1.094730000 |
| C | –3.545103000 | 1.461321000 | 1.729050000 |
| C | –3.911838000 | 0.625939000 | 2.790040000 |
| C | –3.077730000 | –0.412984000 | 3.230190000 |
| C | –1.870174000 | –0.624777000 | 2.550030000 |
| C | 1.181210000 | 2.871884000 | 3.230190000 |
| C | 0.394014000 | 1.932007000 | 2.550030000 |
| C | 0.878485000 | 1.168699000 | 1.467000000 |
| C | 2.217385000 | 1.386336000 | 1.094730000 |
| C | 3.038093000 | 2.339489000 | 1.729050000 |
| C | 2.497998000 | 3.074781000 | 2.790040000 |
| C | 0.000000000 | 0.000000000 | 0.854250000 |
| C | –1.413840000 | 3.700720000 | –2.790040000 |
| C | –1.896520000 | 2.458900000 | –3.230190000 |
| C | –1.476160000 | 1.307230000 | –2.550030000 |
| C | –0.572880000 | 1.345140000 | –1.467000000 |
| C | –0.091910000 | 2.613480000 | –1.094730000 |
| C | –0.507010000 | 3.800810000 | –1.729050000 |
| C | –0.878485000 | –1.168699000 | –1.467000000 |
| C | –2.217385000 | –1.386336000 | –1.094730000 |

| | | | |
|---|---|---|---|
| C | –3.038093000 | –2.339489000 | –1.729050000 |
| C | –2.497998000 | –3.074781000 | –2.790040000 |
| C | –1.181210000 | –2.871884000 | –3.230190000 |
| C | –0.394014000 | –1.932007000 | –2.550030000 |
| C | 3.077730000 | 0.412984000 | –3.230190000 |
| C | 1.870174000 | 0.624777000 | –2.550030000 |
| C | 1.451365000 | –0.176441000 | –1.467000000 |
| C | 2.309295000 | –1.227144000 | –1.094730000 |
| C | 3.545103000 | –1.461321000 | –1.729050000 |
| C | 3.911838000 | –0.625939000 | –2.790040000 |
| C | 0.000000000 | 0.000000000 | –0.854250000 |
| C | –0.090610000 | –5.160740000 | 1.301970000 |
| C | 2.832240000 | –2.401940000 | 4.457470000 |
| C | –4.424027000 | 2.658841000 | 1.301970000 |
| C | –3.496261000 | –1.251822000 | 4.457470000 |
| C | 0.664021000 | 3.653762000 | 4.457470000 |
| C | 4.514637000 | 2.501899000 | 1.301970000 |
| C | –2.832240000 | 2.401940000 | –4.457470000 |
| C | 0.090610000 | 5.160740000 | –1.301970000 |
| C | –4.514637000 | –2.501899000 | –1.301970000 |
| C | –0.664021000 | –3.653762000 | –4.457470000 |
| C | 3.496261000 | 1.251822000 | –4.457470000 |
| C | 4.424027000 | –2.658841000 | –1.301970000 |
| C | 0.634270000 | –6.352450000 | 1.957220000 |
| C | 0.000000000 | –5.338700000 | –0.228330000 |
| C | –1.575270000 | –5.208150000 | 1.730330000 |
| C | 3.254780000 | –0.965050000 | 4.811560000 |
| C | 4.111340000 | –3.226120000 | 4.183310000 |
| C | 2.097820000 | –2.998030000 | 5.681910000 |
| C | –5.818518000 | 2.626931000 | 1.957220000 |
| C | –4.623450000 | 2.669350000 | –0.228330000 |
| C | –3.722755000 | 3.968299000 | 1.730330000 |
| C | –2.463148000 | –2.336197000 | 4.811560000 |
| C | –4.849572000 | –1.947465000 | 4.183310000 |
| C | –3.645280000 | –0.317750000 | 5.681910000 |
| C | –0.791632000 | 3.301247000 | 4.811560000 |
| C | 0.738232000 | 5.173585000 | 4.183310000 |
| C | 1.547460000 | 3.315780000 | 5.681910000 |
| C | 5.184248000 | 3.725519000 | 1.957220000 |
| C | 4.623450000 | 2.669350000 | –0.228330000 |
| C | 5.298025000 | 1.239851000 | 1.730330000 |
| C | –2.097820000 | 2.998030000 | –5.681910000 |
| C | –4.111340000 | 3.226120000 | –4.183310000 |
| C | –3.254780000 | 0.965050000 | –4.811560000 |
| C | 0.000000000 | 5.338700000 | 0.228330000 |
| C | –0.634270000 | 6.352450000 | –1.957220000 |
| C | 1.575270000 | 5.208150000 | –1.730330000 |
| C | –4.623450000 | –2.669350000 | 0.228330000 |
| C | –5.184248000 | –3.725519000 | –1.957220000 |
| C | –5.298025000 | –1.239851000 | –1.730330000 |
| C | –1.547460000 | –3.315780000 | –5.681910000 |
| C | –0.738232000 | –5.173585000 | –4.183310000 |
| C | 0.791632000 | –3.301247000 | –4.811560000 |
| C | 3.645280000 | 0.317750000 | –5.681910000 |
| C | 4.849572000 | 1.947465000 | –4.183310000 |
| C | 2.463148000 | 2.336197000 | –4.811560000 |
| C | 4.623450000 | –2.669350000 | 0.228330000 |
| C | 5.818518000 | –2.626931000 | –1.957220000 |
| C | 3.722755000 | –3.968299000 | –1.730330000 |
| H | 0.526730000 | –6.354820000 | 3.052970000 |
| H | 1.709570000 | –6.360860000 | 1.717340000 |
| H | 0.201800000 | –7.295110000 | 1.585810000 |
| H | –0.518870000 | –4.538830000 | –0.775000000 |
| H | –0.458010000 | –6.296420000 | –0.526480000 |
| H | 1.050210000 | –5.350820000 | –0.560780000 |
| H | –1.672110000 | –5.124880000 | 2.824930000 |
| H | –2.037500000 | –6.160470000 | 1.419590000 |
| H | –2.150560000 | –4.387410000 | 1.276620000 |
| H | 3.920810000 | –0.980560000 | 5.689050000 |
| H | 2.389560000 | –0.331920000 | 5.063090000 |
| H | 3.800590000 | –0.482120000 | 3.987040000 |
| H | 4.770630000 | –3.214560000 | 5.067410000 |
| H | 4.675180000 | –2.810220000 | 3.333290000 |
| H | 3.881430000 | –4.277980000 | 3.953370000 |
| H | 2.747110000 | –2.963620000 | 6.572790000 |
| H | 1.809290000 | –4.047950000 | 5.519140000 |
| H | 1.181380000 | –2.428180000 | 5.906320000 |
| H | –5.766801000 | 2.721248000 | 3.052970000 |
| H | –6.363451000 | 1.699899000 | 1.717340000 |
| H | –6.418651000 | 3.472791000 | 1.585810000 |
| H | –3.671307000 | 2.718770000 | –0.775000000 |
| H | –5.223855000 | 3.544858000 | –0.526480000 |
| H | –5.159051000 | 1.765901000 | –0.560780000 |
| H | –3.602221000 | 4.010530000 | 2.824930000 |
| H | –4.316374000 | 4.844762000 | 1.419590000 |
| H | –2.724329000 | 4.056145000 | 1.276620000 |
| H | –2.809595000 | –2.905241000 | 5.689050000 |
| H | –1.482231000 | –1.903460000 | 5.063090000 |
| H | –2.317823000 | –3.050347000 | 3.987040000 |
| H | –5.169206000 | –2.524207000 | 5.067410000 |
| H | –4.771312000 | –2.643715000 | 3.333290000 |
| H | –5.645554000 | –1.222427000 | 3.953370000 |
| H | –3.940125000 | –0.897257000 | 6.572790000 |
| H | –4.410273000 | 0.457084000 | 5.519140000 |
| H | –2.693556000 | 0.190985000 | 5.906320000 |
| H | –1.111215000 | 3.885801000 | 5.689050000 |
| H | –0.907329000 | 2.235380000 | 5.063090000 |
| H | –1.482767000 | 3.532467000 | 3.987040000 |
| H | 0.398576000 | 5.738767000 | 5.067410000 |
| H | 0.096132000 | 5.453935000 | 3.333290000 |
| H | 1.764124000 | 5.500407000 | 3.953370000 |
| H | 1.193015000 | 3.860877000 | 6.572790000 |
| H | 2.600983000 | 3.590866000 | 5.519140000 |
| H | 1.512176000 | 2.237195000 | 5.906320000 |
| H | 5.240071000 | 3.633572000 | 3.052970000 |
| H | 4.653881000 | 4.660961000 | 1.717340000 |
| H | 6.216851000 | 3.822319000 | 1.585810000 |
| H | 4.190177000 | 1.820060000 | –0.775000000 |
| H | 5.681865000 | 2.751562000 | –0.526480000 |
| H | 4.108841000 | 3.584919000 | –0.560780000 |
| H | 5.274331000 | 1.114350000 | 2.824930000 |
| H | 6.353874000 | 1.315708000 | 1.419590000 |
| H | 4.874889000 | 0.331265000 | 1.276620000 |
| H | –1.809290000 | 4.047950000 | –5.519140000 |
| H | –2.747110000 | 2.963620000 | –6.572790000 |
| H | –1.181380000 | 2.428180000 | –5.906320000 |
| H | –3.881430000 | 4.277980000 | –3.953370000 |
| H | –4.675180000 | 2.810220000 | –3.333290000 |
| H | –4.770630000 | 3.214560000 | –5.067410000 |
| H | –3.800590000 | 0.482120000 | –3.987040000 |
| H | –2.389560000 | 0.331920000 | –5.063090000 |
| H | –3.920810000 | 0.980560000 | –5.689050000 |
| H | 0.458010000 | 6.296420000 | 0.526480000 |
| H | 0.518870000 | 4.538830000 | 0.775000000 |
| H | –1.050210000 | 5.350820000 | 0.560780000 |
| H | –0.201800000 | 7.295110000 | –1.585810000 |
| H | –1.709570000 | 6.360860000 | –1.717340000 |
| H | –0.526730000 | 6.354820000 | –3.052970000 |
| H | 2.037500000 | 6.160470000 | –1.419590000 |
| H | 1.672110000 | 5.124880000 | –2.824930000 |
| H | 2.150560000 | 4.387410000 | –1.276620000 |
| H | –5.681865000 | –2.751562000 | 0.526480000 |
| H | –4.190177000 | –1.820060000 | 0.775000000 |
| H | –4.108841000 | –3.584919000 | 0.560780000 |
| H | –6.216851000 | –3.822319000 | –1.585810000 |
| H | –4.653881000 | –4.660961000 | –1.717340000 |
| H | –5.240071000 | –3.633572000 | –3.052970000 |
| H | –6.353874000 | –1.315708000 | –1.419590000 |
| H | –5.274331000 | –1.114350000 | –2.824930000 |
| H | –4.874889000 | –0.331265000 | –1.276620000 |
| H | –2.600983000 | –3.590866000 | –5.519140000 |
| H | –1.193015000 | –3.860877000 | –6.572790000 |
| H | –1.512176000 | –2.237195000 | –5.906320000 |
| H | –1.764124000 | –5.500407000 | –3.953370000 |
| H | –0.096132000 | –5.453935000 | –3.333290000 |
| H | –0.398576000 | –5.738767000 | –5.067410000 |
| H | 1.482767000 | –3.532467000 | –3.987040000 |
| H | 0.907329000 | –2.235380000 | –5.063090000 |
| H | 1.111215000 | –3.885801000 | –5.689050000 |
| H | 4.410273000 | –0.457084000 | –5.519140000 |
| H | 3.940125000 | 0.897257000 | –6.572790000 |
| H | 2.693556000 | –0.190985000 | –5.906320000 |
| H | 5.645554000 | 1.222427000 | –3.953370000 |
| H | 4.771312000 | 2.643715000 | –3.333290000 |
| H | 5.169206000 | 2.524207000 | –5.067410000 |
| H | 2.317823000 | 3.050347000 | –3.987040000 |
| H | 1.482231000 | 1.903460000 | –5.063090000 |
| H | 2.809595000 | 2.905241000 | –5.689050000 |
| H | 5.223855000 | –3.544858000 | 0.526480000 |
| H | 3.671307000 | –2.718770000 | 0.775000000 |
| H | 5.159051000 | –1.765901000 | 0.560780000 |
| H | 6.418651000 | –3.472791000 | –1.585810000 |
| H | 6.363451000 | –1.699899000 | –1.717340000 |
| H | 5.766801000 | –2.721248000 | –3.052970000 |
| H | 4.316374000 | –4.844762000 | –1.419590000 |
| H | 3.602221000 | –4.010530000 | –2.824930000 |
| H | 2.724329000 | –4.056145000 | –1.276620000 |
| H | 1.738610000 | –4.603420000 | 3.304700000 |
| H | –0.645640000 | –2.687570000 | 0.303490000 |
| H | 1.843040000 | –0.348110000 | 2.893940000 |
| H | –2.004684000 | 1.902926000 | 0.303490000 |
| H | –4.855984000 | 0.796030000 | 3.304700000 |
| H | –1.222992000 | –1.422064000 | 2.893940000 |
| H | –0.620048000 | 1.770174000 | 2.893940000 |
| H | 2.650324000 | 0.784644000 | 0.303490000 |
| H | 3.117374000 | 3.807390000 | 3.304700000 |

| | | | |
|---|---|---|---|
| H | –1.738610000 | 4.603420000 | –3.304700000 |
| H | –1.843040000 | 0.348110000 | –2.893940000 |
| H | 0.645640000 | 2.687570000 | –0.303490000 |
| H | –2.650324000 | –0.784644000 | –0.303490000 |
| H | –3.117374000 | –3.807390000 | –3.304700000 |
| H | 0.620048000 | –1.770174000 | –2.893940000 |
| H | 1.222992000 | 1.422064000 | –2.893940000 |
| H | 2.004684000 | –1.902926000 | –0.303490000 |
| H | 4.855984000 | –0.796030000 | –3.304700000 |

B3LYP-D3(BJ)

| | | | |
|---|---|---|---|
| C | 1.911499000 | –3.472015000 | 2.698540000 |
| C | 0.986957000 | –3.676676000 | 1.669860000 |
| C | 0.402744000 | –2.554050000 | 1.063440000 |
| C | 0.739967000 | –1.243947000 | 1.427110000 |
| C | 1.660030000 | –1.089278000 | 2.477180000 |
| C | 2.242159000 | –2.179358000 | 3.131650000 |
| C | –1.447273000 | –0.018857000 | 1.427110000 |
| C | –2.413244000 | 0.928239000 | 1.063440000 |
| C | –3.677573000 | 0.983608000 | 1.669860000 |
| C | –3.962603000 | 0.080600000 | 2.698540000 |
| C | –3.008459000 | –0.852088000 | 3.131650000 |
| C | –1.773358000 | –0.892989000 | 2.477180000 |
| C | 0.766300000 | 3.031446000 | 3.131650000 |
| C | 0.113328000 | 1.982267000 | 2.477180000 |
| C | 0.707306000 | 1.262803000 | 1.427110000 |
| C | 2.010501000 | 1.625812000 | 1.063440000 |
| C | 2.690616000 | 2.693068000 | 1.669860000 |
| C | 2.051103000 | 3.391415000 | 2.698540000 |
| C | 0.000000000 | 0.000000000 | 0.831070000 |
| C | –1.911499000 | 3.472015000 | –2.698540000 |
| C | –2.242159000 | 2.179358000 | –3.131650000 |
| C | –1.660030000 | 1.089278000 | –2.477180000 |
| C | –0.739967000 | 1.243947000 | –1.427110000 |
| C | –0.402744000 | 2.554050000 | –1.063440000 |
| C | –0.986957000 | 3.676676000 | –1.669860000 |
| C | –0.707306000 | –1.262803000 | –1.427110000 |
| C | –2.010501000 | –1.625812000 | –1.063440000 |
| C | –2.690616000 | –2.693068000 | –1.669860000 |
| C | –2.051103000 | –3.391415000 | –2.698540000 |
| C | –0.766300000 | –3.031446000 | –3.131650000 |
| C | –0.113328000 | –1.982267000 | –2.477180000 |
| C | 3.008459000 | 0.852088000 | –3.131650000 |
| C | 1.773358000 | 0.892989000 | –2.477180000 |
| C | 1.447273000 | 0.018857000 | –1.427110000 |
| C | 2.413244000 | –0.928239000 | –1.063440000 |
| C | 3.677573000 | –0.983608000 | –1.669860000 |
| C | 3.962603000 | –0.080600000 | –2.698540000 |
| C | 0.000000000 | 0.000000000 | –0.831070000 |
| C | 0.536089000 | –5.079268000 | 1.229520000 |
| C | 3.191212000 | –1.989189000 | 4.325610000 |
| C | –4.666819000 | 2.075367000 | 1.229520000 |
| C | –3.318295000 | –1.769076000 | 4.325610000 |
| C | 0.127082000 | 3.758266000 | 4.325610000 |
| C | 4.130730000 | 3.003901000 | 1.229520000 |
| C | –3.191212000 | 1.989189000 | –4.325610000 |
| C | –0.536089000 | 5.079268000 | –1.229520000 |
| C | –4.130730000 | –3.003901000 | –1.229520000 |
| C | –0.127082000 | –3.758266000 | –4.325610000 |
| C | 3.318295000 | 1.769076000 | –4.325610000 |
| C | 4.666819000 | –2.075367000 | –1.229520000 |
| C | 1.360340000 | –6.193245000 | 1.891930000 |
| C | 0.668878000 | –5.221130000 | –0.299340000 |
| C | –0.944230000 | –5.260916000 | 1.625360000 |
| C | 3.419728000 | –0.505757000 | 4.656760000 |
| C | 4.558752000 | –2.631990000 | 4.015880000 |
| C | 2.570730000 | –2.670920000 | 5.564200000 |
| C | –6.043678000 | 1.918534000 | 1.891930000 |
| C | –4.856070000 | 2.031299000 | –0.299340000 |
| C | –4.083972000 | 3.448185000 | 1.625360000 |
| C | –2.147862000 | –2.708693000 | 4.656760000 |
| C | –4.558746000 | –2.632000000 | 4.015880000 |
| C | –3.598449000 | –0.890858000 | 5.564200000 |
| C | –1.271866000 | 3.214450000 | 4.656760000 |
| C | –0.000006000 | 5.263990000 | 4.015880000 |
| C | 1.027719000 | 3.561777000 | 5.564200000 |
| C | 4.683338000 | 4.274712000 | 1.891930000 |
| C | 4.187192000 | 3.189831000 | –0.299340000 |
| C | 5.028202000 | 1.812731000 | 1.625360000 |
| C | –2.570730000 | 2.670920000 | –5.564200000 |
| C | –4.558752000 | 2.631990000 | –4.015880000 |
| C | –3.419728000 | 0.505757000 | –4.656760000 |
| C | –0.668878000 | 5.221130000 | 0.299340000 |
| C | –1.360340000 | 6.193245000 | –1.891930000 |
| C | 0.944230000 | 5.260916000 | –1.625360000 |
| C | –4.187192000 | –3.189831000 | 0.299340000 |
| C | –4.683338000 | –4.274712000 | –1.891930000 |
| C | –5.028202000 | –1.812731000 | –1.625360000 |
| C | –1.027719000 | –3.561777000 | –5.564200000 |
| C | 0.000006000 | –5.263990000 | –4.015880000 |
| C | 1.271866000 | –3.214450000 | –4.656760000 |
| C | 3.598449000 | 0.890858000 | –5.564200000 |
| C | 4.558746000 | 2.632000000 | –4.015880000 |
| C | 2.147862000 | 2.708693000 | –4.656760000 |
| C | 4.856070000 | –2.031299000 | 0.299340000 |
| C | 6.043678000 | –1.918534000 | –1.891930000 |
| C | 4.083972000 | –3.448185000 | –1.625360000 |
| H | 1.244975000 | –6.195168000 | 2.986770000 |
| H | 2.432434000 | –6.096712000 | 1.658870000 |
| H | 1.022202000 | –7.173643000 | 1.521880000 |
| H | 0.098757000 | –4.452471000 | –0.838000000 |
| H | 0.294738000 | –6.205387000 | –0.624830000 |
| H | 1.721935000 | –5.139586000 | –0.610170000 |
| H | –1.067307000 | –5.202074000 | 2.718390000 |
| H | –1.317893000 | –6.242667000 | 1.290290000 |
| H | –1.575105000 | –4.483138000 | 1.171750000 |
| H | 4.113164000 | –0.419537000 | 5.507700000 |
| H | 2.483104000 | 0.002076000 | 4.934840000 |
| H | 3.859202000 | 0.038064000 | 3.807760000 |
| H | 5.229127000 | –2.542830000 | 4.886470000 |
| H | 5.039292000 | –2.133758000 | 3.160380000 |
| H | 4.461671000 | –3.701215000 | 3.773710000 |
| H | 3.219500000 | –2.533117000 | 6.444930000 |
| H | 2.437096000 | –3.752362000 | 5.409400000 |
| H | 1.583405000 | –2.237874000 | 5.792320000 |
| H | –5.987661000 | 2.019404000 | 2.986770000 |
| H | –6.496124000 | 0.941807000 | 1.658870000 |
| H | –6.723658000 | 2.701569000 | 1.521880000 |
| H | –3.905332000 | 2.140709000 | –0.838000000 |
| H | –5.521392000 | 2.847443000 | –0.624830000 |
| H | –5.311980000 | 1.078553000 | –0.610170000 |
| H | –3.971475000 | 3.525352000 | 2.718390000 |
| H | –4.747361000 | 4.262663000 | 1.290290000 |
| H | –3.094959000 | 3.605650000 | 1.171750000 |
| H | –2.419911000 | –3.352336000 | 5.507700000 |
| H | –1.239754000 | –2.151469000 | 4.934840000 |
| H | –1.896636000 | –3.361199000 | 3.807760000 |
| H | –4.816719000 | –3.257142000 | 4.886470000 |
| H | –4.367535000 | –3.297276000 | 3.160380000 |
| H | –5.436182000 | –2.013313000 | 3.773710000 |
| H | –3.803494000 | –1.521610000 | 6.444930000 |
| H | –4.468189000 | –0.234406000 | 5.409400000 |
| H | –2.729758000 | –0.252332000 | 5.792320000 |
| H | –1.693253000 | 3.771873000 | 5.507700000 |
| H | –1.243349000 | 2.149393000 | 4.934840000 |
| H | –1.962565000 | 3.323135000 | 3.807760000 |
| H | –0.412408000 | 5.799972000 | 4.886470000 |
| H | –0.671757000 | 5.431034000 | 3.160380000 |
| H | 0.974511000 | 5.714528000 | 3.773710000 |
| H | 0.583994000 | 4.054727000 | 6.444930000 |
| H | 2.031093000 | 3.986768000 | 5.409400000 |
| H | 1.146353000 | 2.490206000 | 5.792320000 |
| H | 4.742685000 | 4.175764000 | 2.986770000 |
| H | 4.063691000 | 5.154905000 | 1.658870000 |
| H | 5.701456000 | 4.472075000 | 1.521880000 |
| H | 3.806575000 | 2.311762000 | –0.838000000 |
| H | 5.226654000 | 3.357944000 | –0.624830000 |
| H | 3.590044000 | 4.061033000 | –0.610170000 |
| H | 5.038782000 | 1.676722000 | 2.718390000 |
| H | 6.065255000 | 1.980004000 | 1.290290000 |
| H | 4.670064000 | 0.877488000 | 1.171750000 |
| H | –2.437096000 | 3.752362000 | –5.409400000 |
| H | –3.219500000 | 2.533117000 | –6.444930000 |
| H | –1.583405000 | 2.237874000 | –5.792320000 |
| H | –4.461671000 | 3.701215000 | –3.773710000 |
| H | –5.039292000 | 2.133758000 | –3.160380000 |
| H | –5.229127000 | 2.542830000 | –4.886470000 |
| H | –3.859202000 | –0.038064000 | –3.807760000 |
| H | –2.483104000 | –0.002076000 | –4.934840000 |
| H | –4.113164000 | 0.419537000 | –5.507700000 |
| H | –0.294738000 | 6.205387000 | 0.624830000 |
| H | –0.098757000 | 4.452471000 | 0.838000000 |
| H | –1.721935000 | 5.139586000 | 0.610170000 |
| H | –1.022202000 | 7.173643000 | –1.521880000 |
| H | –2.432434000 | 6.096712000 | –1.658870000 |
| H | –1.244975000 | 6.195168000 | –2.986770000 |
| H | 1.317893000 | 6.242667000 | –1.290290000 |
| H | 1.067307000 | 5.202074000 | –2.718390000 |
| H | 1.575105000 | 4.483138000 | –1.171750000 |
| H | –5.226654000 | –3.357944000 | 0.624830000 |
| H | –3.806575000 | –2.311762000 | 0.838000000 |
| H | –3.590044000 | –4.061033000 | 0.610170000 |
| H | –5.701456000 | –4.472075000 | –1.521880000 |
| H | –4.063691000 | –5.154905000 | –1.658870000 |
| H | –4.742685000 | –4.175764000 | –2.986770000 |

| | | | |
|---|---|---|---|
| H | –6.065255000 | –1.980004000 | –1.290290000 |
| H | –5.038782000 | –1.676722000 | –2.718390000 |
| H | –4.670064000 | –0.877488000 | –1.171750000 |
| H | –2.031093000 | –3.986768000 | –5.409400000 |
| H | –0.583994000 | –4.054727000 | –6.444930000 |
| H | –1.146353000 | –2.490206000 | –5.792320000 |
| H | –0.974511000 | –5.714528000 | –3.773710000 |
| H | 0.671757000 | –5.431034000 | –3.160380000 |
| H | 0.412408000 | –5.799972000 | –4.886470000 |
| H | 1.962565000 | –3.323135000 | –3.807760000 |
| H | 1.243349000 | –2.149393000 | –4.934840000 |
| H | 1.693253000 | –3.771873000 | –5.507700000 |
| H | 4.468189000 | 0.234406000 | –5.409400000 |
| H | 3.803494000 | 1.521610000 | –6.444930000 |
| H | 2.729758000 | 0.252332000 | –5.792320000 |
| H | 5.436182000 | 2.013313000 | –3.773710000 |
| H | 4.367535000 | 3.297276000 | –3.160380000 |
| H | 4.816719000 | 3.257142000 | –4.886470000 |
| H | 1.896636000 | 3.361199000 | –3.807760000 |
| H | 1.239754000 | 2.151469000 | –4.934840000 |
| H | 2.419911000 | 3.352336000 | –5.507700000 |
| H | 5.521392000 | –2.847443000 | 0.624830000 |
| H | 3.905332000 | –2.140709000 | 0.838000000 |
| H | 5.311980000 | –1.078553000 | 0.610170000 |
| H | 6.723658000 | –2.701569000 | –1.521880000 |
| H | 6.496124000 | –0.941807000 | –1.658870000 |
| H | 5.987661000 | –2.019404000 | –2.986770000 |
| H | 4.747361000 | –4.262663000 | –1.290290000 |
| H | 3.971475000 | –3.525352000 | –2.718390000 |
| H | 3.094959000 | –3.605650000 | –1.171750000 |
| H | 2.365756000 | –4.328560000 | 3.192610000 |
| H | –0.351453000 | –2.715799000 | 0.304750000 |
| H | 1.913196000 | –0.092396000 | 2.810390000 |
| H | –2.176224000 | 1.662267000 | 0.304750000 |
| H | –4.931521000 | 0.115476000 | 3.192610000 |
| H | –1.036615000 | –1.610678000 | 2.810390000 |
| H | –0.876581000 | 1.703074000 | 2.810390000 |
| H | 2.527677000 | 1.053532000 | 0.304750000 |
| H | 2.565766000 | 4.213085000 | 3.192610000 |
| H | –2.365756000 | 4.328560000 | –3.192610000 |
| H | –1.913196000 | 0.092396000 | –2.810390000 |
| H | 0.351453000 | 2.715799000 | –0.304750000 |
| H | –2.527677000 | –1.053532000 | –0.304750000 |
| H | –2.565766000 | –4.213085000 | –3.192610000 |
| H | 0.876581000 | –1.703074000 | –2.810390000 |
| H | 1.036615000 | 1.610678000 | –2.810390000 |
| H | 2.176224000 | –1.662267000 | –0.304750000 |
| H | 4.931521000 | –0.115476000 | –3.192610000 |

M06-2X

| | | | |
|---|---|---|---|
| C | 1.522580000 | –3.663230000 | 2.698180000 |
| C | 0.567670000 | –3.757410000 | 1.684590000 |
| C | 0.112340000 | –2.576150000 | 1.082920000 |
| C | 0.604320000 | –1.313650000 | 1.433370000 |
| C | 1.542700000 | –1.268040000 | 2.477340000 |
| C | 1.999740000 | –2.418510000 | 3.126800000 |
| C | –1.439814000 | 0.133469000 | 1.433370000 |
| C | –2.287181000 | 1.190786000 | 1.082920000 |
| C | –3.537848000 | 1.387088000 | 1.684590000 |
| C | –3.933740000 | 0.513022000 | 2.698180000 |
| C | –3.094361000 | –0.522571000 | 3.126800000 |
| C | –1.869505000 | –0.701997000 | 2.477340000 |
| C | 1.094621000 | 2.941081000 | 3.126800000 |
| C | 0.326805000 | 1.970037000 | 2.477340000 |
| C | 0.835494000 | 1.180181000 | 1.433370000 |
| C | 2.174841000 | 1.385364000 | 1.082920000 |
| C | 2.970178000 | 2.370322000 | 1.684590000 |
| C | 2.411160000 | 3.150208000 | 2.698180000 |
| C | 0.000000000 | 0.000000000 | 0.832810000 |
| C | –1.522580000 | 3.663230000 | –2.698180000 |
| C | –1.999740000 | 2.418510000 | –3.126800000 |
| C | –1.542700000 | 1.268040000 | –2.477340000 |
| C | –0.604320000 | 1.313650000 | –1.433370000 |
| C | –0.112340000 | 2.576150000 | –1.082920000 |
| C | –0.567670000 | 3.757410000 | –1.684590000 |
| C | –0.835494000 | –1.180181000 | –1.433370000 |
| C | –2.174841000 | –1.385364000 | –1.082920000 |
| C | –2.970178000 | –2.370322000 | –1.684590000 |
| C | –2.411160000 | –3.150208000 | –2.698180000 |
| C | –1.094621000 | –2.941081000 | –3.126800000 |
| C | –0.326805000 | –1.970037000 | –2.477340000 |
| C | 3.094361000 | 0.522571000 | –3.126800000 |
| C | 1.869505000 | 0.701997000 | –2.477340000 |
| C | 1.439814000 | –0.133469000 | –1.433370000 |
| C | 2.287181000 | –1.190786000 | –1.082920000 |
| C | 3.537848000 | –1.387088000 | –1.684590000 |
| C | 3.933740000 | –0.513022000 | –2.698180000 |
| C | 0.000000000 | 0.000000000 | –0.832810000 |
| C | –0.064290000 | –5.089930000 | 1.264800000 |
| C | 2.987290000 | –2.345200000 | 4.297520000 |
| C | –4.375864000 | 2.600642000 | 1.264800000 |
| C | –3.524648000 | –1.414469000 | 4.297520000 |
| C | 0.537358000 | 3.759669000 | 4.297520000 |
| C | 4.440154000 | 2.489288000 | 1.264800000 |
| C | –2.987290000 | 2.345200000 | –4.297520000 |
| C | 0.064290000 | 5.089930000 | –1.264800000 |
| C | –4.440154000 | –2.489288000 | –1.264800000 |
| C | –0.537358000 | –3.759669000 | –4.297520000 |
| C | 3.524648000 | 1.414469000 | –4.297520000 |
| C | 4.375864000 | –2.600642000 | –1.264800000 |
| C | 0.637110000 | –6.290550000 | 1.905020000 |
| C | 0.000000000 | –5.248730000 | –0.260990000 |
| C | –1.536210000 | –5.086170000 | 1.707860000 |
| C | 3.305200000 | –0.899990000 | 4.692290000 |
| C | 4.300090000 | –3.041940000 | 3.906130000 |
| C | 2.379250000 | –3.053910000 | 5.519170000 |
| C | –5.766331000 | 2.593522000 | 1.905020000 |
| C | –4.545534000 | 2.624365000 | –0.260990000 |
| C | –3.636647000 | 3.873482000 | 1.707860000 |
| C | –2.432014000 | –2.412392000 | 4.692290000 |
| C | –4.784442000 | –2.203017000 | 3.906130000 |
| C | –3.834389000 | –0.533536000 | 5.519170000 |
| C | –0.873186000 | 3.312382000 | 4.692290000 |
| C | 0.484352000 | 5.244957000 | 3.906130000 |
| C | 1.455139000 | 3.587446000 | 5.519170000 |
| C | 5.129221000 | 3.697028000 | 1.905020000 |
| C | 4.545534000 | 2.624365000 | –0.260990000 |
| C | 5.172857000 | 1.212688000 | 1.707860000 |
| C | –2.379250000 | 3.053910000 | –5.519170000 |
| C | –4.300090000 | 3.041940000 | –3.906130000 |
| C | –3.305200000 | 0.899990000 | –4.692290000 |
| C | 0.000000000 | 5.248730000 | 0.260990000 |
| C | –0.637110000 | 6.290550000 | –1.905020000 |
| C | 1.536210000 | 5.086170000 | –1.707860000 |
| C | –4.545534000 | –2.624365000 | 0.260990000 |
| C | –5.129221000 | –3.697028000 | –1.905020000 |
| C | –5.172857000 | –1.212688000 | –1.707860000 |
| C | –1.455139000 | –3.587446000 | –5.519170000 |
| C | –0.484352000 | –5.244957000 | –3.906130000 |
| C | 0.873186000 | –3.312382000 | –4.692290000 |
| C | 3.834389000 | 0.533536000 | –5.519170000 |
| C | 4.784442000 | 2.203017000 | –3.906130000 |
| C | 2.432014000 | 2.412392000 | –4.692290000 |
| C | 4.545534000 | –2.624365000 | 0.260990000 |
| C | 5.766331000 | –2.593522000 | –1.905020000 |
| C | 3.636647000 | –3.873482000 | –1.707860000 |
| H | 0.553940000 | –6.274330000 | 3.001840000 |
| H | 1.704580000 | –6.321830000 | 1.638130000 |
| H | 0.170670000 | –7.221350000 | 1.549290000 |
| H | –0.495810000 | –4.418210000 | –0.786460000 |
| H | –0.497660000 | –6.183670000 | –0.564480000 |
| H | 1.046390000 | –5.295770000 | –0.600980000 |
| H | –1.610650000 | –5.041630000 | 2.805320000 |
| H | –2.043560000 | –6.002120000 | 1.365120000 |
| H | –2.069530000 | –4.217640000 | 1.291360000 |
| H | 4.006000000 | –0.895630000 | 5.540490000 |
| H | 2.397960000 | –0.355500000 | 4.996630000 |
| H | 3.773000000 | –0.348430000 | 3.863560000 |
| H | 4.996780000 | –3.044560000 | 4.759380000 |
| H | 4.782800000 | –2.516410000 | 3.068290000 |
| H | 4.127890000 | –4.085500000 | 3.602080000 |
| H | 3.070600000 | –2.993380000 | 6.374050000 |
| H | 2.184280000 | –4.117330000 | 5.318340000 |
| H | 1.428350000 | –2.581110000 | 5.808750000 |
| H | –5.710699000 | 2.657439000 | 3.001840000 |
| H | –6.327155000 | 1.684705000 | 1.638130000 |
| H | –6.339208000 | 3.462870000 | 1.549290000 |
| H | –3.578377000 | 2.638489000 | –0.786460000 |
| H | –5.106385000 | 3.522821000 | –0.564480000 |
| H | –5.109466000 | 1.741685000 | –0.600980000 |
| H | –3.560855000 | 3.915679000 | 2.805320000 |
| H | –4.176208000 | 4.770835000 | 1.365120000 |
| H | –2.617818000 | 3.901086000 | 1.291360000 |
| H | –2.778638000 | –3.021483000 | 5.540490000 |
| H | –1.506852000 | –1.898944000 | 4.996630000 |
| H | –2.188249000 | –3.093299000 | 3.863560000 |
| H | –5.135056000 | –2.805058000 | 4.759380000 |
| H | –4.570675000 | –2.883821000 | 3.068290000 |
| H | –5.602092000 | –1.532108000 | 3.602080000 |
| H | –4.127643000 | –1.162528000 | 6.374050000 |
| H | –4.657852000 | 0.167023000 | 5.318340000 |
| H | –2.949482000 | 0.053568000 | 5.808750000 |
| H | –1.227362000 | 3.917113000 | 5.540490000 |
| H | –0.891108000 | 2.254444000 | 4.996630000 |
| H | –1.584751000 | 3.441729000 | 3.863560000 |

| | | | |
|---|---|---|---|
| H | 0.138276000 | 5.849618000 | 4.759380000 |
| H | –0.212125000 | 5.400231000 | 3.068290000 |
| H | 1.474202000 | 5.617608000 | 3.602080000 |
| H | 1.057043000 | 4.155908000 | 6.374050000 |
| H | 2.473572000 | 3.950307000 | 5.318340000 |
| H | 1.521132000 | 2.527542000 | 5.808750000 |
| H | 5.156759000 | 3.616891000 | 3.001840000 |
| H | 4.622575000 | 4.637125000 | 1.638130000 |
| H | 6.168538000 | 3.758480000 | 1.549290000 |
| H | 4.074187000 | 1.779721000 | –0.786460000 |
| H | 5.604045000 | 2.660849000 | –0.564480000 |
| H | 4.063076000 | 3.554085000 | –0.600980000 |
| H | 5.171505000 | 1.125951000 | 2.805320000 |
| H | 6.219768000 | 1.231285000 | 1.365120000 |
| H | 4.687348000 | 0.316554000 | 1.291360000 |
| H | –2.184280000 | 4.117330000 | –5.318340000 |
| H | –3.070600000 | 2.993380000 | –6.374050000 |
| H | –1.428350000 | 2.581110000 | –5.808750000 |
| H | –4.127890000 | 4.085500000 | –3.602080000 |
| H | –4.782800000 | 2.516410000 | –3.068290000 |
| H | –4.996780000 | 3.044560000 | –4.759380000 |
| H | –3.773000000 | 0.348430000 | –3.863560000 |
| H | –2.397960000 | 0.355500000 | –4.996630000 |
| H | –4.006000000 | 0.895630000 | –5.540490000 |
| H | 0.497660000 | 6.183670000 | 0.564480000 |
| H | 0.495810000 | 4.418210000 | 0.786460000 |
| H | –1.046390000 | 5.295770000 | 0.600980000 |
| H | –0.170670000 | 7.221350000 | –1.549290000 |
| H | –1.704580000 | 6.321830000 | –1.638130000 |
| H | –0.553940000 | 6.274330000 | –3.001840000 |
| H | 2.043560000 | 6.002120000 | –1.365120000 |
| H | 1.610650000 | 5.041630000 | –2.805320000 |
| H | 2.069530000 | 4.217640000 | –1.291360000 |
| H | –5.604045000 | –2.660849000 | 0.564480000 |
| H | –4.074187000 | –1.779721000 | 0.786460000 |
| H | –4.063076000 | –3.554085000 | 0.600980000 |
| H | –6.168538000 | –3.758480000 | –1.549290000 |
| H | –4.622575000 | –4.637125000 | –1.638130000 |
| H | –5.156759000 | –3.616891000 | –3.001840000 |
| H | –6.219768000 | –1.231285000 | –1.365120000 |
| H | –5.171505000 | –1.125951000 | –2.805320000 |
| H | –4.687348000 | –0.316554000 | –1.291360000 |
| H | –2.473572000 | –3.950307000 | –5.318340000 |
| H | –1.057043000 | –4.155908000 | –6.374050000 |
| H | –1.521132000 | –2.527542000 | –5.808750000 |
| H | –1.474202000 | –5.617608000 | –3.602080000 |
| H | 0.212125000 | –5.400231000 | –3.068290000 |
| H | –0.138276000 | –5.849618000 | –4.759380000 |
| H | 1.584751000 | –3.441729000 | –3.863560000 |
| H | 0.891108000 | –2.254444000 | –4.996630000 |
| H | 1.227362000 | –3.917113000 | –5.540490000 |
| H | 4.657852000 | –0.167023000 | –5.318340000 |
| H | 4.127643000 | 1.162528000 | –6.374050000 |
| H | 2.949482000 | –0.053568000 | –5.808750000 |
| H | 5.602092000 | 1.532108000 | –3.602080000 |
| H | 4.570675000 | 2.883821000 | –3.068290000 |
| H | 5.135056000 | 2.805058000 | –4.759380000 |
| H | 2.188249000 | 3.093299000 | –3.863560000 |
| H | 1.506852000 | 1.898944000 | –4.996630000 |
| H | 2.778638000 | 3.021483000 | –5.540490000 |
| H | 5.106385000 | –3.522821000 | 0.564480000 |
| H | 3.578377000 | –2.638489000 | 0.786460000 |
| H | 5.109466000 | –1.741685000 | 0.600980000 |
| H | 6.339208000 | –3.462870000 | –1.549290000 |
| H | 6.327155000 | –1.684705000 | –1.638130000 |
| H | 5.710699000 | –2.657439000 | –3.001840000 |
| H | 4.176208000 | –4.770835000 | –1.365120000 |
| H | 3.560855000 | –3.915679000 | –2.805320000 |
| H | 2.617818000 | –3.901086000 | –1.291360000 |
| H | 1.887550000 | –4.567810000 | 3.184880000 |
| H | –0.680110000 | –2.657400000 | 0.346210000 |
| H | 1.910830000 | –0.308100000 | 2.820440000 |
| H | –1.961321000 | 1.917693000 | 0.346210000 |
| H | –4.899614000 | 0.649239000 | 3.184880000 |
| H | –1.222237000 | –1.500777000 | 2.820440000 |
| H | –0.688593000 | 1.808877000 | 2.820440000 |
| H | 2.641431000 | 0.739707000 | 0.346210000 |
| H | 3.012064000 | 3.918571000 | 3.184880000 |
| H | –1.887550000 | 4.567810000 | –3.184880000 |
| H | –1.910830000 | 0.308100000 | –2.820440000 |
| H | 0.680110000 | 2.657400000 | –0.346210000 |
| H | –2.641431000 | –0.739707000 | –0.346210000 |
| H | –3.012064000 | –3.918571000 | –3.184880000 |
| H | 0.688593000 | –1.808877000 | –2.820440000 |
| H | 1.222237000 | 1.500777000 | –2.820440000 |
| H | 1.961321000 | –1.917693000 | –0.346210000 |
| H | 4.899614000 | –0.649239000 | –3.184880000 |

Cy-**1**$_2$

B3LYP

| | | | |
|---|---|---|---|
| C | 0.000000000 | 0.000000000 | 0.859260000 |
| C | –1.510872000 | 1.397900000 | 2.447540000 |
| C | –1.946663000 | 2.592628000 | 3.046180000 |
| C | –0.526429000 | 1.363075000 | 1.445450000 |
| C | –1.373457000 | 3.799907000 | 2.634090000 |
| C | 0.044194000 | 2.602243000 | 1.089830000 |
| C | –0.366243000 | 3.815848000 | 1.657090000 |
| C | 0.298387000 | 5.116294000 | 1.229690000 |
| C | –0.698614000 | 6.127464000 | 0.620920000 |
| C | 1.091440000 | 5.775870000 | 2.381180000 |
| C | 0.000000000 | 7.418708000 | 0.172050000 |
| C | 1.787861000 | 7.067542000 | 1.931050000 |
| C | 0.791588000 | 8.061196000 | 1.319080000 |
| C | 5.226741000 | –5.082104000 | 1.931050000 |
| C | 4.456331000 | –3.833150000 | 2.381180000 |
| C | 6.585407000 | –4.716133000 | 1.319080000 |
| C | 3.977545000 | –0.710505000 | 2.634090000 |
| C | 4.281648000 | –2.816558000 | 1.229690000 |
| C | 3.218613000 | 0.389546000 | 3.046180000 |
| C | 3.487743000 | –1.590748000 | 1.657090000 |
| C | 1.966053000 | 0.609503000 | 2.447540000 |
| C | 2.231511000 | –1.339395000 | 1.089830000 |
| C | 1.443672000 | –0.225637000 | 1.445450000 |
| C | 6.424789000 | –3.709354000 | 0.172050000 |
| C | 5.655846000 | –2.458715000 | 0.620920000 |
| C | –6.424789000 | –3.709354000 | 0.172050000 |
| C | –4.957233000 | –3.668749000 | 0.620920000 |
| C | –7.376994000 | –3.345063000 | 1.319080000 |
| C | –2.604088000 | –3.089403000 | 2.634090000 |
| C | –4.580034000 | –2.299737000 | 1.229690000 |
| C | –1.271950000 | –2.982174000 | 3.046180000 |
| C | –7.014602000 | –1.985438000 | 1.931050000 |
| C | –3.121500000 | –2.225100000 | 1.657090000 |
| C | –5.547771000 | –1.942720000 | 2.381180000 |
| C | –0.455181000 | –2.007403000 | 2.447540000 |
| C | –2.275705000 | –1.262848000 | 1.089830000 |
| C | –0.917243000 | –1.137438000 | 1.445450000 |
| C | –0.717282000 | –4.474886000 | 6.617460000 |
| C | –1.334756000 | –3.598961000 | 5.518400000 |
| C | –0.698398000 | –3.875902000 | 4.137690000 |
| C | –0.806891000 | –5.966330000 | 6.267860000 |
| C | –0.178788000 | –6.255092000 | 4.897920000 |
| C | –0.795180000 | –5.379744000 | 3.797410000 |
| C | –4.261405000 | 3.378518000 | 3.797410000 |
| C | –3.007430000 | 2.542781000 | 4.137690000 |
| C | –2.449414000 | 2.955413000 | 5.518400000 |
| C | –5.327675000 | 3.282381000 | 4.897920000 |
| C | –4.763548000 | 3.681953000 | 6.267860000 |
| C | –3.516724000 | 2.858627000 | 6.617460000 |
| C | 3.705828000 | 1.333121000 | 4.137690000 |
| C | 5.056585000 | 2.001226000 | 3.797410000 |
| C | 3.784169000 | 0.643548000 | 5.518400000 |
| C | 5.506462000 | 2.972711000 | 4.897920000 |
| C | 4.234006000 | 1.616259000 | 6.617460000 |
| C | 5.570439000 | 2.284377000 | 6.267860000 |
| C | –1.443672000 | 0.225637000 | –1.445450000 |
| C | 0.000000000 | 0.000000000 | –0.859260000 |
| C | 0.917243000 | 1.137438000 | –1.445450000 |
| C | 2.275705000 | 1.262848000 | –1.089830000 |
| C | 3.121500000 | 2.225100000 | –1.657090000 |
| C | 4.580034000 | 2.299737000 | –1.229690000 |
| C | 7.014602000 | 1.985438000 | –1.931050000 |
| C | 7.376994000 | 3.345063000 | –1.319080000 |
| C | 6.424789000 | 3.709354000 | –0.172050000 |
| C | 5.547771000 | 1.942720000 | –2.381180000 |
| C | 4.957233000 | 3.668749000 | –0.620920000 |
| C | 2.604088000 | 3.089403000 | –2.634090000 |
| C | 1.271950000 | 2.982174000 | –3.046180000 |
| C | 0.698398000 | 3.875902000 | –4.137690000 |
| C | 1.334756000 | 3.598961000 | –5.518400000 |
| C | 0.717282000 | 4.474886000 | –6.617460000 |
| C | 0.806891000 | 5.966330000 | –6.267860000 |
| C | 0.178788000 | 6.255092000 | –4.897920000 |
| C | 0.795180000 | 5.379744000 | –3.797410000 |
| C | 0.455181000 | 2.007403000 | –2.447540000 |
| C | 0.526429000 | –1.363075000 | –1.445450000 |
| C | 1.510872000 | –1.397900000 | –2.447540000 |
| C | 1.946663000 | –2.592628000 | –3.046180000 |
| C | 3.007430000 | –2.542781000 | –4.137690000 |
| C | 5.327675000 | –3.282381000 | –4.897920000 |
| C | 4.763548000 | –3.681953000 | –6.267860000 |
| C | 3.516724000 | –2.858627000 | –6.617460000 |
| C | 4.261405000 | –3.378518000 | –3.797410000 |
| C | 2.449414000 | –2.955413000 | –5.518400000 |

| | | | |
|---|---|---|---|
| C | 1.373457000 | –3.799907000 | –2.634090000 |
| C | 0.366243000 | –3.815848000 | –1.657090000 |
| C | –0.298387000 | –5.116294000 | –1.229690000 |
| C | 0.698614000 | –6.127464000 | –0.620920000 |
| C | 0.000000000 | –7.418708000 | –0.172050000 |
| C | –1.787861000 | –7.067542000 | –1.931050000 |
| C | –0.791588000 | –8.061196000 | –1.319080000 |
| C | –1.091440000 | –5.775870000 | –2.381180000 |
| C | –0.044194000 | –2.602243000 | –1.089830000 |
| C | –1.966053000 | –0.609503000 | –2.447540000 |
| C | –3.218613000 | –0.389546000 | –3.046180000 |
| C | –3.705828000 | –1.333121000 | –4.137690000 |
| C | –5.506462000 | –2.972711000 | –4.897920000 |
| C | –5.570439000 | –2.284377000 | –6.267860000 |
| C | –4.234006000 | –1.616259000 | –6.617460000 |
| C | –5.056585000 | –2.001226000 | –3.797410000 |
| C | –3.784169000 | –0.643548000 | –5.518400000 |
| C | –3.977545000 | 0.710505000 | –2.634090000 |
| C | –3.487743000 | 1.590748000 | –1.657090000 |
| C | –4.281648000 | 2.816558000 | –1.229690000 |
| C | –5.226741000 | 5.082104000 | –1.931050000 |
| C | –6.585407000 | 4.716133000 | –1.319080000 |
| C | –6.424789000 | 3.709354000 | –0.172050000 |
| C | –4.456331000 | 3.833150000 | –2.381180000 |
| C | –5.655846000 | 2.458715000 | –0.620920000 |
| C | –2.231511000 | 1.339395000 | –1.089830000 |
| H | –1.963198000 | 0.474068000 | 2.798440000 |
| H | –1.700058000 | 4.737411000 | 3.090350000 |
| H | 0.846789000 | 2.638658000 | 0.360640000 |
| H | 1.026199000 | 4.862200000 | 0.437930000 |
| H | –1.472126000 | 6.375491000 | 1.371070000 |
| H | –1.225536000 | 5.660817000 | –0.228620000 |
| H | 0.400552000 | 6.005083000 | 3.213680000 |
| H | 1.830594000 | 5.059514000 | 2.778190000 |
| H | 0.690426000 | 7.186485000 | –0.660580000 |
| H | –0.742519000 | 8.130099000 | –0.227920000 |
| H | 2.561572000 | 6.819955000 | 1.180020000 |
| H | 2.316371000 | 7.528251000 | 2.783010000 |
| H | 1.318419000 | 8.962956000 | 0.963360000 |
| H | 0.086694000 | 8.398409000 | 2.102790000 |
| H | 4.625468000 | –5.628364000 | 1.180020000 |
| H | 5.361471000 | –5.770162000 | 2.783010000 |
| H | 5.000279000 | –3.349430000 | 3.213680000 |
| H | 3.466371000 | –4.115098000 | 2.778190000 |
| H | 7.102938000 | –5.623263000 | 0.963360000 |
| H | 7.229889000 | –4.274284000 | 2.102790000 |
| H | 4.952747000 | –0.896412000 | 3.090350000 |
| H | 3.697689000 | –3.319814000 | 0.437930000 |
| H | 1.392154000 | 1.463145000 | 2.798440000 |
| H | 1.861750000 | –2.052670000 | 0.360640000 |
| H | 5.878465000 | –4.191169000 | –0.660580000 |
| H | 7.412132000 | –3.422009000 | –0.227920000 |
| H | 6.257400000 | –1.912847000 | 1.371070000 |
| H | 5.515180000 | –1.769063000 | –0.228620000 |
| H | –6.568891000 | –2.995316000 | –0.660580000 |
| H | –6.669613000 | –4.708090000 | –0.227920000 |
| H | –4.785274000 | –4.462644000 | 1.371070000 |
| H | –4.289643000 | –3.891754000 | –0.228620000 |
| H | –8.421357000 | –3.339694000 | 0.963360000 |
| H | –7.316583000 | –4.124125000 | 2.102790000 |
| H | –3.252689000 | –3.840999000 | 3.090350000 |
| H | –4.723888000 | –1.542386000 | 0.437930000 |
| H | –7.187040000 | –1.191591000 | 1.180020000 |
| H | –7.677843000 | –1.758089000 | 2.783010000 |
| H | –5.400831000 | –2.655653000 | 3.213680000 |
| H | –5.296965000 | –0.944417000 | 2.778190000 |
| H | 0.571044000 | –1.937214000 | 2.798440000 |
| H | –2.708539000 | –0.585988000 | 0.360640000 |
| H | 0.345273000 | –4.195893000 | 6.748980000 |
| H | –1.213408000 | –4.275766000 | 7.582630000 |
| H | –1.222009000 | –2.530769000 | 5.769430000 |
| H | –2.422003000 | –3.793894000 | 5.462740000 |
| H | 0.376185000 | –3.631309000 | 4.222660000 |
| H | –1.870235000 | –6.272652000 | 6.252300000 |
| H | –0.318339000 | –6.573384000 | 7.048940000 |
| H | –0.292792000 | –7.321794000 | 4.640120000 |
| H | 0.909349000 | –6.060972000 | 4.948910000 |
| H | –1.857515000 | –5.656985000 | 3.666540000 |
| H | –0.300259000 | –5.573267000 | 2.830740000 |
| H | –3.970335000 | 4.437148000 | 3.666540000 |
| H | –4.676461000 | 3.046665000 | 2.830740000 |
| H | –3.332899000 | 1.489869000 | 4.222660000 |
| H | –1.580706000 | 2.323675000 | 5.769430000 |
| H | –2.074606000 | 3.994463000 | 5.462740000 |
| H | –6.194464000 | 3.914463000 | 4.640120000 |
| H | –5.703630000 | 2.242967000 | 4.948910000 |
| H | –4.497158000 | 4.755997000 | 6.252300000 |
| H | –5.533548000 | 3.562382000 | 7.048940000 |
| H | –3.806387000 | 1.798931000 | 6.748980000 |
| H | –3.096218000 | 3.188725000 | 7.582630000 |
| H | 2.956714000 | 2.141441000 | 4.222660000 |
| H | 5.827850000 | 1.219837000 | 3.666540000 |
| H | 4.976720000 | 2.526601000 | 2.830740000 |
| H | 2.802714000 | 0.207094000 | 5.769430000 |
| H | 4.496610000 | –0.200570000 | 5.462740000 |
| H | 6.487256000 | 3.407331000 | 4.640120000 |
| H | 4.794281000 | 3.818005000 | 4.948910000 |
| H | 3.461114000 | 2.396962000 | 6.748980000 |
| H | 4.309626000 | 1.087041000 | 7.582630000 |
| H | 6.367393000 | 1.516655000 | 6.252300000 |
| H | 5.851887000 | 3.011002000 | 7.048940000 |
| H | 2.708539000 | 0.585988000 | –0.360640000 |
| H | 4.723888000 | 1.542386000 | –0.437930000 |
| H | 7.187040000 | 1.191591000 | –1.180020000 |
| H | 7.677843000 | 1.758089000 | –2.783010000 |
| H | 8.421357000 | 3.339694000 | –0.963360000 |
| H | 7.316583000 | 4.124125000 | –2.102790000 |
| H | 6.568891000 | 2.995316000 | 0.660580000 |
| H | 6.669613000 | 4.708090000 | 0.227920000 |
| H | 5.400831000 | 2.655653000 | –3.213680000 |
| H | 5.296965000 | 0.944417000 | –2.778190000 |
| H | 4.785274000 | 4.462644000 | –1.371070000 |
| H | 4.289643000 | 3.891754000 | 0.228620000 |
| H | 3.252689000 | 3.840999000 | –3.090350000 |
| H | –0.376185000 | 3.631309000 | –4.222660000 |
| H | 1.222009000 | 2.530769000 | –5.769430000 |
| H | 2.422003000 | 3.793894000 | –5.462740000 |
| H | –0.345273000 | 4.195893000 | –6.748980000 |
| H | 1.213408000 | 4.275766000 | –7.582630000 |
| H | 1.870235000 | 6.272652000 | –6.252300000 |
| H | 0.318339000 | 6.573384000 | –7.048940000 |
| H | 0.292792000 | 7.321794000 | –4.640120000 |
| H | –0.909349000 | 6.060972000 | –4.948910000 |
| H | 1.857515000 | 5.656985000 | –3.666540000 |
| H | 0.300259000 | 5.573267000 | –2.830740000 |
| H | –0.571044000 | 1.937214000 | –2.798440000 |
| H | 1.963198000 | –0.474068000 | –2.798440000 |
| H | 3.332899000 | –1.489869000 | –4.222660000 |
| H | 6.194464000 | –3.914463000 | –4.640120000 |
| H | 5.703630000 | –2.242967000 | –4.948910000 |
| H | 4.497158000 | –4.755997000 | –6.252300000 |
| H | 5.533548000 | –3.562382000 | –7.048940000 |
| H | 3.806387000 | –1.798931000 | –6.748980000 |
| H | 3.096218000 | –3.188725000 | –7.582630000 |
| H | 3.970335000 | –4.437148000 | –3.666540000 |
| H | 4.676461000 | –3.046665000 | –2.830740000 |
| H | 1.580706000 | –2.323675000 | –5.769430000 |
| H | 2.074606000 | –3.994463000 | –5.462740000 |
| H | 1.700058000 | –4.737411000 | –3.090350000 |
| H | –1.026199000 | –4.862200000 | –0.437930000 |
| H | 1.472126000 | –6.375491000 | –1.371070000 |
| H | 1.225536000 | –5.660817000 | 0.228620000 |
| H | –0.690426000 | –7.186485000 | 0.660580000 |
| H | 0.742519000 | –8.130099000 | 0.227920000 |
| H | –2.561572000 | –6.819955000 | –1.180020000 |
| H | –2.316371000 | –7.528251000 | –2.783010000 |
| H | –1.318419000 | –8.962956000 | –0.963360000 |
| H | –0.086694000 | –8.398409000 | –2.102790000 |
| H | –0.400552000 | –6.005083000 | –3.213680000 |
| H | –1.830594000 | –5.059514000 | –2.778190000 |
| H | –0.846789000 | –2.638658000 | –0.360640000 |
| H | –1.392154000 | –1.463145000 | –2.798440000 |
| H | –2.956714000 | –2.141441000 | –4.222660000 |
| H | –6.487256000 | –3.407331000 | –4.640120000 |
| H | –4.794281000 | –3.818005000 | –4.948910000 |
| H | –6.367393000 | –1.516655000 | –6.252300000 |
| H | –5.851887000 | –3.011002000 | –7.048940000 |
| H | –3.461114000 | –2.396962000 | –6.748980000 |
| H | –4.309626000 | –1.087041000 | –7.582630000 |
| H | –5.827850000 | –1.219837000 | –3.666540000 |
| H | –4.976720000 | –2.526601000 | –2.830740000 |
| H | –2.802714000 | –0.207094000 | –5.769430000 |
| H | –4.496610000 | 0.200570000 | –5.462740000 |
| H | –4.952747000 | 0.896412000 | –3.090350000 |
| H | –3.697689000 | 3.319814000 | –0.437930000 |
| H | –4.625468000 | 5.628364000 | –1.180020000 |
| H | –5.361471000 | 5.770162000 | –2.783010000 |
| H | –7.102938000 | 5.623263000 | –0.963360000 |
| H | –7.229889000 | 4.274284000 | –2.102790000 |
| H | –5.878465000 | 4.191169000 | 0.660580000 |
| H | –7.412132000 | 3.422009000 | 0.227920000 |
| H | –5.000279000 | 3.349430000 | –3.213680000 |
| H | –3.466371000 | 4.115098000 | –2.778190000 |
| H | –6.257400000 | 1.912847000 | –1.371070000 |
| H | –5.515180000 | 1.769063000 | 0.228620000 |
| H | –1.861750000 | 2.052670000 | –0.360640000 |

B3LYP-D3(BJ)

| | | | |
|---|---|---|---|
| C | 0.000000000 | 0.000000000 | 0.859260000 |
| C | –1.510872000 | 1.397900000 | 2.447540000 |
| C | –1.946663000 | 2.592628000 | 3.046180000 |
| C | –0.526429000 | 1.363075000 | 1.445450000 |
| C | –1.373457000 | 3.799907000 | 2.634090000 |
| C | 0.044194000 | 2.602243000 | 1.089830000 |
| C | –0.366243000 | 3.815848000 | 1.657090000 |
| C | 0.298387000 | 5.116294000 | 1.229690000 |
| C | –0.698614000 | 6.127464000 | 0.620920000 |
| C | 1.091440000 | 5.775870000 | 2.381180000 |
| C | 0.000000000 | 7.418708000 | 0.172050000 |
| C | 1.787861000 | 7.067542000 | 1.931050000 |
| C | 0.791588000 | 8.061196000 | 1.319080000 |
| C | 5.226741000 | –5.082104000 | 1.931050000 |
| C | 4.456331000 | –3.833150000 | 2.381180000 |
| C | 6.585407000 | –4.716133000 | 1.319080000 |
| C | 3.977545000 | –0.710505000 | 2.634090000 |
| C | 4.281648000 | –2.816558000 | 1.229690000 |
| C | 3.218613000 | 0.389546000 | 3.046180000 |
| C | 3.487743000 | –1.590748000 | 1.657090000 |
| C | 1.966053000 | 0.609503000 | 2.447540000 |
| C | 2.231511000 | –1.339395000 | 1.089830000 |
| C | 1.443672000 | –0.225637000 | 1.445450000 |
| C | 6.424789000 | –3.709354000 | 0.172050000 |
| C | 5.655846000 | –2.458715000 | 0.620920000 |
| C | –6.424789000 | –3.709354000 | 0.172050000 |
| C | –4.957233000 | –3.668749000 | 0.620920000 |
| C | –7.376994000 | –3.345063000 | 1.319080000 |
| C | –2.604088000 | –3.089403000 | 2.634090000 |
| C | –4.580034000 | –2.299737000 | 1.229690000 |
| C | –1.271950000 | –2.982174000 | 3.046180000 |
| C | –7.014602000 | –1.985438000 | 1.931050000 |
| C | –3.121500000 | –2.225100000 | 1.657090000 |
| C | –5.547771000 | –1.942720000 | 2.381180000 |
| C | –0.455181000 | –2.007403000 | 2.447540000 |
| C | –2.275705000 | –1.262848000 | 1.089830000 |
| C | –0.917243000 | –1.137438000 | 1.445450000 |
| C | –0.717282000 | –4.474886000 | 6.617460000 |
| C | –1.334756000 | –3.598961000 | 5.518400000 |
| C | –0.698398000 | –3.875902000 | 4.137690000 |
| C | –0.806891000 | –5.966330000 | 6.267860000 |
| C | –0.178788000 | –6.255092000 | 4.897920000 |
| C | –0.795180000 | –5.379744000 | 3.797410000 |
| C | –4.261405000 | 3.378518000 | 3.797410000 |
| C | –3.007430000 | 2.542781000 | 4.137690000 |
| C | –2.449414000 | 2.955413000 | 5.518400000 |
| C | –5.327675000 | 3.282381000 | 4.897920000 |
| C | –4.763548000 | 3.681953000 | 6.267860000 |
| C | –3.516724000 | 2.858627000 | 6.617460000 |
| C | 3.705828000 | 1.333121000 | 4.137690000 |
| C | 5.056585000 | 2.001226000 | 3.797410000 |
| C | 3.784169000 | 0.643548000 | 5.518400000 |
| C | 5.506462000 | 2.972711000 | 4.897920000 |
| C | 4.234006000 | 1.616259000 | 6.617460000 |
| C | 5.570439000 | 2.284377000 | 6.267860000 |
| C | –1.443672000 | 0.225637000 | –1.445450000 |
| C | 0.000000000 | 0.000000000 | –0.859260000 |
| C | 0.917243000 | 1.137438000 | –1.445450000 |
| C | 2.275705000 | 1.262848000 | –1.089830000 |
| C | 3.121500000 | 2.225100000 | –1.657090000 |
| C | 4.580034000 | 2.299737000 | –1.229690000 |
| C | 7.014602000 | 1.985438000 | –1.931050000 |
| C | 7.376994000 | 3.345063000 | –1.319080000 |
| C | 6.424789000 | 3.709354000 | –0.172050000 |
| C | 5.547771000 | 1.942720000 | –2.381180000 |
| C | 4.957233000 | 3.668749000 | –0.620920000 |
| C | 2.604088000 | 3.089403000 | –2.634090000 |
| C | 1.271950000 | 2.982174000 | –3.046180000 |
| C | 0.698398000 | 3.875902000 | –4.137690000 |
| C | 1.334756000 | 3.598961000 | –5.518400000 |
| C | 0.717282000 | 4.474886000 | –6.617460000 |
| C | 0.806891000 | 5.966330000 | –6.267860000 |
| C | 0.178788000 | 6.255092000 | –4.897920000 |
| C | 0.795180000 | 5.379744000 | –3.797410000 |
| C | 0.455181000 | 2.007403000 | –2.447540000 |
| C | 0.526429000 | –1.363075000 | –1.445450000 |
| C | 1.510872000 | –1.397900000 | –2.447540000 |
| C | 1.946663000 | –2.592628000 | –3.046180000 |
| C | 3.007430000 | –2.542781000 | –4.137690000 |
| C | 5.327675000 | –3.282381000 | –4.897920000 |
| C | 4.763548000 | –3.681953000 | –6.267860000 |
| C | 3.516724000 | –2.858627000 | –6.617460000 |
| C | 4.261405000 | –3.378518000 | –3.797410000 |
| C | 2.449414000 | –2.955413000 | –5.518400000 |
| C | 1.373457000 | –3.799907000 | –2.634090000 |
| C | 0.366243000 | –3.815848000 | –1.657090000 |
| C | –0.298387000 | –5.116294000 | –1.229690000 |
| C | 0.698614000 | –6.127464000 | –0.620920000 |
| C | 0.000000000 | –7.418708000 | –0.172050000 |
| C | –1.787861000 | –7.067542000 | –1.931050000 |
| C | –0.791588000 | –8.061196000 | –1.319080000 |
| C | –1.091440000 | –5.775870000 | –2.381180000 |
| C | –0.044194000 | –2.602243000 | –1.089830000 |
| C | –1.966053000 | –0.609503000 | –2.447540000 |
| C | –3.218613000 | –0.389546000 | –3.046180000 |
| C | –3.705828000 | –1.333121000 | –4.137690000 |
| C | –5.506462000 | –2.972711000 | –4.897920000 |
| C | –5.570439000 | –2.284377000 | –6.267860000 |
| C | –4.234006000 | –1.616259000 | –6.617460000 |
| C | –5.056585000 | –2.001226000 | –3.797410000 |
| C | –3.784169000 | –0.643548000 | –5.518400000 |
| C | –3.977545000 | 0.710505000 | –2.634090000 |
| C | –3.487743000 | 1.590748000 | –1.657090000 |
| C | –4.281648000 | 2.816558000 | –1.229690000 |
| C | –5.226741000 | 5.082104000 | –1.931050000 |
| C | –6.585407000 | 4.716133000 | –1.319080000 |
| C | –6.424789000 | 3.709354000 | –0.172050000 |
| C | –4.456331000 | 3.833150000 | –2.381180000 |
| C | –5.655846000 | 2.458715000 | –0.620920000 |
| C | –2.231511000 | 1.339395000 | –1.089830000 |
| H | –1.963198000 | 0.474068000 | 2.798440000 |
| H | –1.700058000 | 4.737411000 | 3.090350000 |
| H | 0.846789000 | 2.638658000 | 0.360640000 |
| H | 1.026199000 | 4.862200000 | 0.437930000 |
| H | –1.472126000 | 6.375491000 | 1.371070000 |
| H | –1.225536000 | 5.660817000 | –0.228620000 |
| H | 0.400552000 | 6.005083000 | 3.213680000 |
| H | 1.830594000 | 5.059514000 | 2.778190000 |
| H | 0.690426000 | 7.186485000 | –0.660580000 |
| H | –0.742519000 | 8.130099000 | –0.227920000 |
| H | 2.561572000 | 6.819955000 | 1.180020000 |
| H | 2.316371000 | 7.528251000 | 2.783010000 |
| H | 1.318419000 | 8.962956000 | 0.963360000 |
| H | 0.086694000 | 8.398409000 | 2.102790000 |
| H | 4.625468000 | –5.628364000 | 1.180020000 |
| H | 5.361471000 | –5.770162000 | 2.783010000 |
| H | 5.000279000 | –3.349430000 | 3.213680000 |
| H | 3.466371000 | –4.115098000 | 2.778190000 |
| H | 7.102938000 | –5.623263000 | 0.963360000 |
| H | 7.229889000 | –4.274284000 | 2.102790000 |
| H | 4.952747000 | –0.896412000 | 3.090350000 |
| H | 3.697689000 | –3.319814000 | 0.437930000 |
| H | 1.392154000 | 1.463145000 | 2.798440000 |
| H | 1.861750000 | –2.052670000 | 0.360640000 |
| H | 5.878465000 | –4.191169000 | –0.660580000 |
| H | 7.412132000 | –3.422009000 | –0.227920000 |
| H | 6.257400000 | –1.912847000 | 1.371070000 |
| H | 5.515180000 | –1.769063000 | –0.228620000 |
| H | –6.568891000 | –2.995316000 | –0.660580000 |
| H | –6.669613000 | –4.708090000 | –0.227920000 |
| H | –4.785274000 | –4.462644000 | 1.371070000 |
| H | –4.289643000 | –3.891754000 | –0.228620000 |
| H | –8.421357000 | –3.339694000 | 0.963360000 |
| H | –7.316583000 | –4.124125000 | 2.102790000 |
| H | –3.252689000 | –3.840999000 | 3.090350000 |
| H | –4.723888000 | –1.542386000 | 0.437930000 |
| H | –7.187040000 | –1.191591000 | 1.180020000 |
| H | –7.677843000 | –1.758089000 | 2.783010000 |
| H | –5.400831000 | –2.655653000 | 3.213680000 |
| H | –5.296965000 | –0.944417000 | 2.778190000 |
| H | 0.571044000 | –1.937214000 | 2.798440000 |
| H | –2.708539000 | –0.585988000 | 0.360640000 |
| H | 0.345273000 | –4.195893000 | 6.748980000 |
| H | –1.213408000 | –4.275766000 | 7.582630000 |
| H | –1.222009000 | –2.530769000 | 5.769430000 |
| H | –2.422003000 | –3.793894000 | 5.462740000 |
| H | 0.376185000 | –3.631309000 | 4.222660000 |
| H | –1.870235000 | –6.272652000 | 6.252300000 |
| H | –0.318339000 | –6.573384000 | 7.048940000 |
| H | –0.292792000 | –7.321794000 | 4.640120000 |
| H | 0.909349000 | –6.060972000 | 4.948910000 |
| H | –1.857515000 | –5.656985000 | 3.666540000 |
| H | –0.300259000 | –5.573267000 | 2.830740000 |
| H | –3.970335000 | 4.437148000 | 3.666540000 |
| H | –4.676461000 | 3.046665000 | 2.830740000 |
| H | –3.332899000 | 1.489869000 | 4.222660000 |
| H | –1.580706000 | 2.323675000 | 5.769430000 |
| H | –2.074606000 | 3.994463000 | 5.462740000 |
| H | –6.194464000 | 3.914463000 | 4.640120000 |
| H | –5.703630000 | 2.242967000 | 4.948910000 |
| H | –4.497158000 | 4.755997000 | 6.252300000 |
| H | –5.533548000 | 3.562382000 | 7.048940000 |
| H | –3.806387000 | 1.798931000 | 6.748980000 |

| | | | |
|---|---|---|---|
| H | –3.096218000 | 3.188725000 | 7.582630000 |
| H | 2.956714000 | 2.141441000 | 4.222660000 |
| H | 5.827850000 | 1.219837000 | 3.666540000 |
| H | 4.976720000 | 2.526601000 | 2.830740000 |
| H | 2.802714000 | 0.207094000 | 5.769430000 |
| H | 4.496610000 | –0.200570000 | 5.462740000 |
| H | 6.487256000 | 3.407331000 | 4.640120000 |
| H | 4.794281000 | 3.818005000 | 4.948910000 |
| H | 3.461114000 | 2.396962000 | 6.748980000 |
| H | 4.309626000 | 1.087041000 | 7.582630000 |
| H | 6.367393000 | 1.516655000 | 6.252300000 |
| H | 5.851887000 | 3.011002000 | 7.048940000 |
| H | 2.708539000 | 0.585988000 | –0.360640000 |
| H | 4.723888000 | 1.542386000 | –0.437930000 |
| H | 7.187040000 | 1.191591000 | –1.180020000 |
| H | 7.677843000 | 1.758089000 | –2.783010000 |
| H | 8.421357000 | 3.339694000 | –0.963360000 |
| H | 7.316583000 | 4.124125000 | –2.102790000 |
| H | 6.568891000 | 2.995316000 | 0.660580000 |
| H | 6.669613000 | 4.708090000 | 0.227920000 |
| H | 5.400831000 | 2.655653000 | –3.213680000 |
| H | 5.296965000 | 0.944417000 | –2.778190000 |
| H | 4.785274000 | 4.462644000 | –1.371070000 |
| H | 4.289643000 | 3.891754000 | 0.228620000 |
| H | 3.252689000 | 3.840999000 | –3.090350000 |
| H | –0.376185000 | 3.631309000 | –4.222660000 |
| H | 1.222009000 | 2.530769000 | –5.769430000 |
| H | 2.422003000 | 3.793894000 | –5.462740000 |
| H | –0.345273000 | 4.195893000 | –6.748980000 |
| H | 1.213408000 | 4.275766000 | –7.582630000 |
| H | 1.870235000 | 6.272652000 | –6.252300000 |
| H | 0.318339000 | 6.573384000 | –7.048940000 |
| H | 0.292792000 | 7.321794000 | –4.640120000 |
| H | –0.909349000 | 6.060972000 | –4.948910000 |
| H | 1.857515000 | 5.656985000 | –3.666540000 |
| H | 0.300259000 | 5.573267000 | –2.830740000 |
| H | –0.571044000 | 1.937214000 | –2.798440000 |
| H | 1.963198000 | –0.474068000 | –2.798440000 |
| H | 3.332899000 | –1.489869000 | –4.222660000 |
| H | 6.194464000 | –3.914463000 | –4.640120000 |
| H | 5.703630000 | –2.242967000 | –4.948910000 |
| H | 4.497158000 | –4.755997000 | –6.252300000 |
| H | 5.533548000 | –3.562382000 | –7.048940000 |
| H | 3.806387000 | –1.798931000 | –6.748980000 |
| H | 3.096218000 | –3.188725000 | –7.582630000 |
| H | 3.970335000 | –4.437148000 | –3.666540000 |
| H | 4.676461000 | –3.046665000 | –2.830740000 |
| H | 1.580706000 | –2.323675000 | –5.769430000 |
| H | 2.074606000 | –3.994463000 | –5.462740000 |
| H | 1.700058000 | –4.737411000 | –3.090350000 |
| H | –1.026199000 | –4.862200000 | –0.437930000 |
| H | 1.472126000 | –6.375491000 | –1.371070000 |
| H | 1.225536000 | –5.660817000 | 0.228620000 |
| H | –0.690426000 | –7.186485000 | 0.660580000 |
| H | 0.742519000 | –8.130099000 | 0.227920000 |
| H | –2.561572000 | –6.819955000 | –1.180020000 |
| H | –2.316371000 | –7.528251000 | –2.783010000 |
| H | –1.318419000 | –8.962956000 | –0.963360000 |
| H | –0.086694000 | –8.398409000 | –2.102790000 |
| H | –0.400552000 | –6.005083000 | –3.213680000 |
| H | –1.830594000 | –5.059514000 | –2.778190000 |
| H | –0.846789000 | –2.638658000 | –0.360640000 |
| H | –1.392154000 | –1.463145000 | –2.798440000 |
| H | –2.956714000 | –2.141441000 | –4.222660000 |
| H | –6.487256000 | –3.407331000 | –4.640120000 |
| H | –4.794281000 | –3.818005000 | –4.948910000 |
| H | –6.367393000 | –1.516655000 | –6.252300000 |
| H | –5.851887000 | –3.011002000 | –7.048940000 |
| H | –3.461114000 | –2.396962000 | –6.748980000 |
| H | –4.309626000 | –1.087041000 | –7.582630000 |
| H | –5.827850000 | –1.219837000 | –3.666540000 |
| H | –4.976720000 | –2.526601000 | –2.830740000 |
| H | –2.802714000 | –0.207094000 | –5.769430000 |
| H | –4.496610000 | 0.200570000 | –5.462740000 |
| H | –4.952747000 | 0.896412000 | –3.090350000 |
| H | –3.697689000 | 3.319814000 | –0.437930000 |
| H | –4.625468000 | 5.628364000 | –1.180020000 |
| H | –5.361471000 | 5.770162000 | –2.783010000 |
| H | –7.102938000 | 5.623263000 | –0.963360000 |
| H | –7.229889000 | 4.274284000 | –2.102790000 |
| H | –5.878465000 | 4.191169000 | 0.660580000 |
| H | –7.412132000 | 3.422009000 | 0.227920000 |
| H | –5.000279000 | 3.349430000 | –3.213680000 |
| H | –3.466371000 | 4.115098000 | –2.778190000 |
| H | –6.257400000 | 1.912847000 | –1.371070000 |
| H | –5.515180000 | 1.769063000 | 0.228620000 |
| H | –1.861750000 | 2.052670000 | –0.360640000 |

M06-2X

| | | | |
|---|---|---|---|
| C | 0.000000000 | 0.000000000 | 0.850080000 |
| C | –1.826830000 | 1.214070000 | 2.230150000 |
| C | –2.521350000 | 2.356810000 | 2.643850000 |
| C | –0.711010000 | 1.274040000 | 1.385770000 |
| C | –2.063950000 | 3.614290000 | 2.240700000 |
| C | –0.247010000 | 2.559060000 | 1.064000000 |
| C | –0.896940000 | 3.723140000 | 1.478060000 |
| C | –0.246020000 | 5.046610000 | 1.127550000 |
| C | –1.180360000 | 6.257440000 | 1.169860000 |
| C | 0.980370000 | 5.294040000 | 2.024110000 |
| C | –0.447570000 | 7.535550000 | 0.757050000 |
| C | 1.718250000 | 6.566340000 | 1.609460000 |
| C | 0.782820000 | 7.775800000 | 1.633290000 |
| C | 4.827492000 | –4.771218000 | 1.609460000 |
| C | 4.094588000 | –3.496045000 | 2.024110000 |
| C | 6.342630000 | –4.565842000 | 1.633290000 |
| C | 4.162042000 | –0.019712000 | 2.240700000 |
| C | 4.493502000 | –2.310245000 | 1.127550000 |
| C | 3.301732000 | 1.005148000 | 2.643850000 |
| C | 3.672804000 | –1.084797000 | 1.478060000 |
| C | 1.964830000 | 0.975046000 | 2.230150000 |
| C | 2.339716000 | –1.065613000 | 1.064000000 |
| C | 1.458856000 | –0.021267000 | 1.385770000 |
| C | 6.749763000 | –3.380168000 | 0.757050000 |
| C | 6.009282000 | –2.106498000 | 1.169860000 |
| C | –6.302193000 | –4.155382000 | 0.757050000 |
| C | –4.828922000 | –4.150942000 | 1.169860000 |
| C | –7.125450000 | –3.209958000 | 1.633290000 |
| C | –2.098092000 | –3.594578000 | 2.240700000 |
| C | –4.247482000 | –2.736365000 | 1.127550000 |
| C | –0.780382000 | –3.361958000 | 2.643850000 |
| C | –6.545742000 | –1.795122000 | 1.609460000 |
| C | –2.775864000 | –2.638343000 | 1.478060000 |
| C | –5.074958000 | –1.797995000 | 2.024110000 |
| C | –0.138000000 | –2.189116000 | 2.230150000 |
| C | –2.092706000 | –1.493447000 | 1.064000000 |
| C | –0.747846000 | –1.252773000 | 1.385770000 |
| C | 0.074190000 | –5.969520000 | 5.415740000 |
| C | –0.797970000 | –5.006590000 | 4.608030000 |
| C | 0.000000000 | –4.367440000 | 3.464800000 |
| C | 0.673900000 | –7.054320000 | 4.520080000 |
| C | 1.460860000 | –6.437460000 | 3.363150000 |
| C | 0.594260000 | –5.463750000 | 2.566210000 |
| C | –5.028876000 | 2.217231000 | 2.566210000 |
| C | –3.782314000 | 2.183720000 | 3.464800000 |
| C | –3.936849000 | 3.194357000 | 4.608030000 |
| C | –6.305434000 | 1.953588000 | 3.363150000 |
| C | –6.446170000 | 2.943545000 | 4.520080000 |
| C | –5.206851000 | 2.920510000 | 5.415740000 |
| C | 3.782314000 | 2.183720000 | 3.464800000 |
| C | 4.434616000 | 3.246519000 | 2.566210000 |
| C | 4.734819000 | 1.812233000 | 4.608030000 |
| C | 4.844574000 | 4.483872000 | 3.363150000 |
| C | 5.132661000 | 3.049010000 | 5.415740000 |
| C | 5.772270000 | 4.110775000 | 4.520080000 |
| C | –1.458856000 | 0.021267000 | –1.385770000 |
| C | 0.000000000 | 0.000000000 | –0.850080000 |
| C | 0.747846000 | 1.252773000 | –1.385770000 |
| C | 2.092706000 | 1.493447000 | –1.064000000 |
| C | 2.775864000 | 2.638343000 | –1.478060000 |
| C | 4.247482000 | 2.736365000 | –1.127550000 |
| C | 6.545742000 | 1.795122000 | –1.609460000 |
| C | 7.125450000 | 3.209958000 | –1.633290000 |
| C | 6.302193000 | 4.155382000 | –0.757050000 |
| C | 5.074958000 | 1.797995000 | –2.024110000 |
| C | 4.828922000 | 4.150942000 | –1.169860000 |
| C | 2.098092000 | 3.594578000 | –2.240700000 |
| C | 0.780382000 | 3.361958000 | –2.643850000 |
| C | 0.000000000 | 4.367440000 | –3.464800000 |
| C | 0.797970000 | 5.006590000 | –4.608030000 |
| C | –0.074190000 | 5.969520000 | –5.415740000 |
| C | –0.673900000 | 7.054320000 | –4.520080000 |
| C | –1.460860000 | 6.437460000 | –3.363150000 |
| C | –0.594260000 | 5.463750000 | –2.566210000 |
| C | 0.138000000 | 2.189116000 | –2.230150000 |
| C | 0.711010000 | –1.274040000 | –1.385770000 |
| C | 1.826830000 | –1.214070000 | –2.230150000 |
| C | 2.521350000 | –2.356810000 | –2.643850000 |
| C | 3.782314000 | –2.183720000 | –3.464800000 |
| C | 6.305434000 | –1.953588000 | –3.363150000 |
| C | 6.446170000 | –2.943545000 | –4.520080000 |
| C | 5.206851000 | –2.920510000 | –5.415740000 |
| C | 5.028876000 | –2.217231000 | –2.566210000 |
| C | 3.936849000 | –3.194357000 | –4.608030000 |
| C | 2.063950000 | –3.614290000 | –2.240700000 |
| C | 0.896940000 | –3.723140000 | –1.478060000 |

| | | | |
|---|---|---|---|
| C | 0.246020000 | –5.046610000 | –1.127550000 |
| C | 1.180360000 | –6.257440000 | –1.169860000 |
| C | 0.447570000 | –7.535550000 | –0.757050000 |
| C | –1.718250000 | –6.566340000 | –1.609460000 |
| C | –0.782820000 | –7.775800000 | –1.633290000 |
| C | –0.980370000 | –5.294040000 | –2.024110000 |
| C | 0.247010000 | –2.559060000 | –1.064000000 |
| C | –1.964830000 | –0.975046000 | –2.230150000 |
| C | –3.301732000 | –1.005148000 | –2.643850000 |
| C | –3.782314000 | –2.183720000 | –3.464800000 |
| C | –4.844574000 | –4.483872000 | –3.363150000 |
| C | –5.772270000 | –4.110775000 | –4.520080000 |
| C | –5.132661000 | –3.049010000 | –5.415740000 |
| C | –4.434616000 | –3.246519000 | –2.566210000 |
| C | –4.734819000 | –1.812233000 | –4.608030000 |
| C | –4.162042000 | 0.019712000 | –2.240700000 |
| C | –3.672804000 | 1.084797000 | –1.478060000 |
| C | –4.493502000 | 2.310245000 | –1.127550000 |
| C | –4.827492000 | 4.771218000 | –1.609460000 |
| C | –6.342630000 | 4.565842000 | –1.633290000 |
| C | –6.749763000 | 3.380168000 | –0.757050000 |
| C | –4.094588000 | 3.496045000 | –2.024110000 |
| C | –6.009282000 | 2.106498000 | –1.169860000 |
| C | –2.339716000 | 1.065613000 | –1.064000000 |
| H | –2.198240000 | 0.256320000 | 2.587810000 |
| H | –2.603860000 | 4.512090000 | 2.546030000 |
| H | 0.664980000 | 2.686970000 | 0.485900000 |
| H | 0.135590000 | 4.952490000 | 0.094920000 |
| H | –1.564020000 | 6.388190000 | 2.197380000 |
| H | –2.055020000 | 6.084080000 | 0.521210000 |
| H | 0.642130000 | 5.378320000 | 3.073400000 |
| H | 1.653460000 | 4.421910000 | 1.974760000 |
| H | –0.128710000 | 7.444790000 | –0.296150000 |
| H | –1.130100000 | 8.397360000 | 0.809200000 |
| H | 2.109110000 | 6.431860000 | 0.584250000 |
| H | 2.587710000 | 6.736060000 | 2.263220000 |
| H | 1.315610000 | 8.681400000 | 1.305540000 |
| H | 0.455270000 | 7.956190000 | 2.672830000 |
| H | 4.515599000 | –5.042473000 | 0.584250000 |
| H | 4.539744000 | –5.609053000 | 2.263220000 |
| H | 4.336697000 | –3.245261000 | 3.073400000 |
| H | 3.002756000 | –3.642893000 | 1.974760000 |
| H | 6.860508000 | –5.480052000 | 1.305540000 |
| H | 6.662628000 | –4.372370000 | 2.672830000 |
| H | 5.209515000 | –0.001036000 | 2.546030000 |
| H | 4.221187000 | –2.593669000 | 0.094920000 |
| H | 1.321100000 | 1.775572000 | 2.587810000 |
| H | 1.994494000 | –1.919375000 | 0.485900000 |
| H | 6.511732000 | –3.610929000 | –0.296150000 |
| H | 7.837377000 | –3.219985000 | 0.809200000 |
| H | 6.314345000 | –1.839614000 | 2.197380000 |
| H | 6.296478000 | –1.262340000 | 0.521210000 |
| H | –6.383022000 | –3.833861000 | –0.296150000 |
| H | –6.707277000 | –5.177375000 | 0.809200000 |
| H | –4.750325000 | –4.548576000 | 2.197380000 |
| H | –4.241458000 | –4.821740000 | 0.521210000 |
| H | –8.176118000 | –3.201348000 | 1.305540000 |
| H | –7.117898000 | –3.583820000 | 2.672830000 |
| H | –2.605655000 | –4.511054000 | 2.546030000 |
| H | –4.356777000 | –2.358821000 | 0.094920000 |
| H | –6.624709000 | –1.389387000 | 0.584250000 |
| H | –7.127454000 | –1.127007000 | 2.263220000 |
| H | –4.978827000 | –2.133059000 | 3.073400000 |
| H | –4.656216000 | –0.779017000 | 1.974760000 |
| H | 0.877140000 | –2.031892000 | 2.587810000 |
| H | –2.659474000 | –0.767595000 | 0.485900000 |
| H | 0.892500000 | –5.401430000 | 5.892540000 |
| H | –0.513440000 | –6.423150000 | 6.228240000 |
| H | –1.216760000 | –4.221640000 | 5.256750000 |
| H | –1.651830000 | –5.568800000 | 4.190940000 |
| H | 0.849240000 | –3.823720000 | 3.918010000 |
| H | –0.143030000 | –7.674160000 | 4.109330000 |
| H | 1.317610000 | –7.725620000 | 5.108450000 |
| H | 1.857500000 | –7.222680000 | 2.700230000 |
| H | 2.331490000 | –5.889780000 | 3.767360000 |
| H | –0.242220000 | –6.009250000 | 2.090120000 |
| H | 1.175690000 | –5.001110000 | 1.750960000 |
| H | –5.083053000 | 3.214394000 | 2.090120000 |
| H | –4.918933000 | 1.482378000 | 1.750960000 |
| H | –3.736059000 | 1.176397000 | 3.918010000 |
| H | –3.047667000 | 3.164565000 | 5.256750000 |
| H | –3.996807000 | 4.214927000 | 4.190940000 |
| H | –7.183774000 | 2.002698000 | 2.700230000 |
| H | –6.266444000 | 0.925760000 | 3.767360000 |
| H | –6.574503000 | 3.960948000 | 4.109330000 |
| H | –7.349388000 | 2.721726000 | 5.108450000 |
| H | –5.124026000 | 1.927787000 | 5.892540000 |
| H | –5.305891000 | 3.656227000 | 6.228240000 |

| | | | |
|---|---|---|---|
| H | 2.886819000 | 2.647323000 | 3.918010000 |
| H | 5.325273000 | 2.794856000 | 2.090120000 |
| H | 3.743243000 | 3.518732000 | 1.750960000 |
| H | 4.264427000 | 1.057075000 | 5.256750000 |
| H | 5.648637000 | 1.353873000 | 4.190940000 |
| H | 5.326274000 | 5.219982000 | 2.700230000 |
| H | 3.934954000 | 4.964020000 | 3.767360000 |
| H | 4.231526000 | 3.473643000 | 5.892540000 |
| H | 5.819331000 | 2.766923000 | 6.228240000 |
| H | 6.717533000 | 3.713212000 | 4.109330000 |
| H | 6.031778000 | 5.003894000 | 5.108450000 |
| H | 2.659474000 | 0.767595000 | –0.485900000 |
| H | 4.356777000 | 2.358821000 | –0.094920000 |
| H | 6.624709000 | 1.389387000 | –0.584250000 |
| H | 7.127454000 | 1.127007000 | –2.263220000 |
| H | 8.176118000 | 3.201348000 | –1.305540000 |
| H | 7.117898000 | 3.583820000 | –2.672830000 |
| H | 6.383022000 | 3.833861000 | 0.296150000 |
| H | 6.707277000 | 5.177375000 | –0.809200000 |
| H | 4.978827000 | 2.133059000 | –3.073400000 |
| H | 4.656216000 | 0.779017000 | –1.974760000 |
| H | 4.750325000 | 4.548576000 | –2.197380000 |
| H | 4.241458000 | 4.821740000 | –0.521210000 |
| H | 2.605655000 | 4.511054000 | –2.546030000 |
| H | –0.849240000 | 3.823720000 | –3.918010000 |
| H | 1.216760000 | 4.221640000 | –5.256750000 |
| H | 1.651830000 | 5.568800000 | –4.190940000 |
| H | –0.892500000 | 5.401430000 | –5.892540000 |
| H | 0.513440000 | 6.423150000 | –6.228240000 |
| H | 0.143030000 | 7.674160000 | –4.109330000 |
| H | –1.317610000 | 7.725620000 | –5.108450000 |
| H | –1.857500000 | 7.222680000 | –2.700230000 |
| H | –2.331490000 | 5.889780000 | –3.767360000 |
| H | 0.242220000 | 6.009250000 | –2.090120000 |
| H | –1.175690000 | 5.001110000 | –1.750960000 |
| H | –0.877140000 | 2.031892000 | –2.587810000 |
| H | 2.198240000 | –0.256320000 | –2.587810000 |
| H | 3.736059000 | –1.176397000 | –3.918010000 |
| H | 7.183774000 | –2.002698000 | –2.700230000 |
| H | 6.266444000 | –0.925760000 | –3.767360000 |
| H | 6.574503000 | –3.960948000 | –4.109330000 |
| H | 7.349388000 | –2.721726000 | –5.108450000 |
| H | 5.124026000 | –1.927787000 | –5.892540000 |
| H | 5.305891000 | –3.656227000 | –6.228240000 |
| H | 5.083053000 | –3.214394000 | –2.090120000 |
| H | 4.918933000 | –1.482378000 | –1.750960000 |
| H | 3.047667000 | –3.164565000 | –5.256750000 |
| H | 3.996807000 | –4.214927000 | –4.190940000 |
| H | 2.603860000 | –4.512090000 | –2.546030000 |
| H | –0.135590000 | –4.952490000 | –0.094920000 |
| H | 1.564020000 | –6.388190000 | –2.197380000 |
| H | 2.055020000 | –6.084080000 | –0.521210000 |
| H | 0.128710000 | –7.444790000 | 0.296150000 |
| H | 1.130100000 | –8.397360000 | –0.809200000 |
| H | –2.109110000 | –6.431860000 | –0.584250000 |
| H | –2.587710000 | –6.736060000 | –2.263220000 |
| H | –1.315610000 | –8.681400000 | –1.305540000 |
| H | –0.455270000 | –7.956190000 | –2.672830000 |
| H | –0.642130000 | –5.378320000 | –3.073400000 |
| H | –1.653460000 | –4.421910000 | –1.974760000 |
| H | –0.664980000 | –2.686970000 | –0.485900000 |
| H | –1.321100000 | –1.775572000 | –2.587810000 |
| H | –2.886819000 | –2.647323000 | –3.918010000 |
| H | –5.326274000 | –5.219982000 | –2.700230000 |
| H | –3.934954000 | –4.964020000 | –3.767360000 |
| H | –6.717533000 | –3.713212000 | –4.109330000 |
| H | –6.031778000 | –5.003894000 | –5.108450000 |
| H | –4.231526000 | –3.473643000 | –5.892540000 |
| H | –5.819331000 | –2.766923000 | –6.228240000 |
| H | –5.325273000 | –2.794856000 | –2.090120000 |
| H | –3.743243000 | –3.518732000 | –1.750960000 |
| H | –4.264427000 | –1.057075000 | –5.256750000 |
| H | –5.648637000 | –1.353873000 | –4.190940000 |
| H | –5.209515000 | 0.001036000 | –2.546030000 |
| H | –4.221187000 | 2.593669000 | –0.094920000 |
| H | –4.515599000 | 5.042473000 | –0.584250000 |
| H | –4.539744000 | 5.609053000 | –2.263220000 |
| H | –6.860508000 | 5.480052000 | –1.305540000 |
| H | –6.662628000 | 4.372370000 | –2.672830000 |
| H | –6.511732000 | 3.610929000 | 0.296150000 |
| H | –7.837377000 | 3.219985000 | –0.809200000 |
| H | –4.336697000 | 3.245261000 | –3.073400000 |
| H | –3.002756000 | 3.642893000 | –1.974760000 |
| H | –6.314345000 | 1.839614000 | –2.197380000 |
| H | –6.296478000 | 1.262340000 | –0.521210000 |
| H | –1.994494000 | 1.919375000 | –0.485900000 |

Ad-**1**$_2$

B3LYP

| | | | |
|---|---|---|---|
| C | 0.000000000 | 0.000000000 | 0.855050000 |
| C | 1.129285000 | –1.627266000 | 2.538220000 |
| C | 1.255460000 | –2.844271000 | 3.230980000 |
| C | 0.232715000 | –1.445159000 | 1.468580000 |
| C | 0.454880000 | –3.915362000 | 2.822100000 |
| C | –0.563011000 | –2.556129000 | 1.112530000 |
| C | –0.465568000 | –3.794725000 | 1.769710000 |
| C | –2.335378000 | –4.724588000 | 0.264080000 |
| C | –1.347501000 | –5.007995000 | 1.419190000 |
| C | –0.464957000 | –6.219585000 | 0.999570000 |
| C | –2.185540000 | –5.431469000 | 2.662260000 |
| C | –3.202696000 | –5.961921000 | –0.046610000 |
| C | –2.298801000 | –7.139941000 | –0.455780000 |
| C | –4.015615000 | –6.352059000 | 1.202210000 |
| C | –1.329873000 | –7.459886000 | 0.697520000 |
| C | –3.048073000 | –6.671877000 | 2.356770000 |
| C | –2.134288000 | –7.845452000 | 1.953900000 |
| C | –4.253978000 | 5.975647000 | 2.356770000 |
| C | –3.611020000 | 4.608468000 | 2.662260000 |
| C | –3.493237000 | 6.653654000 | 1.202210000 |
| C | –5.727216000 | 5.771074000 | 1.953900000 |
| C | –3.618243000 | 1.563744000 | 2.822100000 |
| C | –3.663300000 | 3.670968000 | 1.419190000 |
| C | –3.090941000 | 0.334875000 | 3.230980000 |
| C | –3.561827000 | 5.754577000 | –0.046610000 |
| C | –3.053544000 | 2.300557000 | 1.769710000 |
| C | –2.923924000 | 4.384790000 | 0.264080000 |
| C | –1.973896000 | –0.164357000 | 2.538220000 |
| C | –1.932167000 | 1.765647000 | 1.112530000 |
| C | –1.367902000 | 0.521042000 | 1.468580000 |
| C | –5.795514000 | 4.881647000 | 0.697520000 |
| C | –5.153840000 | 3.512457000 | 0.999570000 |
| C | –5.033970000 | 5.560790000 | –0.455780000 |
| C | 7.125387000 | 2.578239000 | 0.697520000 |
| C | 5.618797000 | 2.707128000 | 0.999570000 |
| C | 7.861505000 | 2.074378000 | 1.953900000 |
| C | 7.332770000 | 1.579151000 | –0.455780000 |
| C | 3.163363000 | 2.351618000 | 2.822100000 |
| C | 5.010801000 | 1.337027000 | 1.419190000 |
| C | 1.835481000 | 2.509396000 | 3.230980000 |
| C | 7.302051000 | 0.696230000 | 2.356770000 |
| C | 3.519113000 | 1.494169000 | 1.769710000 |
| C | 5.796560000 | 0.823001000 | 2.662260000 |
| C | 0.844611000 | 1.791622000 | 2.538220000 |
| C | 2.495178000 | 0.790482000 | 1.112530000 |
| C | 1.135187000 | 0.924116000 | 1.468580000 |
| C | 6.764523000 | 0.207344000 | –0.046610000 |
| C | 5.259302000 | 0.339797000 | 0.264080000 |
| C | 7.508852000 | –0.301595000 | 1.202210000 |
| C | 0.472379000 | 3.895661000 | 7.193770000 |
| C | 1.983298000 | 3.866288000 | 6.896190000 |
| C | 2.255660000 | 2.933634000 | 5.698820000 |
| C | –0.277577000 | 4.411167000 | 5.951300000 |
| C | 0.000000000 | 3.478113000 | 4.755520000 |
| C | 1.511660000 | 3.426532000 | 4.422160000 |
| C | 2.467414000 | 5.290108000 | 6.557920000 |
| C | 0.198189000 | 5.838113000 | 5.619050000 |
| C | 1.709013000 | 5.810857000 | 5.321210000 |
| C | 1.977694000 | 4.880782000 | 4.122280000 |
| C | 3.238034000 | –4.153125000 | 4.122280000 |
| C | 2.211634000 | –3.022402000 | 4.422160000 |
| C | 1.412772000 | –3.420276000 | 5.698820000 |
| C | 4.177843000 | –4.385478000 | 5.321210000 |
| C | 3.012134000 | –1.739056000 | 4.755520000 |
| C | 3.347661000 | –4.781897000 | 6.557920000 |
| C | 2.356654000 | –3.650731000 | 6.896190000 |
| C | 4.956859000 | –3.090693000 | 5.619050000 |
| C | 3.958971000 | –1.965195000 | 5.951300000 |
| C | 3.137552000 | –2.356923000 | 7.193770000 |
| C | –3.012134000 | –1.739056000 | 4.755520000 |
| C | –3.723293000 | –0.404130000 | 4.422160000 |
| C | –5.215729000 | –0.727658000 | 4.122280000 |
| C | –3.681394000 | –2.445972000 | 5.951300000 |
| C | –3.668432000 | 0.486642000 | 5.698820000 |
| C | –5.155048000 | –2.747420000 | 5.619050000 |
| C | –5.886857000 | –1.425380000 | 5.321210000 |
| C | –3.609931000 | –1.538738000 | 7.193770000 |
| C | –4.339953000 | –0.215557000 | 6.896190000 |
| C | –5.815075000 | –0.508211000 | 6.557920000 |
| C | 1.367902000 | –0.521042000 | –1.468580000 |
| C | 0.000000000 | 0.000000000 | –0.855050000 |
| C | –1.135187000 | –0.924116000 | –1.468580000 |
| C | –2.495178000 | –0.790482000 | –1.112530000 |
| C | –3.519113000 | –1.494169000 | –1.769710000 |
| C | –5.010801000 | –1.337027000 | –1.419190000 |
| C | –5.259302000 | –0.339797000 | –0.264080000 |
| C | –6.764523000 | –0.207344000 | 0.046610000 |
| C | –7.508852000 | 0.301595000 | –1.202210000 |
| C | –7.302051000 | –0.696230000 | –2.356770000 |
| C | –7.861505000 | –2.074378000 | –1.953900000 |
| C | –7.125387000 | –2.578239000 | –0.697520000 |
| C | –7.332770000 | –1.579151000 | 0.455780000 |
| C | –5.796560000 | –0.823001000 | –2.662260000 |
| C | –5.618797000 | –2.707128000 | –0.999570000 |
| C | –3.163363000 | –2.351618000 | –2.822100000 |
| C | –1.835481000 | –2.509396000 | –3.230980000 |
| C | –1.511660000 | –3.426532000 | –4.422160000 |
| C | –2.255660000 | –2.933634000 | –5.698820000 |
| C | –1.983298000 | –3.866288000 | –6.896190000 |
| C | –2.467414000 | –5.290108000 | –6.557920000 |
| C | –1.709013000 | –5.810857000 | –5.321210000 |
| C | –0.198189000 | –5.838113000 | –5.619050000 |
| C | 0.277577000 | –4.411167000 | –5.951300000 |
| C | –0.472379000 | –3.895661000 | –7.193770000 |
| C | –1.977694000 | –4.880782000 | –4.122280000 |
| C | 0.000000000 | –3.478113000 | –4.755520000 |
| C | –0.844611000 | –1.791622000 | –2.538220000 |
| C | –0.232715000 | 1.445159000 | –1.468580000 |
| C | –1.129285000 | 1.627266000 | –2.538220000 |
| C | –1.255460000 | 2.844271000 | –3.230980000 |
| C | –2.211634000 | 3.022402000 | –4.422160000 |
| C | –3.012134000 | 1.739056000 | –4.755520000 |
| C | –3.958971000 | 1.965195000 | –5.951300000 |
| C | –4.956859000 | 3.090693000 | –5.619050000 |
| C | –4.177843000 | 4.385478000 | –5.321210000 |
| C | –3.347661000 | 4.781897000 | –6.557920000 |
| C | –2.356654000 | 3.650731000 | –6.896190000 |
| C | –3.137552000 | 2.356923000 | –7.193770000 |
| C | –3.238034000 | 4.153125000 | –4.122280000 |
| C | –1.412772000 | 3.420276000 | –5.698820000 |
| C | –0.454880000 | 3.915362000 | –2.822100000 |
| C | 0.465568000 | 3.794725000 | –1.769710000 |
| C | 1.347501000 | 5.007995000 | –1.419190000 |
| C | 0.464957000 | 6.219585000 | –0.999570000 |
| C | 1.329873000 | 7.459886000 | –0.697520000 |
| C | 2.298801000 | 7.139941000 | 0.455780000 |
| C | 3.202696000 | 5.961921000 | 0.046610000 |
| C | 4.015615000 | 6.352059000 | –1.202210000 |
| C | 3.048073000 | 6.671877000 | –2.356770000 |
| C | 2.134288000 | 7.845452000 | –1.953900000 |
| C | 2.335378000 | 4.724588000 | –0.264080000 |
| C | 2.185540000 | 5.431469000 | –2.662260000 |
| C | 0.563011000 | 2.556129000 | –1.112530000 |
| C | 1.973896000 | 0.164357000 | –2.538220000 |
| C | 3.090941000 | –0.334875000 | –3.230980000 |
| C | 3.723293000 | 0.404130000 | –4.422160000 |
| C | 3.012134000 | 1.739056000 | –4.755520000 |
| C | 3.681394000 | 2.445972000 | –5.951300000 |
| C | 5.155048000 | 2.747420000 | –5.619050000 |
| C | 5.886857000 | 1.425380000 | –5.321210000 |
| C | 5.815075000 | 0.508211000 | –6.557920000 |
| C | 4.339953000 | 0.215557000 | –6.896190000 |
| C | 3.609931000 | 1.538738000 | –7.193770000 |
| C | 5.215729000 | 0.727658000 | –4.122280000 |
| C | 3.668432000 | –0.486642000 | –5.698820000 |
| C | 3.618243000 | –1.563744000 | –2.822100000 |
| C | 3.053544000 | –2.300557000 | –1.769710000 |
| C | 3.663300000 | –3.670968000 | –1.419190000 |
| C | 2.923924000 | –4.384790000 | –0.264080000 |
| C | 3.561827000 | –5.754577000 | 0.046610000 |
| C | 3.493237000 | –6.653654000 | –1.202210000 |
| C | 4.253978000 | –5.975647000 | –2.356770000 |
| C | 5.727216000 | –5.771074000 | –1.953900000 |
| C | 5.795514000 | –4.881647000 | –0.697520000 |
| C | 5.033970000 | –5.560790000 | 0.455780000 |
| C | 3.611020000 | –4.608468000 | –2.662260000 |
| C | 5.153840000 | –3.512457000 | –0.999570000 |
| C | 1.932167000 | –1.765647000 | –1.112530000 |
| H | 1.732695000 | –0.790586000 | 2.865860000 |
| H | 0.534892000 | –4.867617000 | 3.348380000 |
| H | –1.296685000 | –2.443554000 | 0.324100000 |
| H | –1.783241000 | –4.422260000 | –0.640460000 |
| H | –2.987350000 | –3.877044000 | 0.530600000 |
| H | 0.256583000 | –6.457891000 | 1.798440000 |
| H | 0.126637000 | –5.947232000 | 0.109180000 |
| H | –1.519383000 | –5.647061000 | 3.513580000 |
| H | –2.828915000 | –4.588945000 | 2.969210000 |
| H | –3.889246000 | –5.716239000 | –0.874150000 |
| H | –2.912456000 | –8.025867000 | –0.696660000 |
| H | –1.731113000 | –6.885059000 | –1.367630000 |
| H | –4.690832000 | –5.527259000 | 1.490400000 |
| H | –4.652737000 | –7.227380000 | 0.984240000 |

| | | | |
|---|---|---|---|
| H | –0.670176000 | –8.296037000 | 0.408460000 |
| H | –3.622231000 | –6.941844000 | 3.259800000 |
| H | –2.739200000 | –8.747698000 | 1.755970000 |
| H | –1.448440000 | –8.095026000 | 2.782850000 |
| H | –4.200698000 | 6.607866000 | 3.259800000 |
| H | –4.130807000 | 4.139354000 | 3.513580000 |
| H | –2.559685000 | 4.744385000 | 2.969210000 |
| H | –2.441331000 | 6.826009000 | 1.490400000 |
| H | –3.932726000 | 7.643078000 | 0.984240000 |
| H | –6.206129000 | 6.746066000 | 1.755970000 |
| H | –6.286278000 | 5.301899000 | 2.782850000 |
| H | –4.482926000 | 1.970579000 | 3.348380000 |
| H | –3.005786000 | 6.226305000 | –0.874150000 |
| H | –2.938169000 | 3.755462000 | –0.640460000 |
| H | –1.863944000 | 4.525643000 | 0.530600000 |
| H | –1.551015000 | –1.105265000 | 2.865860000 |
| H | –1.467837000 | 2.344739000 | 0.324100000 |
| H | –6.849490000 | 4.728408000 | 0.408460000 |
| H | –5.720989000 | 3.006738000 | 1.798440000 |
| H | –5.213772000 | 2.863945000 | 0.109180000 |
| H | –5.494377000 | 6.535195000 | –0.696660000 |
| H | –5.097079000 | 4.941717000 | –1.367630000 |
| H | 7.519667000 | 3.567629000 | 0.408460000 |
| H | 5.464406000 | 3.451152000 | 1.798440000 |
| H | 5.087136000 | 3.083287000 | 0.109180000 |
| H | 8.945329000 | 2.001632000 | 1.755970000 |
| H | 7.734719000 | 2.793127000 | 2.782850000 |
| H | 8.406833000 | 1.490673000 | –0.696660000 |
| H | 6.828192000 | 1.943342000 | –1.367630000 |
| H | 3.948034000 | 2.897039000 | 3.348380000 |
| H | 7.822929000 | 0.333978000 | 3.259800000 |
| H | 5.650189000 | 1.507706000 | 3.513580000 |
| H | 5.388600000 | –0.155440000 | 2.969210000 |
| H | –0.181680000 | 1.895851000 | 2.865860000 |
| H | 2.764522000 | 0.098815000 | 0.324100000 |
| H | 6.895031000 | –0.510066000 | –0.874150000 |
| H | 4.721410000 | 0.666798000 | –0.640460000 |
| H | 4.851294000 | –0.648599000 | 0.530600000 |
| H | 7.132163000 | –1.298750000 | 1.490400000 |
| H | 8.585463000 | –0.415698000 | 0.984240000 |
| H | 0.266600000 | 4.547563000 | 8.061040000 |
| H | 0.117377000 | 2.884650000 | 7.460410000 |
| H | 2.529478000 | 3.488554000 | 7.777580000 |
| H | 1.929790000 | 1.905378000 | 5.934630000 |
| H | 3.340511000 | 2.885939000 | 5.508880000 |
| H | –1.362589000 | 4.419759000 | 6.150860000 |
| H | –0.566587000 | 3.827728000 | 3.876690000 |
| H | –0.368687000 | 2.464771000 | 4.987990000 |
| H | 3.554378000 | 5.285078000 | 6.362620000 |
| H | 2.299464000 | 5.962849000 | 7.417160000 |
| H | –0.012749000 | 6.516338000 | 6.464530000 |
| H | –0.353949000 | 6.230081000 | 4.746750000 |
| H | 2.058157000 | 6.827932000 | 5.073290000 |
| H | 3.053110000 | 4.889885000 | 3.879700000 |
| H | 1.449330000 | 5.253730000 | 3.227750000 |
| H | 2.708209000 | –5.089013000 | 3.879700000 |
| H | 3.825198000 | –3.882022000 | 3.227750000 |
| H | 0.685211000 | –2.623936000 | 5.934630000 |
| H | 0.829041000 | –4.335937000 | 5.508880000 |
| H | 4.884084000 | –5.196382000 | 5.073290000 |
| H | 3.598203000 | –1.423185000 | 3.876690000 |
| H | 2.318898000 | –0.913093000 | 4.987990000 |
| H | 2.799822000 | –5.720720000 | 6.362620000 |
| H | 4.014246000 | –4.972818000 | 7.417160000 |
| H | 1.756438000 | –3.934869000 | 7.777580000 |
| H | 5.649689000 | –3.247128000 | 6.464530000 |
| H | 5.572383000 | –2.808511000 | 4.746750000 |
| H | 4.508918000 | –1.029843000 | 6.150860000 |
| H | 3.805005000 | –2.504664000 | 8.061040000 |
| H | 2.439492000 | –1.543977000 | 7.460410000 |
| H | –3.031616000 | –2.404543000 | 3.876690000 |
| H | –1.950211000 | –1.551678000 | 4.987990000 |
| H | –5.761319000 | 0.199129000 | 3.879700000 |
| H | –5.274529000 | –1.371708000 | 3.227750000 |
| H | –3.146329000 | –3.389916000 | 6.150860000 |
| H | –2.615001000 | 0.718558000 | 5.934630000 |
| H | –4.169552000 | 1.449998000 | 5.508880000 |
| H | –5.636940000 | –3.269210000 | 6.464530000 |
| H | –5.218434000 | –3.421569000 | 4.746750000 |
| H | –6.942241000 | –1.631550000 | 5.073290000 |
| H | –4.071605000 | –2.042899000 | 8.061040000 |
| H | –2.556869000 | –1.340674000 | 7.460410000 |
| H | –4.285915000 | 0.446315000 | 7.777580000 |
| H | –6.354200000 | 0.435643000 | 6.362620000 |
| H | –6.313710000 | –0.990030000 | 7.417160000 |
| H | –2.764522000 | –0.098815000 | –0.324100000 |
| H | –4.721410000 | –0.666798000 | 0.640460000 |
| H | –4.851294000 | 0.648599000 | –0.530600000 |
| H | –6.895031000 | 0.510066000 | 0.874150000 |
| H | –7.132163000 | 1.298750000 | –1.490400000 |
| H | –8.585463000 | 0.415698000 | –0.984240000 |
| H | –7.822929000 | –0.333978000 | –3.259800000 |
| H | –8.945329000 | –2.001632000 | –1.755970000 |
| H | –7.734719000 | –2.793127000 | –2.782850000 |
| H | –7.519667000 | –3.567629000 | –0.408460000 |
| H | –8.406833000 | –1.490673000 | 0.696660000 |
| H | –6.828192000 | –1.943342000 | 1.367630000 |
| H | –5.650189000 | –1.507706000 | –3.513580000 |
| H | –5.388600000 | 0.155440000 | –2.969210000 |
| H | –5.464406000 | –3.451152000 | –1.798440000 |
| H | –5.087136000 | –3.083287000 | –0.109180000 |
| H | –3.948034000 | –2.897039000 | –3.348380000 |
| H | –1.929790000 | –1.905378000 | –5.934630000 |
| H | –3.340511000 | –2.885939000 | –5.508880000 |
| H | –2.529478000 | –3.488554000 | –7.777580000 |
| H | –3.554378000 | –5.285078000 | –6.362620000 |
| H | –2.299464000 | –5.962849000 | –7.417160000 |
| H | –2.058157000 | –6.827932000 | –5.073290000 |
| H | 0.012749000 | –6.516338000 | –6.464530000 |
| H | 0.353949000 | –6.230081000 | –4.746750000 |
| H | 1.362589000 | –4.419759000 | –6.150860000 |
| H | –0.266600000 | –4.547563000 | –8.061040000 |
| H | –0.117377000 | –2.884650000 | –7.460410000 |
| H | –3.053110000 | –4.889885000 | –3.879700000 |
| H | –1.449330000 | –5.253730000 | –3.227750000 |
| H | 0.566587000 | –3.827728000 | –3.876690000 |
| H | 0.368687000 | –2.464771000 | –4.987990000 |
| H | 0.181680000 | –1.895851000 | –2.865860000 |
| H | –1.732695000 | 0.790586000 | –2.865860000 |
| H | –3.598203000 | 1.423185000 | –3.876690000 |
| H | –2.318898000 | 0.913093000 | –4.987990000 |
| H | –4.508918000 | 1.029843000 | –6.150860000 |
| H | –5.649689000 | 3.247128000 | –6.464530000 |
| H | –5.572383000 | 2.808511000 | –4.746750000 |
| H | –4.884084000 | 5.196382000 | –5.073290000 |
| H | –2.799822000 | 5.720720000 | –6.362620000 |
| H | –4.014246000 | 4.972818000 | –7.417160000 |
| H | –1.756438000 | 3.934869000 | –7.777580000 |
| H | –3.805005000 | 2.504664000 | –8.061040000 |
| H | –2.439492000 | 1.543977000 | –7.460410000 |
| H | –2.708209000 | 5.089013000 | –3.879700000 |
| H | –3.825198000 | 3.882022000 | –3.227750000 |
| H | –0.685211000 | 2.623936000 | –5.934630000 |
| H | –0.829041000 | 4.335937000 | –5.508880000 |
| H | –0.534892000 | 4.867617000 | –3.348380000 |
| H | –0.256583000 | 6.457891000 | –1.798440000 |
| H | –0.126637000 | 5.947232000 | –0.109180000 |
| H | 0.670176000 | 8.296037000 | –0.408460000 |
| H | 2.912456000 | 8.025867000 | 0.696660000 |
| H | 1.731113000 | 6.885059000 | 1.367630000 |
| H | 3.889246000 | 5.716239000 | 0.874150000 |
| H | 4.690832000 | 5.527259000 | –1.490400000 |
| H | 4.652737000 | 7.227380000 | –0.984240000 |
| H | 3.622231000 | 6.941844000 | –3.259800000 |
| H | 2.739200000 | 8.747698000 | –1.755970000 |
| H | 1.448440000 | 8.095026000 | –2.782850000 |
| H | 1.783241000 | 4.422260000 | 0.640460000 |
| H | 2.987350000 | 3.877044000 | –0.530600000 |
| H | 1.519383000 | 5.647061000 | –3.513580000 |
| H | 2.828915000 | 4.588945000 | –2.969210000 |
| H | 1.296685000 | 2.443554000 | –0.324100000 |
| H | 1.551015000 | 1.105265000 | –2.865860000 |
| H | 3.031616000 | 2.404543000 | –3.876690000 |
| H | 1.950211000 | 1.551678000 | –4.987990000 |
| H | 3.146329000 | 3.389916000 | –6.150860000 |
| H | 5.636940000 | 3.269210000 | –6.464530000 |
| H | 5.218434000 | 3.421569000 | –4.746750000 |
| H | 6.942241000 | 1.631550000 | –5.073290000 |
| H | 6.354200000 | –0.435643000 | –6.362620000 |
| H | 6.313710000 | 0.990030000 | –7.417160000 |
| H | 4.285915000 | –0.446315000 | –7.777580000 |
| H | 4.071605000 | 2.042899000 | –8.061040000 |
| H | 2.556869000 | 1.340674000 | –7.460410000 |
| H | 5.761319000 | –0.199129000 | –3.879700000 |
| H | 5.274529000 | 1.371708000 | –3.227750000 |
| H | 2.615001000 | –0.718558000 | –5.934630000 |
| H | 4.169552000 | –1.449998000 | –5.508880000 |
| H | 4.482926000 | –1.970579000 | –3.348380000 |
| H | 2.938169000 | –3.755462000 | 0.640460000 |
| H | 1.863944000 | –4.525643000 | –0.530600000 |
| H | 3.005786000 | –6.226305000 | 0.874150000 |
| H | 2.441331000 | –6.826009000 | –1.490400000 |
| H | 3.932726000 | –7.643078000 | –0.984240000 |
| H | 4.200698000 | –6.607866000 | –3.259800000 |
| H | 6.206129000 | –6.746066000 | –1.755970000 |
| H | 6.286278000 | –5.301899000 | –2.782850000 |

| | | | |
|---|---|---|---|
| H | 6.849490000 | –4.728408000 | –0.408460000 |
| H | 5.494377000 | –6.535195000 | 0.696660000 |
| H | 5.097079000 | –4.941717000 | 1.367630000 |
| H | 4.130807000 | –4.139354000 | –3.513580000 |
| H | 2.559685000 | –4.744385000 | –2.969210000 |
| H | 5.720989000 | –3.006738000 | –1.798440000 |
| H | 5.213772000 | –2.863945000 | –0.109180000 |
| H | 1.467837000 | –2.344739000 | –0.324100000 |

B3LYP-D3(BJ)

| | | | |
|---|---|---|---|
| C | 0.000000000 | 0.000000000 | 0.830910000 |
| C | –1.480055000 | 1.356254000 | 2.444990000 |
| C | –1.883299000 | 2.534573000 | 3.090440000 |
| C | –0.524698000 | 1.346940000 | 1.423370000 |
| C | –1.291324000 | 3.738262000 | 2.697740000 |
| C | 0.060065000 | 2.576919000 | 1.078910000 |
| C | –0.304756000 | 3.774766000 | 1.699960000 |
| C | 1.397028000 | 4.943890000 | 0.199250000 |
| C | 0.394646000 | 5.090982000 | 1.363560000 |
| C | –0.635622000 | 6.179973000 | 0.969700000 |
| C | 1.183225000 | 5.583449000 | 2.605860000 |
| C | 2.097544000 | 6.277906000 | –0.101220000 |
| C | 1.054269000 | 7.343721000 | –0.476960000 |
| C | 2.870068000 | 6.736468000 | 1.147320000 |
| C | 0.066431000 | 7.519378000 | 0.688020000 |
| C | 1.885939000 | 6.918933000 | 2.316070000 |
| C | 0.830638000 | 7.975440000 | 1.943770000 |
| C | 5.049002000 | –5.092738000 | 2.316070000 |
| C | 4.243796000 | –3.816428000 | 2.605860000 |
| C | 4.398918000 | –5.853786000 | 1.147320000 |
| C | 6.491614000 | –4.707074000 | 1.943770000 |
| C | 3.883092000 | –0.750812000 | 2.697740000 |
| C | 4.211596000 | –2.887264000 | 1.363560000 |
| C | 3.136654000 | 0.363698000 | 3.090440000 |
| C | 4.388054000 | –4.955479000 | –0.101220000 |
| C | 3.421421000 | –1.623457000 | 1.699960000 |
| C | 3.583020000 | –3.681807000 | 0.199250000 |
| C | 1.914578000 | 0.603638000 | 2.444990000 |
| C | 2.201645000 | –1.340477000 | 1.078910000 |
| C | 1.428833000 | –0.219068000 | 1.423370000 |
| C | 6.478757000 | –3.817220000 | 0.688020000 |
| C | 5.669825000 | –2.539522000 | 0.969700000 |
| C | 5.832715000 | –4.584884000 | –0.476960000 |
| C | –6.545188000 | –3.702158000 | 0.688020000 |
| C | –5.034203000 | –3.640452000 | 0.969700000 |
| C | –7.322253000 | –3.268366000 | 1.943770000 |
| C | –6.886984000 | –2.758837000 | –0.476960000 |
| C | –2.591768000 | –2.987451000 | 2.697740000 |
| C | –4.606242000 | –2.203718000 | 1.363560000 |
| C | –1.253355000 | –2.898271000 | 3.090440000 |
| C | –6.934942000 | –1.826195000 | 2.316070000 |
| C | –3.116666000 | –2.151309000 | 1.699960000 |
| C | –5.427022000 | –1.767022000 | 2.605860000 |
| C | –0.434523000 | –1.959892000 | 2.444990000 |
| C | –2.261710000 | –1.236442000 | 1.078910000 |
| C | –0.904135000 | –1.127871000 | 1.423370000 |
| C | –6.485598000 | –1.322427000 | –0.101220000 |
| C | –4.980049000 | –1.262083000 | 0.199250000 |
| C | –7.268986000 | –0.882682000 | 1.147320000 |
| C | 0.317723000 | –4.416821000 | 6.916200000 |
| C | –1.070442000 | –4.883256000 | 6.447870000 |
| C | –1.619798000 | –3.899105000 | 5.400650000 |
| C | 1.265373000 | –4.361290000 | 5.707550000 |
| C | 0.711835000 | –3.382067000 | 4.658910000 |
| C | –0.685884000 | –3.824711000 | 4.166740000 |
| C | –0.957469000 | –6.284657000 | 5.821150000 |
| C | 1.387664000 | –5.763205000 | 5.084530000 |
| C | 0.000000000 | –6.234803000 | 4.617900000 |
| C | –0.545460000 | –5.250092000 | 3.571840000 |
| C | –4.273983000 | 3.097428000 | 3.571840000 |
| C | –2.969354000 | 2.506349000 | 4.166740000 |
| C | –2.566825000 | 3.352339000 | 5.400650000 |
| C | –5.399498000 | 3.117401000 | 4.617900000 |
| C | –3.284873000 | 1.074567000 | 4.658910000 |
| C | –4.963938000 | 3.971521000 | 5.821150000 |
| C | –3.693802000 | 3.368658000 | 6.447870000 |
| C | –5.684914000 | 1.679850000 | 5.084530000 |
| C | –4.409675000 | 1.084800000 | 5.707550000 |
| C | –3.983941000 | 1.933254000 | 6.916200000 |
| C | 2.573039000 | 2.307500000 | 4.658910000 |
| C | 3.655239000 | 1.318362000 | 4.166740000 |
| C | 4.819443000 | 2.152664000 | 3.571840000 |
| C | 3.144301000 | 3.276490000 | 5.707550000 |
| C | 4.186623000 | 0.546766000 | 5.400650000 |
| C | 4.297250000 | 4.083355000 | 5.084530000 |
| C | 5.399498000 | 3.117401000 | 4.617900000 |
| C | 3.666218000 | 2.483567000 | 6.916200000 |
| C | 4.764245000 | 1.514598000 | 6.447870000 |
| C | 5.921408000 | 2.313136000 | 5.821150000 |
| C | –1.428833000 | 0.219068000 | –1.423370000 |
| C | 0.000000000 | 0.000000000 | –0.830910000 |
| C | 0.904135000 | 1.127871000 | –1.423370000 |
| C | 2.261710000 | 1.236442000 | –1.078910000 |
| C | 3.116666000 | 2.151309000 | –1.699960000 |
| C | 4.606242000 | 2.203718000 | –1.363560000 |
| C | 4.980049000 | 1.262083000 | –0.199250000 |
| C | 6.485598000 | 1.322427000 | 0.101220000 |
| C | 7.268986000 | 0.882682000 | –1.147320000 |
| C | 6.934942000 | 1.826195000 | –2.316070000 |
| C | 7.322253000 | 3.268366000 | –1.943770000 |
| C | 6.545188000 | 3.702158000 | –0.688020000 |
| C | 6.886984000 | 2.758837000 | 0.476960000 |
| C | 5.427022000 | 1.767022000 | –2.605860000 |
| C | 5.034203000 | 3.640452000 | –0.969700000 |
| C | 2.591768000 | 2.987451000 | –2.697740000 |
| C | 1.253355000 | 2.898271000 | –3.090440000 |
| C | 0.685884000 | 3.824711000 | –4.166740000 |
| C | 1.619798000 | 3.899105000 | –5.400650000 |
| C | 1.070442000 | 4.883256000 | –6.447870000 |
| C | 0.957469000 | 6.284657000 | –5.821150000 |
| C | 0.000000000 | 6.234803000 | –4.617900000 |
| C | –1.387664000 | 5.763205000 | –5.084530000 |
| C | –1.265373000 | 4.361290000 | –5.707550000 |
| C | –0.317723000 | 4.416821000 | –6.916200000 |
| C | 0.545460000 | 5.250092000 | –3.571840000 |
| C | –0.711835000 | 3.382067000 | –4.658910000 |
| C | 0.434523000 | 1.959892000 | –2.444990000 |
| C | 0.524698000 | –1.346940000 | –1.423370000 |
| C | 1.480055000 | –1.356254000 | –2.444990000 |
| C | 1.883299000 | –2.534573000 | –3.090440000 |
| C | 2.969354000 | –2.506349000 | –4.166740000 |
| C | 3.284873000 | –1.074567000 | –4.658910000 |
| C | 4.409675000 | –1.084800000 | –5.707550000 |
| C | 5.684914000 | –1.679850000 | –5.084530000 |
| C | 5.399498000 | –3.117401000 | –4.617900000 |
| C | 4.963938000 | –3.971521000 | –5.821150000 |
| C | 3.693802000 | –3.368658000 | –6.447870000 |
| C | 3.983941000 | –1.933254000 | –6.916200000 |
| C | 4.273983000 | –3.097428000 | –3.571840000 |
| C | 2.566825000 | –3.352339000 | –5.400650000 |
| C | 1.291324000 | –3.738262000 | –2.697740000 |
| C | 0.304756000 | –3.774766000 | –1.699960000 |
| C | –0.394646000 | –5.090982000 | –1.363560000 |
| C | 0.635622000 | –6.179973000 | –0.969700000 |
| C | –0.066431000 | –7.519378000 | –0.688020000 |
| C | –1.054269000 | –7.343721000 | 0.476960000 |
| C | –2.097544000 | –6.277906000 | 0.101220000 |
| C | –2.870068000 | –6.736468000 | –1.147320000 |
| C | –1.885939000 | –6.918933000 | –2.316070000 |
| C | –0.830638000 | –7.975440000 | –1.943770000 |
| C | –1.397028000 | –4.943890000 | –0.199250000 |
| C | –1.183225000 | –5.583449000 | –2.605860000 |
| C | –0.060065000 | –2.576919000 | –1.078910000 |
| C | –1.914578000 | –0.603638000 | –2.444990000 |
| C | –3.136654000 | –0.363698000 | –3.090440000 |
| C | –3.655239000 | –1.318362000 | –4.166740000 |
| C | –2.573039000 | –2.307500000 | –4.658910000 |
| C | –3.144301000 | –3.276490000 | –5.707550000 |
| C | –4.297250000 | –4.083355000 | –5.084530000 |
| C | –5.399498000 | –3.117401000 | –4.617900000 |
| C | –5.921408000 | –2.313136000 | –5.821150000 |
| C | –4.764245000 | –1.514598000 | –6.447870000 |
| C | –3.666218000 | –2.483567000 | –6.916200000 |
| C | –4.819443000 | –2.152664000 | –3.571840000 |
| C | –4.186623000 | –0.546766000 | –5.400650000 |
| C | –3.883092000 | 0.750812000 | –2.697740000 |
| C | –3.421421000 | 1.623457000 | –1.699960000 |
| C | –4.211596000 | 2.887264000 | –1.363560000 |
| C | –3.583020000 | 3.681807000 | –0.199250000 |
| C | –4.388054000 | 4.955479000 | 0.101220000 |
| C | –4.398918000 | 5.853786000 | –1.147320000 |
| C | –5.049002000 | 5.092738000 | –2.316070000 |
| C | –6.491614000 | 4.707074000 | –1.943770000 |
| C | –6.478757000 | 3.817220000 | –0.688020000 |
| C | –5.832715000 | 4.584884000 | 0.476960000 |
| C | –4.243796000 | 3.816428000 | –2.605860000 |
| C | –5.669825000 | 2.539522000 | –0.969700000 |
| C | –2.201645000 | 1.340477000 | –1.078910000 |
| H | –1.917608000 | 0.420615000 | 2.762320000 |
| H | –1.590153000 | 4.667168000 | 3.183460000 |
| H | 0.843003000 | 2.592323000 | 0.335640000 |
| H | 0.877790000 | 4.577941000 | –0.698410000 |
| H | 2.155808000 | 4.187033000 | 0.451330000 |
| H | –1.375480000 | 6.311277000 | 1.775480000 |
| H | –1.191649000 | 5.845266000 | 0.077910000 |

| | | | |
|---|---|---|---|
| H | 0.501739000 | 5.693992000 | 3.464940000 |
| H | 1.925113000 | 4.816870000 | 2.883130000 |
| H | 2.798577000 | 6.129223000 | –0.939050000 |
| H | 1.553863000 | 8.302233000 | –0.700110000 |
| H | 0.512016000 | 7.040231000 | –1.386520000 |
| H | 3.636586000 | 5.987646000 | 1.413160000 |
| H | 3.398401000 | 7.683664000 | 0.943610000 |
| H | –0.691637000 | 8.274675000 | 0.421300000 |
| H | 2.434655000 | 7.241819000 | 3.216760000 |
| H | 1.316529000 | 8.948408000 | 1.757250000 |
| H | 0.128604000 | 8.118657000 | 2.783440000 |
| H | 5.054272000 | –5.729383000 | 3.216760000 |
| H | 4.680273000 | –3.281515000 | 3.464940000 |
| H | 3.208975000 | –4.075632000 | 2.883130000 |
| H | 3.367160000 | –6.143199000 | 1.413160000 |
| H | 4.955048000 | –6.784934000 | 0.943610000 |
| H | 7.091284000 | –5.614352000 | 1.757250000 |
| H | 6.966661000 | –4.170703000 | 2.783440000 |
| H | 4.836963000 | –0.956472000 | 3.183460000 |
| H | 3.908775000 | –5.488250000 | –0.939050000 |
| H | 3.525719000 | –3.049159000 | –0.698410000 |
| H | 2.548173000 | –3.960501000 | 0.451330000 |
| H | 1.323068000 | 1.450390000 | 2.762320000 |
| H | 1.823516000 | –2.026224000 | 0.335640000 |
| H | 7.511898000 | –3.538362000 | 0.421300000 |
| H | 6.153466000 | –1.964438000 | 1.775480000 |
| H | 5.657974000 | –1.890635000 | 0.077910000 |
| H | 6.413013000 | –5.496801000 | –0.700110000 |
| H | 5.841011000 | –3.963535000 | –1.386520000 |
| H | –6.820260000 | –4.736313000 | 0.421300000 |
| H | –4.777986000 | –4.346839000 | 1.775480000 |
| H | –4.466324000 | –3.954632000 | 0.077910000 |
| H | –8.407813000 | –3.334056000 | 1.757250000 |
| H | –7.095265000 | –3.947954000 | 2.783440000 |
| H | –7.966876000 | –2.805432000 | –0.700110000 |
| H | –6.353027000 | –3.076696000 | –1.386520000 |
| H | –3.246810000 | –3.710697000 | 3.183460000 |
| H | –7.488927000 | –1.512436000 | 3.216760000 |
| H | –5.182012000 | –2.412478000 | 3.464940000 |
| H | –5.134089000 | –0.741238000 | 2.883130000 |
| H | 0.594541000 | –1.871005000 | 2.762320000 |
| H | –2.666519000 | –0.566099000 | 0.335640000 |
| H | –6.707351000 | –0.640973000 | –0.939050000 |
| H | –4.403508000 | –1.528782000 | –0.698410000 |
| H | –4.703981000 | –0.226532000 | 0.451330000 |
| H | –7.003747000 | 0.155553000 | 1.413160000 |
| H | –8.353449000 | –0.898731000 | 0.943610000 |
| H | 0.714310000 | –5.107385000 | 7.680250000 |
| H | 0.243420000 | –3.420621000 | 7.385100000 |
| H | –1.760786000 | –4.917088000 | 7.307090000 |
| H | –1.724348000 | –2.891088000 | 5.836990000 |
| H | –2.627260000 | –4.222415000 | 5.096540000 |
| H | 2.260588000 | –4.012323000 | 6.029210000 |
| H | 1.412848000 | –3.334485000 | 3.812450000 |
| H | 0.653400000 | –2.364408000 | 5.080550000 |
| H | –1.952725000 | –6.635000000 | 5.497120000 |
| H | –0.587615000 | –7.005570000 | 6.570190000 |
| H | 1.802812000 | –6.471002000 | 5.822300000 |
| H | 2.085607000 | –5.738520000 | 4.230000000 |
| H | 0.079902000 | –7.236716000 | 4.163640000 |
| H | –1.524769000 | –5.591758000 | 3.199260000 |
| H | 0.129648000 | –5.209218000 | 2.701150000 |
| H | –4.080220000 | 4.116368000 | 3.199260000 |
| H | –4.576138000 | 2.492331000 | 2.701150000 |
| H | –1.641582000 | 2.938873000 | 5.836990000 |
| H | –2.343089000 | 4.386481000 | 5.096540000 |
| H | –6.307131000 | 3.549160000 | 4.163640000 |
| H | –3.594172000 | 0.443680000 | 3.812450000 |
| H | –2.374338000 | 0.616343000 | 5.080550000 |
| H | –4.769716000 | 5.008609000 | 5.497120000 |
| H | –5.773194000 | 4.011675000 | 6.570190000 |
| H | –3.377930000 | 3.983429000 | 7.307090000 |
| H | –6.505458000 | 1.674220000 | 5.822300000 |
| H | –6.012507000 | 1.063071000 | 4.230000000 |
| H | –4.605068000 | 0.048435000 | 6.029210000 |
| H | –4.780281000 | 1.935082000 | 7.680250000 |
| H | –3.084054000 | 1.499502000 | 7.385100000 |
| H | 2.181324000 | 2.890804000 | 3.812450000 |
| H | 1.720938000 | 1.748065000 | 5.080550000 |
| H | 5.604989000 | 1.475390000 | 3.199260000 |
| H | 4.446491000 | 2.716887000 | 2.701150000 |
| H | 2.344480000 | 3.963888000 | 6.029210000 |
| H | 3.365929000 | –0.047785000 | 5.836990000 |
| H | 4.970349000 | –0.164066000 | 5.096540000 |
| H | 4.702646000 | 4.796781000 | 5.822300000 |
| H | 3.926900000 | 4.675449000 | 4.230000000 |
| H | 6.227228000 | 3.687555000 | 4.163640000 |
| H | 4.065970000 | 3.172304000 | 7.680250000 |
| H | 2.840634000 | 1.921118000 | 7.385100000 |
| H | 5.138716000 | 0.933659000 | 7.307090000 |
| H | 6.722441000 | 1.626391000 | 5.497120000 |
| H | 6.360809000 | 2.993895000 | 6.570190000 |
| H | 2.666519000 | 0.566099000 | –0.335640000 |
| H | 4.403508000 | 1.528782000 | 0.698410000 |
| H | 4.703981000 | 0.226532000 | –0.451330000 |
| H | 6.707351000 | 0.640973000 | 0.939050000 |
| H | 7.003747000 | –0.155553000 | –1.413160000 |
| H | 8.353449000 | 0.898731000 | –0.943610000 |
| H | 7.488927000 | 1.512436000 | –3.216760000 |
| H | 8.407813000 | 3.334056000 | –1.757250000 |
| H | 7.095265000 | 3.947954000 | –2.783440000 |
| H | 6.820260000 | 4.736313000 | –0.421300000 |
| H | 7.966876000 | 2.805432000 | 0.700110000 |
| H | 6.353027000 | 3.076696000 | 1.386520000 |
| H | 5.182012000 | 2.412478000 | –3.464940000 |
| H | 5.134089000 | 0.741238000 | –2.883130000 |
| H | 4.777986000 | 4.346839000 | –1.775480000 |
| H | 4.466324000 | 3.954632000 | –0.077910000 |
| H | 3.246810000 | 3.710697000 | –3.183460000 |
| H | 1.724348000 | 2.891088000 | –5.836990000 |
| H | 2.627260000 | 4.222415000 | –5.096540000 |
| H | 1.760786000 | 4.917088000 | –7.307090000 |
| H | 1.952725000 | 6.635000000 | –5.497120000 |
| H | 0.587615000 | 7.005570000 | –6.570190000 |
| H | –0.079902000 | 7.236716000 | –4.163640000 |
| H | –1.802812000 | 6.471002000 | –5.822300000 |
| H | –2.085607000 | 5.738520000 | –4.230000000 |
| H | –2.260588000 | 4.012323000 | –6.029210000 |
| H | –0.714310000 | 5.107385000 | –7.680250000 |
| H | –0.243420000 | 3.420621000 | –7.385100000 |
| H | 1.524769000 | 5.591758000 | –3.199260000 |
| H | –0.129648000 | 5.209218000 | –2.701150000 |
| H | –1.412848000 | 3.334485000 | –3.812450000 |
| H | –0.653400000 | 2.364408000 | –5.080550000 |
| H | –0.594541000 | 1.871005000 | –2.762320000 |
| H | 1.917608000 | –0.420615000 | –2.762320000 |
| H | 3.594172000 | –0.443680000 | –3.812450000 |
| H | 2.374338000 | –0.616343000 | –5.080550000 |
| H | 4.605068000 | –0.048435000 | –6.029210000 |
| H | 6.505458000 | –1.674220000 | –5.822300000 |
| H | 6.012507000 | –1.063071000 | –4.230000000 |
| H | 6.307131000 | –3.549160000 | –4.163640000 |
| H | 4.769716000 | –5.008609000 | –5.497120000 |
| H | 5.773194000 | –4.011675000 | –6.570190000 |
| H | 3.377930000 | –3.983429000 | –7.307090000 |
| H | 4.780281000 | –1.935082000 | –7.680250000 |
| H | 3.084054000 | –1.499502000 | –7.385100000 |
| H | 4.080220000 | –4.116368000 | –3.199260000 |
| H | 4.576138000 | –2.492331000 | –2.701150000 |
| H | 1.641582000 | –2.938873000 | –5.836990000 |
| H | 2.343089000 | –4.386481000 | –5.096540000 |
| H | 1.590153000 | –4.667168000 | –3.183460000 |
| H | 1.375480000 | –6.311277000 | –1.775480000 |
| H | 1.191649000 | –5.845266000 | –0.077910000 |
| H | 0.691637000 | –8.274675000 | –0.421300000 |
| H | –1.553863000 | –8.302233000 | 0.700110000 |
| H | –0.512016000 | –7.040231000 | 1.386520000 |
| H | –2.798577000 | –6.129223000 | 0.939050000 |
| H | –3.636586000 | –5.987646000 | –1.413160000 |
| H | –3.398401000 | –7.683664000 | –0.943610000 |
| H | –2.434655000 | –7.241819000 | –3.216760000 |
| H | –1.316529000 | –8.948408000 | –1.757250000 |
| H | –0.128604000 | –8.118657000 | –2.783440000 |
| H | –0.877790000 | –4.577941000 | 0.698410000 |
| H | –2.155808000 | –4.187033000 | –0.451330000 |
| H | –0.501739000 | –5.693992000 | –3.464940000 |
| H | –1.925113000 | –4.816870000 | –2.883130000 |
| H | –0.843003000 | –2.592323000 | –0.335640000 |
| H | –1.323068000 | –1.450390000 | –2.762320000 |
| H | –2.181324000 | –2.890804000 | –3.812450000 |
| H | –1.720938000 | –1.748065000 | –5.080550000 |
| H | –2.344480000 | –3.963888000 | –6.029210000 |
| H | –4.702646000 | –4.796781000 | –5.822300000 |
| H | –3.926900000 | –4.675449000 | –4.230000000 |
| H | –6.227228000 | –3.687555000 | –4.163640000 |
| H | –6.722441000 | –1.626391000 | –5.497120000 |
| H | –6.360809000 | –2.993895000 | –6.570190000 |
| H | –5.138716000 | –0.933659000 | –7.307090000 |
| H | –4.065970000 | –3.172304000 | –7.680250000 |
| H | –2.840634000 | –1.921118000 | –7.385100000 |
| H | –5.604989000 | –1.475390000 | –3.199260000 |
| H | –4.446491000 | –2.716887000 | –2.701150000 |
| H | –3.365929000 | 0.047785000 | –5.836990000 |
| H | –4.970349000 | 0.164066000 | –5.096540000 |
| H | –4.836963000 | 0.956472000 | –3.183460000 |
| H | –3.525719000 | 3.049159000 | 0.698410000 |

| | | | |
|---|---|---|---|
| H | –2.548173000 | 3.960501000 | –0.451330000 |
| H | –3.908775000 | 5.488250000 | 0.939050000 |
| H | –3.367160000 | 6.143199000 | –1.413160000 |
| H | –4.955048000 | 6.784934000 | –0.943610000 |
| H | –5.054272000 | 5.729383000 | –3.216760000 |
| H | –7.091284000 | 5.614352000 | –1.757250000 |
| H | –6.966661000 | 4.170703000 | –2.783440000 |
| H | –7.511898000 | 3.538362000 | –0.421300000 |
| H | –6.413013000 | 5.496801000 | 0.700110000 |
| H | –5.841011000 | 3.963535000 | 1.386520000 |
| H | –4.680273000 | 3.281515000 | –3.464940000 |
| H | –3.208975000 | 4.075632000 | –2.883130000 |
| H | –6.153466000 | 1.964438000 | –1.775480000 |
| H | –5.657974000 | 1.890635000 | –0.077910000 |
| H | –1.823516000 | 2.026224000 | –0.335640000 |

M06-2X

| | | | |
|---|---|---|---|
| C | 0.000000000 | 0.000000000 | 0.831710000 |
| C | –0.145994000 | 2.019448000 | 2.429520000 |
| C | 0.358025000 | 3.170675000 | 3.052570000 |
| C | 0.546862000 | 1.341553000 | 1.422840000 |
| C | 1.609682000 | 3.639780000 | 2.657890000 |
| C | 1.824062000 | 1.827830000 | 1.089940000 |
| C | 2.369686000 | 2.962616000 | 1.693750000 |
| C | 4.473883000 | 2.582998000 | 0.304530000 |
| C | 3.788270000 | 3.437402000 | 1.385720000 |
| C | 3.784116000 | 4.905967000 | 0.904480000 |
| C | 4.643990000 | 3.348725000 | 2.671730000 |
| C | 5.902716000 | 3.075704000 | 0.038070000 |
| C | 5.875291000 | 4.538692000 | –0.425660000 |
| C | 6.725011000 | 2.970859000 | 1.329310000 |
| C | 5.215824000 | 5.402523000 | 0.656140000 |
| C | 6.074344000 | 3.837981000 | 2.416060000 |
| C | 6.029949000 | 5.300622000 | 1.953230000 |
| C | 0.286617000 | –7.179527000 | 2.416060000 |
| C | 0.578086000 | –5.696176000 | 2.671730000 |
| C | –0.789666000 | –7.309460000 | 1.329310000 |
| C | 1.575498000 | –7.872400000 | 1.953230000 |
| C | 2.347301000 | –3.213915000 | 2.657890000 |
| C | 1.082742000 | –4.999439000 | 1.385720000 |
| C | 2.566872000 | –1.895396000 | 3.052570000 |
| C | –0.287720000 | –6.649754000 | 0.038070000 |
| C | 1.380858000 | –3.533516000 | 1.693750000 |
| C | 0.000000000 | –5.165995000 | 0.304530000 |
| C | 1.821890000 | –0.883290000 | 2.429520000 |
| C | 0.670916000 | –2.493599000 | 1.089940000 |
| C | 0.888388000 | –1.144373000 | 1.422840000 |
| C | 2.070811000 | –7.218298000 | 0.656140000 |
| C | 2.356634000 | –5.730124000 | 0.904480000 |
| C | 0.992977000 | –7.357497000 | –0.425660000 |
| C | –7.286634000 | 1.815774000 | 0.656140000 |
| C | –6.140750000 | 0.824157000 | 0.904480000 |
| C | –7.605448000 | 2.571778000 | 1.953230000 |
| C | –6.868268000 | 2.818805000 | –0.425660000 |
| C | –3.956983000 | –0.425865000 | 2.657890000 |
| C | –4.871012000 | 1.562037000 | 1.385720000 |
| C | –2.924897000 | –1.275279000 | 3.052570000 |
| C | –6.360961000 | 3.341545000 | 2.416060000 |
| C | –3.750544000 | 0.570900000 | 1.693750000 |
| C | –5.222076000 | 2.347451000 | 2.671730000 |
| C | –1.675896000 | –1.136158000 | 2.429520000 |
| C | –2.494978000 | 0.665769000 | 1.089940000 |
| C | –1.435250000 | –0.197180000 | 1.422840000 |
| C | –5.614996000 | 3.574050000 | 0.038070000 |
| C | –4.473883000 | 2.582997000 | 0.304530000 |
| C | –5.935345000 | 4.338601000 | 1.329310000 |
| C | –2.907759000 | –3.517367000 | 6.832330000 |
| C | –4.225889000 | –2.907092000 | 6.341070000 |
| C | –3.930713000 | –1.797572000 | 5.322510000 |
| C | –2.147807000 | –4.102442000 | 5.636070000 |
| C | –1.859703000 | –2.995377000 | 4.612850000 |
| C | –3.167386000 | –2.359393000 | 4.101900000 |
| C | –5.080765000 | –3.994185000 | 5.673420000 |
| C | –2.997765000 | –5.197187000 | 4.975480000 |
| C | –4.317353000 | –4.589296000 | 4.482030000 |
| C | –4.015583000 | –3.482589000 | 3.464440000 |
| C | –1.008219000 | 5.218891000 | 3.464440000 |
| C | –0.459602000 | 3.922733000 | 4.101900000 |
| C | 0.408614000 | 4.302883000 | 5.322510000 |
| C | –1.815771000 | 6.033585000 | 4.482030000 |
| C | –1.664221000 | 3.108239000 | 4.612850000 |
| C | –0.918683000 | 6.397164000 | 5.673420000 |
| C | –0.404671000 | 5.113274000 | 6.341070000 |
| C | –3.002014000 | 5.194734000 | 4.975480000 |
| C | –2.478916000 | 3.911277000 | 5.636070000 |
| C | –1.592250000 | 4.276876000 | 6.832330000 |
| C | 3.523924000 | –0.112861000 | 4.612850000 |
| C | 3.626987000 | –1.563340000 | 4.101900000 |
| C | 5.023802000 | –1.736303000 | 3.464440000 |
| C | 4.626723000 | 0.191166000 | 5.636070000 |
| C | 3.522099000 | –2.505312000 | 5.322510000 |
| C | 5.999778000 | 0.002453000 | 4.975480000 |
| C | 6.133123000 | –1.444289000 | 4.482030000 |
| C | 4.500008000 | –0.759509000 | 6.832330000 |
| C | 4.630560000 | –2.206182000 | 6.341070000 |
| C | 5.999448000 | –2.402979000 | 5.673420000 |
| C | –0.888388000 | 1.144373000 | –1.422840000 |
| C | 0.000000000 | 0.000000000 | –0.831710000 |
| C | 1.435250000 | 0.197180000 | –1.422840000 |
| C | 2.494978000 | –0.665769000 | –1.089940000 |
| C | 3.750544000 | –0.570900000 | –1.693750000 |
| C | 4.871012000 | –1.562037000 | –1.385720000 |
| C | 4.473883000 | –2.582997000 | –0.304530000 |
| C | 5.614996000 | –3.574050000 | –0.038070000 |
| C | 5.935345000 | –4.338601000 | –1.329310000 |
| C | 6.360961000 | –3.341545000 | –2.416060000 |
| C | 7.605448000 | –2.571778000 | –1.953230000 |
| C | 7.286634000 | –1.815774000 | –0.656140000 |
| C | 6.868268000 | –2.818805000 | 0.425660000 |
| C | 5.222076000 | –2.347451000 | –2.671730000 |
| C | 6.140750000 | –0.824157000 | –0.904480000 |
| C | 3.956983000 | 0.425865000 | –2.657890000 |
| C | 2.924897000 | 1.275279000 | –3.052570000 |
| C | 3.167386000 | 2.359393000 | –4.101900000 |
| C | 3.930713000 | 1.797572000 | –5.322510000 |
| C | 4.225889000 | 2.907092000 | –6.341070000 |
| C | 5.080765000 | 3.994185000 | –5.673420000 |
| C | 4.317353000 | 4.589296000 | –4.482030000 |
| C | 2.997765000 | 5.197187000 | –4.975480000 |
| C | 2.147807000 | 4.102442000 | –5.636070000 |
| C | 2.907759000 | 3.517367000 | –6.832330000 |
| C | 4.015583000 | 3.482589000 | –3.464440000 |
| C | 1.859703000 | 2.995377000 | –4.612850000 |
| C | 1.675896000 | 1.136158000 | –2.429520000 |
| C | –0.546862000 | –1.341553000 | –1.422840000 |
| C | 0.145994000 | –2.019448000 | –2.429520000 |
| C | –0.358025000 | –3.170675000 | –3.052570000 |
| C | 0.459602000 | –3.922733000 | –4.101900000 |
| C | 1.664221000 | –3.108239000 | –4.612850000 |
| C | 2.478916000 | –3.911277000 | –5.636070000 |
| C | 3.002014000 | –5.194734000 | –4.975480000 |
| C | 1.815771000 | –6.033585000 | –4.482030000 |
| C | 0.918683000 | –6.397164000 | –5.673420000 |
| C | 0.404671000 | –5.113274000 | –6.341070000 |
| C | 1.592250000 | –4.276876000 | –6.832330000 |
| C | 1.008219000 | –5.218891000 | –3.464440000 |
| C | –0.408614000 | –4.302883000 | –5.322510000 |
| C | –1.609682000 | –3.639780000 | –2.657890000 |
| C | –2.369686000 | –2.962616000 | –1.693750000 |
| C | –3.788270000 | –3.437402000 | –1.385720000 |
| C | –3.784116000 | –4.905967000 | –0.904480000 |
| C | –5.215824000 | –5.402523000 | –0.656140000 |
| C | –5.875291000 | –4.538692000 | 0.425660000 |
| C | –5.902716000 | –3.075704000 | –0.038070000 |
| C | –6.725011000 | –2.970859000 | –1.329310000 |
| C | –6.074344000 | –3.837981000 | –2.416060000 |
| C | –6.029949000 | –5.300622000 | –1.953230000 |
| C | –4.473883000 | –2.582998000 | –0.304530000 |
| C | –4.643990000 | –3.348725000 | –2.671730000 |
| C | –1.824062000 | –1.827830000 | –1.089940000 |
| C | –1.821890000 | 0.883290000 | –2.429520000 |
| C | –2.566872000 | 1.895396000 | –3.052570000 |
| C | –3.626987000 | 1.563340000 | –4.101900000 |
| C | –3.523924000 | 0.112861000 | –4.612850000 |
| C | –4.626723000 | –0.191166000 | –5.636070000 |
| C | –5.999778000 | –0.002453000 | –4.975480000 |
| C | –6.133123000 | 1.444289000 | –4.482030000 |
| C | –5.999448000 | 2.402979000 | –5.673420000 |
| C | –4.630560000 | 2.206182000 | –6.341070000 |
| C | –4.500008000 | 0.759509000 | –6.832330000 |
| C | –5.023802000 | 1.736303000 | –3.464440000 |
| C | –3.522099000 | 2.505312000 | –5.322510000 |
| C | –2.347301000 | 3.213915000 | –2.657890000 |
| C | –1.380858000 | 3.533516000 | –1.693750000 |
| C | –1.082742000 | 4.999439000 | –1.385720000 |
| C | 0.000000000 | 5.165995000 | –0.304530000 |
| C | 0.287720000 | 6.649754000 | –0.038070000 |
| C | 0.789666000 | 7.309460000 | –1.329310000 |
| C | –0.286617000 | 7.179527000 | –2.416060000 |
| C | –1.575498000 | 7.872400000 | –1.953230000 |
| C | –2.070811000 | 7.218298000 | –0.656140000 |
| C | –0.992977000 | 7.357497000 | 0.425660000 |
| C | –0.578086000 | 5.696176000 | –2.671730000 |
| C | –2.356634000 | 5.730124000 | –0.904480000 |
| C | –0.670916000 | 2.493599000 | –1.089940000 |

| | | | |
|---|---|---|---|
| H | –1.101153000 | 1.637686000 | 2.765930000 |
| H | 2.021135000 | 4.537410000 | 3.123530000 |
| H | 2.428279000 | 1.283200000 | 0.375270000 |
| H | 3.882961000 | 2.608956000 | –0.626980000 |
| H | 4.505103000 | 1.528448000 | 0.631890000 |
| H | 3.295740000 | 5.547188000 | 1.656620000 |
| H | 3.188916000 | 4.981428000 | –0.022760000 |
| H | 4.180019000 | 3.947823000 | 3.472450000 |
| H | 4.659184000 | 2.301722000 | 3.018270000 |
| H | 6.355383000 | 2.445122000 | –0.744580000 |
| H | 6.902654000 | 4.891617000 | –0.616450000 |
| H | 5.315975000 | 4.629302000 | –1.370110000 |
| H | 6.771269000 | 1.919720000 | 1.663330000 |
| H | 7.760326000 | 3.304331000 | 1.148370000 |
| H | 5.180003000 | 6.451518000 | 0.322610000 |
| H | 6.654475000 | 3.760302000 | 3.349360000 |
| H | 7.052772000 | 5.676094000 | 1.784930000 |
| H | 5.573789000 | 5.928355000 | 2.736800000 |
| H | –0.070720000 | –7.643095000 | 3.349360000 |
| H | 1.328906000 | –5.593914000 | 3.472450000 |
| H | –0.336242000 | –5.185833000 | 3.018270000 |
| H | –1.723108000 | –6.823951000 | 1.663330000 |
| H | –1.018529000 | –8.372805000 | 1.148370000 |
| H | 1.389256000 | –8.945927000 | 1.784930000 |
| H | 2.347211000 | –7.791220000 | 2.736800000 |
| H | 2.918945000 | –4.019059000 | 3.123530000 |
| H | –1.060154000 | –6.726484000 | –0.744580000 |
| H | 0.317942000 | –4.667221000 | –0.626980000 |
| H | –0.928877000 | –4.665758000 | 0.631890000 |
| H | 1.968855000 | 0.134783000 | 2.765930000 |
| H | –0.102856000 | –2.744551000 | 0.375270000 |
| H | 2.997177000 | –7.711773000 | 0.322610000 |
| H | 3.156136000 | –5.627789000 | 1.656620000 |
| H | 2.719585000 | –5.252396000 | –0.022760000 |
| H | 0.784938000 | –8.423683000 | –0.616450000 |
| H | 1.351105000 | –6.918420000 | –1.370110000 |
| H | –8.177180000 | 1.260255000 | 0.322610000 |
| H | –6.451876000 | 0.080601000 | 1.656620000 |
| H | –5.908501000 | 0.270969000 | –0.022760000 |
| H | –8.442028000 | 3.269833000 | 1.784930000 |
| H | –7.921000000 | 1.862865000 | 2.736800000 |
| H | –7.687592000 | 3.532065000 | –0.616450000 |
| H | –6.667080000 | 2.289118000 | –1.370110000 |
| H | –4.940080000 | –0.518351000 | 3.123530000 |
| H | –6.583754000 | 3.882793000 | 3.349360000 |
| H | –5.508925000 | 1.646091000 | 3.472450000 |
| H | –4.322942000 | 2.884111000 | 3.018270000 |
| H | –0.867701000 | –1.772470000 | 2.765930000 |
| H | –2.325423000 | 1.461351000 | 0.375270000 |
| H | –5.295229000 | 4.281363000 | –0.744580000 |
| H | –4.200903000 | 2.058265000 | –0.626980000 |
| H | –3.576226000 | 3.137310000 | 0.631890000 |
| H | –5.048161000 | 4.904231000 | 1.663330000 |
| H | –6.741797000 | 5.068474000 | 1.148370000 |
| H | –3.108002000 | –4.304981000 | 7.577660000 |
| H | –2.296274000 | –2.744444000 | 7.326660000 |
| H | –4.775946000 | –2.476744000 | 7.192400000 |
| H | –3.328418000 | –0.998541000 | 5.787200000 |
| H | –4.881722000 | –1.344298000 | 5.002530000 |
| H | –1.191509000 | –4.531433000 | 5.974730000 |
| H | –1.301271000 | –3.428814000 | 3.767790000 |
| H | –1.216986000 | –2.218182000 | 5.060810000 |
| H | –6.036986000 | –3.564047000 | 5.331320000 |
| H | –5.319273000 | –4.786637000 | 6.401880000 |
| H | –3.200056000 | –6.005646000 | 5.697550000 |
| H | –2.448030000 | –5.643542000 | 4.128860000 |
| H | –4.929425000 | –5.368083000 | 3.999810000 |
| H | –4.953443000 | –3.055830000 | 3.067300000 |
| H | –3.460314000 | –3.898906000 | 2.604160000 |
| H | –0.169704000 | 5.817723000 | 3.067300000 |
| H | –1.646394000 | 4.946173000 | 2.604160000 |
| H | 0.799447000 | 3.381765000 | 5.787200000 |
| H | 1.276664000 | 4.899844000 | 5.002530000 |
| H | –2.184184000 | 6.953049000 | 3.999810000 |
| H | –2.318805000 | 2.841341000 | 3.767790000 |
| H | –1.312509000 | 2.163032000 | 5.060810000 |
| H | –0.068062000 | 7.010207000 | 5.331320000 |
| H | –1.485713000 | 6.999944000 | 6.401880000 |
| H | 0.243050000 | 5.374463000 | 7.192400000 |
| H | –3.601014000 | 5.774153000 | 5.697550000 |
| H | –3.663436000 | 4.941828000 | 4.128860000 |
| H | –3.328582000 | 3.297593000 | 5.974730000 |
| H | –2.174222000 | 4.844099000 | 7.577660000 |
| H | –1.228621000 | 3.360854000 | 7.326660000 |
| H | 3.620076000 | 0.587474000 | 3.767790000 |
| H | 2.529495000 | 0.055150000 | 5.060810000 |
| H | 5.123148000 | –2.761893000 | 3.067300000 |
| H | 5.106709000 | –1.047267000 | 2.604160000 |
| H | 4.520090000 | 1.233840000 | 5.974730000 |
| H | 2.528971000 | –2.383224000 | 5.787200000 |
| H | 3.605057000 | –3.555546000 | 5.002530000 |
| H | 6.801070000 | 0.231493000 | 5.697550000 |
| H | 6.111466000 | 0.701715000 | 4.128860000 |
| H | 7.113609000 | –1.584965000 | 3.999810000 |
| H | 5.282224000 | –0.539118000 | 7.577660000 |
| H | 3.524896000 | –0.616410000 | 7.326660000 |
| H | 4.532897000 | –2.897719000 | 7.192400000 |
| H | 6.105048000 | –3.446160000 | 5.331320000 |
| H | 6.804985000 | –2.213307000 | 6.401880000 |
| H | 2.325423000 | –1.461351000 | –0.375270000 |
| H | 4.200903000 | –2.058265000 | 0.626980000 |
| H | 3.576226000 | –3.137310000 | –0.631890000 |
| H | 5.295229000 | –4.281363000 | 0.744580000 |
| H | 5.048161000 | –4.904231000 | –1.663330000 |
| H | 6.741797000 | –5.068474000 | –1.148370000 |
| H | 6.583754000 | –3.882793000 | –3.349360000 |
| H | 8.442028000 | –3.269833000 | –1.784930000 |
| H | 7.921000000 | –1.862865000 | –2.736800000 |
| H | 8.177180000 | –1.260255000 | –0.322610000 |
| H | 7.687592000 | –3.532065000 | 0.616450000 |
| H | 6.667080000 | –2.289118000 | 1.370110000 |
| H | 5.508925000 | –1.646091000 | –3.472450000 |
| H | 4.322942000 | –2.884111000 | –3.018270000 |
| H | 6.451876000 | –0.080601000 | –1.656620000 |
| H | 5.908501000 | –0.270969000 | 0.022760000 |
| H | 4.940080000 | 0.518351000 | –3.123530000 |
| H | 3.328418000 | 0.998541000 | –5.787200000 |
| H | 4.881722000 | 1.344298000 | –5.002530000 |
| H | 4.775946000 | 2.476744000 | –7.192400000 |
| H | 6.036986000 | 3.564047000 | –5.331320000 |
| H | 5.319273000 | 4.786637000 | –6.401880000 |
| H | 4.929425000 | 5.368083000 | –3.999810000 |
| H | 3.200056000 | 6.005646000 | –5.697550000 |
| H | 2.448030000 | 5.643542000 | –4.128860000 |
| H | 1.191509000 | 4.531433000 | –5.974730000 |
| H | 3.108002000 | 4.304981000 | –7.577660000 |
| H | 2.296274000 | 2.744444000 | –7.326660000 |
| H | 4.953443000 | 3.055830000 | –3.067300000 |
| H | 3.460314000 | 3.898906000 | –2.604160000 |
| H | 1.301271000 | 3.428814000 | –3.767790000 |
| H | 1.216986000 | 2.218182000 | –5.060810000 |
| H | 0.867701000 | 1.772470000 | –2.765930000 |
| H | 1.101153000 | –1.637686000 | –2.765930000 |
| H | 2.318805000 | –2.841341000 | –3.767790000 |
| H | 1.312509000 | –2.163032000 | –5.060810000 |
| H | 3.328582000 | –3.297593000 | –5.974730000 |
| H | 3.601014000 | –5.774153000 | –5.697550000 |
| H | 3.663436000 | –4.941828000 | –4.128860000 |
| H | 2.184184000 | –6.953049000 | –3.999810000 |
| H | 0.068062000 | –7.010207000 | –5.331320000 |
| H | 1.485713000 | –6.999944000 | –6.401880000 |
| H | –0.243050000 | –5.374463000 | –7.192400000 |
| H | 2.174222000 | –4.844099000 | –7.577660000 |
| H | 1.228621000 | –3.360854000 | –7.326660000 |
| H | 0.169704000 | –5.817723000 | –3.067300000 |
| H | 1.646394000 | –4.946173000 | –2.604160000 |
| H | –0.799447000 | –3.381765000 | –5.787200000 |
| H | –1.276664000 | –4.899844000 | –5.002530000 |
| H | –2.021135000 | –4.537410000 | –3.123530000 |
| H | –3.295740000 | –5.547188000 | –1.656620000 |
| H | –3.188916000 | –4.981428000 | 0.022760000 |
| H | –5.180003000 | –6.451518000 | –0.322610000 |
| H | –6.902654000 | –4.891617000 | 0.616450000 |
| H | –5.315975000 | –4.629302000 | 1.370110000 |
| H | –6.355383000 | –2.445122000 | 0.744580000 |
| H | –6.771269000 | –1.919720000 | –1.663330000 |
| H | –7.760326000 | –3.304331000 | –1.148370000 |
| H | –6.654475000 | –3.760302000 | –3.349360000 |
| H | –7.052772000 | –5.676094000 | –1.784930000 |
| H | –5.573789000 | –5.928355000 | –2.736800000 |
| H | –3.882961000 | –2.608956000 | 0.626980000 |
| H | –4.505103000 | –1.528448000 | –0.631890000 |
| H | –4.180019000 | –3.947823000 | –3.472450000 |
| H | –4.659184000 | –2.301722000 | –3.018270000 |
| H | –2.428279000 | –1.283200000 | –0.375270000 |
| H | –1.968855000 | –0.134783000 | –2.765930000 |
| H | –3.620076000 | –0.587474000 | –3.767790000 |
| H | –2.529495000 | –0.055150000 | –5.060810000 |
| H | –4.520090000 | –1.233840000 | –5.974730000 |
| H | –6.801070000 | –0.231493000 | –5.697550000 |
| H | –6.111466000 | –0.701715000 | –4.128860000 |
| H | –7.113609000 | 1.584965000 | –3.999810000 |
| H | –6.105048000 | 3.446160000 | –5.331320000 |
| H | –6.804985000 | 2.213307000 | –6.401880000 |
| H | –4.532897000 | 2.897719000 | –7.192400000 |
| H | –5.282224000 | 0.539118000 | –7.577660000 |

| | | | |
|---|---|---|---|
| H | –3.524896000 | 0.616410000 | –7.326660000 |
| H | –5.123148000 | 2.761893000 | –3.067300000 |
| H | –5.106709000 | 1.047267000 | –2.604160000 |
| H | –2.528971000 | 2.383224000 | –5.787200000 |
| H | –3.605057000 | 3.555546000 | –5.002530000 |
| H | –2.918945000 | 4.019059000 | –3.123530000 |
| H | –0.317942000 | 4.667221000 | 0.626980000 |
| H | 0.928877000 | 4.665758000 | –0.631890000 |
| H | 1.060154000 | 6.726484000 | 0.744580000 |
| H | 1.723108000 | 6.823951000 | –1.663330000 |
| H | 1.018529000 | 8.372805000 | –1.148370000 |
| H | 0.070720000 | 7.643095000 | –3.349360000 |
| H | –1.389256000 | 8.945927000 | –1.784930000 |
| H | –2.347211000 | 7.791220000 | –2.736800000 |
| H | –2.997177000 | 7.711773000 | –0.322610000 |
| H | –0.784938000 | 8.423683000 | 0.616450000 |
| H | –1.351105000 | 6.918420000 | 1.370110000 |
| H | –1.328906000 | 5.593914000 | –3.472450000 |
| H | 0.336242000 | 5.185833000 | –3.018270000 |
| H | –3.156136000 | 5.627789000 | –1.656620000 |
| H | –2.719585000 | 5.252396000 | 0.022760000 |
| H | 0.102856000 | 2.744551000 | –0.375270000 |

## NMR computations

Symmetry for $^t$Bu-**1**$_2$ was restricted to $S_6$, for tetramethylsilane to $T_d$. Throughout cc-pVDZ was used as basis set.

**Table S18.** Tetramethylsilane NMR shifts computed with GIAO:B3LYP-D3(BJ)/6-31G(d,p) and C-PCM:cyclohexane.

| Atom (Count) | δ [ppm] |
|---|---|
| H(12) | 31.5159 |
| C(4) | 190.4201 |
| Si(1) | 411.4509 |

**Table S19.** $^t$Bu-**1**$_2$ NMR shifts computed with GIAO:B3LYP-D3(BJ)/6-31G(d,p) and C-PCM:cyclohexane in $S_6$ symmetry.

| Atom (Count) | δ [ppm] | δ(rel. to TMS) [ppm] |
|---|---|---|
| H(1) | 30.9663 | 0.5541 |
| H(1) | 30.9663 | 0.5541 |
| H(1) | 30.9659 | 0.5545 |
| H(1) | 30.9659 | 0.5545 |
| H(1) | 30.9656 | 0.5548 |
| H(1) | 30.9656 | 0.5548 |
| H(1) | 30.9170 | 0.6034 |
| H(1) | 30.9170 | 0.6034 |
| H(1) | 30.9165 | 0.6039 |
| H(1) | 30.9165 | 0.6039 |
| H(1) | 30.9160 | 0.6044 |
| H(1) | 30.9160 | 0.6044 |
| H(1) | 30.8679 | 0.6525 |
| H(1) | 30.8679 | 0.6525 |
| H(1) | 30.8678 | 0.6526 |
| H(1) | 30.8678 | 0.6526 |
| H(1) | 30.8671 | 0.6533 |
| H(1) | 30.8671 | 0.6533 |
| H(1) | 30.8216 | 0.6988 |
| H(1) | 30.8216 | 0.6988 |
| H(1) | 30.8206 | 0.6998 |
| H(1) | 30.8206 | 0.6998 |
| H(1) | 30.8204 | 0.7000 |
| H(1) | 30.8204 | 0.7000 |
| H(1) | 30.6704 | 0.8500 |
| H(1) | 30.6704 | 0.8500 |
| H(1) | 30.6700 | 0.8504 |
| H(1) | 30.6700 | 0.8504 |
| H(1) | 30.6695 | 0.8509 |
| H(1) | 30.6695 | 0.8509 |
| H(1) | 30.5653 | 0.9551 |
| H(1) | 30.5653 | 0.9551 |
| H(1) | 30.5649 | 0.9555 |
| H(1) | 30.5649 | 0.9555 |
| H(1) | 30.5628 | 0.9576 |
| H(1) | 30.5628 | 0.9576 |
| H(1) | 30.5531 | 0.9673 |
| H(1) | 30.5531 | 0.9673 |
| H(1) | 30.5529 | 0.9675 |
| H(1) | 30.5529 | 0.9675 |
| H(1) | 30.5528 | 0.9676 |
| H(1) | 30.5528 | 0.9676 |
| H(1) | 30.5365 | 0.9839 |
| H(1) | 30.5365 | 0.9839 |
| H(1) | 30.5361 | 0.9843 |
| H(1) | 30.5361 | 0.9843 |
| H(1) | 30.5351 | 0.9853 |
| H(1) | 30.5351 | 0.9853 |
| H(1) | 30.5336 | 0.9868 |
| H(1) | 30.5336 | 0.9868 |
| H(1) | 30.5327 | 0.9877 |
| H(1) | 30.5327 | 0.9877 |
| H(1) | 30.5319 | 0.9885 |
| H(1) | 30.5319 | 0.9885 |
| H(1) | 30.5271 | 0.9933 |
| H(1) | 30.5271 | 0.9933 |
| H(1) | 30.5265 | 0.9939 |
| H(1) | 30.5265 | 0.9939 |
| H(1) | 30.5265 | 0.9939 |
| H(1) | 30.5265 | 0.9939 |
| H(1) | 30.4849 | 1.0355 |
| H(1) | 30.4849 | 1.0355 |
| H(1) | 30.4847 | 1.0357 |
| H(1) | 30.4847 | 1.0357 |
| H(1) | 30.4838 | 1.0366 |
| H(1) | 30.4838 | 1.0366 |
| H(1) | 30.4392 | 1.0812 |
| H(1) | 30.4392 | 1.0812 |
| H(1) | 30.4374 | 1.0830 |
| H(1) | 30.4374 | 1.0830 |
| H(1) | 30.4369 | 1.0835 |
| H(1) | 30.4369 | 1.0835 |
| H(1) | 30.4200 | 1.1004 |
| H(1) | 30.4200 | 1.1004 |
| H(1) | 30.4191 | 1.1013 |
| H(1) | 30.4191 | 1.1013 |
| H(1) | 30.4185 | 1.1019 |
| H(1) | 30.4185 | 1.1019 |
| H(1) | 30.1966 | 1.3238 |
| H(1) | 30.1966 | 1.3238 |
| H(1) | 30.1951 | 1.3253 |
| H(1) | 30.1951 | 1.3253 |
| H(1) | 30.1944 | 1.3260 |
| H(1) | 30.1944 | 1.3260 |
| H(1) | 30.1400 | 1.3804 |
| H(1) | 30.1400 | 1.3804 |
| H(1) | 30.1330 | 1.3874 |
| H(1) | 30.1330 | 1.3874 |
| H(1) | 30.1240 | 1.3964 |
| H(1) | 30.1240 | 1.3964 |
| H(1) | 30.1109 | 1.4095 |
| H(1) | 30.1109 | 1.4095 |
| H(1) | 30.1105 | 1.4099 |
| H(1) | 30.1105 | 1.4099 |
| H(1) | 30.1100 | 1.4104 |
| H(1) | 30.1100 | 1.4104 |
| H(1) | 29.9754 | 1.5450 |

| | | | | | |
|---|---|---|---|---|---|
| H(1) | 29.9754 | 1.5450 | C(1) | 155.4680 | 34.9089 |
| H(1) | 29.9737 | 1.5467 | C(1) | 155.4680 | 34.9089 |
| H(1) | 29.9737 | 1.5467 | C(1) | 155.4555 | 34.9214 |
| H(1) | 29.9735 | 1.5469 | C(1) | 155.4555 | 34.9214 |
| H(1) | 29.9735 | 1.5469 | C(1) | 155.4487 | 34.9282 |
| H(1) | 29.9567 | 1.5637 | C(1) | 155.4487 | 34.9282 |
| H(1) | 29.9567 | 1.5637 | C(1) | 153.8288 | 36.5481 |
| H(1) | 29.9561 | 1.5643 | C(1) | 153.8288 | 36.5481 |
| H(1) | 29.9561 | 1.5643 | C(1) | 153.8134 | 36.5635 |
| H(1) | 29.9557 | 1.5647 | C(1) | 153.8134 | 36.5635 |
| H(1) | 29.9557 | 1.5647 | C(1) | 153.8109 | 36.5660 |
| H(1) | 25.2600 | 6.2604 | C(1) | 153.8109 | 36.5660 |
| H(1) | 25.2600 | 6.2604 | C(1) | 153.5193 | 36.8576 |
| H(1) | 25.2599 | 6.2605 | C(1) | 153.5193 | 36.8576 |
| H(1) | 25.2599 | 6.2605 | C(1) | 153.5190 | 36.8579 |
| H(1) | 25.2582 | 6.2622 | C(1) | 153.5190 | 36.8579 |
| H(1) | 25.2582 | 6.2622 | C(1) | 153.5189 | 36.8580 |
| H(1) | 23.9909 | 7.5295 | C(1) | 153.5189 | 36.8580 |
| H(1) | 23.9909 | 7.5295 | C(1) | 117.9551 | 72.4218 |
| H(1) | 23.9889 | 7.5315 | C(1) | 117.9551 | 72.4218 |
| H(1) | 23.9889 | 7.5315 | C(1) | 73.8151 | 116.5618 |
| H(1) | 23.9887 | 7.5317 | C(1) | 73.8151 | 116.5618 |
| H(1) | 23.9887 | 7.5317 | C(1) | 73.8040 | 116.5729 |
| H(1) | 23.3480 | 8.1724 | C(1) | 73.8040 | 116.5729 |
| H(1) | 23.3480 | 8.1724 | C(1) | 73.7991 | 116.5778 |
| H(1) | 23.3473 | 8.1731 | C(1) | 73.7991 | 116.5778 |
| H(1) | 23.3473 | 8.1731 | C(1) | 67.8038 | 122.5731 |
| H(1) | 23.3467 | 8.1737 | C(1) | 67.8038 | 122.5731 |
| H(1) | 23.3467 | 8.1737 | C(1) | 67.8001 | 122.5768 |
| C(1) | 161.7969 | 28.5800 | C(1) | 67.8001 | 122.5768 |
| C(1) | 161.7969 | 28.5800 | C(1) | 67.7977 | 122.5792 |
| C(1) | 161.7899 | 28.5870 | C(1) | 67.7977 | 122.5792 |
| C(1) | 161.7899 | 28.5870 | C(1) | 65.6515 | 124.7254 |
| C(1) | 161.7888 | 28.5881 | C(1) | 65.6515 | 124.7254 |
| C(1) | 161.7888 | 28.5881 | C(1) | 65.6460 | 124.7309 |
| C(1) | 161.4918 | 28.8851 | C(1) | 65.6460 | 124.7309 |
| C(1) | 161.4918 | 28.8851 | C(1) | 65.6456 | 124.7313 |
| C(1) | 161.4914 | 28.8855 | C(1) | 65.6456 | 124.7313 |
| C(1) | 161.4914 | 28.8855 | C(1) | 47.8934 | 142.4835 |
| C(1) | 161.4894 | 28.8875 | C(1) | 47.8934 | 142.4835 |
| C(1) | 161.4894 | 28.8875 | C(1) | 47.8902 | 142.4867 |
| C(1) | 158.8161 | 31.5608 | C(1) | 47.8902 | 142.4867 |
| C(1) | 158.8161 | 31.5608 | C(1) | 47.8836 | 142.4933 |
| C(1) | 158.8080 | 31.5689 | C(1) | 47.8836 | 142.4933 |
| C(1) | 158.8080 | 31.5689 | C(1) | 47.4038 | 142.9731 |
| C(1) | 158.8077 | 31.5692 | C(1) | 47.4038 | 142.9731 |
| C(1) | 158.8077 | 31.5692 | C(1) | 47.4007 | 142.9762 |
| C(1) | 157.1310 | 33.2459 | C(1) | 47.4007 | 142.9762 |
| C(1) | 157.1310 | 33.2459 | C(1) | 47.3977 | 142.9792 |
| C(1) | 157.1247 | 33.2522 | C(1) | 47.3977 | 142.9792 |
| C(1) | 157.1247 | 33.2522 | C(1) | 44.1428 | 146.2341 |
| C(1) | 157.1228 | 33.2541 | C(1) | 44.1428 | 146.2341 |
| C(1) | 157.1228 | 33.2541 | C(1) | 44.1387 | 146.2382 |
| C(1) | 156.8415 | 33.5354 | C(1) | 44.1387 | 146.2382 |
| C(1) | 156.8415 | 33.5354 | C(1) | 44.1367 | 146.2402 |
| C(1) | 156.8353 | 33.5416 | C(1) | 44.1367 | 146.2402 |
| C(1) | 156.8353 | 33.5416 | | | |
| C(1) | 156.8321 | 33.5448 | | | |
| C(1) | 156.8321 | 33.5448 | | | |

**Table S20.** Comparison of experimental and computed relative, per group averaged (GIAO:B3LYP-D3(BJ)/6-31G(d,p) and C-PCM:cyclohexane) chemical shifts of $^t$Bu-**1**$_2$.

| Group | ***δ***(Comp.) [ppm] | ***δ***(Found) [ppm] | Position[a)] |
|---|---|---|---|
| $C(C\mathbf{H}_3)_3$ | 1.01 | 0.95 | outside |
| $C(C\mathbf{H}_3)_3$ | 1.07 | 0.98 | inside |
| *o*-Ph C**H** | 6.26 | 6.03 | outside |
| *p*-Ph C**H** | 7.53 | 7.20 | |
| *o*-Ph C**H** | 8.17 | 7.76 | inside |
| $C(\mathbf{C}H_3)_3$ | 31.2 | 31.8 | outside |
| $C(\mathbf{C}H_3)_3$ | 32.4 | 32.1 | inside |
| $\mathbf{C}(CH_3)_3$ | 36.6 | 35.2 | inside |
| $\mathbf{C}(CH_3)_3$ | 36.9 | 35.3 | outside |
| $R_3\mathbf{CC}R_3$ | 72.4 | 71.5 | |
| *p*-Ph $\mathbf{C}_q$ | 116.6 | 120.3 | |
| *o*-Ph **C**H | 122.6 | 128.1 | outside |
| *o*-Ph **C**H | 124.7 | 128.6 | inside |
| *m*-Ph $\mathbf{C}_q$ | 142.5 | 149.5 | inside |
| *m*-Ph $\mathbf{C}_q$ | 143.0 | 149.9 | outside |
| *ipso*-Ph $\mathbf{C}_q$ | 146.2 | 151.1 | |

[a)] outside groups point towards a phenyl moiety within the same trityl group, inside groups point towards an opposite phenyl moiety of the other trityl group.

**Table S21.** Coordinates of optimized structures (in Å) of the NMR computation (GIAO:B3LYP-D3(BJ)/6-31G(d,p) with C–PCM:cyclohexane).

| | | | |
|---|---|---|---|
| | Tetramethylsilane | | |
| Si | 0.000000000 | 0.000000000 | 0.000000000 |
| C | 1.094250000 | –1.094250000 | –1.094250000 |
| C | –1.094250000 | 1.094250000 | –1.094250000 |
| C | 1.094250000 | 1.094250000 | 1.094250000 |
| C | –1.094250000 | –1.094250000 | 1.094250000 |
| H | 1.741910000 | –0.479960000 | –1.741910000 |
| H | 0.479960000 | –1.741910000 | –1.741910000 |
| H | 1.741910000 | –1.741910000 | –0.479960000 |
| H | –0.479960000 | 1.741910000 | –1.741910000 |
| H | –1.741910000 | 1.741910000 | –0.479960000 |
| H | –1.741910000 | 0.479960000 | –1.741910000 |
| H | 1.741910000 | 1.741910000 | 0.479960000 |
| H | 1.741910000 | 0.479960000 | 1.741910000 |
| H | 0.479960000 | 1.741910000 | 1.741910000 |
| H | –1.741910000 | –1.741910000 | 0.479960000 |
| H | –1.741910000 | –0.479960000 | 1.741910000 |
| H | –0.479960000 | –1.741910000 | 1.741910000 |
| | $^t$Bu-**1**$_2$ | | |
| C | 1.911499000 | –3.472015000 | 2.698540000 |
| C | 0.986957000 | –3.676676000 | 1.669860000 |
| C | 0.402744000 | –2.554050000 | 1.063440000 |
| C | 0.739967000 | –1.243947000 | 1.427110000 |
| C | 1.660030000 | –1.089278000 | 2.477180000 |
| C | 2.242159000 | –2.179358000 | 3.131650000 |
| C | –1.447273000 | –0.018857000 | 1.427110000 |
| C | –2.413244000 | 0.928239000 | 1.063440000 |
| C | –3.677573000 | 0.983608000 | 1.669860000 |
| C | –3.962603000 | 0.080600000 | 2.698540000 |
| C | –3.008459000 | –0.852088000 | 3.131650000 |
| C | –1.773358000 | –0.892989000 | 2.477180000 |
| C | 0.766300000 | 3.031446000 | 3.131650000 |
| C | 0.113328000 | 1.982267000 | 2.477180000 |
| C | 0.707306000 | 1.262803000 | 1.427110000 |
| C | 2.010501000 | 1.625812000 | 1.063440000 |
| C | 2.690616000 | 2.693068000 | 1.669860000 |
| C | 2.051103000 | 3.391415000 | 2.698540000 |
| C | 0.000000000 | 0.000000000 | 0.831070000 |
| C | –1.911499000 | 3.472015000 | –2.698540000 |
| C | –2.242159000 | 2.179358000 | –3.131650000 |
| C | –1.660030000 | 1.089278000 | –2.477180000 |
| C | –0.739967000 | 1.243947000 | –1.427110000 |
| C | –0.402744000 | 2.554050000 | –1.063440000 |
| C | –0.986957000 | 3.676676000 | –1.669860000 |
| C | –0.707306000 | –1.262803000 | –1.427110000 |
| C | –2.010501000 | –1.625812000 | –1.063440000 |
| C | –2.690616000 | –2.693068000 | –1.669860000 |
| C | –2.051103000 | –3.391415000 | –2.698540000 |
| C | –0.766300000 | –3.031446000 | –3.131650000 |
| C | –0.113328000 | –1.982267000 | –2.477180000 |
| C | 3.008459000 | 0.852088000 | –3.131650000 |
| C | 1.773358000 | 0.892989000 | –2.477180000 |
| C | 1.447273000 | 0.018857000 | –1.427110000 |
| C | 2.413244000 | –0.928239000 | –1.063440000 |
| C | 3.677573000 | –0.983608000 | –1.669860000 |
| C | 3.962603000 | –0.080600000 | –2.698540000 |
| C | 0.000000000 | 0.000000000 | –0.831070000 |
| C | 0.536089000 | –5.079268000 | 1.229520000 |
| C | 3.191212000 | –1.989189000 | 4.325610000 |
| C | –4.666819000 | 2.075367000 | 1.229520000 |
| C | –3.318295000 | –1.769076000 | 4.325610000 |
| C | 0.127082000 | 3.758266000 | 4.325610000 |
| C | 4.130730000 | 3.003901000 | 1.229520000 |
| C | –3.191212000 | 1.989189000 | –4.325610000 |
| C | –0.536089000 | 5.079268000 | –1.229520000 |
| C | –4.130730000 | –3.003901000 | –1.229520000 |
| C | –0.127082000 | –3.758266000 | –4.325610000 |
| C | 3.318295000 | 1.769076000 | –4.325610000 |
| C | 4.666819000 | –2.075367000 | –1.229520000 |
| C | 1.360340000 | –6.193245000 | 1.891930000 |
| C | 0.668878000 | –5.221130000 | –0.299340000 |
| C | –0.944230000 | –5.260916000 | 1.625360000 |
| C | 3.419728000 | –0.505757000 | 4.656760000 |
| C | 4.558752000 | –2.631990000 | 4.015880000 |
| C | 2.570730000 | –2.670920000 | 5.564200000 |
| C | –6.043678000 | 1.918534000 | 1.891930000 |
| C | –4.856070000 | 2.031299000 | –0.299340000 |
| C | –4.083972000 | 3.448185000 | 1.625360000 |
| C | –2.147862000 | –2.708693000 | 4.656760000 |
| C | –4.558746000 | –2.632000000 | 4.015880000 |

| | | | |
|---|---|---|---|
| C | –3.598449000 | –0.890858000 | 5.564200000 |
| C | –1.271866000 | 3.214450000 | 4.656760000 |
| C | –0.000006000 | 5.263990000 | 4.015880000 |
| C | 1.027719000 | 3.561777000 | 5.564200000 |
| C | 4.683338000 | 4.274712000 | 1.891930000 |
| C | 4.187192000 | 3.189831000 | –0.299340000 |
| C | 5.028202000 | 1.812731000 | 1.625360000 |
| C | –2.570730000 | 2.670920000 | –5.564200000 |
| C | –4.558752000 | 2.631990000 | –4.015880000 |
| C | –3.419728000 | 0.505757000 | –4.656760000 |
| C | –0.668878000 | 5.221130000 | 0.299340000 |
| C | –1.360340000 | 6.193245000 | –1.891930000 |
| C | 0.944230000 | 5.260916000 | –1.625360000 |
| C | –4.187192000 | –3.189831000 | 0.299340000 |
| C | –4.683338000 | –4.274712000 | –1.891930000 |
| C | –5.028202000 | –1.812731000 | –1.625360000 |
| C | –1.027719000 | –3.561777000 | –5.564200000 |
| C | 0.000006000 | –5.263990000 | –4.015880000 |
| C | 1.271866000 | –3.214450000 | –4.656760000 |
| C | 3.598449000 | 0.890858000 | –5.564200000 |
| C | 4.558746000 | 2.632000000 | –4.015880000 |
| C | 2.147862000 | 2.708693000 | –4.656760000 |
| C | 4.856070000 | –2.031299000 | 0.299340000 |
| C | 6.043678000 | –1.918534000 | –1.891930000 |
| C | 4.083972000 | –3.448185000 | –1.625360000 |
| H | 1.244975000 | –6.195168000 | 2.986770000 |
| H | 2.432434000 | –6.096712000 | 1.658870000 |
| H | 1.022202000 | –7.173643000 | 1.521880000 |
| H | 0.098757000 | –4.452471000 | –0.838000000 |
| H | 0.294738000 | –6.205387000 | –0.624830000 |
| H | 1.721935000 | –5.139586000 | –0.610170000 |
| H | –1.067307000 | –5.202074000 | 2.718390000 |
| H | –1.317893000 | –6.242667000 | 1.290290000 |
| H | –1.575105000 | –4.483138000 | 1.171750000 |
| H | 4.113164000 | –0.419537000 | 5.507700000 |
| H | 2.483104000 | 0.002076000 | 4.934840000 |
| H | 3.859202000 | 0.038064000 | 3.807760000 |
| H | 5.229127000 | –2.542830000 | 4.886470000 |
| H | 5.039292000 | –2.133758000 | 3.160380000 |
| H | 4.461671000 | –3.701215000 | 3.773710000 |
| H | 3.219500000 | –2.533117000 | 6.444930000 |
| H | 2.437096000 | –3.752362000 | 5.409400000 |
| H | 1.583405000 | –2.237874000 | 5.792320000 |
| H | –5.987661000 | 2.019404000 | 2.986770000 |
| H | –6.496124000 | 0.941807000 | 1.658870000 |
| H | –6.723658000 | 2.701569000 | 1.521880000 |
| H | –3.905332000 | 2.140709000 | –0.838000000 |
| H | –5.521392000 | 2.847443000 | –0.624830000 |
| H | –5.311980000 | 1.078553000 | –0.610170000 |
| H | –3.971475000 | 3.525352000 | 2.718390000 |
| H | –4.747361000 | 4.262663000 | 1.290290000 |
| H | –3.094959000 | 3.605650000 | 1.171750000 |
| H | –2.419911000 | –3.352336000 | 5.507700000 |
| H | –1.239754000 | –2.151469000 | 4.934840000 |
| H | –1.896636000 | –3.361199000 | 3.807760000 |
| H | –4.816719000 | –3.257142000 | 4.886470000 |
| H | –4.367535000 | –3.297276000 | 3.160380000 |
| H | –5.436182000 | –2.013313000 | 3.773710000 |
| H | –3.803494000 | –1.521610000 | 6.444930000 |
| H | –4.468189000 | –0.234406000 | 5.409400000 |
| H | –2.729758000 | –0.252332000 | 5.792320000 |
| H | –1.693253000 | 3.771873000 | 5.507700000 |
| H | –1.243349000 | 2.149393000 | 4.934840000 |
| H | –1.962565000 | 3.323135000 | 3.807760000 |
| H | –0.412408000 | 5.799972000 | 4.886470000 |
| H | –0.671757000 | 5.431034000 | 3.160380000 |
| H | 0.974511000 | 5.714528000 | 3.773710000 |
| H | 0.583994000 | 4.054727000 | 6.444930000 |
| H | 2.031093000 | 3.986768000 | 5.409400000 |
| H | 1.146353000 | 2.490206000 | 5.792320000 |
| H | 4.742685000 | 4.175764000 | 2.986770000 |
| H | 4.063691000 | 5.154905000 | 1.658870000 |
| H | 5.701456000 | 4.472075000 | 1.521880000 |
| H | 3.806575000 | 2.311762000 | –0.838000000 |
| H | 5.226654000 | 3.357944000 | –0.624830000 |
| H | 3.590044000 | 4.061033000 | –0.610170000 |
| H | 5.038782000 | 1.676722000 | 2.718390000 |
| H | 6.065255000 | 1.980004000 | 1.290290000 |
| H | 4.670064000 | 0.877488000 | 1.171750000 |
| H | –2.437096000 | 3.752362000 | –5.409400000 |
| H | –3.219500000 | 2.533117000 | –6.444930000 |
| H | –1.583405000 | 2.237874000 | –5.792320000 |
| H | –4.461671000 | 3.701215000 | –3.773710000 |
| H | –5.039292000 | 2.133758000 | –3.160380000 |
| H | –5.229127000 | 2.542830000 | –4.886470000 |
| H | –3.859202000 | –0.038064000 | –3.807760000 |
| H | –2.483104000 | –0.002076000 | –4.934840000 |
| H | –4.113164000 | 0.419537000 | –5.507700000 |
| H | –0.294738000 | 6.205387000 | 0.624830000 |
| H | –0.098757000 | 4.452471000 | 0.838000000 |
| H | –1.721935000 | 5.139586000 | 0.610170000 |
| H | –1.022202000 | 7.173643000 | –1.521880000 |
| H | –2.432434000 | 6.096712000 | –1.658870000 |
| H | –1.244975000 | 6.195168000 | –2.986770000 |
| H | 1.317893000 | 6.242667000 | –1.290290000 |
| H | 1.067307000 | 5.202074000 | –2.718390000 |
| H | 1.575105000 | 4.483138000 | –1.171750000 |
| H | –5.226654000 | –3.357944000 | 0.624830000 |
| H | –3.806575000 | –2.311762000 | 0.838000000 |
| H | –3.590044000 | –4.061033000 | 0.610170000 |
| H | –5.701456000 | –4.472075000 | –1.521880000 |
| H | –4.063691000 | –5.154905000 | –1.658870000 |
| H | –4.742685000 | –4.175764000 | –2.986770000 |
| H | –6.065255000 | –1.980004000 | –1.290290000 |
| H | –5.038782000 | –1.676722000 | –2.718390000 |
| H | –4.670064000 | –0.877488000 | –1.171750000 |
| H | –2.031093000 | –3.986768000 | –5.409400000 |
| H | –0.583994000 | –4.054727000 | –6.444930000 |
| H | –1.146353000 | –2.490206000 | –5.792320000 |
| H | –0.974511000 | –5.714528000 | –3.773710000 |
| H | 0.671757000 | –5.431034000 | –3.160380000 |
| H | 0.412408000 | –5.799972000 | –4.886470000 |
| H | 1.962565000 | –3.323135000 | –3.807760000 |
| H | 1.243349000 | –2.149393000 | –4.934840000 |
| H | 1.693253000 | –3.771873000 | –5.507700000 |
| H | 4.468189000 | 0.234406000 | –5.409400000 |
| H | 3.803494000 | 1.521610000 | –6.444930000 |
| H | 2.729758000 | 0.252332000 | –5.792320000 |
| H | 5.436182000 | 2.013313000 | –3.773710000 |
| H | 4.367535000 | 3.297276000 | –3.160380000 |
| H | 4.816719000 | 3.257142000 | –4.886470000 |
| H | 1.896636000 | 3.361199000 | –3.807760000 |
| H | 1.239754000 | 2.151469000 | –4.934840000 |
| H | 2.419911000 | 3.352336000 | –5.507700000 |
| H | 5.521392000 | –2.847443000 | 0.624830000 |
| H | 3.905332000 | –2.140709000 | 0.838000000 |
| H | 5.311980000 | –1.078553000 | 0.610170000 |
| H | 6.723658000 | –2.701569000 | –1.521880000 |
| H | 6.496124000 | –0.941807000 | –1.658870000 |
| H | 5.987661000 | –2.019404000 | –2.986770000 |
| H | 4.747361000 | –4.262663000 | –1.290290000 |
| H | 3.971475000 | –3.525352000 | –2.718390000 |
| H | 3.094959000 | –3.605650000 | –1.171750000 |
| H | 2.365756000 | –4.328560000 | 3.192610000 |
| H | –0.351453000 | –2.715799000 | 0.304750000 |
| H | 1.913196000 | –0.092396000 | 2.810390000 |
| H | –2.176224000 | 1.662267000 | 0.304750000 |
| H | –4.931521000 | 0.115476000 | 3.192610000 |
| H | –1.036615000 | –1.610678000 | 2.810390000 |
| H | –0.876581000 | 1.703074000 | 2.810390000 |
| H | 2.527677000 | 1.053532000 | 0.304750000 |
| H | 2.565766000 | 4.213085000 | 3.192610000 |
| H | –2.365756000 | 4.328560000 | –3.192610000 |
| H | –1.913196000 | 0.092396000 | –2.810390000 |
| H | 0.351453000 | 2.715799000 | –0.304750000 |
| H | –2.527677000 | –1.053532000 | –0.304750000 |
| H | –2.565766000 | –4.213085000 | –3.192610000 |
| H | 0.876581000 | –1.703074000 | –2.810390000 |
| H | 1.036615000 | 1.610678000 | –2.810390000 |
| H | 2.176224000 | –1.662267000 | –0.304750000 |
| H | 4.931521000 | –0.115476000 | –3.192610000 |

## Estimation of the covalent contribution of the overall dissociation energy

### Estimation from heat of formation

To estimate the covalent contribution (BDE) of the dissociation energy of HPE into two TPM radicals from heats of formation ($\Delta H_f^0$) a reference molecule needed to be chosen. The common example for exclusively covalent element–element bonds are the simples stable representative molecules which bare this bond type. In case of a carbon–carbon single bond this is ethane **13**$_2$. The BDEs were derived for the carbon–carbon dissociations of **13**$_2$ and $^t$Bu-**1**$_2$ (Scheme S2). The unknown $\Delta H_f^0$ of $^t$Bu-**1**$_2$ and $^t$Bu-**1•** were calculated from isodesmic reactions (Scheme S4). Reaction enthalpies were derived from optimized structures with B3YLP-D3(BJ)/cc-pVDZ computations. Heats of formation of ethane **13**$_2$, methyl radical **13•**, methane **14**, triphenylmethane **15**, 2,2,3,3-tetramethylbutane **16** and *tert*-butyl radical **17** are listed in Table S24. Here 53% of covalent bonding are left in $^t$Bu-**1**$_2$ regarding **13**$_2$.

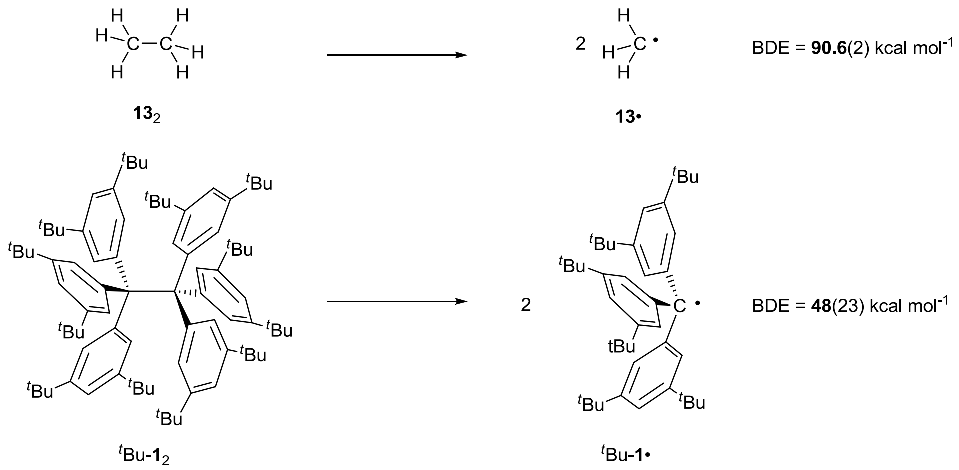


**Scheme S2.** The strength of the central bond estimated for **12**$_2$ and $^t$Bu-**1**$_2$.

### Estimation from enthalpy of hydrogenation

Likewise the determination of aromatization energy *via* the enthalpy of hydrogenation, can the theoretical hydrogenation of a carbon–carbon single bonds be utilized to estimate the covalent bond strength upon stretching the bond relative to ethane. Bond dissociation energies $\Delta H_d^{298}$ were derived from optimized structures with B3YLP-D3(BJ)/cc-pVDZ computations (Table S25 and Table S26). Regarding the hydrogenation of $^t$Bu-**1**$_2$ via $\Delta H_d^{298}$ remain 56%, *via* $\Delta H_f^{298}$ remain 14% of covalent bonding strength (Scheme S3).

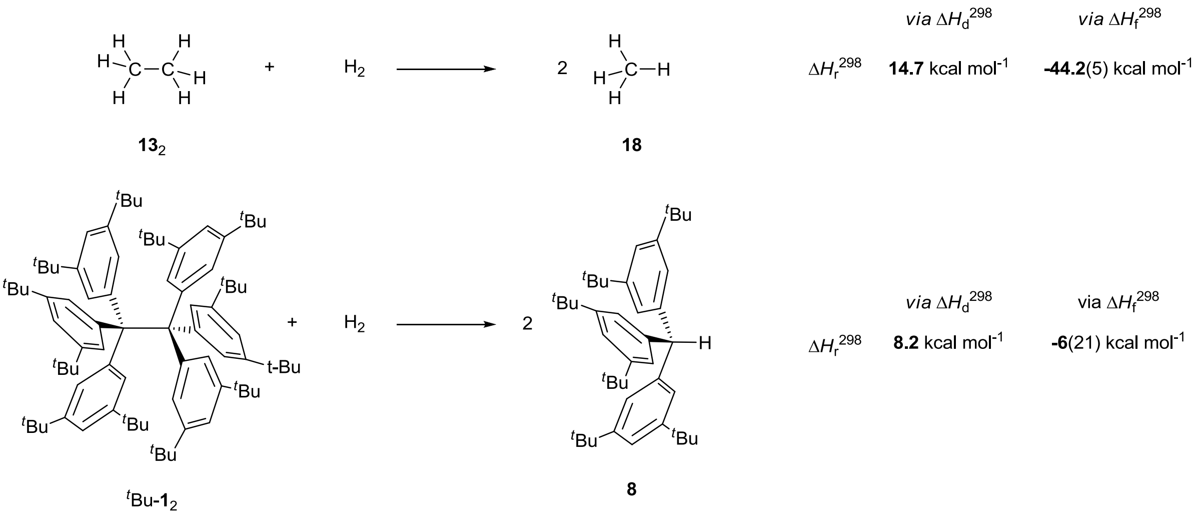


**Scheme S3.** Hydrogenation of ethanes derivatives leads to methanes.

## Estimation from bond vibration

Regarding Hooke's law depend the frequency of a harmonic oscillator on the reduced mass $\mu$ and the stiffness $K_H$. This stiffness correlates direct with bond strength and can therefore serve as a gauge for the covalent contribution relative to ethane. Due to the symmetric weight distribution is the reduced mass deduced from the half molecular weight. The specific symmetric bond stretching frequency $v_s$(C–C) was obtained from B3YLP-D3(BJ)/cc-pVDZ frequency computations ($^t$Bu-**1**$_2$:Table S12 and Table S16 /**13**$_2$:Table S25 and Table S26). Regarding **13**$_2$ were 46% bond strength in $^t$Bu-**1**$_2$ preserved.

**Table S22**. Specifications of the harmonic oscillators **12**$_2$ and $^t$Bu-**1**$_2$.

| Compound | Half mass (u) | $\mu$ (g) | $\tilde{v}$ (cm$^{-1}$) | $v$ (GHz) | $K_H$ (nN m) |
|---|---|---|---|---|---|
| **13**$_2$ | 15.02 | 0.13 10$^{-22}$ | 1013 | 304.7 | 45.4 |
| $^t$Bu-**1**$_2$ | 579.49 | 4.81 10$^{-22}$ | 111 | 33.3 | 21.0 |

## Estimation via ISAPT computations

The intramolecular SAPT method allowed Sherrill *et al.* to compute the non-covalent contributions within $^t$Bu-**1**$_2$.[9] The total non-covalent energy was determined to be repulsive by $E^{\text{non-cov.}}{}_{\text{tot}}$ = –44.97 kcal mol$^{-1}$. Herein the overall dissociation enthalpy $\Delta H_d^{298}$ = 7.94(3) kcal mol$^{-1}$ was determined. A ruff estimation of the covalent contribution is obtained by subtracting all non-covalent contributions from the overall enthalpy. This give 52.91 kcal mol$^{-1}$; 59% of the BDE for ethane.

## Conclusions

On one hand are these estimations no quantitative calculation of the true covalent contribution but rather a qualitative analysis of how much covalent bonding should be expected. The error bars for the heat of formations are quite large and further are attractive interactions lost during the reaction. As these attractions are only within $^t$Bu-**1**$_2$ and on side of the starting materials is the determined bond strength likely too strong. This loss of interactions accounts also for the enthalpy of hydrogenation. The bond strength from hydrogenation *via* $\Delta H_d^{298}$ should appear overestimated, too. The bond strength from hydrogenation *via* $\Delta H_f^{298}$ bares for $^t$Bu-**1**$_2$ an uncertainty of ±21 kcal mol$^{-1}$, more than three times larger than the value. The estimation from bond vibrations depends on an isolated pure $v_s$(C–C). This indeed is obtained for ethane. In case of $^t$Bu-**1**$_2$ instead little mixing with wagging, rocking and deformation vibrations cannot be avoided. In the estimation from the ISAPT computation are energies mixed with enthalpies. In general appear energies to a little stronger relative to the enthalpies. Hence are these derived bond strength likely to be overestimated.

On the other hand, if the heat of hydrogenation *via* $\Delta H_f^{298}$ is because of the prohibitive uncertainty omitted match the here estimated covalent bond strengths roughly and are placed between 46% and 59% regarding ethane as reference system. This gives an averaged BDE of 48(+5/–7) kcal mol$^{-1}$ which should be taken as an upper limit. The 0.13 Å elongation of the central bond in $^t$Bu-**1**$_2$ at least halve its strength, it still appears to be essential to the overall stability of $^t$Bu-**1**$_2$ against dissociation.

## Computational details for estimations of covalent C–C bond contribution

*Dissociation energies, heats of formation and isodesmic reactions*

**Table S23.** Computed dissociation enthalpies with B3YLP-D3(BJ)/cc-pVDZ.

| Compound | Concerned Bond | $\Delta H_d^{298}$ (kcal mol$^{-1}$) |
|---|---|---|
| $H_2$ | H–H | 99.14 |
| **13**$_2$ | $H_3C–CH_3$ | 89.17 |
| **18** | $H_3C–H$ | 101.54 |
| $^t$Bu-**1**$_2$ | $Ar_3C–CAr_3$ | 37.96 |
| **8** | $Ar_3C–H$ | 72.64 |

**Table S24.** Calculated and literature known heats of formation.

| Compound | $\Delta H_f^{298}$ (kcal mol$^{-1}$) | Reference |
|---|---|---|
| $^t$Bu-**1**$_2$ | –198(11) | |
| $^t$Bu-**1•** | –75(6) | |
| **8** | –102(5) | |
| **13**$_2$ | –20.04(7) | [10] |
| **13•** | 35.3(5) | [11] |
| **14** | –32.1(2) | [10] |
| **15** | 66(2) | [12] |
| **16** | –54.0(3) | [13] |
| **17** | 12.3(4) | [14] |

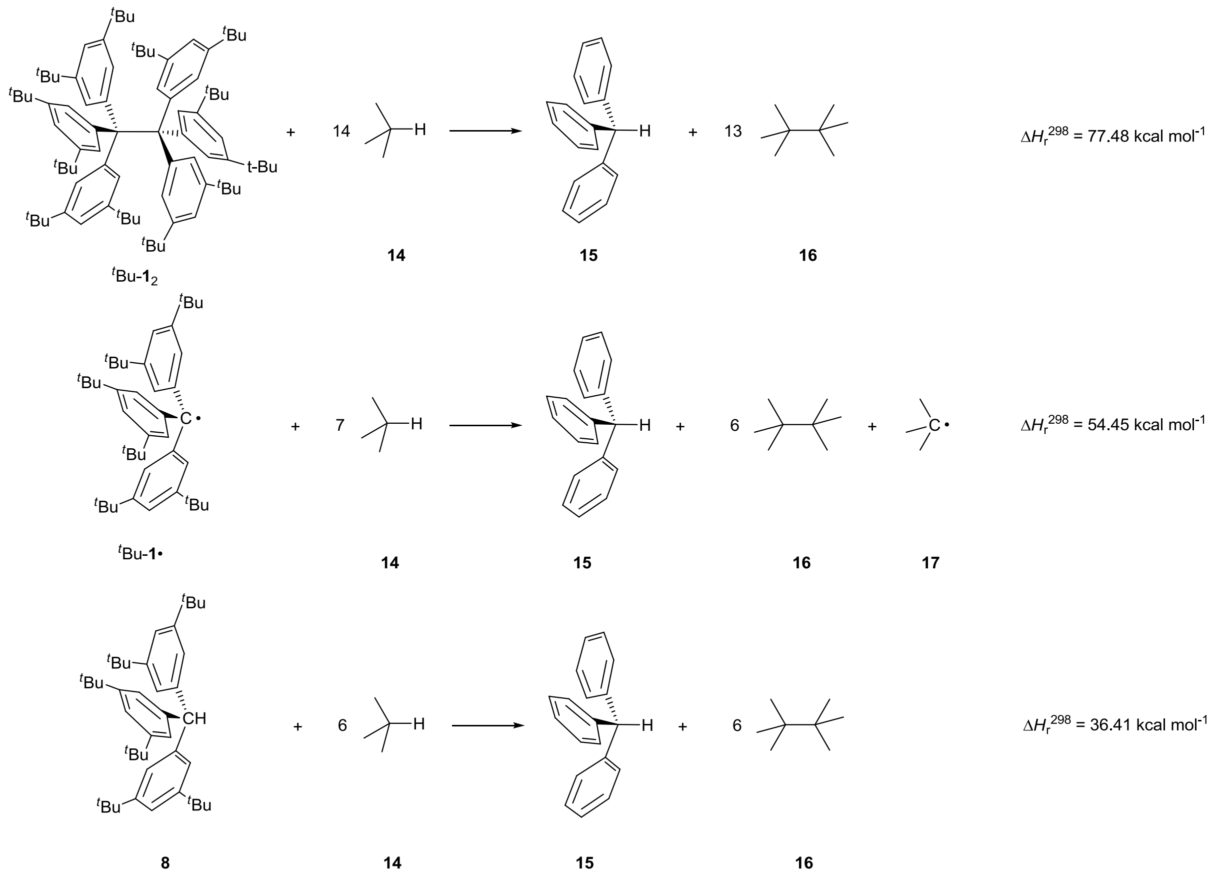


**Scheme S4.** The isodesmic reactions balance the occurrence of bonding situations in starting materials and products to minimize errors.

.

*Energies*

Energies of the optimized structures are given in hartree. Throughout cc-pVDZ was used as basis set. Energies and Cartesian coordinates of $^{t}$Bu-$\mathbf{1}_2$ and $^{t}$Bu-**1**• are listed in section "computations".

**Table S25.** Computed energies from B3YLP-D3(BJ)/cc-pVDZ.

| Compound | EE | ZPVE | $H^{298}$ |
|---|---|---|---|
| H• | –0.501258 | 0 | –0.501258 |
| $H_2$ | –1.173745 | 0.009933 | –1.160508 |
| **8** | –1677.480586 | 0.958430 | –1676.474814 |
| **13•** | –39.840451 | 0.029368 | –39.807036 |
| $\mathbf{13}_2$ | –79.834689 | 0.074095 | –79.756170 |
| **14** | –158.474946 | 0.130565 | –158.337727 |
| **15** | –315.747236 | 0.242372 | –315.493939 |
| **16** | –733.785857 | 0.290371 | –733.479513 |
| **17** | –157.814456 | 0.115387 | –157.691690 |
| **18** | –40.518262 | 0.044344 | –40.470101 |

**Table S26.** Cartesian coordinates of the optimized structures with B3YLP-D3(BJ)/cc-pVDZ.

$H_2$

| | | | |
|---|---|---|---|
| H | 0.000000000 | 0.000000000 | 0.380913000 |
| H | 0.000000000 | 0.000000000 | –0.380913000 |

**13•**

| | | | |
|---|---|---|---|
| C | 0.000000000 | 0.000000000 | 0.000000000 |
| H | 0.000000000 | 1.090744000 | –0.000001000 |
| H | –0.944612000 | –0.545372000 | –0.000001000 |
| H | 0.944612000 | –0.545372000 | –0.000001000 |

**$13_2$**

| | | | |
|---|---|---|---|
| C | 0.000000000 | 0.000000000 | 0.764285000 |
| C | 0.000000000 | 0.000000000 | –0.764285000 |
| H | 0.000000000 | 1.026317000 | 1.167586000 |
| H | 0.888816000 | –0.513158000 | 1.167586000 |
| H | –0.888816000 | –0.513158000 | 1.167586000 |
| H | 0.888816000 | 0.513158000 | –1.167586000 |
| H | –0.888816000 | 0.513158000 | –1.167586000 |
| H | 0.000000000 | –1.026317000 | –1.167586000 |

**18**

| | | | |
|---|---|---|---|
| C | 0.000000000 | 0.000000000 | 0.000000000 |
| H | 0.635031000 | 0.635031000 | 0.635031000 |
| H | –0.635031000 | –0.635031000 | 0.635031000 |
| H | 0.635031000 | –0.635031000 | –0.635031000 |
| H | –0.635031000 | 0.635031000 | –0.635031000 |

**8**

| | | | |
|---|---|---|---|
| C | 0.000000000 | 4.084454000 | –0.113448000 |
| C | –0.751852000 | 3.742103000 | 1.021837000 |
| C | –0.701123000 | 2.415130000 | 1.463497000 |
| C | 0.069141000 | 1.456593000 | 0.795056000 |
| C | 0.814062000 | 1.835598000 | –0.324097000 |
| C | 0.790224000 | 3.153440000 | –0.799453000 |
| C | –1.296017000 | –0.668419000 | 0.795056000 |
| C | –1.741003000 | –1.814755000 | 1.463497000 |
| C | –2.864831000 | –2.522174000 | 1.021837000 |
| C | –3.537241000 | –2.042227000 | –0.113448000 |
| C | –3.126071000 | –0.892366000 | –0.799453000 |
| C | –1.996706000 | –0.212801000 | –0.324097000 |
| C | 2.335847000 | –2.261074000 | –0.799453000 |
| C | 1.182644000 | –1.622798000 | –0.324097000 |
| C | 1.226876000 | –0.788174000 | 0.795056000 |
| C | 2.442125000 | –0.600375000 | 1.463497000 |
| C | 3.616682000 | –1.219929000 | 1.021837000 |
| C | 3.537241000 | –2.042227000 | –0.113448000 |
| C | 0.000000000 | 0.000000000 | 1.246926000 |
| C | –1.604935000 | 4.814070000 | 1.717384000 |
| C | 1.583607000 | 3.586145000 | –2.041465000 |
| C | –3.366639000 | –3.796949000 | 1.717384000 |
| C | –3.897496000 | –0.421629000 | –2.041465000 |
| C | 2.313889000 | –3.164516000 | –2.041465000 |
| C | 4.971574000 | –1.017121000 | 1.717384000 |
| C | –0.697877000 | 5.973975000 | 2.178291000 |
| C | –2.344121000 | 4.262056000 | 2.946161000 |
| C | –2.656253000 | 5.346558000 | 0.720527000 |
| C | 2.419114000 | 2.435347000 | –2.626088000 |
| C | 2.542358000 | 4.734918000 | –1.665537000 |
| C | 0.597578000 | 4.070472000 | –3.125459000 |
| C | –4.824676000 | –3.591367000 | 2.178291000 |
| C | –2.518988000 | –4.161097000 | 2.946161000 |
| C | –3.302129000 | –4.973662000 | 0.720527000 |
| C | –3.318630000 | 0.877341000 | –2.626088000 |
| C | –5.371739000 | –0.165712000 | –1.665537000 |
| C | –3.823921000 | –1.517718000 | –3.125459000 |
| C | 0.899516000 | –3.312688000 | –2.626088000 |
| C | 2.829380000 | –4.569206000 | –1.665537000 |
| C | 3.226343000 | –2.552754000 | –3.125459000 |
| C | 5.522552000 | –2.382608000 | 2.178291000 |
| C | 4.863110000 | –0.100960000 | 2.946161000 |
| C | 5.958382000 | –0.372896000 | 0.720527000 |
| H | –0.177398000 | 6.445514000 | 1.331270000 |
| H | 0.067060000 | 5.614176000 | 2.884541000 |
| H | –1.294506000 | 6.751921000 | 2.682616000 |

**14**

| | | | |
|---|---|---|---|
| C | 0.000000000 | 0.000000000 | 0.375215000 |
| C | 0.000000000 | 1.458123000 | –0.095757000 |
| H | 0.891172000 | 1.997885000 | 0.264481000 |
| H | –0.891172000 | 1.997885000 | 0.264481000 |
| H | 0.000000000 | 1.511787000 | –1.198838000 |
| C | 1.262772000 | –0.729062000 | –0.095757000 |
| H | 2.175805000 | –0.227165000 | 0.264481000 |
| H | 1.309246000 | –0.755893000 | –1.198838000 |
| H | 1.284633000 | –1.770720000 | 0.264481000 |
| C | –1.262772000 | –0.729062000 | –0.095757000 |
| H | –1.309246000 | –0.755893000 | –1.198838000 |
| H | –2.175805000 | –0.227165000 | 0.264481000 |
| H | –1.284633000 | –1.770720000 | 0.264481000 |
| H | 0.000000000 | 0.000000000 | 1.481967000 |

**16**

| | | | |
|---|---|---|---|
| C | 0.000000000 | 0.000000000 | 0.793523000 |
| C | –1.243450000 | 0.717906000 | 1.355812000 |
| H | –2.179960000 | 0.231073000 | 1.045880000 |
| H | –1.289079000 | 1.772818000 | 1.047322000 |
| H | –1.215832000 | 0.700915000 | 2.457258000 |
| C | 0.000000000 | –1.435813000 | 1.355812000 |
| H | –0.890766000 | –2.002784000 | 1.047322000 |
| H | 0.000905000 | –1.403399000 | 2.457258000 |
| H | 0.889865000 | –2.003437000 | 1.045880000 |
| C | 1.243450000 | 0.717906000 | 1.355812000 |
| H | 1.214926000 | 0.702483000 | 2.457258000 |
| H | 1.290095000 | 1.772364000 | 1.045880000 |
| H | 2.179845000 | 0.229966000 | 1.047322000 |
| C | 0.000000000 | 0.000000000 | –0.793523000 |
| C | 0.000000000 | 1.435813000 | –1.355812000 |
| H | –0.889865000 | 2.003437000 | –1.045880000 |
| H | –0.000905000 | 1.403399000 | –2.457258000 |
| H | 0.890766000 | 2.002784000 | –1.047322000 |
| C | 1.243450000 | –0.717906000 | –1.355812000 |
| H | 1.215832000 | –0.700915000 | –2.457258000 |
| H | 1.289079000 | –1.772818000 | –1.047322000 |
| H | 2.179960000 | –0.231073000 | –1.045880000 |
| C | –1.243450000 | –0.717906000 | –1.355812000 |
| H | –1.214926000 | –0.702483000 | –2.457258000 |
| H | –2.179845000 | –0.229966000 | –1.047322000 |
| H | –1.290095000 | –1.772364000 | –1.045880000 |

**17**

| | | | |
|---|---|---|---|
| C | 0.000000000 | 1.485165000 | –0.014961000 |
| C | 0.000000000 | 0.000000000 | 0.157327000 |
| H | –0.894330000 | 1.950658000 | 0.432669000 |
| H | 0.000000000 | 1.775719000 | –1.090228000 |
| H | 0.894330000 | 1.950658000 | 0.432669000 |
| C | 1.286191000 | –0.742583000 | –0.014961000 |
| H | 1.242155000 | –1.749841000 | 0.432669000 |
| H | 2.136484000 | –0.200817000 | 0.432669000 |
| H | 1.537818000 | –0.887860000 | –1.090228000 |
| C | –1.286191000 | –0.742583000 | –0.014961000 |

| | | | |
|---|---|---|---|
| H | –1.537818000 | –0.887860000 | –1.090228000 |
| H | –2.136484000 | –0.200817000 | 0.432669000 |
| H | –1.242155000 | –1.749841000 | 0.432669000 |

**15**

| | | | |
|---|---|---|---|
| C | 0.121253000 | 4.170479000 | –0.422976000 |
| C | –0.654890000 | 3.264296000 | –1.148034000 |
| C | –0.717122000 | 1.921104000 | –0.762902000 |
| C | 0.000000000 | 1.464615000 | 0.349596000 |
| C | 0.776113000 | 2.383390000 | 1.071583000 |
| C | 0.837602000 | 3.724436000 | 0.692471000 |
| C | –1.268394000 | –0.732308000 | 0.349596000 |
| C | –2.452133000 | –0.519561000 | 1.071583000 |
| C | –3.644257000 | –1.136833000 | 0.692471000 |
| C | –3.672368000 | –1.980231000 | –0.422976000 |
| C | –2.499518000 | –2.199300000 | –1.148034000 |
| C | –1.305164000 | –1.581598000 | –0.762902000 |
| C | 3.154409000 | –1.064997000 | –1.148034000 |
| C | 2.022286000 | –0.339506000 | –0.762902000 |
| C | 1.268394000 | –0.732308000 | 0.349596000 |
| C | 1.676020000 | –1.863828000 | 1.071583000 |
| C | 2.806655000 | –2.587602000 | 0.692471000 |
| C | 3.551114000 | –2.190248000 | –0.422976000 |
| C | 0.000000000 | 0.000000000 | 0.777683000 |
| H | 0.165516000 | 5.219977000 | –0.721527000 |
| H | –1.221518000 | 3.602682000 | –2.018442000 |
| H | –1.333752000 | 1.221895000 | –1.329264000 |
| H | 1.344417000 | 2.036814000 | 1.938630000 |
| H | 1.444680000 | 4.425144000 | 1.270043000 |
| H | –2.436141000 | 0.145892000 | 1.938630000 |
| H | –4.554627000 | –0.961443000 | 1.270043000 |
| H | –4.603391000 | –2.466647000 | –0.721527000 |
| H | –2.509255000 | –2.859207000 | –2.018442000 |
| H | –0.391316000 | –1.766011000 | –1.329264000 |
| H | 3.730773000 | –0.743475000 | –2.018442000 |
| H | 1.725068000 | 0.544116000 | –1.329264000 |
| H | 1.091724000 | –2.182706000 | 1.938630000 |
| H | 3.109948000 | –3.463701000 | 1.270043000 |
| H | 4.437875000 | –2.753330000 | –0.721527000 |
| H | 0.000000000 | 0.000000000 | 1.880300000 |

## Appendix

### Thermodynamic data of C–C bond dissociations equilibria

An enthalpy–entropy compensation is found for dissociation of C–C single bonds in solution with low $\Delta H_d^{298}$ (below 15 kcal mol$^{-1}$) in an ensemble of 36 hydrocarbons (Table S27 and Figure S4). Here the $\Delta H_d^{298}$ ranges from 6.2 kcal mol$^{-1}$ to 67.4 kcal mol$^{-1}$. The term $T\Delta S_d^{298}$ at 298 K instead varies around Ø($T\Delta S_d^{298}$) = 6(3) kcal mol$^{-1}$. In this ensemble occurs the compensation phenomenon only in systems with a very low carbon–carbon bond dissociation energy.

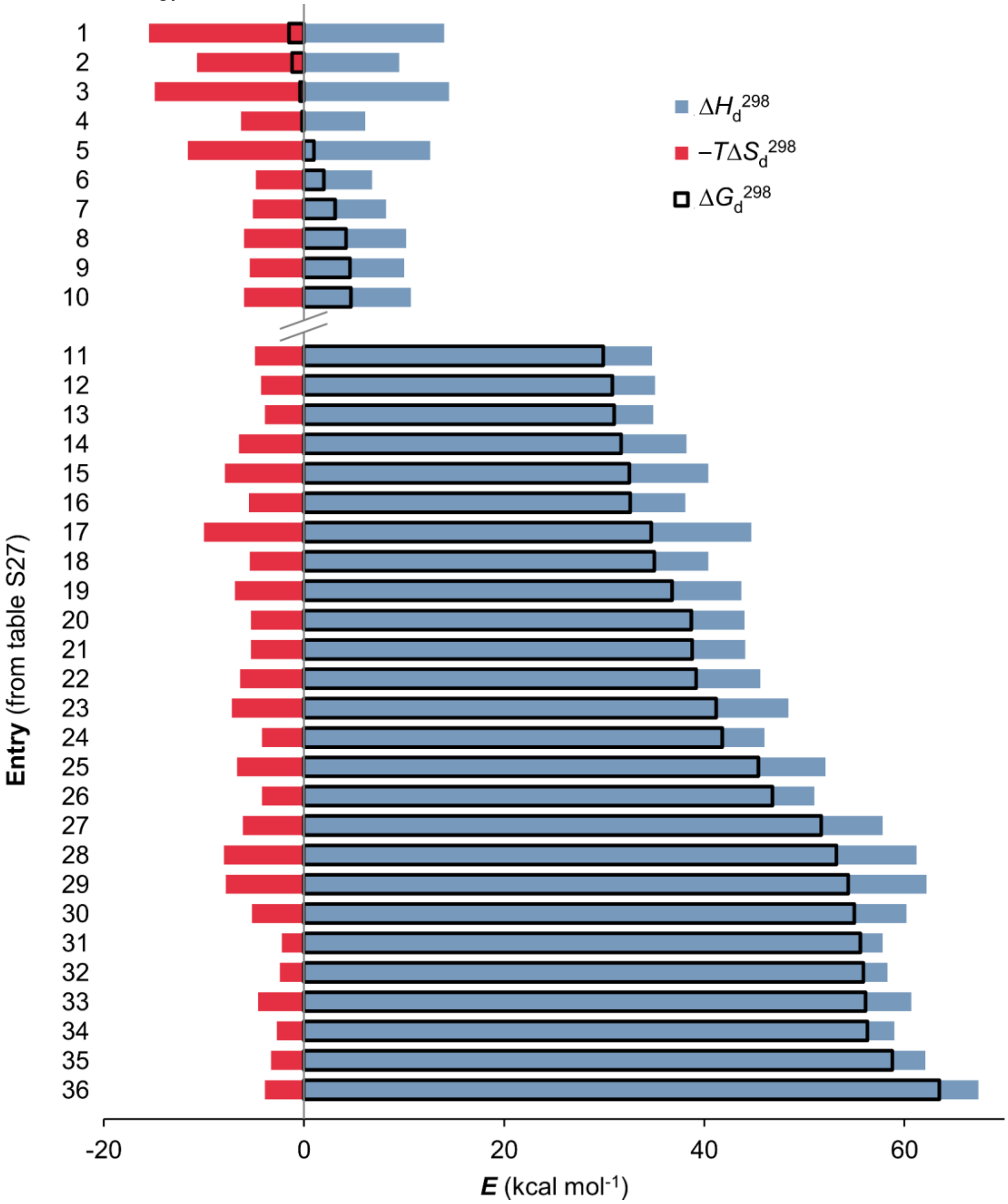


**Figure S4.** Thermodynamic data from table S27 reveal an enthalpy–entropy compensation for low $\Delta H_d^{298}$. With rising $\Delta H_d^{298}$ becomes the term $T\Delta S_d^{298}$ more negligible.

**Table S27.** A list of homolytic C–C bond dissociations in solution.

| Entry | Compound | $R_{CC}$ [Å] | $\Delta H$ [Kcal mol$^{-1}$] | 298K·$\Delta S$ [Kcal mol$^{-1}$] | $\Delta G$ [Kcal mol$^{-1}$] | Reference |
|---|---|---|---|---|---|---|
| 1 | 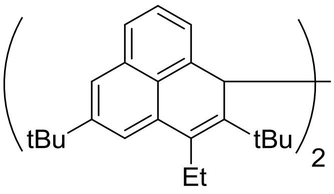 | | 14 | 15.5 | –1.5 | 15 |
| 2 | 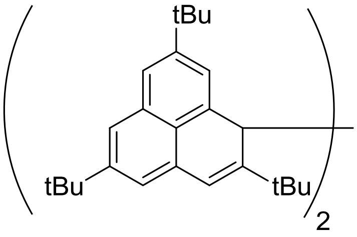 | | 9.5 | 10.7 | –1.2 | 15 |
| 3 | 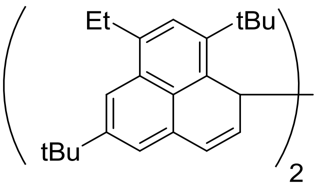 | | 14.5 | 14.9 | –0.4 | 16 |
| 4 | 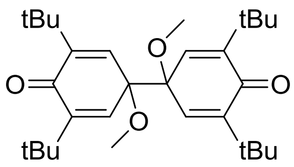 | 1.606 | 6.1 | 6.3 | –0.2 | 17 |
| 5 | 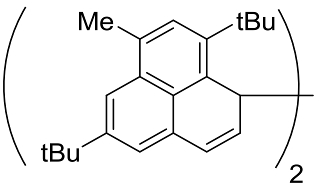 | | 12.6 | 11.6 | 1.0 | 16 |
| 6 | 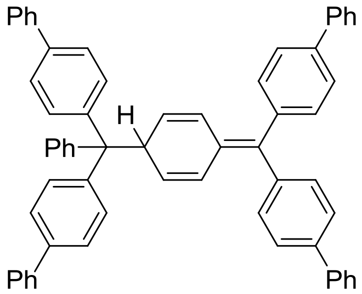 | | 6.8 | 4.8 | 2 | 18 |

Table S27 Continued…

| **Entry** | **Compound** | $R_{CC}$ [Å] | $\Delta H$ [Kcal mol$^{-1}$] | 298K·$\Delta S$ [Kcal mol$^{-1}$] | $\Delta G$ [Kcal mol$^{-1}$] | **Reference** |
|---|---|---|---|---|---|---|
| 7 | 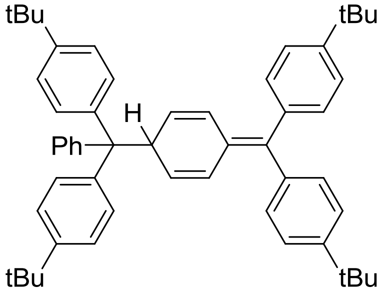 | | 8.2 | 5.1 | 3.1 | 18 |
| 8 | 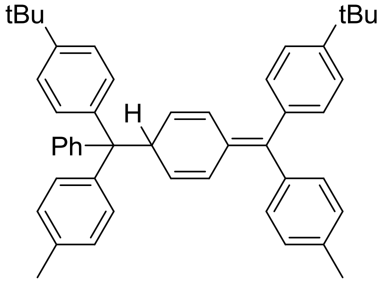 | | 10.2 | 6.0 | 4.2 | 18 |
| 9 | 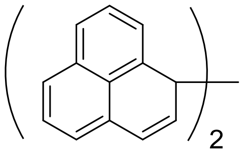 | | 10 | 5.4 | 4.6 | 15 |
| 10 | 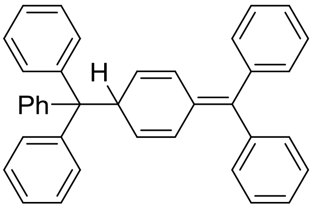 | 1.60 | 10.7 | 6.0 | 4.7 | 18 |
| 11 | 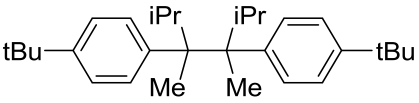 | | 34.8 | 4.9 | 29.9 | 19 |
| 12 | 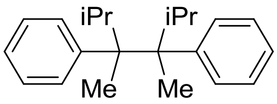 | | 35.1 | 4.3 | 30.8 | 19 |

Table S27 Continued…

| **Entry** | **Compound** | $\boldsymbol{R}_{\mathbf{CC}}$ [Å] | $\Delta\boldsymbol{H}$ [Kcal mol$^{-1}$] | **298K·**$\Delta\boldsymbol{S}$ [Kcal mol$^{-1}$] | $\Delta\boldsymbol{G}$ [Kcal mol$^{-1}$] | **Reference** |
|---|---|---|---|---|---|---|
| 13 | 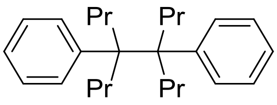 | | 34.9 | 3.9 | 31 | [19] |
| 14 | 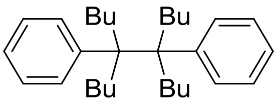 | 1.638 | 38.2 | 6.5 | 31.7 | [19] |
| 15 | 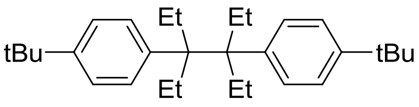 | | 40.4 | 7.9 | 32.5 | [19] |
| 16 | 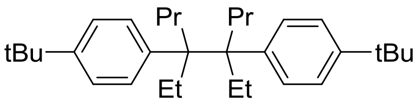 | | 38.1 | 5.5 | 32.6 | [19] |
| 17 | 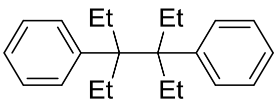 | 1.622 | 44.7 | 10.0 | 34.7 | [19] |
| 18 | 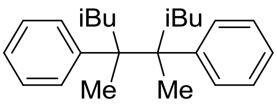 | | 40.4 | 5.4 | 35 | [19] |

Table S27 Continued…

| **Entry** | **Compound** | ***R*<sub></sub>** $R_{CC}$ [Å] | $\Delta H$ [Kcal mol$^{-1}$] | 298K·$\Delta S$ [Kcal mol$^{-1}$] | $\Delta G$ [Kcal mol$^{-1}$] | **Reference** |
|---|---|---|---|---|---|---|
| 19 | 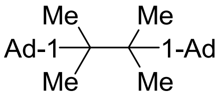 | 1.639 | 43.7 | 6.9 | 36.8 | 20 |
| 20 | 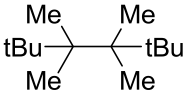 | | 44.0 | 5.3 | 38.7 | 21 |
| 21 | 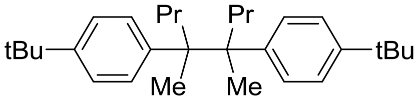 | | 44.1 | 5.3 | 38.8 | 19 |
| 22 | 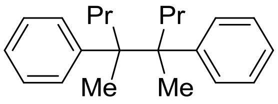 | | 45.6 | 6.4 | 39.2 | 19 |
| 23 | 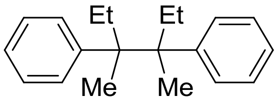 | | 48.4 | 7.2 | 41.2 | 19 |
| 24 | 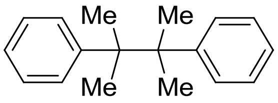 | 1.585 | 46.0 | 4.2 | 41.8 | 19 |

Table S27 Continued…

| Entry | Compound | $R_{CC}$ [Å] | $\Delta H$ [Kcal mol$^{-1}$] | 298K·$\Delta S$ [Kcal mol$^{-1}$] | $\Delta G$ [Kcal mol$^{-1}$] | Reference |
|---|---|---|---|---|---|---|
| 25 | 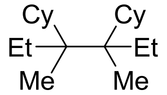  | | 52.1 | 6.7 | 45.4 | [21] |
| 26 | 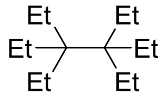  | | 51.0 | 4.2 | 46.8 | [21] |
| 27 | 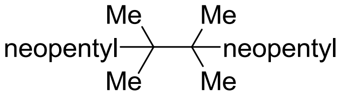  | | 57.8 | 6.1 | 51.7 | [21] |
| 28 | 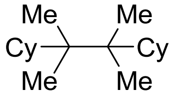  | | 61.2 | 8.0 | 53.2 | [21] |
| 29 | 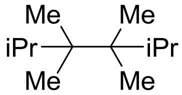  | | 62.2 | 7.8 | 54.4 | [21] |
| 30 | 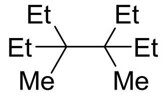  | 1.626 | 60.2 | 5.2 | 55 | [21] |

Table S27 Continued…

| Entry | Compound | $R_{CC}$ [Å] | $\Delta H$ [Kcal mol$^{-1}$] | 298K·$\Delta S$ [Kcal mol$^{-1}$] | $\Delta G$ [Kcal mol$^{-1}$] | Reference |
|---|---|---|---|---|---|---|
| 31 | 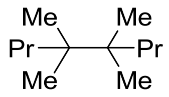 | | 57.8 | 2.2 | 55.6 | [21] |
| 32 | 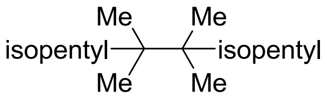 | | 58.3 | 2.4 | 55.9 | [21] |
| 33 | 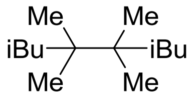 | | 60.7 | 4.6 | 56.1 | [21] |
| 34 | 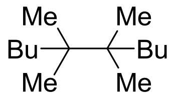 | | 59.0 | 2.7 | 56.3 | [21] |
| 35 | 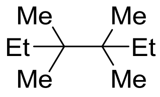 | | 62.1 | 3.3 | 58.8 | [21] |
| 36 | 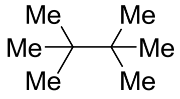 | 1.582 | 67.4 | 3.9 | 63.5 | [21] |

## References


1. M. J. Frampton, H. Akdas, A. R. Cowley, J. E. Rogers, J. E. Slagle, P. A. Fleitz, M. Drobizhev, A. Rebane and H. L. Anderson, *Org. Lett.*, 2005, **7**, 5365-5368.
2. T. A. V. Khuong, G. Zepeda, R. Ruiz, S. I. Khan and M. A. Garcia-Garibay, *Cryst. Growth Des.*, 2004, **4**, 15.
3. H. Sakurai, H. Umino and H. Sugiyama, *J. Am. Chem. Soc.*, 1980, **102**, 6837.
4. T.-A. V. Khuong, G. Zepeda, C. N. Sanrame, H. Dang, M. D. Bartberger, K. N. Houk and M. A. Garcia-Garibay, *J. Am. Chem. Soc.*, 2004, **126**, 14778-14786.
5. A. Ono, N. Suzuki and J. Kamimura, *Synthesis*, 1987, 736.
6. M. Stein and A. Rieker, *Tetrahedron Lett.*, 1975, **16**, 2123-2126.
7. B. Kahr, D. v. Engen and K. Mislow, *J. Am. Chem. Soc.*, 1986, **108**, 8305-8307.
8. M. J. Frisch, G. W. Trucks, H. B. Schlegel, G. E. Scuseria, M. A. Robb, J. R. Cheeseman, G. Scalmani, V. Barone, B. Mennucci, G. A. Petersson, H. Nakatsuji, M. Caricato, X. Li, H. P. Hratchian, A. F. Izmaylov, J. Bloino, G. Zheng, J. L. Sonnenberg, M. Hada, M. Ehara, K. Toyota, R. Fukuda, J. Hasegawa, M. Ishida, T. Nakajima, Y. Honda, O. Kitao, H. Nakai, T. Vreven, J. J. A. Montgomery, J. E. Peralta, F. Ogliaro, M. Bearpark, E. B. J. J. Heyd, K. N. Kudin, V. N. Staroverov, T. Keith, R. Kobayashi, J. Normand, K. Raghavachari, A. Rendell, J. C. Burant, S. S. Iyengar, J. Tomasi, M. Cossi, N. Rega, J. M. Millam, M. Klene, J. E. Knox, J. B. Cross, V. Bakken, C. Adamo, J. Jaramillo, R. Gomperts, R. E. Stratmann, O. Yazyev, A. J. Austin, C. P. R. Cammi, J. W. Ochterski, R. L. Martin, K. Morokuma, V. G. Zakrzewski, G. A. Voth, P. Salvador, J. J. Dannenberg, S. Dapprich, A. D. Daniels, O. Farkas, J. B. Foresman, J. V. Ortiz, J. Cioslowski and D. J. Fox, *Journal*, 2013.
9. R. M. Parrish, J. F. Gonthier, C. Corminboeuf and C. D. Sherrill, *J. Chem. Phys.*, 2015, **143**, 0511031-0051135.
10. D. A. Pittam and G. Pilcher, *J. Chem. Soc. Faraday Trans. 1*, 1972, **68**, 2224-2229.
11. J. M. Nicovich, C. A. Van Dijk, K. D. Kreutter and P. H. Wine, *J. Phys. Chem.*, 1991, **95**, 9890-9896.
12. M. V. Roux, M. Temprado, J. S. Chickos and Y. Nagano, *J. Phys. Chem. Ref. Data*, 2008, **37**, 1855-1996.
13. W. D. Good, *J. Chem. Thermodyn.*, 1972, **4**, 709-714.
14. P. W. Seakins, M. J. Pilling, J. T. Niiranen, D. Gutman and L. N. Krasnoperov, *J. Phys. Chem.*, 1992, **96**, 9847-9855.
15. D. Small, S. V. Rosokha, J. K. Kochi and M. Head-Gordon, *J. Phys. Chem. A*, 2005, **109**, 11261-11267.
16. V. Zaitsev, S. V. Rosokha, M. Head-Gordon and J. K. Kochi, *J. Org. Chem.*, 2006, **71**, 520-526.
17. J. M. Wittman, R. Hayoun, W. Kaminsky, M. K. Coggins and J. M. Mayer, *J. Am. Chem. Soc.*, 2013, **135**, 12956-12959.
18. W. P. Neumann, W. Uzick and A. K. Zarkadis, *J. Am. Chem. Soc.*, 1986, **108**, 3762-3770.
19. C. Rüchardt and H.-D. Beckhaus, *Angew. Chem. Int. Ed. Engl.*, 1985, **24**, 529-538.
20. M. A. F.-t. Meer, H.-D. Beckhaus, K. Peters, H.-G. v. Schnering and C. Rüchardt, *Chem. Ber.*, 1985, **118**, 4665.
21. R. Winiker, H.-D. Beckhaus and C. Rüchardt, *Chem. Ber.*, 1980, **113**, 3456-3476.
